# Palladium-Induced Temporal Internalization of MHC Class I Contributes to T Cell-Mediated Antigenicity

**DOI:** 10.3389/fimmu.2021.736936

**Published:** 2021-12-23

**Authors:** Koyu Ito, Takayuki Kanaseki, Serina Tokita, Toshihiko Torigoe, Noriyasu Hirasawa, Kouetsu Ogasawara

**Affiliations:** ^1^ Department of Immunobiology, Institute of Development Aging and Cancer, Tohoku University, Sendai, Japan; ^2^ Department of Pathology, Sapporo Medical University School of Medicine, Sapporo, Japan; ^3^ Academic Center, Sapporo Dohto Hospital, Sapporo, Japan; ^4^ Laboratory of Pharmacotherapy of Life-Style Related Diseases, Graduate School of Pharmaceutical Sciences, Tohoku University, Sendai, Japan

**Keywords:** metal allergy, palladium, MHC class I internalization, alternative peptide presentation, dental biomaterials

## Abstract

Palladium (Pd) is a widely used metal and extremely important biomaterial for the reconstruction of occlusions during dental restorations. However, metallic biomaterials can cause serious allergic reactions, such as Pd-related oral mucositis seen in dentistry. Metal allergy is categorized as a type IV allergy and we demonstrated that CD8 T cells play an important role in Pd allergy previously. As TCR of CD8 T cells recognizes MHC class I/peptide complex, the antigen specificity to this complex seems to be generated during Pd allergy. However, it remains unknown if Pd affects the MHC class I/peptide complex. In this study, we investigated the behavior of the MHC class I/peptide complex in response to Pd treatment. We found that PdCl_2_ treatment altered peptide presentation on MHC class I and that co-culture with Pd-treated DC2.4 cells induced activation of Pd-responsive TCR-expressing T cell line. Furthermore, PdCl_2_ treatment induced temporal MHC class I internalization and inhibition of membrane movement suppressed Pd-induced T cell-mediated antigenicity. These data suggest that Pd-induced MHC class I internalization is critical for generation of antigenicity through a mechanism including differential peptide loading on MHC class I, which results in Pd allergy.

## Introduction

Biomaterials contribute widely to the development of human therapeutics (1–3). Metals, in particular, are broadly useful in many fields due to their hardness, strength, durability, and workability. Palladium (Pd) is commonly used, including in dentistry as an extremely important metallic biomaterial for the reconstruction of occlusions. However, metallic biomaterials can cause allergy, and in dentistry Pd-related oral mucositis is a serious problem. In addition, the allergic response can also result in skin conditions, such as palmoplantar pustulosis (4, 5).

Metal allergy is categorized as a type IV allergy, which is mediated by T cells (6). An inherent conflict exists in T cell-mediated immune responses between the induction of tolerance and the activation of appropriate immune responses (7). Classically, T cell-mediated allergic reactions against foreign substances have been considered a process to eliminate exogeneous antigens phagocytosed and presented by antigen-presenting cells (APCs) (8). However, recent reports of the two major types of T cell-mediated delayed type hypersensitivity, metal allergy and drug hypersensitivity, suggest that antigenicity is acquired through various mechanisms, and the target of the resulting allergic reaction is not always exogeneous antigens phagocytosed by APCs (9–11). Classical understanding of metal or drug hypersensitivity was based on the concept that exogenous substances form haptenated antigens with self-proteins (10, 11). In this concept, haptenated antigens phagocytosed by APCs are cross-presented by MHC class I after undergoing processing (12). In other words, the peptide loading onto the MHC class I is derived from haptenated antigens. However, this hapten theory does not address all mechanisms of antigen induction for metals and drugs. Some metals, like beryllium, cobalt and nickel, can bind directly to MHC molecules and some drugs, such as abacavir, cause adverse effects related to the HLA haplotype of an individual (6, 9, 13–17). These direct associations between MHC molecules and metals/drugs result in altered antigenicity through conformational changes or peptide exchange, which in turn enable escape from tolerance and induction of antigen-specific T cell-immune responses.

Although it has been reported that both CD4 and CD8 T cells are responsible for the induction of Pd allergy (18–21), our previous study showed that repeated adoptive transfer of lymph node cells from Pd-treated mice skews the CD4/CD8 T cell-balance toward CD8 T cells. Thus, this suggests CD8 T cells play a more important role in Pd allergy than do CD4 T cells (22). Moreover, the TCR repertoires of CD8 T cells from Pd-treated mice were significantly biased, suggesting that the activation of CD8 T cells is antigen specific (21). Considering that TCR of CD8 T cells recognizes MHC class I in complex with peptide, the antigen specificity of CD8 T cells seems to be generated through events related to this complex. However, the detailed mechanisms underlying the generation of antigenicity in Pd allergy are not well understood. To this end, we investigated the behavior of the MHC class I/peptide complex in response to Pd treatment.

## Materials and Methods

### Ethics Statement

Mice were maintained under specific pathogen-free conditions, and all procedures were performed according to the protocols approved by the Institutional Committee for Use and Care of Laboratory Animals of Tohoku University, which was granted by Tohoku University Ethics Review Board (2019AcA-003). For collection of tissue samples, mice were sacrificed by cervical dislocation, and all efforts were made to minimize suffering.

### Mice

C57BL/6 mice (6 to 8-week-old females) were purchased from CLEA Japan. OT-I mice were purchased from Jackson Laboratories.

### Induction of Palladium Allergy

Palladium allergy was induced as described previously (23). In brief, mice were injected twice into both sides of the groin with 250 μl PBS containing 10 mM PdCl_2_ and 10 μg/ml LPS at an interval of seven days. Seven days after the 2^nd^ injection, mice were challenged by intradermal injection of 25 μl of 10 mM PdCl_2_ in PBS into both the left and right footpads.

### Generation of Pd-Responsive T Cells

Pd-responsive T cells were generated as described in a previous report (24). In brief, Pd allergy was induced in mice as described above. Twenty-four hours after challenge, inguinal and popliteal lymph nodes (LN) were collected. LN cells were cultured in the presence of 20 μM PdCl_2_ for 5 days. After washing, these cells were further co-cultured with irradiated (20 Gy) splenocytes in the presence of 20 μM PdCl_2_ and 100 U/ml recombinant IL-2 (Wako) for 5 days. Finally, LN cells were cultured in the presence of 100 U/ml IL-2 alone for 3 days. TCR repertoire analysis was performed as described previously (21).

### Cell Lines

The murine dendritic cell line DC2.4 (H-2K^b^, H-2D^b^) was purchased from Merck Millipore. Human CD8-expressing TG40 cells were a kind gifted from Dr. Kishi (Toyama University).

### Antibodies for Flow Cytometric Analysis

All antibodies used in flow cytometric analysis were purchased from Biolegend: FITC-conjugated anti-H-2K^b^D^b^ antibody (clone 28-8-6), FITC-conjugated anti-H-2K^b^ antibody (clone AF6-88.5), APC-conjugated anti-H-2D^b^ antibody (clone KH95), APC-conjugated anti-CD8α antibody (clone 53-6.7), APC-conjugated antibody for H-2K^b^ bound to SIINFEKL (clone 25D1.16), FITC-conjugated anti-CD69 antibody (clone H1.2F3) and APC-conjugated anti-TCRβ antibody (clone H57-597).

### Flow Cytometric Analysis

DC2.4 (2 x 10^5^ cells) were cultured with 200 μM PdCl_2_ in AIM-V medium (Gibco) at 37°C for 0 15, 30, 60, and 120 min. Cells were then washed with FACS buffer (0.5% BSA, 0.5 mM EDTA, 0.09% NaN_3_/PBS) and stained with FITC-anti H-2K^b^D^b^ antibody at 4°C for 20 min. All analyses were performed on FACS Canto II (BD Biosciences) with FlowJo software (TOMY digital biology). In some experiments, prior to staining cells were fixed and permeabilized with BD Fix/Perm Buffer according to the manufacturers’ instruction.

### Analysis of Antigen Recognition by PdCl_2_-Treated Cells

Mice were treated intravenously with 1 mg OVA protein (SIGMA) 50 μg OVA_257-264_ (SIINFEKL) (Iwaki Bioservice). Twelve hours after injection, mice were administrated intraperitoneally with 300 μl of 10 mM PdCl_2_. Twelve hours after PdCl_2_ treatment, spleens were collected and lysed with ACK lysis buffer (155 mM NH_4_Cl, 10 mM KHCO_3_, 1 mM EDTA), and then washed. Splenocytes were irradiated (20 Gy) for use as antigen presenting cells. For preparation of responder cells, splenocytes was obtained from OT-I mice. These cells were labeled with 5 μM CFSE (Dojindo). Antigen-presenting cells and responder cells were then co-cultured for 48 hours. CFSE dilution in CD8α+ cells were evaluated by flow cytometric analysis to assess proliferation in response to OVA antigen recognition.

### Peptide Alteration Assay by 25D1.16

DC2.4 cells were pulsed with 5 ng/ml OVA_257-264_ (SIINFEKL) (Iwaki Bioservice) in 10% fetal bovine serum (FBS)/RPMI for 1 hour at 26°C and washed with PBS. Then cells were treated with 200 μM PdCl_2_ for 2 hours at 26°C. In some experiments, cells were washed and recovered in 10% FBS/RPMI for 1 hour. Finally, cells were stained with antibodies against H-2K^b^ or 25D1.16.

### Western Blot Analysis

DC2.4 cells were treated with 25 U/ml recombinant mouse IFN-γ (Peprotech) for 48 hours. Cells were then treated with 100 μM PdCl_2_ for 30 min followed by lysis with RIPA buffer (50 mM Tris-HCl (pH 7.4), 150 mM NaCl, 1% Triton X-100, 1% Sodium deoxycholate, 0.1% SDS) supplemented with protease inhibitor cocktail cOmplete™ (Roche) and kept on ice for 30 min. Lysates were centrifugated for 30 min at 4°C. Proteins in supernatant were quantified by MicroBCA protein assay kit (ThermoFicher Scientific). Supernatants were then used for SDS-PAGE, electrotransferred onto polyvinylidene difluoride membranes (Millipore), and membranes probed with the indicated primary antibodies for MHC class I, H-2K^b^ and H-2D^b^ (Abcam), followed by HRP-conjugated secondary antibodies. Membranes were then washed, and bands visualized with the enhanced chemiluminescence detection system (ECL) by Chemilumi One L (Nacalai Tesque). Band Intensity was quantified using Image J software.

### Confocal Microscopy

DC2.4 cells were treated with 1 μg/ml SIINFEKL for 1 hour at 37°C and then cells were cultured in the presence or absence of PdCl_2_ for 30 min. Cells were fixed with 1% paraformaldehyde/PBS for 10 min at 4°C, and then permeabilized with 0.1% Triton X100 for 10 min at room temperature. Cells were stained with antibody 25D1.16 prior to analysis with a TCS SP8 microscope (Leica).

### MHC Ligandome Analysis Using Mass Spectrometry

DC2.4 cells were treated with and 10 ng/ml LPS with or without 50 μM PdCl_2_ (depicted as (+) or (-) PdCl_2_ in the figures, respectively) for 18 hours at 37°C. Cells were then washed with PBS and harvested using a cell scraper. Approximately 2 x10^9^ cells were used in the analysis. MHC ligands were isolated and sequenced using mass spectrometry as previously described (25). Briefly, peptide-MHC complexes in samples were captured by affinity chromatography using monoclonal antibodies (Y-3 for K^b^ and 28-14-8S for D^b^). The MHC ligands were eluted, desalted, and injected into LC-MS/MS (Easy-nLC 1000 system and Q Exactive Plus, Thermo). In mass spectrometry, data were acquired with a data-dependent top 10 method. Survey scan spectra were acquired at a resolution of 70,000 at 200 m/z with an AGC target value of 3e6 ions and a maximum IT of 100 ms, ranging from 350 to 2,000 m/z with charge states between 1+ and 4+. MS/MS resolution was 17,500 at 200 m/z with an AGC target value of 1e5 ions and a maximum IT of 120 ms. MS/MS data were searched against the Swiss-Prot database using the Sequest HT along with the Percolator algorithm on the Proteome Discoverer platform (Thermo). The tolerance of precursor and fragment ions was set at 10 ppm and 0.02 Da, respectively, and no specific enzyme was selected for the search. Concatenated target-decoy selection was validated based on q-values, and a false discovery rate (FDR) of 0.05 was used in the Percolator node as a peptide detection threshold. Only the 8-11 mer (K^b^) and 8-12 mer (D^b^) peptides with IC50 (NetMHC-4.0) < 5,000 were counted as natural MHC ligands. Ligandome analysis were performed twice in each condition and whole peptides in the identified in both experiments were used for analysis. Complete list used in analysis are shown in **Supplementary Tables 1**–**4**. Peptide analysis was performed by R venn-diagram package (ver. 3.5.2).

### Construction of TCR-Expressing Vector

To generate a TCR α and β co-expression plasmid, the C-region of TCRα and β chain were first amplified from cDNA from wild type splenocytes and ligated into the pMX-IP vector (Cell Biolabs). Pd-reactive T cells were collected, RNA extracted using the RNeasy mini kit (QIAGEN), and then cDNA synthesized using Superscript III (ThermoFisher Scientific). The cDNA was then used to generate TCRα and β libraries as described previously (21). Each TCR library was mixed with a forward primer consisting of the annealing site of the adapter DNA (5’- AGCTAGTTAATTAAGGATCCTGATCACCGGACAGGAATTCC -3’) and 20 bps overlapping the pMX-IP vector and a reverse primer specific for the C-region of the TCR α and β (for TCRα 5’- TGGTACACAGCAGGTTCTGGGTTC-3’, for TCRβ 5’- CAAGGAGACCTTGGGTGGAGTCAC-3’). Next, TCR fragments were amplified by PCR. PCR fragments were ligated into the pMX-IP vector digested with BamHI and XhoI (TakaraBio) by mix with NEBuilder HiFi DNA Assembly Master Mix (New England Bio. Lab.) and incubation at 50°C for 15 min. The resulting vectors were transformed into NEB5α competent cells. Transformed cells were spread on LB agar plates and incubated for 16 hours at 37°C. Colonies were then collected, and mixed plasmids were purified using a QIAGEN miniprep kit.

### Establishment of TCRα and β-Expressing TG40 Cell

Plat E cells (1 x10^6^ cells) were seeded on 6-well plates, then 1.25 μg of each TCRα and β/pMX-IP plasmid was transfected using polyethylene imine (Polysciences). Two days after transfection, supernatant was collected, and 4 μg/ml of polybrene was added to the supernatant. Then, TG40 cells (2x10^5^ cells) were mixed with supernatant and centrifuged at 1,800 g at 32°C for 60 min and seeded in 24 well plates. Two days after transduction, cells were selected by 1 μg/ml puromycin (Wako). After 6 days of selection, TCRβ-expressing cells were sorted by FACS Aria III (BD Biosciences).

### Activation of TCR-Expressing TG40 in Response to Pd

DC2.4 cells (1x10^4^ cells) in 10% FBS/RPMI1640 (Wako) were seeded in 96-well flat bottom plates (FALCON). Six hours after cell seeding, cells were treated with 100 μM PdCl_2_ and 25 U/ml recombinant mouse IFN-γ (Peprotech) for 18 hours. Then cells were washed with PBS, and co-cultured with TCR- transduced TG40 (2 x10^4^ cells) for 24 hours. Expression of CD69 on TCRβ positive cells was analyzed by flow cytometry to examine activation of TG40 cells in response to Pd. In some experiments, prior to co-culture with TG40 cells, peptides on MHC class I were stripped as described previously (26).

### Statistical Analyses

**Figure 1:**
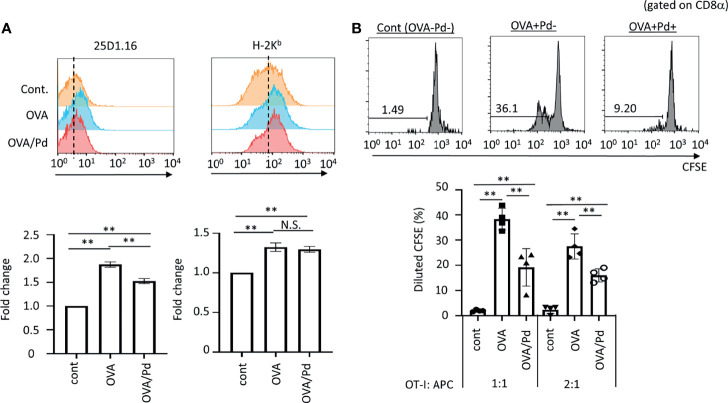
Effect of PdCl_2_ on antigenicity of splenocyte of mice treated with OVA. **(A)** Effect of 25D1.16 antibody binding in splenocyte of mice treated with PdCl_2_. Mice were injected with OVA protein and 12 hours after injection, PdCl_2_ was injected intraperitoneally. After 12 hours, splenocytes were then analyzed by using antibody clone 25D1.16. Orange histograms represent non-treated cells. Blue histograms represent splenocytes from mice treated with OVA protein. Red histograms represent splenocytes from mice treated with OVA proteins and PdCl_2_. Bottom graphs show fold change of MFI of indicated antibodies to control. Error bars indicate ± S.D., of three independent experiments. **(B)** Antigenicity of Pd-modulated OVA-loading splenocytes. Antigen-presenting cells (APC) were prepared from mice treated with OVA protein and PdCl_2_ described as **(A)**. After splenocytes were irradiated, these cells were cocultured with CFSE-labeled OT-I cells. (top) Histograms indicate representative results of CFSE dilution gated on CD8α. (bottom) The graphs show the mean % diluted CFSE. Error bars indicate ± S.D. (n=3). Data are representative of three independent experiments. One-way ANOVA with Tukey’s multiple comparison. ** denote p<0.05 and p<0.01 compared to the control. N.S., not significant.

**Figure 2:**
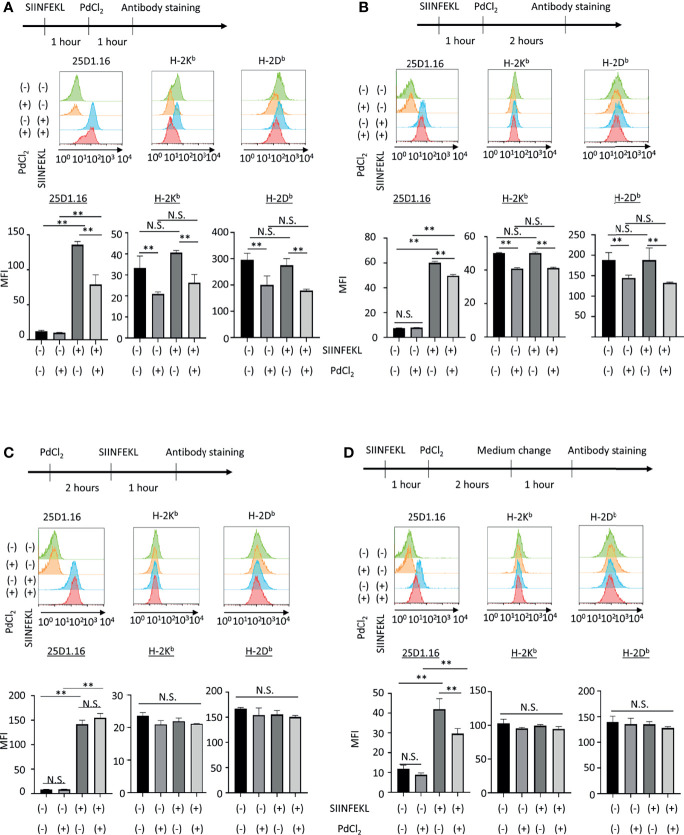
*In vitro* analysis of the effect of PdCl_2_ treatment on OVA peptide presentation by MHC class I. Alternative peptide presentation study in DC2.4 cells. DC2.4 cells were pulsed with SIINFEKL for 1 hour and then cells were treated with PdCl_2_ for **(A)** 1 hour and **(B)** 2 hours. **(A, B)** Antibody staining was preformed just after Pd treatment. **(C)** Effect of PdCl_2_ in antibody binding. DC2.4 cells were treated with PdCl_2_ for 2 hours and cells were pulsed with SIINFEKL peptide for 1 hour and binding of antibody for SIINFEKL on H-2K^b^ (25D1.16), H-2K^b^ and H-2D^b^. **(D)** SIINFEKL-treated DC2.4 cells were treated with PdCl_2_ for 2 hours and cultured in PdCl_2_-free media for 1 hour. Then, cells were stained with antibodies. Histograms shows (top) 25D1.16 antibody binding and (bottom) H-2K^b^ expression. The graph shows MFI of antibody binding. Error bars indicate ± S.D. (triplicated wells). Data are representative of three independent experiments. Statistical difference was determined One-way ANOVA with Tukey’s multiple comparison. ** denote p<0.05 compared to the control. N.S., not significant.

**Figure 3:**
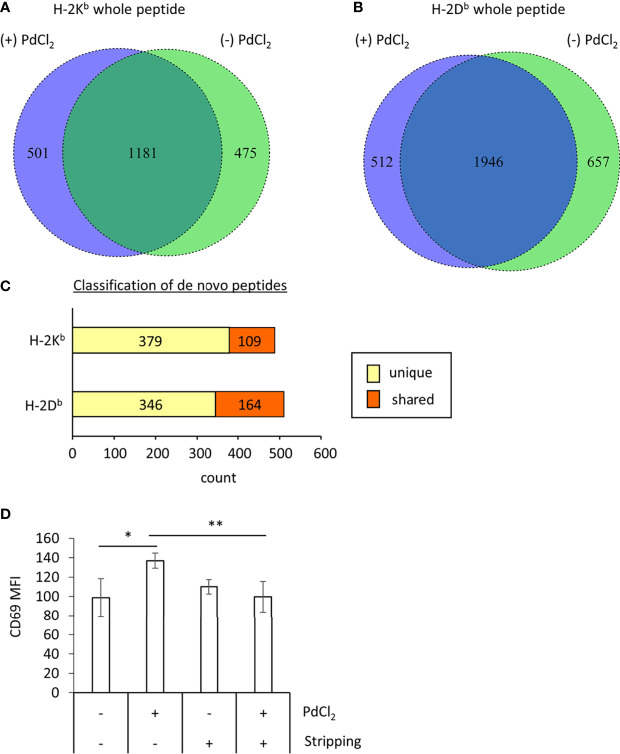
MHC class I ligandome analysis of PdCl_2_-treated cells. DC2.4 cells were treated with PdCl_2_ and LPS (depicted as (+) PdCl_2_) or LPS alone (depicted as (-) PdCl_2_) for 18 hours before cells were lysed and H-2K^b^ and anti-H-2D^b^ immunoprecipitated, followed by analysis of presented peptides by LC-MS/MS. Ligandome analysis were performed twice for each condition and whole peptides in the identified in both experiments were used for analysis. Peptide lists were depicted in **Supplementary Tables 1**–**4**. **(A, B)** Comparison of peptide sequence between the presence and absence of PdCl_2_ (**Supplementary Tables 1**–**4** “sequence” column). Differential presentation of peptides by **(A)** H-2K^b^ and **(B)** H-2D^b^ in cells treated with (purple area) and without (green area) PdCl_2_. **(C)** Comparison of source proteins of peptides presented by H-2K^b^ and H-2D^b^ following PdCl_2_ treatment of cells. Designated proteins of purple area in **(A, B)** were compared with the protein list in the absence of PdCl_2_. “Unique” means proteins which were not listed in the absence of PdCl_2_ whereas “Shared” means proteins which also listed in the without culture of PdCl_2_. **(D)** CD69 expression on Pd-TCR/TG40 cells co-cultured with PdCl_2_-treated DC2.4 cells. DC2.4 cells were cultured with PdCl_2_ in the presence of recombinant mouse IFN-γ for 18 hours. After washing, DC2.4 cells were cocultured with Pd-TCR/TG40 cells and CD69 expression on TG40 was examined. The graph shows the MFI of CD69 expression on TCRβ-expressing cells. Error bars denote ± S.D. (quadruplicated samples, three independent experiments). Statistical differences were determined by one-way ANOVA with Tukey’s multiple comparison. ** and * denote p<0.05 and p<0.01compared to the control, respectively.

**Figure 4:**
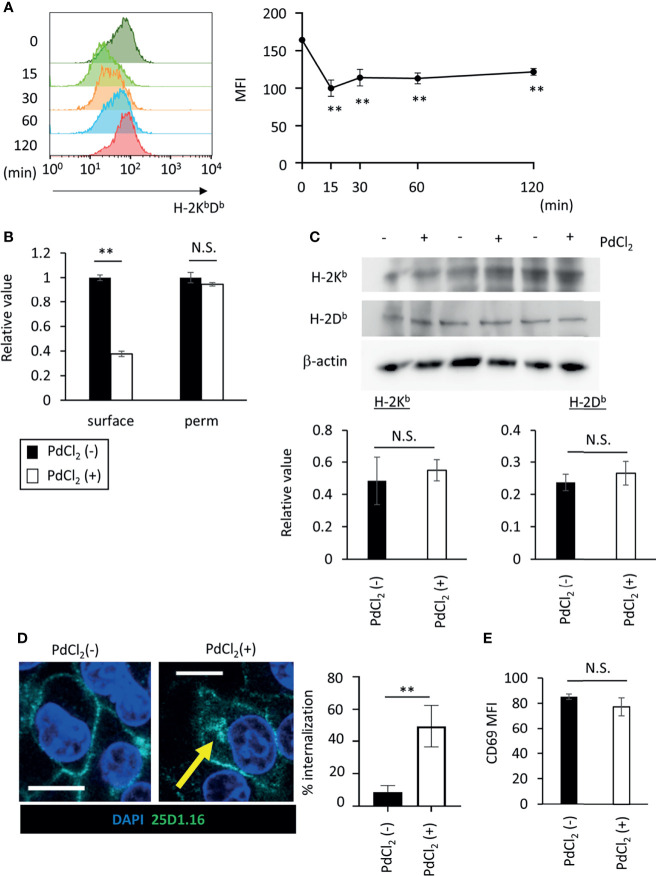
PdCl_2_ treatment induces temporal internalization of MHC molecules. **(A)** Surface MHC class I expression after PdCl_2_ treatment. DC2.4 cells were treated with PdCl_2_ and cultured for the indicated times (0, 15, 30, 60, and 120 min) and cells were stained for surface MHC class I. Representative histograms of MHC class I from four independent experiment (left panels). The graphs show MFI of MHC molecules (right panels). Error bars denote ± S.D. (triplicated samples, four independent experiments). Statistical difference was determined One-way ANOVA with Tukey’s multiple comparison. ** denote p<0.05 compared to the control. **(B)** Flow cytometric analysis of total MHC class I (H-2K^b^D^b^) in permeabilized cells. Thirty minutes after Pd treatment, DC2.4 cells were fixed and permeabilized and subsequent staining with MHC class I. The graph shows relative value of MFI. MFI of untreated cells was set at 1. **(C)** Western blot analysis of MHC class I in DC2.4 cells treated with 25 U/ml rmIFNγ for 48 hours, and then treated with PdCl_2_ for 30 min. Graphs show the relative value of H-2K^b^ and H-2D^b^ to β-actin. (triplicated samples, representative data from 2 independent experiments) **(B, C)** Statistical difference was determined unpaired two-sided Student’s t-test. N.S. means not significantly difference. **(D)** Confocal microscopic analysis of internalization of MHC class I in response to PdCl_2_. DC2.4 cells were cultured with 1 μg/ml SIINFEKL peptide and cells were treated with PdCl_2_ for 30 min. Cells were then fixed, permeabilized, and stained with DAPI (blue) and 25D1.16 antibody (green). Arrow indicates the intracellular SIINFEKL-loading H-2K^b^. Graph shows % of the mean internalization in the four fields. **(E)** DC2.4 cells were treated with PdCl_2_ in the presence or absence of 0.25% NaN_3_, and then these cells were co-cultured with Pd-TCR/TG40 cells for 24 hours followed by evaluation of CD69 expression on TCRβ−expressing cells as described in **Figure 3D**. The graph shows the MFI of CD69 expression on TCRβ-expressing cells. Error bars denote ± S.D. (quadruplicated samples, three independent experiments). Statistical differences were determined using unpaired two-sided Student’s t-test. N.S. means not significantly difference.

All data are presented as mean ± S.D. Significance of difference between two groups was determined using unpaired two-sided Student’s t-test (**Figures 4B**–**E** and **Supplementary Figure 3C**) and one-way ANOVA with Tukey’s multiple comparison (**Figures 1A, B**, **2**, **3D**, **4A** and **Supplementary Figure 1**, **2**, **4**, **5**). * and ** denote p<0.05 and p<0.01 compared to the control. N.S., not significant. All analyses were performed using Graphpad Prism 8 (GraphPad).

## Results

### PdCl_2_ Treatment Affects Antigenicity by MHC Class I

First, we asked whether Pd-treatment affects MHC class I/peptide complexes. To examine this, mice were injected with ovalbumin (OVA) protein and 12 hours after injection, PdCl_2_ was injected intraperitoneally. After 12 hours, splenocytes were then analyzed by using antibody clone 25D1.16, which recognizes MHC class I H-2K^b^-loading SIINFEKL, an OVA-derived 8-mer peptide. Splenocytes from mice injected with OVA alone exhibited increased 25D1.16 binding as compared with non-treated mice. Interestingly, splenocytes from mice treated with OVA and PdCl_2_ exhibited reduced binding of 25D1.16 (**Figure 1A**, left panel). In contrast, the total H-2K^b^ level was comparable regardless of PdCl_2_ treatment (**Figure 1A**, right panel) with H-2K^b^ equally upregulated after OVA treatment in the presence and absence of PdCl_2_. It has been reported that commercial OVA protein contains LPS (27) and thus, this H-2K^b^ upregulation is thought to be the result of LPS contamination. Therefore, we performed *in vivo* experiment using SIINFEKL peptide (**Supplementary Figure 1**). When we used SIINFEKL peptide, we found that splenocytes from mice treated with SIINFEKL and PdCl_2_ exhibited reduced binding of 25D1.16 as compared with group of SIINFEKL alone (**Supplementary Figure 1A**, left panel). In contrast, the total H-2K^b^ level was comparable regardless of PdCl_2_ treatment (**Supplementary Figure 1A**, right panel). Next, we examined whether reduction of 25D1.16 binding was resulted in attenuation of CD8 T cell recognition. To test this, we used OT-I mice, which are OVA_257-264_-specific TCR transgenic mice (28). Antigen-presenting cells (APC) were prepared from mice treated with OVA protein and PdCl_2_ as described above. These irradiated APCs were then cocultured with CFSE-labeled OT-I cells. APCs obtained from mice treated with OVA protein alone induced robust OT-I proliferation (**Figure 1B**), while cells obtained from OVA and PdCl_2_-treated mice exhibited reduced OT-I proliferation (**Figure 1B**). We also found that reduction of OT-I proliferation by splenocyte of SIINFEKL/PdCl_2_-treated mice as compared with that of SIINFEKL alone treated mice (**Supplementary Figure 1**). These results suggested three possibilities as follows; (1) PdCl_2_ treatment reduced OVA peptide presentation on H-2K^b^, (2) there is less of SIINFEKL-H-2K^b^ available for recognition by antibody/OT-I cells or (3) PdCl_2_ changed SIINFEKL-H-2K^b^ (directly or indirectly) whilst SIINFEKL presentation is maintained.

Next, we also performed *in vitro* analysis using DC2.4 cells, a C57BL/6 (H-2K^b^, H-2D^b^) mouse-derived dendritic cell line (**Figure 2**). DC2.4 cells were treated with SIINFEKL peptide, and then cultured in the presence of PdCl_2_ for 1 hour or 2 hours. These cells were stained with 25D1.16 and antibody for H-2K^b^. Consistent with **Figure 1A**, SIINFEKL-treated DC2.4 cells exhibited increased binding of 25D1.16 antibody, and this effect was reduced by PdCl_2_ treatment for 1 hour or 2 hours (**Figure 2A, B**). However, in contrast with **Figure 1A**, total H-2K^b^ expression was also reduced after PdCl_2_ treatment (**Figures 2A, B**). In addition, H-2D^b^ expression, which was not relevant to SIINFEKL loading, also reduced as well as H-2K^b^ after PdCl_2_ treatment (**Figures 2A, B**). To examine the effect on antibody recognition by PdCl_2_ treatment, DC2.4 cells were treated with PdCl_2_ before SIINFEKL treatment, and then cells were stained with antibodies. As shown in **Figure 2C**, pretreatment with PdCl_2_ did not affect 25D1.16 binding. This data suggested that PdCl_2_ treatment did not affect recognition site of these antibodies in our experimental condition. To examine whether the reduction of 25D1.16 binding was the result due to MHC class I down-regulation, Pd-treated DC2.4 cells were cultured in the absence of PdCl_2_ for an additional 60 min to aim to recover MHC class I expression. In this condition, we found that 25D1.16 binding was reduced whereas H-2K^b^ level was comparable regardless cells were pre-cultured with PdCl_2_ or not (**Figure 2D**). These data collectively suggested that PdCl_2_ treatment alters MHC class I/peptide complexes. Thus, to further analyze the mechanism through which Pd treatment alters peptide presentation by PdCl_2_, we used DC2.4 cells.

### PdCl_2_ Treatment Induces Alternative Peptide Presentation on MHC Class I

Next, to examine the mechanism underlying the reduction of 25D1.16 antibody binding in DC2.4 cells as shown in **Figure 2**, we focused on loading peptide on MHC class I molecules, H-2K^b^ and H-2D^b^ after PdCl_2_ treatment. For these experiments we performed MHC class I ligandome analysis (25). As described in the Materials and Methods, DC2.4 cells were treated with PdCl_2_ prior to immunoprecipitation of MHC class I (H-2K^b^ and H-2D^b^) and identification of the presented peptides by LC-MS/MS (**Figure 3** and **Supplementary Tables 1**–**4**). Then, we compared peptide list on H-2K^b^ and H-2D^b^ between the presence or absence of PdCl_2_, respectively (Comparison of “Sequence” column in **Supplementary Tables**). When cells were treated with PdCl_2_, 501 and 512 peptides were differentially loaded on H-2K^b^ and H-2D^b^ as compared with the absence of PdCl_2_, respectively (**Figures 3A, B** purple area), indicating that PdCl_2_ treatment affected alternative peptide loading on H-2K^b^ and H-2D^b^.

Furthermore, we analyzed source proteins from which the PdCl_2_ treatment-induced peptides were derived (purple area in **Figures 3A, B**). The analysis steps show as follow: (1) Protein name was referenced from peptide sequence by Swissprot. (2) We extracted proteins which was designated by above 501/512 peptides induced by PdCl_2._ (3) We examined whether these proteins were found in the list without PdCl_2_. We found that 379 and 346 source proteins in H-2K^b^ and H-2D^b^, respectively, were not listed on the absence of PdCl_2_, these results indicate that these peptides were derived from unique source protein (**Figure 3C**, yellow bar). On the other hand, 109 and 164 proteins bound to H-2K^b^ and H-2D^b^, respectively, were shared between presence and absence of PdCl_2_ (**Figure 3C**, red bar).

To demonstrate the importance of differential peptide loading with T cell stimulation, we performed a peptide stripping experiment as described previously, with some modifications (26). First, the effect of peptide stripping was examined using antibody 25D1.16 and OVA_257_-pulsed DC2.4 cells (**Supplementary Figure 2**). Binding of antibody 25D1.16 to SIINFEKL-pulsed DC2.4 cells was reduced by peptide stripping (**Supplementary Figure 2A**). Furthermore, CD69 expression on OT-I TCR-expressing TG40 cells was also reduced by coculture with peptide-stripped cells (**Supplementary Figure 2B**).

To examine whether the differential peptide presentation caused by PdCl_2_ induces activation of Pd-responsive T cells, Pd-responsive T cells were established using LNs from mice sensitized and challenged with PdCl_2_ as reported previously, with some modifications (24). After continuous and low dose Pd treatment, oligoclonal T cells were successfully established (**Supplementary Figures 3A, B**). In these cells, we found that highest frequent of TCR repertoire was TRAV16D/DV11-3-CAMRAYANKMIF-TRAJ47-03 and TRBV13-3-01-CASSDRTTNSDYTF-TRBJ1-2-01. Then RNA obtained from these cells was used to construct a TCR expression vector. These expression plasmids were then retrovirally transduced into TG40 cells (T cell hybridoma cell line lacking TCRα and TCRβ) and after selection with puromycin, TCRβ-expressing TG40 cells were sorted. Following co-culture with PdCl_2_-pretreated DC2.4 for 24 hours, CD69 expression was analyzed as a marker of TG40 activation. We found that TG40 cells expressing the TCR library obtained from Pd-sensitized mice upregulated CD69 expression in response to PdCl_2_-treated DC2.4 cells (**Figure 3D**). However, TG40 cells which express TCR library from lymph node of naïve mice did not respond to PdCl_2_-treated DC2.4 cells (**Supplementary Figure 3C**).

Thus, we examined the effect of peptide stripping after PdCl_2_ treatment and co-culture with Pd-responsive T cells. We confirmed that CD69 expression on Pd-responsive TG40 cells was reduced following co-culture with peptide stripped PdCl_2_-treated DC2.4 cells (**Figure 3D**, right 2 columns). These results suggest that the reason for the reduction in 25D1.16 binding following PdCl_2_ treatment is due to alternative peptide loading on MHC class I.

### MHC Class I Is Internalized Following PdCl_2_ Treatment, Resulting in Alteration in Antigenicity

As shown in **Figure 2A**, Pd-treatment temporally reduces cell-surface expression of H-2K^b^. To explore the behavior of MHC class I in response to PdCl_2_ treatment, we analyzed MHC class I expression on the cell surface in response to PdCl_2_ treatment. DC2.4 cells were treated with PdCl_2_ and cultured for the indicated times (0, 15, 30, 60, and 120 min), and subsequent staining for surface MHC class I. PdCl_2_ treatment resulted in a reduction of surface MHC class I (H-2K^b^D^b^) within 15 min after which surface expression gradually recovered, albeit only partially, until 120 min (**Figure 4A**). Next, we assessed whether the reduction in MHC was due to degradation or internalization. To examine the level of MHC class I in PdCl_2_-treated cells under permeabilizing conditions, DC2.4 cells were treated with PdCl_2_ for 30 min, then cells were permeabilized and stained with antibodies for MHC class I. While surface MHC class I was reduced in the presence of PdCl_2_ under non-permeabilizing conditions, total MHC expression in cells treated with PdCl_2_ was comparable with that of non-treated cells (**Figure 4B**). In addition, western blot analysis confirmed that MHC class I levels of PdCl_2_-treated cells are comparable with that of non-treated cells (**Figure 4C**). These data suggest that PdCl_2_ treatment induces MHC class I internalization, and not degradation. Next, we followed the movement of MHC class I after PdCl_2_ treatment. To this end, we used the 25D1.16 antibody to stain cells treated with SIINFEKL peptide prior to fixation and permeabilization. As shown in **Figure 4D**, surface staining of 25D1.16 was observed regardless of PdCl_2_ treatment, but only following PdCl_2_ treatment was staining also observed around the perinuclear area (**Figure 4D**), which has been reported as the recycling center (29). These results indicated that PdCl_2_ treatment induced temporal internalization of MHC class I.

Finally, to examine the significance of MHC class I internalization on alternative peptide loading, DC2.4 cells were treated with PdCl_2_ and LPS in the presence of sodium azide (NaN_3_), which inhibits internalization of surface molecules. We found that NaN_3_ treatment partially suppresses MHC class I internalization in response to PdCl_2_ treatment (**Supplementary Figure 4**). As a control experiment, we examined whether NaN_3_ treatment affect antigenicity of exogenously added antigen. To this end, NaN_3_-treated DC2.4 cells were pulsed with SIINFEKL, and then cells were co-cultured with OT-I TCR/TG40 and CD69 on TG40 cells was analyzed. As shown in **Supplementary Figure 5**, activation of OT-1/TG40 by NaN_3_-treated DC2.4 that had been exogenously added with SIINFEKL was comparable with non-NaN_3_ treated SIINFEKL-loading DC2.4 cells. The result suggested that NaN_3_ treatment affected inhibition of membrane movement rather than cell metabolism. Then, to examine the antigenicity of PdCl_2_-treated DC2.4 cells in inhibition of membrane movement, DC2.4 cells were treated with or without PdCl_2_ in the presence NaN_3_, and then these cells were co-cultured with Pd-responsive TG40 cells. We found that upregulation of CD69 expression on Pd-responsive TG40 cells, as shown in **Figure 3D**, is suppressed by NaN_3_-treated DC2.4 cells (**Figure 4E**). Thus, MHC class I internalization by PdCl_2_ is crucial for alternative peptide loading on MHC class I.

## Discussion

In this study, we show that Pd treatment affects MHC class I/peptide complexes by altering MHC class I peptide loading. The mechanism underlying this is the Pd-induced temporal internalization of MHC class I. Under normal conditions, cell surface MHC class I is spontaneously internalized and re-expressed (30), and this intracellular trafficking contributes to stable surface expression of MHC class I and presentation of self-antigens for monitoring of self (31). Here, we show for the first time that a metal, Pd, induces MHC class I internalization in the process of generating antigenicity.

PdCl_2_ treatment induced alternative peptide loading on both H-2K^b^ and H-2D^b^ (**Figures 3A, B**) and PdCl_2_-treated DC2.4 cells can activate Pd-responsive TCR-expressing TG40 cells (**Figure 3D**). The data suggested that alternative peptide loading induced by PdCl_2_ treatment exert T cell-mediated antigenicity. In this study, we found that highest frequent TCR from *in vitro* low dose PdCl_2_ stimulation was TRAV16D/DV11-3-CAMRAYANKMIF-TRAJ47-03 and TRBV13-3-01-CASSDRTTNSDYTF-TRBJ1-2-01 (**Supplementary Figure 3**). Although we detected TRAV7-TRAJ22 as the previous *in vivo* experiments (21), this TCR is not major repertoire in this study. There was a possibility that differential peptides might be presented by splenocyte in mice as compared with lymph node cells. Further analysis of the source proteins of these alternatively loaded peptides revealed that majority of source proteins were derived from proteins that differed from those of non-treated cells (**Figure 3C**). Abacavir is known to bind the F-pocket of the peptide-binding groove of HLA-B*57:01 resulting in alteration of the MHC-presented self-peptide repertoire (32, 33). Therefore, it is possible that PdCl_2_ also affects the receiving groove of MHC class I, resulting in alternative peptide binding. In addition, 109 and 164 protein antigens that bound H-2K^b^ and H-2D^b^, respectively, were shared between the presence and absence of PdCl_2_ (**Figure 3C**). A previous study reported that Au (III) causes alteration of a model antigen to cryptic peptides (34). Thus, our results suggested that PdCl_2_ treatment also affected alternative peptide loading through processing of antigenic proteins. To discern between these possibilities, a novel Pd indicator similar to Newport green which is an indicator of Ni, will be needed (35). Studies to further determine the intracellular localization of Pd will help reveal whether Pd binds to MHC class I and/or peptides during processing. In addition, as many metals exert cellular toxicity, it has a possibility that PdCl_2_ treatment constitutes stress for the cells even if low dose PdCl_2_. Thus, the relationship among temporal internalization of MHC class I, alternative peptide loading, and cellular stress by PdCl_2_ will be interesting target for detailed understanding of antigenicity generation during Pd allergy.

As shown in **Figure 4A**, PdCl_2_ treatment reduces surface expression of MHC class I, but does not affect the total cellular level of MHC class I (**Figures 4B, C**). This indicates that surface MHC class I is internalized in response to Pd treatment. Interestingly, PdCl_2_-induced antigenicity was suppressed by inhibition of membrane movement (**Figure 4E**). While these results indicate that PdCl_2_ treatment induces MHC class I internalization and alternative peptide loading, it should be noted that 0.25% NaN_3_ treatment did not completely suppress this effect (**Supplementary Figure 4**). Overdose treatment of NaN_3_ is highly cytotoxic and thus, this is an experimental limitation in this study. Therefore, additional approaches, such as use of molecules known to interfere with trafficking of MHC class I, will be needed in the future. It has been reported that during MHC class I recycling, Rab proteins induce membrane trafficking by cycling between an active GTP-bound state and in-active GDP-bound state (36). Moreover, intracellular trafficking of MHC I by Rab22a contributes to antigen cross-presentation (29). We found that MHC I internalization following PdCl_2_ treatment occurred within a short time frame (within 15 min, **Figure 4A**). Therefore, we hypothesize that PdCl_2_ treatment induces rapid Rab family activation/inactivation, resulting in MHC class I internalization. Further studies will be needed to confirm if this is the underlying mechanism controlling this process.

We show that Pd induces temporal MHC class I internalization and partially recovering. At steady state, spontaneous MHC recycling contributes to the stable expression of self-antigens required for tolerance (31, 37). We found that Pd responsive TCR-expressing TG40 cells are activated by incubation with PdCl_2_-treated DC2.4 cells and this activation is inhibited by co-culture with DC2.4 cells, which suppress membrane movement (**Figures 3D**, **4E**). This suggests that Pd-induced MHC class I internalization is involved in the generation of antigenicity through alternative peptide loading and pathogenic T cell activation, which together are responsible for Pd allergy. This is a novel mechanism of tolerance breakdown in which Pd induces antigenicity of self-proteins through temporal MHC class I internalization. Furthermore, these alternatively selected peptides may be candidate target molecules for therapeutic approaches to treat Pd allergy. However, further investigation will be required to determine the precise mechanism underlying this process in the development of Pd allergy.

## Data Availability Statement

The datasets presented in this study can be found in online repositories. The names of the repository/repositories and accession number(s) can be found below: ProteomeXchange, accession no: PXD028795.

## Ethics Statement

The animal study was reviewed and approved by Institutional Committee for Use and Care of Laboratory Animals of Tohoku University.

## Author Contributions

Conducting experiments: KI, TK and ST. Acquiring data: KI, TK, and ST. Analyze data: KI, TK, and ST. Writing manuscript: KI, TK, and KO. Conceptualization: KI, TT, NH, and KO. Funding acquisition: TK, TT, NH, and KO. Supervision: TT and KO. All authors contributed to the article and approved the submitted version.

## Funding

This work was supported by Grant-in-Aid for Scientific Research from the Japan Society for the Promotion of Science (JSPS) KAKENHI to KO (16H06497, 19H03835) and TK (JP19094976 and JP20240606) and the Japan Agency for Medical Research and Development (AMED) Grant to TK (JP19cm0106352) and partly supported by the Cooperative Research Project Program Joint Usage/Research Center at the Institute of Development, Aging and Cancer, Tohoku University to NH.

## Conflict of Interest

The authors declare that the research was conducted in the absence of any commercial or financial relationships that could be construed as a potential conflict of interest.

The reviewer N.S has declared a shared affiliation with some of the authors KI, NH, KO to the handling editor at the time of review.

## Publisher’s Note

All claims expressed in this article are solely those of the authors and do not necessarily represent those of their affiliated organizations, or those of the publisher, the editors and the reviewers. Any product that may be evaluated in this article, or claim that may be made by its manufacturer, is not guaranteed or endorsed by the publisher.

## Supplementary Material

Supplementary Figure 1 Supplementary Figure 1 Effect of PdCl2 on antigenicity of splenocytes of mice treated with SIINFEKL peptide (A) Mice were injected with SIINFEKL and 12 hours after injection, PdCl2 was injected intraperitoneally. After 12 hours, splenocytes were then analyzed by using antibody clone 25D1.16. Orange histograms represent non-treated cells. Blue histograms represent splenocytes from mice treated with SIINFEKL. Red histograms represent splenocytes from mice treated with SIINFEKL and PdCl2. Bottom graph shows MFI of indicated antibodies. Error bars indicate ± S.D., three mice per group. The data are representative of three independent experiments. (B) Antigenicity of Pd-modulated SIINFEKL-loading splenocytes. Antigen-presenting cells (APC) were prepared from mice treated with SIINFEKL and PdCl2 described as supplementary Fig. 1A. After splenocytes were irradiated, these cells were cocultured with CFSE-labeled OT-I cells. (top) Histograms indicate representative results of CFSE dilution gated on CD8α. (bottom) The graphs show the mean % diluted CFSE. Error bars indicate ± S.D. (n=3). Data are representative of three independent experiments.

Supplementary Figure 2 OVA peptide stripping effect on 25D1.16 binding and OT-I TCR recognitionDC2.4 cells were pulsed with OVA257-264 (SIINFEKL) for 1 hour. After washing, peptide was stripping. (A) 25D1.16 binding to peptide-stripped DC2.4 cells was examined by flow cytometry. The graph shows mean of MFI of 25D1.16. Error bars denote S.D. (triplicated samples, two independent experiments). (B) Peptide-stripped DC2.4 cells were co-cultured with OT-I TCR expressing TG40 cells. Twenty-four hours later, CD69 expression on TG40 cells were evaluated. Graph shows MFI of CD69 expression. Error bars denote S.D. (quadruplicated samples, three independent experiments).

Supplementary Figure 3 TCR repertoire analysis of Pd responsive T cells and naïve lymph node cells The TCR repertoire of naïve LN cells and Pd-responsive T cells was analyzed by next generation sequencing. (A) 3-D graph of TRAV-TRAJ distribution. (B) Pie-chart of CDR3 frequency on TCRα chain. (C) CD69 expression on TCR, which obtained from naïve LN cells, -expressing TG40 cells co-cultured with PdCl2-treated DC2.4 cells as described in Fig 3D. The graph shows the MFI of CD69 expression on TCRβ-expressing cells. Error bars denote ±S.D. (quadruplicated samples, three independent experiments). Statistical differences were determined by unpaired two-sided Student's t-test

Supplementary Figure 4 Inhibition of MHC class I internalization by NaN3Flow cytometric analysis following inhibition of membrane movement by NaN3 treatment. DC2.4 cells were treated with PdCl2 in the absence (left) or presence (right) of 0.25% NaN3 for 30 min and surface MHC class I was stained. The graph shows the relative value of MHC I expression compared to the control without PdCl2 treatment. Error bars denote ±S.D. (triplicated samples) One-way ANOVA with Tukey’s multiple comparison. * and ** denote p<0.05 0.01 compared to the control.

Supplementary Figure 5 Figure 5 The effect of OT-I TCR/TG40 activation on NaN3-treated and exogenously SIINFEKL added-DC2.4.DC2.4 cells were treated with indicated dose of NaN3 for 30 min. After washing, cells were cocultured with OT-I TCR/TG40 cells in the presence of SIINFEKL peptide. Twenty-hours after coculture, CD69 expression was examined by flow cytometry. The graph shows MFI of CD69 expression. Error bars indicate ±S.D. (triplicated wells, three independent experiments). Statistical difference was determined One-way ANOVA with Tukey’s multiple comparison. N.S., not significant.

Supplementary Tables 1–4 Results of MHC ligandome analysis.
