# Supplementary material for: Palladium-Induced Temporal Internalization of MHC Class I Contributes to T Cell-Mediated Antigenicity
Source: Front Immunol. 2021 Dec 23;12:736936. doi: 10.3389/fimmu.2021.736936 (PMC8732370; doi:10.3389/fimmu.2021.736936)

Supplementary Figure 1

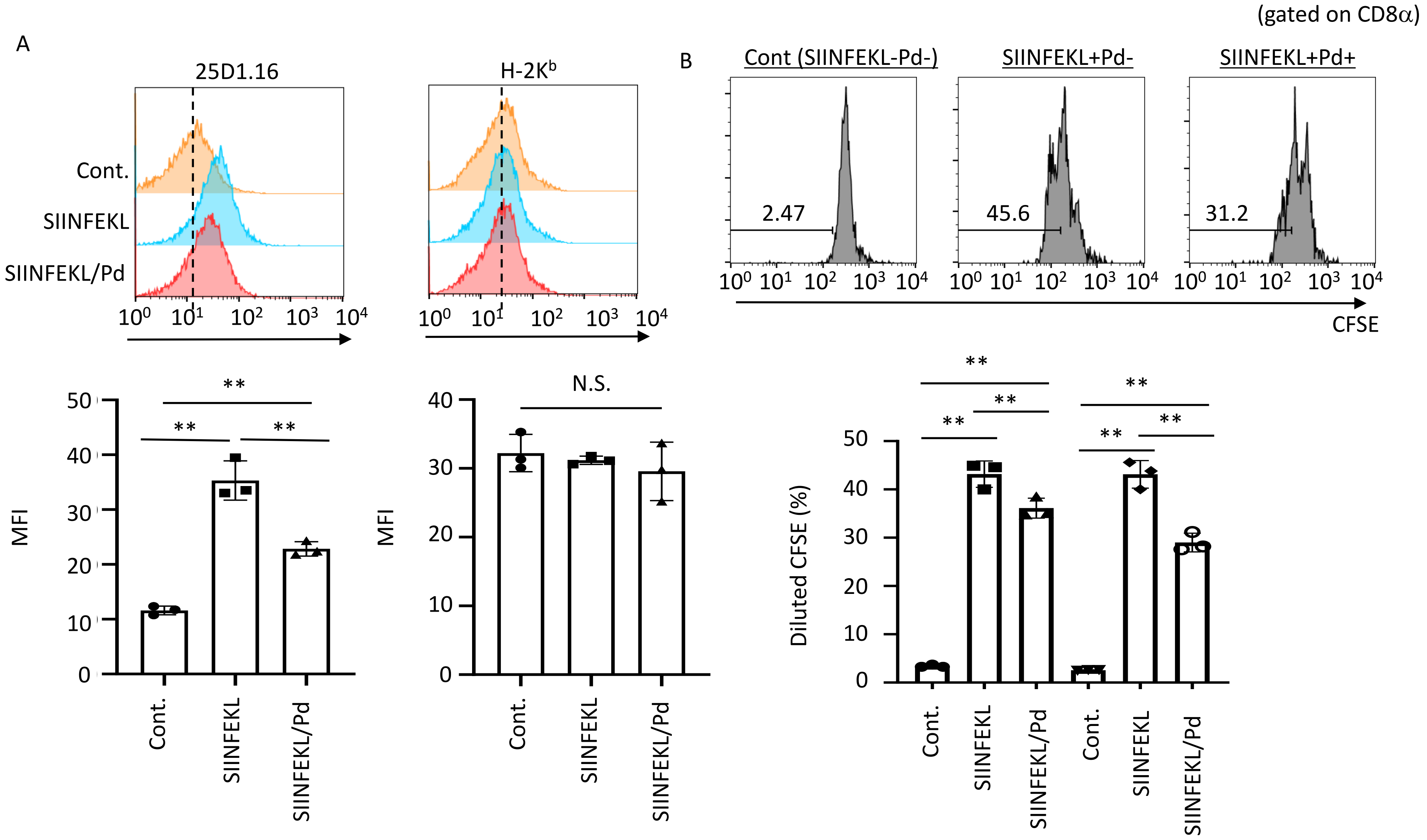

Supplementary Figure 2

A

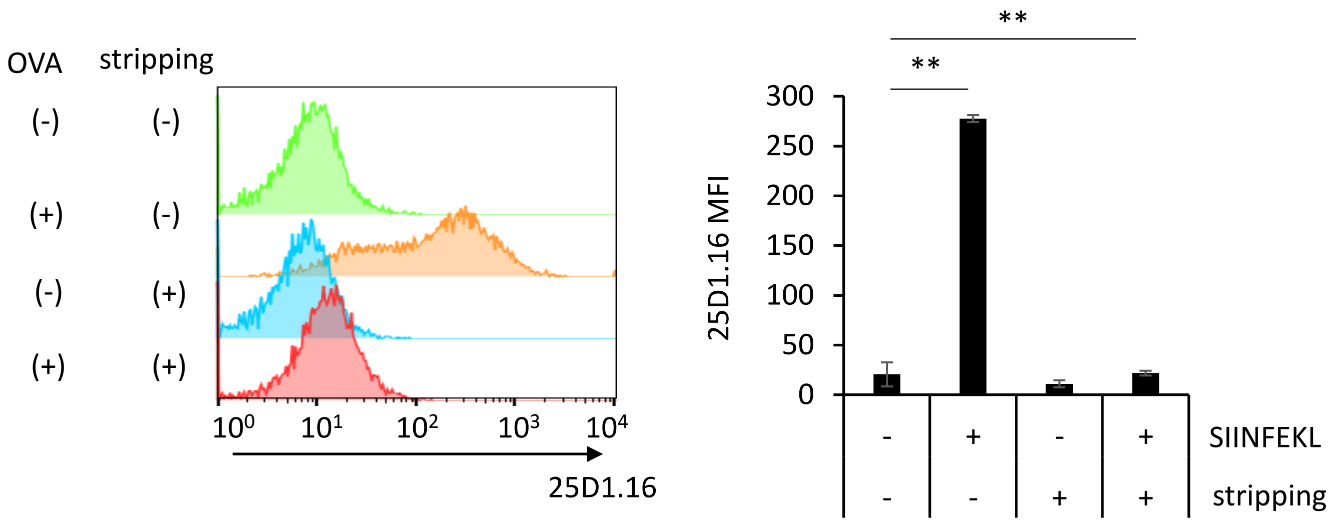

B

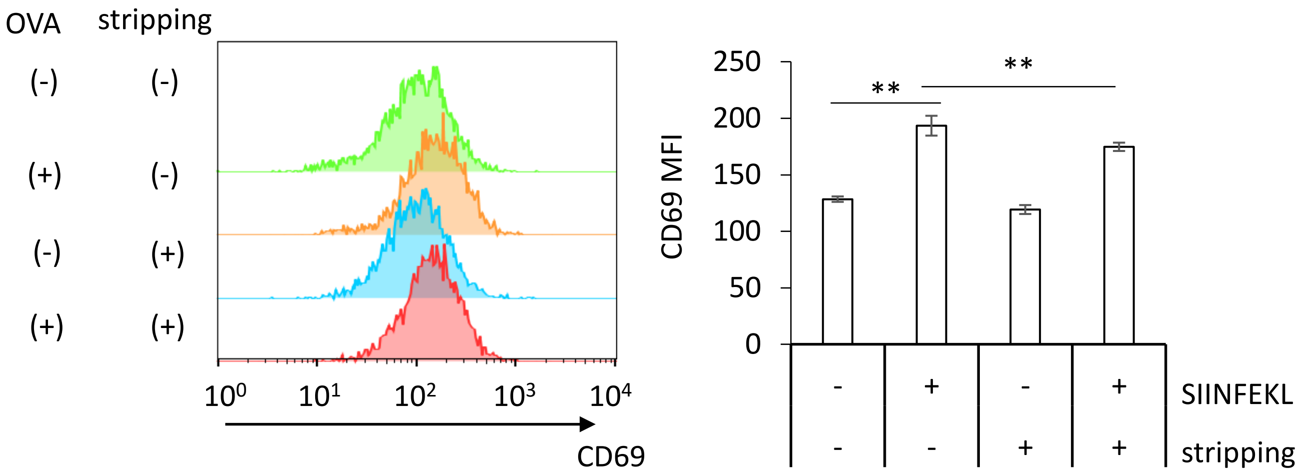

Supplementary Figure 3

A

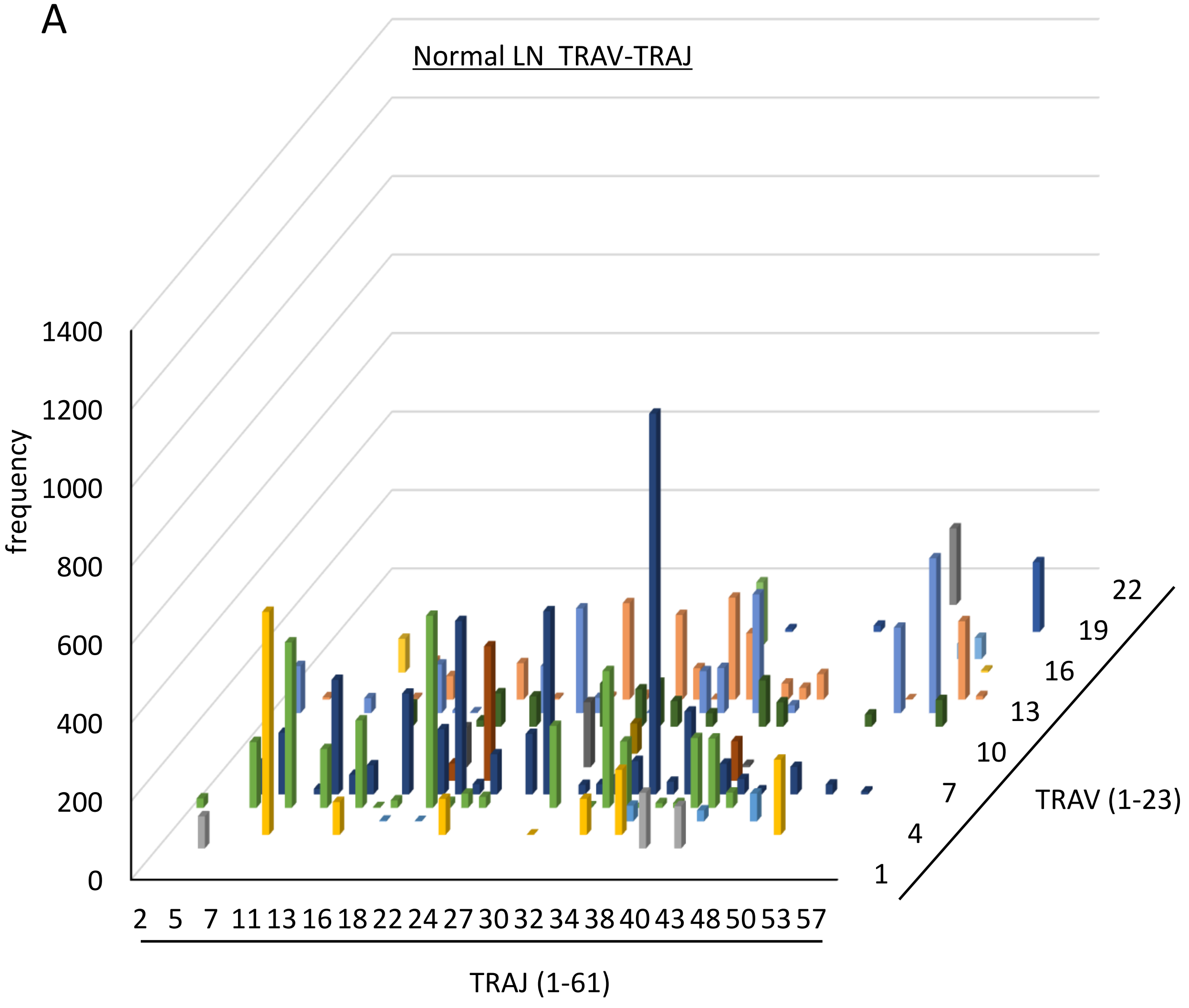

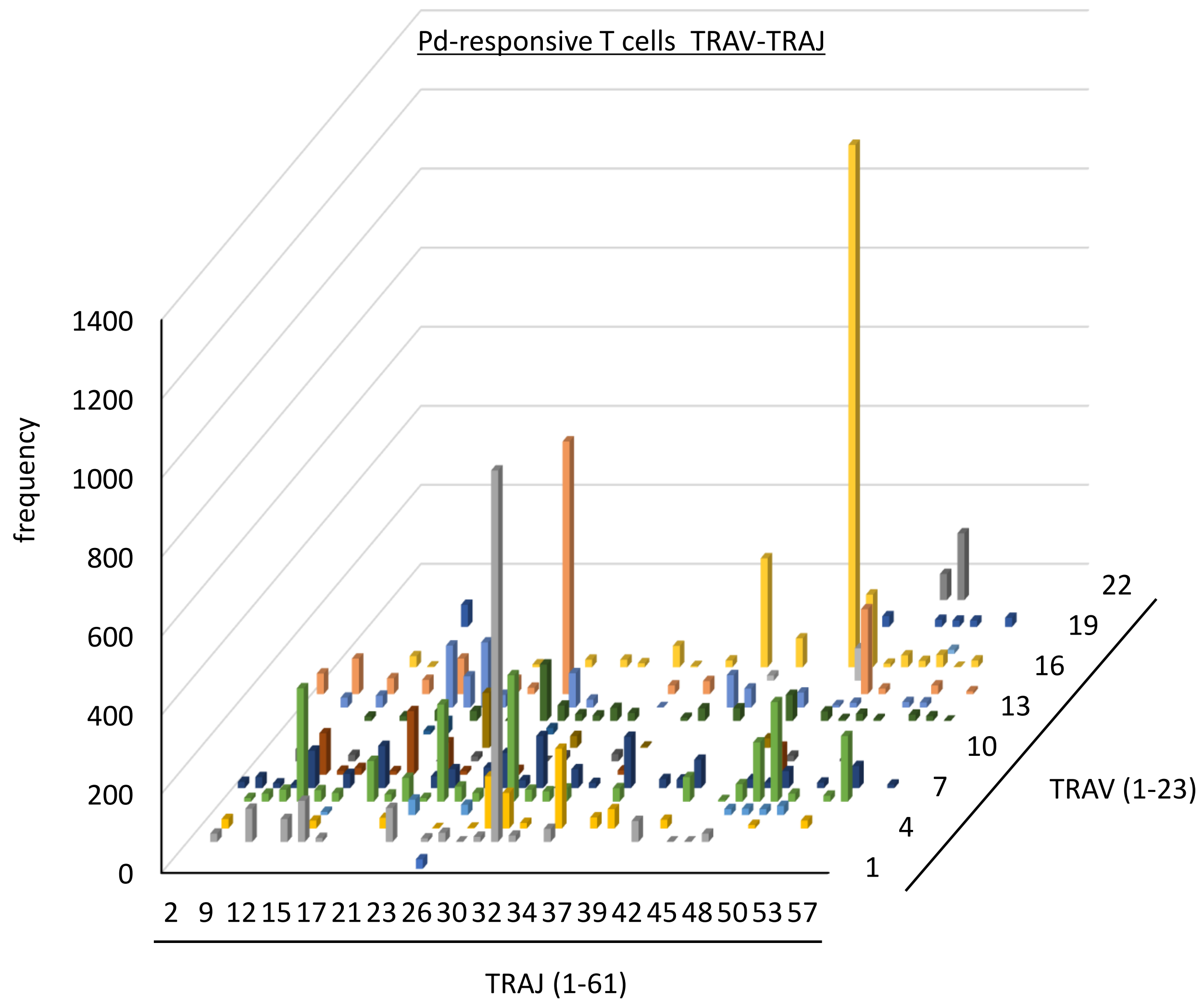

B

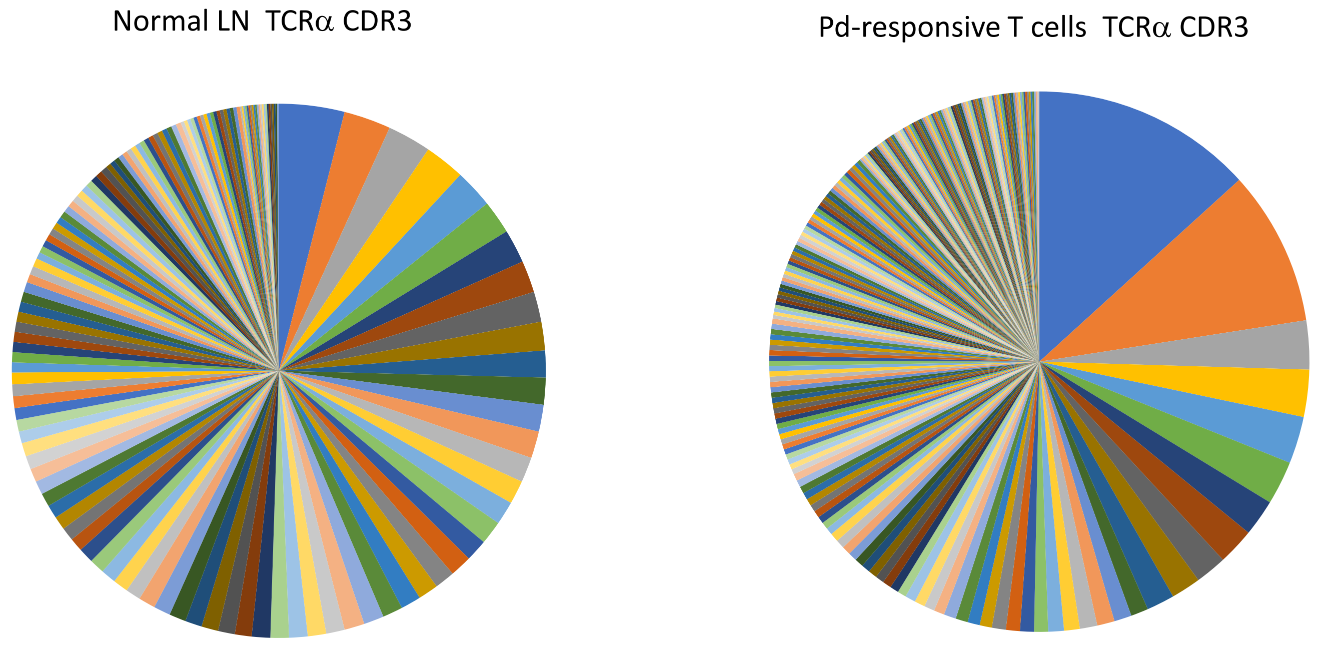

C

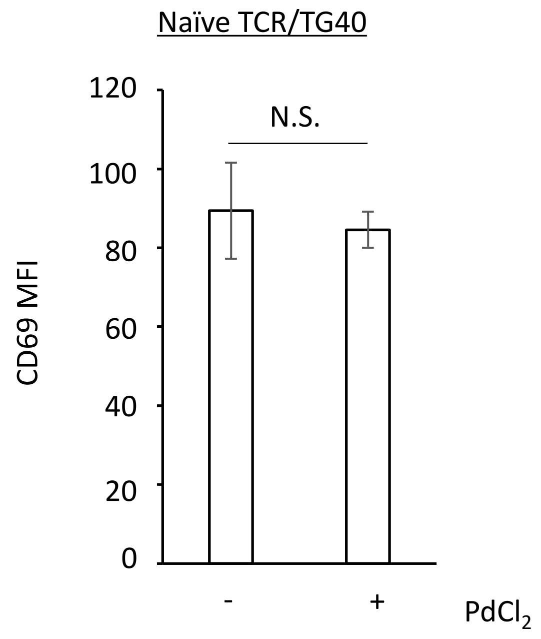

Supplementary Figure 4

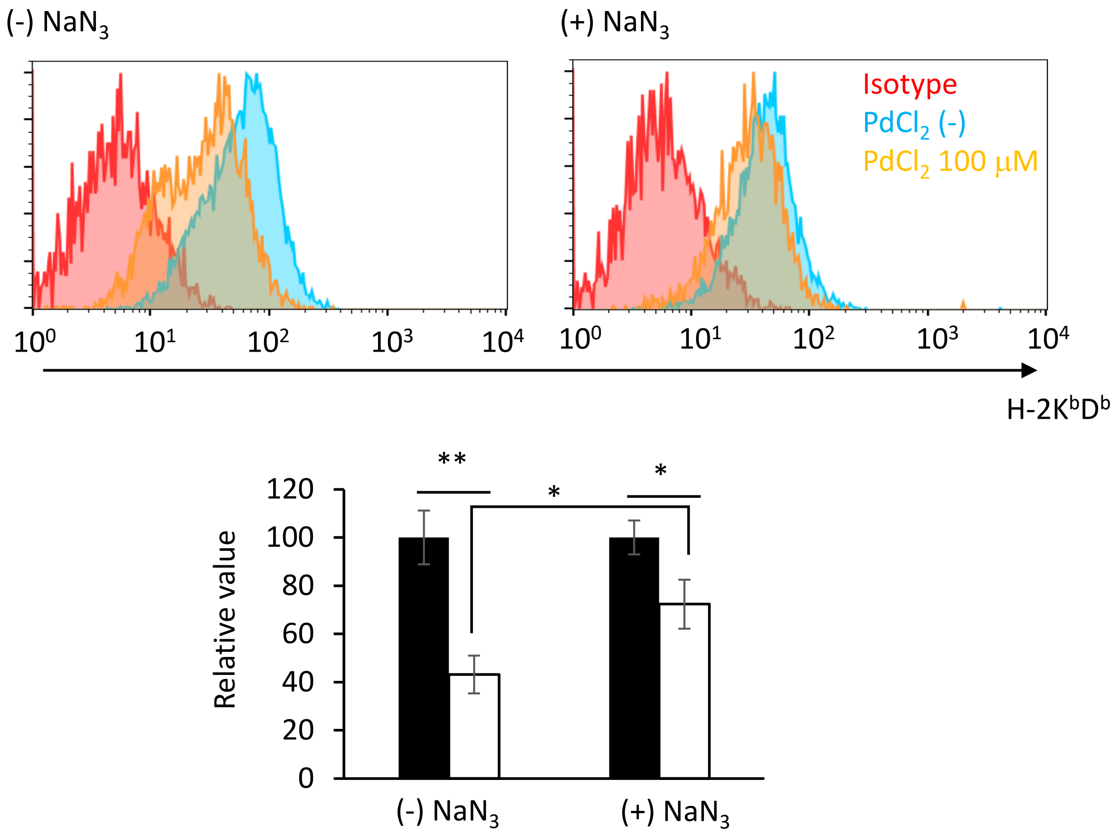

Supplementary Figure 5

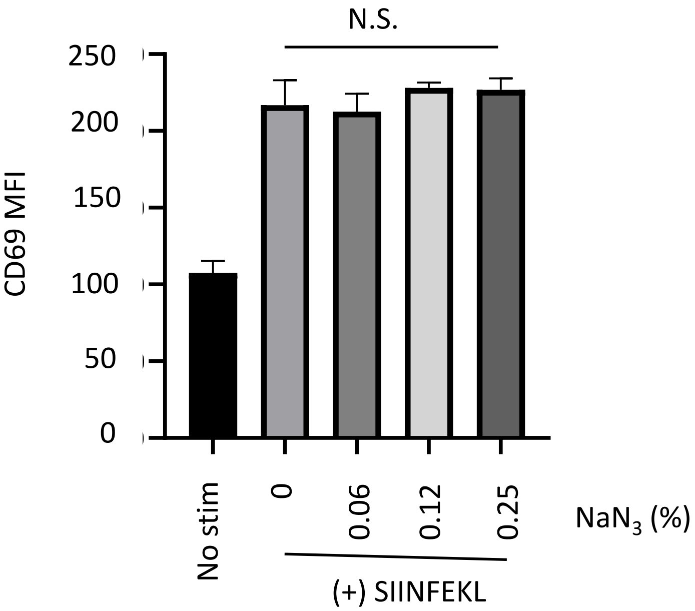

Supplement: Supplementary Figure 1 — Effect of PdCl2 on antigenicity of splenocytes of mice treated with SIINFEKL peptide (A) Mice were injected with SIINFEKL and 12 hours after injection, PdCl2 was injected intraperitoneally. After 12 hours, splenocytes were then analyzed by using antibody clone 25D1.16. Orange histograms represent non-treated cells. Blue histograms represent splenocytes from mice treated with SIINFEKL. Red histograms represent splenocytes from mice treated with SIINFEKL and PdCl2. Bottom graph shows MFI of indicated antibodies. Error bars indicate ± S.D., three mice per group. The data are representative of three independent experiments. (B) Antigenicity of Pd-modulated SIINFEKL-loading splenocytes. Antigen-presenting cells (APC) were prepared from mice treated with SIINFEKL and PdCl2 described as supplementary Fig. 1A. After splenocytes were irradiated, these cells were cocultured with CFSE-labeled OT-I cells. (top) Histograms indicate representative results of CFSE dilution gated on CD8α. (bottom) The graphs show the mean % diluted CFSE. Error bars indicate ± S.D. (n=3). Data are representative of three independent experiments. [file DataSheet_1.pdf]
