# Supplementary material for: Palladium-Induced Temporal Internalization of MHC Class I Contributes to T Cell-Mediated Antigenicity
Source: Front Immunol. 2021 Dec 23;12:736936. doi: 10.3389/fimmu.2021.736936 (PMC8732370; doi:10.3389/fimmu.2021.736936)
Supplement: Supplementary Tables 1–4 — Results of MHC ligandome analysis. [file Table_1.pdf]

Supplementary Table 1 Peptide list on H-2K^b in the absence of $PdCl_2$

Sequence	Sequence Length	Modification	Affinity (nM)	Protein	Protein Accession
ISYQFSNL	8		1.7	Tripartite motif-containing protein 12A	Q99PQ1
VSYLFSHV	8		1.8	ribose-phosphate pyrophosphokinase 2	Q9CS42; Q9D7G0
LSYSFAHL	8		1.8	NACHT, LRR and PYD domains-containing protein 1b allele 2	A1Z198; Q2LKV2; Q0GKD5; Q2LKU9-2; A1Z198-3; Q2LKV5; Q2LKV2-2; Q0GKD5-2; A1Z198-2; Q2LKW6; Q2LKU9
MSFQFAHL	8		1.8	Vacuolar protein sorting-associated protein 13B OS=Mus musculus OX=10090	Q80TY5; Q80TY5
SSFAFAGL	8		1.8	Phospholipid phosphatase 5	Q3UMZ3; Q3UMZ3-2
VSFTYRYL	8		1.9	Vacuolar protein sorting-associated protein 16 homolog	Q920Q4
INYSYINL	8		1.9	Ubiquitin carboxyl-terminal hydrolase 24	B1AY13
VTYIFNHL	8		1.9	Transmembrane 6 superfamily member 1	P58749
FAYRFSNL	8		2	Periodic tryptophan protein 2 homolog	Q8BU03
SSFVFLNL	8		2	surfeit locus protein 4	Q64310
VVYRFFSRL	9		2	Protein RER1	Q9CQU3
IAYAFFHL	8		2	Suppressor of tumorigenicity 7 protein-like	Q99M96-5; Q99M96-2; Q99M96-9; Q8K4P7-3; Q99M96-4; Q99M96-8; Q99M96-6; Q8K4P7; Q99M96-3; Q99M96-7; Q99M96
SSYSFRHL	8		2.1	Lysosomal thioesterase PPT2	O35448
ISYLYNKL	8		2.1	Protein Tob1	Q9JM55; Q61471
ISLRFTHL	8		2.1	Phosphatidylinositol 3,4,5-trisphosphate 5-phosphatase 2	Q6P549
VSYQFPKL	8		2.1	Suppression of tumorigenicity 5 protein	Q924W7-2; Q924W7; Q924W7-3
ASYLFRGL	8		2.1	Transmembrane channel-like protein 6	Q7TN60; Q7TN60-2; Q7TN60-3
ISFEFRSL	8		2.2	Coenzyme Q-binding protein COQ10 homolog B, mitochondrial	Q3THF9-1; Q3THF9-2
VVFYFTHL	8		2.2	Metalloendopeptidase OMA1, mitochondrial	Q9D8H7-2; Q9D8H7
AIYAFSHL	8		2.2	ATP-binding cassette sub-family B member 9	Q9JJ59-1; Q9JJ59-2
SSYKFNHL	8		2.3	protein farnesyltransferase subunit beta	Q8K2I1
VQYKFSHL	8		2.3	Histone acetyltransferase KAT2B	Q9JHD1; Q9JHD2
VSLWFRHL	8		2.3	Fanconi anemia group C protein homolog	P50652
LVYKFMNL	8	1xOxidation [M6]	2.3	Proteasome-associated protein ECM29 homolog	Q6PDI5-1
SNFTFSHL	8		2.3	Protein dispatched homolog 1	Q3TDN0-1
VVYSYHYL	8		2.3	General transcription and DNA repair factor IIH helicase subunit XPD	O08811
TSFRYSSL	8		2.3	Fibronectin type-III domain-containing protein 3A	Q8BX90
VSYQHAFL	8		2.3	Nucleolar complex protein 2 homolog	Q9WV70
VTYLFKQL	8		2.3	Exportin-7	Q9EPK7-2; Q9EPK7
SNYLFTKL	8		2.4	endothelial PAS domain-containing protein 1	P97481
TAYEFAKL	8		2.4	Estradiol 17-beta-dehydrogenase 11	Q9EQ06-2; Q9EQ06
KSYLFQLL	8		2.4	cyclin-dependent kinase 2	P97377-2; P97377-1
KSFLFSAL	8		2.4	Elongation of very long chain fatty acids protein 6	Q920L5
YSYSFFHL	8		2.4	Serine incorporator 3	Q9QZI9
RAYLFAHV	8		2.5	Pre-rRNA-processing protein TSR1 homolog	Q5SWD9-1; Q5SWD9-2; Q5SWD9-3
VSIQFYHL	8		2.5	Vacuolar protein sorting-associated protein 13a	Q5H8C4; Q5H8C4-2
SAYRFSGV	8		2.5	protein elys	Q8CJF7
VSLKYAHM	8		2.5	Adenylosuccinate synthetase isozyme 2	P46664
SGFVFTRL	8		2.5	Helicase-like transcription factor	Q6PCN7
VSYEKFSNL	9		2.5	TBC1 domain family member 9B	Q5SVR0; Q5SVR0-2
VAYTYDNL	8		2.5	Ubiquitin carboxyl-terminal hydrolase 34	Q6ZQ93; Q6ZQ93-3; Q6ZQ93-4; Q6ZQ93-2
VVYIYHSL	8		2.6	ribosomal oxygenase 2	Q8CD15
QSYEFFHL	8		2.6	Nucleotide-binding oligomerization domain-containing protein 1	Q8BHB0
LTYTFSGL	8		2.6	DNA oxidative demethylase ALKBH2	Q6P6J4
ASYEFTTL	8		2.6	Developmentally-regulated GTP-binding protein 2	Q9QXB9
SSYTFEQL	8		2.6	Myb/SANT-like DNA-binding domain-containing protein 2	Q6NZR2
IIPMFSNL	8		2.6	Serine/threonine-protein phosphatase 2A 65 kDa regulatory subunit A alpha isoform	Q76MZ3
SSYIYGYV	8		2.6	Proteasome subunit beta type-9	P28076; Q60692

SSLDFYANV	9		2.6	Sialoadhesin	Q62230-3; Q62230
SSYMHFTNV	9		2.6	Tensin-3	Q5SSZ5
VAYKFPEL	8		2.6	E3 ubiquitin-protein ligase UBR2	Q6WKZ8; Q6WKZ8-3; Q6WKZ8-2
SGYKFFSL	8		2.6	WD repeat domain phosphoinositide-interacting protein 2	Q80W47
TNYRFKNL	8		2.7	G1/S-specific cyclin-E2	Q9Z238
ISFKFDHL	8		2.7	F-actin-capping protein subunit alpha-1	P47753
HVYYFAHL	8		2.7	Interferon-induced very large GTPase 1	Q80SU7
RSYRFPKL	8		2.7	Insulin-like growth factor 1 receptor	Q60751
SSYNYIRV	8		2.7	Unconventional myosin-Id	Q5SYD0-2; Q5SYD0
LIYTYSGL	8		2.7	Myosin-11	Q8BTV2-2; Q6URW6-2; Q8BTV2; O08638-2; O08638-1; Q8VDD5; Q6URW6-1; Q6URW6-3; Q61879
SSIVFAEL	8		2.7	Eukaryotic translation initiation factor 2-alpha kinase 1	Q9Z2R9
IAYLYDRL	8		2.7	DNA replication complex GINS protein PSF1	Q9CZ15
KVYLYTHL	8		2.7	Uncharacterized protein KIAA0513	Q8R0A7
STFTFADL	8		2.7	E3 SUMO-protein ligase RanBP2	Q9ERU9
VNLLFSGL	8		2.7	High affinity copper uptake protein 1	Q8K211
SAYLFTYV	8		2.7	Cationic amino acid transporter 4	Q8BLQ7
TTYKYFAL	8		2.7	Terminal uridylyltransferase 7	Q5BLK4
SALTFAGL	8		2.8	Succinate dehydrogenase [ubiquinone] cytochrome b small subunit, mitochondrial	Q9CXV1
RNFIFSRL	8		2.9	Lymphocyte-specific helicase	Q60848-2; Q60848-1
RTYSFLNL	8		2.9	Transmembrane protein 39B	Q810L4
VGYRYETL	8		2.9	AMP deaminase 2	Q9DBT5
SSFVFSTV	8		2.9	3-hydroxy-3-methylglutaryl-coenzyme A reductase	Q01237
ASFIFRQL	8		2.9	PAS domain-containing serine/threonine-protein kinase	Q8CEE6
SAYLYTYV	8		2.9	Cationic amino acid transporter 2	P18581-2; P18581
KSYNFHTGL	9		2.9	Protein SMG8	Q8VE18
VNFTYQFL	8		2.9	WASH complex subunit 4	Q3UMB9
VNYDYSTL	8		2.9	Zinc finger BED domain-containing protein 6	D2EAC2; D2EAC2-2
TNYKFFML	8	1xOxidation [M7]	2.9	Palmitoyltransferase ZDHHC20	Q5Y5T1-2; Q5Y5T1
ISVRFHNL	8		2.9	Zinc finger SWIM domain-containing protein 3	Q8CFL8
HGYTFANL	8		3	SUMO-activating enzyme subunit 1	Q9R1T2; Q9R1T2-2
RNYRYFYL	8		3	Palmitoyltransferase ZDHHC9	P59268
SAYLFVKL	8		3	PDZ domain-containing protein 8 OS=Mus musculus OX=10090	B9EJ80
VIVKFAQL	8		3	Protein RRP5 homolog	Q6NS46
VVFFFTRL	8		3	Protein BTG3	P50615
ATFPFAML	8		3	Sodium- and chloride-dependent taurine transporter	O35316
IRYIFAYL	8		3.1	Hematopoietic prostaglandin D synthase	Q9JHF7
ATYIFNGL	8		3.1	Coatomer subunit gamma-2	Q9QXK3; Q9QXK3-4
SSYRFVQNV	9		3.1	Forkhead box protein K1	P42128
VAHLYSNL	8		3.1	ATP-binding cassette sub-family D member 2	Q61285; P48410
LNYTYSQL	8		3.1	Girdin	Q5SNZ0-2; Q5SNZ0-3; Q5SNZ0
QSFFFTHL	8		3.1	Cytoplasmic FMR1-interacting protein 2	Q5SQX6
VALEFTHL	8		3.1	p53-induced death domain-containing protein 1	Q9ERV7
AGYMYTQL	8		3.1	E3 ubiquitin-protein ligase UBR4	A2AN08-3; A2AN08-5; A2AN08
VALAFRHL	8		3.2	A-kinase anchor protein 13	E9Q394; E9Q394-2
VTFIYQKL	8		3.2	Coiled-coil domain-containing protein 159	Q8C963-2; Q8C963-1
STYLFRLM	8		3.2	ubiquitin carboxyl-terminal hydrolase 30	Q3UN04
SSYLHSLL	8		3.2	N-alpha-acetyltransferase 25, NatB auxiliary subunit	Q8BWZ3-2; Q8BWZ3
IVPLFTNL	8		3.2	Serine/threonine-protein phosphatase 2A 65 kDa regulatory subunit A beta isoform	Q7TNP2
IGWRYSLL	8		3.2	Monocarboxylate transporter 7	B1AT66; B1AT66-2
SSYVHSNL	8		3.2	Sorting nexin-19	Q6P4T1
ISRTFPNL	8		3.2	TBC1 domain family member 14	Q8CGA2
TSFMFQRV	8		3.3	annexin A4	P97429
VTYLFHIL	8		3.3	Stimulated by retinoic acid gene 6 protein-like	Q9DBN1; Q9DBN1-2

RSFLHARL	8	3.3	26S proteasome non-ATPase regulatory subunit 3	P14685
KIYYFAAV	8	3.3	Tyrosine-protein phosphatase non-receptor type 23	Q6PB44-1; Q6PB44-2
SAFSFRTL	8	3.3	F-box only protein 38	Q8BMI0
AAYEFTTL	8	3.3	Developmentally-regulated GTP-binding protein 1	P32233
SQYRFCQL	8	3.3	Exocyst complex component 6	Q8R313
AGYCFGNL	8	3.3	Phosphoribosylformylglycinamidine synthase	Q5SUR0
VNYRHLAL	8	3.3	DNA-directed RNA polymerase II subunit RPB1	P08775
VTFSFKQL	8	3.3	Phosphatidylinositol 3,4,5-trisphosphate-dependent Rac exchanger 1 protein	Q69ZK0; Q69ZK0-2
SALRFLNL	8	3.3	Serine/threonine-protein kinase 11-interacting protein	Q3TAA7
TAFRFSEL	8	3.3	TBC1 domain family member 8B	A3KGB4
SSYTFPKM	8	3.4	Enoyl-CoA delta isomerase 2, mitochondrial	Q9WUR2; Q9WUR2-2
SNFHFAVL	8	3.4	Vacuolar protein sorting-associated protein 13B OS=Mus musculus OX=10090	Q80TY5; Q80TY5
ASYLLAAL	8	3.4	60S acidic ribosomal protein P2	P99027
QNYRFYRGM	9	3.4	GC-rich sequence DNA-binding factor 2	Q8BKT3-2; Q8BKT3
VAYRYEVL	8	3.4	Dual specificity tyrosine-phosphorylation-regulated kinase 2	Q5U4C9
VIYPFMQGL	9	3.5	DENN domain-containing protein 2D	Q91VV4; Q91VV4-2
SSYTFPQI	8	3.5	Chromodomain Y-like protein 2	Q9D5D8
ISYRLPGL	8	3.5	E3 ubiquitin-protein ligase RNF213	E9Q555
RAYRYLQL	8	3.6	Cytochrome c oxidase assembly protein COX14	Q8BH51
VALLFRQL	8	3.6	Myotubularin-related protein 5	Q6ZPE2-2; Q6ZPE2
SGYDFSRL	8	3.6	Sterol regulatory element-binding protein cleavage-activating protein	Q6GQT6
SALVFTRL	8	3.6	Glycosylated lysosomal membrane protein	Q9JHJ3
VTIEYYSQL	9	3.6	4-trimethylaminobutyraldehyde dehydrogenase	Q9JLJ2
VTWKYTAL	8	3.6	Pyruvate dehydrogenase phosphatase regulatory subunit, mitochondrial	Q7TSQ8
VNFVHTNL	8	3.6	60S ribosomal protein L4	Q9D8E6
FTFQFNNL	8	3.6	Ubiquitin carboxyl-terminal hydrolase 15	Q8R5H1; Q8R5H1-2; Q8R5H1-5
ISILYHQL	8	3.7	Protein AAR2 homolog	Q9D2V5
RNYEYCRL	8	3.7	SNF-related matrix-associated actin-dependent regulator of chromatin subfamily A meml	Q91ZW3
ITFIFKSL	8	3.7	Serine/threonine-protein kinase mTOR	Q9JLN9
VNYRHLALL	9	3.7	DNA-directed RNA polymerase II subunit RPB1	P08775
VSRTFHNL	8	3.7	Endoplasmic reticulum metallopeptidase 1	Q3UVK0
TIYRFLKL	8	3.7	Protein fem-1 homolog C	Q8CEF1
AVYTYLRL	8	3.7	Integrator complex subunit 3	Q7TPD0-2; Q7TPD0
ANFTFPRL	8	3.7	Sorting nexin-27	Q3UHD6; Q3UHD6-2
KGFYFAKL	8	3.8	E3 SUMO-protein ligase RanBP2	Q9ERU9
VAYGFRNI	8	3.8	Cytosolic iron-sulfur assembly component 3	Q7TMW6-2; Q7TMW6
SQYRFEHL	8	3.8	GTP-binding protein 2	Q3UJK4
TQYSFYQQL	9	3.8	Inositol 1,4,5-trisphosphate receptor type 2	Q9Z329; Q9Z329-3; Q9Z329-2
FTYRYLAL	8	3.8	Vacuolar protein sorting-associated protein 16 homolog	Q920Q4
VNLQYRRL	8	3.9	lysosomal protective protein	P16675
VTYVYQLI	8	3.9	Vacuolar protein sorting-associated protein 29	Q9QZ88; Q9QZ88-2
QNYTYSSL	8	3.9	Calcium uniporter regulatory subunit MCUb, mitochondrial	Q810S1
VNYDFGHM	8	3.9	E3 ubiquitin-protein ligase RNF216	P58283-2; P58283
SNYHFYSSI	9	3.9	Nuclear factor erythroid 2-related factor 2	Q60795
IGPTYYQRL	9	3.9	DNA-directed RNA polymerase II subunit RPB2	Q8CFI7
ITYAWTRL	8	3.9	Probable dolichyl pyrophosphate Glc1Man9GlcNAc2 alpha-1,3-glucosyltransferase	Q6P8H8
ATLRYASL	8	3.9	Amyloid-beta A4 precursor protein-binding family B member 2	Q9DBR4-2; Q9DBR4; Q9DBR4-3
NSYMFTSL	8	3.9	Transmembrane 7 superfamily member 3	Q9CRG1
RNYRYFFL	8	3.9	Palmitoyltransferase ZDHHC5	Q8VDZ4; Q8VDZ4-2; Q5Y5T5
KVYNYNHL	8	4	60S ribosomal protein L27	P61358
RSFDFIHL	8	4	TRMT1-like protein	A2RSY6; A2RSY6-2
RQYMFSSL	8	4	X-ray repair cross-complementing protein 5	P27641
SSYDYEAL	8	4	Apoptotic protease-activating factor 1	O88879-2; O88879
LQWRFANL	8	4	Tether containing UBX domain for GLUT4	Q8VBT9

SSMRYVLL	8		4	Alpha-L-iduronidase	P48441
AIFNFQSL	8		4	Protein kish-A	Q9CR64; Q9CR64-2
SSFRFEVL	8		4	Short transient receptor potential channel 4	Q9QUQ5-2; Q9QUQ5
STYVLSNL	8		4.1	F-box only protein 22	Q78JE5
SVYKFFDL	8		4.1	Retinoblastoma-associated protein	P13405
STLLFSSL	8		4.1	Thyroid adenoma-associated protein homolog	A8C756
RTYIFTFL	8		4.2	Ras-GEF domain-containing family member 1B	Q8JZL7-2; Q8JZL7
KAFTYINL	8		4.2	Transferrin receptor protein 1	Q62351
KGFTFSAL	8		4.2	transmembrane protein 135	Q9CYV5
ASYEFVQRL	9		4.2	Cytoplasmic dynein 1 heavy chain 1	Q9JHU4
IALRYVAL	8		4.2	Coatomer subunit beta	Q9JIF7
TSYIFVSV	8		4.2	Phosphatidylinositol 4,5-bisphosphate 3-kinase catalytic subunit alpha isoform	P42337
QAIHFANL	8		4.2	Myotubularin-related protein 13	E9PXF8-2; E9PXF8
TILSFTNL	8		4.2	Sodium-coupled neutral amino acid transporter 2	Q8CFE6
VKYLFTGL	8		4.2	Ubiquitin carboxyl-terminal hydrolase 14	Q9JMA1
SAVVYAQL	8		4.2	5'-3' exoribonuclease 1	P97789-2; P97789-3; P97789
TILEFAQL	8		4.3	UDP-glucuronic acid decarboxylase 1	Q91XL3
ISFEFRSLL	9		4.3	Coenzyme Q-binding protein COQ10 homolog B, mitochondrial	Q3THF9; Q3THF9-2
VSISFKSL	8		4.4	Spatacsin	Q3UHA3-2; Q3UHA3
SGYQYKRL	8		4.4	Histone-lysine N-methyltransferase SETDB1	O88974-4; O88974-1
ASYVYLSM	8	1xOxidation [M8]	4.4	Ferritin, mitochondrial	P09528; Q9D5H4
VVYIYRQI	8		4.4	Isoform 4 of Sorbin and SH3 domain-containing protein 1	Q62417-3; Q62417-5; Q62417-2; Q62417-4; Q62417; Q62417-7; Q62417-6
KSYSFIARM	9		4.4	Calcium homeostasis endoplasmic reticulum protein	Q8CGZ0
TLYEYSPL	8		4.4	Cell growth regulator with RING finger domain protein 1	Q8BMJ7
SSLHFSFL	8		4.4	DnaJ homolog subfamily C member 16	Q80TN4
ISLDYQHL	8		4.4	Protein KIAA0100	Q5SYL3
VTWGFPNL	8		4.4	60S ribosomal protein L7-like 1	Q9D8M4
SAYEYLEL	8		4.4	Sodium-dependent multivitamin transporter	Q5U4D8
ANYDFYQL	8		4.4	Protein SMG8	Q8VE18
SGYSFTHI	8		4.5	Actin-related protein 6	Q9D864
IAMEFNHL	8		4.5	Prostaglandin G/H synthase 1	P22437
SQYLFPKL	8		4.5	Peroxisome proliferator-activated receptor delta	P35396
TIYKFQGM	8		4.5	general transcription factor 3C polypeptide 5	Q8R2T8-2; Q8R2T8
VGMKYRNL	8		4.5	Neurolysin, mitochondrial	Q91YP2
TAYAFHFL	8		4.5	Peroxisomal acyl-coenzyme A oxidase 1	Q9R0H0; Q9R0H0-2
AAYPFVNM	8		4.5	Peroxisome assembly protein 12	Q8VC48
SSYSFRHLL	9		4.5	Lysosomal thioesterase PPT2	O35448
VGFIYGYV	8		4.5	Signal peptidase complex subunit 1	Q9D958
RSYRFMVM	8		4.6	serine/threonine-protein kinase VRK2	Q8BN21
VAFAFKKL	8		4.6	Interleukin-1 receptor-associated kinase-like 2	Q8CFA1-1; Q8CFA1-4; Q8CFA1-3; Q8CFA1-2
KVMPFANL	8		4.6	DNA-directed RNA polymerase III subunit RPC5	Q9CZT4-2; Q9CZT4-1
VILEYFTRL	9		4.6	Ribonuclease P protein subunit p29	Q9CR08
TSYLMGNL	8		4.6	Rho guanine nucleotide exchange factor 7	Q9ES28; Q9ES28-7; Q9ES28-5; Q9ES28-3; Q9ES28-4; Q9ES28-6; Q9ES28-8; Q9ES28-2
SVYTHSYL	8		4.6	Ectonucleoside triphosphate diphosphohydrolase 8	Q3TZX8-3; Q3TZX8-2; Q8K0L2-2; Q8K0L2; Q3TZX8
LSLRYHNL	8		4.6	DNA excision repair protein ERCC-1	P07903
SGYKFGVL	8		4.6	General transcription factor IIE subunit 2	Q9D902
VGPRYTNL	8		4.6	Mitogen-activated protein kinase 1	P63085
SSILYPLL	8		4.6	E3 ubiquitin-protein ligase SHPRH	Q7TPQ3-5; Q7TPQ3; Q7TPQ3-3; Q7TPQ3-2
YAYSFKYL	8		4.6	Transcription elongation factor SPT6	Q62383
VSPLFQKL	8		4.7	Methionine--tRNA ligase, cytoplasmic	Q68FL6
SIYRFHAQF	9		4.7	FYVE, RhoGEF and PH domain-containing protein 2	Q8BY35
STLQYLNL	8		4.7	Histone acetyltransferase KAT5	Q8CHK4-3; Q8CHK4-4; Q8CHK4-2; Q8CHK4

RAFGFSHL	8		4.7	Ubiquitin thioesterase OTUB1	Q7TQI3
LQYIFAHV	8		4.7	Pre-mRNA-processing-splicing factor 8	Q99PV0
VIISFNSL	8		4.7	Putative sodium-coupled neutral amino acid transporter 10	Q5I012-3; Q5I012; Q5I012-2; Q5I012-4
IHYFFSKL	8		4.7	Transmembrane 9 superfamily member 2	P58021
SGYKYVGM	8	1xOxidation [M8]	4.8	protein YIF1A	Q91XB7
LQYEFTHL	8		4.8	formin-like protein 1	Q9JL26-2; Q9JL26
SGYIYHKL	8		4.8	Regulator of nonsense transcripts 1	Q9EPU0-1; Q9EPU0-2
VAYEYLCHL	9		4.8	Ras GTPase-activating-like protein IQGAP1	Q9JKF1
ISYDYLTSL	9		4.8	Uncharacterized aarF domain-containing protein kinase 1	Q9D0L4
STLLFAHI	8		4.8	Anaphase-promoting complex subunit 1	P53995
SSAIYMNL	8		4.8	Transcriptional repressor p66-beta	Q8VHR5-2; Q8VHR5
SIWLFQANL	9		4.8	Bax inhibitor 1	Q9D2C7
VYRFFSRL	8		4.9	Protein RER1	Q9CQU3
AAFVFRKL	8		4.9	snRNA-activating protein complex subunit 1	Q8K0S9
SSLLFRRV	8		4.9	Mitochondrial carrier homolog 1	Q791T5-2; Q791T5
RGYLYQTL	8		4.9	Serine/threonine-protein kinase Kist	P97343
VNIPFVRL	8		5	NFX1-type zinc finger-containing protein 1	Q8R151
VNYYFERNM	9		5	PHD finger protein 14	Q9D4H9; Q9D4H9-3; Q9D4H9-2
TSLKYMLL	8		5.1	26S proteasome non-ATPase regulatory subunit 11	Q8BG32
SSVRFSYM	8		5.1	Nucleolar protein 6	Q8R5K4-2; Q8R5K4
QNPVYAPL	8		5.2	heme oxygenase 1	P14901
QIIAFFAHL	9		5.2	E1A-binding protein p400	Q8CHI8-4; Q8CHI8-3; Q8CHI8-2; Q8CHI8-5; Q8CHI8
STYDFMSTL	9		5.2	Beta-hexosaminidase subunit alpha	P29416
STSRFARL	8		5.2	3-hydroxy-3-methylglutaryl-coenzyme A reductase	Q01237
ISLSYSRI	8		5.2	26S proteasome non-ATPase regulatory subunit 3	P14685
TSVRFTQL	8		5.3	nitric oxide synthase-interacting protein	Q9D6T0-1
SSFRMRHL	8		5.3	Microtubule-actin cross-linking factor 1	Q9QXZ0-3; Q9QXZ0-2; Q9QXZ0-4; Q9QXZ0
SSYDFTSI	8		5.3	Acyl-CoA synthetase family member 2, mitochondrial	Q8VCW8
RAFAFTNV	8		5.3	Large neutral amino acids transporter small subunit 4	Q8CGA3
VAYQVGNL	8		5.4	ABI gene family member 3	Q8BYZ1-2; Q8BYZ1
SSYNYRVV	8		5.4	Inositol 1,4,5-trisphosphate receptor type 1	P11881-7; P11881-2; P11881-8; P11881-5; P11881-3; P11881-4; P11881-6; P11881
IAFGFHQL	8		5.4	Peroxisomal membrane protein PEX14	Q9R0A0
AHYTYNNL	8		5.4	Sodium/hydrogen exchanger 7	Q8BLV3-2; Q8BLV3; Q8BZ00
KGYRFIFL	8		5.4	Vacuolar protein sorting-associated protein 33B	P59016
IVLRFRNI	8		5.4	Vacuolar protein sorting-associated protein 52 homolog	Q8C754; Q8C754-2
STVEFTNL	8		5.4	Centromere/kinetochore protein zw10 homolog	O54692
QSIEFSRL	8		5.5	Eukaryotic translation initiation factor 3 subunit A	P23116
VQFLYREL	8		5.5	Dynein assembly factor 5, axonemal	B9EJR8
SNYLYREV	8		5.5	Xylosyltransferase 2	Q9EPL0
VVYAVRNL	8		5.5	Ataxin-10	P28658
SAFIFRVL	8		5.5	Chloride channel protein 2	Q9R0A1
GNYNFFNL	8		5.5	Lipase maturation factor 2	Q8C3X8
IAWGYPNL	8		5.5	60S ribosomal protein L7	P14148
TNYRFPSSL	9		5.5	UDP-glucuronic acid/UDP-N-acetylgalactosamine transporter	A2AKQ0; A2AKQ0-2
SIHSFQNL	8		5.6	Transcription factor p65	Q04207-1; Q04207-2
NAYKFPNL	8		5.6	Aldehyde oxidase 1	O54754
SNYSYPQV	8		5.6	RNA-binding protein EWS	Q61545
IAYGYNNI	8		5.6	Phenylalanine--tRNA ligase beta subunit	Q9WUA2
SAFEFNEL	8		5.6	Myotubularin-related protein 1	Q9Z2C4
QVFIFTGL	8		5.6	Metalloendopeptidase OMA1, mitochondrial	Q9D8H7
SSLLFVKL	8		5.6	Ribonuclease H2 subunit B	Q80ZV0
FVYVFHTL	8		5.7	Transmembrane glycoprotein NMB	Q99P91
RSLKFYSL	8		5.7	Pre-mRNA-processing factor 19	Q99KP6-1; Q99KP6-2; Q99KP6-3

SNLQYSLL	8	5.7	Multidrug resistance-associated protein 5	Q9R1X5
SSLEFTEL	8	5.7	Caspase-6	O08738
TAFQFLQL	8	5.7	Cyclin-G1	P51945
STIHFYSL	8	5.8	Protein transport protein Sec24A	Q3U2P1; Q3U2P1-2
SMILFTSL	8	5.8	Ataxin-10	P28658
FTFEYRYL	8	5.8	CCR4-NOT transcription complex subunit 3	Q8K0V4
KTFIFVRL	8	5.8	DNA helicase B	Q6NVF4
SSPLYTTL	8	5.8	Ribosome biogenesis protein NSA2 homolog	Q9CR47
VSYPFDTV	8	5.8	ADP/ATP translocase 1	P48962
KAFWYGQL	8	5.8	Zinc transporter ZIP11	Q8BWY7-2; Q8BWY7; Q8BWY7-3
LIYKFLNV	8	5.8	Exportin-1	Q6P5F9
VIFIFTKV	8	5.9	Glycosaminoglycan xylosylkinase	Q8VCS3
SPYKFRNL	8	5.9	5-phosphohydroxy-L-lysine phospho-lyase	Q8R1K4-2; Q8R1K4; Q8R1K4-3
VIFEMTNL	8	5.9	Insulin-like growth factor 1 receptor	Q60751
VAPRYVALL	9	5.9	Bifunctional glutamate/proline--tRNA ligase	Q8CGC7
VLYQFRGV	8	5.9	DnaJ homolog subfamily C member 11	Q5U458
STWVFPTL	8	5.9	Elongation factor 1-gamma	Q9D8N0
AVIHFAGL	8	5.9	UDP-glucose 4-epimerase	Q8R059
QNFMYTML	8	5.9	Protein SDA1 homolog	Q80UZ2-2; Q80UZ2; Q80UZ2-3
SAARFALL	8	5.9	Solute carrier family 12 member 6	Q924N4; Q924N4-2
KNFVYRTL	8	5.9	tRNA wybutosine-synthesizing protein 5	A2RSX7
ISVSFYHV	8	6	Eukaryotic translation initiation factor 3 subunit B	Q8JZQ9
SVYRVRNL	8	6	U4/U6 small nuclear ribonucleoprotein Prp3	Q922U1
SNLVFLGL	8	6	Zinc finger protein 728	Q6P5C7
VMYKFLTV	8	6.1	Caveolin-2	Q9WVC3
STLLYGQL	8	6.1	DnaJ homolog subfamily C member 10	Q9DC23
KTYHYLYL	8	6.1	Protein fem-1 homolog B	Q9Z2G0
KTWRFSNM	8	6.1	Fermitin family homolog 3	Q8K1B8; Q8CIB5
VAVKFVRL	8	6.1	T-complex protein 11-like protein 1	Q8BTG3
SRYQFRNL	8	6.2	Peptidyl-tRNA hydrolase ICT1, mitochondrial	Q8R035-2; Q8R035-1
INFDFPKL	8	6.2	Probable ATP-dependent RNA helicase DDX6	P54823
ATFHFRTL	8	6.2	AH receptor-interacting protein	O08915
TNINFPNL	8	6.2	Histone-lysine N-methyltransferase 2C	Q8BRH4-2; Q8BRH4
QGYTFLQL	8	6.2	Lysophosphatidylcholine acyltransferase 2	Q8BYI6-2; Q8BYI6
VAYWRQAGL	9	6.3	ATP synthase subunit epsilon, mitochondrial	P56382
VAFEYCQRL	9	6.3	AP-5 complex subunit zeta-1	Q3U829
SIYARFVQL	9	6.3	Polyphosphoinositide phosphatase	Q91WF7
TNYIFDSL	8	6.3	TBC1 domain family member 15	Q9CXF4
YQFVYQNL	8	6.3	Up-regulator of cell proliferation	Q5NCI0-2; Q5NCI0
RSYSFQKV	8	6.4	Isoform 2 of DIS3-like exonuclease 2	Q8CI75-2; Q8CI75
HGYIFSSL	8	6.4	Exosome complex component csl4	Q9DAA6-2; Q9DAA6-1
SQFKYALV	8	6.4	Developmentally-regulated GTP-binding protein 2	Q9QXB9
ISARFVQL	8	6.4	Cytoplasmic polyadenylation element-binding protein 2	Q812E0; Q7TN98-5; Q7TN99-2; Q7TN98; Q7TN99-4; Q7TN98-2; Q7TN99-6; Q7TN99-3; Q7TN98-3; Q7TN99; Q7TN98-4
SNLKYSLL	8	6.4	Protein dopey-2	Q3UHQ6
SALKYYQL	8	6.4	KIF1-binding protein	Q6ZPU9; Q6ZPU9-3
TQYIFNNM	8	6.4	Alpha-mannosidase 2	P27046
SAPLFTGL	8	6.4	Nuclear envelope pore membrane protein POM 121	Q8K3Z9
IALFFRSL	8	6.4	Transcription factor 25	Q8R3L2; Q8R3L2-4; Q8R3L2-2; Q8R3L2-3; Q8R3L2-5
STYKFFEV	8	6.5	60S ribosomal protein L15	Q9CZM2
TAFKFKAL	8	6.5	Neurofibromin	Q04690-3; Q04690; Q04690-4; Q04690-2
LNYAYTAQL	9	6.5	Influenza virus NS1A-binding protein homolog	Q920Q8-4; Q920Q8; Q920Q8-3; Q920Q8-2
VTVRFQKL	8	6.5	Low-density lipoprotein receptor-related protein 10	Q7TQH7
VVFFFKTL	8	6.5	Selenoprotein K	Q9JLJ1

VSFDYHQM	8		6.5	Synaptojanin-1	Q8CHC4
TNVEYAHL	8		6.6	Acyl-CoA dehydrogenase family member 10	Q8K370
RNFVFHTL	8		6.6	Claspin	Q80YR7
RNYLHYSL	8		6.6	26S proteasome non-ATPase regulatory subunit 3	P14685
TSLRFVFL	8		6.6	Reticulophagy regulator 3	Q9CQV4
QAYAFLQYL	9		6.6	Dolichyl-diphosphooligosaccharide--protein glycosyltransferase subunit STT3B	Q3TDQ1
YNALFANL	8		6.6	Gamma-tubulin complex component 6	G5E8P0-2; G5E8P0
SVYVYKVL	8		6.7	Histone H2B type 1-P	Q64475; Q8CGP2; Q64525; Q8CGP1; P10854; Q64478; Q8CGP2-2; Q6ZWY9; P10853
VTPRFPKL	8		6.7	Activating signal cointegrator 1 complex subunit 3	E9PZJ8-2; E9PZJ8-1
RQYIFSKL	8		6.8	Interferon-induced very large GTPase 1	Q80SU7
SILQYSNV	8		6.8	GATOR complex protein NPRL2	Q9WUE4
YTYLYVRM	8		6.8	Activating signal cointegrator 1 complex subunit 3	E9PZJ8-1
RGYDFAAV	8		6.8	EH domain-containing protein 1	Q9QXY6; Q9WVK4
VGPRYTQL	8		6.8	Mitogen-activated protein kinase 3	Q63844
VGYRFVTAI	9		6.8	Nischarin	Q80TM9-3; Q80TM9-2; Q80TM9
TGYNFQRV	8		6.8	Pleiotropic regulator 1	Q922V4
ATLVFHNL	8		6.9	Signal transducer and activator of transcription 3	P42227-3; P42227; P42227-2
STFFYPKL	8		6.9	Sentrin-specific protease 2	Q91ZX6-3; Q91ZX6-2; Q91ZX6
TSLPYYTGV	9		6.9	Ubiquitin-associated protein 2-like	Q80X50-4; Q80X50-3; Q80X50-5; Q80X50-2; Q80X50
SAYIFNSNV	9		6.9	Signal peptide peptidase-like 3	Q9CUS9
VNFSPANL	8		7	HAUS augmin-like complex subunit 1	Q8BHX1
STFVYNSM	8	1xOxidation [M8]	7	Guanylate-binding protein 4	Q61107
TNYNFQYI	8		7	glycerol-3-phosphate acyltransferase 4	Q8K2C8
KALSYASL	8		7	Rapamycin-insensitive companion of mTOR	Q6QI06; Q6QI06-2
RAYLFNSV	8		7	Protein yippee-like 1	Q9ESC7; Q65Z93; P61237; Q65Z95
SVIKFENL	8		7.1	Isoform 4 of Palmitoyltransferase ZDHHC6	Q9CPV7-3; Q9CPV7-2; Q9CPV7; Q9CPV7-4
VTYHGFPNL	9		7.1	N-glycosylase/DNA lyase	O08760
THYSFLATL	9		7.1	Major facilitator superfamily domain-containing protein 3	Q5U419
SGYDFGYM	8		7.1	CCR4-NOT transcription complex subunit 8	Q9D8X5
VSPEFHTL	8		7.2	Protein Hook homolog 2	Q7TMK6
SSFHFNQHL	9		7.2	Codanin-1	Q8CC12-2; Q8CC12-3; Q8CC12
RSIWFQQL	8		7.2	Motile sperm domain-containing protein 2	Q9CWP6
TSAHFARL	8		7.3	ankyrin repeat domain-containing protein 13A	Q80UP5
TSFTFRKV	8		7.3	Alkylated DNA repair protein alkB homolog 8	Q80Y20-2; Q80Y20
SGYHYVCL	8		7.3	1-phosphatidylinositol 4,5-bisphosphate phosphodiesterase beta-3	P51432
VAVIYAGV	8		7.4	ATP synthase subunit alpha, mitochondrial	Q03265
VGPMFLYL	8	1xOxidation [M4]	7.4	Cytochrome b-245 heavy chain	Q61093
VAPLYKRL	8		7.4	Methylcytosine dioxygenase TET3	Q8BG87; Q8BG87-4
KIITYRNL	8		7.5	PCI domain-containing protein 2	Q8BFV2
VSFPFGKI	8		7.5	Protein RRP5 homolog	Q6NS46
VIQVFQQL	8		7.5	dephospho-CoA kinase domain-containing protein	Q8BHC4
KSITFSKL	8		7.5	Delta(3,5)-Delta(2,4)-dienoyl-CoA isomerase, mitochondrial	O35459
KGYIFLTL	8		7.6	E3 ubiquitin-protein ligase TRIM56	Q80VI1
VNRSFIAL	8		7.6	Receptor expression-enhancing protein 5	Q60870
RNLDYARL	8		7.6	Serine hydroxymethyltransferase, cytosolic	P50431
ATYTFIQQL	9		7.6	galactokinase	Q9R0N0
VAPSFGTL	8		7.6	Nucleoporin NUP188 homolog	Q6ZQH8
RGFKYLRL	8		7.6	Transcription activator BRG1	Q3TKT4; Q3TKT4-2
STFSFTKV	8		7.7	Extended synaptotagmin-2	Q3TZZ7-1
SNTQYARL	8		7.7	Very long-chain specific acyl-CoA dehydrogenase, mitochondrial	P50544
SGFEFTSV	8		7.7	Putative tRNA pseudouridine synthase Pus10	Q9D3U0
FAYRFSNLL	9		7.7	Periodic tryptophan protein 2 homolog	Q8BU03
SQYVFTEM	8	1xOxidation [M8]	7.8	Transmembrane protein 199	Q5SYH2

IQWAFKNL	8		7.8	Ubiquitin carboxyl-terminal hydrolase 34	Q6ZQ93-1; Q6ZQ93-3; Q6ZQ93-2
SSFLFWRM	8		7.8	protein AF1q	P97783
VGFTFPNRL	9		7.8	Neurochondrin	Q9Z0E0-2; Q9Z0E0-1
VSVSFRVL	8		7.8	UDP-GalNAc:beta-1,3-N-acetylgalactosaminyltransferase 2	Q8BG28; Q8BG28-2
SIYAPARL	8		7.8	Cyclin-F	P51944-2; P51944; P51944-3
YNFQYISL	8		7.8	Glycerol-3-phosphate acyltransferase 4	Q8K2C8
KNVLFSHL	8		7.9	cAMP-dependent protein kinase type I-beta regulatory subunit	P12849; Q9DBC7
HIYEFPQL	8		7.9	Protein unc-119 homolog B	Q8C4B4
RSLRFVTL	8		7.9	Lysosomal acid phosphatase	P24638
ISLDYHQL	8		7.9	E3 ubiquitin-protein ligase DZIP3	Q7TPV2
TAYLFSRF	8		8.1	Exportin-T	Q9CRT8
SAPQYSRL	8		8.1	Extracellular sulfatase Sulf-2	Q8CFG0
INLSFNKL	8		8.1	Leucine-rich repeat-containing protein 40	Q9CRC8
ANHRYANV	8		8.1	Bromodomain-containing protein 8	Q8R3B7-1; Q8R3B7-2
RTYSFLNLL	9		8.1	Transmembrane protein 39B	Q810L4
SSPQFVGNL	9		8.2	Cyclin-H	Q61458
STIVYYKL	8		8.2	tRNA-splicing endonuclease subunit Sen15	Q8R3W5
VNYEFGIAL	9		8.2	Uncharacterized protein C12orf29 homolog	Q8BHN7; Q8BHN7-2
STLIYRNM	8	1xOxidation [M8]	8.3	Protein lin-37 homolog	Q9D8N6
NGYKYMAL	8		8.3	alpha-galactosidase A	P51569
KSRIFQNL	8		8.3	Multifunctional procollagen lysine hydroxylase and glycosyltransferase LH3	Q9R0E1
SNLLYVNV	8		8.3	Prolyl endopeptidase-like	Q8C167-3; Q8C167; Q8C167-2
IISTFQNL	8		8.3	Protein SCAF8	Q6DID3
LGYLYRCL	8		8.3	XK-related protein 8	Q8C0T0
SAMVFSAM	8	2xOxidation [M3; M8	8.4	V-type proton ATPase 16 kDa proteolipid subunit	P63082
VNVRFTGV	8		8.5	Ubiquitin carboxyl-terminal hydrolase MINDY-1	Q76LS9-2; Q6PDI6-3; Q6PDI6; Q76LS9-3; Q76LS9-1; Q6PDI6-2
INIEMYQRL	9		8.5	RUN and FYVE domain-containing protein 2	Q8R4C2
SSHSFPQL	8		8.5	Angiomotin-like protein 2	Q8K371
INYDHYGTM	9		8.5	C5a anaphylatoxin chemotactic receptor 1	P30993
SNYRVSLL	8		8.5	Isoform 2 of CLIP-associating protein 2	Q8BRT1-5
VNYPFIDM	8		8.5	ETS domain-containing transcription factor ERF	P70459
VNYHYMSQV	9		8.6	Baculoviral IAP repeat-containing protein 6	O88738-2; O88738; O88738-3
SALEFLTHL	9		8.6	BRCA1-associated ATM activator 1	Q8C3R1-2; Q8C3R1
VNQKFNNL	8		8.6	Disks large-associated protein 5	Q8K4R9; Q8K4R9-3; Q8K4R9-2
LQYCFPRL	8		8.6	DNA primase small subunit	P20664
TSVQFMKL	8		8.7	Fatty acid synthase	P19096
VGYVLGQL	8		8.7	Deoxyhypusine hydroxylase	Q99LN9
AQYRFIYM	8		8.7	Tyrosine-protein phosphatase non-receptor type 11	P35235; P35235-2
VIYEPFQLL	9		8.7	Testis-expressed protein 2	Q6ZPJ0
SAALFSRL	8		8.7	Mitochondrial import inner membrane translocase subunit TIM50	Q9D880
ASRIFPHL	8		8.8	Protein phosphatase Slingshot homolog 3	Q8K330-2; Q8K330
RALNYTHL	8		8.8	nuclear pore complex protein Nup98-Nup96	Q6PFD9
SIYRFHGQF	9		8.8	FYVE, RhoGEF and PH domain-containing protein 3	O88842-2; O88842
TAFTYEQL	8		8.8	NK1 transcription factor-related protein 2	P42580
SSLRFTTI	8		8.8	WD repeat-containing protein 43	Q6ZQL4
YAMIYRNL	8	1xOxidation [M3]	8.9	E3 ubiquitin-protein ligase Mdm2	P23804-1; P23804-2
VQWEYGRL	8		8.9	Glutamine--tRNA ligase OS=Mus musculus OX=10090	Q8BML9
KQYPYNNL	8		8.9	NADH dehydrogenase [ubiquinone] 1 beta subcomplex subunit 8, mitochondrial	Q9D6J5
SSFSYQFSDL	10		8.9	Emerin	O08579
RILEFYSKL	9		8.9	Protein O-GlcNAcase	Q9EQQ9; Q9EQQ9-3; Q9EQQ9-2
RVYYFNHI	8		9	peptidyl-prolyl cis-trans isomerase NIMA-interacting 1	Q9QUR7
VRYDFSGL	8		9	Ceramide kinase	Q8K4Q7
VAFDFYHKASRL	12		9	E3 ubiquitin-protein ligase UBR1	O70481
SNIQYRSL	8		9.1	neuron navigator 1	Q8CH77-2; Q8CH77; Q8CH77-3; Q8CH77-4

TNLRYLAL	8		9.1	AP-2 complex subunit alpha-2	P17426-2; P17427; P17426
RNILYHNL	8		9.1	Cell surface hyaluronidase	Q5FWI3
SIDQFANL	8		9.1	U6 snRNA-associated Sm-like protein LSm1	Q8VC85
VCMAFAGL	8		9.1	Proteasome subunit alpha-type 7-like	Q9CWH6; Q9Z2U0
ANLIYYSL	8		9.2	ATP synthase subunit gamma, mitochondrial	Q91VR2
YNWRYKNL	8		9.2	RNA helicase aquarius	Q8CFQ3
IACKFAEL	8		9.2	Elongation factor 1-alpha 1	P10126; P62631
RGFFFSHV	8		9.2	Acyl-CoA desaturase 3	Q99PL7; Q6T707; P13011; P13516
TQYLFIKL	8		9.3	Probable Leucine--tRNA ligase, mitochondrial	Q8VDC0
VNYRVPNM	8		9.3	1-phosphatidylinositol 4,5-bisphosphate phosphodiesterase gamma-1	Q62077
SSLRYLQV	8		9.3	Ankyrin repeat domain-containing protein 54	Q91WK7
SSPKFSEL	8		9.3	Cytoskeleton-associated protein 5	A2AGT5; Q91VM3-2; A2AGT5-3; Q91VM3; A2AGT5-2
NSFRYNGL	8		9.3	60S ribosomal protein L28	P41105
VGYLHEGL	8		9.3	U5 small nuclear ribonucleoprotein 200 kDa helicase	Q6P4T2
TNLVYFGL	8		9.3	Protein TEX261	Q62302
RTYTYEKL	8		9.4	Catenin beta-1	Q02248
SNLYYKYL	8		9.4	A-kinase anchor protein 10, mitochondrial	O88845-2; O88845-3; O88845-1
VAYLMQKL	8		9.4	Dual specificity protein phosphatase 6	Q9DBB1
RSYLFLGGI	9		9.4	Bax inhibitor 1	Q9D2C7
ITFSYVNNM	9	1xOxidation [M9]	9.4	Ceramide synthase 6	Q8C172
GVFSFSRL	8		9.4	Transmembrane protein 260	Q8BMD6
VIQPFSSL	8		9.4	Bombesin receptor-activated protein C6orf89 homolog	Q99KU6
VNTHFSHL	8		9.5	Tetratricopeptide repeat protein 21B	Q0HA38
VAYKFPELL	9		9.5	E3 ubiquitin-protein ligase UBR2	Q6WKZ8-1; Q6WKZ8-3; Q6WKZ8-2
VSYPHLVLL	9		9.5	Tyrosine-protein kinase JAK3	Q62137; Q62137-2
ATIFFTRL	8		9.5	Phosphatidylinositol 4-phosphate 3-kinase C2 domain-containing subunit alpha	Q61194; Q61194-2
VAIRFDSGL	9		9.5	Zinc finger protein 22	Q9ERU3
KVFEFYLL	8		9.6	Probable C-mannosyltransferase DPY19L4	A2AJQ3-2; A2AJQ3-1
RVYKFCSKL	9		9.6	CREB-binding protein	P45481
AVLNFTAL	8		9.6	E3 ubiquitin-protein ligase HUWE1	Q7TMY8-4; Q7TMY8-3; Q7TMY8; Q7TMY8-2
IGYFYGQGL	9		9.6	Mannosyl-oligosaccharide glucosidase	Q80UM7
AAYGFRNI	8		9.6	Nuclear prelamin A recognition factor	Q9CYQ7
IVWEFEQL	8		9.6	Zinc finger protein RFP	Q62158
SNALFAKL	8		9.7	Ankyrin repeat domain-containing protein 13B	Q5F259; Q5F259-2
KGYVFKEL	8		9.8	Toll-like receptor 7	P58681
TNVQYSNL	8		9.8	Cytoplasmic dynein 2 heavy chain 1	Q45VK7; Q45VK7-2
STRLFAVL	8		9.8	Dolichyl-diphosphooligosaccharide--protein glycosyltransferase subunit STT3A	P46978
TNYNFQYISL	10		9.9	glycerol-3-phosphate acyltransferase 4	Q8K2C8
KSYLMNRL	8		9.9	Guanylate-binding protein 4	Q61107
TNFEYLTHL	9		10	Lysosomal-trafficking regulator	P97412-1
AVVRFPRL	8		10	Integrator complex subunit 7	Q7TQK1; Q7TQK1-2
KSYEFEDL	8		10.1	DENN domain-containing protein 2A	Q8C4S8
VSQYYPKL	8		10.1	Heterochromatin protein 1-binding protein 3	Q3TEA8; Q3TEA8-3; Q3TEA8-2
SGREFVNL	8		10.1	Ribosome biogenesis protein TSR3 homolog	Q5HZH2
SIVSYNHL	8		10.2	Inositol-3-phosphate synthase 1	Q9JHU9
LGYKYVGM	8	1xOxidation [M8]	10.2	Protein YIF1B	Q9CX30; Q9CX30-2
TNLVYPAL	8		10.4	Hypoxia-inducible factor 1-alpha inhibitor	Q8BLR9-1; Q8BLR9-2
VVYTPWSNL	9		10.4	Coiled-coil domain-containing protein 25	Q78PG9
IAYLYDRLL	9		10.4	DNA replication complex GINS protein PSF1	Q9CZ15
AVYQFGSAL	9		10.4	Membrane progestin receptor alpha	Q80ZE4
SIYDPFAGM	9		10.4	Phosphoinositide 3-kinase adapter protein 1	Q9EQ32-2; Q9EQ32
RAFEFTYV	8		10.4	dTDP-D-glucose 4,6-dehydratase	Q8VDR7
KVYTFNSV	8		10.5	Plasma membrane calcium-transporting ATPase 4	Q6Q477-2; Q6Q477; G5E829; Q9R0K7
TNISFTNM	8		10.5	PRELI domain containing protein 3B	Q9CYY7

VAFNYKTV	8		10.5	Prostaglandin reductase 1	Q91YR9
AIVSFAHV	8		10.5	DNA mismatch repair protein Msh2	P43247
VAFDFTKV	8		10.6	Phosphatidylinositol-binding clathrin assembly protein	Q7M6Y3-1; Q7M6Y3-5; Q7M6Y3-6; Q7M6Y3-4; Q7M6Y3-2; Q7M6Y3-3
STYGWTANM	9		10.6	Heat shock protein HSP 90-beta	P07901; P11499
VIFEMVHL	8		10.6	Insulin receptor	P15208
HTFTYTGL	8		10.7	Mediator of RNA polymerase II transcription subunit 12	A2AGH6-2; A2AGH6-1
VNLEYLARV	9		10.7	Cytoplasmic aconitate hydratase	P28271
IGLDYSSL	8		10.7	Class E basic helix-loop-helix protein 41	Q99PV5
QSPGFYRNV	9		10.7	Tryptophan--tRNA ligase, cytoplasmic	P32921-2; P32921
LQYEFTKL	8		10.8	Formin-like protein 3	A2APV2-3; Q6ZPF4-2; Q6ZPF4-1; A2APV2-1; A2APV2-2
QAFKFRRV	8		10.8	E3 ubiquitin-protein ligase UBR2	Q6WKZ8-1; Q6WKZ8-3; Q6WKZ8-2
SAFDFENM	8	1xOxidation [M8]	10.8	Rab GDP dissociation inhibitor alpha	P50396
IAVSFREL	8		10.8	Nucleolar protein 11	Q8BJW5-2; Q8BJW5
YGYHFPEL	8		10.8	Nucleolar protein 56	Q9D6Z1
GSYQFSMV	8	1xOxidation [M7]	10.8	Runt-related transcription factor 1	Q03347; Q03347-2; Q03347-4
SSLSFNTRL	9		10.9	Vesicle transport protein SFT2A OS=Mus musculus OX=10090	Q5SSN7
KNIRYVAL	8		10.9	AP-1 complex subunit gamma-1	O88512; P22892
LAPAYALL	8		10.9	5-demethoxyubiquinone hydroxylase, mitochondrial	P97478
KSLDYLNL	8		10.9	UNC119-binding protein C5orf30 homolog	Q8VEB3
IIIIYNRV	8		10.9	Heparan sulfate 2-O-sulfotransferase 1	Q8R3H7
SSPGYSHL	8		10.9	Low-density lipoprotein receptor-related protein 10	Q7TQH7
RSIDQFANL	9		10.9	U6 snRNA-associated Sm-like protein LSm1	Q8VC85
RSPWFTTL	8		11	MLV-related proviral Env polyprotein	P10404; P11370
SNHVFNAL	8		11	serine/threonine-protein kinase RIO3	Q9DBU3
SVVAYNNL	8		11	Transcriptional adapter 1	Q99LM9
VNLTFRTV	8		11	Alpha-ketoglutarate-dependent dioxygenase alkB homolog 3	Q8K1E6
SSPAYPSL	8		11	Autophagy-related protein 2 homolog B	Q80XK6
SQIRFGLL	8		11	Conserved oligomeric Golgi complex subunit 1	Q9Z160
KIIEFANI	8		11	Meiosis-specific nuclear structural protein 1	Q61884
VVHRFESL	8		11	Torsin-1A-interacting protein 1	Q921T2; Q921T2-2; Q921T2-3
VTWRVTNL	8		11	MLV-related proviral Env polyprotein	P10404
RNYQFDFL	8		11.1	splicing factor 3A subunit 1	Q8K4Z5
TNVLFNHL	8		11.1	RNA polymerase II-associated protein 3	Q9D706
SVVNFTSL	8		11.1	Histone-lysine N-methyltransferase ASH1L	Q99MY8
IIITFNDL	8		11.1	Peptide-N(4)-(N-acetyl-beta-glucosaminyl)asparagine amidase	Q9JI78
VQMKFRLL	8		11.2	G2/mitotic-specific cyclin-B1	P24860
TGYQYPTM	8	1xOxidation [M8]	11.2	protein NDRG3	Q9QYF9
RAIAFQHL	8		11.2	Myb-binding protein 1A	Q7TPV4
KNFPFERL	8		11.2	U5 small nuclear ribonucleoprotein 200 kDa helicase	Q6P4T2
IGPRFVLNL	9		11.2	Ribosome biogenesis protein BRX1 homolog	Q9DCA5
AMYSFPQI	8		11.2	Alanine aminotransferase 1	Q8QZR5
VINVFHHL	8		11.3	Cyclin-L1	Q52KE7-2; Q52KE7
ATLEFTQL	8		11.3	Nitric oxide synthase, inducible	P29477
VIHIFSHI	8		11.4	Adenine DNA glycosylase	Q99P21
QALKYFNL	8		11.4	Protein sel-1 homolog 1	Q9Z2G6; Q9Z2G6-2
AIRVFANI	8		11.5	eukaryotic translation initiation factor 3 subunit L	Q8QZY1
KGFRFTLV	8		11.5	Dolichyl pyrophosphate Man9GlcNAc2 alpha-1,3-glucosyltransferase	Q3TAE8
STRVYASM	8		11.6	Transforming growth factor beta regulator 1	Q3UB74
RNYSYEKL	8		11.6	Junction plakoglobin	Q02257
VALKFSQI	8		11.6	Transcription termination factor 1	Q62187
KSLEYHHL	8		11.6	Zinc finger SWIM domain-containing protein 6	Q80TB7; Q80TB7-2
KGYLFNTV	8		11.7	M-phase inducer phosphatase 1	P48964
VTFDYEEL	8		11.7	Protein FAM111A	Q9D2L9
EVYSFSGL	8		11.7	Oxidative stress-responsive serine-rich protein 1	Q9D722

RSPRYRLL	8		11.8	Replication protein A 70 kDa DNA-binding subunit	Q8VEE4
SSYQHTSV	8		11.8	Exportin-T	Q9CRT8
SSVLFETL	8		11.8	Monocarboxylate transporter 1	P53986
RNYEYLIRL	9		11.9	Transcription factor 25	Q8R3L2-1; Q8R3L2-4; Q8R3L2-2; Q8R3L2-3; Q8R3L2-5
TAPQYYRL	8		11.9	DALR anticodon-binding domain-containing protein 3	Q6PJN8
AVFTWTNL	8		12	Disco-interacting protein 2 homolog A	Q8BWT5; Q3UH60-2; Q3UH60-1
IAYLYNEGL	9		12	Cyclin-F	P51944-2; P51944; P51944-3
VTPEGYAHL	9		12	Histone deacetylase 6	Q9Z2V5
RNLEYLNL	8		12.1	F-box/LRR-repeat protein 2	Q8BH16
RNPTFMGL	8	1xOxidation [M6]	12.1	AP-2 complex subunit alpha-2	P17427
IDYEFSAL	8		12.1	5'-3' exoribonuclease 1	P97789-2; P97789-3; P97789
SSPHYTTL	8		12.2	Alpha-mannosidase 2	P27046
VGVTYRTL	8		12.2	Protein MMS22-like	B1AUR6-1
SSVKFNPV	8		12.2	DDB1- and CUL4-associated factor 13	Q6PAC3
VAYRHLVGV	9		12.3	Cytochrome c1, heme protein, mitochondrial	Q9D0M3; Q9D0M3-2
AVYTFETLL	9		12.3	Niban-like protein 1	Q8R1F1
SSLGFERL	8		12.3	Proline-rich protein 12	E9PYL2
VSYWFDQRF	9		12.4	CMP-N-acetylneuraminate-beta-galactosamide-alpha-2,3-sialyltransferase 1	P54751
SGYIPARL	8		12.5	Serine/threonine-protein kinase PLK1	Q07832
NAFKFMSL	8		12.5	Vascular endothelial growth factor receptor 1	P35969
TALRYYEL	8		12.6	Intraflagellar transport protein 140 homolog	E9PY46
QGYEYDNL	8		12.6	Meckel syndrome type 1 protein homolog	Q5SW45
VINVFHRL	8		12.7	cyclin-L2	Q9JJA7-2; Q9JJA7-3; Q9JJA7-1
CIINFQHL	8		12.7	Activating signal cointegrator 1 complex subunit 3	E9PZJ8-1
SNFVFARTM	9		12.7	Insulin-like growth factor 1 receptor	Q60751
QILWFRGL	8		12.7	Plasma membrane calcium-transporting ATPase 1	G5E829; Q9R0K7
SLYRFTTI	8		12.7	DEP domain-containing protein 7	Q91WS7
STYLRLQLL	9		12.7	Extracellular serine/threonine protein kinase FAM20C	Q5MJS3
SHYEFHNI	8		13	Phosphatidylinositol 3,4,5-trisphosphate-dependent Rac exchanger 1 protein	Q69ZK0
SSFSWTNGL	9		13	Transmembrane protein 138	Q9D6G5
YNFDFSKL	8		13	Nucleolar pre-ribosomal-associated protein 1	Q571H0
TSPHYQNL	8		13.1	Tyrosine-protein phosphatase non-receptor type 18	Q61152
VSTKFEHL	8		13.1	trafficking protein particle complex subunit 11	B2RXC1
QQYLFDRL	8		13.1	Nardilysin	Q8BHG1
HQYQFNNL	8		13.2	Integrin alpha-M	P05555-1; P05555-2
TGLSFADL	8		13.2	E3 SUMO-protein ligase RanBP2	Q9ERU9
AGFTRFPSL	9		13.2	Dehydrogenase/reductase SDR family member 4	Q99LB2
KTYQHFTL	8		13.2	Alpha-1,3-mannosyl-glycoprotein 4-beta-N-acetylglucosaminyltransferase B	Q812F8
IAPAFSSM	8		13.2	Thioredoxin-like protein 1	Q8CDN6
YGVLFRNL	8		13.3	Leukocyte surface antigen CD53	Q61451
RIYGFTAV	8		13.3	E3 ubiquitin-protein ligase hectd1	Q69ZR2
TSLAFESRL	9		13.3	Cytospin-B	Q5SXY1-1; Q5SXY1-2
IVYTFMTHF	9		13.3	Ubiquitin carboxyl-terminal hydrolase 34	Q6ZQ93-1; Q6ZQ93-3; Q6ZQ93-2
TALAFRTL	8		13.3	MAP/microtubule affinity-regulating kinase 4	Q8CIP4
QVFKYRKL	8		13.4	lysosomal acid lipase/cholesteryl ester hydrolase	Q9Z0M5
IAYQFLRA	8		13.4	structural maintenance of chromosomes protein 2	Q8CG48
TAVKFSQL	8		13.4	Collectin-12	Q8K4Q8
ANVVFTQL	8		13.4	Arf-GAP with GTPase, ANK repeat and PH domain-containing protein 3	Q8VHH5
SAVVFRHM	8		13.4	Protocadherin Fat 3	Q8BNA6
QSIAFISRL	9		13.5	Transmembrane protein 33	Q9CR67
VIWKYPTM	8		13.5	Cell division cycle protein 20 homolog	Q9JJ66
RAFTYSTV	8		13.5	Solute carrier family 17 member 9	Q8VCL5
VSYLRKISL	9		13.5	Vacuolar protein sorting-associated protein 13C	Q8BX70-3; Q8BX70-2; Q8BX70
KAYIFEGAL	9		13.6	Vacuolar ATPase assembly integral membrane protein vma21	Q78T54

KNLRYQLL	8		13.6	Matrin-3	Q8K310
RSYQQALL	8		13.6	SERTA domain-containing protein 3	Q9ERC3
VNSIFQHL	8		13.7	Interferon-induced very large GTPase 1	Q80SU7
KNFAFTMV	8		13.7	Phospholipid-transporting ATPase ID	P98199
SSYTFPKMM	9		13.7	Enoyl-CoA delta isomerase 2, mitochondrial	Q9WUR2; Q9WUR2-2
VIQKFLYL	8		13.7	Guanine deaminase	Q9R111
KCYLFGGL	8		13.8	Host cell factor 1	Q61191
VITEFARI	8		13.9	tRNA (guanine(26)-N(2))-dimethyltransferase	Q3TX08
ESFKFVRL	8		13.9	Translation initiation factor eIF-2B subunit alpha	Q99LC8
VAFKHLFL	8		13.9	Mucolipin-2	Q8K595-2; Q8K595
AVLRYTKL	8		14	Replication factor C subunit 2	Q9WUK4
VTVVFSTV	8		14	Actin-related protein 2/3 complex subunit 2	Q9CVB6
TGPDYYSQL	9		14	Histone-lysine N-methyltransferase 2D	Q6PDK2
IGPRFKLL	8		14	Phosphatidylinositol 4-kinase alpha	E9Q3L2
VNLAYENV	8		14.1	Cytoplasmic dynein 1 heavy chain 1	Q9JHU4
IVFEDFARL	9		14.1	Beta-arrestin-2	Q91YI4-1; Q91YI4-2
TTYAFFNTF	9		14.1	Beta-hexosaminidase subunit beta	P20060
VGWKYQAV	8		14.1	60S ribosomal protein L13a	P19253
SGYDFENRL	9		14.2	Tetratricopeptide repeat protein 39C	Q8VE09
TAVSFSSL	8		14.2	Transmembrane 7 superfamily member 3	Q9CRG1
AIVEFLSNL	9		14.3	Chromatin assembly factor 1 subunit B	Q9D0N7
SSVYFRSV	8		14.4	BLOC-1-related complex subunit 8	Q9D6Y4
RGLSRYPNL	9		14.4	protein farnesyltransferase/geranylgeranyltransferase type-1 subunit alpha	Q61239
TIIVFHSL	8		14.4	Eyes absent homolog 1	P97767; Q9Z191; P97767-2
SGLKYVNV	8		14.6	Adenylate kinase isoenzyme 6	Q8VCP8
KNFAFTLV	8		14.6	Phospholipid-transporting ATPase IC	Q148W0; A3FIN4-1
RGLDYFSSL	9		14.6	Transmembrane protein 245	B1AZA5
STFIYNSI	8		14.6	Guanylate-binding protein 1	Q01514; Q9Z0E6
VTIHYNKL	8		14.6	PRKCA-binding protein	Q62083
AVFRFKVL	8		14.6	STARD3 N-terminal-like protein	Q9DCI3
ITGMFQRL	8		14.7	elongation factor 1-gamma	Q9D8N0
KNLNYLHL	8		14.7	Pre-mRNA-processing-splicing factor 8	Q99PV0
RGPLFSHL	8		14.7	Zinc transporter ZIP6	Q8C145
RGYEFIVRL	9		14.8	Serine/threonine-protein kinase ATR	Q9JKK8
SIMAFHKL	8	1xOxidation [M3]	14.8	Myotubularin-related protein 10	Q7TPM9
RGYIYWRL	8		14.9	AP-1 complex subunit beta-1	Q9DBG3-2; O35643; Q9DBG3
TSPTYRSL	8		14.9	Regulator of G-protein signaling 19	Q9CX84
IGPYYRKL	8		15	Transmembrane and coiled-coil domain-containing protein 3	Q8BH01; Q8BH01-2
SILRYLARI	9		15	Bifunctional glutamate/proline--tRNA ligase	Q8CGC7
VTYSFRQSF	9		15	IQ motif and SEC7 domain-containing protein 2	Q8R0S2-2; Q5DU25; Q8R0S2
KNFKFLGTL	9		15	Ras-responsive element-binding protein 1	Q3UH06-2; Q3UH06-3; Q3UH06-1
LILDYRNL	8		15	Magnesium transporter MRS2 homolog, mitochondrial	Q5NCE8
IAPAYVEL	8		15	Sacsin	Q9JLC8-3; Q9JLC8; Q9JLC8-2
HTYVHATL	8		15	Two pore calcium channel protein 1	Q9EQJ0-2; Q9EQJ0
TEYVFTHL	8		15.1	Fibronectin type III domain-containing protein 3B	Q6NWW9
AFYTVAHL	8		15.1	Leucine--tRNA ligase, cytoplasmic	Q8BMJ2
VNFPFLVKL	9		15.1	cAMP-dependent protein kinase catalytic subunit alpha	P05132-2; P05132
TVIIYVRL	8		15.1	Dolichyl-diphosphooligosaccharide--protein glycosyltransferase subunit 1	Q91YQ5
KGLDFALL	8		15.2	Protein Red	Q9Z1M8
VNVRFSTI	8		15.2	Dual specificity protein phosphatase 1	P28563
SSPVYIDL	8		15.2	Uridine 5'-monophosphate synthase	P13439
IILKYIGM	8		15.3	ADP-ribosylation factor-like protein 6-interacting protein 1	Q9JKW0
AQFRYLQRL	9		15.3	Phospholipid-transporting ATPase IC	Q148W0
VAITYKEL	8		15.3	Interferon-induced, double-stranded RNA-activated protein kinase	Q03963

VTVDFSKL	8		15.3	DNA helicase B	Q6NVF4
SSTYFHQL	8		15.4	Transcriptional regulator Kaiso	Q8BN78
QNYSYSSI	8		15.4	Ubiquitin carboxyl-terminal hydrolase 47	Q8BY87-2; Q8BY87
IGVIFTHV	8		15.5	Ribonuclease 3	Q5HZJ0
GVLKFARL	8		15.5	Protein C10	O35127
GVLEYANL	8		15.6	Arginyl-tRNA--protein transferase 1	Q9Z2A5-2; Q9Z2A5
FSQEYINL	8		15.7	Minor histocompatibility antigen H13	Q9D8V0; Q9D8V0-3; Q9D8V0-2; Q9D8V0-4
AAVKFHNL	8		15.8	Glycerol-3-phosphate dehydrogenase, mitochondrial	Q64521
SNATFARV	8		15.8	DNA polymerase delta subunit 2	O35654
FQFTFKHL	8		15.8	DnaJ homolog subfamily A member 2	Q9QYJ0
VNVPFHLAL	9		15.8	Rab3 GTPase-activating protein non-catalytic subunit	Q8BMG7
GILTFSNL	8		15.8	Nodal modulator 1	Q6GQT9
TAISFNLL	8		15.8	Natural resistance-associated macrophage protein 1	P41251
RGYEFLGV	8		15.9	F-box/SPRY domain-containing protein 1	Q8K3B1
VNYDFGHMHV	10		15.9	E3 ubiquitin-protein ligase RNF216	P58283-2; P58283
TTFEYQDL	8		15.9	Receptor-type tyrosine-protein phosphatase-like N	Q60673
KAFHFSSQL	9		15.9	Zinc finger protein 60	P16374
SGYEFIHKL	9		16	Tyrosine--tRNA ligase, mitochondrial	Q8BYL4
YEYLFTNL	8		16.1	Protein artemis	Q8K4J0-3; Q8K4J0-2; Q8K4J0
TSYFTSGL	8		16.1	Ribonucleoprotein PTB-binding 1	Q9CW46
VIWGKYAQV	9		16.2	Ribosome biogenesis protein NSA2 homolog	Q9CR47
SVYLVRQL	8		16.2	E3 SUMO-protein ligase PIAS3	Q8C5D8-2; O54714-2; O54714; Q8C5D8-5; Q8C5D8-4; O54714-3; Q8C5D8-3; Q8C5D8
RTFEFQLM	8		16.3	Programmed cell death protein 2-like	Q8C5N5
TAYLFSRFV	9		16.5	Exportin-T	Q9CRT8
ANFRFTDRL	9		16.6	Haptoglobin	Q61646
STLKFLKL	8		16.6	Nucleotide-binding oligomerization domain-containing protein 2	Q8K3Z0; Q8K3Z0-2
TSVVFNKL	8		16.7	DNA-directed RNA polymerase I subunit RPA12	Q791N7
AGPWYRNL	8		16.7	Probable phospholipid-transporting ATPase IIB	P98195-2; P98195-1
KTFSYAGF	8		16.7	T-complex protein 1 subunit eta	P80313
YSLEYGFL	8		16.7	Membralin	Q8CIV2; Q8CIV2-2
SNVKYVML	8	1xOxidation [M7]	16.8	Protein patched homolog 1	Q61115
STFEFHSI	8		16.8	E3 ubiquitin-protein ligase UBR1	O70481
KTYEHFNAM	9		16.8	NADPH--cytochrome P450 reductase	P37040
ISTIFKSL	8		17	Ubiquitin conjugation factor E4 B	Q9ES00
ASPIFTHV	8		17.1	Pre-mRNA-splicing factor CWC22 homolog	Q8C5N3; Q8C5N3-2
RGYDFAAVL	9		17.1	EH domain-containing protein 1	Q9QXY6; Q9WVK4
SNPEFRQL	8		17.3	Lysine-specific demethylase 7A	Q3UWM4
RILEFFGL	8		17.3	Protein disulfide-isomerase	P09103
KTFLFSATM	9	1xOxidation [M9]	17.4	Probable ATP-dependent RNA helicase DDX47	Q9CWX9
ISPRFDVQL	9		17.4	40S ribosomal protein S15a	P62245
SCIRFINL	8		17.5	SLIT-ROBO Rho GTPase-activating protein 3	Q812A2; Q91Z69
LVYKNFPQL	9		17.5	Transmembrane protein 41B	Q8K1A5; Q8K1A5-2
SAVSFHSL	8		17.6	Laccase domain-containing protein 1	Q8BZT9
RIFQFQNF	8		17.7	Pre-rRNA-processing protein TSR1 homolog	Q5SWD9-1; Q5SWD9-2
IVELFRNL	8		17.8	Signal transducer and activator of transcription 3	P42227-3; P42227; P42227-2
VNVEFVRV	8		17.9	[F-actin]-methionine sulfoxide oxidase MICAL2	Q8BML1-3; Q8BML1; Q8BML1-2
RIYRFDTV	8		17.9	Isoform 2 of Myomegalin	Q80YT7-2
SNLQFIDL	8		17.9	Solute carrier family 23 member 2	Q9EPR4
VSYRWICEM	9		18.1	C-type lectin domain family 5 member A	Q9R007; Q9R007-2; Q9R007-1
SLLRFNAL	8		18.1	Piezo-type mechanosensitive ion channel component 1	E2JF22
KVFEYHNV	8		18.2	Amyloid protein-binding protein 2	Q9DAX9
VVYPFPTF	8		18.2	Isoform 2 of DDB1- and CUL4-associated factor 15	Q6PFH3-1; Q6PFH3-2
VNYLRTVSL	9		18.2	Prokineticin receptor 1	Q9JKL1; Q8K458

RSYSFLNSSL	10		18.3	Deoxyribonuclease-1-like 1	Q9D7J6
SQYDFIDL	8		18.3	Dipeptidyl peptidase 8	Q80YA7
IGYPFLVSV	9		18.3	Ubiquitin carboxyl-terminal hydrolase 19	Q3UJD6-2; Q3UJD6
TIIIFHSL	8		18.4	eyes absent homolog 2	O08575; O08575-2; P97480; P97480-2
VIVEFRDL	8		18.4	Protein arginine N-methyltransferase 7	Q922X9
VIPVYVGV	8		18.4	Krev interaction trapped protein 1	Q6S5J6; Q6S5J6-2; Q6S5J6-3
ESYSFEARM	9		18.5	Zinc finger MYND domain-containing protein 19	Q9CQG3-1
VCWAFSSL	8		18.5	Importin subunit beta-1	P70168
RGFEFTLM	8		18.5	Septin-7	O55131
VSFDFHGRRM	10		18.6	nucleoporin Seh1	Q8R2U0-1; Q8R2U0-2
TTFSYVNNM	9		18.6	Ceramide synthase 5	Q9D6K9-2; Q9D6K9
VHYKYTVV	8		18.6	Neutrophil cytosol factor 2	O70145
TTLIFQKL	8		18.8	Proto-oncogene c-Rel	P15307
AQYKFIYV	8		18.9	Tyrosine-protein phosphatase non-receptor type 6	P29351; P29351-2; P29351-3
RSPAFTSRL	9		19	E3 ubiquitin-protein ligase HUWE1	Q7TMY8-4; Q7TMY8-3; Q7TMY8; Q7TMY8-2
KVFIFRCL	8		19.1	Protein MMS22-like	B1AUR6-1
KGLRFIQL	8		19.1	Calpain-7	Q9R1S8
SSIVFGRF	8		19.1	Leukocyte immunoglobulin-like receptor subfamily B member 4	Q64281; Q64281-2
TSYPFDTV	8		19.1	ADP/ATP translocase 2	P51881
RIYGKFLGL	9		19.2	orm 1 of Serine/threonine-protein phosphatase 2A 56 kDa regulatory subunit gamma isof	Q60996-2; Q60996-4; Q60996-1; Q6PD03; Q61151; Q60996-3
SALPFVKL	8		19.3	Protein TANC1	Q0VGY8-2; Q0VGY8
FNLVFERL	8		19.3	Ankyrin repeat and SOCS box protein 6	Q91ZU1
ILIVYENL	8		19.3	Eukaryotic peptide chain release factor subunit 1	Q8BWY3
SGLQYNSL	8		19.3	Tumor necrosis factor alpha-induced protein 3	Q60769
IGPRYSSV	8		19.4	HEAT repeat-containing protein 5A	Q5PRF0
VGITYQHI	8		19.6	Eukaryotic translation initiation factor 3 subunit K	Q9DBZ5-2; Q9DBZ5
QSLAFHTL	8		19.7	Elongator complex protein 2	Q91WG4-2; Q91WG4
INFDFNTI	8		19.8	BTB/POZ domain-containing protein KCTD20	Q8CDD8-2; Q8CDD8
FVYIFQEV	8		19.9	Plastin-3	Q99K51
VHLKFSAKL	9		20	Growth arrest-specific protein 7	Q60780-2; Q60780-1
SIVQFYYM	8		20.1	Metastasis-associated protein MTA2	Q9R190
AVLSFSTRL	9		20.1	Dolichyl-diphosphooligosaccharide--protein glycosyltransferase subunit STT3A	P46978
IGLAYVNHL	9		20.1	Vacuolar protein sorting-associated protein 41 homolog	Q5KU39
TNLIYNLL	8		20.1	Leucyl-cystinyl aminopeptidase	Q8C129
KGFEFTLM	8		20.2	septin-2	P42208
RGYSYERV	8		20.4	Chromodomain-helicase-DNA-binding protein 1-like	Q9CXF7-1
VTLVFEHI	8		20.6	Cyclin-dependent kinase 4	P30285
VNRKYEYL	8		20.6	Protein archease	Q505B7-2; Q505B7
STIEFKNM	8	1xOxidation [M8]	20.7	Regulation of nuclear pre-mRNA domain-containing protein 2	Q6NXI6; Q6NXI6-2
SNVVFKLL	8		20.7	Prefoldin subunit 6	Q03958
ASPEFTKL	8		20.9	protein FAM98A	Q3TJZ6
VQYTFDLQL	9		21	G/T mismatch-specific thymine DNA glycosylase	P56581-2; P56581
SSMAFKQM	8		21.2	Transgelin-2	Q9WVA4
KTVCFQNL	8		21.2	Cytoplasmic FMR1-interacting protein 1 OS=Mus musculus OX=10090	Q7TMB8
INYDYVHEL	9		21.2	Zinc finger CCCH domain-containing protein 13	E9Q784
SIINFIERL	9		21.3	Putative homeodomain transcription factor 1	Q9QZ09
SSFDHTTL	8		21.4	Cis-aconitate decarboxylase	P54987
RSYDFEFM	8		21.5	Vacuolar protein sorting-associated protein 26A	P40336; P40336-2
ICFKFDHL	8		21.6	F-actin-capping protein subunit alpha-2	P47754
SAPEYVFL	8		21.6	Protein strawberry notch homolog 2	Q7TNB8; Q7TNB8-2
IDYSFPSL	8		21.7	MKI67 FHA domain-interacting nucleolar phosphoprotein	Q91VE6; Q91VE6-2
TALRFLEL	8		21.7	Phosphoinositide 3-kinase regulatory subunit 4	Q8VD65
VAVTFSERL	9		21.8	Cystine/glutamate transporter	Q9WTR6
AGFAFLTGV	9		21.9	Bax inhibitor 1	Q9D2C7

ASITFEHM	8		22	Eukaryotic translation initiation factor 3 subunit H	Q91WK2
RNFNYHIL	8		22.1	Cleavage and polyadenylation specificity factor subunit 3	Q9QXK7
NTYIYSGM	8		22.1	NEDD8-activating enzyme E1 regulatory subunit	Q8VBW6
FTFKYHHV	8		22.2	Membrane-associated progesterone receptor component 1	O55022
ATLAYTKL	8		22.2	Dedicator of cytokinesis protein 1	Q8BUR4-1; Q8BUR4-2
SMYQPLNL	8		22.2	Globoside alpha-1,3-N-acetylgalactosaminyltransferase 1	Q8VI38
TALSPYASL	9		22.2	RNA-binding protein 5	Q91YE7-2; Q91YE7
VIYTHPEV	8		22.4	Dihydrolipoyl dehydrogenase, mitochondrial	O08749
AGYMYTQLM	9		22.6	E3 ubiquitin-protein ligase UBR4	A2AN08-3; A2AN08-5; A2AN08
KCYYYHARV	9		22.8	26S proteasome non-ATPase regulatory subunit 3	P14685
RGYDFPAV	8		22.8	EH domain-containing protein 2	Q8BH64
VNVYYTTM	8	1xOxidation [M8]	22.9	NTF2-related export protein 1	Q9QZV9
SSPVFKAM	8	1xOxidation [M8]	22.9	Kelch-like ECH-associated protein 1	Q9Z2X8
RSYNMPSL	8		23	Melanoma inhibitory activity protein 2	Q91ZV0; Q91ZV0-3
VVARFLSL	8		23.3	Mini-chromosome maintenance complex-binding protein	Q8R3C0
SQLEFRQNL	9		23.5	SEC14 domain and spectrin repeat-containing protein 1	Q80UK0
TTMNFPKL	8		23.6	Regulator of microtubule dynamics protein 2	Q8BSE0
ETYKYFSL	8		23.6	Transmembrane 9 superfamily member 3	Q9ET30
KTLRYNFL	8		23.8	Nucleoporin NUP188 homolog	Q6ZQH8
SNIHYHTL	8		23.9	Chromatin assembly factor 1 subunit B	Q9D0N7
KNYGFVHI	8		23.9	RNA-binding protein 4B	Q8VE92; Q8C7Q4-2; Q8C7Q4
VAPDRFPTL	9		24.2	Centrosomal protein POC5	Q9DBS8; Q9DBS8-2
RAFLFNKV	8		24.2	Protein yippee-like 5	P62700
VAPSFGTLL	9		24.4	Nucleoporin NUP188 homolog	Q6ZQH8
RNNRFPNL	8		24.8	pumilio homolog 2	Q80U58-3; Q80U58-2; Q80U58-1
INSRFAKV	8		24.8	Exosome complex component CSL4	Q9DAA6
ISPCFQERL	9		24.8	FAST kinase domain-containing protein 1, mitochondrial	Q6DI86-2; Q6DI86-3; Q6DI86
SVVGFYSL	8		24.8	Limb region 1 protein	Q9JIT0; Q9JIT0-3
TIYERFVLV	9		24.9	Crooked neck-like protein 1	P63154
KNYSFPLNNL	10		25	Deubiquitinating protein VCIP135	Q8CDG3; Q8CDG3-2
RTPVFSFL	8		25	SIN3-HDAC complex-associated factor	Q8C8M1
KTYQVAHM	8		25.1	Multidrug resistance-associated protein 1	O35379
LSLKYESL	8		25.2	H(+)/Cl(-) exchange transporter 7	O70496
VNLREYPSL	9		25.2	Sp110 nuclear body protein	Q8BVK9
AQYNFILV	8		25.3	Threonine--tRNA ligase, cytoplasmic	Q9D0R2; Q8BLY2
ANLKYLSL	8		25.3	UDP-N-acetylglucosamine transporter	Q8R1T4
IIYNPKNL	8		25.5	Splicing factor 3A subunit 3	Q9D554
RGPAFTNL	8		25.5	Mitochondrial mRNA pseudouridine synthase Trub2	Q91WG3
AQFKFTVL	8		25.6	Proliferation-associated protein 2G4	P50580; P50580-2
AQLIYRHL	8		25.6	Ras GTPase-activating protein 4	Q6PFQ7-2; Q6PFQ7
SVAHFINL	8		25.8	PR domain zinc finger protein 15	E9Q8T2; E9Q8T2-2
STLRFLCKL	9		25.9	Coatomer subunit beta	Q9JIF7
VNVDYSKL	8		26	Cytochrome c oxidase subunit NDUFA4	Q62425
KSYSFDEV	8		26.2	nicotinamide phosphoribosyltransferase	Q99KQ4
CGYEFTSKL	9		26.2	Cullin-2	Q9D4H8
QSVAFTKL	8		26.2	Kinesin-like protein KIF20B	Q80WE4; Q80WE4-4; Q80WE4-3
SAYLYKQGF	9		26.3	Transmembrane protein 41A	Q9D8U2
TTYVHKGL	8		26.3	Mitochondrial import receptor subunit TOM70	Q9CZW5
ATFDFSQYM	9		26.5	DNA mismatch repair protein Msh2	P43247
TGPKYIHL	8		26.7	Tyrosine-protein kinase Mer	Q60805
KNFKFTMDL	9		26.7	Toll-like receptor 9	Q9EQU3
LVYQFKEM	8		27	ETS-related transcription factor Elf-4	Q9Z2U4; Q60775
SALDFVKL	8		27	NLR family CARD domain-containing protein 4	Q3UP24
KAYIHTRM	8		27.1	Actin-related protein 2/3 complex subunit 2	Q9CVB6

VDYVFTAM	8		27.1	TBC1 domain family member 5	Q80XQ2
KTTFFRNL	8		27.2	Isoform 2 of Ras association domain-containing protein 5	Q5EBH1-2
KGYAYTFI	8		27.4	probable ATP-dependent RNA helicase DDX46	Q569Z5-1; Q569Z5-2
STYDYGRQL	9		27.4	SEC14 domain and spectrin repeat-containing protein 1	Q80UK0
SCFSFRKL	8		27.6	natural resistance-associated macrophage protein 2	P49282
ATYSYKEAL	9		27.7	cell division cycle protein 16 homolog	Q8R349
VIMKLFPQL	9		27.8	all-trans-retinol 13,14-reductase	Q64FW2
QNPRFSKL	8		28	HAUS augmin-like complex subunit 4	Q8BFT2
KTYTFDMV	8		28.3	Kinesin-like protein KIF11	Q6P9P6
RSPEYLSL	8		28.5	Cullin-3	Q9JLV5
TTYKYEMI	8		28.6	eukaryotic translation initiation factor 3 subunit L	Q8QZY1
SQHNFNNL	8		28.6	Zinc finger CCHC domain-containing protein 2	Q69ZB8-2; Q69ZB8-1
LSYSYQSRF	9		28.8	Splicing factor 3B subunit 3	Q921M3-2; Q921M3-1
SVIKYELL	8		28.8	Receptor-type tyrosine-protein phosphatase S	B0V2N1-2; B0V2N1; B0V2N1-3; B0V2N1-6; B0V2N1-4
RIILFDRL	8		29	TBC domain-containing protein kinase-like protein	Q8BM85-2; Q8BM85
VIYPMAVV	8		29.1	mRNA export factor	Q8C570
YVLHFTAL	8		29.1	Ribosome production factor 2 homolog	Q9JJ80
SIFIFDEM	8		29.1	Prosalusin	P0C7W3; Q8R1J9
FGYEYITV	8		29.3	natural resistance-associated macrophage protein 2	P49282-4; P49282-3; P49282-2; P49282
VAFDYQSKM	9		29.3	Protein LSM12 homolog	Q9D0R8
KSYLMNKL	8		29.4	Guanylate-binding protein 5	Q8CFB4; Q01514; Q8CFB4-2; Q9Z0E6
KNYDFAQVL	9		29.5	PAB-dependent poly(A)-specific ribonuclease subunit PAN2	Q8BGF7-2; Q8BGF7; Q8BGF7-3
CAMIFRQL	8		29.5	Anaphase-promoting complex subunit 5	Q8BTZ4; Q8BTZ4-2
IAPIYKKL	8		29.5	Methylcytosine dioxygenase TET2	Q4JK59
LSYTRFSLA	9		29.5	Transferrin receptor protein 1	Q62351
SQYEFENYM	9		29.6	Serine/threonine-protein kinase 40	Q7TNL3-1; Q7TNL3-2
VSPRLTFL	8		30	Antigen peptide transporter 2	P36371
IAYKFGKTV	9		30.1	Store-operated calcium entry-associated regulatory factor	Q8R3Q0
VAPGYPLL	8		30.3	Embigin	P21995
FVYTYKLV	8		30.4	Splicing factor 3B subunit 3	Q921M3-2; Q921M3
VILSFENHV	9		30.5	1-phosphatidylinositol 4,5-bisphosphate phosphodiesterase beta-3	P51432; A3KGF7; A3KGF7-3; A3KGF7-4; A3KGF7-2
TSIAFKNI	8		30.6	MAP/microtubule affinity-regulating kinase 3	Q03141; Q03141-2; Q8VHJ5; Q03141-3
GVLRFVNL	8		30.8	Putative Dol-P-Glc:Glc(2)Man(9)GlcNAc(2)-PP-Dol alpha-1,2-glucosyltransferase	Q3UGP8
AVPIFVAL	8		31	Cystine/glutamate transporter	Q9WTR6
VITNFSARI	9		31.1	Vacuolar protein sorting-associated protein 51 homolog	Q3UVL4-1; Q3UVL4-2
VNIEFKDL	8		31.2	ATP-binding cassette sub-family G member 1	Q64343
SIANFTNV	8		31.2	ATP-citrate synthase	Q91V92
IMYDKHIQM	9		31.4	Interferon-induced very large GTPase 1	Q80SU7
TNYRFKNLF	9		31.5	G1/S-specific cyclin-E2	Q9Z238
RALEHFPML	9	1xOxidation [M8]	31.6	Glycerol-3-phosphate dehydrogenase, mitochondrial	Q64521
KVLVFSQM	8		31.7	Lymphocyte-specific helicase	Q60848-2; Q60848-1
AMYIFLHTV	9		31.7	ORM1-like protein 2	Q9CQZ0
GTYDYTQL	8		31.7	Dol-P-Man:Man(5)GlcNAc(2)-PP-Dol alpha-1,3-mannosyltransferase	Q8K2A8
RVLLFSQM	8		31.8	SNF-related matrix-associated actin-dependent regulator of chromatin subfamily A mem	Q9CXF7-2; E9PZM4; Q9CXF7; Q6PGB8; P40201; A2AJK6-3; Q6PDQ2; A2AJK6; Q91ZW3; A2A8L1; Q6PGB8-2
TITSFPRL	8		32.3	Glutamate--cysteine ligase catalytic subunit	P97494
LQYVYNLV	8		32.3	DNA replication licensing factor MCM4	P49717
RQYVHPRL	8		32.4	DNA helicase MCM8	Q9CWV1-1; Q9CWV1-2
KAFDYPSRL	9		32.4	Zinc finger protein 728	Q6P5C7
AICVFPRL	8		32.8	Poly(A)-specific ribonuclease PARN	Q8VDG3
KVLRFLNV	8		32.8	TBC1 domain family member 31	Q6NXY1
VTYSKPRL	8		32.9	ATP synthase subunit g, mitochondrial	Q9CPQ8
SIYTPLANI	9		32.9	E3 ubiquitin-protein ligase TTC3	O88196-2; O88196-4; O88196-6; O88196; O88196-3; O88196-5
RGYDFCQV	8		33	EH domain-containing protein 4	Q9EQP2

STYSVAKM	8		33.1	Lysosomal alpha-mannosidase	O09159
INYVIKQL	8		33.2	Dihydroxyacetone phosphate acyltransferase	P98192
SGPTYIKL	8		33.3	Uncharacterized aarF domain-containing protein kinase 2	Q6NSR3
SNYNFEKPF	9		33.6	GTP-binding nuclear protein Ran, testis-specific isoform	Q61820; P62827
HIYQFEYM	8		33.8	striatin-interacting protein 1	Q8C079-4; Q8C079
SNYEVFQL	8		33.8	DNA-directed RNA polymerase III subunit RPC9	O35427
TILEFSQNM	9	1xOxidation [M9]	34.2	Exportin-1	Q6P5F9
VSYKNPSL	8		34.3	FACT complex subunit SPT16	Q920B9
FAPIYADL	8		34.3	Thioredoxin-related transmembrane protein 2	Q9D710
VMYRVIQV	8		34.4	Upstream stimulatory factor 1	Q61069
QNYEMPNL	8	1xOxidation [M5]	34.5	Short transmembrane mitochondrial protein 1	P0DP99
VIEEFRHL	8		34.6	Signal transducer and transcription activator 6	P52633
NKMPLSVFPYYF	14		34.6	Protein mono-ADP-ribosyltransferase PARP14	Q2EMV9
VIAGFNRL	8		34.7	Prefoldin subunit 2	O70591
RVYEFLDKL	9		34.9	26S proteasome non-ATPase regulatory subunit 3	P14685
NIYRFIMV	8		35	Protein cornichon homolog 4	Q9CX13
QVYTFTERM	9		35.1	-acetyl-neuraminyl-2,3-beta-galactosyl-1,3-N-acetyl-galactosaminide alpha-2,6-sialyltran	Q9R2B6-2; Q9R2B6; Q9R2B6-3
NVWRFPYL	8		35.2	Sodium- and chloride-dependent GABA transporter 2	P31649; Q761V0-1; Q9D687; Q9JMA9; O88575; Q8VBW1-2; P31651; Q8BG16; P31648; Q761V0-2; P28571-1; Q8VBW1-3; P31650; Q61327; O55192; Q8VDB9; O35316; P28571; Q8VBW1-1; P28571-2
KNIRFPLM	8		35.4	kelch-like protein 9	Q80TF4-2; Q80TF4-5; Q80TF4-4; Q80TF4; Q80TF4-3; Q6ZPT1
RILKFLQL	8		35.5	5'-AMP-activated protein kinase subunit gamma-2	Q91WG5-2; Q91WG5
INYSFPAKGKL	11		35.6	ATP-dependent RNA helicase DDX54	Q8K4L0
VFSSFSAL	8		35.6	Solute carrier family 49 member A3	Q8CE47
KVLVFNFL	8		36	Anoctamin-10	Q8BH79; Q8BH79-3; Q8BH79-2; Q8BH79-4
VITKFDHL	8		36.1	C2 domain-containing protein 3	Q52KB6-2; Q52KB6-3; Q52KB6
RQIDYYCRL	9		36.2	adenylate cyclase type 7	P51829
QNYLFGCEL	9		36.3	Nucleophosmin	Q61937
AQYSFDKL	8		36.3	HEAT repeat-containing protein 5B	Q8C547; Q8C547-2
SQQLYRHL	8		36.4	AT-rich interactive domain-containing protein 5B	Q8BM75; Q8BM75-2; Q6ZQ18-2; Q6ZQ18
AIIAFKTL	8		36.5	Rho guanine nucleotide exchange factor 6	Q8K4I3
VAASFKGL	8		36.5	RRP12-like protein	Q6P5B0
VQYEMRTL	8		36.7	28S ribosomal protein S10, mitochondrial	Q80ZK0
KNYQMASV	8		36.7	Myoferlin	Q69ZN7-4; Q69ZN7
VNYDYSTLIL	10		36.8	Zinc finger BED domain-containing protein 6	D2EAC2; D2EAC2-2
QVVQFNRL	8		36.9	Ragulator complex protein LAMTOR3	O88653
RNNRYPNL	8		37	Pumilio homolog 1	Q80U78-2; Q80U78-1; Q80U78-3
VNYEPLGL	8		37	Death-associated protein kinase 1	Q80YE7; Q8VDF3; Q8VDF3-2; O54784; Q80YE7-2
NTYKYAKI	8		37.2	Ankyrin repeat and BTB/POZ domain-containing protein 2	Q7TQI7
RVLRFSLV	8		37.2	Exostosin-like 2	Q9ES89
SALRFEGQM	9		37.2	Adenylyltransferase and sulfurtransferase MOCS3	A2BDX3
RGYDFCQVL	9		37.4	EH domain-containing protein 4	Q9EQP2
ATRSFPQL	8		37.4	Annexin A7	Q07076
RIYRMIKL	8		37.5	Heat shock protein HSP 90-beta	P07901; P11499
SSPVFKAMF	9		37.9	Kelch-like ECH-associated protein 1	Q9Z2X8
INLNYKDL	8		38.1	Translocon-associated protein subunit alpha	Q9CY50
YGLAYRSL	8		38.5	DENN domain-containing protein 4C	A6H8H2; A6H8H2-2
VCYGSFYNL	9		38.5	GPI transamidase component PIG-T	Q8BXQ2
TCLCFARL	8		38.7	E3 ubiquitin-protein ligase TRIP12	G5E870
KNWQFVENL	9		38.7	Ribonuclease H2 subunit A	Q9CWY8
KSFEWLSQM	9		38.9	Cytoplasmic dynein 1 heavy chain 1	Q9JHU4
ATYIFLQTF	9		39	Transmembrane protein 41B	Q8K1A5; Q8K1A5-2; Q8K1A5-3
VNMEKYPDL	9		39.1	Component of Sp100-rs	Q99388; O35892-2; O35892-1
SGLKYVAV	8		39.3	Flavin reductase (NADPH)	Q923D2

VAFDRHLYV	9		39.3	Leucine-zipper-like transcriptional regulator 1	Q9CQ33-2; Q9CQ33
RNPTFILL	8		39.4	solute carrier organic anion transporter family member 4A1	Q8K078-2; Q8K078
SDYVYPSL	8		39.5	lysine-specific demethylase phf2	Q9WTU0
NGYSFINI	8		39.5	Erbin	Q80TH2-2; Q80TH2; Q80TH2-1
ALVRFVNL	8		39.5	Ribosomal biogenesis protein LAS1L	A2BE28-2; A2BE28
VVYIYKEHF	9		39.8	Small subunit processome component 20 homolog	Q5XG71
VDYEYSEL	8		40.2	N-alpha-acetyltransferase 15, NatA auxiliary subunit	Q80UM3
LGPYPYATL	9		40.2	Protein AAR2 homolog	Q9D2V5
INQRFEEL	8		40.2	Signal transducer and activator of transcription 5A	P42230
KNYVLQTL	8		40.5	Importin subunit beta-1	P70168
INAEFVTQL	9		40.7	Intron-binding protein aquarius	Q8CFQ3
IGYGYLHRI	9		40.8	Golgi reassembly-stacking protein 2	Q99JX3; Q99JX3-2; Q91X51
TSPEYQKL	8		40.9	Alsin	Q920R0
EIFRFYKL	8		41.2	Coronin-2A	Q8C0P5
KIIDFGFARL	10		41.3	Ribosomal protein S6 kinase alpha-4	Q9Z2B9
RQVRYSYL	8		41.4	Zinc finger protein RFP	Q62158
RVLIFSQM	8	1xOxidation [M8]	42.3	Chromodomain-helicase-DNA-binding protein 2	Q9CXF7-2; E9PZM4; Q9CXF7-1; Q6PGB8; P40201; A2AJK6-3; Q6PDQ2; A2AJK6-1; Q91ZW3; A2A8L1; Q6PGB8-2
RVLIFSQM	8		42.3	Chromodomain-helicase-DNA-binding protein 2	Q9CXF7-2; E9PZM4; Q9CXF7-1; Q6PGB8; P40201; A2AJK6-3; Q6PDQ2; A2AJK6-1; Q91ZW3; A2A8L1; Q6PGB8-2
SVIVFGSI	8		42.4	E3 ubiquitin-protein ligase MARCH6	Q6ZQ89; Q6ZQ89-2; Q6ZQ89-3
TGLRYNMRL	9		42.4	UDP-glucose:glycoprotein glucosyltransferase 1	Q6P5E4
QPYLWARL	8		42.7	Uncharacterized protein C12orf29 homolog	Q8BHN7; Q8BHN7-2
ETPVYANL	8		42.8	Transcription factor jun-D	P15066
RTYSVPRL	8		43	Palmitoyltransferase ZDHHC16	Q9ESG8
SIFKAADL	8		43.2	Branched-chain-amino-acid aminotransferase, mitochondrial	O35855
VNFEFPEF	8		43.3	40S ribosomal protein S7	P62082
VGPKFRGV	8		43.4	Protein AAR2 homolog	Q9D2V5
RTYTYEKLL	9		43.4	Catenin beta-1	Q02248
KSPQFRNI	8		43.9	ATP-dependent RNA helicase DHX29	Q6PGC1
RTFSWASV	8		44.1	Ras GTPase-activating protein-binding protein 1	P97855
QSPEYENL	8		44.1	Nesprin-1	Q6ZWR6-4; Q6ZWR6
ENFRFLSL	8		44.2	Calcium homeostasis modulator protein 2	Q8VEC4
TSPEFTSV	8		44.3	Serine/threonine-protein kinase SMG1	Q8BKX6-3; Q8BKX6; Q8BKX6-2
SRIVFRHL	8		44.5	28S ribosomal protein S14, mitochondrial	Q9CR88
RAYSFKVVL	9		44.5	Ras-related protein Rab-21	P35282
INQIYEARV	9		44.6	Arf-GAP with coiled-coil, ANK repeat and PH domain-containing protein 1	Q8K2H4
SNPEFSSV	8		44.6	Talin-1	P26039
SNLKYILV	8		44.9	MAX gene-associated protein	A2AWL7-2; A2AWL7-3; A2AWL7-4; A2AWL7
IWIRVASL	8		44.9	Kelch-like protein 24	Q8BRG6
VRIIFSTL	8		45.1	Solute carrier family 22 member 4	Q9Z306
RVYEFTRA	8		45.2	Globoside alpha-1,3-N-acetylgalactosaminyltransferase 1	Q8VI38
VAPEYQLV	8		45.4	Sn1-specific diacylglycerol lipase beta	Q91WC9
HQYIFSHI	8		45.7	Endoribonuclease Dicer	Q8R418; Q8R418-2
SNFVHRDL	8		45.9	Tyrosine-protein kinase SYK	P48025
STVEFTCL	8		46	Rho guanine nucleotide exchange factor 6	Q8K4I3
RVFQFLVL	8		46	39S ribosomal protein L37, mitochondrial	Q921S7
TILNFNDL	8		46.1	L-aminoadipate-semialdehyde dehydrogenase-phosphopantetheinyl transferase	Q9CQF6; Q9CQF6-2
VGINYREV	8		46.2	DNA topoisomerase 2-alpha	Q01320
AIYEFIHNF	9		46.3	AP-4 complex subunit sigma-1	Q9WVL1
QALIYAEM	8		46.3	Calpain-7	Q9R1S8
YAYRHPLV	8		46.6	Gamma-adducin	Q9QYB5-2; Q9QYB5
RSTVFGTL	8		46.8	Stromal interaction molecule 2	P83093
SLILFSTRL	9		47	Baculoviral IAP repeat-containing protein 1b	Q9QUK4

RGLEYLYL	8	47.3	Cytoplasmic dynein 1 light intermediate chain 1	Q8R1Q8; Q6PDL0
VINSFVHV	8	47.4	Cullin-2	Q9D4H8; Q9D4H8-2
SILRFITI	8	47.5	Myeloid differentiation primary response protein MyD88	P22366; P22366-2
KVYKYVFI	8	47.6	Monocarboxylate transporter 4	P57787
SIAAFIQRL	9	47.7	Large proline-rich protein BAG6	Q9Z1R2
SILALTHL	8	47.9	RRP12-like protein	Q6P5B0
SIAQFKYL	8	48	Phospholipid-transporting ATPase IA	P70704-3; P70704-2; P70704
IPYDFNRV	8	48.1	Receptor-type tyrosine-protein phosphatase epsilon	P49446-3; P49446-2; P49446
VWYWRRITM	9	48.3	Protein wntless homolog	Q6DID7; Q6DID7-2
SIYDAFPKV	9	48.5	Microtubule-actin cross-linking factor 1	Q9QXZ0-3; Q91ZU6-8; Q91ZU6-3; Q91ZU6-4; Q91ZU6-2; Q9QXZ0-2; Q9QXZ0-4; Q91ZU6-6; Q9QXZ0; Q91ZU6
IQFSFKEKL	9	48.8	Protein dopey-1	Q8BL99; Q8BL99-6; Q8BL99-5
QNHVFPLL	8	48.9	Importin-8	Q7TMY7-2; Q7TMY7
RNQVYTQL	8	49	Melanoma inhibitory activity protein 2	Q91ZV0; Q8R311
VRVFFSGL	8	49	RNA-binding protein 12B-A	Q80YR9; Q66JV4
NAIKYVNL	8	49.3	Dual specificity protein kinase TTK	P35761; P35761-2
KQFSYTHI	8	49.5	Electron transfer flavoprotein subunit alpha, mitochondrial	Q99LC5
VNRVFDKL	8	49.7	Proteasome subunit beta type-9	O35522; P28076
NTYMHLHL	8	49.7	Adhesion G protein-coupled receptor E1	Q61549
RIYQFTAA	8	49.9	src kinase-associated phosphoprotein 2	Q3UND0-2; Q3UND0-1
TAPEYVFL	8	49.9	Protein strawberry notch homolog 1	Q689Z5; Q689Z5-2
VNFGFSKF	8	50.9	Double-strand break repair protein MRE11	Q61216; Q61216-2
KVLRFPNRL	9	50.9	Protein MTO1 homolog, mitochondrial	Q923Z3
VNYDFGHMHVP	11	51.2	E3 ubiquitin-protein ligase RNF216	P58283-2; P58283
QPYEFTTL	8	51.8	Islet cell autoantigen 1	P97411; P97411-2
IFYYVQKL	8	52.7	E3 ubiquitin-protein ligase hectd1	Q69ZR2
TVPKFVKL	8	53.3	Rho GTPase-activating protein 12	Q8C0D4
SAPWYLNRV	9	53.6	Beta-hexosaminidase subunit alpha	P29416
SIYAREALI	9	53.6	Structural maintenance of chromosomes protein 1A	Q9CU62
VAFIFNQKF	9	53.7	[F-actin]-monooxygenase MICAL2	Q8CJ19-3; Q8BML1-3; Q8CJ19; Q8BML1; Q8BML1-2; Q8CJ19-2
VGITYDHV	8	54.1	SPRY domain-containing protein 7	Q3TFQ1
NSPVFVGL	8	54.3	Protocadherin Fat 3	Q8BNA6
KNLDWFPRM	9	54.3	Inositol 1,4,5-trisphosphate receptor type 2	Q9Z329; Q9Z329-3; P70227; Q9Z329-2
SAISLRNL	8	54.3	SCL-interrupting locus protein homolog	Q60988
VQYLYRVF	8	54.4	probable ATP-dependent RNA helicase DDX10	Q80Y44
SCFEYQKL	8	54.6	dual specificity tyrosine-phosphorylation-regulated kinase 3	Q922Y0
SSDVFPLL	8	54.6	Transcription factor E2F5	Q61502
VNCPFISTL	9	55.2	Transcription regulator protein BACH1	P97302
NIFMFSKV	8	55.6	Thioredoxin-related transmembrane protein 2	Q9D710
VNFIKENLL	9	55.6	Transcriptional regulator ATRX	Q61687
FSPVYRCL	8	56.7	Exocyst complex component 6	Q8R313; A6H5Z3
SAYEVIKL	8	56.8	L-lactate dehydrogenase B chain	P16125; P06151
CVYEFRDKL	9	57.3	Heat shock protein 105 kDa	Q61699
SNYLHRVV	8	57.5	F-box only protein 22	Q78JE5
SVYGFTGA	8	57.6	DNA polymerase delta catalytic subunit	P52431
RSPKYLEL	8	58	Myb-binding protein 1A	Q7TPV4
NQYKFILL	8	58.2	Inositol hexakisphosphate kinase 2	Q80V72
QQYVFINQM	9	58.4	volume-regulated anion channel subunit LRRC8D	Q8BGR2
TIILFTKV	8	58.7	Importin-11	Q8K2V6-2; Q8K2V6-1
LVAIFTHL	8	58.8	Cytoplasmic dynein 1 heavy chain 1	Q9JHU4
SVVTFSVHM	9	59.2	Multidrug resistance-associated protein 5	Q9R1X5
RIFEFQVM	8	59.4	Programmed cell death protein 2	P46718
PTYIYRLL	8	59.4	Isoform 2 of Ubiquitin carboxyl-terminal hydrolase 10	P52479; P52479-2
QQYRFSVI	8	59.4	Inverted formin-2	Q0GNC1; Q0GNC1-3

SLVKYVPL	8	60.2	Sodium- and chloride-dependent taurine transporter	O35316
RAPSYRTL	8	60.3	Suppression of tumorigenicity 5 protein	Q924W7-2; Q924W7; Q924W7-3
RIVELFRNL	9	60.4	Signal transducer and activator of transcription 3	P42227-3; P42227; P42227-2
LVHPFRAL	8	60.7	Nucleolar protein 14	Q8R3N1
INFDFHQF	8	60.9	Synaptojanin-2	Q9D2G5-4; Q9D2G5-6; Q9D2G5-5; Q9D2G5-1; Q9D2G5-2; Q9D2G5-3
TQYIFTEKL	9	61.1	H/ACA ribonucleoprotein complex non-core subunit NAF1	Q3UMQ8
VGPRFELKL	9	61.7	U3 small nucleolar ribonucleoprotein protein IMP4	Q8VHZ7
VWLEAARL	8	61.9	Pre-mRNA-processing factor 6	Q91YR7; Q91YR7-2
RVPTFAFV	8	62.5	Up-regulator of cell proliferation	Q5NCI0-2; Q5NCI0
SVNIFRTL	8	62.6	Serine/threonine-protein phosphatase 2A 56 kDa regulatory subunit beta isoform	Q6PD28
SINSRFAKV	9	63.1	Exosome complex component CSL4	Q9DAA6
KIFEFKETL	9	63.4	calcium/calmodulin-dependent protein kinase type 1D	Q8BW96-2; Q8BW96
SVILMQHL	8	63.8	Transmembrane protein 39B	Q810L4
TNLIYQQV	8	63.8	Unconventional myosin-VIIa	P97479-1; P97479-2
IGYEHEVL	8	64	Uncharacterized protein C15orf41 homolog	Q3U4G0-2; Q3U4G0
LAPVFQRV	8	64.3	Conserved oligomeric Golgi complex subunit 8	Q9JJA2
SNYERLESL	9	64.4	Perilipin-2	P43883
KAYSFKEQI	9	64.5	eIF-2-alpha kinase activator GCN1	E9PVA8
AVCTFIHL	8	64.7	3-beta-hydroxysteroid-delta(8),delta(7)-isomerase	P70245
KGVAYVYL	8	65	Tudor domain-containing protein 7	Q8K1H1
RAPAFHQL	8	65	Zinc finger SWIM domain-containing protein 8	Q3UHH1-2; Q3UHH1; Q3UHH1-3
KVYMFKCV	8	65.2	Lysosomal-associated transmembrane protein 5 OS=Mus musculus OX=10090	Q61168
KVVEFSEL	8	65.2	nucleolar MIF4G domain-containing protein 1	Q3UFM5
LIYTFTTTF	9	65.6	Tripartite motif-containing protein 26	Q99PN3
QQYSFINQM	9	65.7	Volume-regulated anion channel subunit LRRC8C	Q8R502
VNFKHEVSV	9	65.8	RNA-binding protein 7	Q9CQT2
KILTFDQL	8	66.5	60S ribosomal protein L18	P35980
VNFEKFWEL	9	66.6	Ras-GEF domain-containing family member 1B	Q8JZL7-2; Q8JZL7
RNPTFMCL	8	66.8	AP-2 complex subunit alpha-1	P17426-2; P17426
RNLQFVGV	8	66.8	KN motif and ankyrin repeat domain-containing protein 2	Q8BX02; Q8BX02-2
VNVCYKEL	8	66.9	THO complex subunit 5 homolog	Q8BKT7
SAPTFINF	8	67.5	Magnesium transporter protein 1	Q9CQY5-2; Q9CQY5-3; Q9CQY5
ATQQFQQL	8	67.6	Retrovirus-related Env polyprotein from Fv-4 locus	P11370
ASCIYPLL	8	67.6	RNA N6-adenosine-methyltransferase METTL16	Q9CQG2; Q9CQG2-2
INVAFSCV	8	67.8	Fermitin family homolog 3	Q8K1B8
SGVGFGGL	8	67.8	Isoform 2 of Myelin expression factor 2	Q8C854-3
AALIYTSV	8	67.9	Cytoplasmic dynein 1 light intermediate chain 1	Q8R1Q8; Q6PDL0
TQFLYPKV	8	68.3	Transcription factor jun-D	P15066
VISDFITRL	9	68.6	Sterol 26-hydroxylase, mitochondrial	Q9DBG1
SCYAFLQV	8	69	CD302 antigen	Q9DCG2
VNVRFSTIV	9	69.5	Dual specificity protein phosphatase 1	P28563
TIVVFNGM	8	70.2	Poly [ADP-ribose] polymerase 9	Q8CAS9-2; Q8CAS9
SRIVFIPL	8	70.4	Equilibrative nucleoside transporter 1	Q9JIM1; Q9JIM1-2
AVIKFLEL	8	70.5	DNA mismatch repair protein Msh2	P43247
VQRSFSQV	8	70.6	Sterol regulatory element-binding protein 2	Q3U1N2-1
IITGFRNV	8	70.6	Mitotic checkpoint serine/threonine-protein kinase BUB1 beta	Q9Z1S0
VMIQHVENL	9	70.8	Lymphoid-restricted membrane protein	Q60664
TSLKYLEM	8	70.9	Biogenesis of lysosome-related organelles complex 1 subunit 2	Q9CWG9
LNFEFQIV	8	71.5	E3 SUMO-protein ligase RanBP2	Q9ERU9
KVVKFSYM	8	71.7	Speckle-type POZ protein	Q6ZWS8; Q2M2N2
SQYGWSGNM	9	71.8	Endoplasmin	P08113
TAPHYQLL	8	72	WD repeat-containing protein 20	Q9D5R2
VAHTFVIGV	9	72.7	proliferation-associated protein 2G4	P50580; P50580-2

SIIRLQSL	8		73.3	Unconventional myosin-IXb	Q9QY06-3; Q9QY06-2; Q9QY06
KTFRKSFNL	9		74	Suppressor of cytokine signaling 6	Q9JLY0
VQQYYRKL	8		74.1	Phosphatidylinositol 3,4,5-trisphosphate-dependent Rac exchanger 1 protein	Q69ZK0; Q69ZK0-2
IMYKLEVL	8		74.1	Short transient receptor potential channel 4-associated protein	Q9JLV2; Q9JLV2-2
SLIEFFNKM	9		74.7	Retinoblastoma-like protein 2	Q64700
RNYIHRDL	8		74.9	Tyrosine-protein kinase HCK	P08103-1; P08103-2
KVALFNRL	8		75.2	Recombining binding protein suppressor of hairless	P31266-2; P31266
VRYVLPRL	8		75.6	Son of sevenless homolog 2	Q02384
SVINVSNL	8		75.7	S-phase kinase-associated protein 2	Q9Z0Z3; Q9Z0Z3-2
QIYDIFQKL	9		76.4	Eukaryotic initiation factor 4A-I	P60843
RVMEYINRL	9		78.4	Clathrin heavy chain 1	Q68FD5
RGPTYVNM	8		78.8	Arf-GAP domain and FG repeat-containing protein 1	Q8K2K6; Q8K2K6-3; Q8K2K6-1; Q8K2K6-2
IFYFVNKL	8		79.5	Transmembrane 9 superfamily member 4	Q8BH24
IHYDRITSL	9		80.6	RNA helicase aquarius	Q8CFQ3
RILDFRRV	8		80.8	Pseudokinase FAM20A	Q8CID3; Q5MJS3
VCPTFGSL	8		81.1	Anoctamin-6	Q6P9J9
VNYHFTRQC	9		81.7	adical S-adenosyl methionine domain-containing protein 2 OS=Mus musculus OX=1009	Q8CBB9
RVFLFEQI	8		81.9	Rho guanine nucleotide exchange factor 25	A2CG49-2; A2CG49-4; Q0KL02-4; Q9CWR0; A2CG49; A2CG49-6; A2CG49-7; Q9CWR0-2; Q0KL02; Q0KL02-3
SNYHFGVTYV	10		82	Mitochondrial import receptor subunit TOM40 homolog	Q9QYA2
SVVLMSHL	8		82.6	Phosphoglycerate kinase 2	P09041; P09411
VNIRLVEL	8		82.8	E3 SUMO-protein ligase RanBP2	Q9ERU9
KNIDRFIPV	9		82.8	Protein YIF1B	Q9CX30; Q9CX30-2
KNYIHRDL	8		83.5	Tyrosine-protein kinase Lyn	P25911-1; P25911-2
SIIKATNL	8		83.5	Symplekin	Q80X82
RIVRFLEL	8		83.9	ATP-binding cassette sub-family D member 4	O89016
IIYDRKFLM	9		84.1	Eukaryotic translation initiation factor 4E-binding protein 1	Q60876
STYTHCEI	8		85	DNA-directed RNA polymerase II subunit RPB2	Q8CFI7
SMYVPGKL	8	1xOxidation [M2]	85.4	prefoldin subunit 5	Q9WU28
SLVTFRTL	8		85.4	Plectin	Q9QXS1-6; Q9QXS1-13; Q9QXS1-7; Q9QXS1-9; Q9QXS1-5; Q9QXS1-14; Q9QXS1-12; Q9QXS1-3; Q9QXS1-2; Q9QXS1-10; Q9QXS1-15; Q9QXS1-11; Q9QXS1-16; Q9QXS1-4; Q9QXS1; Q9QXS1-8
RIMEFTTTL	9		85.7	DNA-dependent protein kinase catalytic subunit	P97313-1
VFYEREVQM	9		86.2	V-type proton ATPase subunit d 2	Q80SY3
KNFTYSDTNL	10		86.3	Fanconi anemia group J protein homolog	Q5SXJ3
VNYKMKSV	8		86.6	cleft lip and palate transmembrane protein 1-like protein	Q8BXA5
RLYEFSCRM	9		86.9	Kelch-like protein 25	Q8R2P1
AFYYIHNL	8		87.6	Alpha-adducin	Q9QYC0-1
TNPSFDGRL	9		88.6	Atlastin-2	Q6PA06-2; Q6PA06-1
KALEYLKL	8		88.9	Cytoskeleton-associated protein 5	A2AGT5; A2AGT5-3; A2AGT5-2
KTYQFLNDI	9		89.3	Ribosomal RNA processing protein 36 homolog	Q3UFY0
KIYQWINEL	9		89.5	CCR4-NOT transcription complex subunit 9	Q9JKY0
VFQYLTRL	8		89.6	Nucleoporin NUP188 homolog	Q6ZQH8
VAYSHDGAFL	10		89.7	WD repeat-containing protein 1	O88342
SIYEKLIQF	9		89.8	SAP30-binding protein	Q02614
INRIFHERF	9		89.8	Dynamin-1	P39053; P39053-3; P39054; P39053-4; P39054-2; Q8BZ98; Q8BZ98-2; P39053-5; P39053-6
KSPDFYEEV	9		89.9	Dedicator of cytokinesis protein 8	Q8C147; Q8C147-2
IIFNFEKAYF	10		90.2	AP-1 complex subunit sigma-3	Q7TN05; P61967; Q9DB50
KTFTFDTV	8		90.2	Kinesin-like protein KIF3A	P28741
HTYDFEKL	8		90.4	Ribonucleoside-diphosphate reductase large subunit	P07742
STFSHHSM	8		90.4	Ubiquitin-associated protein 2-like	Q80X50-4; Q80X50-3; Q80X50-5; Q80X50-2; Q80X50
TNVDFPSL	8		90.7	SHC SH2 domain-binding protein 1	Q9Z179

VHYHPHPFL	9		90.8	Protein lyl-1	P27792
SQVIFHLL	8		91.1	Nucleoporin NDC1	Q8VCB1
GQYEFHSL	8		91.9	Lipopolysaccharide-binding protein	Q61805
RNPQFQKL	8		93.2	glucose-6-phosphate isomerase	P06745
VLRSFYEL	8		95.1	Dipeptidase 3	Q9DA79
SCLQFIGL	8		95.1	Inhibitor of Bruton tyrosine kinase	Q6ZPR6; Q6ZPR6-2
IAGPYNRL	8		95.3	Vigilin	Q8VDJ3
AEYIYPSL	8		95.4	Histone lysine demethylase PHF8	Q80TJ7-2; Q80TJ7
TTVAFTQV	8		95.5	60S ribosomal protein L7a	P12970
VIVRFLTVM	9		96.4	40S ribosomal protein S15a	P62245
KAPGFAQM	8		96.7	T-complex protein 1 subunit theta	P42932
TGFSYESLF	9		97.2	MIT domain-containing protein 1	Q8VDV8
VRFKHRYL	8		97.3	Ribonuclease P/MRP protein subunit POP5	Q9DB28
VGVKYVNKL	9		97.9	Solute carrier family 12 member 7	Q9WVL3; Q9WVL3-2
SGLIFNKV	8		97.9	Surfeit locus protein 6	P70279
SNYQHITNF	9		98.7	AP-3 complex subunit delta-1	O54774
SRFIFNYV	8		100.1	Polycomb protein Suz12	Q80U70
IAVQFVDM	8	1xOxidation [M8]	100.5	D-3-phosphoglycerate dehydrogenase	Q61753
KIFRFQDTGL	10		100.7	Solute carrier family 35 member C2	Q8VCX2
VADKFSEL	8		100.9	Serine/threonine-protein phosphatase 2A 65 kDa regulatory subunit A beta isoform	Q7TNP2
IIYEFESSTQM	11	1xOxidation [M11]	101.2	GON-4-like protein	Q9DB00
VNWDFVEQV	9		101.4	RING finger protein 10	Q3UIW5-1; Q3UIW5-2
RAPLFKFL	8		101.4	E3 ubiquitin-protein ligase RNF213	E9Q555
RSPVYSHF	8		103.1	Multidrug resistance-associated protein 1	O35379
ATPIFSKM	8	1xOxidation [M8]	103.3	Glutathione S-transferase Mu 1	P10649
IMIALPSL	8	1xOxidation [M2]	104.9	Cytochrome c oxidase subunit 2	P00405
STYDYGRQLL	10		105.8	SEC14 domain and spectrin repeat-containing protein 1	Q80UK0
SQYRFIVF	8		106.1	Embigin	P21995
TTPEFLTRI	9		107.2	Prostaglandin G/H synthase 2	Q05769
KVLRFIAEV	9		107.3	Melanoma-associated antigen D1	Q9QYH6
RVIDFVAQV	9		107.5	Protein mono-ADP-ribosyltransferase PARP4 OS=Mus musculus OX=10090	E9PYK3
VCFSYRNNF	9		107.5	Membrane-bound transcription factor site-2 protease	Q8CHX6
HSALIYSNL	9		107.8	Trafficking protein particle complex subunit 3	O55013
VNVPFANGI	9		108.4	Volume-regulated anion channel subunit LRRC8D	Q8BGR2
TNVTFSKV	8		111.7	RRP12-like protein	Q6P5B0
VNLVFPRC	8		111.8	Transmembrane glycoprotein NMB	Q99P91
VGYRQPLV	8		112.4	Sodium/potassium-transporting ATPase subunit beta-3	P97370
TICRFESL	8		112.5	POU domain, class 4, transcription factor 2	Q63934; Q63934-2; P17208; P17208-2; Q63955
SIIIFTNT	8		113	Probable ATP-dependent RNA helicase DDX49	Q4FZF3
AVVEFSRNV	9		113.2	CTP synthase 1	P70698
RGLRFIQV	8		113.3	Glycolipid transfer protein	Q9JL62
QQFIYEKL	8		114.5	Importin-11	Q8K2V6-2; Q8K2V6-1
SKYLHRQL	8		114.8	2'-5'-oligoadenylate synthase 1A	P11928
SNIQYITRF	9		115	Rab5 GDP/GTP exchange factor	Q9JM13
RVDVFTNL	8		115.5	THO complex subunit 6 homolog	Q5U4D9
ITGYFPSM	8	1xOxidation [M8]	115.6	Neutrophil cytosol factor 1	Q09014
RNVRYVHI	8		116.5	U7 snRNA-associated Sm-like protein LSm10	Q8QZX5
NTHEFVNL	8		116.6	Vacuolar protein sorting-associated protein 26A	P40336; P40336-2
ATQVYPKL	8		118.9	Isoform 1 of BRISC and BRCA1-A complex member 2	Q8K3W0-4; Q8K3W0-2; Q8K3W0-1; Q8K3W0-6; Q8K3W0-5
KALTYEKL	8		119.7	Serpin B8	O08800
CAPLFRNI	8		120.5	Sterol O-acyltransferase 1	Q61263
VRYIHVEL	8		120.7	E3 ubiquitin-protein ligase HERC2	Q4U2R1-2; Q4U2R1
VHYKYTVVM	9		120.7	Neutrophil cytosol factor 2	O70145
VIFNYKGKNV	10		122.2	Calreticulin	P14211

KVIEFKKL	8		122.5	Sodium/hydrogen exchanger 8	Q8R4D1; Q8R4D1-2
SAYNYAEQTM	10		123.5	RNA-binding protein 4B	Q8VE92
VSPIILRL	8		124.3	Integrin alpha-M	P05555-1; P05555-2
VGLRYEKI	8		124.7	Serine/threonine-protein kinase TBK1	Q9WUN2
NSPEYQRL	8		126.3	Ataxin-3	Q9CVD2
YNLDYTEL	8		130.1	Store-operated calcium entry-associated regulatory factor	Q8R3Q0
SLTAFNNL	8		130.5	Keratinocyte-associated protein 2	Q5RL79
IIWVKTNQL	9		130.8	N6-adenosine-methyltransferase subunit METTL3	Q8C3P7; Q8C3P7-2
IPPEYRHL	8		132.1	Calpastatin	P51125-4; P51125-7; P51125-6; P51125-5; P51125-1; P51125-3; P51125-2
KNYLLPIL	8		132.1	Corticosteroid 11-beta-dehydrogenase isozyme 1	P50172
VSPDIYQQV	9		132.2	DNA-binding protein SATB2	Q8VI24-2; Q8VI24
VNIDYTISF	9		132.2	Tripeptidyl-peptidase 2	Q64514; Q64514-2
STFDHPEL	8		132.3	T-complex protein 1 subunit beta	P80314
RVAEFTTNL	9		132.4	Myosin-9	Q8VDD5
TLIAFTKL	8		133.9	Mediator of RNA polymerase II transcription subunit 14	A2ABV5
NTYSYQKV	8		134.4	JmjC domain-containing protein 8 OS=Mus musculus OX=10090	Q3TA59; Q3TA59
SGPLEYPQL	9		135	H-2 class II histocompatibility antigen gamma chain	P04441; P04441-2
KVITFIDL	8		135.5	GTP-binding protein 1	O08582
TNIDFAFKRL	10		136.9	Beta-hexosaminidase subunit alpha	P29416
TNRKYPKL	8		137.7	DNA-directed RNA polymerase III subunit RPC10	Q9CQZ7
TAYKRIYGI	9		138	Osteocalcin-2	P86547; P86546
SSGDFPSL	8		138.2	EH domain-containing protein 1	Q9WVK4
KIVPFFKL	8		139.2	Cytoplasmic dynein 1 heavy chain 1	Q9JHU4
QIIPFKTL	8		139.6	Ribosomal L1 domain-containing protein 1	Q8BVY0
ICYIFHETF	9		140.3	Dynamin-1-like protein	Q8K1M6-3; Q8K1M6-4; Q8K1M6; Q8K1M6-2
VADKFTEL	8		140.9	serine/threonine-protein phosphatase 2A 65 kDa regulatory subunit A alpha isoform	Q76MZ3
SFYEHIITV	9		141.5	DDB1- and CUL4-associated factor 12	Q8BGZ3
KQRLFENL	8		141.8	Histone deacetylase 1	O09106; P70288
VVDIFRKL	8		142.8	H(+)/Cl(-) exchange transporter 4	Q61418; P51791-2; Q9WVD4; P51791
KVFQFLNA	8		143.9	60S ribosomal protein L24	Q8BP67
NVYTYEAV	8		144	RING-type E3 ubiquitin-protein ligase PPIL2	Q9D787
KNYSYLHC	8		145.8	Chromodomain-helicase-dna-binding protein 8	Q09XV5; Q8BYH8
TCFVFKEL	8		146	Protein FAM111A	Q9D2L9
EIISFQHL	8		146.9	Volume-regulated anion channel subunit LRRC8D	Q5DU41; Q80WG5; Q8BGR2; Q5DU41-2
SHYDFGLRAL	10		147.1	Cytoplasmic dynein 1 heavy chain 1	Q9JHU4
AILERFPTI	9		147.7	Fanconi anemia group C protein homolog	P50652
LSPPSYSKL	9		148.2	Probable phospholipid-transporting ATPase IIB	P98195-2; P98195
TNQDFIQRL	9		149.4	Nischarin	Q80TM9-3; Q80TM9-2; Q80TM9-1
IIVSFVNA	8		149.8	Splicing factor 3B subunit 3	Q921M3-2; Q921M3
IWITAAKL	8		150	Pre-mRNA-processing factor 6	Q91YR7; Q91YR7-2
SNYYRTWNV	9		152	Sterol O-acyltransferase 1	O88908; Q61263
LSPKYIKM	8	1xOxidation [M8]	152.1	Eukaryotic initiation factor 4A-I	P60843
KGIIYRDL	8		152.1	Protein kinase C beta type	P68404; P23298; P28867-2; Q8BSK8; Q8BSK8-2; P68404-2; P0C605-2; P0C605-1; P28867-1
SVVPFIAV	8		152.1	Phosphatidylcholine translocator ABCB4	P21440
TTPTYGDL	8		152.8	Tubulin beta-2A chain	Q7TMM9; Q9D6F9; A2AQ07; P99024; P68372; Q9CWF2; Q922F4
STCEFVRTL	9		154.9	F-box/WD repeat-containing protein 1A	Q5SRY7-2; Q3ULA2-2; Q5SRY7-3; Q5SRY7-4; Q3ULA2; Q5SRY7
VWYRVIQI	8		155.6	AP-2 complex subunit alpha-2	P17426-2; P17427; P17426
VSLEFPQCV	9		155.9	Nuclear receptor 2C2-associated protein	Q3TV70
GNYKWINYL	9		156.6	Long-chain-fatty-acid--CoA ligase 4	Q9QUJ7; Q9QUJ7-2
RGIIYRDL	8		157.7	Protein kinase C iota type	Q02956-2; Q62074; Q02956; P20444
AICIFREL	8		158.9	FACT complex subunit SSRP1	Q08943-2; Q08943
SIIKLTTL	8		159.7	Cleavage and polyadenylation specificity factor subunit 2	O35218

HTFRYAVF	8		160.2	Nitric oxide synthase, inducible	P29477
FSPSFINHI	9		161.1	Fas-activated serine/threonine kinase	Q9JIX9
STFDFRTGKM	10		162.2	Methyl-CpG-binding domain protein 3	Q9Z2D8-2; Q9Z2D8
SGFPTLGLL	9		164.4	Maestro heat-like repeat-containing protein family member 7	A2AVR2
RAPKFTQV	8		164.9	Discoidin, CUB and LCCL domain-containing protein 2	Q91ZV3
TAFEFNEYF	9		165.4	Myotubularin-related protein 2	Q9Z2D1
VNRKFGNRI	9		165.8	Protein O-mannosyl-transferase 2	Q8BGQ4-3; Q8BGQ4-2; Q8BGQ4
KEFEFSQL	8		166.9	signal recognition particle receptor subunit beta	P47758
VMVEHKLGL	9		167.1	Proteasome maturation protein	Q9CQT5
VQQYYRVL	8		168.9	eIF-2-alpha kinase activator GCN1	E9PVA8
SRLPFTAL	8		169.7	Proteasome subunit beta type-10	O35955
VTFERVEQM	9		169.8	Transient receptor potential cation channel subfamily M member 7	Q923J1
INKKFPNI	8		170.3	Nck-associated protein 1-like	Q8K1X4
KIFTASNV	8		171.6	Ubiquitin-associated protein 2-like	Q80X50-4; Q80X50-3; Q80X50-5; Q80X50-2; Q80X50
VVADFGLARL	10		171.6	LIM domain kinase 1	P53668
KNVVYRDL	8		172.5	RAC-alpha serine/threonine-protein kinase	P31750
ATRLMAQL	8		173.5	Ribosomal biogenesis factor	Q0VG62; Q0VG62-1
VIASFKVL	8		173.6	Alpha-actinin-4	P57780
AVVEFLTSV	9		175.6	Ribonuclease 3	Q5HZJ0
AVLKYYKV	8		177	Ubiquitin-40S ribosomal protein S27a	P62983
VNVDHPINL	9		178.5	RING finger protein 10	Q3UIW5-1; Q3UIW5-2
SVFAFGENKMG	13		178.9	Protein RCC2	Q8BK67
QQYRFSVIM	9		181.2	Inverted formin-2	Q0GNC1; Q0GNC1-3
SIMFMPSL	8	2xOxidation [M3; M5	182.7	Calcium-activated chloride channel regulator 2	Q8BG22; Q8BG22-2
IIGTFERM	8		184.8	Vacuolar protein sorting-associated protein 35	Q9EQH3
AIVEYRDL	8		186.3	Serrate RNA effector molecule homolog	Q99MR6-3; Q99MR6; Q99MR6-4; Q99MR6-2
SVISVIHL	8		187.5	CDP-diacylglycerol--inositol 3-phosphatidyltransferase	Q8VDP6
VIQDFVKM	8		189.8	CGG triplet repeat-binding protein 1	Q8BHG9
VCVTYEHL	8		190.3	Bifunctional glutamate/proline--tRNA ligase	Q8CGC7
VAPRYNWM	8		191.7	ATP-binding cassette sub-family A member 3	Q8R420
QVYGFLEV	8		191.7	Endoplasmic reticulum-Golgi intermediate compartment protein 3	Q9CQE7-2; Q9CQE7
SITKFLNRI	9		195.8	Protein strawberry notch homolog 2	Q7TNB8-1; Q7TNB8-2
SAPIYKRI	8		197.1	exosome complex exonuclease RRP44	Q9CSH3
ITPPGYSHV	9		197.3	Lethal(3)malignant brain tumor-like protein 3	Q8BLB7-2; Q8BLB7
KVQEFVLL	8		198.9	Protein dopey-2	Q3UHQ6; Q3UHQ6-2
AINIFQKL	8		199.3	Interferon-inducible protein AIM2	Q91VJ1
AVIDFSEAHL	10		199.7	Nucleic acid dioxygenase ALKBH1	P0CB42
RNLTFHKM	8		200	Cytochrome b-245 heavy chain	Q61093
VGLYYINKI	9		200.2	Keratinocyte-associated protein 2	Q5RL79
KIQSFINRM	9		202.8	vacuolar fusion protein CCZ1 homolog	Q8C1Y8
SSFYPSLTV	9		206.2	HMG box-containing protein 1	Q8R316-2; Q8R316
KNLVYGTI	8		206.8	Poly [ADP-ribose] polymerase 12	Q8BZ20
RIYRFDTVI	9		210	Isoform 2 of Myomegalin	Q80YT7-2
SLLSFEKL	8		210.6	Protein lin-52 homolog	Q8CD94
KVLEFERV	8		211.9	Peptidylprolyl isomerase domain and WD repeat-containing protein 1	Q8CEC6
TNYRFKNLFI	10		212.2	G1/S-specific cyclin-E2	Q9Z238
IGPEYKSM	8		212.5	Pyridoxal-dependent decarboxylase domain-containing protein 1	Q99K01
TCFPFTSRF	9		212.6	Inactive rhomboid protein 2 OS=Mus musculus OX=10090	Q80WQ6
TGVEYFNTF	9		214.2	5-phosphohydroxy-L-lysine phospho-lyase	Q8R1K4-2; Q8R1K4
ANYQRDGPM	9	1xOxidation [M9]	214.7	catalase	P24270
RDYQFKRL	8		215.7	ESF1 homolog	Q3V1V3
AVVRFINRF	9		216.3	Myotubularin-related protein 5	Q6ZPE2-2; Q6ZPE2
ACLMFKHL	8		218.9	ribonucleoside-diphosphate reductase subunit M2	P11157
VWITRAPGM	9		219.3	CCR4-NOT transcription complex subunit 2	Q8C5L3-2; Q8C5L3; Q8C5L3-3

RSPDWYNKV	9		220.5	StAR-related lipid transfer protein 8	Q8K031
VSVEYTEKM	9		225.2	Ribosomal RNA small subunit methyltransferase Nep1	O35130
ARYIHRRL	8		225.4	Sn1-specific diacylglycerol lipase beta	Q91WC9
HFLEKFGPL	9		227.3	Vacuolar protein sorting-associated protein 18 homolog	Q8R307
ANPRLWLRL	9		228.4	CCR4-NOT transcription complex subunit 10	Q8BH15-4; Q8BH15-3; Q8BH15-2; Q8BH15
ATFIREVLM	9		230.8	Prostaglandin G/H synthase 1	P22437
SCYTFPLRI	9		231.2	Ubiquitin carboxyl-terminal hydrolase 40	Q8BWR4; Q8BWR4-3; Q8BWR4-4; Q8BWR4-2
RIYEYTSC	8		231.3	F-box only protein 3	Q9DC63
SAYQRGESL	9		233.2	Exocyst complex component 4	O35382
KNHEFIATF	9		233.3	protein kinase C delta type	P28867-2; P28867-1
SSIIFEDI	8		234.4	LMBR1 domain-containing protein 2	Q8C561; Q8C561-3; Q8C561-2
KQWTFNYV	8		235.7	Rab-like protein 6	Q5U3K5
VNIEHRDL	8		237.6	MORC family CW-type zinc finger protein 2A	Q69ZX6
TQQDYVNL	8		237.9	V-type proton ATPase subunit d 2	Q80SY3
SFYGSSSL	8		238.8	Pumilio homolog 2	Q80U58-3; Q80U58-2; Q80U58
EAITFKNL	8		239.4	Calpain-1 catalytic subunit	O35350
VNVERVLNV	9		239.5	Probable helicase with zinc finger domain	Q6DFV5; Q6DFV5-2; Q6DFV5-3
KGYDFDTV	8		244	MLX-interacting protein	Q2VPU4; Q2VPU4-2
RVIDFTVL	8		246.4	Lethal(2) giant larvae protein homolog 2	Q3TJ91
ESLRYKLL	8		246.7	40S ribosomal protein S3	P62908
RGVEFVHV	8		247.2	UDP-N-acetylhexosamine pyrophosphorylase-like protein 1	Q3TW96; Q3TW96-2
KNFAFLEF	8		247.3	Splicing factor U2AF 65 kDa subunit	P26369
KSCQFVAV	8		247.6	Bifunctional glutamate/proline--tRNA ligase	Q8CGC7
TQQLYPSL	8		248.3	Ran-binding protein 9	P69566-2; P69566
SWLLARGPL	9		248.4	Tubulin epsilon and delta complex protein 1	Q3UK37
VIFTSETGM	9	1xOxidation [M9]	248.5	Mitochondrial import inner membrane translocase subunit TIM50	Q9D880
SLYDAFPKV	9		249.3	Dystonin	Q91ZU6-8; Q91ZU6-3; Q91ZU6-4; Q91ZU6-2; Q91ZU6-6; Q91ZU6
LSFRSLLGV	9		251.5	Centromere protein P	Q9CZ92
VNVAKLRYM	9		256.1	Serine/threonine-protein kinase RIO2	Q9CQS5
VAFSRSGRLLL	11		261.8	Guanine nucleotide-binding protein G(I)/G(S)/G(T) subunit beta-2	P62880
VHIKLPHL	8		262.7	Creatine kinase B-type	Q04447
TFFTPGNL	8		262.9	Probable ATP-dependent RNA helicase DDX17	Q501J6
VMVQPINL	8		263.1	Small nuclear ribonucleoprotein E	P62305
ITHEFINTF	9		268	Ribonuclease P protein subunit p40	Q8R1F9
HILDFTCRL	9		268.7	Nischarin	Q80TM9-1
EQYKFYSV	8		269.9	Cytochrome c oxidase subunit NDUFA4	Q62425
SYFKGASL	8		270.1	Leucyl-cystinyl aminopeptidase	Q8C129
ISPPIPHL	8		270.5	TBC1 domain family member 9B	Q5SVR0; Q5SVR0-2
VNFGRQGLNL	10		270.9	Receptor expression-enhancing protein 3	Q99KK1
SIVPMVHL	8		274.9	Lysine-specific demethylase 6A	O70546; O70546-2
SFSDYPPL	8		275.3	Elongation factor 1-alpha 1	P10126
IIQEFPEI	8		277.4	Uracil phosphoribosyltransferase homolog	B1AVZ0
INHNFSQQCL	10		277.8	Eukaryotic translation initiation factor 3 subunit D OS=Mus musculus OX=10090	O70194
RVLGFVKL	8		277.8	4-trimethylaminobutyraldehyde dehydrogenase	Q9JLJ2
SMVSLRAL	8		280.7	Carbonyl reductase [NADPH] 1	P48758
SVVVFQSF	8		282.8	Interferon-induced transmembrane protein 2	Q99J93
AQYKYIVI	8		285.5	Transmembrane protein 11, mitochondrial	Q8BK08
TAVPFVNGV	9		287	Protein FAM102A	Q78T81
EVYLFERI	8		288.1	Vacuolar protein sorting-associated protein 45	P97390
RSVQVVNL	8		288.4	GPN-loop GTPase 3	Q9D3W4
AQQMYQSL	8		291.5	Hydroxyacylglutathione hydrolase-like protein	Q9DB32; Q9DB32-2
QIPVFVTM	8	1xOxidation [M8]	292.3	Transmembrane glycoprotein NMB	Q99P91
VFYAVKVL	8		295.7	Serine/threonine-protein kinase Sgk1	Q9WVC6-3; Q9WVC6-2; Q9WVC6
RCIRFWNTL	9		298.9	Fizzy-related protein homolog	Q9R1K5

VFRLLPQL	8	299.9	Acidic leucine-rich nuclear phosphoprotein 32 family member B	Q9EST5-1; Q9EST5-2
NTYRFLTF	8	301.7	Small subunit processome component 20 homolog	Q5XG71
KNVTFEHV	8	302.9	ATP-dependent zinc metalloprotease YME1L1	O88967
RLPLYLRL	8	303.5	Short transient receptor potential channel 4-associated protein	Q9JLV2; Q9JLV2-2
VSPGPHLNM	9	303.9	structural maintenance of chromosomes protein 5	Q8CG46-1; Q8CG46-2
VNNFFQLTV	9	306.6	Huntingtin-interacting protein 1	Q8VD75
VVADFGLSRL	10	307.6	LIM domain kinase 2	O54785; O54785-2; O54785-3
IYFKVTHV	8	308.1	Pre-mRNA-splicing factor 38B	Q80SY5
RVLQFKQV	8	309	N-acetylgalactosamine kinase	Q68FH4
KQFEYIEV	8	309.5	Huntingtin	P42859; P42859-2
RGVDYHAL	8	312.2	Deoxyhypusine synthase	Q3TXU5
NAPTFGSL	8	312.2	Nuclear pore complex protein Nup214	Q80U93
TIIKPFNL	8	316.3	Targeting protein for Xklp2	A2APB8
TSVRVMEL	8	318	Rab proteins geranylgeranyltransferase component A 2	Q9QZD5
TNMASFPKM	9	319.7	Cathepsin K	P55097
SCVHFMTL	8	321.5	THO complex subunit 5 homolog	Q8BKT7
VNAQFPRF	8	322.2	Aladin	P58742
KAFTHGVAM	9	323.4	Gamma-adducin	Q9QYB5-2; Q9QYB5
SWIAVSAL	8	325.3	Lysine-specific demethylase 2B	Q6P1G2-2; Q6P1G2
RCWQYRQL	8	328.5	60S ribosomal protein L15	Q9CZM2
ESFSDYPPL	9	329.2	Elongation factor 1-alpha 1	P10126
QVVNFLQTF	9	332.8	ZW10 interactor	Q9CQU5
RLLDWFRSL	9	333	ADP-ribosylation factor-like protein 8B	Q9CQW2
KALDYIHHM	9	336.5	STE20-related kinase adapter protein alpha	Q3UUJ4; Q3UUJ4-2; Q3UUJ4-3
KNVVYERV	8	337.2	Protein POF1B	Q8K4L4
IICEYPSI	8	340.8	DNA polymerase epsilon subunit 2	O54956
AIITGFRNV	9	341.5	Mitotic checkpoint serine/threonine-protein kinase BUB1 beta	Q9Z1S0
KNYRIATF	8	346.2	Baculoviral IAP repeat-containing protein 5	O70201; O70201-2; O70201-3
EIYEFHKM	8	349.3	Neutrophil cytosol factor 1	Q09014
KALQFLEQV	9	353	T-complex protein 1 subunit zeta	P80317
VGPEFKDKL	9	361	plectin	Q9QXS1-1
SILAMINNM	9	361.5	CCR4-NOT transcription complex subunit 8	Q9D8X5
RNLSFVARQM	10	363.9	Isoform E of Tuberin	Q61037-6; Q61037-7
KNLEVFMHV	9	366.1	Fatty acyl-CoA reductase 1	Q922J9-3; Q922J9-4; Q922J9-2; Q922J9
KIVQFIVTL	9	367.1	Heat shock factor protein 2	P38533-2; P38533
KTLILPRL	8	368.8	Transcription initiation factor TFIID subunit 6	Q62311
SSLHPMGGL	9	369	CUGBP Elav-like family member 1	P28659-2; P28659-1; P28659-3; P28659-4
AGPGFINV	8	372.2	Arginine--tRNA ligase, cytoplasmic	Q9D0I9
RVIDFFTV	8	373.1	Lethal(2) giant larvae protein homolog 1	Q80Y17
ACPEYSRL	8	374.3	TATA element modulatory factor	B9EKI3
SVFAFGENKM	10	377.8	Protein RCC2	Q8BK67
KIVEFLQSF	9	379.4	Sarcoplasmic/endoplasmic reticulum calcium ATPase 2	O55143-2; O55143
QRVEFAAL	8	381	Nuclear mitotic apparatus protein 1	E9Q7G0
SLYEHVERM	9	383.3	Pleckstrin homology domain-containing family M member 1	Q7TSI1
SRVLFNQL	8	388.8	Dedicator of cytokinesis protein 1	Q8BUR4; Q8BUR4-2
NIIQFYGV	8	393.6	Mitogen-activated protein kinase kinase kinase 20	Q9ESL4-2; Q9ESL4-3; Q9ESL4
TIVEFLHSF	9	395.2	Bromodomain adjacent to zinc finger domain protein 2A	Q91YE5-2; Q91YE5; Q91YE5-3
AIVEHLVTL	9	401	NF-kappa-B inhibitor alpha	Q9Z1E3
SQYIRNCGV	9	403	Lysozyme C-1 OS=Mus musculus OX=10090	P17897; P08905
KAGKFPSL	8	403.4	60S ribosomal protein L10a	P53026
SSPSFPNFF	9	404.9	Hydroxycarboxylic acid receptor 2	Q9EP66
SIFEHKIVF	9	409.8	Zinc transporter ZIP6	Q8C145
EIVSFQHL	8	411.6	Volume-regulated anion channel subunit LRRC8C	Q8R502
IERYFPAL	8	427	UAP56-interacting factor	Q91Z49-3; Q91Z49; Q91Z49-2

INYTNEKL	8		429.6	Myosin-11	Q6URW6-2; O08638-2; O08638-1; Q8VDD5; Q6URW6-1; Q6URW6-3; Q61879
SGVDYRGV	8		431.9	U6 snRNA-associated Sm-like protein LSm6	P62313
SNVKHVINF	9		436.4	ATP-dependent RNA helicase DDX3Y	Q62095; Q62167; P16381
IRYFPTQAL	9		437.1	ADP/ATP translocase 1	Q3V132; P48962; P51881
INMPREVSV	9		449.7	Interferon-induced transmembrane protein 2	Q99J93; Q9CQW9
QVVEFKKL	8		449.9	Intraflagellar transport protein 52 homolog	Q62559
KNIFYKAI	8		450.2	lanosterol 14-alpha demethylase	Q8K0C4
RQRENFPNL	9		451.1	Isoform 2 of FLYWCH-type zinc finger-containing protein 1	Q8CI03-3; Q8CI03-2; Q8CI03-1
NVYLFNLSI	9		452.3	Succinate receptor 1	Q99MT6
IGPRYSSVF	9		460.2	HEAT repeat-containing protein 5A	Q5PRF0
KIFSFAHQTI	10		462.7	Vacuolar protein sorting-associated protein 35	Q9EQH3
SNLELHSL	8		485	Proteasome subunit beta type-7	P70195
AIYCPPKL	8		490.8	26S proteasome non-ATPase regulatory subunit 11	Q8BG32
ICIKFDPM	8		491.7	THO complex subunit 3	Q8VE80
TTFYALSM	8	1xOxidation [M8]	498.1	Serpin B11	Q9CQV3
SIVTIIHL	8		500.9	Calcitonin gene-related peptide type 1 receptor	Q9R1W5
SWWTHVEM	8		501.3	Aspartate aminotransferase, mitochondrial	P05202
VTPPMPLL	8		520.1	Poly(U)-binding-splicing factor PUF60	Q3UEB3-2; Q3UEB3; Q3UEB3-3
SVVDLTCRL	9		522.8	glyceraldehyde-3-phosphate dehydrogenase	Q64467; P16858
RGPEYLTQM	9		523.1	Importin-5	Q8BKC5-2; Q8BKC5
SVVVFRII	8		529.9	Tumor necrosis factor receptor superfamily member 22	Q9ER62
VINFDFPKL	9		530.2	Probable ATP-dependent RNA helicase DDX6	P54823
TIQEFLERI	9		530.8	SAM domain-containing protein SAMSN-1	P57725
VNFNHIHKRI	10		535.9	phosphatidylinositol 4-kinase alpha	E9Q3L2
ESPLFNNV	8		546	Vacuolar protein-sorting-associated protein 25	Q9CQ80; Q9CQ80-2
AYWRQAGL	8		556.5	ATP synthase subunit epsilon, mitochondrial	P56382
TVIKLENL	8		556.5	Toll-like receptor 13	Q6R5N8
SCLEFSLRI	9		560.5	E3 ubiquitin-protein transferase MAEA OS=Mus musculus OX=10090	Q4VC33
EITTFSRL	8		570.5	E3 ubiquitin-protein ligase RNF213	E9Q555
VWIKPSGL	8		585.7	Integrator complex subunit 14	Q8R3P6
RAYEFAERC	9		587.5	Clathrin heavy chain 1	Q68FD5
VRYSHEKL	8		591.2	Alanine--tRNA ligase, cytoplasmic	Q8BGQ7
EVFDFRGM	8		591.2	Nck-associated protein 1	P28660; P28660-2
IPPEYRKL	8		591.6	Calpastatin	P51125-7; P51125-6; P51125; P51125-3; P51125-2
AQFEHTIL	8		606.6	Methionine aminopeptidase 2	O08663; Q8BP48
VFIDLPTL	8		609.3	Paraplegin	Q3ULF4
VNVGRGYGL	9		609.8	Supervillin	Q8K4L3; Q8K4L3-3
SMIIIRTL	8		612.4	Transmembrane 9 superfamily member 4	Q8BH24
KAPEYLHRF	9		620.4	Ras GTPase-activating protein-binding protein 2	P97379-1; P97379-2
IIYPPPEV	8		622.2	splicing factor 3A subunit 1	Q8K4Z5
SMIRLSESM	9		627.5	DNA replication licensing factor MCM6	P97311
ESPSYRTL	8		637.4	PHD finger protein 20	Q8BLG0-2; Q8BLG0
VVYDLSIRGF	10		641.5	Translin	Q62348
VAVDFGNYHL	10		642.8	large neutral amino acids transporter small subunit 1	Q9Z127
VQFDYSQERV	10		642.9	Dual adapter for phosphotyrosine and 3-phosphotyrosine and 3-phosphoinositide	Q9QXT1
TTLPHMLM	8		645.3	Transcriptional repressor p66-beta	Q8VHR5-2; Q8VHR5
LSAFGFLGL	9		656.4	Alpha-1,3-galactosyltransferase 2	Q3V1N9-2; Q3V1N9
VAYDWSERNSS	12		658.6	Dystonin	Q91ZU6-3; Q91ZU6-4; Q91ZU6-2; Q91ZU6-5; Q91ZU6
SCYEHIQV	8		659.8	Myotubularin-related protein 4	Q91XS1; Q91XS1-2
FQQEFPSL	8		661.9	Protein Prrc2c	Q3TLH4; Q3TLH4-5
QGPQFVTGV	9		669.3	La-related protein 7	Q05CL8
RGLRYIHSM	9		670.3	Wee1-like protein kinase	P47810
TITDFINI	8		672.4	5'-AMP-activated protein kinase subunit gamma-2	Q91WG5-2; Q91WG5; O54950

KDYVFKEL	8		684	RNA polymerase II elongation factor ELL2	Q3UKU1
IMIERGELF	9		685	Translation initiation factor eIF-2B subunit alpha	Q99LC8
SIIAMKTL	8		690	WD repeat and FYVE domain-containing protein 3	Q6VNB8; Q6VNB8-2
SCPTFLRM	8		690.9	Ubiquitin-protein ligase E3A	O08759
SSLNLRETNL	10		692.3	Vimentin	P20152
KGLTYITI	8		694.3	Sodium/potassium/calcium exchanger 5	Q8C261
IWLAAVKL	8		696.4	Pre-mRNA-processing factor 6	Q91YR7
AQQSYERL	8		696.6	Formin-binding protein 1-like	Q8K012-2; Q8K012
LNVEFKDL	8		700.1	DENN domain-containing protein 4C	A6H8H2-1
VAVPLIGKL	9		700.7	Derlin-1	Q99J56
VFRELPSL	8		713.9	General transcription factor IIH subunit 4	O70422
VDYDFSYHQM	10		714.1	Mini-chromosome maintenance complex-binding protein	Q8R3C0
VAEFVQSL	8		722.9	Lethal(3)malignant brain tumor-like protein 4	B1B1A0
VYIEHRLM	8		730.4	DNA replication licensing factor MCM7	Q61881
VWINAHGL	8		731.9	Aldehyde dehydrogenase family 16 member A1	Q571I9
LSVPFEKHSTL	11		732.9	6-phosphogluconolactonase	Q9CQ60
SRYTGASL	8		738.9	Histone deacetylase 3	O88895-1
YCLKFTKL	8		748.1	STAGA complex 65 subunit gamma	Q9CZV5
SVVSFDKV	8		752.5	Scaffold attachment factor B1	D3YXK2
AVWNFGAVGM	10		754.1	presenilin-2	Q61144-1; Q61144-2
AIVQFTRTF	9		763.4	vacuolar fusion protein CCZ1 homolog	Q8C1Y8
SSLEMTTTF	9		766.4	Thymocyte nuclear protein 1	Q91YJ3
RIYSFGLGGNGC	13		770.9	Probable E3 ubiquitin-protein ligase HERC4	Q6PAV2-2; Q6PAV2
VWREVTTQL	9		772.2	Protein tweety homolog 2	Q3TH73; Q3TH73-2
TNIELATV	8		774.4	Proteasome subunit alpha type-5	Q9Z2U1
KRLNFHLYM	9		777	Bystin	O54825
AIYGFGDKGSL	11		779.3	saccharopine dehydrogenase-like oxidoreductase	Q8R127
VQHLTAYGL	9		779.7	KICSTOR complex protein SZT2	A2A9C3; A2A9C3-2
MRYVASYLL	9		781.5	60S acidic ribosomal protein P2	P99027
RCFSFLSV	8		783.1	Sphingosine kinase 2	Q9JIA7
RVTSFRDL	8		798.3	NSFL1 cofactor p47	Q9CZ44-2; Q9CZ44-3; Q9CZ44
VCDVFQHL	8		804.4	Ras-like protein family member 11A OS=Mus musculus OX=10090	Q6IMB1
EVIDFSSL	8		809.6	Cysteine-rich protein 2-binding protein	Q8CID0
TCLDYSNM	8		810.9	NADH dehydrogenase [ubiquinone] 1 alpha subcomplex subunit 8	Q9DCJ5
VHYDRSGRSL	10		818.4	THO complex subunit 4	O08583-2; O08583
RSLAYIHSF	9		818.5	Glycogen synthase kinase-3 beta	Q9WV60
VGVKYVNKF	9		821.7	Solute carrier family 12 member 4	Q9JIS8; Q91V14-2; Q91V14
IIQDFRGTLL	10		826.1	Probable arginine--tRNA ligase, mitochondrial	Q3U186
TLVHPFRAL	9		837.9	Nucleolar protein 14	Q8R3N1
LQQYYVKL	8		846.2	spliceosome RNA helicase DDX39B	Q9Z1N5; Q8VDW0-2; Q8VDW0-1
IERPTYTNL	9		848.9	Tubulin alpha-3 chain	P05214; Q9JJZ2; P68369; P05213; P68373; P68368
RCIEFTKL	8		864.9	WD repeat-containing protein 7	Q920I9
KAYKYIVTC	9		873.5	Dynein light chain Tctex-type 3	P56387
SCIRLAEL	8		876.4	DNA (cytosine-5)-methyltransferase 1	P13864-2; P13864
TSVPRGAPF	9		893.2	Nucleobindin-1	Q02819
RIIEFQARC	9		895.3	Unconventional myosin-VIIa	P97479-1
EIITFTAM	8	1xOxidation [M8]	907.5	E3 ubiquitin-protein ligase UBR4	A2AN08-3; A2AN08-4; A2AN08-5; A2AN08
NKMLFSHL	8	1xOxidation [M3]	920.4	Linker for activation of T-cells family member 2	Q9JHL0; Q9JHL0-2
ISDIHTKL	8		925.2	Serine/threonine-protein kinase TBK1	Q9WUN2
KAPNYSCV	8		939.5	mRNA export factor	Q8C570
VILHEDYSKL	10		953.4	Inactive serine protease 39	O70169
VAPFLRQEF	9		955.8	Probable ATP-dependent RNA helicase DDX27	Q921N6
KMLSKLETV	9		956.4	BRCA1-associated RING domain protein 1	O70445
ARIFFTYM	8		971.7	Acyl-CoA (8-3)-desaturase	Q920L1

RSNYVSLL	8		971.9	NAD-dependent malic enzyme, mitochondrial	Q99KE1
KWDFIHMIQM	10	2xOxidation [M7; M1	983	Histamine N-methyltransferase	Q91VF2
KGPRYWEL	8		992	Anaphase-promoting complex subunit 1	P53995
SCVDFRNM	8		1010.2	Negative regulator of reactive oxygen species	Q8BMT4
IRTGFINL	8		1010.4	H/ACA ribonucleoprotein complex subunit DKC1	Q9ESX5
IRAPGFARL	9		1012.4	Glutamine amidotransferase-like class 1 domain-containing protein 1	Q8BFQ8
AMAPRTLLL	9		1015.9	H-2 class I histocompatibility antigen, Q10 alpha chain	P01898; P01900; P01897; P01899
QNVEFIEV	8		1016.1	Serine/threonine-protein kinase ATR	Q9JKK8
LSFDFKDTDM	10	1xOxidation [M10]	1019	Merlin	P46662; P46662-2
RICTFEGKL	9		1022.8	EPM2A-interacting protein 1	Q8VEH5
IWLKVNRV	8		1032.7	Macrophage colony-stimulating factor 1 receptor	P09581
EGYKFCKI	8		1038.6	DNA-directed RNA polymerase II subunit RPB2	Q8CFI7
QTYDYAKTIL	10		1043.5	ATP-citrate synthase	Q91V92
NIFQKLNYM	9		1053	Interferon-inducible protein AIM2	Q91VJ1
KGFSYLVTA	9		1067.6	Cytochrome b-c1 complex subunit Rieske, mitochondrial	Q9CR68
LCPPRYPKL	9		1072.7	Chloride intracellular channel protein 1	Q9Z1Q5
VIAELVNV	8		1075.9	General transcription factor IIH subunit 5	Q8K2X8
IGLDYSSLYM	10	1xOxidation [M10]	1085.4	Class E basic helix-loop-helix protein 41	Q99PV5
SSLLTKSQI	9		1099	THUMP domain-containing protein 3	P97770; P97770-2
YGVSGYPTL	9		1106.4	Protein disulfide-isomerase A3	P27773
EVFDFRGMRL	10		1108.8	Nck-associated protein 1	P28660; P28660-2
EIVTFERL	8		1126.5	Fragile X mental retardation syndrome-related protein 1	Q61584; Q61584-5; Q61584-3; Q61584-6; Q61584-4; Q61584-2; Q61584-7
VAYPTDLSTI	10		1134.5	PH-interacting protein	Q8VDD9
AMGVNLTSM	9	1xOxidation [M9]	1147.9	proliferating cell nuclear antigen	P17918
IWIKRGDFL	9		1156.3	Probable RNA-binding protein EIF1AD	Q3THJ3
VNDIFERI	8		1171.7	Histone H2B type 1-P	Q64475; Q8CGP2; Q64525; Q64524; Q8CGP1; P10854; Q64478; Q9D2U9; Q8CGP2-2; Q6ZWY9; Q8CGP0; P10853
SCPYFCSV	8		1185.8	Ribonuclease inhibitor	Q91VI7
RNVAKLLYM	9		1192	AP-1 complex subunit gamma-1	P22892
QILSDFPKL	9		1193.6	Nucleolar GTP-binding protein 1	Q99ME9
RNTPFMGI	8		1194.9	Protein TANC1	Q0VGY8
RSLSYSEILRL	11		1228	Engulfment and cell motility protein 2	Q8BHL5; Q8BHL5-2; Q8BHL5-3
AQQSYKSL	8		1252.4	Ubiquitin-conjugating enzyme E2 Q1	Q7TSS2-2; Q7TSS2
RAPSYIEI	8		1287.5	E3 ubiquitin-protein ligase UBR4	A2AN08-3; A2AN08-5; A2AN08
KGPSFDVQV	9		1291.9	Dipeptidyl peptidase 3	Q99KK7
LPGRFSHL	8		1301	Tumor necrosis factor-inducible gene 6 protein	O08859
VTDLRVTGM	9		1311	N-alpha-acetyltransferase 35, NatC auxiliary subunit	Q6PHQ8
SMLYPLSHGF	10		1355.1	UDP-N-acetylglucosamine--peptide N-acetylglucosaminyltransferase 110 kDa subunit	Q8CGY8-2; Q8CGY8-1
QRIKFINM	8		1360.6	Nuclear receptor corepressor 2	Q9WU42; Q9WU42-2
KICKFTEV	8		1365.2	60S ribosomal protein L13a	P19253
RSIKNVTEL	9		1370.7	protein RTF2 homolog	Q99K95
KLYTHSYLGF	10		1372.7	Ectonucleoside triphosphate diphosphohydrolase 5	Q9WUZ9
SMVDVVML	8	2xOxidation [M2; M7	1390.9	Large proline-rich protein BAG6	Q9Z1R2
AYQHLFYL	8		1408.8	Ras GTPase-activating-like protein IQGAP1	Q9JKF1
VFIELNHI	8		1422.9	Isoform 14 of MAP kinase-activating death domain protein	Q80U28-14
NCLLFIQRL	9		1430.7	Centromere protein W	Q3URR0
SCIVLSAL	8		1473.3	Mast cell-expressed membrane protein 1	Q9D8U6
SCVNFKEMM	9		1476.6	Interleukin-18	P70380
SCNIFRTL	8		1489	Serine/threonine-protein phosphatase 2A 56 kDa regulatory subunit epsilon isoform	Q61151
SAPENAVRM	9		1503.2	Protein C10	O35127
SAPRNFVENF	10		1507.1	Elongator complex protein 2	Q91WG4-2; Q91WG4
SCPVFTSI	8		1525	Solute carrier family 35 member G1	Q8BY79
TLLLYQELM	9	1xOxidation [M9]	1550.2	Serine/threonine-protein kinase TBK1	Q9WUN2

KIFKKEKEM	9		1582	proliferation-associated protein 2G4	P50580; P50580-2
ANPTFPNFF	9		1599.1	Glutaminyl-peptide cyclotransferase	Q9CYK2-2; Q9CYK2
SWLHTSEL	8		1600.3	Large subunit GTPase 1 homolog	Q3UM18; Q3UM18-2
SLLPSSNL	8		1623.5	Ribonucleoprotein PTB-binding 1	Q9CW46
SRVTFVNF	8		1632.4	Cytoplasmic dynein 1 heavy chain 1	Q9JHU4
SWIEVQFL	8		1634	E3 ubiquitin-protein ligase ARIH1	Q9Z1K5
SRFQPLNL	8		1667.6	Genetic suppressor element 1	Q3U3C9-4; Q3U3C9-3; Q3U3C9-2; Q3U3C9
VLAVLPRL	8		1686.3	Probable rRNA-processing protein EBP2	Q9D903
LQSQYRSL	8		1689.5	Rho guanine nucleotide exchange factor 7	Q9ES28; Q9ES28-7; Q9ES28-5; Q9ES28-3; Q9ES28-4; Q9ES28-6; Q9ES28-2
RNYQRKNDM	9		1694.1	Chromodomain-helicase-DNA-binding protein 4	Q6PDQ2; A2A8L1
RIADFGAAARL	11		1710.3	Mitogen-activated protein kinase kinase kinase 1	P53349
PRLNFSTIFKSL	12		1716.8	Isoform 2 of Iroquois-class homeodomain protein IRX-3	P81067-2
SWIAVQEL	8		1736.3	Pyroglutamyl-peptidase 1	Q9ESW8
SLNLRETNL	9		1745.1	Vimentin	P20152
AWIHAAHV	8		1760.9	Retrovirus-related Pol polyprotein	P10400
AWLQVSPV	8		1767.4	Transmembrane protein 87A	Q8BXN9-1; Q8BXN9-3; Q8BXN9-2
VRYINENL	8		1786.4	T-complex protein 1 subunit alpha	P11983-2; P11983
VVFDFGRRP	9		1827.5	Helicase with zinc finger domain 2	E9QAM5
VGGYFLAGRSM	11	1xOxidation [M11]	1833.2	Sodium/glucose cotransporter 2	Q923I7; Q8VDT1
KVFDFAGEEV	10		1841.9	Craniofacial development protein 1	O88271
VGPTLKATF	9		1850	ATPase family AAA domain-containing protein 2	Q8CDM1-2; Q8CDM1
KGVTYVNSF	9		1897.1	Thioredoxin reductase 3	Q99MD6
AWIPVRML	8		1932	Zinc finger CW-type PWWP domain protein 1	Q6IR42
VNYDVRVF	8		1933.5	Nuclear pore complex protein Nup133	Q8R0G9
TSLKLRKFDSL	11		1947.7	Membrane magnesium transporter 1	Q8K273
IRIVLVGL	8		1949.1	Disintegrin and metalloproteinase domain-containing protein 9	Q61072
ALNEKLVNL	9		1960.8	Eukaryotic translation initiation factor 3 subunit F	Q9DCH4
IHLYPPLTL	9		2019.8	Vacuolar protein sorting-associated protein 13C	Q8BX70-3; Q8BX70-2; Q8BX70
VYIYKEHF	8		2053.4	Small subunit processome component 20 homolog	Q5XG71
EQYIYPTI	8		2060.1	tRNA pseudouridine synthase A, mitochondrial	Q9WU56-2; Q9WU56-3; Q9WU56-4; Q9WU56
ENYDFTKF	8		2175.3	EH domain-containing protein 4	Q9EQP2
SWLHPPPVL	9		2181.6	Phosphatidylinositol N-acetylglucosaminyltransferase subunit Q	Q9QYT7
SIIIFTNTC	9		2182.6	Probable ATP-dependent RNA helicase DDX49	Q4FZF3
IGHRYIEVF	9		2188.9	Heterogeneous nuclear ribonucleoprotein F	Q9Z2X1-2; Q9Z2X1
RGWYLTDL	8		2197.5	Laminin subunit alpha-2	Q60675
SGYQRDGYQQN	12		2203.1	Caprin-1	Q60865
NGYDYGQCRL	10		2217.5	Serine/arginine-rich splicing factor 9	Q9D0B0
MCPEEYPHL	9	1xOxidation [M1]	2223.3	Nck-associated protein 1-like	Q8K1X4
ATHSSRFIPLK	11		2253.5	40S ribosomal protein S15	P62843
SLIILHQL	8		2299.2	Nuclear pore complex protein Nup133	Q8R0G9
QWIVVRTL	8		2317	Solute carrier organic anion transporter family member 4A1	Q8K078-2; Q8K078
SHITFLTIKGAGH	14		2362.3	Lysosomal protective protein	P16675
QGPDYVLV	8		2417.5	Proteasome subunit beta type-2	Q9R1P3
LQQQYNRV	8		2444.8	Talin-1	P26039
RTYFPYGAPC	10		2457.4	Fanconi anemia group C protein homolog	P50652
AFGTVYKGL	9		2532.5	Epidermal growth factor receptor	Q01279
TCVAFSLV	8		2538.1	Myeloid-associated differentiation marker	O35682
RIVELISRV	9		2553.6	TOM1-like protein 2	Q5SRX1-3; Q5SRX1-4; Q5SRX1; Q5SRX1-2
LSGFLRTL	8		2603.6	Ribosomal oxygenase 2	Q8CD15
TIFIRGGNKM	10		2628.3	T-complex protein 1 subunit epsilon	P80316
TIHGLIYNAL	10		2681.4	Serine/threonine-protein phosphatase 2A 56 kDa regulatory subunit gamma isoform	Q60996-2; Q60996-4; Q60996; Q60996-3
ASVLNVNHI	9		2684.9	Ankyrin repeat domain-containing protein 17	Q99NH0-1
YLALYQKL	8		2707.3	E3 ubiquitin-protein ligase UBR4	A2AN08-2; A2AN08-3; A2AN08-5; A2AN08

NYRFKNLF	8		2839.8	G1/S-specific cyclin-E2	Q9Z238
KNQVLTTNI	9		2858.7	Neuronal acetylcholine receptor subunit alpha-7	P49582
KMLLSLKM	8	2xOxidation [M2; M8	2860.3	Tryptophan--tRNA ligase, cytoplasmic	P32921-2; P32921
KRYIHRDL	8		2875.4	Tyrosine-protein kinase JAK2	Q62120
RIIDFKEI	8		2968.5	Chondroitin sulfate synthase 1	Q6ZQ11
TKHFPQFSQI	10		3017.2	ATP-dependent RNA helicase DDX55	Q6ZPL9
TCLKYLVL	8		3020.9	COP9 signalosome complex subunit 2	P61202; P61202-2
KICDFGLARV	10		3025	mitogen-activated protein kinase 1	P63085; O54949
ELFPVFTQL	9		3071.7	Bombesin receptor-activated protein C6orf89 homolog	Q99KU6
RIVVMSQV	8		3126.7	TBCC domain-containing protein 1	Q640P7
VFLERGEVM	9		3131.6	DNA-directed RNA polymerase II subunit RPB1	P08775
KNYEQPTI	8		3148.2	Spermatogenesis-associated protein 6	Q3U6K5
SLVELTSL	8		3155.5	Adenine phosphoribosyltransferase	P08030
KRGWKMFHTL	10		3158.8	PH and SEC7 domain-containing protein 4	Q8BLR5
SCFKLRTV	8		3184.1	Active breakpoint cluster region-related protein	Q5SSL4; Q5SSL4-2; Q5SSL4-4; Q5SSL4-3
RSPDGYLQI	9		3186.1	Alpha-1,3-mannosyl-glycoprotein 4-beta-N-acetylglucosaminyltransferase B	Q812F8
KICDFGLARI	10		3223.3	Mitogen-activated protein kinase 3	Q63844
KMIGGSMLI	9	2xOxidation [M2; M7	3294.1	Synaptic vesicle glycoprotein 2B	Q8BG39
SRYAHWVV	8		3332.8	Transportin-1	Q8BFY9; Q8BFY9-2; Q99LG2
ARIIFNQV	8		3458.5	Bcl-2-related protein A1	Q07440
GYEFIHKL	8		3508.7	Tyrosine--tRNA ligase, mitochondrial	Q8BYL4
SSYDWSKGHEF	11		3520.9	mRNA export factor	Q8C570
RIAELTSQL	9		3529.4	Protein phosphatase 1 regulatory subunit 21	Q3TDD9-2; Q3TDD9
VALTRARYGVI	11		3535.4	Regulator of nonsense transcripts 1	Q9EPU0; Q9EPU0-2
KPMDFFTM	8	2xOxidation [M3; M8	3590.4	Peregrin	B2RRD7
TIVLYILCI	9		3635.5	Transmembrane protein 260	Q8BMD6; Q8BMD6-2
EESFPGFVNL	10		3644.2	Nesprin-1	Q6ZWR6-2; Q6ZWR6-3; Q6ZWR6-4; Q6ZWR6
KRYVESLL	8		3716.4	E3 ubiquitin-protein ligase RNF213	E9Q555
INQDGYPDI	9		3800	Integrin alpha-6	Q61739; Q61739-2
VGHDVSVSV	9		3817.9	Hematopoietic lineage cell-specific protein	P49710
DVPAFRTLV	9		3881.7	Dual specificity testis-specific protein kinase 1	O70146
LSHTQGPSL	9		3890.6	Breast cancer type 2 susceptibility protein homolog	P97929
VTHHYFLRWTR	12		3927.4	C-type lectin domain family 4 member D	Q9Z2H6
AWIKVEQL	8		3940.3	Putative oxidoreductase GLYR1	Q922P9
SFKKGASL	8		3961	SLIT-ROBO Rho GTPase-activating protein 3	Q91Z67; Q812A2; Q91Z69
SLVDLNTRL	9		3966.7	Mitochondrial amidoxime reducing component 2	Q922Q1
TGPEFLVGC	9		3978.7	Histone-lysine N-methyltransferase SETDB1	O88974-4; O88974-7; O88974
NAPEFVKV	8		3980.5	Protein FAM107B	Q3TGF2
KAFHDPSKL	9		3982.4	Zinc finger protein 728	Q6P5C7
IWLHVEGV	8		3988.3	Pyridoxal-dependent decarboxylase domain-containing protein 1	Q99K01-4; Q99K01-5; Q99K01-3; Q99K01; Q99K01-2
ITGYFPSMY	9		4045	Neutrophil cytosol factor 1	Q09014
GCPLYHNL	8		4067.9	Histone acetyltransferase KAT7	Q5SVQ0-4; Q5SVQ0-5; Q5SVQ0-2; Q5SVQ0-3; Q5SVQ0
LAGAGWIAL	9		4113.9	Corticosteroid 11-beta-dehydrogenase isozyme 2	P51661
SGYDMPYA	8		4128.9	Serrate RNA effector molecule homolog	Q99MR6-3; Q99MR6; Q99MR6-4; Q99MR6-2
KCVDFQTL	8		4132.3	DNA replication licensing factor MCM5 OS=Mus musculus OX=10090	P49718
QSLSHPNIV	9		4162.7	Serine/threonine-protein kinase DCLK3	Q8BWQ5
YSAVYAVA	8		4176.7	Taste receptor type 1 member 2	Q925I4
QLLRLFKL	8		4209.2	Two pore calcium channel protein 1	Q9EQJ0
KIVELNTKL	9		4215.5	dystonin	Q91ZU6-3; Q91ZU6-4; Q91ZU6-2; Q91ZU6
IHISKKWGF	9		4252.3	60S ribosomal protein L10-like	P86048; Q6ZWV3
VQKVGFFGI	9		4327.7	Vacuole membrane protein 1	Q99KU0
VGPDFGTKKL	10		4340	DNA-dependent protein kinase catalytic subunit	P97313
KNLYRQLI	8		4384.4	DNA replication licensing factor MCM4	P49717
FQIVNPHLL	9		4427.7	Ribonucleoside-diphosphate reductase large subunit	P07742

SPRFDVQL	8		4532.9	40S ribosomal protein S15a	P62245
ICPKYSNC	8		4614.8	Adhesion G protein-coupled receptor E1	Q61549
SLARLPSL	8		4639.4	Acidic leucine-rich nuclear phosphoprotein 32 family member E	P97822-2; P97822
TRLYPEPSL	9		4723.6	NF-kappa-B inhibitor delta	Q2TB02
ITCPKVNQF	9		4734.8	26S proteasome non-ATPase regulatory subunit 1	Q3TXS7
RIVELEGRV	9		4819.5	Golgi membrane protein 1	Q91XA2
QALMRSTV	8	1xOxidation [M4]	4868.8	Regulator of G-protein signaling 9	O54828-2; O54828
SFLDVRNI	8		4873	26S proteasome non-ATPase regulatory subunit 2	Q8BRH0-2; Q8BRH0; Q8VDM4
SWLDVRHI	8		4877.9	Interleukin-1 receptor-associated kinase 3	Q8K4B2
NSYRYSVP	8		4893.4	Claudin-8	Q9Z260
ACIGFPVL	8		4939.9	Minor histocompatibility antigen H13	Q9D8V0; Q9D8V0-3; Q9D8V0-4
