# Supplementary material for: Palladium-Induced Temporal Internalization of MHC Class I Contributes to T Cell-Mediated Antigenicity
Source: Front Immunol. 2021 Dec 23;12:736936. doi: 10.3389/fimmu.2021.736936 (PMC8732370; doi:10.3389/fimmu.2021.736936)
Supplement: Supplementary file 3 [file Table_2.pdf]

Supplementary Table 2 Peptide list on H-2K^b in the presence of $PdCl_2$

Sequence	Sequence Length	Modification	Affinity (nM)	Protein	Protein Accession
VSYLFSHV	8		1.8	ribose-phosphate pyrophosphokinase 2	Q9CS42; Q9D7G0
MSFQFAHL	8		1.8	Vacuolar protein sorting-associated protein 13B OS=Mus musculus OX=10090	Q80TY5; Q80TY5
LSYSFAHL	8		1.8	NACHT, LRR and PYD domains-containing protein 1b allele 5	A1Z198; Q2LKV2; Q0GKD5; Q2LKU9-2; A1Z198-3; Q2LKV5; Q2LKV2-2; Q0GKD5-2; A1Z198-2; Q2LKW6; Q2LKU9
VTYIFNHL	8		1.9	Transmembrane 6 superfamily member 1	P58749
VSFTYRYL	8		1.9	Vacuolar protein sorting-associated protein 16 homolog	Q920Q4
INYSYINL	8		1.9	Ubiquitin carboxyl-terminal hydrolase 24	B1AY13
FAYRFSNL	8		2	Periodic tryptophan protein 2 homolog	Q8BU03
IAYAFFHL	8		2	Isoform 5 of Suppressor of tumorigenicity 7 protein	Q99M96-5; Q99M96-2; Q99M96-9; Q8K4P7-3; Q99M96-4; Q99M96-8; Q99M96-6; Q8K4P7; Q99M96-3; Q99M96-7; Q99M96-1
SSFVFLNL	8		2	surfeit locus protein 4	Q64310
VVYRFFSRL	9		2	Protein RER1	Q9CQU3
IAFIFNNL	8		2	CCR4-NOT transcription complex subunit 1	Q6ZQ08-2; Q6ZQ08-4; Q6ZQ08
ISYLYNKL	8		2.1	Protein Tob2	Q9JM55; Q61471
SSYSFRHL	8		2.1	Lysosomal thioesterase PPT2	O35448
ISLRFTHL	8		2.1	Phosphatidylinositol 3,4,5-trisphosphate 5-phosphatase 2	Q6P549
VSYQFPKL	8		2.1	Suppression of tumorigenicity 5 protein	Q924W7-2; Q924W7; Q924W7-3
TSYRFLAL	8		2.1	HAUS augmin-like complex subunit 3	Q8QZX2; Q8QZX2-2
ASYLFRGL	8		2.1	Transmembrane channel-like protein 6	Q7TN60; Q7TN60-2; Q7TN60-3
ISFEFRSL	8		2.2	Coenzyme Q-binding protein COQ10 homolog B, mitochondrial	Q3THF9-1; Q3THF9-2
AIYAFSHL	8		2.2	ATP-binding cassette sub-family B member 9	Q9JJ59-1; Q9JJ59-2
SSYKFNHL	8		2.3	protein farnesyltransferase subunit beta	Q8K2I1
VVYSYHYL	8		2.3	Tfiih basal transcription factor complex helicase xpd subunit	O08811
VQYKFSHL	8		2.3	Histone acetyltransferase KAT2B	Q9JHD1; Q9JHD2
SNFTFSHL	8		2.3	Protein dispatched homolog 1	Q3TDN0-1
VSLWFRHL	8		2.3	Fanconi anemia group C protein homolog	P50652
VTYLFKQL	8		2.3	exportin-7	Q9EPK7-2; Q9EPK7
TSFRYSSL	8		2.3	Fibronectin type-III domain-containing protein 3A	Q8BX90
TAYEFAKL	8		2.4	Estradiol 17-beta-dehydrogenase 11	Q9EQ06-2; Q9EQ06
MSYLFRNI	8		2.4	Phosphoribosyl pyrophosphate synthase-associated protein 2	Q8R574
SNYLFTKL	8		2.4	endothelial PAS domain-containing protein 1	P97481
KSFLFSAL	8		2.4	Elongation of very long chain fatty acids protein 6	Q920L5
KSYLFQLL	8		2.4	cyclin-dependent kinase 2	P97377-2; P97377-1
FSYNFSSL	8		2.4	Isoform B of Calcipressin-1	Q9JHG6-2
YSYSFFHL	8		2.4	Serine incorporator 3	Q9QZI9
SAYRFSGV	8		2.5	protein elys	Q8CJF7
RAYLFAHV	8		2.5	Pre-rRNA-processing protein TSR1 homolog	Q5SWD9-1; Q5SWD9-2; Q5SWD9-3
VSLKYAHM	8		2.5	adenylosuccinate synthetase isozyme 2	P46664
AAYRYPYL	8		2.5	Protein O-glucosyltransferase 3 OS=Mus musculus OX=10090	G5E897
VSIQFYHL	8		2.5	Vacuolar protein sorting-associated protein 13a	Q5H8C4; Q5H8C4-2
ISLEFRNL	8		2.5	squalene synthase	P53798
SGFVFTRL	8		2.5	Helicase-like transcription factor	Q6PCN7
VSYEKFSNL	9		2.5	TBC1 domain family member 9B	Q5SVR0; Q5SVR0-2
SGYKFFSL	8		2.6	WD repeat domain phosphoinositide-interacting protein 2	Q80W47
IIPMFSNL	8		2.6	erine/threonine-protein phosphatase 2A 65 kDa regulatory subunit A alpha isoforn	Q76MZ3
VVYIYHSL	8		2.6	ribosomal oxygenase 2	Q8CD15
VAYKFPEL	8		2.6	E3 ubiquitin-protein ligase UBR2	Q6WKZ8-1; Q6WKZ8-3; Q6WKZ8-2
SSYMHFTNV	9		2.6	Tensin-3	Q5SSZ5-1
SSLDFYANV	9		2.6	sialoadhesin	Q62230-3; Q62230
LTYTFSGL	8		2.6	DNA oxidative demethylase ALKBH2	Q6P6J4

ASYEFTTL	8	2.6	Developmentally-regulated GTP-binding protein 2	Q9QXB9
QSYEFFHL	8	2.6	Nucleotide-binding oligomerization domain-containing protein 1	Q8BHB0
SNYVFVFL	8	2.6	Prenylated Rab acceptor protein 1	Q9Z0S9
RSYRFPKL	8	2.7	Insulin-like growth factor 1 receptor	Q60751
TNYRFKNL	8	2.7	G1/S-specific cyclin-E2	Q9Z238
ISFKFDHL	8	2.7	F-actin-capping protein subunit alpha-1	P47753
SSYNYIRV	8	2.7	Unconventional myosin-Id	Q5SYD0-2; Q5SYD0
KVYLYTHL	8	2.7	Uncharacterized protein KIAA0513	Q8R0A7
HVYYFAHL	8	2.7	Interferon-induced very large GTPase 1	Q80SU7
IAYLYDRL	8	2.7	DNA replication complex GINS protein PSF1	Q9CZ15
VSYKYFSRI	9	2.7	Urokinase-type plasminogen activator	P06869
SSIVFAEL	8	2.7	Eukaryotic translation initiation factor 2-alpha kinase 1	Q9Z2R9
STFTFADL	8	2.7	E3 SUMO-protein ligase RanBP2	Q9ERU9
VNLLFSGL	8	2.7	High affinity copper uptake protein 1	Q8K211
TTYKYFAL	8	2.7	Terminal uridylyltransferase 7	Q5BLK4
SNYKFFLL	8	2.7	Palmitoyltransferase ZDHHC2	P59267
SALTFAGL	8	2.8	Succinate dehydrogenase [ubiquinone] cytochrome b small subunit, mitochondrial	Q9CXV1
ISVRFHNL	8	2.9	Zinc finger SWIM domain-containing protein 3	Q8CFL8
TNYKFFML	8	2.9	Probable palmitoyltransferase ZDHHC20	Q5Y5T1-2; Q5Y5T1
RTYSFLNL	8	2.9	Transmembrane protein 39B	Q810L4
RNFIFSRL	8	2.9	Lymphocyte-specific helicase	Q60848-2; Q60848-1
VGYRYETL	8	2.9	AMP deaminase 2	Q9DBT5
SSFVFSTV	8	2.9	3-hydroxy-3-methylglutaryl-coenzyme A reductase	Q01237
ASFIFRQL	8	2.9	PAS domain-containing serine/threonine-protein kinase	Q8CEE6
VNFTYQFL	8	2.9	WASH complex subunit 4	Q3UMB9
VNYDYSTL	8	2.9	Zinc finger BED domain-containing protein 6	D2EAC2; D2EAC2-2
HGYTFANL	8	3	SUMO-activating enzyme subunit 1	Q9R1T2; Q9R1T2-2
RNYRYFYL	8	3	Palmitoyltransferase ZDHHC9	P59268
SAYLFVKL	8	3	PDZ domain-containing protein 8 OS=Mus musculus OX=10090	B9EJ80
VVFFFTRL	8	3	Protein BTG3	P50615
ATFPFAML	8	3	Sodium- and chloride-dependent taurine transporter	O35316
IRYIFAYL	8	3.1	Hematopoietic prostaglandin D synthase	Q9JHF7
ATYIFNGL	8	3.1	Coatomer subunit gamma-2	Q9QXK3; Q9QXK3-4
QSFFFTHL	8	3.1	Cytoplasmic FMR1-interacting protein 2	Q5SQX6
SSYRFVQNV	9	3.1	Forkhead box protein K1	P42128
VALEFTHL	8	3.1	p53-induced death domain-containing protein 1	Q9ERV7
SSYVHSNL	8	3.2	sorting nexin-19	Q6P4T1
VTFIYQKL	8	3.2	Coiled-coil domain-containing protein 159	Q8C963-2; Q8C963-1
STYLFRLM	8	3.2	ubiquitin carboxyl-terminal hydrolase 30	Q3UN04
TAFVFPRL	8	3.2	Olfactomedin-like protein 3	Q8BK62
IVPLFTNL	8	3.2	Serine/threonine-protein phosphatase 2A 65 kDa regulatory subunit A beta isoform	Q7TNP2
IGWRYSLL	8	3.2	Monocarboxylate transporter 7	B1AT66; B1AT66-2
ISRTFPNL	8	3.2	TBC1 domain family member 14	Q8CGA2
VIYWFRQI	8	3.3	Uncharacterized protein C20orf24 homolog	Q9CQT9
TSFMFQRV	8	3.3	annexin A4	P97429
RSFLHARL	8	3.3	26S proteasome non-ATPase regulatory subunit 3	P14685
TAFRFSEL	8	3.3	TBC1 domain family member 8B	A3KGB4
VNYRHLAL	8	3.3	DNA-directed RNA polymerase II subunit RPB1	P08775
SALRFLNL	8	3.3	Serine/threonine-protein kinase 11-interacting protein	Q3TAA7
KIYYFAAV	8	3.3	Tyrosine-protein phosphatase non-receptor type 23	Q6PB44-1; Q6PB44-2
AAYEFTTL	8	3.3	Developmentally-regulated GTP-binding protein 1	P32233
SQYRFCQL	8	3.3	Exocyst complex component 6	Q8R313
SAFSFRTL	8	3.3	F-box only protein 38	Q8BMI0
VTFSFKQL	8	3.3	Phosphatidylinositol 3,4,5-trisphosphate-dependent Rac exchanger 1 protein	Q69ZK0; Q69ZK0-2

VTYLFHIL	8	3.3	Stimulated by retinoic acid gene 6 protein-like	Q9DBN1; Q9DBN1-2
VAYRYEVL	8	3.4	Dual specificity tyrosine-phosphorylation-regulated kinase 2	Q5U4C9
SSYTFPKM	8	3.4	Enoyl-CoA delta isomerase 2, mitochondrial	Q9WUR2; Q9WUR2-2
QNYRFYRGM	9	3.4	GC-rich sequence DNA-binding factor 2	Q8BKT3-2; Q8BKT3
KSLTYYTNL	9	3.4	CSC1-like protein 1	Q91YT8
INLIFRYL	8	3.4	Small nuclear ribonucleoprotein E	P62305
SALIYSNL	8	3.4	Trafficking protein particle complex subunit 3	O55013
SNFHFAVL	8	3.4	Vacuolar protein sorting-associated protein 13B	Q80TY5
VIYPFMQGL	9	3.5	DENN domain-containing protein 2D	Q91VV4; Q91VV4-2
SSYTFPQI	8	3.5	Chromodomain Y-like protein 2	Q9D5D8
ISYRLPGL	8	3.5	E3 ubiquitin-protein ligase RNF213	E9Q555
VSPYFVAL	8	3.5	X-ray repair cross-complementing protein 6	P23475
RAYRYLQL	8	3.6	Cytochrome c oxidase assembly protein COX14	Q8BH51
VNFVHTNL	8	3.6	60S ribosomal protein L4	Q9D8E6
SALVFTRL	8	3.6	Glycosylated lysosomal membrane protein	Q9JHJ3
SGYDFSRL	8	3.6	Sterol regulatory element-binding protein cleavage-activating protein	Q6GQT6
VALLFRQL	8	3.6	Myotubularin-related protein 5	Q6ZPE2-2; Q6ZPE2
FTFQFNNL	8	3.6	Ubiquitin carboxyl-terminal hydrolase 15	Q8R5H1; Q8R5H1-2; Q8R5H1-5
ISILYHQL	8	3.7	Protein AAR2 homolog	Q9D2V5
RNYEYCRL	8	3.7	[illegible]-related matrix-associated actin-dependent regulator of chromatin subfamily A m[illegible]	Q91ZW3
ITFIFKSL	8	3.7	Serine/threonine-protein kinase mTOR	Q9JLN9
SSYAYTKV	8	3.7	Methylcytosine dioxygenase TET3	Q8BG87
AVYTYLRL	8	3.7	Integrator complex subunit 3	Q7TPD0-2; Q7TPD0
VNYRHLALL	9	3.7	DNA-directed RNA polymerase II subunit RPB1	P08775
TIYRFLKL	8	3.7	Protein fem-1 homolog C	Q8CEF1
VGFRFPIL	8	3.7	RAS guanyl-releasing protein 2	Q9QUG9; Q9QUG9-2
AVYAFLGL	8	3.7	SH3 domain-binding glutamic acid-rich-like protein	Q9JJU8
ANFTFPRL	8	3.7	Sorting nexin-27	Q3UHD6; Q3UHD6-2
KGFYFAKL	8	3.8	E3 SUMO-protein ligase RanBP2	Q9ERU9
SQYRFEHL	8	3.8	GTP-binding protein 2	Q3UJK4
VAYGFRNI	8	3.8	Cytosolic fe-s cluster assembly factor narfl	Q7TMW6-2; Q7TMW6-1
TQYSFYQQL	9	3.8	Inositol 1,4,5-trisphosphate receptor type 2	Q9Z329; Q9Z329-3; Q9Z329-2
FSLRFNNL	8	3.8	Toll-like receptor 13	Q6R5N8
FTYRYLAL	8	3.8	Vacuolar protein sorting-associated protein 16 homolog	Q920Q4
RNYRYFFL	8	3.9	Probable palmitoyltransferase ZDHHC8	Q8VDZ4; Q8VDZ4-2; Q5Y5T5
VTYVYQLI	8	3.9	Vacuolar protein sorting-associated protein 29	Q9QZ88; Q9QZ88-2
VNYDFGHM	8	3.9	E3 ubiquitin-protein ligase RNF216	P58283-2; P58283
SNYHFYSSI	9	3.9	Nuclear factor erythroid 2-related factor 2	Q60795
IGPTYYQRL	9	3.9	DNA-directed RNA polymerase II subunit RPB2	Q8CFI7
ITYAWTRL	8	3.9	[illegible]robable dolichyl pyrophosphate Glc1Man9GlcNAc2 alpha-1,3-glucosyltransfera[illegible]	Q6P8H8
NNYVYAGL	8	3.9	E3 ubiquitin-protein ligase RFWD3	Q8CIK8
KVYNYNHL	8	4	60S ribosomal protein L27	P61358
RSFDFIHL	8	4	TRMT1-like protein	A2RSY6; A2RSY6-2
RQYMFSSL	8	4	X-ray repair cross-complementing protein 5	P27641
LQWRFANL	8	4	Tether containing UBX domain for GLUT4	Q8VBT9
SSMRYVLL	8	4	Alpha-L-iduronidase	P48441
RSWLFQHL	8	4	Homeobox protein PKNOX2	Q8BG99
RGYLYGQL	8	4	Isoform PLEC-1G of Plectin	Q9QXS1-14
AFFRFSGL	8	4	StAR-related lipid transfer protein 3	Q61542
AIFNFQSL	8	4	Protein kish-A	Q9CR64; Q9CR64-2
SGLLFTHL	8	4.1	Galactosylgalactosylxylosylprotein 3-beta-glucuronosyltransferase 3	P58158
IVIKFRYL	8	4.1	Protein dispatched homolog 1	Q3TDN0-1
SVYKFFDL	8	4.1	Retinoblastoma-associated protein	P13405
RTYIFTFL	8	4.2	Ras-GEF domain-containing family member 1B	Q8JZL7-2; Q8JZL7

KGFTFSAL	8		4.2	transmembrane protein 135	Q9CYV5
KAFTYINL	8		4.2	Transferrin receptor protein 1	Q62351
SAVVYAQL	8		4.2	5'-3' exoribonuclease 1	P97789-2; P97789-3; P97789-1
VGYNPYSHL	9		4.2	F-related matrix-associated actin-dependent regulator of chromatin subfamily E m	O54941
ASYEFVQRL	9		4.2	Cytoplasmic dynein 1 heavy chain 1	Q9JHU4
IALRYVAL	8		4.2	Coatomer subunit beta	Q9JIF7
TSYIFVSV	8		4.2	Phosphatidylinositol 4,5-bisphosphate 3-kinase catalytic subunit alpha isoform	P42337
TILSFTNL	8		4.2	Sodium-coupled neutral amino acid transporter 2	Q8CFE6
VKYLFTGL	8		4.2	Ubiquitin carboxyl-terminal hydrolase 14	Q9JMA1
KSFDYGNL	8		4.3	Transcription factor HIVEP2	Q3UHF7
ISFEFRSLL	9		4.3	Coenzyme Q-binding protein COQ10 homolog B, mitochondrial	Q3THF9; Q3THF9-2
TILEFAQL	8		4.3	UDP-glucuronic acid decarboxylase 1	Q91XL3
ISLDYQHL	8		4.4	Protein KIAA0100	Q5SYL3
SGYQYKRL	8		4.4	Histone-lysine N-methyltransferase SETDB1	O88974-4; O88974-1
VVYIYRQI	8		4.4	Sorbin and SH3 domain-containing protein 1	Q62417-3; Q62417-5; Q62417-2; Q62417-4; Q62417; Q62417-7; Q62417-6
VGLRYYTGV	9		4.4	Helicase-like transcription factor	Q6PCN7
KSYSFIARM	9		4.4	Calcium homeostasis endoplasmic reticulum protein	Q8CGZ0
SSLHFSFL	8		4.4	DnaJ homolog subfamily C member 16	Q80TN4
ASYVYLSM	8		4.4	Ferritin heavy chain	P09528; Q9D5H4
VTWGFPNL	8		4.4	60S ribosomal protein L7-like 1	Q9D8M4
SAYEYLEL	8		4.4	Sodium-dependent multivitamin transporter	Q5U4D8
ANYDFYQL	8		4.4	Protein SMG8	Q8VE18
AVYSFEAL	8		4.4	von Willebrand factor A domain-containing protein 5A	Q99KC8
TAYAFHFL	8		4.5	Peroxisomal acyl-coenzyme A oxidase 1	Q9R0H0-1; Q9R0H0-2
IAMEFNHL	8		4.5	Prostaglandin G/H synthase 1	P22437
SQYLFPKL	8		4.5	Peroxisome proliferator-activated receptor delta	P35396
VGMKYRNL	8		4.5	Neurolysin, mitochondrial	Q91YP2
TIYKFQGM	8		4.5	general transcription factor 3C polypeptide 5	Q8R2T8-2; Q8R2T8
SGYSFTHI	8		4.5	Actin-related protein 6	Q9D864
SSYSFRHLL	9		4.5	Lysosomal thioesterase PPT2	O35448
VAVIFSGL	8		4.5	RNA polymerase II subunit A C-terminal domain phosphatase	Q7TSG2
VAFAFKKL	8		4.6	Interleukin-1 receptor-associated kinase-like 2	Q8CFA1-1; Q8CFA1-4; Q8CFA1-3; Q8CFA1-2
KVMPFANL	8		4.6	DNA-directed RNA polymerase III subunit RPC5	Q9CZT4-2; Q9CZT4-1
SGYKFGVL	8		4.6	General transcription factor IIE subunit 2	Q9D902
RSYRFMVM	8		4.6	serine/threonine-protein kinase VRK2	Q8BN21; Q8BN21-2; Q8BN21-3
YAYSFKYL	8		4.6	transcription elongation factor spt6	Q62383
SVYTHSYL	8		4.6	Ectonucleoside triphosphate diphosphohydrolase 8	Q3TZX8-3; Q3TZX8-2; Q8K0L2-2; Q8K0L2; Q3TZX8
VILEYFTRL	9		4.6	Ribonuclease P protein subunit p29	Q9CR08
IVLTFRQL	8		4.6	Solute carrier family 25 member 44	Q8BGF9
VSPLFQKL	8		4.7	Methionine--tRNA ligase, cytoplasmic	Q68FL6
SIYRFHAQF	9		4.7	FYVE, RhoGEF and PH domain-containing protein 2	Q8BY35
SNWWFVAHL	9		4.7	Nuclear pore complex protein Nup85	Q8R480
RAFGFSHL	8		4.7	Ubiquitin thioesterase OTUB1	Q7TQI3
LQYIFAHV	8		4.7	Pre-mRNA-processing-splicing factor 8	Q99PV0
VIISFNSL	8		4.7	Putative sodium-coupled neutral amino acid transporter 10	Q5I012-3; Q5I012; Q5I012-2; Q5I012-4
IHYFFSKL	8		4.7	Transmembrane 9 superfamily member 2	P58021
SGYKYVGM	8	1xOxidation [M8]	4.8	protein YIF1A	Q91XB7
LQYEFTHL	8		4.8	formin-like protein 1	Q9JL26-2; Q9JL26
SGYIYHKL	8		4.8	Regulator of nonsense transcripts 1	Q9EPU0-1; Q9EPU0-2
STLLFAHI	8		4.8	anaphase-promoting complex subunit 1	P53995
VAYEYLCHL	9		4.8	Ras GTPase-activating-like protein IQGAP1	Q9JKF1
SSAIYMNL	8		4.8	Transcriptional repressor p66-beta	Q8VHR5-2; Q8VHR5
SIWLFQANL	9		4.8	Bax inhibitor 1	Q9D2C7

RGYLYQTL	8		4.9	Serine/threonine-protein kinase Kist	P97343
QAYDFYYL	8		4.9	Tyrosine--tRNA ligase, mitochondrial	Q8BYL4
HAFMFAKL	8		5	Endosomal/lysomomal potassium channel TMEM175	Q9CXY1
VNYYFERNM	9		5	PHD finger protein 14	Q9D4H9; Q9D4H9-3; Q9D4H9-2
VNIPFVRL	8		5	NFX1-type zinc finger-containing protein 1	Q8R151
TSLKYMLL	8	1xOxidation [M6]	5.1	26S proteasome non-ATPase regulatory subunit 11	Q8BG32
ISVQFNQL	8		5.1	Integrator complex subunit 8	Q80V86
SNISFLHL	8		5.1	UPF0489 protein C5orf22 homolog	Q8BGC1; Q8BGC1-3
QIIAFFAHL	9		5.2	E1A-binding protein p400	Q8CHI8-4; Q8CHI8-3; Q8CHI8-2; Q8CHI8-5; Q8CHI8
KIFMFQLL	8		5.2	Cyclin-dependent kinase 18	Q04899
STYDFMSTL	9		5.2	Beta-hexosaminidase subunit alpha	P29416
QNPVYAPL	8		5.2	Heme oxygenase 1	P14901
ITYGQFAQL	9		5.2	1-phosphatidylinositol 4,5-bisphosphate phosphodiesterase gamma-1	Q62077
TSVRFTQL	8		5.3	nitric oxide synthase-interacting protein	Q9D6T0-1
SSFRMRHL	8		5.3	Microtubule-actin cross-linking factor 1	Q9QXZ0-3; Q9QXZ0-2; Q9QXZ0-4; Q9QXZ0
RAFAFTNV	8		5.3	Large neutral amino acids transporter small subunit 4	Q8CGA3
SSYNYRVV	8		5.4	Inositol 1,4,5-trisphosphate receptor type 1	P11881-7; P11881-2; P11881-8; P11881-5; P11881-3; P11881-4; P11881-6; P11881-1
STVEFTNL	8		5.4	Centromere/kinetochore protein zw10 homolog	O54692
IAFGFHQL	8		5.4	Peroxisomal membrane protein PEX14	Q9R0A0
KGYRFIFL	8		5.4	Vacuolar protein sorting-associated protein 33B	P59016
QSIEFSRL	8		5.5	Eukaryotic translation initiation factor 3 subunit A	P23116
KNYDFAQV	8		5.5	PAB-dependent poly(A)-specific ribonuclease subunit PAN2	Q8BGF7-2; Q8BGF7; Q8BGF7-3
SNYLYREV	8		5.5	Xylosyltransferase 2	Q9EPL0
VQFLYREL	8		5.5	Dynein assembly factor 5, axonemal	B9EJR8
TNYRFPSSL	9		5.5	UDP-glucuronic acid/UDP-N-acetylgalactosamine transporter	A2AKQ0; A2AKQ0-2
SAFIFRVL	8		5.5	Chloride channel protein 2	Q9R0A1
ASLRYLGL	8		5.5	Leucine-rich repeat-containing protein 14	Q8VC16
SIHSFQNL	8		5.6	Transcription factor p65	Q04207-1; Q04207-2
NAYKFPNL	8		5.6	Aldehyde oxidase 1	O54754
SAFEFNEL	8		5.6	Myotubularin-related protein 1	Q9Z2C4
QVFIFTGL	8		5.6	Metalloendopeptidase OMA1, mitochondrial	Q9D8H7
SSLLFVKL	8		5.6	Ribonuclease H2 subunit B	Q80ZV0
RNFLYLRL	8		5.6	Probable global transcription activator SNF2L2	Q6DIC0
ISYRYLII	8		5.6	Sulfide:quinone oxidoreductase, mitochondrial	Q9R112
FVYVFHTL	8		5.7	Transmembrane glycoprotein NMB	Q99P91
RSLKFYSL	8		5.7	Pre-mRNA-processing factor 19	Q99KP6-1; Q99KP6-2; Q99KP6-3
SNLQYSLL	8		5.7	Multidrug resistance-associated protein 5	Q9R1X5
SSLEFTEL	8		5.7	Caspase-6	O08738
TAFQFLQL	8		5.7	Cyclin-G1	P51945
VAPEFRRL	8		5.7	N-alpha-acetyltransferase 20	P61600
KAFWYGQL	8		5.8	zinc transporter zip11	Q8BWY7-2; Q8BWY7-1; Q8BWY7-3
VSYNHTNI	8		5.8	Uncharacterized protein CXorf23 homolog	A2AG58-2; A2AG58
RAFDYFNL	8		5.8	Protein sel-1 homolog 1	Q9Z2G6-1; Q9Z2G6-2
FTFEYRYL	8		5.8	CCR4-NOT transcription complex subunit 3	Q8K0V4
LIYKFLNV	8		5.8	Exportin-1	Q6P5F9
STIHFYSL	8		5.8	Protein transport protein Sec24A	Q3U2P1; Q3U2P1-2
SMILFTSL	8		5.8	Ataxin-10	P28658
KTFIFVRL	8		5.8	DNA helicase B	Q6NVF4
AVIHFAGL	8		5.9	UDP-glucose 4-epimerase	Q8R059
SPYKFRNL	8		5.9	5-phosphohydroxy-L-lysine phospho-lyase	Q8R1K4-2; Q8R1K4; Q8R1K4-3
SAARFALL	8		5.9	Solute carrier family 12 member 6	Q924N4; Q924N4-2
VLYQFRGV	8		5.9	DnaJ homolog subfamily C member 11	Q5U458
ISYNFIEKL	9		5.9	Dynein light chain 1, axonemal	Q05A62; Q05A62-2; Q05A62-3

VIFIFTKV	8		5.9	Glycosaminoglycan xylosylkinase	Q8VCS3
VIFEMTNL	8		5.9	Insulin-like growth factor 1 receptor	Q60751
ISVSFYHV	8		6	Eukaryotic translation initiation factor 3 subunit B	Q8JZQ9
VLYAHFRHL	9		6	Cytoplasmic tRNA 2-thiolation protein 1	Q99J10
SNLVFLGL	8		6	Zinc finger protein 728	Q6P5C7
KTWRFSNM	8		6.1	Fermitin family homolog 3	Q8K1B8; Q8CIB5
VMYKFLTV	8		6.1	Caveolin-2	Q9WVC3
ANYNFRGM	8		6.1	Double-stranded RNA-binding protein Staufen homolog 2	Q8CJ67-3; Q8CJ67-2; Q8CJ67-1
KTYHYLYL	8		6.1	Protein fem-1 homolog B	Q9Z2G0
STYSHSAL	8		6.1	transcriptional regulator ATRX	Q61687
VAVKFVRL	8		6.1	T-complex protein 11-like protein 1	Q8BTG3
SSLKFLLL	8		6.1	ATP-dependent DNA helicase DDX11	Q6AXC6
SRYQFRNL	8		6.2	Peptidyl-tRNA hydrolase ICT1, mitochondrial	Q8R035-2; Q8R035-1
INFDFPKL	8		6.2	Probable ATP-dependent RNA helicase DDX6	P54823
TNINFPNL	8		6.2	Histone-lysine N-methyltransferase 2C	Q8BRH4-2; Q8BRH4-1
ATFHFRTL	8		6.2	AH receptor-interacting protein	O08915
QGYTFLQL	8		6.2	Lysophosphatidylcholine acyltransferase 2	Q8BYI6-2; Q8BYI6
RSPFFRNL	8		6.3	BTB/POZ domain-containing protein 7	Q8CFE5-2; Q8CFE5-3; Q8CFE5-1
YQFVYQNL	8		6.3	Up-regulator of cell proliferation	Q5NCI0-2; Q5NCI0-1
VAYWRQAGL	9		6.3	ATP synthase subunit epsilon, mitochondrial	P56382
VAFEYCQRL	9		6.3	AP-5 complex subunit zeta-1	Q3U829
SIYARFVQL	9		6.3	Polyphosphoinositide phosphatase	Q91WF7
TNYIFDSL	8		6.3	TBC1 domain family member 15	Q9CXF4
HGYIFSSL	8		6.4	Exosome complex component csl4	Q9DAA6-2; Q9DAA6-1
SALKYYQL	8		6.4	KIF1-binding protein	Q6ZPU9-1; Q6ZPU9-3
RSYSFQKV	8		6.4	DIS3-like exonuclease 2	Q8CI75-2; Q8CI75
TQYIFNNM	8		6.4	Alpha-mannosidase 2	P27046
SNLRYLSL	8		6.4	Toll-like receptor 3	Q99MB1
SQFKYALV	8		6.4	Developmentally-regulated GTP-binding protein 2	Q9QXB9
ISARFVQL	8		6.4	Cytoplasmic polyadenylation element-binding protein 2	Q812E0; Q7TN98-5; Q7TN99-2; Q7TN98-1; Q7TN99-4; Q7TN98-2; Q7TN99-6; Q7TN99-3; Q7TN98-3; Q7TN99; Q7TN98-4
VLYVWAQL	8		6.4	Derlin-1	Q99J56
SAPLFTGL	8		6.4	Nuclear envelope pore membrane protein POM 121	Q8K3Z9
IALFFRSL	8		6.4	Transcription factor 25	Q8R3L2; Q8R3L2-4; Q8R3L2-2; Q8R3L2-3; Q8R3L2-5
STYKFFEV	8		6.5	60S ribosomal protein L15	Q9CZM2
TAFKFKAL	8		6.5	Neurofibromin	Q04690-3; Q04690; Q04690-4; Q04690-2
VSFDYHQM	8		6.5	Synaptojanin-1	Q8CHC4
ANINFPNL	8		6.5	Histone-lysine N-methyltransferase 2D	Q6PDK2
VVFFFKTL	8		6.5	Selenoprotein K	Q9JLJ1
RNYLHYSL	8		6.6	26S proteasome non-ATPase regulatory subunit 3	P14685
TNVEYAHL	8		6.6	Acyl-CoA dehydrogenase family member 10	Q8K370
RNFVFHTL	8		6.6	Claspin	Q80YR7
ANYCFASRL	9	Carbamidomethyl [C	6.6	Poly [ADP-ribose] polymerase 2	O88554
SSLKFYLRNL	10		6.6	Oligophrenin-1	Q99J31
GNYRFMKL	8		6.6	Inversin	O89019-7; O89019-3; O89019-6; O89019; O89019-4; O89019-2; O89019-5
TSLRFVFL	8		6.6	Reticulophagy regulator 3	Q9CQV4
QAYAFLQYL	9		6.6	Dolichyl-diphosphooligosaccharide--protein glycosyltransferase subunit STT3B	Q3TDQ1
SVYVYKVL	8		6.7	Histone H2B type 2-B	Q64475; Q8CGP2; Q64525; Q8CGP1; P10854; Q64478; Q8CGP2-2; Q6ZWY9; P10853
VTHKYVHL	8		6.7	Zinc finger protein 865	Q3U3I9
VTPRFPKL	8		6.7	Activating signal cointegrator 1 complex subunit 3	E9PZJ8-2; E9PZJ8-1
RGYDFAAV	8		6.8	EH domain-containing protein 1	Q9QXY6; Q9WVK4
RQYIFSKL	8		6.8	Interferon-induced very large GTPase 1	Q80SU7

VGPRYTQL	8		6.8	mitogen-activated protein kinase 3	Q63844
TGYNFQRV	8		6.8	Pleiotropic regulator 1	Q922V4
YTYLYVRM	8		6.8	Activating signal cointegrator 1 complex subunit 3	E9PZJ8-1
VGYRFVTAI	9		6.8	Nischarin	Q80TM9-3; Q80TM9-2; Q80TM9-1
ATLVFHNL	8		6.9	Signal transducer and activator of transcription 3	P42227-3; P42227; P42227-2
STFFYPKL	8		6.9	Sentrin-specific protease 2	Q91ZX6-3; Q91ZX6-2; Q91ZX6
RAYLFNSV	8		7	Protein yippee-like 1	Q9ESC7; Q65Z93; P61237; Q65Z95
VNFSPANL	8		7	HAUS augmin-like complex subunit 1	Q8BHX1
TNYNFQYI	8		7	glycerol-3-phosphate acyltransferase 4	Q8K2C8
QTMVFYNL	8		7	Proteasome activator complex subunit 4	Q5SSW2-1
STFVYNSM	8		7	Guanylate-binding protein 4	Q61107
YAYLYIRM	8		7	U5 small nuclear ribonucleoprotein 200 kDa helicase	Q6P4T2
SVIKFENL	8		7.1	Isoform 2 of Palmitoyltransferase ZDHHC6	Q9CPV7-3; Q9CPV7-2; Q9CPV7; Q9CPV7-4
SSFAHAQV	8		7.1	protein O-GlcNAcase	Q9EQQ9-1; Q9EQQ9-3
THYSFLATL	9		7.1	Major facilitator superfamily domain-containing protein 3	Q5U419
VTYHGFPNL	9		7.1	N-glycosylase/DNA lyase	O08760
RIYSFQMAL	9		7.1	Cyclin-dependent kinase 6	Q64261
VTHWYENL	8		7.1	Ubiquitin carboxyl-terminal hydrolase 34	Q6ZQ93; Q6ZQ93-3; Q6ZQ93-4; Q6ZQ93-2
SSFHFNQHL	9		7.2	Codanin-1	Q8CC12-2; Q8CC12-3; Q8CC12-1
SILAFHYL	8		7.2	Cadherin EGF LAG seven-pass G-type receptor 3	Q91ZI0
RSIWFQQL	8		7.2	Motile sperm domain-containing protein 2	Q9CWP6
TSAHFARL	8		7.3	ankyrin repeat domain-containing protein 13A	Q80UP5
SGYHYVCL	8		7.3	1-phosphatidylinositol 4,5-bisphosphate phosphodiesterase beta-3	P51432
VGLHFLTNL	9		7.4	Leucine-rich repeat and coiled-coil domain-containing protein 1	Q69ZB0-3; Q69ZB0-2; Q69ZB0
VAPLYKRL	8		7.4	Methylcytosine dioxygenase TET3	Q8BG87; Q8BG87-4
KIITYRNL	8		7.5	PCI domain-containing protein 2	Q8BFV2
VIQVFQQL	8		7.5	dephospho-CoA kinase domain-containing protein	Q8BHC4
RGYIFSLV	8		7.5	Dedicator of cytokinesis protein 6	Q8VDR9-1
VSFPFGKI	8		7.5	Protein RRP5 homolog	Q6NS46
RNLDYARL	8		7.6	Serine hydroxymethyltransferase, cytosolic	P50431
RGFKYLRL	8		7.6	Transcription activator BRG1	Q3TKT4-1; Q3TKT4-2
VNRSFIAL	8		7.6	Receptor expression-enhancing protein 5	Q60870
ATYTFIQQL	9		7.6	galactokinase	Q9R0N0
EAYRFTGL	8		7.6	Centromere protein O	Q8K015; Q8K015-2
VAPSFGTL	8		7.6	Nucleoporin NUP188 homolog	Q6ZQH8
KGYIFLTL	8		7.6	E3 ubiquitin-protein ligase TRIM56	Q80VI1
SNTQYARL	8		7.7	Very long-chain specific acyl-CoA dehydrogenase, mitochondrial	P50544
FAYRFSNLL	9		7.7	Periodic tryptophan protein 2 homolog	Q8BU03
STFSFTKV	8		7.7	Extended synaptotagmin-2	Q3TZZ7
SNFEYLMQL	9		7.7	Neurobeachin-like protein 2	Q6ZQA0; Q6ZQA0-2
SSFLFWRM	8		7.8	protein AF1q	P97783
IQWAFKNL	8		7.8	Ubiquitin carboxyl-terminal hydrolase 34	Q6ZQ93-1; Q6ZQ93-3; Q6ZQ93-2
SQYVFTEM	8	1xOxidation [M8]	7.8	Transmembrane protein 199	Q5SYH2
VGFTFPNRL	9		7.8	Neurochondrin	Q9Z0E0-2; Q9Z0E0-1
YNFQYISL	8		7.8	Glycerol-3-phosphate acyltransferase 4	Q8K2C8
FSPRFVAL	8		7.8	E3 ubiquitin-protein ligase synoviolin	Q9DBY1-2; Q9DBY1
KNVLFSHL	8		7.9	cAMP-dependent protein kinase type I-beta regulatory subunit	P12849; Q9DBC7
RSLRFVTL	8		7.9	Lysosomal acid phosphatase	P24638
HIYEFPQL	8		7.9	Protein unc-119 homolog B	Q8C4B4
IIYKFVKNV	9		7.9	Vacuolar protein sorting-associated protein 13C	Q8BX70-3; Q8BX70-2; Q8BX70-1
SIWEFKYL	8		7.9	Shieldin complex subunit 2	Q3UEN2
ANHRYANV	8		8.1	Bromodomain-containing protein 8	Q8R3B7-1; Q8R3B7-2
FSYSHTGL	8		8.1	Phosphatidylinositol 4,5-bisphosphate 3-kinase catalytic subunit alpha isoform	P42337
INLSFNKL	8		8.1	Leucine-rich repeat-containing protein 40	Q9CRC8

IALAFHLL	8		8.1	Actin-histidine N-methyltransferase	Q91WC0-4; Q91WC0; Q91WC0-2
RTYSFLNLL	9		8.1	Transmembrane protein 39B	Q810L4
TAYLFSRF	8		8.1	Exportin-T	Q9CRT8
STIVYYKL	8		8.2	tRNA-splicing endonuclease subunit Sen15	Q8R3W5
VNYEFGIAL	9		8.2	Uncharacterized protein C12orf29 homolog	Q8BHN7; Q8BHN7-2
AVFTFEQL	8		8.2	Protein Niban	Q3UW53
AMYSFINAL	9		8.2	Protein TALPID3	E9PV87
STLIYRNM	8		8.3	Protein lin-37 homolog	Q9D8N6
KSRIFQNL	8		8.3	Procollagen-lysine,2-oxoglutarate 5-dioxygenase 3	Q9R0E1
STLTYSRM	8		8.3	Serine/threonine-protein kinase Sgk1	Q9WVC6-1
LGYLYRCL	8		8.3	XK-related protein 8	Q8C0T0
ISVEFCHV	8		8.3	Vacuolar protein sorting-associated protein 53 homolog	Q8CCB4
NGYKYMAL	8		8.3	Alpha-galactosidase A	P51569
SAMVFSAM	8	2xOxidation [M3; M8	8.4	V-type proton ATPase 16 kDa proteolipid subunit	P63082
SNYRVSLL	8		8.5	Isoform 2 of CLIP-associating protein 2	Q8BRT1-5
VNVRFTGV	8		8.5	Ubiquitin carboxyl-terminal hydrolase MINDY-2	Q76LS9-2; Q6PDI6-3; Q6PDI6; Q76LS9-3; Q76LS9-1; Q6PDI6-2
INIEMYQRL	9		8.5	RUN and FYVE domain-containing protein 2	Q8R4C2
VNYPFIDM	8		8.5	ETS domain-containing transcription factor ERF	P70459
VNQKFNNL	8		8.6	Disks large-associated protein 5	Q8K4R9; Q8K4R9-3; Q8K4R9-2
SALEFLTHL	9		8.6	BRCA1-associated ATM activator 1	Q8C3R1-2; Q8C3R1-1
TALNFLHL	8		8.6	Cyclin-G2	O08918
LQYCFPRL	8		8.6	DNA primase small subunit	P20664
AQYRFIYM	8	1xOxidation [M8]	8.7	tyrosine-protein phosphatase non-receptor type 11	P35235; P35235-2
VSLNYTKV	8		8.7	Microtubule-associated protein RP/EB family member 2	Q8R001; Q8R001-2
TSVQFMKL	8		8.7	Fatty acid synthase	P19096
SAALFSRL	8		8.7	Mitochondrial import inner membrane translocase subunit TIM50	Q9D880
RALNYTHL	8		8.8	nuclear pore complex protein Nup98-Nup96	Q6PFD9
SSLRFTTI	8		8.8	WD repeat-containing protein 43	Q6ZQL4
YAMIYRNL	8		8.9	E3 ubiquitin-protein ligase Mdm2	P23804-1; P23804-2
QTFTFTRV	8		8.9	Extended synaptotagmin-1	Q3U7R1; Q3U7R1-2
VQWEYGRL	8		8.9	Glutamine--tRNA ligase OS=Mus musculus OX=10090	Q8BML9
RILEFYSKL	9		8.9	protein O-GlcNAcase	Q9EQQ9-1; Q9EQQ9-3; Q9EQQ9-2
RVYYFNHI	8		9	peptidyl-prolyl cis-trans isomerase NIMA-interacting 1	Q9QUR7
VRYDFSGL	8		9	Ceramide kinase	Q8K4Q7
VAFDFYHKASI	12		9	E3 ubiquitin-protein ligase UBR1	O70481
SNIQYRSL	8		9.1	neuron navigator 1	Q8CH77-2; Q8CH77; Q8CH77-3; Q8CH77-4
TNLRYLAL	8		9.1	AP-2 complex subunit alpha-2	P17426-2; P17427; P17426
RNILYHNL	8		9.1	Cell surface hyaluronidase	Q5FWI3
VCMAFAGL	8		9.1	Proteasome subunit alpha-type 7-like	Q9CWH6; Q9Z2U0
YNWRYKNL	8		9.2	Intron-binding protein aquarius	Q8CFQ3
ANLIYYSL	8		9.2	ATP synthase subunit gamma, mitochondrial	Q91VR2
RGFFFSHV	8		9.2	Acyl-CoA desaturase 2	Q99PL7; Q6T707; P13011; P13516
SSPKFSEL	8		9.3	Cytoskeleton-associated protein 5	A2AGT5; Q91VM3-2; A2AGT5-3; Q91VM3; A2AGT5-2
NSFRYNGL	8		9.3	60S ribosomal protein L28	P41105
VNYRVPNM	8		9.3	1-phosphatidylinositol 4,5-bisphosphate phosphodiesterase gamma-1	Q62077
SIYRFHGQFL	10		9.3	FYVE, RhoGEF and PH domain-containing protein 3	O88842-2; O88842
TQYLFIKL	8		9.3	Probable leucine--tRNA ligase, mitochondrial	Q8VDC0
VAYLMQKL	8		9.4	Dual specificity protein phosphatase 6	Q9DBB1
RTYTYEKL	8		9.4	Catenin beta-1	Q02248
RSYLFLGGI	9		9.4	Bax inhibitor 1	Q9D2C7
VMLETYRNL	9		9.4	Zinc finger protein 431	Q8BZW4-2; E9QAG8; Q8BZW4
SNLYYKYL	8		9.4	A-kinase anchor protein 10, mitochondrial	O88845-2; O88845-3; O88845
ITFSYVNNM	9		9.4	Ceramide synthase 6	Q8C172
CSPRFFSL	8		9.4	Ral GTPase-activating protein subunit alpha-2	A3KGS3; A3KGS3-2

GVFSFSRL	8	9.4	Transmembrane protein 260	Q8BMD6
VNTHFSHL	8	9.5	Tetratricopeptide repeat protein 21B	Q0HA38
ATIFFTRL	8	9.5	phosphatidylinositol 4-phosphate 3-kinase C2 domain-containing subunit alpha	Q61194-1; Q61194-2
VAIRFDSGL	9	9.5	zinc finger protein 22	Q9ERU3
VAYKFPELL	9	9.5	E3 ubiquitin-protein ligase UBR2	Q6WKZ8-1; Q6WKZ8-3; Q6WKZ8-2
VSYPHLVLL	9	9.5	Tyrosine-protein kinase JAK3	Q62137; Q62137-2
KVFEFYLL	8	9.6	Probable C-mannosyltransferase DPY19L4	A2AJQ3-2; A2AJQ3-1
SGIVYSRM	8	9.6	acetoacetyl-CoA synthetase	Q9D2R0
RVYKFCSKL	9	9.6	CREB-binding protein	P45481
AAYGFRNI	8	9.6	Nuclear prelamin A recognition factor	Q9CYQ7
IVWEFEQL	8	9.6	Zinc finger protein RFP	Q62158
SNALFAKL	8	9.7	Ankyrin repeat domain-containing protein 13B	Q5F259; Q5F259-2
SQFKFLQRL	9	9.7	Phospholipid-transporting ATPase ID	P98199
KGYVFKEL	8	9.8	Toll-like receptor 7	P58681
STRLFAVL	8	9.8	Dolichyl-diphosphooligosaccharide--protein glycosyltransferase subunit STT3A	P46978
KTFMYNEL	8	9.8	kelch domain-containing protein 4	Q921I2
KAFHFPSL	8	9.8	Zinc finger protein 728	Q6P5C7
RSYENHMYL	9	9.9	Chromodomain-helicase-DNA-binding protein 1-like	Q9CXF7-2; Q9CXF7-1
TNYNFQYISL	10	9.9	glycerol-3-phosphate acyltransferase 4	Q8K2C8
KSYLMNRL	8	9.9	Guanylate-binding protein 4	Q61107
AGFAFSCL	8	9.9	Signal peptidase complex subunit 1	Q9D958
TNFEYLTHL	9	10	Lysosomal-trafficking regulator	P97412-1
AVVRFPRL	8	10	Integrator complex subunit 7	Q7TQK1; Q7TQK1-2
KSYEFEDL	8	10.1	DENN domain-containing protein 2A	Q8C4S8
VSQYYPKL	8	10.1	Heterochromatin protein 1-binding protein 3	Q3TEA8; Q3TEA8-3; Q3TEA8-2
LGYKYVGM	8	10.2	Protein YIF1B	Q9CX30-1; Q9CX30-2
SIVSYNHL	8	10.2	Inositol-3-phosphate synthase 1	Q9JHU9
TNLVYPAL	8	10.4	Hypoxia-inducible factor 1-alpha inhibitor	Q8BLR9-1; Q8BLR9-2
RAFEFTYV	8	10.4	dTDP-D-glucose 4,6-dehydratase	Q8VDR7
SIYDPFAGM	9	10.4	Phosphoinositide 3-kinase adapter protein 1	Q9EQ32-2; Q9EQ32-1
VVYTPWSNL	9	10.4	Coiled-coil domain-containing protein 25	Q78PG9
IAYLYDRLL	9	10.4	DNA replication complex GINS protein PSF1	Q9CZ15
AVYQFGSAL	9	10.4	Membrane progestin receptor alpha	Q80ZE4
SALFFHYL	8	10.4	E3 ubiquitin-protein ligase UBR2	Q6WKZ8; Q6WKZ8-3; Q6WKZ8-2
VAFNYKTV	8	10.5	Prostaglandin reductase 1	Q91YR9
KVYTFNSV	8	10.5	Plasma membrane calcium-transporting ATPase 4	Q6Q477-2; Q6Q477; G5E829; Q9R0K7
TNISFTNM	8	10.5	PRELI domain containing protein 3B	Q9CYY7
AILKFQGL	8	10.5	ATP-binding cassette sub-family B member 6, mitochondrial	Q9DC29
SNITFVVNL	9	10.5	Transmembrane glycoprotein NMB	Q99P91
VAFDFTKV	8	10.6	Phosphatidylinositol-binding clathrin assembly protein	Q7M6Y3-1; Q7M6Y3-5; Q7M6Y3-6; Q7M6Y3-4; Q7M6Y3-2; Q7M6Y3-3
STYGWTANM	9	10.6	Heat shock protein HSP 90-beta	P07901; P11499
IIQEFPAL	8	10.6	eIF-2-alpha kinase GCN2	Q9QZ05; Q9QZ05-3; Q9QZ05-4; Q9QZ05-2
VIFEMVHL	8	10.6	Insulin receptor	P15208
VNLEYLARV	9	10.7	Cytoplasmic aconitate hydratase	P28271
QSPGFYRNV	9	10.7	Tryptophan--tRNA ligase, cytoplasmic	P32921-2; P32921
IGLDYSSL	8	10.7	Class E basic helix-loop-helix protein 41	Q99PV5
HTFTYTGL	8	10.7	Mediator of RNA polymerase II transcription subunit 12	A2AGH6-2; A2AGH6
LQYEFTKL	8	10.8	Formin-like protein 3	A2APV2-3; Q6ZPF4-2; Q6ZPF4-1; A2APV2-1; A2APV2-2
YGYHFPEL	8	10.8	Nucleolar protein 56	Q9D6Z1
QAFKFRRV	8	10.8	E3 ubiquitin-protein ligase UBR2	Q6WKZ8-1; Q6WKZ8-3; Q6WKZ8-2
SAFDFENM	8	10.8	Rab GDP dissociation inhibitor alpha	P50396
IAVSFREL	8	10.8	Nucleolar protein 11	Q8BJW5-2; Q8BJW5
AFYQFVNNL	9	10.8	E3 ubiquitin-protein ligase RLIM	Q9WTV7
KSLDYLNL	8	10.9	UNC119-binding protein C5orf30 homolog	Q8VEB3

SSPGYSHL	8	10.9	Low-density lipoprotein receptor-related protein 10	Q7TQH7
KNIRYVAL	8	10.9	AP-1 complex subunit gamma-1	O88512; P22892
SSLSFNTRL	9	10.9	vesicle transport protein sft2a	Q5SSN7
RSIDQFANL	9	10.9	U6 snRNA-associated Sm-like protein LSm1	Q8VC85
LSLPFEARL	9	10.9	Galectin-8	Q9JL15
IIIIYNRV	8	10.9	Heparan sulfate 2-O-sulfotransferase 1	Q8R3H7
SVVRYVQL	8	11	U6 snRNA-associated Sm-like protein LSm2	O35900
RSPWFTTL	8	11	MLV-related proviral Env polyprotein	P10404; P11370
VTWRVTNL	8	11	MLV-related proviral Env polyprotein	P10404
VNLTFRTV	8	11	alpha-ketoglutarate-dependent dioxygenase alkB homolog 3	Q8K1E6
SQIRFGLL	8	11	Conserved oligomeric Golgi complex subunit 1	Q9Z160
GVFRFLSNL	9	11	Leucine-rich repeats and immunoglobulin-like domains protein 2	Q52KR2
KIIEFANI	8	11	Meiosis-specific nuclear structural protein 1	Q61884
SNHVFNAL	8	11	Serine/threonine-protein kinase RIO3	Q9DBU3
RNYQFDFL	8	11.1	splicing factor 3A subunit 1	Q8K4Z5
SIYRVPLL	8	11.1	CTP synthase 1	P70303; P70303-3; P70303-2; P70698
TNVLFNHL	8	11.1	RNA polymerase II-associated protein 3	Q9D706
TNLRFLNRL	9	11.1	Up-regulator of cell proliferation	Q5NCI0-2; Q5NCI0-1
IIITFNDL	8	11.1	Peptide-N(4)-(N-acetyl-beta-glucosaminyl)asparagine amidase	Q9JI78
VQMKFRLL	8	11.2	G2/mitotic-specific cyclin-B1	P24860
RAIAFQHL	8	11.2	Myb-binding protein 1A	Q7TPV4
KNFPFERL	8	11.2	U5 small nuclear ribonucleoprotein 200 kDa helicase	Q6P4T2
IGPRFVLNL	9	11.2	Ribosome biogenesis protein BRX1 homolog	Q9DCA5
VINVFHHL	8	11.3	Cyclin-L1	Q52KE7-2; Q52KE7
ATLEFTQL	8	11.3	Nitric oxide synthase, inducible	P29477
VIHIFSHI	8	11.4	adenine DNA glycosylase	Q99P21
SMVAFENL	8	11.4	poly [ADP-ribose] polymerase 14	Q2EMV9
AALRFLSQL	9	11.4	Vacuolar protein sorting-associated protein 33A	Q9D2N9
QALKYFNL	8	11.4	Protein sel-1 homolog 1	Q9Z2G6; Q9Z2G6-2
RAFLFRIL	8	11.4	Nesprin-1	Q6ZWR6-2; Q6ZWR6-3; Q6ZWR6-4; Q6ZWR6
AIRVFANI	8	11.5	eukaryotic translation initiation factor 3 subunit L	Q8QZY1
KGFRFTLV	8	11.5	Dolichyl pyrophosphate Man9GlcNAc2 alpha-1,3-glucosyltransferase	Q3TAE8
SAFAFMESL	9	11.5	Short-chain dehydrogenase/reductase 3	O88876-2; O88876
STRVYASM	8	11.6	Transforming growth factor beta regulator 1	Q3UB74
RNYSYEKL	8	11.6	Junction plakoglobin	Q02257
KGYLFNTV	8	11.7	M-phase inducer phosphatase 1	P48964
VIVLFGARL	9	11.7	2-hydroxyacyl-CoA lyase 1	Q9QXE0
EVYSFSGL	8	11.7	Oxidative stress-responsive serine-rich protein 1	Q9D722
SSYQHTSV	8	11.8	Exportin-T	Q9CRT8
TGYAYRHPL	9	11.8	Gamma-adducin	Q9QYB5-2; Q9QYB5-1
TAPQYYRL	8	11.9	DALR anticodon-binding domain-containing protein 3	Q6PJN8
RNYEYLIRL	9	11.9	Transcription factor 25	Q8R3L2-1; Q8R3L2-4; Q8R3L2-2; Q8R3L2-3; Q8R3L2-5
SNFRRHILL	9	11.9	THO complex subunit 1	Q8R3N6
VSFLLPKL	8	11.9	Leucine-rich repeat and WD repeat-containing protein 1	Q8BUI3
AVFTWTNL	8	12	Disco-interacting protein 2 homolog A	Q8BWT5; Q3UH60-2; Q3UH60
RNLEYLNL	8	12.1	F-box/LRR-repeat protein 2	Q8BH16
RNPTFMGL	8	12.1	AP-2 complex subunit alpha-2	P17427
IDYEFSAL	8	12.1	5'-3' exoribonuclease 1	P97789-2; P97789-3; P97789
SSVKFNPV	8	12.2	DDB1- and CUL4-associated factor 13	Q6PAC3
SSPHYTTL	8	12.2	Alpha-mannosidase 2	P27046
VGVTYRTL	8	12.2	Protein MMS22-like	B1AUR6-2; B1AUR6-1
VAYRHLVGV	9	12.3	Cytochrome c1, heme protein, mitochondrial	Q9D0M3; Q9D0M3-2
AVYTFETLL	9	12.3	Niban-like protein 1	Q8R1F1
VQYVLPRL	8	12.3	Son of sevenless homolog 1	Q62245

VSYWFDQRF	9		12.4	CMP-N-acetylneuraminate-beta-galactosamide-alpha-2,3-sialyltransferase 1	P54751
SGYIPARL	8		12.5	Serine/threonine-protein kinase PLK1	Q07832
SLYRFTTI	8		12.7	DEP domain-containing protein 7	Q91WS7
VINVFHRL	8		12.7	cyclin-L2	Q9JJA7-2; Q9JJA7-3; Q9JJA7-1
CIINFQHL	8		12.7	Activating signal cointegrator 1 complex subunit 3	E9PZJ8-1
SNFVFARTM	9	1xOxidation [M9]	12.7	Insulin-like growth factor 1 receptor	Q60751
QILWFRGL	8		12.7	Plasma membrane calcium-transporting ATPase 1	G5E829; Q9R0K7
STYLRLQLL	9		12.7	Extracellular serine/threonine protein kinase FAM20C	Q5MJS3
SIFEFVHAL	9		12.7	Importin-9	Q91YE6
KSIAFQNV	8		12.8	zinc finger protein 445	Q8R2V3
LNYFFRAI	8		12.8	Serine/threonine-protein phosphatase 2A regulatory subunit B'' subunit gamma	Q9JK24
SHYEFHNI	8		13	Phosphatidylinositol 3,4,5-trisphosphate-dependent Rac exchanger 1 protein	Q69ZK0
SSFSWTNGL	9		13	Transmembrane protein 138	Q9D6G5
TSPHYQNL	8		13.1	Tyrosine-protein phosphatase non-receptor type 18	Q61152
VSTKFEHL	8		13.1	trafficking protein particle complex subunit 11	B2RXC1
QQYLFDRL	8		13.1	Nardilysin	Q8BHG1
RAPGFARL	8		13.1	Glutamine amidotransferase-like class 1 domain-containing protein 1	Q8BFQ8
AQFNFQNV	8		13.1	Poly(A) RNA polymerase GLD2	Q91YI6; Q91YI6-2
HQYQFNNL	8		13.2	Integrin alpha-M	P05555-1; P05555-2
KTYQHFTL	8		13.2	Alpha-1,3-mannosyl-glycoprotein 4-beta-N-acetylglucosaminyltransferase B	Q812F8
LIYNFGCHL	9		13.2	Death domain-associated protein 6	O35613
YGVLFRNL	8		13.3	Leukocyte surface antigen CD53	Q61451
RIYGFTAV	8		13.3	E3 ubiquitin-protein ligase hectd1	Q69ZR2
TSLAFESRL	9		13.3	Cytospin-B	Q5SXY1-1; Q5SXY1-2
IVYTFMTHF	9		13.3	Ubiquitin carboxyl-terminal hydrolase 34	Q6ZQ93-1; Q6ZQ93-3; Q6ZQ93-2
QVFKYRKL	8		13.4	lysosomal acid lipase/cholesteryl ester hydrolase	Q9Z0M5
SAVVFRHM	8		13.4	Protocadherin Fat 3	Q8BNA6
IAYQFLRA	8		13.4	Structural maintenance of chromosomes protein 2	Q8CG48
RAFTYSTV	8		13.5	Solute carrier family 17 member 9	Q8VCL5
VIWKYPTM	8		13.5	cell division cycle protein 20 homolog	Q9JJ66
QSIAFISRL	9		13.5	Transmembrane protein 33	Q9CR67
RSYQQALL	8		13.6	SERTA domain-containing protein 3	Q9ERC3
KAYIFEGAL	9		13.6	Vacuolar ATPase assembly integral membrane protein vma21	Q78T54
VNSIFQHL	8		13.7	Interferon-induced very large GTPase 1	Q80SU7
KNFAFTMV	8		13.7	Phospholipid-transporting ATPase ID	P98199
SEYRYTLL	8		13.7	Endoplasmic reticulum resident protein 44	Q9D1Q6
SSYTFPKMM	9		13.7	Enoyl-CoA delta isomerase 2, mitochondrial	Q9WUR2; Q9WUR2-2
KCYLFGGL	8		13.8	Host cell factor 1	Q61191
VITEFARI	8		13.9	tRNA (guanine(26)-N(2))-dimethyltransferase	Q3TX08
ESFKFVRL	8		13.9	Translation initiation factor eIF-2B subunit alpha	Q99LC8
TNHSYFNL	8		13.9	Aldose 1-epimerase	Q8K157
VAFKHLFL	8		13.9	Mucolipin-2	Q8K595-2; Q8K595
AVLRYTKL	8		14	Replication factor C subunit 2	Q9WUK4
VTVVFSTV	8		14	Actin-related protein 2/3 complex subunit 2	Q9CVB6
IGPRFKLL	8		14	Phosphatidylinositol 4-kinase alpha	E9Q3L2
VNWRFMRGI	9		14	E3 ubiquitin-protein ligase SMURF1	Q9CUN6
IVFEDFARL	9		14.1	Beta-arrestin-2	Q91YI4-1; Q91YI4-2
YTFVYRVL	8		14.1	Tyrosine-protein phosphatase non-receptor type 21	Q62136
SGYDFENRL	9		14.2	Tetratricopeptide repeat protein 39C	Q8VE09
INFLHHNKL	9		14.2	dual specificity protein kinase CLK4	O35493-1; O35493-2
AIVEFLSNL	9		14.3	Chromatin assembly factor 1 subunit B	Q9D0N7
SSVYFRSV	8		14.4	BLOC-1-related complex subunit 8	Q9D6Y4
TIIVFHSL	8		14.4	Eyes absent homolog 4	P97767-1; Q9Z191; P97767-2
RGLSRYPNL	9		14.4	protein farnesyltransferase/geranylgeranyltransferase type-1 subunit alpha	Q61239

VTIHYNKL	8	14.6	PRKCA-binding protein	Q62083
KNFAFTLV	8	14.6	phospholipid-transporting ATPase FetA	Q148W0; A3FIN4-1
SGLKYVNV	8	14.6	Adenylate kinase isoenzyme 6	Q8VCP8
RGLDYFSSL	9	14.6	Transmembrane protein 245	B1AZA5
STFIYNSI	8	14.6	Guanylate-binding protein 1	Q01514; Q9Z0E6
AVFRFKVL	8	14.6	STARD3 N-terminal-like protein	Q9DCI3
KNLNYLHL	8	14.7	Pre-mRNA-processing-splicing factor 8	Q99PV0
RGPLFSHL	8	14.7	Zinc transporter ZIP6	Q8C145
RGYEFIVRL	9	14.8	serine/threonine-protein kinase ATR	Q9JKK8
SIMAFHKL	8	14.8	Myotubularin-related protein 10	Q7TPM9
RGYIYWRL	8	14.9	AP-1 complex subunit beta-1	Q9DBG3-2; O35643; Q9DBG3
TSPTYRSL	8	14.9	Regulator of G-protein signaling 19	Q9CX84
VNMVPFPRL	9	14.9	tubulin beta-3 chain	Q7TMM9; Q9D6F9; Q9ERD7; A2AQ07; P99024; P68372; Q9CWF2; Q922F4
IGPYYRKL	8	15	Transmembrane and coiled-coil domain-containing protein 3	Q8BH01; Q8BH01-2
HTYVHATL	8	15	Two pore calcium channel protein 1	Q9EQJ0-2; Q9EQJ0
SILRYLARI	9	15	Bifunctional glutamate/proline--tRNA ligase	Q8CGC7
VTYSFRQSF	9	15	IQ motif and SEC7 domain-containing protein 1	Q8R0S2-2; Q5DU25; Q8R0S2
KNFKFLGTL	9	15	Ras-responsive element-binding protein 1	Q3UH06-2; Q3UH06-3; Q3UH06-1
VGNEFSHL	8	15	E3 ubiquitin-protein ligase Arkadia	Q99ML9; Q99ML9-2
TEYVFTHL	8	15.1	Fibronectin type III domain-containing protein 3B	Q6NWW9
AFYTVAHL	8	15.1	Leucine--tRNA ligase, cytoplasmic	Q8BMJ2
VNFPFLVKL	9	15.1	cAMP-dependent protein kinase catalytic subunit alpha	P05132-2; P05132-1
TVIIYVRL	8	15.1	Dolichyl-diphosphooligosaccharide--protein glycosyltransferase subunit 1	Q91YQ5
KGLDFALL	8	15.2	Protein Red	Q9Z1M8
SSPVYIDL	8	15.2	Uridine 5'-monophosphate synthase	P13439
IILKYIGM	8	15.3	ADP-ribosylation factor-like protein 6-interacting protein 1	Q9JKW0
RVLLFRNM	8	15.3	Ubiquitin-protein ligase E3B	Q9ES34
AQFRYLQRL	9	15.3	Phospholipid-transporting ATPase IC	Q148W0
VTVDFSKL	8	15.3	DNA helicase B	Q6NVF4
VQYEPAHL	8	15.5	Zinc finger and BTB domain-containing protein 18	Q9WUK6
IGVIFTHV	8	15.5	Ribonuclease 3	Q5HZJ0
GVLKFARL	8	15.5	Protein C10	O35127
RIILFGSL	8	15.6	Fanconi anemia group D2 protein homolog	Q80V62
CIIAFQRL	8	15.7	Basic immunoglobulin-like variable motif-containing protein	Q8CBX9
FSQEYINL	8	15.7	Minor histocompatibility antigen H13	Q9D8V0; Q9D8V0-3; Q9D8V0-2; Q9D8V0-4
AAVKFHNL	8	15.8	Glycerol-3-phosphate dehydrogenase, mitochondrial	Q64521
SNATFARV	8	15.8	DNA polymerase delta subunit 2	O35654
FNLVYENL	8	15.8	NFAT activation molecule 1	Q8R4V1; Q8R4V1-2; Q8R4V1-3
FQFTFKHL	8	15.8	DnaJ homolog subfamily A member 2	Q9QYJ0
GILTFSNL	8	15.8	Nodal modulator 1	Q6GQT9
VNVPFHLAL	9	15.8	Rab3 GTPase-activating protein non-catalytic subunit	Q8BMG7
TAISFNLL	8	15.8	Natural resistance-associated macrophage protein 1	P41251
RGYEFLGV	8	15.9	F-box/SPRY domain-containing protein 1	Q8K3B1
VNYDFGHMHV	10	15.9	E3 ubiquitin-protein ligase RNF216	P58283-2; P58283
AGYGFCHV	8	16	b9 domain-containing protein 2	Q3UK10
SGYEFIHKL	9	16	Tyrosine--tRNA ligase, mitochondrial	Q8BYL4
TGLPYYTGV	9	16	Ubiquitin-associated protein 2	Q91VX2
YEYLFTNL	8	16.1	Protein artemis	Q8K4J0-3; Q8K4J0-2; Q8K4J0
SVYLVRQL	8	16.2	E3 SUMO-protein ligase PIAS3	Q8C5D8-2; O54714-2; O54714; Q8C5D8-5; Q8C5D8-4; O54714-3; Q8C5D8-3; Q8C5D8-1
RTFEFQLM	8	16.3	Programmed cell death protein 2-like	Q8C5N5
FSILFNRV	8	16.3	Cytosolic phospholipase A2	P47713
RIYIFHYGI	9	16.3	Lysophospholipid acyltransferase 1	Q8BH98

TAYLFSRFV	9		16.5	Exportin-T	Q9CRT8
ANFRFTDRL	9		16.6	Haptoglobin	Q61646
STLKFLKL	8		16.6	Nucleotide-binding oligomerization domain-containing protein 2	Q8K3Z0; Q8K3Z0-2
TSVVFNKL	8		16.7	DNA-directed RNA polymerase I subunit RPA12	Q791N7
KTFSYAGF	8		16.7	T-complex protein 1 subunit eta	P80313
AGPWYRNL	8		16.7	Probable phospholipid-transporting ATPase IIB	P98195-2; P98195
STFEFHSI	8		16.8	E3 ubiquitin-protein ligase UBR1	O70481
INYQHLRI	8		16.8	2'-5'-oligoadenylate synthase 1A	P11928
SNVKYVML	8		16.8	Protein patched homolog 1	Q61115
KTYEHFNAM	9		16.8	NADPH--cytochrome P450 reductase	P37040
VNIQYLDL	8		17	Nuclear pore complex protein Nup133	Q8R0G9
ISTIFKSL	8		17	Ubiquitin conjugation factor E4 B	Q9ES00
ASPIFTHV	8		17.1	Pre-mRNA-splicing factor CWC22 homolog	Q8C5N3-1; Q8C5N3-2
SNPEFRQL	8		17.3	Lysine-specific demethylase 7A	Q3UWM4
RILEFFGL	8		17.3	Protein disulfide-isomerase	P09103
KNFQYRQV	8		17.4	Isoform 3 of Endothelin-converting enzyme 2	Q80Z60; Q80Z60-3; P0DPD9; Q80Z60-2
KTFLFSATM	9		17.4	Probable ATP-dependent RNA helicase DDX47	Q9CWX9
ISPRFDVQL	9		17.4	40S ribosomal protein S15a	P62245
SCIRFINL	8		17.5	SLIT-ROBO Rho GTPase-activating protein 3	Q812A2; Q91Z69
SAVSFHSL	8		17.6	Laccase domain-containing protein 1	Q8BZT9
RIFQFQNF	8		17.7	Pre-rRNA-processing protein TSR1 homolog	Q5SWD9-1; Q5SWD9-2
IVELFRNL	8		17.8	Signal transducer and activator of transcription 3	P42227-3; P42227; P42227-2
KIYEFDYHL	9		17.8	DNA topoisomerase 3-alpha	O70157
VNVEFVRV	8		17.9	[F-actin]-methionine sulfoxide oxidase MICAL2	Q8BML1-3; Q8BML1; Q8BML1-2
KGWNFNYL	8		18	sphingosine-1-phosphate lyase 1	Q8R0X7
VSYRWICEM	9		18.1	C-type lectin domain family 5 member A OS=Mus musculus OX=10090	Q9R007
SLLRFNAL	8		18.1	Piezo-type mechanosensitive ion channel component 1	E2JF22
KVFEYHNV	8		18.2	Amyloid protein-binding protein 2	Q9DAX9
VVYPFPTF	8		18.2	DDB1- and CUL4-associated factor 15	Q6PFH3-1; Q6PFH3-2
VNYLRTVSL	9		18.2	Prokineticin receptor 1	Q9JKL1; Q8K458
RSYSFLNSSL	10		18.3	Deoxyribonuclease-1-like 1	Q9D7J6
SQYDFIDL	8		18.3	Dipeptidyl peptidase 8	Q80YA7
IGYPFLVSV	9		18.3	Ubiquitin carboxyl-terminal hydrolase 19	Q3UJD6-2; Q3UJD6
TIIIFHSL	8		18.4	eyes absent homolog 3	O08575; O08575-2; P97480; P97480-2
VIVEFRDL	8		18.4	Protein arginine N-methyltransferase 7	Q922X9
ESYSFEARM	9		18.5	Zinc finger MYND domain-containing protein 19	Q9CQG3-1
RGFEFTLM	8		18.5	Septin-7	O55131
VCWAFSSL	8		18.5	Importin subunit beta-1	P70168
VHYKYTVV	8		18.6	Neutrophil cytosol factor 2	O70145
TTFSYVNNM	9	1xOxidation [M9]	18.6	Ceramide synthase 5	Q9D6K9-2; Q9D6K9
VSFDFHGRRM	10		18.6	nucleoporin Seh1	Q8R2U0-1; Q8R2U0-2
AAPHFFHL	8		18.8	Isoform 3 of AT-rich interactive domain-containing protein 5A	Q3U108-3; Q3U108; Q3U108-4; Q3U108-5; Q3U108-2
AQYKFIYV	8		18.9	tyrosine-protein phosphatase non-receptor type 6	P29351; P29351-2; P29351-3
MNRVFLQRL	9		18.9	ATP-binding cassette sub-family D member 1	P48410
RNMEYYKSL	9		18.9	Cartilage-associated protein	Q9CYD3
RSPAFTSRL	9		19	E3 ubiquitin-protein ligase HUWE1	Q7TMY8-4; Q7TMY8-3; Q7TMY8; Q7TMY8-2
GNYSFYVL	8		19	Insulin-like growth factor 1 receptor	Q60751
KGLRFIQL	8		19.1	Calpain-7	Q9R1S8
SSIVFGRF	8		19.1	Leukocyte immunoglobulin-like receptor subfamily B member 4	Q64281; Q64281-2
KVFIFRCL	8		19.1	Protein MMS22-like	B1AUR6
RIYPTFLHL	9		19.1	FACT complex subunit SSRP1	Q08943-2; Q08943
RIYGKFLGL	9		19.2	erine/threonine-protein phosphatase 2A 56 kDa regulatory subunit gamma isoforr	Q60996-2; Q60996-4; Q60996-1; Q6PD03; Q61151; Q60996-3
SALPFVKL	8		19.3	Protein TANC1	Q0VGY8-2; Q0VGY8
FNLVFERL	8		19.3	Ankyrin repeat and SOCS box protein 6	Q91ZU1

KTFDFKGL	8		19.5	Suppression of tumorigenicity 5 protein	Q924W7
VGITYQHI	8		19.6	Eukaryotic translation initiation factor 3 subunit K	Q9DBZ5-2; Q9DBZ5
QSLAFHTL	8		19.7	elongator complex protein 2	Q91WG4-2; Q91WG4
INFDFNTI	8		19.8	BTB/POZ domain-containing protein KCTD20	Q8CDD8-2; Q8CDD8
VTVVFERM	8		19.9	Protein MON2 homolog	Q80TL7-1; Q80TL7-2
FVYIFQEV	8		19.9	Plastin-3	Q99K51
RNLEQFARL	9		20	ATP-dependent RNA helicase DDX24	Q9ESV0
SIVQFYYM	8	1xOxidation [M8]	20.1	Metastasis-associated protein MTA2	Q9R190
AVLSFSTRL	9		20.1	Dolichyl-diphosphooligosaccharide--protein glycosyltransferase subunit STT3A	P46978
TNLIYNLL	8		20.1	Leucyl-cystinyl aminopeptidase	Q8C129
IGLAYVNHL	9		20.1	Vacuolar protein sorting-associated protein 41 homolog	Q5KU39
KGFEFTLM	8		20.2	septin-2	P42208
RNYQHQLL	8		20.3	centrosomal protein of 295 kDa	Q8BQ48-1; Q8BQ48-6; Q8BQ48-4; Q8BQ48-2; Q8BQ48-5; Q8BQ48-3
IGYGFRCV	8		20.4	Inward rectifier potassium channel 4	P35561; P52189
VTLVFEHI	8		20.6	Cyclin-dependent kinase 4	P30285
SNVVFKLL	8		20.7	Prefoldin subunit 6	Q03958
ASPEFTKL	8		20.9	protein FAM98A	Q3TJZ6
SSFVLPKL	8		21	Ras and Rab interactor 2	Q9D684-2; Q9D684-1
VQYTFDLQL	9		21	G/T mismatch-specific thymine DNA glycosylase	P56581-2; P56581
SSMAFKQM	8		21.2	Transgelin-2	Q9WVA4
KTVCFQNL	8		21.2	cytoplasmic FMR1-interacting protein 1	Q7TMB8-1
INYDYVHEL	9		21.2	Zinc finger CCCH domain-containing protein 13	E9Q784
TNQNFTHL	8		21.3	NACHT, LRR and PYD domains-containing protein 3	Q8R4B8-3; Q8R4B8-1
SIINFIERL	9		21.3	Putative homeodomain transcription factor 1	Q9QZ09
RSYDFEFM	8		21.5	Vacuolar protein sorting-associated protein 26A	P40336; P40336-2
ICFKFDHL	8		21.6	F-actin-capping protein subunit alpha-2	P47754
INYILRVL	8		21.7	E3 ubiquitin-protein ligase HUWE1	Q7TMY8-4; Q7TMY8-3; Q7TMY8; Q7TMY8-2
IDYSFPSL	8		21.7	MKI67 FHA domain-interacting nucleolar phosphoprotein	Q91VE6; Q91VE6-2
TALRFLEL	8		21.7	Phosphoinositide 3-kinase regulatory subunit 4	Q8VD65
VAVTFSERL	9		21.8	Cystine/glutamate transporter	Q9WTR6
AGFAFLTGV	9		21.9	Bax inhibitor 1	Q9D2C7
ASITFEHM	8		22	Eukaryotic translation initiation factor 3 subunit H	Q91WK2
TAVVFVAL	8		22	Sodium/calcium exchanger 1	P70414
RNFNYHIL	8		22.1	Cleavage and polyadenylation specificity factor subunit 3	Q9QXK7
FTFKYHHV	8		22.2	Membrane-associated progesterone receptor component 1	O55022
SMYQPLNL	8		22.2	Globoside alpha-1,3-N-acetylgalactosaminyltransferase 1	Q8VI38
LQFQFAEV	8		22.3	Leucine-rich repeat flightless-interacting protein 1	Q3UZ39-2; Q3UZ39
VGAEYARV	8		22.4	MAU2 chromatid cohesion factor homolog	Q9D2X5-1; Q9D2X5-2
VVRSFLHARL	10		22.4	26S proteasome non-ATPase regulatory subunit 3	P14685
QAPRYSSL	8		22.6	DENN domain-containing protein 2A	Q8C4S8
RALDFLSRL	9		22.6	Breast cancer type 2 susceptibility protein homolog	P97929
RNIDYYRL	8		22.7	transcriptional regulator ATRX	Q61687
LCYKYRGL	8		22.8	KICSTOR complex protein kaptin OS=Mus musculus OX=10090	Q8VCX6
KCYYYHARV	9		22.8	26S proteasome non-ATPase regulatory subunit 3	P14685
SSPVFKAM	8		22.9	Kelch-like ECH-associated protein 1	Q9Z2X8
IIVQFRYI	8		23	Magnesium transporter NIPA1	Q8BHK1
RSYNMPSL	8		23	Melanoma inhibitory activity protein 2	Q91ZV0; Q8R311
VVARFLSL	8		23.3	Mini-chromosome maintenance complex-binding protein	Q8R3C0
ETYKYFSL	8		23.6	Transmembrane 9 superfamily member 3	Q9ET30
KTLRYNFL	8		23.8	Nucleoporin NUP188 homolog	Q6ZQH8
SNIHYHTL	8		23.9	Chromatin assembly factor 1 subunit B	Q9D0N7
KNYGFVHI	8		23.9	RNA-binding protein 4B	Q8VE92; Q8C7Q4-2; Q8C7Q4
RAFLFNKV	8		24.2	Protein yippee-like 5	P62700
VAPSFGTLL	9		24.4	Nucleoporin NUP188 homolog	Q6ZQH8

SINKFLSHL	9		24.6	Activating signal cointegrator 1 complex subunit 3	E9PZJ8-2; E9PZJ8-1
ISPCFQERL	9		24.8	FAST kinase domain-containing protein 1, mitochondrial	Q6DI86-2; Q6DI86-3; Q6DI86
RNNRFPNL	8		24.8	Pumilio homolog 2	Q80U58-3; Q80U58-2; Q80U58
TIYERFVLV	9		24.9	Crooked neck-like protein 1	P63154
KNYSFPLNNL	10		25	Deubiquitinating protein VCIP135	Q8CDG3; Q8CDG3-2
KTYQVAHM	8		25.1	Multidrug resistance-associated protein 1	O35379
IQLEFREL	8		25.1	endoplasmic reticulum mannosyl-oligosaccharide 1,2-alpha-mannosidase	A2AJ15
VNLREYPSL	9		25.2	SP110 nuclear body protein	Q8BVK9
AQYNFILV	8		25.3	Threonine--tRNA ligase, cytoplasmic	Q9D0R2; Q8BLY2
RNYNYRVV	8		25.4	DNA repair protein Rad50	P70388; P70388-2; P70388-3
IIYNPKNL	8		25.5	splicing factor 3a subunit 3	Q9D554
VAYEAPSL	8		25.6	Proteasome subunit beta type-4	P99026
AQFKFTVL	8		25.6	Proliferation-associated protein 2G4	P50580; P50580-2
MVFDFKGQL	9		25.6	Phosphatidylinositol 4,5-bisphosphate 3-kinase catalytic subunit beta isoform	Q8BTI9
SVAHFINL	8		25.8	PR domain zinc finger protein 15	E9Q8T2; E9Q8T2-2
STLRFLCKL	9		25.9	Coatomer subunit beta	Q9JIF7
VNVDYSKL	8		26	Cytochrome c oxidase subunit NDUFA4	Q62425
KSYSFDEV	8		26.2	nicotinamide phosphoribosyltransferase	Q99KQ4
CGYEFTSKL	9		26.2	Cullin-2	Q9D4H8; Q9D4H8-2
TTYVHKGL	8		26.3	Mitochondrial import receptor subunit TOM70	Q9CZW5
SAYLYKQGF	9		26.3	Transmembrane protein 41A	Q9D8U2
TTFSRLINL	9		26.6	CD180 antigen	Q62192
TGPKYIHL	8		26.7	Tyrosine-protein kinase Mer	Q60805
SAVSFTIRL	9		26.7	Patatin-like phospholipase domain-containing protein 2	Q8BJ56; Q8BJ56-2; Q8BJ56-3
KNFKFTMDL	9		26.7	Toll-like receptor 9	Q9EQU3
LVYQFKEM	8	1xOxidation [M8]	27	ETS-related transcription factor Elf-4	Q9Z2U4; Q60775
SALDFVKL	8		27	NLR family CARD domain-containing protein 4	Q3UP24
KAYIHTRM	8		27.1	Actin-related protein 2/3 complex subunit 2	Q9CVB6
STYDYGRQL	9		27.4	SEC14 domain and spectrin repeat-containing protein 1	Q80UK0
KGYAYTFI	8		27.4	Probable ATP-dependent RNA helicase DDX46	Q569Z5; Q569Z5-2
SGYKYLDYM	9		27.4	Serine beta-lactamase-like protein LACTB, mitochondrial	Q9EP89
IIQEFCQAL	9		27.5	Exportin-T	Q9CRT8
SCFSFRKL	8		27.6	natural resistance-associated macrophage protein 2	P49282
ATYSYKEAL	9		27.7	Cell division cycle protein 16 homolog	Q8R349
VIMKLFPQL	9		27.8	all-trans-retinol 13,14-reductase	Q64FW2
QNPRFSKL	8		28	HAUS augmin-like complex subunit 4	Q8BFT2
SAFTFRVTV	9		28	Kinesin-like protein KIF1B	Q60575; Q60575-2
TSIKFSFI	8		28.2	NADH-ubiquinone oxidoreductase chain 5	P03921
KTYTFDMV	8		28.3	Kinesin-like protein KIF11	Q6P9P6
RAIGFLSRL	9		28.4	Transmembrane protein 186	Q9CR76
RSPEYLSL	8		28.5	Cullin-3	Q9JLV5
IGPCFPSRL	9	Carbamidomethyl [C	28.5	Cullin-7	Q8VE73; Q8VE73-3
TTYKYEMI	8		28.6	eukaryotic translation initiation factor 3 subunit L	Q8QZY1
SQHNFNNL	8		28.6	Zinc finger CCHC domain-containing protein 2	Q69ZB8-2; Q69ZB8-1
VILESFRAL	9		28.8	Protein Abhd15	Q5F2F2
LSYSYQSRF	9		28.8	Splicing factor 3B subunit 3	Q921M3-2; Q921M3-1
RIILFDRL	8		29	TBC domain-containing protein kinase-like protein	Q8BM85-2; Q8BM85-1
VIYPMAVV	8		29.1	mRNA export factor	Q8C570
YVLHFTAL	8		29.1	Ribosome production factor 2 homolog	Q9JJ80
TIRDKFARL	9		29.3	Conserved oligomeric Golgi complex subunit 4	Q8R1U1
MPLSVFPYYPS	12		29.3	Protein mono-ADP-ribosyltransferase PARP14	Q2EMV9
FGYEYITV	8		29.3	Natural resistance-associated macrophage protein 2	P49282-4; P49282-3; P49282-2; P49282
KSYLMNKL	8		29.4	Guanylate-binding protein 5	Q8CFB4; Q01514; Q8CFB4-2; Q9Z0E6
LSYTRFSLA	9		29.5	Transferrin receptor protein 1	Q62351

KNYDFAQVL	9		29.5	PAB-dependent poly(A)-specific ribonuclease subunit PAN2	Q8BGF7-2; Q8BGF7; Q8BGF7-3
CAMIFRQL	8		29.5	Anaphase-promoting complex subunit 5	Q8BTZ4; Q8BTZ4-2
SQYEFENYM	9		29.6	Serine/threonine-protein kinase 40	Q7TNL3-1; Q7TNL3-2
IATEFNQL	8		29.7	Conserved oligomeric Golgi complex subunit 2	Q921L5
VSPRLTFL	8		30	Antigen peptide transporter 2	P36371
IAYKFGKTV	9		30.1	Store-operated calcium entry-associated regulatory factor	Q8R3Q0
VAPGYPLL	8		30.3	Embigin	P21995
SILTLSHL	8		30.3	Tubulin delta chain	Q9R1K7
ICFSFRAV	8		30.4	DDB1- and CUL4-associated factor 1	Q80TR8; Q80TR8-4; Q80TR8-2; Q80TR8-3
VQPHYPYL	8		30.5	Lactosylceramide 1,3-N-acetyl-beta-D-glucosaminyltransferase	Q8BGY6
VILSFENHV	9		30.5	1-phosphatidylinositol 4,5-bisphosphate phosphodiesterase beta-3	P51432; A3KGF7; A3KGF7-3; A3KGF7-4; A3KGF7-2
VQYDHHYL	8		30.6	Antigen peptide transporter 1	P21958
LVLLFREL	8		30.8	Ataxin-10	P28658
VITNFSARI	9		31.1	Vacuolar protein sorting-associated protein 51 homolog	Q3UVL4-1; Q3UVL4-2
RAVLFVGL	8		31.2	signal recognition particle receptor subunit beta	P47758
VNIEFKDL	8		31.2	ATP-binding cassette sub-family G member 1	Q64343
IMYDKHIQM	9		31.4	Interferon-induced very large GTPase 1	Q80SU7
TNYRFKNLF	9		31.5	G1/S-specific cyclin-E2	Q9Z238
SALAFYKNGRI	11		31.5	Complex I assembly factor TMEM126B, mitochondrial	Q9D1R1
KVLVFSQM	8		31.7	Lymphocyte-specific helicase	Q60848-2; Q60848-1
AMYIFLHTV	9		31.7	ORM1-like protein 2	Q9CQZ0
RVLLFSQM	8		31.8	Chromodomain-helicase-DNA-binding protein 1-like	Q9CXF7-2; E9PZM4; Q9CXF7-1; Q6PGB8; P40201; A2AJK6-3; Q6PDQ2; A2AJK6-1; Q91ZW3; A2A8L1; Q6PGB8-2
SSFRKADL	8		31.8	Dedicator of cytokinesis protein 6	Q8VDR9-1
VIHRFLELL	9		32.1	Mediator of RNA polymerase II transcription subunit 23	Q80YQ2-2; Q80YQ2
IAAVFHTL	8		32.3	Progressive ankylosis protein	Q9JHZ2
RGLDYYTGV	9		32.3	probable histidine--tRNA ligase, mitochondrial	Q99KK9; Q61035
TITSFPRL	8		32.3	Glutamate--cysteine ligase catalytic subunit	P97494
RQYVHPRL	8		32.4	DNA helicase MCM8	Q9CWV1-1; Q9CWV1-2
KAFDYPSRL	9		32.4	zinc finger protein 728	Q6P5C7
FIQEYSHL	8		32.8	Lysine--tRNA ligase	Q99MN1
AICVFPRL	8		32.8	Poly(A)-specific ribonuclease PARN	Q8VDG3
RGYDFCQV	8		33	EH domain-containing protein 4	Q9EQP2
RGVQYTRL	8		33	E3 ubiquitin-protein ligase HUWE1	Q7TMY8-4; Q7TMY8-3; Q7TMY8; Q7TMY8-2
STYSVAKM	8		33.1	Lysosomal alpha-mannosidase	O09159
SGPTYIKL	8		33.3	Uncharacterized aarF domain-containing protein kinase 2	Q6NSR3
HIYQFEYM	8		33.8	striatin-interacting protein 1	Q8C079-4; Q8C079
TILEFSQNM	9		34.2	Exportin-1	Q6P5F9
SGFYYKTRI	9		34.5	Nucleolar protein 10	Q5RJG1
QNYEMPNL	8	1xOxidation [M5]	34.5	Short transmembrane mitochondrial protein 1	P0DP99
NKMPLSVFPY`	14		34.6	Protein mono-ADP-ribosyltransferase PARP14	Q2EMV9
VIAGFNRL	8		34.7	Prefoldin subunit 2	O70591
IAVAYHNI	8		34.7	cell division cycle protein 23 homolog	Q8BGZ4; Q8BGZ4-2
TSMAFKNI	8		34.9	Serine/threonine-protein kinase MARK2	Q05512-2; Q05512-4; Q05512; Q05512-3
RVYEFLDKL	9		34.9	26S proteasome non-ATPase regulatory subunit 3	P14685
NIYRFIMV	8		35	Protein cornichon homolog 4	Q9CX13
QVYTFTERM	9		35.1	etyl-neuraminyl-2,3-beta-galactosyl-1,3-N-acetyl-galactosaminide alpha-2,6-sialylt	Q9R2B6-2; Q9R2B6; Q9R2B6-3
NVWRFPYL	8		35.2	Sodium- and chloride-dependent neutral and basic amino acid transporter B(0+)	P31649; Q761V0-1; Q9D687; Q9JMA9; O88575; Q8VBW1-2; P31651; Q8BG16; P31648; Q761V0-2; P28571-1; Q8VBW1-3; P31650; Q61327; O55192; Q8VDB9; O35316; P28571; Q8VBW1-1; P28571-2
KNIRFPLM	8		35.4	kelch-like protein 9	Q80TF4-2; Q80TF4-5; Q80TF4-4; Q80TF4; Q80TF4-3; Q6ZPT1
RILKFLQL	8		35.5	5'-AMP-activated protein kinase subunit gamma-2	Q91WG5-2; Q91WG5
INYSFPAKGKL	11		35.6	ATP-dependent RNA helicase DDX54	Q8K4L0
VSLDGYFHL	9		35.9	DDB1- and CUL4-associated factor 12	Q8BGZ3; Q8BGW4; Q8CBW4

HTLKFRHL	8		36	Leucine-rich repeat-containing protein 41 OS=Mus musculus OX=10090	Q8K1C9
RNLAFHTL	8		36	UPF0668 protein C10orf76 homolog	Q6PD19-1; Q6PD19-2
KVLVFNFL	8		36	Anoctamin-10	Q8BH79; Q8BH79-3; Q8BH79-2; Q8BH79-4
VITKFDHL	8		36.1	C2 domain-containing protein 3	Q52KB6-2; Q52KB6-3; Q52KB6
VQLAIFANM	9		36.1	Dolichyl-diphosphooligosaccharide--protein glycosyltransferase subunit 4	Q99LX8
RQIDYYCRL	9		36.2	adenylate cyclase type 7	P51829
AQYSFDKL	8		36.3	HEAT repeat-containing protein 5B	Q8C547; Q8C547-2
QNYLFGCEL	9	Carbamidomethyl [C	36.3	Nucleophosmin	Q61937
SVVRFIARF	9		36.4	Myotubularin-related protein 13	E9PXF8-2; E9PXF8
VAASFKGL	8		36.5	RRP12-like protein	Q6P5B0
AIIAFKTL	8		36.5	Rho guanine nucleotide exchange factor 6	Q8K4I3
SIFEFVHA	8		36.6	Importin-9	Q91YE6
RFLSFSSL	8		36.6	Nesprin-1	Q6ZWR6-5; Q6ZWR6-4; Q6ZWR6
VQYEMRTL	8		36.7	28S ribosomal protein S10, mitochondrial	Q80ZK0
QVVQFNRL	8		36.9	ragulator complex protein LAMTOR3	O88653
RNNRYPNL	8		37	Pumilio homolog 1	Q80U78-2; Q80U78-1; Q80U78-3
VNYEPLGL	8		37	Death-associated protein kinase 1	Q80YE7; Q8VDF3; Q8VDF3-2; O54784; Q80YE7-2
NTYKYAKI	8		37.2	Ankyrin repeat and BTB/POZ domain-containing protein 2	Q7TQI7
ATRSFPQL	8		37.4	Annexin A7	Q07076
RGYDFCQVL	9		37.4	EH domain-containing protein 4	Q9EQP2
TNQEYARM	8		37.5	Hyaluronan mediated motility receptor	Q00547-2; Q00547-1
RIYRMIKL	8		37.5	Heat shock protein HSP 90-beta	P07901; P11499
VHIRMAFL	8		37.6	Protein lifeguard 4	Q9DA39
SAAKFRQL	8		38.1	toll-like receptor 6	Q9EPW9
INLNYKDL	8		38.1	Translocon-associated protein subunit alpha	Q9CY50
YGLAYRSL	8		38.5	DENN domain-containing protein 4C	A6H8H2-1; A6H8H2-2
TCLCFARL	8		38.7	E3 ubiquitin-protein ligase TRIP12	G5E870
KNWQFVENL	9		38.7	Ribonuclease H2 subunit A	Q9CWY8
KAYRFLFGF	9		38.8	Protein jagunal homolog 1	Q5XKN4
KSFEWLSQM	9		38.9	Cytoplasmic dynein 1 heavy chain 1	Q9JHU4
ATYIFLQTF	9		39	Transmembrane protein 41B	Q8K1A5; Q8K1A5-2; Q8K1A5-3
SITQFIRNL	9		39.1	Tankyrase-2	Q3UES3
SGLKYVAV	8		39.3	Flavin reductase (NADPH)	Q923D2
VAFDRHLYV	9		39.3	Leucine-zipper-like transcriptional regulator 1	Q9CQ33-2; Q9CQ33
RNPTFILL	8		39.4	solute carrier organic anion transporter family member 4A1	Q8K078-2; Q8K078
ALVRFVNL	8		39.5	Ribosomal biogenesis protein LAS1L	A2BE28-2; A2BE28
INVFFYTV	8		39.6	Phosphatidylinositol glycan anchor biosynthesis class U protein	Q8K358
VSFIRTIQM	9		39.7	Unconventional myosin-Va	Q99104
VVYIYKEHF	9		39.8	Small subunit processome component 20 homolog	Q5XG71
VIVRFLTV	8		40	40S ribosomal protein S15a	P62245
INQRFEEL	8		40.2	Signal transducer and activator of transcription 5A	P42230
LGPYPYATL	9		40.2	Protein AAR2 homolog	Q9D2V5
RAYFFVEV	8		40.5	General transcription factor II-I repeat domain-containing protein 2	Q99NI3
INAEFVTQL	9		40.7	Intron-binding protein aquarius	Q8CFQ3
IGYGYLHRI	9		40.8	Golgi reassembly-stacking protein 2	Q99JX3; Q99JX3-2; Q91X51
TSPEYQKL	8		40.9	Alsin	Q920R0-1
EIFRFYKL	8		41.2	Coronin-2A	Q8C0P5
VNLKYLLKL	9		41.2	Poly [ADP-ribose] polymerase 1	P11103-2; P11103
KIIDFGFARL	10		41.3	Ribosomal protein S6 kinase alpha-4	Q9Z2B9
RVLIFSQM	8	1xOxidation [M8]	42.3	F-related matrix-associated actin-dependent regulator of chromatin subfamily A m	E9PZM4; Q6PGB8; P40201; A2AJK6-3; Q6PDQ2; A2AJK6; Q91ZW3; A2A8L1; Q6PGB8-2
TGLRYNMRL	9		42.4	UDP-glucose:glycoprotein glucosyltransferase 1	Q6P5E4
QPYLWARL	8		42.7	Uncharacterized protein C12orf29 homolog	Q8BHN7; Q8BHN7-2
VNFEFPEF	8		43.3	40S ribosomal protein S7	P62082

VGPKFRGV	8	43.4	Protein AAR2 homolog	Q9D2V5
MVVEFRHM	8	44	V-type proton ATPase subunit d 1	P51863
RTFSWASV	8	44.1	Ras GTPase-activating protein-binding protein 1	P97855
VTYPQPRL	8	44.2	alpha-ketoglutarate-dependent dioxygenase alkB homolog 3	Q8K1E6
VAFLHKQQM	9	44.4	UV radiation resistance associated protein	Q8K245
SRIVFRHL	8	44.5	28S ribosomal protein S14, mitochondrial	Q9CR88
RAYSFKVVL	9	44.5	Ras-related protein Rab-21	P35282
SNPEFSSV	8	44.6	Talin-1	P26039
INQIYEARV	9	44.6	Arf-GAP with coiled-coil, ANK repeat and PH domain-containing protein 1	Q8K2H4
SNQRYSLV	8	44.7	ALS2 C-terminal-like protein	Q60I26-3; Q60I26-2; Q60I26-1
NVFQYLTRL	9	44.8	Nucleoporin NUP188 homolog	Q6ZQH8
IWIRVASL	8	44.9	Kelch-like protein 24	Q8BRG6
STVEFTCL	8	46	Rho guanine nucleotide exchange factor 6	Q8K4I3
RVFQFLVL	8	46	39S ribosomal protein L37, mitochondrial	Q921S7
VGINYREV	8	46.2	DNA topoisomerase 2-alpha	Q01320
AIYEFIHNF	9	46.3	AP-4 complex subunit sigma-1	Q9WVL1
AIYYFKIAV	9	46.7	Sarcoplasmic/endoplasmic reticulum calcium ATPase 2	O55143-2; O55143; Q8R429
SLILFSTRL	9	47	Baculoviral IAP repeat-containing protein 1b	Q9QUK4
SANKFENL	8	47.3	CD180 antigen	Q62192
RGLEYLYL	8	47.3	Cytoplasmic dynein 1 light intermediate chain 1	Q8R1Q8; Q6PDL0
VINSFVHV	8	47.4	Cullin-2	Q9D4H8; Q9D4H8-2
SILRFITI	8	47.5	Myeloid differentiation primary response protein MyD88	P22366; P22366-2
KVYKYVFI	8	47.6	Monocarboxylate transporter 4	P57787
SIAAFIQRL	9	47.7	Large proline-rich protein BAG6	Q9Z1R2
SILALTHL	8	47.9	RRP12-like protein	Q6P5B0
IDFDFTHL	8	48.1	Protein diaphanous homolog 2	O70566
IPYDFNRV	8	48.1	Receptor-type tyrosine-protein phosphatase epsilon	P49446-3; P49446-2; P49446-1
SVFEKYFQF	9	48.1	Actin-related protein 2/3 complex subunit 2	Q9CVB6
VWYWRRITM	9	48.3	Protein wntless homolog	Q6DID7; Q6DID7-2
SIYDAFPKV	9	48.5	Microtubule-actin cross-linking factor 1	Q9QXZ0-3; Q91ZU6-8; Q91ZU6-3; Q91ZU6-4; Q91ZU6-2; Q9QXZ0-2; Q9QXZ0-4; Q91ZU6-6; Q9QXZ0; Q91ZU6
IQFSFKEKL	9	48.8	protein dopey-1	Q8BL99-1; Q8BL99-6; Q8BL99-5
VNKNYLYL	8	48.9	Tumor protein D52	Q62393-1
QNHVFPLL	8	48.9	Importin-8	Q7TMY7-2; Q7TMY7
RNQVYTQL	8	49	Melanoma inhibitory activity protein 2	Q91ZV0; Q8R311
VRVFFSGL	8	49	RNA-binding protein 12B-B	Q80YR9; Q66JV4-1
VMFKKVHGL	9	49.1	Secretory carrier-associated membrane protein 1	Q8K021
KQFSYTHI	8	49.5	Electron transfer flavoprotein subunit alpha, mitochondrial	Q99LC5
VNRVFDKL	8	49.7	Proteasome subunit beta type-9	O35522; P28076
NTYMHLHL	8	49.7	Adhesion G protein-coupled receptor E1	Q61549
VSMDFVQRF	9	49.8	Conserved oligomeric Golgi complex subunit 2	Q921L5
RIYQFTAA	8	49.9	src kinase-associated phosphoprotein 2	Q3UND0-2; Q3UND0-1
VNFGFSKF	8	50.9	Double-strand break repair protein MRE11	Q61216-1; Q61216-2
VNYDFGHMHV	11	51.2	E3 ubiquitin-protein ligase RNF216	P58283-2; P58283
VWMPFVRNV	9	51.3	Tuftelin-interacting protein 11	Q9ERA6
EGLRWYASL	9	51.6	Translocator protein	P50637
RVLGFCHL	8	52.1	Sodium/potassium-transporting ATPase subunit alpha-1	Q8VDN2; Q9Z1W8
VNFRYLIKF	9	52.5	Rho GTPase-activating protein 17	Q3UIA2-2; Q3UIA2-4; Q3UIA2-3; Q3UIA2
IFYYVQKL	8	52.7	E3 ubiquitin-protein ligase HECTD1	Q69ZR2
RAFSFLNEV	9	52.9	vesicle-associated membrane protein 7	P70280
TVYDFWRM	8	53.1	Receptor-type tyrosine-protein phosphatase kappa	P35822
KTFQKLSNL	9	53.5	WD repeat-containing protein 43	Q6ZQL4
SSPKFSEI	8	53.5	WD repeat domain phosphoinositide-interacting protein 4	Q91VM3-2; Q91VM3
SIYAREALI	9	53.6	structural maintenance of chromosomes protein 1a	Q9CU62

SAPWYLNRV	9	53.6	Beta-hexosaminidase subunit alpha	P29416
KNLDWFPRM	9	54.3	Inositol 1,4,5-trisphosphate receptor type 2	Q9Z329-1; Q9Z329-3; P70227; Q9Z329-2
VQYLYRVF	8	54.4	probable ATP-dependent RNA helicase DDX10	Q80Y44
SCFEYQKL	8	54.6	dual specificity tyrosine-phosphorylation-regulated kinase 3	Q922Y0
VNCPFISTL	9	55.2	Transcription regulator protein BACH1	P97302
NIFMFSKV	8	55.6	Thioredoxin-related transmembrane protein 2	Q9D710
IGPCFPNRL	9	55.7	Cullin-9	Q80TT8; Q80TT8-2
SAPRFLTAF	9	56.3	Post-GPI attachment to proteins factor 2	Q3TQR0; Q3TQR0-3; Q3TQR0-2
FSPVYRCL	8	56.7	Exocyst complex component 6	Q8R313; A6H5Z3
SAYEVIKL	8	56.8	L-lactate dehydrogenase B chain	P16125; P06151
CVYEFRDKL	9	57.3	Heat shock protein 105 kDa	Q61699
SNYLHRVV	8	57.5	F-box only protein 22	Q78JE5
RSPKYLEL	8	58	Myb-binding protein 1A	Q7TPV4
SAYDPSNL	8	58.1	Vacuolar protein sorting-associated protein VTA1 homolog	Q9CR26
NQYKFILL	8	58.2	Inositol hexakisphosphate kinase 2	Q80V72
RNFDYHVL	8	58.2	Prostaglandin G/H synthase 1	P22437
KQYEAARL	8	58.4	Arfaptin-2	Q8K221
QQYVFINQM	9	58.4	volume-regulated anion channel subunit LRRC8D	Q8BGR2
TIILFTKV	8	58.7	Importin-11	Q8K2V6-2; Q8K2V6
LVAIFTHL	8	58.8	Cytoplasmic dynein 1 heavy chain 1	Q9JHU4
HVFTFTIL	8	59.1	Ribosome biogenesis protein BRX1 homolog	Q9DCA5
SVVTFSVHM	9	59.2	Multidrug resistance-associated protein 5	Q9R1X5
RIFEFQVM	8	59.4	Programmed cell death protein 2	P46718
PTYIYRLL	8	59.4	Ubiquitin carboxyl-terminal hydrolase 10	P52479; P52479-2
QQYRFSVI	8	59.4	Inverted formin-2	Q0GNC1; Q0GNC1-3
SALRFLDM	8	59.4	Transforming growth factor beta activator LRRC33	Q8BMT4; Q8BMT4-2; Q8BMT4-3
KIHIFKYL	8	59.7	Leucine-rich repeat and calponin homology domain-containing protein 3	Q8BVU0
SLVKYVPL	8	60.2	Sodium- and chloride-dependent taurine transporter	O35316
RAPSYRTL	8	60.3	Suppression of tumorigenicity 5 protein	Q924W7-2; Q924W7; Q924W7-3
RIVELFRNL	9	60.4	Signal transducer and activator of transcription 3	P42227-3; P42227; P42227-2
INFDFHQF	8	60.9	Synaptojanin-2	Q9D2G5-4; Q9D2G5-6; Q9D2G5-5; Q9D2G5; Q9D2G5-2; Q9D2G5-3
KTVEYHRL	8	61.1	Keratinocyte-associated transmembrane protein 2	Q8K201
TQYIFTEKL	9	61.1	H/ACA ribonucleoprotein complex non-core subunit NAF1	Q3UMQ8
RTLIYITL	8	61.4	Actin-related protein 2/3 complex subunit 3	Q9JM76
VGPRFELKL	9	61.7	U3 small nucleolar ribonucleoprotein protein IMP4	Q8VHZ7
VWLEAARL	8	61.9	Pre-mRNA-processing factor 6	Q91YR7; Q91YR7-2
SVVSFRLV	8	61.9	cleavage stimulation factor subunit 2 tau variant	Q8C7E9
SVNIFRTL	8	62.6	Serine/threonine-protein phosphatase 2A 56 kDa regulatory subunit beta isoform	Q6PD28
KIFEFKETL	9	63.4	calcium/calmodulin-dependent protein kinase type 1D	Q8BW96-2; Q8BW96
TTYVFLKF	8	63.8	ATP-binding cassette sub-family B member 6, mitochondrial	Q9DC29
SVILMQHL	8	63.8	Transmembrane protein 39B	Q810L4
TNLIYQQV	8	63.8	Unconventional myosin-VIIa	P97479-1; P97479-2
SNYERLESL	9	64.4	perilipin-2	P43883
KAYSFKEQI	9	64.5	eIF-2-alpha kinase activator GCN1	E9PVA8
AVCTFIHL	8	64.7	3-beta-hydroxysteroid-delta(8),delta(7)-isomerase	P70245
CSYDFTVRF	9	64.9	Peroxisomal targeting signal 2 receptor	P97865
RAPAFHQL	8	65	Zinc finger SWIM domain-containing protein 8	Q3UHH1-2; Q3UHH1; Q3UHH1-3
KVYMFKCV	8	65.2	Lysosomal-associated transmembrane protein 5	Q61168
TGRSYTSL	8	65.2	Endothelial protein C receptor	Q64695
KVVEFSEL	8	65.2	nucleolar MIF4G domain-containing protein 1	Q3UFM5
LIYTFTTTF	9	65.6	Tripartite motif-containing protein 26	Q99PN3
QQYSFINQM	9	65.7	volume-regulated anion channel subunit LRRC8C	Q8R502
KILTFDQL	8	66.5	60S ribosomal protein L18	P35980
VNFEKFWEL	9	66.6	Ras-GEF domain-containing family member 1B	Q8JZL7-2; Q8JZL7

RNPTFMCL	8		66.8	AP-2 complex subunit alpha-1	P17426
RNLQFVGV	8		66.8	KN motif and ankyrin repeat domain-containing protein 2	Q8BX02; Q8BX02-2
VNVCYKEL	8		66.9	THO complex subunit 5 homolog	Q8BKT7
SAPTFINF	8		67.5	Isoform 2 of Magnesium transporter protein 1	Q9CQY5-2; Q9CQY5-3; Q9CQY5
ATQQFQQL	8		67.6	Retrovirus-related Env polyprotein from Fv-4 locus	P11370
RIFKHNNM	8		67.7	Serine palmitoyltransferase 2	P97363
INVAFSCV	8		67.8	Fermitin family homolog 3	Q8K1B8
TQFLYPKV	8		68.3	Transcription factor jun-D	P15066
VISDFITRL	9		68.6	Sterol 26-hydroxylase, mitochondrial	Q9DBG1
SGIDFKQL	8		68.8	Integrin-linked protein kinase	O55222
IVIPFKCL	8		68.8	Amyloid-like protein 2	Q06335-2; Q06335
SCYAFLQV	8		69	CD302 antigen OS=Mus musculus OX=10090	Q9DCG2
VNVRFSTIV	9		69.5	Dual specificity protein phosphatase 1	P28563
SNPEYAKI	8		69.8	Zinc finger protein 318 OS=Mus musculus OX=10090	Q99PP2; Q99PP2; Q99PP2-2
TIVVFNGM	8	1xOxidation [M8]	70.2	Poly [ADP-ribose] polymerase 9	Q8CAS9-2; Q8CAS9
SRIVFIPL	8		70.4	Equilibrative nucleoside transporter 1	Q9JIM1; Q9JIM1-2
AVIKFLEL	8		70.5	DNA mismatch repair protein Msh2	P43247
IITGFRNV	8		70.6	Mitotic checkpoint serine/threonine-protein kinase BUB1 beta	Q9Z1S0
TSLKYLEM	8		70.9	biogenesis of lysosome-related organelles complex 1 subunit 2	Q9CWG9
KVAVFRYL	8		71.1	Piezo-type mechanosensitive ion channel component 1	E2JF22
LNFEFQIV	8		71.5	E3 SUMO-protein ligase RanBP2	Q9ERU9
KVVKFSYM	8		71.7	Speckle-type POZ protein	Q6ZWS8; Q2M2N2
TAPHYQLL	8		72	WD repeat-containing protein 20	Q9D5R2
VAHTFVIGV	9		72.7	Proliferation-associated protein 2G4	P50580; P50580-2
LIYKFSQC	8		73.1	Tripartite motif-containing protein 34A	Q99PP6-1
KTFRKSFNL	9		74	Suppressor of cytokine signaling 6	Q9JLY0
VQQYYRKL	8		74.1	Phosphatidylinositol 3,4,5-trisphosphate-dependent Rac exchanger 1 protein	Q69ZK0; Q69ZK0-2
LQVIFGHL	8		74.1	Probable ubiquitin carboxyl-terminal hydrolase FAF-X	P70398
SLIEFFNKM	9		74.7	Retinoblastoma-like protein 2	Q64700
VNLVFEKI	8		74.7	Cingulin-like protein 1	Q6AW69-4; Q6AW69-5; Q6AW69; Q6AW69-3
RNYIHRDL	8		74.9	Tyrosine-protein kinase HCK	P08103-1; P08103-2
KVALFNRL	8		75.2	Recombining binding protein suppressor of hairless	P31266-2; P31266
VRYVLPRL	8		75.6	Son of sevenless homolog 2	Q02384
QIYDIFQKL	9		76.4	Eukaryotic initiation factor 4A-I	P60843
VAYWRQAGLS	14		76.9	ATP synthase subunit epsilon, mitochondrial	P56382
RVMEYINRL	9		78.4	Clathrin heavy chain 1	Q68FD5
IIFQFTKC	8		78.6	Tyrosine-protein kinase JAK2	Q62120
RGPTYVNM	8		78.8	Arf-GAP domain and FG repeat-containing protein 1	Q8K2K6-4; Q8K2K6-3; Q8K2K6-1; Q8K2K6-2
RNLEFHEL	8		79.3	Breast carcinoma-amplified sequence 3 homolog	Q8CCN5-2; Q8CCN5-1
IFYFVNKL	8		79.5	Transmembrane 9 superfamily member 4	Q8BH24
VQQFYSHV	8		79.8	EMILIN-2	Q8K482
CQYKFDFL	8		80.1	Dedicator of cytokinesis protein 10	Q8BZN6-3; Q8BZN6-4; Q8BZN6; Q8BZN6-2
KVPTFVRM	8		80.2	Phosphatidylinositol transfer protein alpha isoform	P53810
VQALARFSLL	10		80.9	Brefeldin A-inhibited guanine nucleotide-exchange protein 2	A2A5R2
SSFRFWQA	8		81.4	Short transient receptor potential channel 4-associated protein	Q9JLV2; Q9JLV2-2
VNYHFTRQC	9		81.7	Radical S-adenosyl methionine domain-containing protein 2	Q8CBB9
SVILFKGI	8		81.7	Dedicator of cytokinesis protein 9	Q8BIK4-2; Q8BIK4
TSIQFNLRNL	10		81.8	Patatin-like phospholipase domain-containing protein 2	Q8BJ56; Q8BJ56-2; Q8BJ56-3
RVFLFEQI	8		81.9	Rho guanine nucleotide exchange factor 25	A2CG49-2; A2CG49-4; Q0KL02-4; Q9CWR0; A2CG49; A2CG49-6; A2CG49-7; Q9CWR0-2; Q0KL02; Q0KL02-3
VGFSDFPKL	9		82.1	Olfactory receptor 1440	Q8VFV4
SVVLMSHL	8		82.6	Phosphoglycerate kinase 2	P09041; P09411
VNIRLVEL	8		82.8	E3 SUMO-protein ligase RanBP2	Q9ERU9
AIMKFHSM	8		82.8	DNA repair protein Rad50	P70388; P70388-2; P70388-3

KNIDRFIPV	9	82.8	Protein YIF1B	Q9CX30-1; Q9CX30-2
ATYIHNCL	8	83.1	Vacuolar protein sorting-associated protein VTA1 homolog	Q9CR26
KNYIHRDL	8	83.5	Tyrosine-protein kinase Lyn	P25911-1; P25911-2
VNFEFPEFQL	10	83.8	40S ribosomal protein S7	P62082
RIVRFLEL	8	83.9	ATP-binding cassette sub-family D member 4	O89016
ATLAYKKL	8	84	Dedicator of cytokinesis protein 2	Q8C3J5
IIYDRKFLM	9	84.1	Eukaryotic translation initiation factor 4E-binding protein 1	Q60876
SMYVPGKL	8	85.4	prefoldin subunit 5	Q9WU28
SLVTFRTL	8	85.4	plectin	Q9QXS1-6; Q9QXS1-13; Q9QXS1-7; Q9QXS1-9; Q9QXS1-5; Q9QXS1-14; Q9QXS1-12; Q9QXS1-3; Q9QXS1-2; Q9QXS1-10; Q9QXS1-15; Q9QXS1-11; Q9QXS1-16; Q9QXS1-4; Q9QXS1-1; Q9QXS1-8
RIMEFTTTL	9	85.7	DNA-dependent protein kinase catalytic subunit	P97313-1
VFYEREVQM	9	86.2	V-type proton ATPase subunit d 2	Q80SY3
KNFTYSDTNL	10	86.3	Fanconi anemia group J protein homolog	Q5SXJ3
VNYKMKSV	8	86.6	cleft lip and palate transmembrane protein 1-like protein	Q8BXA5
RLYEFSCRM	9	86.9	Kelch-like protein 25 OS=Mus musculus OX=10090	Q8R2P1
AFYYIHNL	8	87.6	Alpha-adducin	Q9QYC0-1; Q9QYC0-2
RSYHFGIVGL	10	88.5	Protein O-linked-mannose beta-1,2-N-acetylglucosaminyltransferase 1	Q91X88-2; Q91X88-1; Q91X88-3
TNPSFDGRL	9	88.6	Atlastin-2	Q6PA06-2; Q6PA06-1
KTYQFLNDI	9	89.3	ribosomal RNA processing protein 36 homolog	Q3UFY0
KIYQWINEL	9	89.5	CCR4-NOT transcription complex subunit 9	Q9JKY0
VFQYLTRL	8	89.6	Nucleoporin NUP188 homolog	Q6ZQH8
VAYSHDGAFL	10	89.7	WD repeat-containing protein 1	O88342
SIYEKLIQF	9	89.8	SAP30-binding protein	Q02614
INRIFHERF	9	89.8	Dynamin-2	P39053; P39053-3; P39054-1; P39053-4; P39054-2; Q8BZ98; Q8BZ98-2; P39053-5; P39053-6
KTFTFDTV	8	90.2	kinesin-like protein KIF3A	P28741
IIFNFEKAYF	10	90.2	AP-1 complex subunit sigma-3	Q7TN05; P61967; Q9DB50
HTYDFEKL	8	90.4	Ribonucleoside-diphosphate reductase large subunit	P07742
TNVDFPSL	8	90.7	SHC SH2 domain-binding protein 1	Q9Z179
IIWAFKHTM	9	90.8	Exportin-1	Q6P5F9
SQVIFHLL	8	91.1	Nucleoporin Ndc1	Q8VCB1
TIVSFRSANL	10	91.7	Isoform HK1 of Hexokinase-1	P17710-1; P17710-2; P17710-4; P17710-3; Q91W97
SFTSFQGL	8	91.8	Glucagon-like peptide 1 receptor	O35659
RNPQFQKL	8	93.2	glucose-6-phosphate isomerase	P06745
SIIRYAIGV	9	93.3	Integrin alpha-X	Q9QXH4
VLRSFYEL	8	95.1	Dipeptidase 3	Q9DA79
SCLQFIGL	8	95.1	Inhibitor of Bruton tyrosine kinase	Q6ZPR6; Q6ZPR6-2
KSVLFVCL	8	95.4	Low molecular weight phosphotyrosine protein phosphatase	Q9D358-2; Q9D358
AEYIYPSL	8	95.4	Histone lysine demethylase PHF8	Q80TJ7-2; Q80TJ7
TTVAFTQV	8	95.5	60S ribosomal protein L7a	P12970
VIVRFLTVM	9	96.4	40S ribosomal protein S15a	P62245
SGPRYQGV	8	97.2	Spermatogenesis-associated serine-rich protein 2	Q8K1N4-1
VRFKHRYL	8	97.3	Ribonuclease P/MRP protein subunit POP5	Q9DB28
VNWEKHVLI	9	97.3	Synaptophysin-like protein 1	O09117-2; O09117-1
SGLIFNKV	8	97.9	Surfeit locus protein 6	P70279
SNYQHITNF	9	98.7	AP-3 complex subunit delta-1	O54774
KSPEYESL	8	99.2	Cytosolic purine 5'-nucleotidase	Q3V1L4
SRFIFNYV	8	100.1	Polycomb protein Suz12	Q80U70
TCYKFLKHM	9	100.5	Lysosomal-associated transmembrane protein 5	Q61168
IAVQFVDM	8	100.5	D-3-phosphoglycerate dehydrogenase	Q61753
VADKFSEL	8	100.9	;erine/threonine-protein phosphatase 2A 65 kDa regulatory subunit A beta isoforn	Q7TNP2
QAFDFEFTHV	10	101.1	Vacuolar protein sorting-associated protein 26B	Q8C0E2-2; Q8C0E2

IIYEFESSTQM	11	1xOxidation [M11]	101.2	GON-4-like protein	Q9DB00
RSYERFTTC	9		101.4	Polyamine-modulated factor 1 OS=Mus musculus OX=10090	Q9CPV5
VNWDFVEQV	9		101.4	RING finger protein 10	Q3UIW5-1; Q3UIW5-2
RAPLFKFL	8		101.4	E3 ubiquitin-protein ligase RNF213	E9Q555
SYEFVQRL	8		102.7	Cytoplasmic dynein 1 heavy chain 1	Q9JHU4
LNFDFTRSYL	10		103	Nck-associated protein 1-like	Q8K1X4
RSPVYSHF	8		103.1	Multidrug resistance-associated protein 1	O35379
SVLLFIEHV	9		103.5	Lysosomal-associated transmembrane protein 5	Q61168
SQYRFIVF	8		106.1	Embigin	P21995
KTLNYTARF	9		106.4	DNA-directed RNA polymerase II subunit RPB4	Q9D7M8
KTYCYDLRM	9		106.4	Mitochondrial carrier homolog 2	Q791V5
TGIKFVVL	8		107.1	Trafficking protein particle complex subunit 4	Q9ES56
TTPEFLTRI	9		107.2	Prostaglandin G/H synthase 2	Q05769
KVLRFIAEV	9		107.3	Melanoma-associated antigen D1	Q9QYH6
VCFSYRNNF	9		107.5	Membrane-bound transcription factor site-2 protease	Q8CHX6
HSALIYSNL	9		107.8	Trafficking protein particle complex subunit 3	O55013
KILPWFEQL	9		108.6	Ran-binding protein 6	Q8BIV3
KQYLFPETL	9		108.7	Alpha-1,6-mannosyl-glycoprotein 2-beta-N-acetylglucosaminyltransferase	Q921V5
TCFLFSTV	8		110.6	Upstream-binding protein 1	Q811S7; Q811S7-2
TNVTFSKV	8		111.7	RRP12-like protein	Q6P5B0
VNLVFPRC	8		111.8	Transmembrane glycoprotein NMB	Q99P91
VGYRQPLV	8		112.4	sodium/potassium-transporting ATPase subunit beta-3	P97370
TICRFESL	8		112.5	POU domain, class 4, transcription factor 2	Q63934; Q63934-2; P17208; P17208-2; Q63955
SIIIFTNT	8		113	Probable ATP-dependent RNA helicase DDX49	Q4FZF3
AVVEFSRNV	9		113.2	CTP synthase 1	P70698
AQYLFIQTF	9		114	Ribosomal RNA processing protein 1 homolog B	Q91YK2
TVVNFLIRV	9		114	Transformation/transcription domain-associated protein	Q80YV3
SCPLFVRRL	9		114.7	Transmembrane protein 33	Q9CR67
SKYLHRQL	8		114.8	2'-5'-oligoadenylate synthase 1A	P11928
SNIQYITRF	9		115	Rab5 GDP/GTP exchange factor	Q9JM13
RVDVFTNL	8		115.5	THO complex subunit 6 homolog	Q5U4D9
ITGYFPSM	8		115.6	Neutrophil cytosol factor 1	Q09014
RNVRYVHI	8		116.5	U7 snRNA-associated Sm-like protein LSm10	Q8QZX5
NTHEFVNL	8		116.6	Vacuolar protein sorting-associated protein 26A	P40336; P40336-2
TCFIFSMV	8		117	Keratinocyte-associated protein 2	Q5RL79
ATQVYPKL	8		118.9	BRISC and BRCA1-A complex member 2	Q8K3W0-4; Q8K3W0-2; Q8K3W0-1; Q8K3W0-6; Q8K3W0-5
KALTYEKL	8		119.7	Serpin B8	O08800
CAPLFRNI	8		120.5	Sterol O-acyltransferase 1	Q61263
VRYIHVEL	8		120.7	E3 ubiquitin-protein ligase HERC2	Q4U2R1-2; Q4U2R1
VIFNYKGKNV	10		122.2	Calreticulin	P14211
KVIEFKKL	8		122.5	Sodium/hydrogen exchanger 8	Q8R4D1; Q8R4D1-2
ICLEYPLL	8		123.3	TFIIH basal transcription factor complex helicase XPB subunit	P49135
SAYNYAEQTM	10		123.5	RNA-binding protein 4B	Q8VE92
VSPIILRL	8		124.3	Integrin alpha-M	P05555; P05555-2
VGLRYEKI	8		124.7	Serine/threonine-protein kinase TBK1	Q9WUN2
NSPEYQRL	8		126.3	Ataxin-3	Q9CVD2
SLLYFINNM	9		126.4	Nucleoporin NDC1	Q8VCB1
SWLYASRL	8		128.4	NADH dehydrogenase [ubiquinone] 1 alpha subcomplex subunit 10, mitochondria	Q99LC3
IIWVKTNQL	9		130.8	N6-adenosine-methyltransferase subunit METTL3	Q8C3P7-1; Q8C3P7-2
IPPEYRHL	8		132.1	Calpastatin	P51125-4; P51125-7; P51125-6; P51125-5; P51125-1; P51125-3; P51125-2
KNLMFLVL	8		132.1	Asparagine--tRNA ligase, cytoplasmic	Q8BP47
VNIDYTISF	9		132.2	Tripeptidyl-peptidase 2	Q64514; Q64514-2
STFDHPEL	8		132.3	T-complex protein 1 subunit beta	P80314

RVAEFTTNL	9	132.4	Myosin-9	Q8VDD5
NTYSYQKV	8	134.4	JmjC domain-containing protein 8 OS=Mus musculus OX=10090	Q3TA59; Q3TA59
SGPLEYPQL	9	135	H-2 class II histocompatibility antigen gamma chain	P04441; P04441-2
KVITFIDL	8	135.5	GTP-binding protein 1	O08582
QVMWFEKL	8	135.7	Pecanex-like protein 3	Q8VI59-2; Q8VI59
VNIKLNQL	8	136	Cap-specific mRNA (nucleoside-2'-O-)-methyltransferase 1	Q9DBC3
VQFDFKGAPV	10	136.5	Unconventional myosin-Ic	Q9WTI7; Q9WTI7-3; Q9WTI7-2; Q9WTI7-4
TNIDFAFKRL	10	136.9	Beta-hexosaminidase subunit alpha	P29416
TNRKYPKL	8	137.7	DNA-directed RNA polymerase III subunit RPC10	Q9CQZ7
SSGDFPSL	8	138.2	EH domain-containing protein 1	Q9WVK4
KIVPFFKL	8	139.2	Cytoplasmic dynein 1 heavy chain 1	Q9JHU4
QIIPFKTL	8	139.6	Ribosomal L1 domain-containing protein 1	Q8BVY0
ICYIFHETF	9	140.3	Dynamin-1-like protein	Q8K1M6-1
IWISKLPHF	9	140.7	tudor domain-containing protein 7	Q8K1H1
VADKFTEL	8	140.9	erine/threonine-protein phosphatase 2A 65 kDa regulatory subunit A alpha isoforn	Q76MZ3
LARIFSIL	8	141.1	ATP-binding cassette sub-family A member 1	P41233
SFYEHIITV	9	141.5	DDB1- and CUL4-associated factor 12	Q8BGZ3
VNVERYKQI	9	142.3	Vacuolar protein sorting-associated protein 35	Q9EQH3
VVDIFRKL	8	142.8	H(+)/Cl(-) exchange transporter 3	Q61418; P51791-2; Q9WVD4; P51791-1
TSVRFDSV	8	143.1	ATPase family AAA domain-containing protein 2	Q8CDM1
RVISFIRQM	9	143.8	Cytoplasmic dynein 1 heavy chain 1	Q9JHU4
KNYSYLHC	8	145.8	Chromodomain-helicase-dna-binding protein 8	Q09XV5; Q8BYH8
TCFVFKEL	8	146	Protein FAM111A	Q9D2L9
EIISFQHL	8	146.9	Volume-regulated anion channel subunit LRRC8B	Q5DU41; Q80WG5; Q8BGR2; Q5DU41-2
KNFDKLSFL	9	146.9	coatomer subunit alpha	Q8CIE6
SHYDFGLRAL	10	147.1	Cytoplasmic dynein 1 heavy chain 1	Q9JHU4
AILERFPTI	9	147.7	Fanconi anemia group C protein homolog	P50652
TNQDFIQRL	9	149.4	Nischarin	Q80TM9-3; Q80TM9-2; Q80TM9-1
IWITAAKL	8	150	Pre-mRNA-processing factor 6	Q91YR7; Q91YR7-2
KIYVYWEV	8	150.8	KICSTOR complex protein ITFG2	Q91WI7; Q91WI7-2
SVSSFPKL	8	151.1	Phosphatidylinositol 3,4,5-trisphosphate 5-phosphatase 1	Q9ES52-5; Q9ES52-2; Q9ES52-3; Q9ES52-1; Q9ES52-6
KGIIYRDL	8	152.1	Protein kinase C eta type	P68404; P23298; P28867-2; Q8BSK8; Q8BSK8-2; P68404-2; P0C605-2; P0C605-1; P28867-1
STCEFVRTL	9	154.9	F-box/WD repeat-containing protein 1A	Q3ULA2-1; Q5SRY7
VMFKKIKSF	9	155.2	thioredoxin-interacting protein	Q8BG60; Q8BG60-2
VWYRVIQI	8	155.6	AP-2 complex subunit alpha-2	P17426-2; P17427; P17426
GNYKWINYL	9	156.6	Long-chain-fatty-acid--CoA ligase 4	Q9QUJ7-1; Q9QUJ7-2
RIIKNFERL	9	158.6	NADP-dependent malic enzyme	P06801
AICIFREL	8	158.9	FACT complex subunit SSRP1	Q08943
FSPSFINHI	9	161.1	fas-activated serine/threonine kinase	Q9JIX9
RIYKYIHKV	9	162.1	Methylsterol monooxygenase 1	Q9CRA4
SRAVFSNL	8	162.8	H2.0-like homeobox protein	Q61670
RAPKFTQV	8	164.9	Discoidin, CUB and LCCL domain-containing protein 2	Q91ZV3
VNRKFGNRI	9	165.8	protein O-mannosyl-transferase 2	Q8BGQ4-3; Q8BGQ4-2; Q8BGQ4
VQQYYRVL	8	168.9	eIF-2-alpha kinase activator GCN1	E9PVA8
SRLPFTAL	8	169.7	Proteasome subunit beta type-10	O35955
VTFERVEQM	9	169.8	Transient receptor potential cation channel subfamily M member 7	Q923J1
INKKFPNI	8	170.3	Nck-associated protein 1-like	Q8K1X4
NTPKYAKL	8	171.4	Isoform 2 of Zinc finger Ran-binding domain-containing protein 2	Q9R020-2; Q9R020-1
KIFTASNV	8	171.6	Isoform 2 of Ubiquitin-associated protein 2-like	Q80X50-4; Q80X50-3; Q80X50-5; Q80X50-2; Q80X50
VVADFGLARL	10	171.6	LIM domain kinase 1	P53668
SVFQTMVQM	9	172.4	nuclear factor of activated T-cells 5	Q9WV30-4; Q9WV30-1; Q9WV30-2
KNVVYRDL	8	172.5	RAC-alpha serine/threonine-protein kinase	P31750
AVVEFLTSV	9	175.6	Ribonuclease 3	Q5HZJ0

AVLKYYKV	8	177	Ubiquitin-40S ribosomal protein S27a	P62983
SVFAFGENKM	13	178.9	Protein RCC2	Q8BK67
SQQYYHSL	8	180	Pseudopodium-enriched atypical kinase 1	Q69Z38
QQYRFSVIM	9	181.2	Inverted formin-2	Q0GNC1; Q0GNC1-3
IIGTFERM	8	184.8	Vacuolar protein sorting-associated protein 35	Q9EQH3
RNPRYEKL	8	186.5	Pre-mRNA-processing factor 6	Q91YR7
SVISVIHL	8	187.5	CDP-diacylglycerol--inositol 3-phosphatidyltransferase	Q8VDP6
VIQDFVKM	8	189.8	CGG triplet repeat-binding protein 1	Q8BHG9
VCVTYEHL	8	190.3	Bifunctional glutamate/proline--tRNA ligase	Q8CGC7
VAPRYNWM	8	191.7	ATP-binding cassette sub-family A member 3	Q8R420
QVYGFLEV	8	191.7	Endoplasmic reticulum-Golgi intermediate compartment protein 3	Q9CQE7-2; Q9CQE7
IAPSFVKGF	9	193.5	ATP-dependent zinc metalloprotease YME1L1	O88967
VFTEVANL	8	195.2	Isoform 1 of Paired amphipathic helix protein Sin3b	Q62141-1; Q62141-2; Q62141-3; Q62141
SLFLFDEM	8	195.4	Torsin-1A	Q9ER39; P0C7W3; Q8R1J9
SITKFLNRI	9	195.8	Protein strawberry notch homolog 2	Q7TNB8-1; Q7TNB8-2
SAPIYKRI	8	197.1	exosome complex exonuclease RRP44	Q9CSH3
ITPPGYSHV	9	197.3	Lethal(3)malignant brain tumor-like protein 3	Q8BLB7-2; Q8BLB7-1
RTPDFFGSAL	10	197.3	Integrin alpha-5	P11688
AINIFQKL	8	199.3	Interferon-inducible protein AIM2	Q91VJ1
AVIDFSEAHL	10	199.7	Nucleic acid dioxygenase ALKBH1	P0CB42
VGLYYINKI	9	200.2	Keratinocyte-associated protein 2	Q5RL79
SLYSLPKL	8	202.4	BTB/POZ domain-containing protein 9	Q8C726
KIQSFINRM	9	202.8	vacuolar fusion protein CCZ1 homolog	Q8C1Y8
LTQQYHQL	8	206.2	MLV-related proviral Env polyprotein	P10404; P11370
IAPELYHKM	9	209.3	Lysine--tRNA ligase	Q99MN1
VWYRVLQI	8	209.6	AP-2 complex subunit alpha-2	P17426-2; P17427; P17426
RIYRFDTVI	9	210	Isoform 2 of Myomegalin	Q80YT7-2
KVLEFERV	8	211.9	Peptidylprolyl isomerase domain and WD repeat-containing protein 1	Q8CEC6
TNYRFKNLFI	10	212.2	G1/S-specific cyclin-E2	Q9Z238
IGPEYKSM	8	212.5	Pyridoxal-dependent decarboxylase domain-containing protein 1	Q99K01-4; Q99K01-3; Q99K01; Q99K01-2
TCFPFTSRF	9	212.6	Inactive rhomboid protein 2	Q80WQ6
VRYTGAGL	8	213.5	Cleavage stimulation factor subunit 1	Q99LC2
ANYQRDGPM	9	214.7	catalase	P24270
RDYQFKRL	8	215.7	ESF1 homolog	Q3V1V3
AVVRFINRF	9	216.3	Myotubularin-related protein 5	Q6ZPE2-2; Q6ZPE2
ACLMFKHL	8	218.9	Ribonucleoside-diphosphate reductase subunit M2 OS=Mus musculus OX=10090	P11157
VWITRAPGM	9	219.3	CCR4-NOT transcription complex subunit 2	Q8C5L3-2; Q8C5L3-1; Q8C5L3-3
RSPDWYNKV	9	220.5	StAR-related lipid transfer protein 8	Q8K031
SGVEFREM	8	224	transcriptional regulator ATRX	Q61687
VSVEYTEKM	9	225.2	Ribosomal RNA small subunit methyltransferase Nep1	O35130
ARYIHRRL	8	225.4	Sn1-specific diacylglycerol lipase beta	Q91WC9
SCQEFVTNL	9	225.6	PH and SEC7 domain-containing protein 4	Q8BLR5
HFLEKFGPL	9	227.3	Vacuolar protein sorting-associated protein 18 homolog	Q8R307
ANPRLWLRL	9	228.4	CCR4-NOT transcription complex subunit 10	Q8BH15-4; Q8BH15-3; Q8BH15-2; Q8BH15
IGVDHVARM	9	229.5	COP9 signalosome complex subunit 6	O88545
ATFIREVLM	9	230.8	Prostaglandin G/H synthase 1	P22437
SCYTFPLRI	9	231.2	Ubiquitin carboxyl-terminal hydrolase 40	Q8BWR4; Q8BWR4-3; Q8BWR4-4; Q8BWR4-2
RIYEYTSC	8	231.3	F-box only protein 3	Q9DC63
SAYQRGESL	9	233.2	Exocyst complex component 4	O35382
KNHEFIATF	9	233.3	Isoform 2 of Protein kinase C delta type	P28867-2; P28867-1
SQQTYYRV	8	234.3	ATP-binding cassette sub-family D member 1	P48410
KQWTFNYV	8	235.7	Rab-like protein 6	Q5U3K5
AVIQFLERI	9	236.9	Nucleolar pre-ribosomal-associated protein 1	Q571H0
VNIEHRDL	8	237.6	MORC family CW-type zinc finger protein 2A	Q69ZX6

SAVRPASL	8	238.4	Dedicator of cytokinesis protein 7	Q8R1A4-2; Q8R1A4
VNVERVLNV	9	239.5	Probable helicase with zinc finger domain	Q6DFV5-2; Q6DFV5-1; Q6DFV5-3
TIAKFHFL	8	239.5	ATP-binding cassette sub-family A member 5	Q8K448
QCFHFPTL	8	241.5	Integral membrane protein DGCR2/IDD	P98154
VAPHHLFL	8	242.8	CAD protein	B2RQC6-2; B2RQC6
RTWEFLTV	8	245.4	Isoform 3 of Protein-glucosylgalactosylhydroxylysine glucosidase	Q8BP56-3; Q8BP56-1; Q8BP56-2
RFFKMPYL	8	245.8	Lysosomal-associated transmembrane protein 5	Q61168
RVIDFTVL	8	246.4	Lethal(2) giant larvae protein homolog 2	Q3TJ91
ESLRYKLL	8	246.7	40S ribosomal protein S3	P62908
KNFAFLEF	8	247.3	Splicing factor U2AF 65 kDa subunit	P26369
TQQLYPSL	8	248.3	Ran-binding protein 9	P69566-2; P69566
SWLLARGPL	9	248.4	Tubulin epsilon and delta complex protein 1	Q3UK37
SLYDAFPKV	9	249.3	Dystonin	Q91ZU6-8; Q91ZU6-3; Q91ZU6-4; Q91ZU6-2; Q91ZU6-6; Q91ZU6
RSPKYDNV	8	249.4	Enkurin domain-containing protein 1	Q7TSV9
VNVAKLRYM	9	256.1	Serine/threonine-protein kinase rio2	Q9CQS5
RVYEMFQVF	9	259.7	ATP-binding cassette sub-family D member 1	P48410
VKYSPHCKL	9	261.1	L-lactate dehydrogenase A chain	P06151
SNLLLPKL	8	261.3	Anaphase-promoting complex subunit 4	Q91W96
VAFSRSGRLLL	11	261.8	Guanine nucleotide-binding protein G(I)/G(S)/G(T) subunit beta-2	P62880
VHIKLPHL	8	262.7	Creatine kinase B-type	Q04447
TFFTPGNL	8	262.9	Probable ATP-dependent RNA helicase DDX17	Q501J6
VMVQPINL	8	263.1	Small nuclear ribonucleoprotein E	P62305
VILPLISKL	9	265.8	Sorting nexin-19	Q6P4T1
TMFEVGKL	8	266.9	Protein FAM91A1	Q3UVG3
ITHEFINTF	9	268	Ribonuclease P protein subunit p40	Q8R1F9
TTFEHAHNM	9	268.3	Elongation factor 2	P58252
HILDFTCRL	9	268.7	Nischarin	Q80TM9-1
EQYKFYSV	8	269.9	Cytochrome c oxidase subunit NDUFA4	Q62425
SYFKGASL	8	270.1	Leucyl-cystinyl aminopeptidase	Q8C129
SIVPMVHL	8	274.9	Lysine-specific demethylase 6A	O70546-1; O70546-2
INHNFSQQCL	10	277.8	Eukaryotic translation initiation factor 3 subunit D	O70194
NIWRFPYI	8	281	Sodium-dependent neutral amino acid transporter B(0)AT3	O88576-6; O88576-5; Q60857; O88576; O88576-2; Q8BJI1; O88576-3; O88576-4
SSLRFVFC	8	281.2	Nischarin	Q80TM9-3; Q80TM9-2; Q80TM9
RQYVHRDL	8	282.7	Tyrosine-protein kinase JAK1	P52332
SVVVFQSF	8	282.8	Interferon-induced transmembrane protein 2	Q99J93
AQYKYIVI	8	285.5	Transmembrane protein 11, mitochondrial	Q8BK08
SRVSFTSL	8	287.2	Glucocorticoid-induced transcript 1 protein	Q8K3I9-3; Q8K3I9-1; Q8K3I9-2
EVYLFERI	8	288.1	Vacuolar protein sorting-associated protein 45	P97390
AQQMYQSL	8	291.5	Hydroxyacylglutathione hydrolase-like protein	Q9DB32; Q9DB32-2
ATKYFTNRL	9	293.4	Equilibrative nucleoside transporter 1	Q9JIM1-1; Q9JIM1-2
VFYAVKVL	8	295.7	Serine/threonine-protein kinase Sgk1	Q9WVC6-3; Q9WVC6-2; Q9WVC6
KWFFQKLRF	9	298	60S ribosomal protein L27	P61358
RCIRFWNTL	9	298.9	Fizzy-related protein homolog	Q9R1K5
SVVALHNL	8	299	26S proteasome non-ATPase regulatory subunit 7	P26516
IMYLIVETV	9	299.5	Striatin-interacting protein 1	Q8C079-3; Q8C079-2; Q8C079-4; Q8C079
VFRLLPQL	8	299.9	Acidic leucine-rich nuclear phosphoprotein 32 family member B	Q9EST5-1; Q9EST5-2
NTYRFLTF	8	301.7	Small subunit processome component 20 homolog	Q5XG71
VVMHPMPRV	9	301.8	CAD protein	B2RQC6-2; B2RQC6
KNVTFEHV	8	302.9	ATP-dependent zinc metalloprotease YME1L1	O88967
CSVRFPDM	8	303	Coatomer subunit beta	Q9JIF7
RLPLYLRL	8	303.5	Short transient receptor potential channel 4-associated protein	Q9JLV2; Q9JLV2-2
VNNFFQLTV	9	306.6	Huntingtin-interacting protein 1	Q8VD75
VVADFGLSRL	10	307.6	LIM domain kinase 2	O54785-1; O54785-2; O54785-3

IYFKVTHV	8		308.1	Pre-mRNA-splicing factor 38B	Q80SY5
KQFEYIEV	8		309.5	huntingtin	P42859; P42859-2
RGVDYHAL	8		312.2	deoxyhypusine synthase	Q3TXU5
TIIKPFNL	8		316.3	Targeting protein for Xklp2	A2APB8
VNNIFQLTV	9		316.5	Huntingtin-interacting protein 1-related protein	Q9JKY5
WVVVFKSL	8		316.8	Phosphatidylinositol-binding clathrin assembly protein	Q7M6Y3; Q7M6Y3-5; Q7M6Y3-6; Q7M6Y3-4; Q7M6Y3-2; Q7M6Y3-3
TSVRVMEL	8		318	Rab proteins geranylgeranyltransferase component A 2	Q9QZD5
SVITVKNL	8		319.1	protein O-mannosyl-transferase 2	Q8BGQ4-3; Q8BGQ4-2; Q8BGQ4
TNMASFPKM	9		319.7	Cathepsin K	P55097
VMVTIRSHM	9		320.8	Calcium-binding mitochondrial carrier protein Aralar1	Q8BH59
SCVHFMTL	8		321.5	THO complex subunit 5 homolog	Q8BKT7
KAFTHGVAM	9		323.4	Gamma-adducin	Q9QYB5-2; Q9QYB5-1
SWIAVSAL	8		325.3	Lysine-specific demethylase 2B	Q6P1G2-2; Q6P1G2
TNMTYEKM	8	1xOxidation [M8]	326.7	Transcription factor ETV6	P97360
RVQDYARI	8		326.8	Multidrug resistance-associated protein 6	Q9R1S7
RNVESYTKL	9		327.9	Dolichyl-diphosphooligosaccharide--protein glycosyltransferase subunit 1	Q91YQ5
RCWQYRQL	8		328.5	60S ribosomal protein L15	Q9CZM2
KNFHKSTGM	9		330	Caspase-3	P70677
QVVNFLQTF	9		332.8	ZW10 interactor	Q9CQU5
RLLDWFRSL	9		333	ADP-ribosylation factor-like protein 8B	Q9CQW2
KALDYIHHM	9		336.5	STE20-related kinase adapter protein alpha	Q3UUJ4; Q3UUJ4-2; Q3UUJ4-3
AVVAFVMKM	9		339.2	h-2 class I histocompatibility antigen, K-D alpha chain	P01901; P04223; P01902; P04223-2
AIITGFRNV	9		341.5	Mitotic checkpoint serine/threonine-protein kinase BUB1 beta	Q9Z1S0
THFQPAQL	8		345.9	adenylosuccinate lyase	P54822
EIYEFHKM	8		349.3	Neutrophil cytosol factor 1	Q09014
KALQFLEQV	9		353	T-complex protein 1 subunit zeta	P80317
RLIFIIILAYSYL	13		353.5	Isoform 2 of Rieske domain-containing protein	Q8K2P6-2
EVFEFLQQV	9		354.6	Protein mono-ADP-ribosyltransferase PARP14	Q2EMV9
VGPEFKDKL	9		361	plectin	Q9QXS1-6; Q9QXS1-13; Q9QXS1-7; Q9QXS1-9; Q9QXS1-5; Q9QXS1-14; Q9QXS1-12; Q9QXS1-3; Q9QXS1-2; Q9QXS1-10; Q9QXS1-15; Q9QXS1-11; Q9QXS1-16; Q9QXS1-4; Q9QXS1-1; Q9QXS1-8
SILAMINNM	9		361.5	CCR4-NOT transcription complex subunit 8	Q9D8X5
RNLSFVARQM	10		363.9	Isoform E of Tuberin	Q61037-6; Q61037-7
KNLEVFMHV	9		366.1	Fatty acyl-CoA reductase 1	Q922J9-3; Q922J9-4; Q922J9-2; Q922J9-1
KIVQFIVTL	9		367.1	Heat shock factor protein 2	P38533-2; P38533
KTLILPRL	8		368.8	Transcription initiation factor TFIID subunit 6	Q62311
SSLHPMGGL	9		369	CUGBP Elav-like family member 1	P28659-2; P28659-1; P28659-3; P28659-4
SVLDLSYNRL	10		370.3	Monocyte differentiation antigen CD14	P10810
RVIDFFTV	8		373.1	Lethal(2) giant larvae protein homolog 1	Q80Y17
SFIERLVEM	9		373.3	Transformation/transcription domain-associated protein	Q80YV3
ACPEYSRL	8		374.3	TATA element modulatory factor	B9EKI3
KSLEFWQV	8		376.9	regulator of telomere elongation helicase 1	Q0VGM9-2; Q0VGM9; Q0VGM9-4; Q0VGM9-5; Q0VGM9-3
SVFAFGENKM	10		377.8	Protein RCC2	Q8BK67
KIVEFLQSF	9		379.4	Sarcoplasmic/endoplasmic reticulum calcium ATPase 2	O55143-2; O55143-1
QRVEFAAL	8		381	nuclear mitotic apparatus protein 1	E9Q7G0
SLYEHVERM	9		383.3	Pleckstrin homology domain-containing family M member 1	Q7TSI1
RNIHYNSV	8		386.2	OTU domain-containing protein 5	Q3U2S4-2; Q3U2S4-1
SRVLFNQL	8		388.8	Dedicator of cytokinesis protein 1	Q8BUR4-1; Q8BUR4-2
VNDLFSRKF	9		392.4	General transcription factor II-I	Q9ESZ8-5; Q99NI3; Q9ESZ8-4; Q9ESZ8-2; Q9ESZ8-1; Q9ESZ8-3; Q9ESZ8-6
TIVEFLHSF	9		395.2	Bromodomain adjacent to zinc finger domain protein 2A	Q91YE5-2; Q91YE5; Q91YE5-3
SQYIRNCGV	9		403	lysozyme c-2	P17897; P08905
SIFEHKIVF	9		409.8	Zinc transporter ZIP6	Q8C145

EIVSFQHL	8		411.6	volume-regulated anion channel subunit LRRC8C	Q8R502
IQQNYKKL	8		414.6	Thyroid transcription factor 1-associated protein 26	Q8R2N0; Q8R2N0-2
FNFEKAYFIL	10		416.7	AP-1 complex subunit sigma-3	Q7TN05; P61967; Q9DB50
KNPGYIKL	8		416.9	Prohibitin-2	O35129
QNYDVAQV	8		418	Protein ecdysoneless homolog	Q9CS74
INYTNEKL	8		429.6	Myosin-11	Q6URW6-2; O08638-2; O08638-1; Q8VDD5; Q6URW6-1; Q6URW6-3; Q61879
SGVDYRGV	8		431.9	U6 snRNA-associated Sm-like protein LSm6	P62313
SNVKHVINF	9		436.4	atp-dependent rna helicase ddx3y	Q62095; Q62167; P16381
IRYFPTQAL	9		437.1	ADP/ATP translocase 1	Q3V132; P48962; P51881
RIVVMNNL	8		440.8	Phosphatidylinositol 4-phosphate 5-kinase type-1 alpha	P70182-2; P70182-3; P70182
FSYAFPKS	8		448.6	Monocarboxylate transporter 1	P53986
QVVEFKKL	8		449.9	Intraflagellar transport protein 52 homolog	Q62559
KNIFYKAI	8		450.2	lanosterol 14-alpha demethylase	Q8K0C4
RQRENFPNL	9		451.1	FLYWCH-type zinc finger-containing protein 1	Q8CI03-3; Q8CI03-2; Q8CI03-1
VMYQVVVI	8		452	Sorting nexin-20	Q9D2Y5
NVYLFNLSI	9		452.3	Succinate receptor 1	Q99MT6
SQQLYRHI	8		459.7	protein EFR3 homolog B	Q8BM75; Q8BM75-2; Q6ZQ18-2; Q6ZQ18
IGPRYSSVF	9		460.2	HEAT repeat-containing protein 5A	Q5PRF0-1
KIFSFAHQTI	10		462.7	Vacuolar protein sorting-associated protein 35	Q9EQH3
IFRLLPTL	8		479.1	F-box only protein 34	Q80XI1; Q80XI1-2
ENYDRGYSNL	10		479.8	probable ATP-dependent RNA helicase DDX5	Q61656
IAPGYHSI	8		483.6	Bromodomain-containing protein 8	Q8R3B7-1; Q8R3B7-2
RTWEFLTVV	9		486.7	Protein-glucosylgalactosylhydroxylysine glucosidase	Q8BP56-3; Q8BP56; Q8BP56-2
AIYCPPKL	8		490.8	26S proteasome non-ATPase regulatory subunit 11	Q8BG32
ICIKFDPM	8	1xOxidation [M8]	491.7	THO complex subunit 3	Q8VE80
VAYVLVCMLG	10		497.6	Inositol 1,4,5-trisphosphate receptor type 2	Q9Z329; Q9Z329-3; Q9Z329-2
TCLRFLLL	8		499.8	AT-rich interactive domain-containing protein 2	E9Q7E2
VILKFDQNRV	10		501.4	Procollagen-lysine,2-oxoglutarate 5-dioxygenase 3	Q9R0E1
AVSTFVNRM	9		515.7	Hepatocyte growth factor-regulated tyrosine kinase substrate	Q99LI8
SSVDFSLSGRI	11		521.5	Guanine nucleotide-binding protein subunit beta-5	P62881; P62881-2
SVVDLTCRL	9		522.8	glyceraldehyde-3-phosphate dehydrogenase	Q64467; P16858
RGPEYLTQM	9		523.1	Importin-5	Q8BKC5-2; Q8BKC5-1
SVVVFRII	8		529.9	Tumor necrosis factor receptor superfamily member 22	Q9ER62
ENFTFQKV	8		530.1	Isoform 2 of Protein kinase C delta type	P28867-2; P28867-1
VINFDFPKL	9		530.2	Probable ATP-dependent RNA helicase DDX6	P54823
TIQEFLERI	9		530.8	SAM domain-containing protein SAMSN-1	P57725
VNFNHIHKRI	10		535.9	phosphatidylinositol 4-kinase alpha	E9Q3L2
KMVVLLNL	8		545.6	E3 ubiquitin-protein ligase RNF213	E9Q555
CVYVMPTV	8		548.6	Unconventional myosin-VIIa	P97479; P97479-2
KIWEWLVGV	9		556.6	Zinc finger ZZ-type and EF-hand domain-containing protein 1	Q5SSH7; Q5SSH7-2
KILDTFEKL	9		556.8	Integrin alpha-L	P24063
SCLEFSLRI	9		560.5	E3 ubiquitin-protein transferase MAEA	Q4VC33; Q4VC33-2
SLIALAKL	8		560.7	Manganese-transporting ATPase 13A1	Q9EPE9
VAPHLTKL	8		561.5	volume-regulated anion channel subunit LRRC8D	Q8BGR2
FQYEHILSF	9		563.7	Unconventional myosin-X	F8VQB6; F8VQB6-2
SILERLEQM	9		575.3	Calmodulin-binding transcription activator 1	A2A891; A2A891-2; A2A891-5; Q80Y50-5; A2A891-3; Q80Y50-2; A2A891-4; Q80Y50-4; Q80Y50-3; Q80Y50
SNVERITNV	9		581.5	Phospholipid-transporting ATPase IA	P70704-3; P70704-2; P70704-1
SSMEKPPSL	9		584.2	Sperm-associated antigen 7	Q7TNE3
VWIKPSGL	8		585.7	Integrator complex subunit 14	Q8R3P6-1
RAYEFAERC	9		587.5	Clathrin heavy chain 1	Q68FD5
EVFDFRGM	8		591.2	Nck-associated protein 1	P28660; P28660-2
IPPEYRKL	8		591.6	Calpastatin	P51125-7; P51125-6; P51125-1; P51125-3; P51125-2

SMIIIRTL	8	612.4	Transmembrane 9 superfamily member 4	Q8BH24
KIINFAPSLL	10	613.7	Importin-8	Q7TMY7-2; Q7TMY7
RIVAFGVGL	9	616	2-oxoglutarate and iron-dependent oxygenase domain-containing protein 3	Q9D136
AMIELVERL	9	617	Tripartite motif-containing protein 44	Q9QXA7
KAPEYLHRF	9	620.4	Ras GTPase-activating protein-binding protein 2	P97379-1; P97379-2
IIYPPPEV	8	622.2	splicing factor 3A subunit 1	Q8K4Z5
SMIRLSESM	9	627.5	DNA replication licensing factor MCM6	P97311
EGPYYTHL	8	629.3	Methylcytosine dioxygenase TET3	Q8BG87
ESPSYRTL	8	637.4	PHD finger protein 20	Q8BLG0-2; Q8BLG0
KHFIHSCL	8	640.1	Nucleolar protein 7	Q9D7Z3
VVYDLSIRGF	10	641.5	Translin	Q62348
VAVDFGNYHL	10	642.8	large neutral amino acids transporter small subunit 1	Q9Z127
VQFDYSQERV	10	642.9	Dual adapter for phosphotyrosine and 3-phosphotyrosine and 3-phosphoinositide	Q9QXT1
KAWDFIQTQL	10	643.3	Leukocyte surface antigen CD53	Q61451
TTLPHMLM	8	645.3	Transcriptional repressor p66-beta	Q8VHR5-2; Q8VHR5
VIDKKLRNL	9	658.1	Caprin-1	Q60865
SCYEHIQV	8	659.8	Myotubularin-related protein 4	Q91XS1
IMVQLLRTV	9	661.1	Dedicator of cytokinesis protein 2	Q8C3J5
FQQEFPSL	8	661.9	Protein Prrc2c	Q3TLH4; Q3TLH4-5
RGLRYIHSM	9	670.3	Wee1-like protein kinase	P47810
TITDFINI	8	672.4	5'-AMP-activated protein kinase subunit gamma-2	Q91WG5-2; Q91WG5; O54950
KDYVFKEL	8	684	RNA polymerase II elongation factor ELL2	Q3UKU1
QWFQPPNL	8	686.1	serine/threonine-protein kinase haspin	Q9Z0R0
RKYYLPHL	8	688	Ral GTPase-activating protein subunit alpha-2	A3KGS3-1; A3KGS3-2
SCPTFLRM	8	690.9	Ubiquitin-protein ligase E3A	O08759-2; O08759; O08759-3
KGLTYITI	8	694.3	Sodium/potassium/calcium exchanger 5	Q8C261
IWLAAVKL	8	696.4	Pre-mRNA-processing factor 6	Q91YR7
VAVPLIGKL	9	700.7	Derlin-1	Q99J56
LASRFLGAV	9	703.6	Esterase OVCA2	Q9D7E3
VFRELPSL	8	713.9	General transcription factor IIH subunit 4	O70422
VDYDFSYHQM	10	714.1	Mini-chromosome maintenance complex-binding protein	Q8R3C0
QRYLHENL	8	727.8	saccharopine dehydrogenase-like oxidoreductase	Q8R127
VYIEHRLM	8	730.4	DNA replication licensing factor MCM7	Q61881
VWINAHGL	8	731.9	Aldehyde dehydrogenase family 16 member A1	Q571I9
LRYDHQSRL	9	737.8	Casein kinase II subunit alpha	Q60737
SRYTGASL	8	738.9	Histone deacetylase 3	O88895-1
YCLKFTKL	8	748.1	STAGA complex 65 subunit gamma	Q9CZV5
SVVSFDKV	8	752.5	Scaffold attachment factor B1	D3YXK2
AVWNFGAVGI	10	754.1	presenilin-2	Q61144-1; Q61144-2
NLFVFKEL	8	758.6	Rap guanine nucleotide exchange factor 2	Q8CHG7
AIVQFTRTF	9	763.4	vacuolar fusion protein CCZ1 homolog	Q8C1Y8
SSVDLRSL	8	770.5	Exosome complex exonuclease RRP42	Q9D0M0
RIYSFGLGGNC	13	770.9	Probable E3 ubiquitin-protein ligase HERC4	Q6PAV2-2; Q6PAV2
VWREVTTQL	9	772.2	Protein tweety homolog 2	Q3TH73-1; Q3TH73-2
AQRKHFPSV	9	772.9	V-type proton ATPase catalytic subunit A	P50516-1; P50516-2
KRLNFHLYM	9	777	Bystin	O54825
AIYGFGDKGSL	11	779.3	saccharopine dehydrogenase-like oxidoreductase	Q8R127
MRYVASYLL	9	781.5	60S acidic ribosomal protein P2	P99027
RCFSFLSV	8	783.1	Sphingosine kinase 2	Q9JIA7
NIIHFESL	8	784.4	Serine/threonine-protein kinase SMG1	Q8BKX6
RVTSFRDL	8	798.3	NSFL1 cofactor p47	Q9CZ44-2; Q9CZ44-3; Q9CZ44-1
QTFPSLQL	8	802.3	Homeobox protein Hox-B2	P0C1T1
VWRNPLNL	8	802.9	Isoform 5 of Suppressor of tumorigenicity 7 protein	Q99M96-5; Q99M96-2; Q99M96-9; Q8K4P7-3; Q8K4P7-2; Q99M96-4; Q99M96-8; Q99M96-6; Q8K4P7; Q99M96-3; Q99M96-7; Q99M96-1

VCDVFQHL	8	804.4	Ras-like protein family member 11A OS=Mus musculus OX=10090	Q6IMB1
EVIDFSSL	8	809.6	Cysteine-rich protein 2-binding protein	Q8CID0
TCLDYSNM	8	810.9	NADH dehydrogenase [ubiquinone] 1 alpha subcomplex subunit 8	Q9DCJ5
VHYDRSGRSL	10	818.4	THO complex subunit 4	O08583-2; O08583-1
RSLAYIHSF	9	818.5	Glycogen synthase kinase-3 beta	Q9WV60
VGVKYVNKF	9	821.7	Solute carrier family 12 member 4	Q9JIS8; Q91V14-2; Q91V14
TLVHPFRAL	9	837.9	Nucleolar protein 14	Q8R3N1
LQQYYVKL	8	846.2	spliceosome RNA helicase DDX39B	Q9Z1N5; Q8VDW0-2; Q8VDW0-1
RCIEFTKL	8	864.9	WD repeat-containing protein 7	Q920I9
KAYKYIVTC	9	873.5	Dynein light chain Tctex-type 3 OS=Mus musculus OX=10090	P56387
SCIRLAEL	8	876.4	DNA (cytosine-5)-methyltransferase 1	P13864-2; P13864
RIIEFQARC	9	895.3	Unconventional myosin-VIIa	P97479-1
QNHVFPLF	8	899.8	Importin-7	Q9EPL8
CRYWHRSL	8	903.2	glycylpeptide N-tetradecanoyltransferase 1	O70311; O70310
EIITFTAM	8	907.5	E3 ubiquitin-protein ligase UBR4	A2AN08-3; A2AN08-4; A2AN08-5; A2AN08
SRYFHWKL	8	911.7	Golgi pH regulator	Q8BS95
ISDIHTKL	8	925.2	Serine/threonine-protein kinase TBK1	Q9WUN2
QNVEFLQVI	9	931.4	Outer dense fiber protein 2	A3KGV1; A3KGV1-4; A3KGV1-3; A3KGV1-2
KGLEYLHFM	9	938.4	serine/threonine-protein kinase 4	Q9JI11; Q9JI10-2; Q9JI10
AFVRLPSL	8	950.5	Signal peptide peptidase-like 3	Q9CUS9
VAPFLRQEF	9	955.8	Probable ATP-dependent RNA helicase DDX27	Q921N6-1
KMLSKLETV	9	956.4	BRCA1-associated RING domain protein 1	O70445
SRVKFTRV	8	957.1	N-alpha-acetyltransferase 35, NatC auxiliary subunit	Q6PHQ8
ARIFFTYM	8	971.7	Fatty acid desaturase 1	Q920L1
KTFSYAGFEM	10	976.8	T-complex protein 1 subunit eta	P80313
KGPRYWEL	8	992	Anaphase-promoting complex subunit 1	P53995
VGPPHFQV	8	995.2	cytoplasmic FMR1-interacting protein 1	Q7TMB8-2; Q7TMB8-1
IILEFKGRKI	10	1008.3	Cleavage and polyadenylation specificity factor subunit 3	Q9QXK7
SCVDFRNM	8	1010.2	Negative regulator of reactive oxygen species	Q8BMT4
IRTGFINL	8	1010.4	H/ACA ribonucleoprotein complex subunit DKC1	Q9ESX5
AMAPRTLLL	9	1015.9	H-2 class I histocompatibility antigen, D-D alpha chain	P01898; P01900; P01897; P01899
RICTFEGKL	9	1022.8	EPM2A-interacting protein 1	Q8VEH5
IWLKVNRV	8	1032.7	Macrophage colony-stimulating factor 1 receptor	P09581
EGYKFCKI	8	1038.6	DNA-directed RNA polymerase II subunit RPB2	Q8CFI7
NIFQKLNYM	9	1053	Interferon-inducible protein AIM2	Q91VJ1
IGIENIHYL	9	1055.8	Isoform 2 of Malignant T-cell-amplified sequence 1	Q9DB27-2; Q9CQ21; Q9DB27
VIAELVNV	8	1075.9	General transcription factor IIH subunit 5	Q8K2X8
INAARGLGL	9	1077.3	DNA mismatch repair protein MSH2	P43247
IGLDYSSLYM	10	1085.4	Class E basic helix-loop-helix protein 41	Q99PV5
EVFDFRGMRL	10	1108.8	Nck-associated protein 1	P28660; P28660-2
VNYDLALKYF	10	1111.3	Protein sel-1 homolog 1	Q9Z2G6-1; Q9Z2G6-2
SRLDKFKQL	9	1125.1	TBC1 domain family member 22A	Q8R5A6-2; Q8R5A6
EIVTFERL	8	1126.5	Fragile X mental retardation syndrome-related protein 1	Q61584; Q61584-5; Q61584-3; Q61584-6; Q61584-4; Q61584-2; Q61584-7
INIAHRDV	8	1145.7	MAP kinase-activated protein kinase 2	P49138
AMGVNLTSM	9	1147.9	proliferating cell nuclear antigen	P17918
EGYKYERI	8	1170.8	Chromodomain-helicase-DNA-binding protein 4	Q6PDQ2; A2A8L1
VNDIFERI	8	1171.7	Histone H2B type 2-B	Q64475; Q8CGP2; Q64525; Q64524; Q8CGP1; P10854; Q64478; Q9D2U9; Q8CGP2-2; Q6ZWY9; Q8CGP0; P10853
QILSDFPKL	9	1193.6	Nucleolar GTP-binding protein 1	Q99ME9
RNTPFMGI	8	1194.9	Protein TANC1	Q0VGY8
RSLSYSEILRL	11	1228	Engulfment and cell motility protein 2	Q8BHL5; Q8BHL5-2; Q8BHL5-3
RVVVFVDM	8	1248.8	Heat shock protein 105 kDa	Q61699; Q61699-2
AQQSYKSL	8	1252.4	Ubiquitin-conjugating enzyme E2 Q1	Q7TSS2-2; Q7TSS2

SWINGIRGL	9		1253.1	Spectrin alpha chain, non-erythrocytic 1	P16546; P16546-2
IRPEHVLRL	9		1273	Protein unc-119 homolog B	Q8C4B4; Q8C4B4-2
RAPSYIEI	8		1287.5	E3 ubiquitin-protein ligase UBR4	A2AN08-3; A2AN08-5; A2AN08
KGPSFDVQV	9		1291.9	dipeptidyl peptidase 3	Q99KK7
VADYMRYLM	9	1xOxidation [M9]	1293.8	60S ribosomal protein L5	P47962
SAAENMIKL	9		1294.1	Succinate--CoA ligase [ADP-forming] subunit beta, mitochondrial	Q9Z2I9
LPGRFSHL	8		1301	Tumor necrosis factor-inducible gene 6 protein	O08859
VTDLRVTGM	9		1311	N-alpha-acetyltransferase 35, NatC auxiliary subunit	Q6PHQ8
SMLYPLSHGF	10		1355.1	DP-N-acetylglucosamine--peptide N-acetylglucosaminyltransferase 110 kDa subu	Q8CGY8-2; Q8CGY8
KICKFTEV	8		1365.2	60S ribosomal protein L13a OS=Mus musculus OX=10090	P19253
SGFQAKTQM	9		1372.1	Nucleolus and neural progenitor protein	Q8R2U2
SGPQLNAQL	9		1384.5	Protein MEMO1	Q91VH6
SMVDVVML	8	1xOxidation [M7]	1390.9	Large proline-rich protein BAG6	Q9Z1R2
AYQHLFYL	8		1408.8	Ras GTPase-activating-like protein IQGAP1	Q9JKF1
KVFDFAGEEVI	12		1408.9	Craniofacial development protein 1	O88271
VFIELNHI	8		1422.9	Isoform 14 of MAP kinase-activating death domain protein	Q80U28-14
NCLLFIQRL	9		1430.7	centromere protein W	Q3URR0
SCIVLSAL	8		1473.3	Mast cell-expressed membrane protein 1	Q9D8U6
SCVNFKEMM	9		1476.6	Interleukin-18	P70380
SCNIFRTL	8		1489	erine/threonine-protein phosphatase 2A 56 kDa regulatory subunit epsilon isoforn	Q61151
VMVEMERV	8		1502.7	Integrin alpha-4	Q00651
SAPENAVRM	9		1503.2	Protein C10	O35127
VAPPYPHFF	9		1513.5	Protein misato homolog 1	Q2YDW2-1
IIQSIRMGM	9		1523.2	Stress-associated endoplasmic reticulum protein 1	Q6TAW2-1; Q9Z1W5
SCPVFTSI	8		1525	Solute carrier family 35 member G1	Q8BY79
KTFEVNVL	8		1526.5	Estradiol 17-beta-dehydrogenase 11	Q9EQ06-2; Q9EQ06
RRLPIFSRL	9		1552.7	mRNA decay activator protein ZFP36L2	P23949; P23950
VQVASFAM	8		1576.6	Cholesterol side-chain cleavage enzyme, mitochondrial	P9QZ82
KIFKKEKEM	9		1582	proliferation-associated protein 2G4	P50580; P50580-2
ANPTFPNFF	9		1599.1	Glutaminyl-peptide cyclotransferase	Q9CYK2-2; Q9CYK2
SWLHTSEL	8		1600.3	Large subunit GTPase 1 homolog	Q3UM18; Q3UM18-2
VFEIKLQSF	9		1623.6	Exocyst complex component 5	Q3TPX4
LRAYFQGL	8		1631	Protein patched homolog 2	O35595
SCFIFLVGT	9		1631.1	Ras-related protein Rab-36	Q8CAM5
SRVTFVNF	8		1632.4	Cytoplasmic dynein 1 heavy chain 1	Q9JHU4
SWIEVQFL	8		1634	E3 ubiquitin-protein ligase ARIH1	Q9Z1K5
IRTLYLTL	8		1640.2	Transformation/transcription domain-associated protein	Q80YV3
INFDFNTIRC	10		1650.6	BTB/POZ domain-containing protein KCTD20	Q8CDD8-2; Q8CDD8
KNVPRSLGM	9		1654.9	Isoform 2 of Mediator of RNA polymerase II transcription subunit 1	Q925J9-4; Q925J9-2; Q925J9-3; Q925J9-1
SRFQPLNL	8		1667.6	Genetic suppressor element 1	Q3U3C9-4; Q3U3C9-3; Q3U3C9-2; Q3U3C9
QCVKFLVTL	9		1667.7	Ubiquitin carboxyl-terminal hydrolase 24	B1AY13
VLAVLPRL	8		1686.3	Probable rRNA-processing protein EBP2	Q9D903
LQSQYRSL	8		1689.5	Rho guanine nucleotide exchange factor 7	Q9ES28-1; Q9ES28-7; Q9ES28-5; Q9ES28-3; Q9ES28-4; Q9ES28-6; Q9ES28-2
RNYQRKNDM	9		1694.1	Chromodomain-helicase-DNA-binding protein 4	Q6PDQ2; A2A8L1
KPVQRFPQFIL	12		1705	Rho guanine nucleotide exchange factor 10-like protein	A2AWP8; A2AWP8-4; A2AWP8-2; A2AWP8-3
VSLLDIDHL	9		1708.2	HEAT repeat-containing protein 3	Q8BQM4
RIADFGAAARL	11		1710.3	Mitogen-activated protein kinase kinase kinase 1	P53349
SWIAVQEL	8		1736.3	Pyroglutamyl-peptidase 1	Q9ESW8
AWIHAAHV	8		1760.9	Retrovirus-related Pol polyprotein	P10400
VRYINENL	8		1786.4	T-complex protein 1 subunit alpha	P11983-2; P11983
SLYDYQSLS	9		1793	Dedicator of cytokinesis protein 3	Q8CIQ7
FKFSLPQL	8		1803.9	Bardet-Biedl syndrome 1 protein homolog	Q3V3N7
VGGYFLAGRS	11	1xOxidation [M11]	1833.2	Sodium/glucose cotransporter 2	Q923I7; Q8VDT1

KVFDFAGEEV	10		1841.9	Craniofacial development protein 1	O88271
RWLRAVLL	8		1871.1	LRP chaperone MESD	Q9ERE7
TVGYSLSLI	9		1884.7	glucagon-like peptide 2 receptor	Q5IXF8
VCFESKENL	9		1892	Alpha-catulin	O88327
AFDFYHKASRL	11		1901	E3 ubiquitin-protein ligase UBR1	O70481
ILIDFTKRGL	10		1921.8	Hexokinase-2	O08528
AWIPVRML	8		1932	Zinc finger CW-type PWWP domain protein 1	Q6IR42
VNYDVRVF	8		1933.5	Nuclear pore complex protein Nup133	Q8R0G9
VNMDMSLHSM	10		1939.2	CCR4-NOT transcription complex subunit 11	Q9CWN7
IRIVLVGL	8		1949.1	Disintegrin and metalloproteinase domain-containing protein 9	Q61072
VYIYKEHF	8		2053.4	Small subunit processome component 20 homolog	Q5XG71
TRYQGVNL	8		2061.8	Polyadenylate-binding protein 1	P29341
SLLSFEKI	8		2108.4	Cysteine and histidine-rich protein 1	Q9QXA1-2; Q9QXA1-3; Q9QXA1
SWLHPPPVL	9		2181.6	Phosphatidylinositol N-acetylglucosaminyltransferase subunit Q	Q9QYT7
IGHRYIEVF	9		2188.9	Heterogeneous nuclear ribonucleoprotein F	Q9Z2X1-2; Q9Z2X1-1
IIDEKKYYL	9		2212.5	Nuclear inhibitor of protein phosphatase 1	Q8R3G1
NGYDYGQCRL	10		2217.5	Serine/arginine-rich splicing factor 9 OS=Mus musculus OX=10090	Q9D0B0
MCPEEYPHL	9		2223.3	Nck-associated protein 1-like	Q8K1X4
SMVKLIGI	8		2262.3	Inactive phospholipid phosphatase 7	Q91WB2
SFDFHGRRM	9	1xOxidation [M9]	2271.3	nucleoporin Seh1	Q8R2U0-1
VNFEFPEFQ	9		2280.6	40S ribosomal protein S7	P62082
SLIILHQL	8		2299.2	Nuclear pore complex protein Nup133	Q8R0G9
QWIVVRTL	8		2317	solute carrier organic anion transporter family member 4A1	Q8K078-2; Q8K078
KHLEKILNV	9		2336.7	Integrator complex subunit 7	Q7TQK1-1; Q7TQK1-2
SHITFLTIKGAG	14		2362.3	Lysosomal protective protein	P16675
QGPDYVLV	8		2417.5	proteasome subunit beta type-2	Q9R1P3
EIVDFLTGV	9		2432.7	KAT8 regulatory NSL complex subunit 3	A2RSY1-2; A2RSY1; A2RSY1-3
LQQQYNRV	8		2444.8	Talin-1	P26039
IAGFQAFEV	9		2503.9	Poly(A)-specific ribonuclease PNLDC1	B2RXZ1
KCYIVATV	8		2507.9	rRNA-processing protein FCF1 homolog	Q9CTH6
SRVTYKNV	8		2512.4	GTP-binding nuclear protein RAN	Q61820; P62827
VGPEFKDKLL	10		2521.6	plectin	Q9QXS1-6; Q9QXS1-13; Q9QXS1-7; Q9QXS1-9; Q9QXS1-5; Q9QXS1-14; Q9QXS1-12; Q9QXS1-3; Q9QXS1-2; Q9QXS1-10; Q9QXS1-15; Q9QXS1-11; Q9QXS1-16; Q9QXS1-4; Q9QXS1-1; Q9QXS1-8
CIVDFQKRQGL	11		2545.9	Hexokinase-3	Q3TRM8
RIVELISRV	9		2553.6	TOM1-like protein 2	Q5SRX1-3; Q5SRX1-4; Q5SRX1; Q5SRX1-2
SADKFVKI	8		2570.5	Periodic tryptophan protein 1 homolog	Q99LL5
THVRLPLL	8		2582	Kelch-like protein 24	Q8BRG6
TIFIRGGNKM	10		2628.3	T-complex protein 1 subunit epsilon	P80316
TIHGLIYNAL	10		2681.4	erine/threonine-protein phosphatase 2A 56 kDa regulatory subunit gamma isoforr	Q60996-2; Q60996-4; Q60996-1; Q60996-3
ASVLNVNHI	9		2684.9	Ankyrin repeat domain-containing protein 17	Q99NH0-1
KFLLLPMV	8		2716.1	Cathepsin K	P55097
VRILLTNI	8		2729.5	Bifunctional coenzyme A synthase	Q9DBL7
YCLYWSTL	8		2792.3	Cyclic nucleotide-gated olfactory channel	Q62398
SSVKKIEKM	9		2816.4	SLIT-ROBO Rho GTPase-activating protein 2	Q91Z67; Q812A2; Q91Z69
NYRFKNLF	8		2839.8	G1/S-specific cyclin-E2	Q9Z238
KRYIHRDL	8		2875.4	Tyrosine-protein kinase JAK2	Q62120
VNCEHIKSF	9		2875.4	DNA replication licensing factor MCM4	P49717
TKHFPQFSQI	10		3017.2	ATP-dependent RNA helicase DDX55	Q6ZPL9
KICDFGLARV	10		3025	mitogen-activated protein kinase 1	P63085; O54949
SLIRQLSL	8		3071.6	Protein moonraker	Q6A000
ELFPVFTQL	9		3071.7	Bombesin receptor-activated protein C6orf89 homolog	Q99KU6
EICRFIQQL	9		3077.5	snRNA-activating protein complex subunit 2	Q91XA5

VFLERGEVM	9		3131.6	DNA-directed RNA polymerase II subunit RPB1	P08775
KWTSVIRL	8		3143.6	Platelet-activating factor acetylhydrolase IB subunit alpha	P63005-1; P63005-2
KNYEQPTI	8		3148.2	Spermatogenesis-associated protein 6	Q3U6K5
SLVELTSL	8		3155.5	Adenine phosphoribosyltransferase	P08030
RSPDGYLQI	9		3186.1	Alpha-1,3-mannosyl-glycoprotein 4-beta-N-acetylglucosaminyltransferase B	Q812F8
KICDFGLARI	10		3223.3	mitogen-activated protein kinase 3	Q63844
YAPTIRAL	8		3257.5	Nephrin	Q9QZS7-1
KSFERKNKMLI	11		3264.8	Linker for activation of T-cells family member 2	Q9JHL0-1; Q9JHL0-2
SRYAHWVV	8		3332.8	transportin-1	Q8BFY9; Q8BFY9-2; Q99LG2
MLAVAYGHI	9	1xOxidation [M1]	3397.2	Serine/threonine-protein phosphatase 6 regulatory ankyrin repeat subunit B	B2RXR6
GYEFIHKL	8		3508.7	Tyrosine--tRNA ligase, mitochondrial	Q8BYL4
SSYDWSKGHE	11		3520.9	mRNA export factor	Q8C570
HGTFWKHWPS	11		3571.3	tRNA 2'-phosphotransferase 1	Q8K3A2
KCYEMTDL	8		3615.3	Serine/threonine-protein kinase PLK2	P53351
EESFPGFVNL	10		3644.2	Nesprin-1	Q6ZWR6-2; Q6ZWR6-3; Q6ZWR6-4; Q6ZWR6-1
AWIHIKAL	8		3666.8	V-type proton ATPase subunit C 2	Q99L60; Q99L60-3; Q9Z1G3; Q99L60-2
KRYVESLL	8		3716.4	E3 ubiquitin-protein ligase RNF213	E9Q555
LAVSGFHL	8		3837.4	Uncharacterized protein C14orf79 homolog	Q8BHN9
DVPAFRTLV	9		3881.7	Dual specificity testis-specific protein kinase 1	O70146
QWIQVRNM	8		3909.7	ATP synthase subunit gamma, mitochondrial	Q91VR2
VTHHYFLRWT	12		3927.4	C-type lectin domain family 4 member D	Q9Z2H6
AWIKVEQL	8		3940.3	Putative oxidoreductase GLYR1	Q922P9
SLVDLNTRL	9		3966.7	Mitochondrial amidoxime reducing component 2	Q922Q1
NAPEFVKV	8		3980.5	Protein FAM107B	Q3TGF2
IWLHVEGV	8		3988.3	Pyridoxal-dependent decarboxylase domain-containing protein 1	Q99K01-4; Q99K01-5; Q99K01-3; Q99K01; Q99K01-2
ITGYFPSMY	9		4045	Neutrophil cytosol factor 1	Q09014
LAPEFAKRNV	10		4124.3	Peroxiredoxin-6 OS=Mus musculus OX=10090	O08709
YSPPLNKL	8		4127	Cellular tumor antigen p53 OS=Mus musculus OX=10090	P02340; P02340
KCVDFQTL	8		4132.3	DNA replication licensing factor MCM5 OS=Mus musculus OX=10090	P49718
PAAMFRLL	8		4171.1	Amyloid-beta A4 precursor protein-binding family A member 2	P98084
CVDDFTARAL	10		4192.1	2-(3-amino-3-carboxypropyl)histidine synthase subunit 1	Q5NCQ5
MWIQVRTM	8		4210.2	E3 ubiquitin-protein ligase UHRF1	Q8VDF2-2; Q8VDF2
KIVELNTKL	9		4215.5	dystonin	Q91ZU6-3; Q91ZU6-4; Q91ZU6-2; Q91ZU6
IHISKKWGF	9		4252.3	60S ribosomal protein L10-like	P86048; Q6ZWV3
VGPDFGTKKL	10		4340	DNA-dependent protein kinase catalytic subunit	P97313-1
VFINVEKV	8		4495.4	exocyst complex component 1	Q8R3S6
RLYLNGNQI	9		4583.8	SLIT and NTRK-like protein 4	Q810B8
ICPKYSNC	8		4614.8	Adhesion G protein-coupled receptor E1	Q61549
TRLYPEPSL	9		4723.6	NF-kappa-B inhibitor delta	Q2TB02
RVFEEKKYM	9		4744	RasGAP-activating-like protein 1	Q9Z268
MRILHVNGF	9		4768.1	Guanine nucleotide-binding protein G(S) subunit alpha isoforms XLas	P63094; P63094-2; P63094-3; Q8CGK7; Q6R0H7; Q6R0H7-4
SFLDVRNI	8		4873	26S proteasome non-ATPase regulatory subunit 2	Q8VDM4
SWLDVRHI	8		4877.9	Interleukin-1 receptor-associated kinase 3	Q8K4B2
NSYRYSVP	8		4893.4	Claudin-8	Q9Z260
ERFPNFRNV	9		4931.9	Hypoxia-inducible factor 1-alpha inhibitor	Q8BLR9-1
