# Supplementary material for: Palladium-Induced Temporal Internalization of MHC Class I Contributes to T Cell-Mediated Antigenicity
Source: Front Immunol. 2021 Dec 23;12:736936. doi: 10.3389/fimmu.2021.736936 (PMC8732370; doi:10.3389/fimmu.2021.736936)
Supplement: Supplementary file 4 [file Table_3.pdf]

Supplementary Table 3 Peptide list on H-2D^b in the absence of $PdCl_2$

Sequence	Sequence Length	Modification	Affinity (nM)	Protein	Protein Accession
SAISNLDYI	9		1.9	Guanine nucleotide exchange factor VAV3	Q9R0C8-4; Q9R0C8-1
TAVVNVTYM	9	1xOxidation [M9]	2.8	Insulin-like growth factor 2 mRNA-binding protein 2	Q5SF07-2; Q5SF07
YQVVNSYPL	9		2.8	Probable ATP-dependent RNA helicase DDX20	Q9JJY4
YAVGNHDFI	9		3.2	PCI domain-containing protein 2	Q8BFV2
FAIQNPTLI	9		3.2	Transcription factor Sp2	Q9D2H6-1; Q9D2H6-2
YSITNTEEL	9		3.2	Leucine-rich repeats and immunoglobulin-like domains protein 2	Q52KR2
RAIENIDTL	9		3.4	Protein phosphatase 1 regulatory subunit 7	Q3UM45
YAVNNQFTM	9		3.4	Histone-arginine methyltransferase CARM1	Q9WVG6; Q9WVG6-2
NSIRNLDTI	9		3.6	Ataxin-10	P28658
RAIRNMNTL	9		3.6	E3 ubiquitin-protein ligase synoviolin	Q9DBY1-2; Q9DBY1
ESAISNLDYI	10		3.9	Guanine nucleotide exchange factor VAV3	Q9R0C8-4; Q9R0C8-1
AAMLNYTHI	9		3.9	Dol-P-Man:Man(7)GlcNAc(2)-PP-Dol alpha-1,6-mannosyltransferase	Q8VDB2
FALANEHYL	9		4	N-acyl-phosphatidylethanolamine-hydrolyzing phospholipase D	Q8BH82
FSVTNPHTM	9		4	Ligand-dependent nuclear receptor-interacting factor 1	Q8CDD9-2; Q8CDD9
VALINKDFL	9		4	Transmembrane protein 82	Q8R115
YQLSNLYAI	9		4	Multivesicular body subunit 12B	Q6KAU4
SQISNTEFL	9		4.1	BRISC complex subunit Abraxas 2	Q3TCJ1
YMLANLTHL	9		4.2	kelch-like protein 28	Q9CR40-1; Q9CR40-2
ASLSNRLYI	9		4.2	phosphatidylinositol 4-kinase alpha	E9Q3L2
FALVNGNNI	9		4.2	AP-1 complex subunit gamma-1	P22892
KSIVNQVFL	9		4.4	RING finger and transmembrane domain-containing protein 1	Q9DCN7
SGIRNISFM	9		4.4	Disintegrin and metalloproteinase domain-containing protein 10	O35598
SAMENLNEM	9	1xOxidation [M9]	4.4	N-alpha-acetyltransferase 16, NatA auxiliary subunit	Q9DBB4
SAPNNITVL	9		4.5	Beta-galactosidase	P23780
STIRNADVI	9		4.6	Phosphatidylcholine translocator ABCB4	P21440
AAISNYDRPL	10		4.6	Low-density lipoprotein receptor-related protein 8	Q924X6; Q924X6-2
YAPINANAI	9		4.6	Metastasis-associated protein MTA2	Q9R190
TAPVNIAVI	9		4.6	Diphosphomevalonate decarboxylase	Q99JF5
SAVVNKVPL	9		4.6	Transmembrane protein 263	Q9DAM7
KAPTNVTCI	9		4.6	von Willebrand factor A domain-containing protein 8	Q8CC88-2; Q8CC88
RALSNLESI	9		4.7	Ubiquilin-1	Q8R317-2; Q8R317; Q8C5U9
SSLINGSFL	9		4.8	Tyrosine-protein kinase ABL2	Q4JIM5
FSVENFDAL	9		4.9	Integrin alpha-X	Q9QXH4
AAMKNVTEL	9		5	14-3-3 protein gamma	P61982
YGLKNLTAL	9		5	Phosphoinositide 3-kinase adapter protein 1	Q9EQ32-3; Q9EQ32-2; Q9EQ32-1
SAVENLNEM	9	1xOxidation [M9]	5.1	N-alpha-acetyltransferase 15, NatA auxiliary subunit	Q80UM3
YSVNNVSVI	9		5.1	Ankyrin repeat domain-containing protein 13A	Q80UP5
SAIRNGTDM	9		5.1	Coiled-coil domain-containing protein 189	Q6NZQ0
SMLSNPDLM	9		5.1	Isoform 2 of Ubiquilin-1	Q8R317-2; Q8R317; Q9QZM0
AQLANTTAI	9		5.2	Pre-mRNA-processing factor 40 homolog A	Q9R1C7-2; Q9R1C7
YSVENGDCL	9		5.2	Tubulin-specific chaperone E	Q8CIV8
SQIVNPESI	9		5.3	Pentatricopeptide repeat-containing protein 2, mitochondrial	Q8R3K3
SSLENATSL	9		5.4	Signal peptide peptidase-like 2A	Q9JJF9
AALLNQQFL	9		5.4	Serine/threonine-protein phosphatase 2B catalytic subunit beta isoform	P48455; P48453-2; P48453
SAILNEWTL	9		5.5	Pleckstrin homology domain-containing family M member 1	Q7TSI1
KALINADEL	9		5.5	Spectrin alpha chain, non-erythrocytic 1	P16546; P16546-2
SSLVNSQTL	9		5.5	TGF-beta-activated kinase 1 and MAP3K7-binding protein 2	Q99K90
YAIENVETNL	10		5.5	transportin-3	Q6P2B1-1; Q6P2B1-2
SMLINGYAL	9		5.5	T-complex protein 1 subunit alpha	P11983-2; P11983

VQIANPAFI	9		5.6	Protein mono-ADP-ribosyltransferase PARP4 OS=Mus musculus OX=10090	E9PYK3
RQILNADAM	9		5.6	protein virilizer homolog	A2AIV2-1
SAVKNDYEM	9		5.6	Replication protein A 70 kDa DNA-binding subunit	Q8VEE4
SSLQNLTSL	9		5.6	Toll-like receptor 13	Q6R5N8
STIQNADLI	9		5.7	Phosphatidylcholine translocator ABCB4	P21440; P06795; P21447
YGLLNVTKI	9		5.8	Rho GTPase-activating protein 12	Q8C0D4
RSLENYHFV	9		5.8	Clathrin interactor 1	Q99KN9-1; Q99KN9-2
FAHTNIESL	9		5.9	Protein disulfide-isomerase A3	P27773
AALLNTDLV	9		5.9	U3 small nucleolar RNA-interacting protein 2	Q91WM3
YQYSNTVYL	9		6	Aminopeptidase N	P97449
VALRNLEQI	9		6.1	SH3 domain-binding protein 5-like	Q99LH9
KSLKNYITI	9		6.1	Probable cysteine--tRNA ligase, mitochondrial	Q8BYM8
AAPSNLPYL	9		6.1	Dual specificity protein phosphatase 4	Q8BFV3
FSVLNWDQV	9		6.2	Protein FAM46C	Q5SSF7
ASMTNRELM	9		6.2	ADP-dependent glucokinase	Q8VDL4; Q8VDL4-3
VALRNINLI	9		6.2	AP-1 complex subunit beta-1	Q9DBG3-2; O35643; Q9DBG3
KSVANLEYL	9		6.2	GRIP and coiled-coil domain-containing protein 2	Q8CHG3
SALANYIHL	9		6.3	Integral membrane protein GPR180	Q8BPS4
SSLQNYAKI	9		6.3	natural resistance-associated macrophage protein 1	P41251
RSLSNATII	9		6.3	signal recognition particle 54 kDa protein	P14576-2; P14576-1
ASPTNPTAI	9		6.3	Ankyrin repeat domain-containing protein 13D	Q6PD24
AALQNLVKI	9		6.4	Importin subunit beta-1	P70168
RMLENYEEI	9		6.4	Cullin-7	Q8VE73; Q8VE73-3
FAPVNVTTEV	10		6.4	Elongation factor 1-alpha 1	P10126
KSIKNANTI	9		6.4	Plexin-C1	Q9QZC2
SSLENAFQI	9		6.5	DDB1- and CUL4-associated factor 17	Q3TUL7-2; Q3TUL7
KALANVATV	9		6.6	Nicastrin	P57716
SGLANGIFM	9		6.6	Mucosa-associated lymphoid tissue lymphoma translocation protein 1 homolog	Q2TBA3-2; Q2TBA3-1
TALENLSTL	9		6.6	Fatty acid synthase	P19096
FGIKNFSAI	9		6.6	poyllysine-residue acetyltransferase component of pyruvate dehydrogenase complex, mito	Q8BMF4
SSVLNLTEL	9		6.6	Glycosyltransferase-like domain-containing protein 1	Q8BW56; Q8BW56-2
YAIVGLETI	9		6.6	Xaa-Pro aminopeptidase 1	Q6P1B1
YAMENTRQTI	10		6.7	Nuclear receptor corepressor 1	Q60974-1; Q60974-2
YAVSNHSGTT	11		6.7	Ubiquitin carboxyl-terminal hydrolase 2	O88623-2; O88623-1; O88623-4; O88623-3
SSPCNIEVV	9		6.7	DNA replication ATP-dependent helicase/nuclease DNA2	Q6ZQJ5; Q6ZQJ5-2
YMNVNNYWI	9	1xOxidation [M2]	6.7	GPI inositol-deacylase	Q3UUQ7
KANVNLAYL	9		6.7	RNA-binding protein 38	D3Z4I3; Q62176
RSIQNAQFL	9		6.8	Lysosomal acid phosphatase	P24638
AQMQNPDTL	9		6.8	Isoform 2 of Ubiquilin-1	Q8R317-2
SSLHNVYLI	9		6.8	Origin recognition complex subunit 2	Q60862
ASLVNADKL	9		6.9	Vacuolar protein sorting-associated protein 33B	P59016
FQFINPTTI	9		6.9	GATOR complex protein WDR59	Q8C0M0-1; Q8C0M0-2; Q8C0M0-3
SSVQNKEYL	9		6.9	zinc finger protein 518B	B2RRE4
AAIENIEHL	9		6.9	Leucine-rich PPR motif-containing protein, mitochondrial	Q6PB66
SSLKNGVVL	9		6.9	Rho guanine nucleotide exchange factor 6	Q8K4I3
FSPTNYHFL	9		7	Beta-mannosidase	Q8K2I4
STVRNADVI	9		7	Multidrug resistance protein 1B	P06795; P21447
SSPLNHIYL	9		7	Methionine aminopeptidase 1D, mitochondrial	Q9CPW9
YMHANGASM	9		7.1	cytochrome b	P00158
YQVLNEVVI	9		7.1	NAD kinase	P58058
AALENTHLL	9		7.2	Mitotic spindle-associated MMXD complex subunit MIP18	Q9D187
SSLSNEHVL	9		7.2	Serine/threonine-protein phosphatase 6 regulatory ankyrin repeat subunit C	Q8BTI7

AAITNKYQL	9		7.2	RNA polymerase I-specific transcription initiation factor RRN3	B2RS91
RALLNAESL	9		7.2	Exonuclease 3'-5' domain-containing protein 2	Q8VEG4-2; Q8VEG4
YALSDLDTL	9		7.2	THAP domain-containing protein 3	Q8BJ25
SSVSNKTTL	9		7.2	LisH domain and HEAT repeat-containing protein KIAA1468	Q148V7-3; Q148V7-1; Q148V7-2
SSHCNAEAM	9		7.2	Protein JBTS17	Q8CE72
ASFSNSTYL	9		7.3	Myotubularin-related protein 13	E9PXF8-2; E9PXF8
KAPVNTAEL	9		7.4	BRCA2 and CDKN1A-interacting protein	Q9CWI3
SMVQNRVFL	9		7.4	Dedicator of cytokinesis protein 2	Q8C3J5
MGILNTDTL	9		7.4	Rho GTPase-activating protein 24	Q8C4V1-3; Q8C4V1-1; Q8C4V1-2
AAPTNANSL	9		7.5	Isoform 3 of CCR4-NOT transcription complex subunit 4	Q8BT14; Q8BT14-2; Q8BT14-3
SSIENKQDWI	10		7.5	Triple functional domain protein	Q0KL02-4; Q0KL02; Q0KL02-3; Q0KL02-2
TTICNLYTM	9		7.5	Folliculin-interacting protein 1	Q68FD7
FQIVNPHLL	9		7.6	Ribonucleoside-diphosphate reductase large subunit	P07742
SSPANLNAM	9		7.6	zinc finger protein 382	B2RXC5
YSLPNAPTL	9		7.6	Tubulin polyglutamylase complex subunit 2	Q66JT5
GSLTNLHTL	9		7.7	zinc finger protein 40	Q03172
AALKNAFSL	9		7.7	DmX-like protein 1	Q8BPN8; Q8BPN8-2; Q6PNC0
STISNDVFI	9		7.7	Small G protein signaling modulator 2	Q80U12; Q80U12-2
RSIKNVTEL	9		7.8	protein RTF2 homolog	Q99K95
RALSNLESV	9		7.8	Ubiquilin-4	Q99NB8
SAALNKDFL	9		7.8	Centrosome-associated protein 350	E9Q309
LSLPNTDYI	9		7.8	Interferon-inducible double-stranded RNA-dependent protein kinase activator A	Q9WTX2
YSVRNIFHL	9		7.9	Centromere/kinetochore protein zw10 homolog	O54692
SQVINPTAI	9		7.9	Host cell factor 1	Q61191
TAILNDYEL	9		7.9	Isoform 1 of Ninein-like protein	Q6ZQ12-1; Q6ZQ12-2; Q6ZQ12
NAIVNTSEM	9		7.9	Protein Mis18-beta	A2AQ14
YQYTNSEVL	9		7.9	Exportin-T	Q9CRT8
RQLENGTTL	9		8	Extended synaptotagmin-2	Q3TZZ7-2; Q3TZZ7-1
SGLVNHVPL	9		8	Aminoacyl tRNA synthase complex-interacting multifunctional protein 1	P31230
CMLSNTTAI	9	1xOxidation [M2]	8	Tubulin alpha-3 chain	P05214; Q9JJZ2; Q3UX10; P68369; P05213; P68373; P68368
FAHHNRYVL	9		8.2	Tyrosine-protein kinase Fes/Fps	P16879
YVHVNRDTL	9		8.3	Bifunctional polynucleotide phosphatase/kinase	Q9JLV6-1; Q9JLV6-2
FGLSNYHQL	9		8.5	Regulator of chromosome condensation	Q8VE37
SMLGNYDEM	9		8.5	AF4/FMR2 family member 4	Q9ESC8
AALCNVCEL	9		8.5	Trafficking protein particle complex subunit 11	B2RXC1
ASVSNPLFL	9		8.6	exostosin-2	P70428
VSVANVDLL	9		8.6	Lethal(2) giant larvae protein homolog 1	Q80Y17
VMIPNVETI	9		8.6	Putative sodium-coupled neutral amino acid transporter 10	Q5I012-5; Q5I012-3; Q5I012; Q5I012-6; Q5I012-2; Q5I012-4
SQVLNFEEI	9		8.7	mitogen-activated protein kinase kinase kinase 7	Q62073
SALNNFQVV	9		8.7	Syntaxin-12	Q9ER00
SAVRNGLLL	9		8.7	Cullin-9	Q80TT8; Q80TT8-4
GSLKNVTTL	9		8.7	Erbin	Q80TH2-2; Q80TH2; Q80TH2-1
KSLKNFITI	9		8.8	Cysteine--tRNA ligase, cytoplasmic	Q9ER72; Q9ER72-2
ASLSNLHSL	9		8.9	Protein chibby homolog 1	Q9D1C2
SQISNGSHM	9		8.9	Serine/threonine-protein kinase TAO1	Q5F2E8
AMPINKATI	9		8.9	ATP-dependent zinc metalloprotease YME1L1	O88967
SSLANILEL	9		8.9	Translational activator of cytochrome c oxidase 1	Q8K0Z7
SSVVNFQII	9		8.9	Zinc finger CCCH-type antiviral protein 1-like	Q8BFR1; Q8BFR1-2
RQIFNGTFV	9		9	60S ribosomal protein L7	P14148
KAVLNSEVL	9		9	Shootin-1	Q8K2Q9-1; Q8K2Q9-2
SAIINEDNL	9		9	protein EFR3 homolog A	Q8BG67-2; Q8BG67-1

FGPVNHEEL	9		9.1	Cyclin-dependent kinase inhibitor 1B	P46414
MMHSNMETL	9		9.1	Protein diaphanous homolog 1	O08808
YTATNQDFI	9		9.1	Nischarin	Q80TM9-3; Q80TM9-2; Q80TM9
TALENLIVL	9		9.2	E3 ubiquitin-protein ligase hectd1	Q69ZR2
YSHFNETLL	9		9.3	Multidrug resistance-associated protein 1	O35379
HSLENCTTM	9		9.3	Leucyl-cystinyl aminopeptidase	Q8C129
KSLENWDYF	9		9.4	NLR family CARD domain-containing protein 4	Q3UP24
QQIVNIDLM	9		9.4	Ufm1-specific protease 2	Q99K23
FQNVNSVTL	9		9.5	Thioredoxin-like protein 1	Q8CDN6
FAVVNHQGTL	10		9.5	ubiquitin carboxyl-terminal hydrolase 22	Q8CEG8; Q5DU02
AALPNIYEL	9		9.5	DNA replication licensing factor MCM5	P49718
AMLTNLESL	9		9.6	anaphase-promoting complex subunit 2	Q8BZQ7
AAPRNKHWL	9		9.6	G-patch domain and KOW motifs-containing protein	Q56A08
YMPANGETV	9		9.6	Integrator complex subunit 11	Q9CWS4
WAFKNPDTI	9		9.7	Isoform 2 of Endoplasmic reticulum-Golgi intermediate compartment protein 3	Q9CQE7-2; Q9CQE7
VMVTNVTSL	9		9.7	Talin-2	Q71LX4; P26039
AAPQNFTPSM	10		9.7	AP-3 complex subunit beta-1	Q9Z1T1
AAVENVSEL	9		9.7	Biorientation of chromosomes in cell division protein 1-like 1	E9Q6J5; E9Q6J5-3; E9Q6J5-2
KQLENGYTL	9		9.7	E3 ubiquitin-protein ligase UHRF2	Q7TMI3; Q7TMI3-3; Q7TMI3-2
SQLRNEVAI	9		9.8	Serine/threonine-protein kinase D1	Q8K1Y2; Q62101; Q8BZ03
RAGTNLTTL	9		9.8	BAH and coiled-coil domain-containing protein 1	Q3UHR0
SSIQNGKYTL	10		9.8	Lysine-specific demethylase 7A	Q3UWM4
SAVTDFDFI	9		9.8	52 kDa repressor of the inhibitor of the protein kinase	Q9CUX1
FAPENTCHL	9		9.9	Inositol 1,4,5-trisphosphate receptor-interacting protein-like 1	A2ASA8
ISPVNPVAI	9		9.9	RNA-binding protein 47	Q91WT8-1
FALSNEHYSL	10		9.9	Leucine zipper protein 1	Q8R4U7
YMPQNPCII	9	xCarbamidomethyl [C7	10	Histone-binding protein RBBP4	Q60972
ASHLNLDAL	9		10.1	E3 ubiquitin-protein ligase TRIM32	Q8CH72
SGLQNVPPL	9		10.1	Oxidative stress-responsive serine-rich protein 1	Q9D722
RSGLNATFM	9		10.1	Ribosomal protein S6 kinase alpha-4	Q9Z2B9
VALENANAV	9		10.1	Nucleolar protein 56	Q9D6Z1
WSMENLEEI	9		10.1	DNA-directed RNA polymerase II subunit RPB2	Q8CFI7
KQIENGVYL	9		10.2	Isoform 2 of Protein odr-4 homolog	Q4PJX1-2; Q4PJX1-1
SAVWNSPPL	9		10.2	Inner centromere protein	Q9WU62-2; Q9WU62-1
SSYENPWTI	9		10.2	Cardiolipin synthase (CMP-forming)	Q80ZM8
LQLLNTDYL	9		10.2	Pericentriolar material 1 protein	Q9R0L6; Q9R0L6-2
MSMTNTHEL	9		10.3	Protein FAM26F	Q8C9E8
SALRMADVI	9		10.3	Putative pre-mRNA-splicing factor ATP-dependent RNA helicase DHX32	Q8BZS9-2; Q8BZS9-1
VALLNLENM	9	1xOxidation [M9]	10.3	Protein RRP5 homolog	Q6NS46
VSILNRQVL	9		10.4	nuclear mitotic apparatus protein 1	E9Q7G0
HAIENIDTF	9		10.4	Breast cancer type 2 susceptibility protein homolog	P97929
YSHWNLILI	9		10.4	Probable palmitoyltransferase ZDHHC16	Q9ESG8
SSVLNVVSM	9		10.4	Histone-lysine N-methyltransferase 2A	P55200-2; P55200
VGLSNLQFI	9		10.4	Solute carrier family 23 member 2	Q9EPR4
LSMRNTSVM	9		10.5	BCL2/adenovirus E1B 19 kDa protein-interacting protein 3	O55003
VGIENIHVM	9		10.6	Myotubularin-related protein 6	Q8VE11
GQITNFEYL	9		10.6	Lysosomal-trafficking regulator	P97412
GSLSNSVFL	9		10.6	Thioredoxin domain-containing protein 16	Q7TN22; Q7TN22-2
MSIENQEEL	9		10.7	Centromere-associated protein E	Q6RT24
IAVANAQEL	9		10.7	Mitochondrial import inner membrane translocase subunit Tim13	P62075
TSVENHEFL	9		10.8	protein elys	Q8CJF7
KANENASFL	9		10.8	CAP-Gly domain-containing linker protein 1	Q922J3-2; Q922J3

SSVQNYFHL	9		10.8	Trafficking kinesin-binding protein 1	Q6PD31; Q6PD31-2
AAINNRLEL	9		10.8	Palmitoyltransferase ZDHHC13	Q9CWU2
SAIQNLHSF	9		10.9	eukaryotic translation initiation factor 1	P48024
ASYVNLPTI	9		10.9	40S ribosomal protein SA	P14206
FAIINSNPIEL	11		11	Transmembrane protein 131	O70472
SQMTNLQEL	9		11	Volume-regulated anion channel subunit LRRC8D	Q8BGR2
FTVTNTDCL	9		11	DNA polymerase epsilon subunit 2	O54956
KSLLNRYAV	9		11.1	Translocon-associated protein subunit beta	Q9CPW5
FGIKNMDQV	9		11.1	Protein C-ets-2	P15037
VSLINAHSL	9		11.2	E3 ubiquitin-protein ligase RNF31	Q924T7; Q924T7-2
SQLLNLTQL	9		11.2	Erbin	Q80TH2-2; Q80TH2; Q80TH2-1
ALIQNADTL	9		11.2	SH3 domain-binding protein 1	P55194
KALVNAVKL	9		11.2	Vacuolar protein sorting-associated protein 13B	Q80TY5
SMNVNEIFM	9		11.3	Ras-related protein Rab-5A	Q9CQD1
KSLENILTL	9		11.4	F-box DNA helicase 1	Q8K2I9-1
IQVRNMATL	9		11.4	ATP synthase subunit gamma, mitochondrial	Q91VR2
SALVNHMIV	9		11.4	Small G protein signaling modulator 1	Q8BPQ7; Q8BPQ7-2
QGLVNRAYI	9		11.5	Exocyst complex component 6B	A6H5Z3; A6H5Z3-2
HGITNLCVI	9	xCarbamidomethyl [C7	11.5	ATP-dependent 6-phosphofructokinase, liver type	P12382
SSPSNLQII	9		11.5	Ubiquitin carboxyl-terminal hydrolase 24	B1AY13
STYINASYI	9		11.5	Receptor-type tyrosine-protein phosphatase C	P06800-5; P06800-6; P06800
SAHSNINQI	9		11.5	Tribbles homolog 2	Q8K4K3
GALKNTDYF	9		11.7	Cytosolic 5'-nucleotidase 3A	Q9D020; Q9D020-1
FGATNWDLI	9		11.7	Protein RCC2	Q8BK67
YLVVNPNYL	9		11.8	DNA replication licensing factor MCM6	P97311
SQHVNLDQL	9		11.9	Beclin 1-associated autophagy-related key regulator	Q8CDJ3
KAVKNWQFV	9		11.9	Ribonuclease H2 subunit A	Q9CWY8
SGPSNLLEI	9		11.9	T-cell-interacting, activating receptor on myeloid cells protein 1	B6A8R8
RALSNTLLL	9		11.9	Exportin-6	Q924Z6-2; Q924Z6
YGIRNSLLI	9		12	Ribonucleoside-diphosphate reductase large subunit	P07742
AALVNVQIPL	10		12	ATP-binding cassette sub-family B member 8, mitochondrial	Q9CXJ4
AALKNLPLI	9		12	Long-chain-fatty-acid--CoA ligase 3	Q9CZW4
SSPANISSL	9		12	Calpastatin	P51125-4; P51125-7; P51125-6; P51125-5; P51125; P51125-3; P51125-2
SAVENVVKL	9		12.1	Phospholipase A1 member A	Q8VI78
SGVSNPHVI	9		12.1	YEATS domain-containing protein 2	Q3TUF7-2; Q3TUF7; Q3TUF7-3
RAVQNHNTYN	10		12.1	Adhesion G protein-coupled receptor E1	Q61549
FAPKNIYSI	9		12.1	Macrophage colony-stimulating factor 1 receptor	P09581
SSPRNHLAM	9		12.1	Regulator of G-protein signaling 12	Q8CGE9
LAIRNDEEL	9		12.2	Histone H2AX	Q64523; Q8BFU2; P22752; Q8CGP5; Q8R1M2; P27661; Q8CGP7; Q8CGP6; Q6GSS7
TGIRNLEWL	9		12.2	Transmembrane protein 260	Q8BMD6-1; Q8BMD6-2
SSPSNPAAL	9		12.2	Retinoic acid-induced protein 1	Q61818; Q61818-2
FSPFNPTSL	9		12.3	ETS-related transcription factor Elf-4	Q9Z2U4
AAVENLPTFL	10		12.3	Importin subunit beta-1	P70168
SAYQNHLVL	9		12.3	Anoctamin-10	Q8BH79; Q8BH79-3; Q8BH79-2; Q8BH79-4
YAASNGVQM	9		12.3	Ornithine decarboxylase	P00860
SAPENAVRM	9		12.4	Protein C10	O35127
GAIRNACQM	9		12.4	Cullin-3	Q9JLV5
SGLQNFEAL	9		12.4	Protein unc-45 homolog A	Q99KD5
KAISNSHYVL	10		12.4	autophagy-related protein 2 homolog B	Q80XK6
STVVNADQI	9		12.5	ATP-binding cassette sub-family B member 6, mitochondrial	Q9DC29

YQLENIQVL	9	12.5	Citron Rho-interacting kinase	P49025-4; P49025; P49025-3; P49025-5
AAIGNQLYV	9	12.5	Rab9 effector protein with kelch motifs	Q8VCH5
VSPLNVTAV	9	12.5	WASH complex subunit 3	Q9CR27
YSMLNAYSNL	10	12.5	Probable ribonuclease ZC3H12C	Q5DTV4
ASLKNLCEM	9	12.5	Zinc finger SWIM domain-containing protein 3	Q8CFL8
FSPTNPAHL	9	12.6	WD repeat-containing protein 76	A6PWY4-1; A6PWY4-3; A6PWY4-2
SQLQNEFYL	9	12.6	Collagen alpha-2(VI) chain	Q02788
RALENRVAV	9	12.6	NIF3-like protein 1	Q9EQ80
LSVLNHHFI	9	12.7	GPI inositol-deacylase	Q3UUQ7-1
SAIHNSTKV	9	12.8	Interferon-induced very large GTPase 1	Q80SU7
YALENFVENL	10	12.8	Importin-4	Q8VI75
VGLANLHAM	9	13	U4/U6 small nuclear ribonucleoprotein Prp3	Q922U1
VAPVNLQHDF	11	13	Fanconi anemia group D2 protein homolog	Q80V62
YASTNLEWL	9	13	Cytochrome c oxidase subunit 1	P00397
SALENGRYEL	10	13.1	Bromodomain adjacent to zinc finger domain protein 1A	O88379
GSLANHTSI	9	13.1	Vacuolar protein sorting-associated protein 33A	Q9D2N9
VAVNNTECL	9	13.1	Zinc finger FYVE domain-containing protein 16	Q80U44
IGIENIHYL	9	13.2	malignant T-cell-amplified sequence 2	Q9DB27-2; Q9CQ21; Q9DB27
AQHVNLAVL	9	13.2	GON-4-like protein	Q9DB00
STPVNVTSL	9	13.2	Kinesin-like protein KIF23	E9Q5G3
YSLVNQQSF	9	13.2	Ras-related protein Rap-2c	Q8BU31; P61226; Q80ZJ1-2; Q80ZJ1
VAYMNPIAM	9	13.3	Protein FAM133B	Q9CVI2; Q9CVI2-2
ASPANVGTL	9	13.3	Guanine nucleotide exchange protein SMCR8	Q3UMB5
QSLRNIHTL	9	13.3	E3 ubiquitin-protein ligase synoviolin	Q9DBY1-2; Q9DBY1
YTIQNRVDI	9	13.5	Cohesin subunit SA-1	Q9D3E6
YSGANSIFL	9	13.5	Bystin	O54825
GALSNLEQL	9	13.5	Volume-regulated anion channel subunit LRRC8B	Q5DU41; Q5DU41-2
SSLVNGSTF	9	13.5	Magnesium transporter NIPA1	Q8BHK1
TTLTNSTPL	9	13.6	Protein fem-1 homolog C	Q8CEF1
LSVRNGATL	9	13.6	Peroxisome proliferator-activated receptor gamma coactivator 1-beta	Q8VHJ7-2; Q8VHJ7
FQWRNLNTI	9	13.7	Protein asteroid homolog 1	Q8BIR2; Q8BIR2-2
NMPWNVDTL	9	13.7	Hsp90 co-chaperone Cdc37	Q61081
AALQNAVAF	9	13.7	E3 ubiquitin-protein ligase TRIM56	Q80VI1
SSVENYYVFL	10	13.7	Isoform 2 of Tetratricopeptide repeat protein 39B	Q8BYY4-2
ISGVNRYYV	9	13.8	NADH dehydrogenase [ubiquinone] 1 alpha subcomplex subunit 1	O35683
RSPFNACVL	9	13.9	glycerophosphodiester phosphodiesterase 1	Q9JL56
SAIFNFQSL	9	13.9	Protein kish-A	Q9CR64-1; Q9CR64-2
LSLENIATL	9	14	Protein regulator of cytokinesis 1	Q99K43
AVLTNQETI	9	14.1	Brefeldin A-inhibited guanine nucleotide-exchange protein 3	Q3UGY8
ASLQNAEKTM	10	14.1	Ubiquitin carboxyl-terminal hydrolase 38	Q8BW70
TSNMNSSYI	9	14.1	Transcriptional regulator ATRX	Q61687
FALINKLDI	9	14.2	Arginyl-tRNA--protein transferase 1	Q9Z2A5-2; Q9Z2A5
YTLRNQDTF	9	14.2	period circadian protein homolog 1	O35973
STIKNANFV	9	14.2	ATP-binding cassette sub-family B member 10, mitochondrial	Q9JI39
HSGMNATTI	9	14.3	N-acetylglucosamine-1-phosphotransferase subunits alpha/beta	Q69ZN6; Q69ZN6-2
YSIGNLQKI	9	14.4	Origin recognition complex subunit 5	Q9WUV0
ASVLNVNHI	9	14.4	Ankyrin repeat domain-containing protein 17	Q99NH0-1
KAIRNYTHF	9	14.4	R degradation-enhancing alpha-mannosidase-like protein 2 OS=Mus musculus OX=1009	Q8BJT9
VQIKNDVFI	9	14.5	Kelch domain-containing protein 10	Q6PAR0-3; Q6PAR0-2; Q6PAR0
SLLSNLDEI	9	14.5	Programmed cell death 6-interacting protein	Q9WU78-3; Q9WU78
KAILNGIDSI	10	14.6	Structural maintenance of chromosomes protein 3	Q9CW03
AAPQNFTPSM	11	14.6	AP-3 complex subunit beta-1	Q9Z1T1

YAIKNIHGI	9		14.6	Dynamin-1	P39053; P39053-3; P39053-4; Q8BZ98; Q8BZ98-2; P39053-5; P39053-6
FAPVNVTTEVI	13		14.6	Elongation factor 1-alpha 1	P10126
TSIPNFSYM	9	1xOxidation [M9]	14.7	Polyadenylate-binding protein-interacting protein 1	Q8VE62
NALRNFACL	9		14.7	Ubiquitin recognition factor in ER-associated degradation protein 1	P70362
FAQNNVTEI	9		14.8	Solute carrier family 12 member 7	Q9WVL3; Q9WVL3-2
LALENEGYI	9		14.8	Serine/threonine-protein phosphatase 4 regulatory subunit 3A	Q922R5-2; Q6P2K6; Q922R5
RAILNDTKL	9		14.9	Rab GTPase-binding effector protein 1	O35551-6; O35551-1; O35551-3; O35551-2
GAHVNAQTV	9		14.9	Ankyrin repeat and SOCS box protein 11	Q9CQ31-1; Q9CQ31-2
FQHRNHTCL	9		14.9	Homeodomain-interacting protein kinase 3	Q9ERH7
GSLRNLEEL	9		14.9	Baculoviral IAP repeat-containing protein 1e	Q9R016; Q9QWK5; Q9JIB6; Q9JIB3
YTVANKEYV	9		15	Endoplasmic reticulum-Golgi intermediate compartment protein 1	Q9DC16
KGIVNEQFL	9		15	Very long-chain specific acyl-CoA dehydrogenase, mitochondrial	P50544
YQYINCSTL	9		15	Mitotic checkpoint serine/threonine-protein kinase BUB1 beta	Q9Z1S0
SSPFNASSL	9		15	Suppression of tumorigenicity 18 protein	Q80TY4
KAHMNSVLM	9		15.1	Charged multivesicular body protein 3	Q9CQ10
NSQVNFDFI	9		15.1	Ral guanine nucleotide dissociation stimulator-like 1	Q60695
AALLNFDEF	9		15.1	FH1/FH2 domain-containing protein 1	Q6P9Q4
SGLLNPYSL	9		15.2	DNA helicase ino80	Q6ZPV2-2; Q6ZPV2-1
YAGQFNQDYL	10		15.3	Annexin A11	P97384
NSLTNIQWL	9		15.3	Forkhead box protein M1	O08696
YAPMGMHPM	9	1xOxidation [M4]	15.3	Pre-mRNA-processing factor 40 homolog A	Q9R1C7-2; Q9R1C7
KSMENLIEL	9		15.4	Tubby-related protein 3	O88413
NSLVNICLI	9	xCarbamidomethyl [C7	15.5	E3 ubiquitin-protein ligase RNF26	Q8BUH7
TQIRNRALI	9		15.5	COP9 signalosome complex subunit 1	Q99LD4; Q99LD4-2
GAIENAFHL	9		15.6	Protein phosphatase 1J	Q149T7
RAAANSETI	9		15.6	GON-4-like protein	Q9DB00
LAVVNGANI	9		15.6	Baculoviral IAP repeat-containing protein 6	O88738-2; O88738; O88738-3
SSPRNSDWF	9		15.6	Serine/threonine-protein kinase ULK2	Q9QY01
AALENPMLL	9		15.8	Ubiquitin-like modifier-activating enzyme ATG7	Q9D906
IGVSNFNPL	9		15.8	Aldo-keto reductase family 1 member B1	P45376
VSLNNLIYV	9		15.8	Kelch-like protein 24	Q8BRG6
SAVENKDKL	9		15.9	SprT-like domain-containing protein Spartan	G3X912
YTFVNPSVL	9		15.9	Plexin-A2	P70207
GSPENEEYI	9		15.9	Regulatory-associated protein of mTOR	Q8K4Q0-2; Q8K4Q0
ISWHNIDYI	9		16	Unconventional myosin-IXa	Q8C170-2; Q8C170
QAHLNLETL	9		16	N-acylethanolamine-hydrolyzing acid amidase	Q9D7V9
AMNVNEIFM	9	1xOxidation [M9]	16.1	Ras-related protein Rab-5C	P35278
GSIVNEGYL	9		16.2	Synaptogyrin-1	O55100; O55100-2
ISIMNGSSV	9		16.3	Polyadenylate-binding protein-interacting protein 2B	Q91W45
AAGSNQVTL	9		16.4	Serine/threonine-protein kinase greatwall	Q8C0P0
YAFYNTEQL	9		16.5	\F5-like RNA polymerase II p300/CBP-associated factor-associated factor 65 kDa subunit	Q91WQ5
VALRNDSYTL	10		16.6	26S proteasome regulatory subunit 8	P62196
SAMSNPRAM	9		16.7	Ubiquilin-1	Q8R317-2; Q8R317
RSFENNTYL	9		16.7	RecQ-mediated genome instability protein 1	Q9D4G9
YSGVNVTDL	9		16.7	[F-actin]-methionine sulfoxide oxidase MICAL3	Q8CJ19-3; Q8CJ19-1; Q8CJ19-2
FSYLNPQEL	9		16.8	F-box/LRR-repeat protein 5	Q8C2S5-3; Q8C2S5-4; Q8C2S5; Q8C2S5-2; Q8C2S5-5
SSPQNQEQI	9		16.8	Metal regulatory transcription factor 1	Q07243
YGIQNSYVKL	10		16.9	Vacuolar protein sorting-associated protein 33A	Q9D2N9
SSPENKNWL	9		16.9	Denticleless protein homolog	Q3TLR7-2; Q3TLR7
GSLSNHTEL	9		17	Tonsoku-like protein	Q6NZL6
SALANGRAL	9		17.1	Homeobox protein DLX-1	Q64317

QAVLNLHSI	9		17.1	Isoform 2 of DIS3-like exonuclease 2	Q8CI75-2; Q8CI75
KMIINEELM	9		17.1	Eukaryotic translation initiation factor 3 subunit C	Q8R1B4
YQHINSYQL	9		17.1	lated matrix-associated actin-dependent regulator of chromatin subfamily A containing DE	Q04692-2; Q04692
AALNNHVEV	9		17.1	E3 ubiquitin-protein ligase MIB1	Q80SY4
YSPQNSDYLL	10		17.2	Sortilin	Q6PHU5; Q6PHU5-2
FSPMNRTPVLI	11		17.4	Aminoacylase-1	Q99JW2
IALFNQELV	9		17.4	Isoform 1 of Paired amphipathic helix protein Sin3b	Q62141-1; Q62141
GGVVNMYHM	9		17.5	Proteasome subunit beta type-8	P28063
SSIVNSIQV	9		17.5	Ribosome-binding protein 1	Q99PL5-10; Q99PL5-11; Q99PL5-6; Q99PL5-12; Q99PL5-4; Q99PL5-9; Q99PL5-7; Q99PL5-1; Q99PL5-8; Q99PL5-5; Q99PL5-3; Q99PL5-2
SSIRNFLIYV	10		17.5	Isoform 3 of Mitochondrial import receptor subunit TOM5 homolog	B1AXP6-2; B1AXP6-1; B1AXP6-4; B1AXP6-3
SSLSSITPM	9		17.5	Centrosomal protein of 164 kDa	Q5DU05-3; Q5DU05; Q5DU05-2
RGIVNLSEL	9		17.6	Decaprenyl-diphosphate synthase subunit 2	Q33DR3; Q33DR3-2; Q33DR3-3
KATINIDAI	9		17.6	Fas apoptotic inhibitory molecule 1	Q9WUD8
DAVKNGDYI	9		17.6	M-phase phosphoprotein 8	Q3TYA6
STIRNAQSI	9		17.7	Neutrophil cytosol factor 1	Q09014
SQPVNPHSL	9		17.7	Zinc finger SWIM domain-containing protein 8	Q3UHH1-2; Q3UHH1; Q3UHH1-3; Q3UHH1-4
GSLINPNVI	9		17.8	EKC/KEOPS complex subunit Tprkb	Q8QZZ7
GANVNHTTV	9		17.9	Protein fem-1 homolog B	Q9Z2G0
FQVRNLPTL	9		18	Uncharacterized protein C12orf29 homolog	Q8BHN7; Q8BHN7-2
SAVKNLQQL	9		18	GATOR complex protein NPRL3	Q8VIJ8
GAFSNPETL	9		18.1	acetoacetyl-CoA synthetase	Q9D2R0
TQVLNTHYV	9		18.1	Transcription activator BRG1	Q3TKT4-1; Q6DIC0; Q3TKT4-2
SAHQNYAEWL	10		18.1	Ribosome-binding protein 1	Q99PL5-1
TTVTNSTPL	9		18.1	Protein fem-1 homolog B	Q9Z2G0
FALKNPFYSL	10		18.2	Trafficking protein particle complex subunit 4	Q9ES56
FSFRNTQEV	9		18.3	Interferon-induced very large GTPase 1	Q80SU7
RGLENLTLL	9		18.3	Toll-like receptor 13	Q6R5N8
AALVNPDSF	9		18.4	Zinc fingers and homeoboxes protein 1	P70121
STITNPSAI	9		18.4	Ubiquitin carboxyl-terminal hydrolase 42	B2RQC2
NGVINAAFM	9		18.4	Clathrin coat assembly protein AP180	Q61548; Q7M6Y3-1; Q7M6Y3-5; Q61548-2; Q7M6Y3-6; Q7M6Y3-4; Q7M6Y3-2; Q7M6Y3-3; Q61548-3
GSIINISSI	9		18.4	Estradiol 17-beta-dehydrogenase 8	P50171; P50171-2
SAMVSIEYV	9		18.4	E3 ubiquitin-protein ligase RNF149	Q3U2C5
VSVTNSMTV	9	1xOxidation [M7]	18.5	Ubiquitin-like protein ISG15	Q64339
TQPLNHYFI	9		18.5	1-phosphatidylinositol 4,5-bisphosphate phosphodiesterase beta-2	A3KGF7; A3KGF7-3; A3KGF7-4; A3KGF7-2
SGPTNEDLYI	10		18.6	3-hydroxy-3-methylglutaryl-coenzyme a reductase	Q01237
HALVNIRAI	9		18.6	Armadillo-like helical domain-containing protein 3	Q6PD19; Q6PD19-2
DALTNLTVL	9		18.7	Protein phosphatase 1 regulatory subunit 7	Q3UM45
GAVKNLTYF	9		18.7	CMP-N-acetylneuraminate-beta-galactosamide-alpha-2,3-sialyltransferase 4	Q91Y74
IMVRNIDLV	9		18.8	vesicle-associated membrane protein 7	P70280
FATSNQISL	9		18.8	COMM domain-containing protein 7	Q8BG94
FGLSNQLVV	9		18.8	Mucolipin-1	Q99J21-2; Q8K595-2; Q99J21; Q8K595
ISPQNMDIL	9		18.8	Alpha-N-acetylgalactosaminidase	Q9QWR8
TTVTNPEPM	9		18.9	DNA (cytosine-5)-methyltransferase 1	P13864-2; P13864
AQVRNQGYL	9		19.1	Centrosomal protein kizuna	Q3UXL4
AGPTNVHLI	9		19.1	homeobox protein SIX5	P70178
FSLHNPYNL	9		19.2	T-box transcription factor TBX15	O70306
SSPRNSQEL	9		19.2	caspase recruitment domain-containing protein 9	A2AIV8
ASVLNTDPAL	10		19.2	dipeptidyl peptidase 3	Q99KK7
SAIHNFYDNI	10		19.2	TGF-beta-activated kinase 1 and MAP3K7-binding protein 2	Q99K90

VSPTNPTKL	9		19.2	Disabled homolog 2-interacting protein	Q3UHC7-3; Q3UHC7-2; Q3UHC7
RQIGNRDTL	9		19.3	Mitochondrial antiviral-signaling protein	Q8VCF0
AAVLNPRFL	9		19.3	C2 domain-containing protein 5	Q7TPS5-3; Q7TPS5-2; Q7TPS5
NAVRNLQEL	9		19.3	Centrosomal protein of 70 kDa	Q6IQY5-2; Q6IQY5
AQILNQVMM	9		19.4	Mitotic checkpoint serine/threonine-protein kinase BUB1	O08901
FSNKNLEEL	9		19.4	DIS3-like exonuclease 1	Q8C0S1; Q8C0S1-2
SMISNDSETL	10		19.4	E3 ubiquitin-protein ligase MIB1	Q80SY4
FSVVNPASF	9		19.6	Rho-related GTP-binding protein RhoQ	Q8R527
AALVSRTFL	9		19.7	ATP-binding cassette sub-family D member 1	P48410
FSTWNLDHI	9		19.7	DNA fragmentation factor subunit beta	O54788
SSLVNKEDVL	10		19.7	Nodal modulator 1	Q6GQT9
RALKNFSSL	9		19.8	Ral guanine nucleotide dissociation stimulator	Q03385
SAVRNHTYQM	11		19.8	FTS and Hook-interacting protein	Q3U2I3-3; Q3U2I3-2; Q3U2I3
AMGVNLTSM	9	1xOxidation [M9]	19.9	proliferating cell nuclear antigen	P17918
AALENDKTI	9		19.9	WD repeat-containing protein 5	P61965; Q9D7H2
NQPVNTAVL	9		19.9	Activity-dependent neuroprotector homeobox protein 2	Q8CHC8
AGIYNTTEL	9		20	Ubiquinone biosynthesis protein COQ9, mitochondrial	Q8K1Z0
RAVANRTDAC	12		20	26S proteasome regulatory subunit 7	P46471
NQLKNTSTI	9		20.1	Probable ATP-dependent RNA helicase DDX6	P54823
KSYVNPTEL	9		20.1	Ral GTPase-activating protein subunit beta	Q8BQZ4-1
SVLFNLTTM	9	1xOxidation [M9]	20.1	Trafficking protein particle complex subunit 12	Q8K2L8
SAVPNQISI	9		20.1	E3 SUMO-protein ligase PIAS2	Q8C5D8-2; Q8C5D8-5; Q8C5D8-4; Q8C5D8-3; Q8C5D8
FALNNPEMV	9		20.3	Protein NDRG1	Q62433
KGLLNAIVI	9		20.3	Ornithine aminotransferase, mitochondrial	P29758
SMAENSIPL	9	1xOxidation [M2]	20.3	Forkhead box protein P1	P58462-3; P58462-4; P58462-1; P58462-2; P58462-5
YSVSNSVKEI	10		20.3	Cathepsin B	P10605
SALANYDET	9		20.3	DNA polymerase epsilon catalytic subunit A	Q9WVF7
SGVWNVTEL	9		20.4	Nuclear factor 1 X-type	P70257; P70257-2; P70257-1
TALKNPNAM	9		20.5	Ras GTPase-activating-like protein IQGAP1	Q9JKF1
YAADNQIYI	9		20.5	Heterogeneous nuclear ribonucleoprotein L	Q8R081
SQALNTTVL	9		20.5	Triggering receptor expressed on myeloid cells 2	Q99NH8; Q99NH8-2
YMGTNIHSL	9		20.7	Inositol polyphosphate 1-phosphatase	P49442
SALINLVEF	9		20.7	DNA-dependent protein kinase catalytic subunit	P97313; P97313-2
FVLENFSTL	9		20.9	E3 ubiquitin-protein ligase TRIM37	Q6PCX9
AAATNPSWL	9		21	Integrin alpha-X	Q9QXH4
SGLENTAAL	9		21	Melanoma inhibitory activity protein 2	Q91ZV0; Q91ZV0-3
AAVRNCDEFL	10		21.1	Cytoplasmic aconitate hydratase	P28271
SSPVNPVVF	9		21.2	peptidyl-prolyl cis-trans isomerase H	Q9D868; Q9D868-2
MGLSNVTVV	9		21.2	Up-regulator of cell proliferation	Q5NCI0-2; Q5NCI0
WAILNEIHI	9		21.4	Alpha-1,3-mannosyl-glycoprotein 4-beta-N-acetylglucosaminyltransferase A	Q812G0-2; Q812G0
VSYRNIEEL	9		21.5	Nucleoprotein TPR	F6ZDS4
TALNNPYYYL	10		21.5	Origin recognition complex subunit 3	Q9JK30-2; Q9JK30
AGTRNIYYL	9		21.6	heat shock protein 75 kDa, mitochondrial	Q9CQN1
SSTANIIVM	9		21.6	Solute carrier family 12 member 2	P55012
ASLVNSPSYL	10		21.7	Transmembrane protein 131-like	Q3U3D7-2; Q3U3D7-1; Q3U3D7-3
YMPQNPHII	9		21.8	Histone-binding protein RBBP7	Q60973
SSIVNSIQVL	10		21.8	Ribosome-binding protein 1	Q99PL5-10; Q99PL5-11; Q99PL5-6; Q99PL5-12; Q99PL5-4; Q99PL5-9; Q99PL5-7; Q99PL5-1; Q99PL5-8; Q99PL5-5; Q99PL5-3; Q99PL5-2
LMLDNYDTM	9		21.8	Ribosomal biogenesis protein LAS1L	A2BE28-2; A2BE28
KQIANIDRI	9		21.9	Zinc finger MYND domain-containing protein 11	Q8R5C8
VQPFNYVTL	9		22.1	Protein fem-1 homolog A-A	Q8C0T1; Q9Z2G1

TSIKNQTQL	9		22.1	Pseudouridylate synthase 7 homolog	Q91VU7; Q91VU7-2
GSLVNQNLL	9		22.3	Forkhead box protein O3	Q9WVH4
IANINQELI	9		22.4	F-box only protein 38	Q8BMI0
YMLKNVRVL	9		22.5	Neuropathy target esterase	Q3TRM4; Q3TRM4-2; Q3TRM4-4; A2AJ88-3; A2AJ88; A2AJ88-2; Q3TRM4-3
NANTNTEVL	9		22.5	Charged multivesicular body protein 4b	Q9D8B3
SGLCNEDHL	9		22.5	E3 ubiquitin-protein ligase NEDD4	Q8CFI0-2; P46935; Q8CFI0; Q8CFI0-3
SAVENKQQI	9		22.6	Serine/threonine-protein kinase PLK2	P53351
GSPMNTTQL	9		22.6	NF-kappa-B inhibitor zeta	Q9EST8-2; Q9EST8
RSIRNILDI	9		22.6	Tumor necrosis factor alpha-induced protein 2	Q61333
LAVRNDEEL	9		22.7	Histone H2A type 2-B	Q64522
KTLLNPEYL	9		22.7	TBC1 domain family member 5	Q80XQ2
KAMENIGEL	9		22.7	Probable ATP-dependent RNA helicase DDX46	Q569Z5; Q569Z5-2
VAVVNKVDI	9		22.8	Niemann-Pick C1 protein	O35604
GMIENGPFL	9		22.8	Interferon-induced very large GTPase 1	Q80SU7
SSMTDGTPL	9		22.8	Intraflagellar transport protein 57 homolog	Q8BXG3; Q8BXG3-2
RATSNVFAM	9		22.8	Myosin regulatory light chain 12B	Q9CQ19; Q3THE2
SSIVNSTYYA	10		22.8	DNA-binding protein SATB1	Q8VI24-2; Q60611; Q8VI24
RAIKNDSVV	9		22.9	T-complex protein 1 subunit eta	P80313
SALTNCITQL	10		23	Melanoma inhibitory activity protein 3	Q8BI84-1
SSGKNLFYL	9		23.1	Peptidyl-glycine alpha-amidating monooxygenase	P97467
GAVQNIAHL	9		23.2	Armadillo repeat-containing protein 8	Q9DBR3; Q9DBR3-3; Q9DBR3-2
KSITNPRYV	9		23.2	DNA-directed RNA polymerase I subunit RPA1	O35134
ATLSNTQTL	9		23.2	Histone-lysine N-methyltransferase SETDB2	Q8C267; Q8C267-2
YQPYNKDWI	9		23.3	Enhancer of rudimentary homolog	P84089
SQIGNLEAL	9		23.3	Leucine-rich repeat and calponin homology domain-containing protein 3	Q8BVU0
SSTINPVPL	9		23.3	Rab GTPase-activating protein 1	A2AWA9; A2AWA9-3; A2AWA9-2
WAVSNREML	9	1xOxidation [M8]	23.4	Protein RMD5 homolog A	Q80YQ8
TGVTNRDLI	9		23.4	rho GTPase-activating protein 21	Q6DFV3
SMEVNVDTL	9		23.4	Dysbindin domain-containing protein 2	Q9CRD4
FKIINHSFI	9		23.4	Cytochrome b	P00158
SAVVDKDFL	9		23.6	Adenosine kinase	P55264; P55264-2
GAPINVMFL	9		23.6	Acid sphingomyelinase-like phosphodiesterase 3b	P58242
SSGINGSFL	9		23.7	Tyrosine-protein kinase ABL1	P00520-4; P00520-3; P00520-2; P00520
YALEHMITL	9		23.7	Kinetochore-associated protein 1	Q8C3Y4
TSVQNVSQI	9		23.8	C-type lectin domain family 5 member A	Q9R007-3; Q9R007-2
DAIHNFDFL	9		23.9	probable cation-transporting ATPase 13A3	Q5XF89-2; Q5XF89-1
GSIANFTNV	9		23.9	ATP-citrate synthase	Q91V92
IQHSNIVTL	9		24	PAB-dependent poly(A)-specific ribonuclease subunit PAN3	Q640Q5-1
VMLENYNHL	9		24.2	Zinc finger protein 90	Q61967
AAIRNYGIEL	10		24.3	Methylenetetrahydrofolate reductase	Q9WU20
GGLRNVDCL	9		24.4	Serine/threonine-protein kinase TBK1	Q9WUN2
YSYQNRYHF	9		24.5	CREB-binding protein	P45481; B2RWS6
EAIQNSTSI	9		24.5	Cullin-4B	A2A432-2; A2A432
AQIQNITTSPM	11		24.5	Trafficking protein particle complex subunit 13	Q3TIR1-2; Q3TIR1; Q3TIR1-3
SAIMNPASKVI	11		24.6	Clathrin heavy chain 1	Q68FD5
LQLQNLATL	9		24.6	CUGBP Elav-like family member 2	Q9Z0H4; Q9Z0H4-7; Q9Z0H4-11; Q9Z0H4-9; Q9Z0H4-4; Q9Z0H4-8; Q9Z0H4-6; Q9Z0H4-2; Q9Z0H4-3; Q9Z0H4-5
NAIINTLAV	9		24.7	Pleiotropic regulator 1	Q922V4
KSVINTTLV	9		24.7	Lysosome membrane protein 2	O35114
YVLTNVAYF	9		24.7	Cystine/glutamate transporter	Q9WTR6
KAIHNICSV	9		24.9	transportin-3	Q6P2B1-1

HSPTNTVHM	9		24.9	ubiquitin carboxyl-terminal hydrolase 8	Q80U87
LSPINHNTL	9		24.9	Rapamycin-insensitive companion of mTOR	Q6QI06; Q6QI06-2
LSVTNSEEL	9		24.9	Interleukin-6 receptor subunit beta	Q00560
RSAENFTVL	9		24.9	P2X purinoceptor 7	Q9Z1M0
ASHLNNVFV	9		25	exocyst complex component 1	Q8R3S6
SAIFNNVMTL	10	1xOxidation [M8]	25	Equilibrative nucleoside transporter 1	Q9JIM1-1; Q9JIM1-2
YAPANGDFTF	10		25	Protein YIPF4	Q8C407
VMLENYSHL	9		25.1	Zinc finger protein 30	P16372; Q60585; Q62396
AANRNNDAL	9		25.2	Vimentin	P20152
GAIVNGKVL	9		25.2	Ral GTPase-activating protein subunit alpha-1	Q6GYP7-6; Q6GYP7; Q6GYP7-2; Q6GYP7-3
ISLKNSQEI	9		25.4	Interferon-induced very large GTPase 1	Q80SU7
VALDNSVYL	9		25.5	Cell division cycle protein 20 homolog	Q9JJ66
SAATNPNAM	9		25.5	Phosphoenolpyruvate carboxykinase [GTP], mitochondrial	Q8BH04
SAPVNFISA	9		25.5	Tuftelin-interacting protein 11	Q9ERA6
SSLHNEQVL	9		25.6	Membrane-bound transcription factor site-2 protease	Q8CHX6
FQHKNHTCL	9		25.6	Homeodomain-interacting protein kinase 1	O88904-1; Q9QZR5
HAIRNSFQYL	10		25.6	Myotubularin-related protein 4	Q91XS1; Q91XS1-2
MAPQNLSTF	9		25.7	DnaJ homolog subfamily B member 11	Q99KV1
GSIYNPEVL	9		25.9	60S acidic ribosomal protein P0	P14869
YVLHNSNTM	9		26.1	26S proteasome non-ATPase regulatory subunit 2	Q8VDM4
NSLLNQGFL	9		26.3	Myb-related protein B	P48972
KATTNIVEM	9		26.4	T-complex protein 11-like protein 2	Q8K1H7
QQVVNIECI	9		26.7	AP-2 complex subunit alpha-2	P17427
SSATNRITV	9		26.9	E3 SUMO-protein ligase PIAS4	Q9JM05
GAPINKYVV	9		26.9	Fibronectin type-III domain-containing protein 3A	Q8BX90
ASWENPLPL	9		27	Flap endonuclease GEN homolog 1	Q8BMI4; Q8BMI4-2
FQYRNRIAM	9		27.1	Prostaglandin G/H synthase 1	P22437
RGILNWCVV	9	xCarbamidomethyl [C7	27.2	Diacylglycerol O-acyltransferase 1	Q9Z2A7
RSMQNYVQFL	10		27.2	Isoform 4 of Outer dense fiber protein 2	A3KGV1; A3KGV1-4; A3KGV1-3; A3KGV1-2
SAASNPFYF	9		27.2	Lysosomal-trafficking regulator	P97412-2; P97412
SQLQNQANL	9		27.5	Oncostatin-M	P53347
SAPRNFVENF	13		27.5	Elongator complex protein 2	Q91WG4-2; Q91WG4
VAILDSTTI	9		27.5	DNA topoisomerase 2-alpha	Q01320
FSGSNAEQM	9		27.6	Rho guanine nucleotide exchange factor 2	Q60875-3; Q60875; Q60875-5; Q60875-2; Q60875-4
YTLHNIDQL	9		27.6	Zinc finger FYVE domain-containing protein 16	Q80U44
FMISHLDYL	9		27.7	Serine-protein kinase ATM	Q62388
VGIRNTFLL	9		27.8	Poly(A) RNA polymerase GLD2	Q91YI6-1; Q91YI6-2
YAGSNREDVL	10		27.8	26S proteasome non-ATPase regulatory subunit 2	Q8VDM4
AAPRTNGVAM	10		27.8	Protein FAM168A	Q8BGZ2-1; Q8BGZ2-2
SSIHNPTGRSY	13		27.8	Protein arginine N-methyltransferase 5	Q8CIG8
RSLLNSHIL	9		27.8	WD repeat and FYVE domain-containing protein 3	Q6VNB8
WSLANHEYF	9		28	Engulfment and cell motility protein 1	Q8BPU7-3; Q8BPU7-1
YQILNNEVF	9		28.1	Cytoplasmic FMR1-interacting protein 2	Q5SQX6
SQISNTEFLQV	12		28.2	BRISC complex subunit Abraxas 2	Q3TCJ1
KAVTNEQEL	9		28.4	Transcription termination factor 1a, mitochondrial	Q8CHZ9; B9EJ57
SSNINSATL	9		28.4	SLAIN motif-containing protein 2	Q8CI08-2; Q8CI08
AAPINPSDI	9		28.5	Enoyl-[acyl-carrier-protein] reductase, mitochondrial	Q9DCS3
SSVFNVEMV	9		28.5	ATP synthase mitochondrial F1 complex assembly factor 1	Q811I0
RQLENTEHM	9		28.7	Structure-specific endonuclease subunit SLX4	Q6P1D7
GALKNIVAV	9		28.7	Glycerol-3-phosphate dehydrogenase 1-like protein	Q3ULJ0-2; P13707; Q3ULJ0
SALCNSCRL	9	Carbamidomethyl [C4;	28.9	Talin-1	P26039
VTNLNPDFI	9		29	polypyrimidine tract-binding protein 3	Q8BHD7-2; Q8BHD7

EAIVNQEII	9		29	Xylosyltransferase 1	Q811B1
ISGVNGTHI	9		29.1	SRSF protein kinase 1	O70551
ISLHNPVSI	9		29.1	Probable ATP-dependent RNA helicase DDX31	Q6NZQ2
YGGSNYVVV	9		29.1	Nucleoporin Nup37	Q9CWU9
KAPTNVTCIL	10		29.2	von Willebrand factor A domain-containing protein 8	Q8CC88-2; Q8CC88
SAPSCWTTL	9		29.3	Vacuolar protein sorting-associated protein 16 homolog	Q920Q4
YVHVNQYDI	9		29.4	Coatomer subunit beta	Q9JIF7
RIIENVDVI	9		29.4	Actin-related protein 8	Q8R2S9
ASIQNGKDSL	10		29.7	Serine/threonine-protein kinase MARK2	Q05512-2; Q05512
KAKVNQETI	9		29.8	SS18-like protein 2	Q9D174
VAPTNPQAV	9		29.8	39S ribosomal protein L9, mitochondrial	Q99N94
TVIENIHTI	9		29.8	Ubiquitin carboxyl-terminal hydrolase 24	B1AY13
LSHVNEEWL	9		30	Neutrophil cytosol factor 2	O70145
VSLVNQRDEL	10		30	EH domain-binding protein 1-like protein 1	Q99MS7-3; Q99MS7; Q99MS7-4; Q99MS7-5; Q99MS7-2
YSIKCFDTV	9		30.1	TBC1 domain family member 31	Q6NXY1
YEIENQNPL	9		30.2	Nuclear fragile X mental retardation-interacting protein 2	Q5F2E7-2; Q5F2E7
FMECNLDEL	9		30.2	Proteasome subunit alpha type-1	Q9R1P4
IQIGNHNYM	9	1xOxidation [M9]	30.2	Receptor-interacting serine/threonine-protein kinase 1	Q60855
GAACNVWYL	9		30.2	Tensin-3	Q5SSZ5; Q5SSZ5-2
STLINSLFL	9		30.3	Septin-2	P42208; O55131; P42209
AACVNAATL	9		30.4	Exosome complex component RRP41	Q921I9
YTVKNGDSL	9		30.5	Trafficking protein particle complex subunit 10	Q3TLI0
FGLQNDHCV	9		30.6	Transforming acidic coiled-coil-containing protein 3	Q9JJ11; Q9JJ11-2
AAPVSATVL	9		30.7	Long-chain-fatty-acid--CoA ligase 1	P41216
AQQVNRTTL	9		30.7	Transcription initiation factor TFIID subunit 6	Q62311
RSLSNSGVL	9		30.9	Baculoviral IAP repeat-containing protein 6	O88738-2; O88738; O88738-3
AALQNYNNEL	10		31	Sjoegren syndrome nuclear autoantigen 1 homolog	Q9JJ94
SMEVNVDVL	9		31.1	dysbindin	Q91WZ8-2; Q91WZ8-3; Q91WZ8-1
FSFRNTQEVI	10		31.2	Interferon-induced very large GTPase 1	Q80SU7
VALKNKQEL	9		31.2	E3 ubiquitin-protein ligase FANCL	Q9CR14-2; Q9CR14
SQNRNGEFV	9		31.3	Pre-mRNA-splicing factor 38A	Q4FK66-1
VSVENFALL	9		31.3	Ribosomal protein S6 kinase alpha-4	Q9Z2B9
ASLTNPFGKGA	13		31.4	Nucleolar GTP-binding protein 2	Q99LH1
YSHVDLVVM	9		31.5	Serine--tRNA ligase, cytoplasmic	P26638
SMIVDNTTM	9		31.7	Isocitrate dehydrogenase [NAD] subunit gamma 1, mitochondrial	P70404
QTLKNITFI	9		31.7	Macrophage scavenger receptor types I and II	P30204-2; P30204
SAVTNKAYVF	10		31.7	Lysosome membrane protein 2	O35114
TSAQNVEFL	9		31.8	Outer dense fiber protein 2	A3KGV1; A3KGV1-4; A3KGV1-3; A3KGV1-2
FGGSNVHVI	9		32	Fatty acid synthase	P19096
YSIQGQHTI	9		32.1	Poly(RC)-binding protein 1	P60335
AVLTNPETL	9		32.1	Procollagen-lysine,2-oxoglutarate 5-dioxygenase 3	Q9R0E1
GAIGNPEVL	9		32.2	Deoxyhypusine hydroxylase	Q99LN9-2; Q99LN9
HSLLNKATV	9		32.4	Neurofibromin	Q04690-3; Q04690
KSVENFVSL	9		32.5	Dedicator of cytokinesis protein 2	Q8C3J5
TALKNSTMV	9		32.5	Synembryn-B	Q80XE1; Q80XE1-1
VSPSNEQVL	9		32.8	Amphoterin-induced protein 1	Q80ZD8
HSPTNPGFM	9	1xOxidation [M9]	32.8	Cleavage stimulation factor subunit 1	Q99LC2
SGLVNQSNM	9		32.9	39S ribosomal protein L45, mitochondrial	Q9D0Q7
GSVSNIYKL	9		32.9	cytoplasmic FMR1-interacting protein 1	Q7TMB8-2; Q7TMB8-1
HSVQNTEYF	9		33.1	Bloom syndrome protein homolog	O88700
AGHRNREVL	9		33.2	Tudor domain-containing protein 3	Q91W18-1; Q91W18-3; Q91W18-2
TALWNPVSL	9		33.2	Genetic suppressor element 1	Q3U3C9-4; Q3U3C9-3; Q3U3C9-2; Q3U3C9

VSNLNRQFL	9		33.4	SUMO-activating enzyme subunit 2	Q8C878; Q8C878-2; Q9Z1F9
NSPVNQPAM	9		33.4	Histone acetyltransferase p300	B2RWS6
KQILNIYDL	9		33.5	Vacuolar protein sorting-associated protein 11 homolog	Q91W86
SSIINFGALI	10		33.5	Hydrocephalus-inducing protein	Q80W93-1
SSPSNKFFF	9		33.5	Histone-lysine N-methyltransferase SETD2	E9Q5F9-2; E9Q5F9
KSLVNKGTL	9		33.7	Histone H1.1	P43275
AAVRNCDEF	9		33.7	Cytoplasmic aconitate hydratase	P28271
RAQTNYTCV	9		33.8	Receptor-type tyrosine-protein phosphatase C	P06800-5; P06800-6; P06800
AAITNKYQLV	10		33.9	RNA polymerase I-specific transcription initiation factor RRN3	B2RS91
AVVLNATWL	9		33.9	Acyl-CoA desaturase 3	Q99PL7; P13011; Q99PL7-2
FSGHNLEYL	9		34	Glutathione-specific gamma-glutamylcyclotransferase 1	Q8R3J5
IGPKNYEFL	9		34	Sorting nexin-14	Q8BHY8
KSISNTSKL	9		34	protein elys	Q8CJF7
AAAKNGTSL	9		34.1	Signal transducing adapter molecule 2	O88811-2; O88811-1
VAPLNLGMI	9	1xOxidation [M8]	34.3	U5 small nuclear ribonucleoprotein 200 kDa helicase	Q6P4T2
YQYQNIFGPL	10		34.3	Regulator of nonsense transcripts 1	Q9EPU0-1; Q9EPU0-2
AQISNGQQL	9		34.3	Kinesin-like protein KIF23	E9Q5G3
KAIENCEYA	9		34.3	Mothers against decapentaplegic homolog 2	Q62432-2; Q62432
VMLENYSNL	9		34.4	Zinc finger protein 354B	Q9QXT9; E9PW05; P08042-2; Q6P5C7; P08042; Q571J5; Q8BI99; Q61751
AAIEAMNGQYI	11		34.5	Splicing factor 3b subunit 4	Q8QZY9
SSLHNSLLL	9		34.5	Kinesin-like protein KIF1B	Q60575; Q60575-2
NTHENLTYL	9		34.7	epiplakin	Q9QXS1-6; Q9QXS1-13; Q9QXS1-7; Q9QXS1-9; Q9QXS1-5; Q9QXS1-14; Q9QXS1-12; Q9QXS1-3; Q9QXS1-2; Q9QXS1-10; Q8R0W0; Q9QXS1-15; Q9QXS1-11; Q9QXS1-16; Q9QXS1-4; Q9QXS1-1; Q9QXS1-8
AAVSNLVRV	9		34.7	Vinculin	Q64727
YCITNRSHL	9		34.8	Lysine-specific demethylase 2A	P59997-1
SALVHLEVL	9		34.8	SUMO-specific isopeptidase USPL1	Q3ULM6-4; Q3ULM6-1; Q3ULM6-2; Q3ULM6-3
RSIQNNPSI	9		34.8	integrator complex subunit 1	Q6P4S8
SAQQNQTKI	9		35	Shugoshin 2	Q7TSY8
WSPTNLAYF	9		35.4	Leucine-rich repeat and WD repeat-containing protein 1	Q8BUI3
FAHTNIESLV	10		35.5	Protein disulfide-isomerase A3	P27773
SMNSNIWQI	9		35.7	TELO2-interacting protein 1 homolog	Q91V83
YSIIDDATM	9		35.7	Transmembrane protein KIAA1109	A2AAE1-3; A2AAE1-4; A2AAE1-6; A2AAE1; A2AAE1-2
SAINSIVYI	9		35.7	Major facilitator superfamily domain-containing protein 1	Q9DC37
FMMTNKLDTA	11		35.8	Transmembrane protein 33	Q9CR67
VAPLNADDQT	11		35.8	Xenotropic and polytropic retrovirus receptor 1	Q9Z0U0-2; Q9Z0U0
KSPLNKTEV	9		36.1	Peroxisomal acyl-coenzyme A oxidase 1	Q9R0H0; Q9R0H0-2
SACLNPTVL	9		36.2	2'-deoxynucleoside 5'-phosphate N-hydrolase 1	Q80VJ3
SQTSNSQFI	9		36.2	Protein SMG7	Q5RJH6-3; Q5RJH6; Q5RJH6-2
SALRNSAFESI	11		36.2	U5 small nuclear ribonucleoprotein 200 kDa helicase	Q6P4T2
AANINKESI	9		36.3	Aspartate--tRNA ligase, cytoplasmic	Q922B2
FGIRNGSRL	9		36.3	SUMO-activating enzyme subunit 2	Q9Z1F9
FSVGNHGCYI	10		36.4	Fas apoptotic inhibitory molecule 1	Q9WUD8
SSVSNVNLTL	10		36.5	Nuclear receptor coactivator 2	Q61026
ASFSNGPVL	9		36.6	Nuclear fragile X mental retardation-interacting protein 2	Q5F2E7-2; Q5F2E7
KGIHNFESI	9		36.7	Erythroid differentiation-related factor 1	Q6GQV7
SGLLNSQAL	9		36.7	Fatty acid synthase	P19096
SSPTNGMKTM	10		36.7	Beta-mannosidase	Q8K2I4
AAIENIEHLL	10		36.7	Leucine-rich PPR motif-containing protein, mitochondrial	Q6PB66
ISPSNCIYM	9		36.7	DNA-directed RNA polymerase III subunit RPC6	Q921X6

ATIINEEVL	9		36.7	Signal recognition particle 54 kDa protein	P14576-2; P14576
AAPGNKTSYI	10		36.8	Probable 28S rRNA (cytosine-C(5))-methyltransferase	Q8K4F6
FSHKNWVVF	9		36.8	von Willebrand factor A domain-containing protein 8	Q8CC88
KSIKNIQKI	9		37	ATP synthase subunit gamma, mitochondrial	Q91VR2
QSPLNPCVI	9		37	Phosphatidylinositol 4,5-bisphosphate 3-kinase catalytic subunit beta isoform	Q8BTI9
VAASNIVQM	9		37.1	Proteasome subunit beta type-2	Q9R1P3
SSCQNQDVL	9		37.1	Sacsin	Q9JLC8-3; Q9JLC8; Q9JLC8-2
FMATNPEHL	9		37.2	ER degradation-enhancing alpha-mannosidase-like protein 3	Q2HXL6
SGPINFTVF	9		37.2	Myosin regulatory light chain 2, skeletal muscle isoform	P97457
KAIVNVIGM	9		37.3	splicing factor 3B subunit 1	Q99NB9
AAGINVEII	9		37.3	Inverted formin-2	Q0GNC1; Q0GNC1-3
SSLVNKEDV	9		37.5	Nodal modulator 1	Q6GQT9
SGIENVNSV	9		37.5	Nuclear receptor subfamily 1 group D member 2	Q60674
VTLMNGALM	9	1xOxidation [M9]	37.5	Sodium- and chloride-dependent taurine transporter	O35316
CSLQNKLVI	9		37.7	ZW10 interactor	Q9CQU5
YGLTNNSKL	9		37.8	cell division cycle protein 16 homolog	Q8R349
TQVTNQHLL	9		37.8	Neuronal PAS domain-containing protein 2	P97460
RTNVNFQYI	9		37.9	Glycerol-3-phosphate acyltransferase 3	Q8C0N2
FAYEGRDYI	9		38	H-2 class I histocompatibility antigen, D-B alpha chain	P14431; P14429; P14430; P01899
TSLINSADVL	10		38.2	DNA-directed RNA polymerase III subunit RPC7	Q6NXY9
KAIHNKVNI	9		38.4	Septin-2	P42208
SAVENKQQIGI	13		38.4	Serine/threonine-protein kinase PLK2	P53351
RAIHNIFRM	9		38.5	DCC-interacting protein 13-alpha	Q8K3H0; Q8K3G9
NTLNNIDVI	9		38.5	Cytosolic phospholipase A2	P47713
SSPTSTEYI	9		38.7	Glucocorticoid modulatory element-binding protein 2	P58929
YSLSLQHTL	9		38.8	CASP8 and FADD-like apoptosis regulator OS=Mus musculus OX=10090	O35732; O35732
SAPRDAYWI	9		38.9	Cell division cycle protein 123 homolog	Q8CII2-1; Q8CII2-2
YAIKNIHGV	9		38.9	Isoform 2 of Dynamin-2	P39054-1; P39054-2
STYRNAYDI	9		39	Protein mono-ADP-ribosyltransferase PARP12	Q8BZ20
SAGLNGEIL	9		39	U3 small nucleolar RNA-associated protein 4 homolog	Q8R2N2
YLMKNADYF	9		39.1	OTU domain-containing protein 5	Q3U2S4-2; Q3U2S4
SSLLNNKHFL	10		39.1	Plexin-D1	Q3UH93
RALINKHTF	9		39.3	Putative Polycomb group protein ASXL3	Q8C4A5-2; Q8C4A5; Q8BZ32
FMYKNLQCL	9		39.4	ATP-dependent RNA helicase DDX18	Q8K363
RAVDNQVYV	9		39.5	Omega-amidase NIT2	Q9JHW2
SQIKNEINI	9		39.5	ER membrane protein complex subunit 1	Q8C7X2-2; Q8C7X2
SALENAENHV	10		39.8	uncharacterized protein C17orf53 homolog	Q32P12; Q32P12-2
IQLMNTAHL	9		40	peroxisomal carnitine O-octanoyltransferase	Q9DC50
AAPSSPTTM	9		40.1	zinc finger BED domain-containing protein 3	Q9D0L1
QQPSNYGPM	9		40.1	heterogeneous nuclear ribonucleoproteins A2/B1	O88569-3; O88569; O88569-2
KAVKNFTDV	9		40.2	Catalase	P24270
FGIHNGVETL	10		40.3	Glycerophosphocholine phosphodiesterase GPCPD1	Q8C0L9-1
KAQQNHAFL	9		40.3	DNA-directed RNA polymerase III subunit RPC5	Q9CZT4-2; Q9CZT4
SSCMNMHSI	9		40.4	anaphase-promoting complex subunit 1	P53995
TSYINTSLI	9		40.4	E3 SUMO-protein ligase PIAS1	O88907
VGVWNVPYI	9		40.5	Procollagen-lysine,2-oxoglutarate 5-dioxygenase 3	Q9R0E1; Q9R0E2
AALRLLTYL	9		40.8	Treslin	Q8BQ33
ATVSNGPFL	9		40.9	E3 ubiquitin-protein ligase MSL2	Q69ZF8
RSLENRVTF	9		41	von Willebrand factor A domain-containing protein 5A	Q99KC8
TMAYNITPL	9		41	cold shock domain-containing protein E1	Q91W50
RTIRNQNTV	9		41.1	Protein MCM10 homolog	Q0VBD2
SSIEHLTTL	9		41.2	E3 ubiquitin-protein ligase HUWE1	Q7TMY8-4; Q7TMY8-3; Q7TMY8; Q7TMY8-2

ALLQNKDVI	9		41.2	Alpha-galactosidase A	P51569
RSIGNKNTI	9		41.3	hippocampus abundant transcript 1 protein	P70187
FAPVNVTTEVI	14		41.3	Elongation factor 1-alpha 1	P10126
SSGTNGVSM	9		41.3	Ran-binding protein 3	Q9CT10
KQLVNKEHL	9		41.6	Telomere-associated protein RIF1	Q6PR54-2; Q6PR54-3; Q6PR54-1
GQLANFQEM	9		41.6	AMP deaminase 2	Q9DBT5
VQLENQMVL	9	1xOxidation [M7]	41.7	copper chaperone for superoxide dismutase	Q9WU84
FSELNSTEM	9		41.7	Coiled-coil and C2 domain-containing protein 1B	Q8BRN9
FQLENVNKL	9		41.7	Golgi membrane protein 1	Q91XA2
VGVNNPVFL	9		41.9	Lon protease homolog 2, peroxisomal	Q9DBN5-3; Q9DBN5; Q9DBN5-2
YSIGNGSVF	9		42.1	Malate dehydrogenase, cytoplasmic	P14152
RAIEDADWI	9		42.2	LYR motif-containing protein 9	Q3UN90
AATFNPELI	9		42.3	Peroxisomal acyl-coenzyme A oxidase 1	Q9R0H0; Q9R0H0-2
AGPENIPEPM	10		42.4	Trafficking protein particle complex subunit 12	Q8K2L8
SAGRNALHL	9		42.4	Uveal autoantigen with coiled-coil domains and ankyrin repeats	Q8CGB3-3; Q8CGB3
GAICNSWKL	9		42.5	Mediator of RNA polymerase II transcription subunit 23	Q80YQ2-2; Q80YQ2
SQNFNEEFL	9		42.6	Dedicator of cytokinesis protein 7	Q8R1A4-2; Q8R1A4
QAIKNGQAL	9		42.8	Microtubule-actin cross-linking factor 1	Q9QXZ0-3; Q9QXZ0-2; Q9QXZ0-4; Q9QXZ0
RMPMNKNEL	9		42.9	Beta-1,4-glucuronyltransferase 1	Q8BWP8-2; Q8BWP8
TGVVNGESL	9		43.3	Protein HIRA	Q61666; Q61666-3; Q61666-2
VQPINQHVL	9		43.3	Golgin-45	Q8R2X8; Q8R2X8-2
FAEANTEAI	9		43.3	CCR4-NOT transcription complex subunit 1	Q6ZQ08-2; Q6ZQ08-4; Q6ZQ08; Q6ZQ08-3
NSPRNLAMEA	13		43.3	Eukaryotic translation initiation factor 3 subunit D	O70194
LATANGTHI	9		43.4	Zinc finger protein 76	Q8BMU0-1
GAVHNIESL	9		43.5	Mitochondrial chaperone BCS1	Q9CZP5
FSEENHEPL	9		43.5	Vacuolar protein sorting-associated protein 35	Q9EQH3
YGPENTLPTL	10		43.5	G2/M phase-specific E3 ubiquitin-protein ligase	Q5RJY2
YGVSNLQEF	9		43.6	Adiponectin receptor protein 1	Q91VH1
VAPYNTTQFL	10		43.6	Protein HEXIM1	Q8R409
FALANHLIKV	10		43.8	EH domain-containing protein 1	Q9QXY6; Q9WVK4
AQVQNSEQL	9		43.8	Rab GTPase-binding effector protein 2	Q91WG2-2; Q91WG2; Q91WG2-1
SAVTNSGVHL	10		44.1	GTP-binding protein 8	Q9CY28-2; Q9CY28-1
AAISNKITSCI	11		44.5	multifunctional protein ADE2	Q9DCL9
TAVANNQAL	9		44.8	Calcitonin gene-related peptide type 1 receptor	Q9R1W5
RVPINETFI	9		44.8	WASH complex subunit 5	Q8C2E7
SGLLNQQSL	9		45.3	Mediator of RNA polymerase II transcription subunit 27	Q9DB40-2; Q9DB40-1
VAIGNPVHL	9		45.3	Zinc finger homeobox protein 3	Q61329
TAVISYDYL	9		45.3	Uncharacterized aarF domain-containing protein kinase 1	Q9D0L4
FGGHNEDFI	9		45.9	WD repeat-containing protein 26	Q8C6G8
DALSNVDLL	9		46	cytoplasmic FMR1-interacting protein 1	Q7TMB8-2; Q7TMB8-1; Q5SQX6
STMVNADHF	9		46.1	Phospholipid scramblase 4	P58196
CTIVNIHEL	9		46.2	Constitutive coactivator of peroxisome proliferator-activated receptor gamma	Q6RI63-1
GAVVNVADL	9		46.2	Tankyrase-2	Q3UES3
TQGRNSTPL	9		46.2	Tankyrase-1	Q6PFX9-1; Q6PFX9-2
SAIINPPQACI	11		46.2	›oyllysine-residue acetyltransferase component of pyruvate dehydrogenase complex, mito	Q8BMF4
YGIVSGDLI	9		46.3	F-box only protein 7	Q3U7U3
SSLSNPIANTM	11		46.4	F-box only protein 33	Q8VE08
NSLNNLIEL	9		46.4	E3 ubiquitin-protein ligase HERC2	Q4U2R1-2; Q4U2R1
SAVENILEHL	10		46.7	28S ribosomal protein S6, mitochondrial	P58064
QSFTNPTLI	9		46.8	Peroxisomal membrane protein pex13	Q9D0K1
SSFKNLVSL	9		46.8	Toll-like receptor 9	Q9EQU3
SGANNVVFI	9		46.9	THUMP domain-containing protein 1	Q99J36

GTHVNVTCI	9	47.3	Tumor necrosis factor receptor superfamily member 1B	P25119
KAVENSSTAI	10	47.5	Proteasome subunit alpha type-3	O70435
SSVSNPRNWL	10	47.5	Tumor necrosis factor receptor superfamily member 23	Q9ER63
SSYQNSSYGL	10	47.5	Nuclear receptor coactivator 3	O09000
FGWGNSEYL	9	47.6	RCC1-like G exchanging factor-like protein	Q9CYF5
FSPTNPAHLL	10	47.7	WD repeat-containing protein 76	A6PWY4-1; A6PWY4-3; A6PWY4-2
TSFVNFTDI	9	47.8	Eukaryotic translation initiation factor 2 subunit 2	Q99L45
VALYNQGHI	9	47.9	Synaptic vesicle membrane protein VAT-1 homolog	Q62465
SLLTNHVTL	9	47.9	Homeodomain-interacting protein kinase 1	O88904-1; O88904-2
LSAKNKDYM	9	48.1	bifunctional UDP-N-acetylglucosamine 2-epimerase/N-acetylmannosamine kinase	Q91WG8
AALVDLDSL	9	48.2	Epsin-1	Q80VP1-2; Q80VP1
SSPSNGAFGE	14	48.2	T-box transcription factor TBX15	O70306
HAPCNVPPYL	10	48.3	Poly(A) RNA polymerase GLD2	Q91YI6-1
SAVSNNYIQTL	11	48.3	Catenin delta-1	P30999-2; P30999-1; P30999-3
GSLRNVLSL	9	48.3	Peroxisomal membrane protein PMP34	O70579
YGINNIREL	9	48.5	Phenylalanine--tRNA ligase alpha subunit	Q8C0C7
TGPPNTSYM	9	48.5	Tumor susceptibility gene 101 protein	Q61187
TTLRNGEVL	9	48.6	transmembrane protein 135	Q9CYV5
GAVTNVKVI	9	48.6	ELAV-like protein 3	Q60900; P70372; Q60900-2; Q60899
FMKQNLDEL	9	48.7	5'-AMP-activated protein kinase subunit gamma-2	Q91WG5-2; Q91WG5
YIHSNQNVI	9	48.8	DNA excision repair protein ERCC-6-like 2	Q9JIM3
KAFSNKQTV	9	49	Exportin-T	Q9CRT8
IAMSNSMINTI	11	49.1	Prokineticin receptor 1	Q9JKL1; Q8K458
RSPENVIETI	10	49.2	eIF-2-alpha kinase activator GCN1	E9PVA8
GSHTNIHLL	9	49.2	Activity-dependent neuroprotector homeobox protein	Q9Z103
FMIGNGENL	9	49.6	Sterol-4-alpha-carboxylate 3-dehydrogenase, decarboxylating	Q9R1J0
ISPNNRNAI	9	49.8	LIM domain kinase 2	O54785-1; O54785-2; O54785-3
YQIAMVHYI	9	49.8	Inosine-5'-monophosphate dehydrogenase 1	P50096
GQVINLDQL	9	49.8	Protein FAM91A1	Q3UVG3
SGVENTELV	9	49.8	DNA-directed RNA polymerase III subunit RPC5	Q9CZT4-2; Q9CZT4
GAPSNPDVF	9	50.1	Ribosyldihydronicotinamide dehydrogenase [quinone]	Q9JI75
KAPTNEFYA	9	50.2	syndecan-4	O35988
LALVNSSNV	9	50.2	AP-1 complex subunit gamma-like 2	O88512
FSLQNQLRL	9	50.3	protein flightless-1 homolog	Q9JJ28
RAIPNNQVL	9	50.4	Endothelial differentiation-related factor 1	Q9JMG1
YGGYNIEEI	9	50.5	NAD(P) transhydrogenase, mitochondrial	Q61941
SAIMDSDLL	9	50.6	Histone-lysine N-methyltransferase SETD1A	E9PYH6
RGPSNSTSL	9	50.7	Chloride channel protein 2	Q9R0A1
SSIQNPKVSL	10	50.7	Ubinuclein-2	Q80WC1-4; Q80WC1-2; Q80WC1; Q80WC1-3
LAPLNQAFF	9	50.8	Monocarboxylate transporter 1	P53986
SALQNFASTF	10	51.1	Brefeldin A-inhibited guanine nucleotide-exchange protein 3	Q3UGY8
TALRNLPEYTL	11	51.2	integrator complex subunit 1	Q6P4S8
SAPANPSSNI	10	51.5	SLAIN motif-containing protein 2	Q8CI08-2; Q8CI08
VSVENGMII	9	51.7	Proteasome inhibitor PI31 subunit	Q8BHL8
VGVENVAEL	9	51.7	Glycogen phosphorylase, liver form	Q9ET01
SCPVNIASI	9	51.9	Thioredoxin domain-containing protein 16	Q7TN22; Q7TN22-2
FNLVNNALL	9	52.2	Apoptosis inhibitor 5	O35841
AQPVNVQQL	9	52.2	E1A-binding protein p400	Q8CHI8-4; Q8CHI8-3; Q8CHI8-2; Q8CHI8-5; Q8CHI8
KAPDNRETL	9	52.7	Dolichyl-diphosphooligosaccharide--protein glycosyltransferase subunit STT3B	Q3TDQ1
GAIRNGGLYV	10	52.7	Uncharacterized aarF domain-containing protein kinase 5	Q80V03-2; Q80V03
SAIMNPASKVL	13	52.8	Clathrin heavy chain 1	Q68FD5
FAYTSRHEI	9	52.8	Dehydrodolichyl diphosphate synthase complex subunit Dhdds	Q99KU1; Q99KU1-2

FSNVNLKEV	9		52.9	DNA damage-binding protein 1	Q3U1J4
VQIHNTENI	9		52.9	Ankyrin repeat and SOCS box protein 6	Q91ZU1
VAAINPELL	9		52.9	Kinesin-like protein KIF2C	Q922S8
MAGINTDHL	9		53.1	Piezo-type mechanosensitive ion channel component 1	E2JF22
ASVQNEAKL	9		53.1	coronin-1C	Q9WUM4
NMHNNLDEL	9		53.2	Sodium bicarbonate cotransporter 3	Q8BTY2; Q8BTY2-2
SNLSNYHQYI	10		53.3	Phosphatidylinositol 5-phosphate 4-kinase type-2 gamma	Q91XU3
VCIENTETL	9		53.4	Kinesin-like protein KIF20A	P97329
YLHENASYV	9		53.5	Protein phosphatase 1 regulatory subunit 21	Q3TDD9
SGGQNITIM	9	1xOxidation [M9]	53.7	Plexin-C1	Q9QZC2
YAGSNFPEHI	10		53.8	Actin-related protein 2	P61161
STIQNADLIVVI	12		53.8	Phosphatidylcholine translocator ABCB4	P21440; P06795; P21447
SSIQGLATI	9		53.8	Multidrug resistance-associated protein 5	Q9R1X5
YSIQGQSVM	9		53.8	Protein MON2 homolog	Q80TL7; Q80TL7-2
FSITDQQTL	9		54.3	Protein NLRC5	C3VPR6
RGPVNLQHL	9		54.5	Leucine-rich repeat and fibronectin type-III domain-containing protein 4	Q80XU8
SALEDLEVL	9		54.6	Mediator of RNA polymerase II transcription subunit 4	Q9CQA5; Q9CQA5-2
HAPRNLRLTYI	11		54.7	sialoadhesin	Q62230-3; Q62230
STVNNTVTV	9		54.7	Dedicator of cytokinesis protein 10	Q8BZN6-3; Q8BZN6-4; Q8BZN6; Q8BZN6-2
SLITNKVVM	9		54.8	Purine nucleoside phosphorylase	P23492
VAIENPADVSV	12		55	40S ribosomal protein SA	P14206
HSVQNGTVRM	10		55	Rho GTPase-activating protein 24	Q8C4V1-3; Q8C4V1; Q8C4V1-2
SQGMNVTNM	9	1xOxidation [M9]	55.4	Histone acetyltransferase p300	B2RWS6
VQVNNVVVL	9		55.5	Histone chaperone ASF1A	Q9CQE6
FAFVNEEKF	9		55.6	Myotubularin	Q9Z2C5
SVVLNTSTL	9		55.7	E3 ubiquitin-protein ligase TTC3	O88196-2; O88196-4; O88196-6; O88196-1; O88196-3; O88196-5
AGLLNNPHFI	10		55.9	Small glutamine-rich tetratricopeptide repeat-containing protein alpha	Q8BJU0-1; Q8BJU0-2
RSVPNVEEL	9		56.3	Beta/gamma crystallin domain-containing protein 3	Q80W49; Q80W49-2
VTIHNQDLL	9		56.4	Zinc finger protein ZXDC	Q8C8V1-3; Q8C8V1-4; Q8C8V1-1; Q8C8V1-2
SAPSNFEHRV	10		56.5	serine/threonine-protein kinase PAK 4	Q8BTW9
RAAYNVTLL	9		56.6	alpha-ketoglutarate-dependent dioxygenase FTO	Q8BGW1
HGPTNIMVYI	10		57.1	Magnesium transporter NIPA1	Q8BHK1
SALQNAESDR	11		57.1	Centromere-associated protein E	Q6RT24
SGIKNGNFAL	10		57.2	Protocadherin Fat 3	Q8BNA6; Q8BNA6-2
ASIVNKDGL	9		57.2	Peroxisome proliferator-activated receptor delta	P35396
FAPVNVTTEVI	11		57.3	Elongation factor 1-alpha 1	P10126
YVFFNGDHM	9		57.4	E3 ubiquitin-protein ligase RNF213	E9Q555
TSTTNPEFV	9		57.5	Isoform 4 of High mobility group protein 20A	Q9DC33-4; Q9DC33-3; Q9DC33-2; Q9DC33-1
RALLLSTYI	9		57.5	AP-2 complex subunit alpha-2	P17426-2; P17427; P17426
YGYSNRVVDL	10		57.5	Glyceraldehyde-3-phosphate dehydrogenase	Q64467; P16858
AKLVNQEVL	9		57.6	NADH-ubiquinone oxidoreductase 75 kDa subunit, mitochondrial	Q91VD9
SAETNLEAL	9		57.6	Dual specificity mitogen-activated protein kinase kinase 1	P31938
RIIANVEEI	9		57.8	ATP-binding cassette sub-family D member 2	Q61285
KQAQNIVTL	9		57.8	Citramalyl-CoA lyase, mitochondrial	Q8R4N0
VSYKNPSLM	9		58	FACT complex subunit SPT16	Q920B9
AVVNVTYM	8	1xOxidation [M8]	58	Insulin-like growth factor 2 mRNA-binding protein 2	Q5SF07-2; Q5SF07
QSITNLSNL	9		58.4	Protein strawberry notch homolog 1	Q689Z5; Q689Z5-2
HSLQDWDTI	9		58.5	camp-dependent protein kinase catalytic subunit prkx	Q922R0
ANYAGQFNQD	12		58.6	Annexin A11	P97384
TATMNLDRI	9		58.7	Inositol 1,4,5-trisphosphate receptor type 3	P70227
AGIYNLDDL	9		58.7	General transcription and DNA repair factor IIH helicase subunit XPD	O08811

SAVGNDTQL	9		58.9	Telomerase-binding protein EST1A	P61406
QAPQNKITV	9		59.1	L-lactate dehydrogenase A chain	P06151
SNLVNENTL	9		59.3	ATP-dependent RNA helicase DDX18	Q8K363
AAHTNRKEYT	11		59.3	Claudin domain-containing protein 1	Q9CQX5
QAINNAINYL	10		59.4	DNA-binding protein Ikaros	Q03267-2; Q03267-4; Q03267-3; Q03267-6; Q03267-5; Q03267
SQVYNDAHI	9		59.5	Protein polybromo-1	Q8BSQ9; Q8BSQ9-2
MAIKNPKATL	10		59.8	Hypoxia up-regulated protein 1	Q9JKR6
LSLENGTHTL	10		60.4	DNA (cytosine-5)-methyltransferase 1	P13864
YSIRNPPQPLI	11		60.4	focadhesin	A2AKG8-2; A2AKG8
VALRNDSYT	9		60.5	26S proteasome regulatory subunit 8	P62196
FSKVNIQVL	9		61.1	2'-5'-oligoadenylate synthase-like protein 2	Q9Z2F2
AQLENAAKV	9		61.2	Exportin-4	Q9ESJ0
ASARNLEHL	9		61.3	F-box only protein 38	Q8BMI0
ASTTNQQEI	9		61.3	/SNF-related matrix-associated actin-dependent regulator of chromatin subfamily D memb	Q99JR8-1; Q99JR8-2
SAVQNLVVEG	11		61.4	AMSH-like protease	Q76N33; Q76N33-2
SSPENLKGFI	10		61.7	Testis-expressed protein 10	Q3URQ0
RSLLNKQETL	10		62.1	IQ calmodulin-binding motif-containing protein 1	Q8BP00-2; Q8BP00-1
KAVANGKVL	9		62.2	Cyclin-H	Q61458
KALEDVDYV	9		62.2	Serine/threonine-protein phosphatase 4 regulatory subunit 3A	Q6P2K6-2; Q6P2K6-1
DSLVNQEVL	9		62.3	DNA-directed RNA polymerase I subunit RPA2	P70700
IQAENAEFM	9		62.6	heme oxygenase 1	P14901
AAVVSVAYL	9		62.7	Natural resistance-associated macrophage protein 2	P49282-4; P49282-3; P49282-2; P49282
SAIQNGSSGG	13		62.9	Forkhead box protein P1	P58462; P58462-2; P58462-5
AAVQNPALTA	11		63.1	Sterol regulatory element-binding protein 2	Q3U1N2-1; Q3U1N2-2
YLMGNLEEI	9		63.8	Rho guanine nucleotide exchange factor 7	Q9ES28; Q9ES28-7; Q9ES28-5; Q9ES28-3; Q9ES28-4; Q9ES28-6; Q9ES28-8; Q9ES28-2
TQPVNLFNL	9		63.9	Pericentriolar material 1 protein	Q9R0L6; Q9R0L6-2
KGLANHVVV	9		63.9	Rho family-interacting cell polarization regulator 1	Q68FE6
KSLINKNEL	9		64.1	Inositol polyphosphate 5-phosphatase OCRL-1	Q6NVF0-2; Q6NVF0
FLVQNIHTL	9		64.2	Ribosome biogenesis protein BRX1 homolog	Q9DCA5
SGIKNPVSV	9		64.8	Threonine aspartase 1	Q8R1G1; Q8R1G1-2
SGGSNIIYV	9		64.8	Vam6/Vps39-like protein	Q8R5L3-2; Q8R5L3
SSLKNFQSCI	10		65	E3 SUMO-protein ligase NSE2	Q91VT1; Q91VT1-2
FAPQHYTEL	9		65.2	Chromosome transmission fidelity protein 18 homolog	Q8BIW9
YAAQATTPM	9		65.5	RNA-binding protein 14	Q8C2Q3; Q8C2Q3-2
SNPRNWLFL	9		65.6	Tumor necrosis factor receptor superfamily member 23	Q9ER63
YQYTSPDFL	9		65.8	Insulin-induced gene 1 protein	Q8BGI3; Q91WG1
VSLLNPPETL	10		66	Cyclin-A2	P51943
YQHKNLLIL	9		66	Transportin-1	Q8BFY9; Q8BFY9-2; Q99LG2
VTVLNVDHL	9		66.3	CASP8-associated protein 2	Q9WUF3
RMVLNFGTEM	10		66.7	Cap-specific mRNA (nucleoside-2'-O-)-methyltransferase 2	Q8BWQ4
SALENGRYELI	11		66.7	Bromodomain adjacent to zinc finger domain protein 1A	O88379
VSQINAEFV	9		67.1	RNA helicase aquarius	Q8CFQ3
SSIVNKEGL	9		67.4	Tropomodulin-1	P49813
GGPENVAEM	9	1xOxidation [M9]	67.4	protein strawberry notch homolog 1	Q689Z5-1; Q689Z5-2
NQGTNVETI	9		67.6	C2 domain-containing protein 3	Q52KB6-2; Q52KB6-3; Q52KB6
SGHSNHIAL	9		68	N-acetylglucosaminyl-phosphatidylinositol de-N-acetylase	Q5SX19
SRILNQNYI	9		68.1	Galactocerebrosidase	P54818
SSMRGMDTV	9		68.1	Arginine/serine-rich coiled-coil protein 2	A2RTL5
SLVTNMDKL	9		68.3	Nck-associated protein 1-like	Q8K1X4
ATLENVTNL	9		68.5	CXXC motif containing zinc binding protein	Q8BHG2-2; Q8BHG2; Q8BHG2-3

SAVRNGLLLL	10		69	Cullin-9	Q80TT8-1; Q80TT8-4
FQVKNPPHTY	11		69	Formin-like protein 3	A2APV2-3; Q6ZPF4-2; Q6ZPF4-1; A2APV2-1; A2APV2-2
SHYADVDPEN	14		69	Sodium-coupled neutral amino acid transporter 2	Q8CFE6
KQIKNSSLL	9		69.1	uncharacterized protein KIAA1143 homolog	Q8K039
SALRNCFSHL	10		69.3	mannose-6-phosphate isomerase	Q924M7
ISPENHISL	9		69.4	Interferon-inducible double-stranded RNA-dependent protein kinase activator A	Q9WTX2
ATISNDGATI	10		69.4	T-complex protein 1 subunit eta	P80313
GSPANTRYL	9		69.5	Serine/threonine-protein phosphatase 2B catalytic subunit beta isoform	P48453-2; P63328; P63328-2; P48453
RTISNPEVV	9		69.6	Afadin	Q9QZQ1; Q9QZQ1-2
FMALNKEHL	9		69.7	caspase recruitment domain-containing protein 9	A2AIV8
FCYVNDIVI	9		69.7	Histone deacetylase 3	O88895-1; O09106; P70288
HALLNDAWVL	10		69.9	Lethal(2) giant larvae protein homolog 2	Q3TJ91
SIISNTEAV	9		70	Heat shock protein 75 kDa, mitochondrial	Q9CQN1
SGIVNMDTP	9		70.1	Ankyrin repeat domain-containing protein 17	Q99NH0
RNISNQLVI	9		70.2	Checkpoint protein HUS1	Q8BQY8-3; Q8BQY8; Q8BQY8-2
AMGVNMETA	9		70.9	V-type proton ATPase subunit B, brain isoform	P62814
SVIRNPEIL	9		70.9	eIF-2-alpha kinase activator GCN1	E9PVA8
YQGQNAWFL	9		70.9	4F2 cell-surface antigen heavy chain	P10852; P10852-2
SGLSNGTGST	11		71	Isoform 4 of CUGBP Elav-like family member 1	P28659-2; P28659; P28659-3; P28659-4
SVATNIDEI	9		71.2	Structural maintenance of chromosomes protein 1A	Q9CU62
VGLNNINAL	9		71.3	CUGBP Elav-like family member 2	Q9Z0H4; Q9Z0H4-7; Q9Z0H4-11; Q9Z0H4-9; Q9Z0H4-4; Q9Z0H4-8; Q9Z0H4-6; Q9Z0H4-2; Q9Z0H4-3; Q9Z0H4-5
ISLANLDTNKL	11		71.9	Putative ataxin-7-like protein 3B	Q3UD01
SASANLPVM	9		71.9	Forkhead box protein O3	Q9WVH4
HSIQNSQDM	9	1xOxidation [M9]	72.3	BAG family molecular chaperone regulator 2	Q91YN9
AGQFNQDYL	9		72.4	Annexin A11	P97384
WAQLNRDLI	9		73	Derlin-1	Q99J56
ASVLNKWQM	9		73.1	ATP-dependent RNA helicase DDX1	Q91VR5
NMHQNIKYI	9		73.2	Glycoprotein endo-alpha-1,2-mannosidase	Q6NXH2
VTITNDGATI	10		73.4	T-complex protein 1 subunit alpha	P80315; P11983-2; P80316; P11983
HGVLNTDNM	9		73.5	Selenoprotein O	Q9DBC0
ASLTNNQNL	9		73.8	Nucleoprotein TPR	F6ZDS4
AALSNPGAKM	10	1xOxidation [M10]	73.9	Centrosomal protein kizuna	Q3UXL4
GKLLNAEYI	9		74.1	Actin-binding LIM protein 2	Q8BL65-4; Q8BL65-5; Q8BL65-3; Q8BL65-2; Q8BL65
SQWNNDNPL	9		74.2	Integrin beta-2	P11835
RSPENKFPVI	10		74.3	Nuclear factor interleukin-3-regulated protein	O08750
TSVVDGTPL	9		74.3	Protein O-GlcNAcase	Q9EQQ9; Q9EQQ9-3
FAFSNGYLASI	11		74.5	Equilibrative nucleoside transporter 1	Q9JIM1; Q9JIM1-2
NGIRNIDLHYI	11		74.9	E3 ubiquitin-protein ligase MARCH6	Q6ZQ89; Q6ZQ89-3
AAVANQHSSF	11		75	Kinesin-like protein KIF11	Q6P9P6
SSFVDHDFL	9		75.4	Histone-lysine N-methyltransferase ASH1L	Q99MY8
GQLENAEQL	9		75.8	Tetratricopeptide repeat protein 19, mitochondrial	Q8CC21
KAVVNEKVL	9		76.4	Protein MON2 homolog	Q80TL7-1; Q80TL7-2
SSTTNIDDL	9		76.5	Cytoskeleton-associated protein 5	A2AGT5; A2AGT5-3; A2AGT5-2
KSPTNQQPTL	10		76.5	Cytosolic carboxypeptidase-like protein 5	Q09M02-2; Q09M02-5; Q09M02-7; Q09M02-3; Q09M02; Q09M02-4; Q09M02-6
GAPLNIHKV	9		76.5	TBC1 domain family member 5	Q80XQ2
HILANADAI	9		76.6	Tumor necrosis factor alpha-induced protein 2	Q61333
IMGQNVADYM	10		76.9	60S ribosomal protein L5	P47962
NTPENREYL	9		77	Actin-related protein 3B	Q641P0; Q641P0-2
STLMNHERI	9		77.1	Zinc finger protein 250	Q7TNU6
YSTNNPSEL	9		77.4	COP9 signalosome complex subunit 3	O88543

TSPINPQHM	9	77.5	Trinucleotide repeat-containing gene 6C protein	Q3UHC0
RSITNDSAVL	10	77.7	Hepatocyte growth factor-regulated tyrosine kinase substrate	Q99LI8
AAIANAFRRI	10	77.7	DNA-directed RNA polymerases I and III subunit RPAC1	P52432
GALENAKAEI	10	78.4	Centrosomal protein POC5	Q9DBS8
TMKTNLEYL	9	78.5	Structural maintenance of chromosomes protein 6	Q924W5; Q924W5-2
RQATNQIVM	9	78.9	Puromycin-sensitive aminopeptidase	Q11011
VGPKNKTSI	9	79.2	Ubiquitin carboxyl-terminal hydrolase 10	P52479; P52479-2
VSILNYQSAL	10	79.4	Homeodomain-interacting protein kinase 2	Q9QZR5
TAISNYMNQL	10	79.5	DCC-interacting protein 13-alpha	Q8K3H0
VGLINKDSV	9	79.7	Histone-lysine N-methyltransferase ASH1L	Q99MY8
FAHFNSHAA	9	80.3	Arf-GAP domain and FG repeat-containing protein 1	Q8K2K6-4; Q8K2K6-3; Q8K2K6-1; Q8K2K6-2
GGNINVETI	9	80.5	Dolichyl-diphosphooligosaccharide--protein glycosyltransferase 48 kDa subunit	O54734
IAVANAQELL	10	80.6	mitochondrial import inner membrane translocase subunit TIM13	P62075
SALTNGGLNL	10	80.6	Chromodomain Y-like protein 2	Q9D5D8
SSVLNQEEAY	11	80.7	Interleukin-7 receptor subunit alpha	P16872
SHLTNKYVL	9	80.9	transmembrane protein 241	Q3UME2-3; Q3UME2-2; Q3UME2-4; Q3UME2
TSTTNFTKI	9	80.9	Transmembrane protein 131-like	Q3U3D7-2; Q3U3D7; Q3U3D7-3
TSPVNPAVF	9	81	transmembrane protein 258	P61166
GMHVNGAPP	10	81	Cleavage stimulation factor subunit 2	Q8BIQ5-2; Q8BIQ5
SSLLDLETL	9	81.1	Proline and serine-rich protein 3	Q7TSA6-2; Q7TSA6
KGVFNVEVV	9	81.2	Dedicator of cytokinesis protein 7	Q8R1A4-2; Q8R1A4
NVLRNTEVM	9	81.8	Protein FAM118B	Q8C569-2; Q8C569
YNISNSSTF	9	81.8	Plexin-A1	P70206
SAPQSAVEL	9	81.9	Clathrin interactor 1	Q99KN9; Q99KN9-2
ISKENGDYL	9	81.9	Phospholipase DDHD2	Q80Y98; Q80Y98-3
YSPESLHYM	9	82.1	Thyroid adenoma-associated protein homolog	A8C756-1
LAVSNHVFHL	10	82.3	Zinc finger MIZ domain-containing protein 1	Q6P1E1-2; Q6P1E1
SAIANIPAAAV	11	82.8	eyes absent homolog 3	P97480; P97480-2
RLITNSEEI	9	83	ATP-binding cassette sub-family D member 3	P55096
TGLENSTAV	9	83.2	Secretion-regulating guanine nucleotide exchange factor	Q80YD6
HTHMNVHYV	9	83.4	male-specific lethal 3 homolog	Q9WVG9-2; Q9WVG9-1
NSIKNGILYL	10	83.9	1-phosphatidylinositol 4,5-bisphosphate phosphodiesterase gamma-1	Q62077
FAIDDHDYL	9	84.2	Phosphatidylinositol 5-phosphate 4-kinase type-2 gamma	Q91XU3
MSLVNKTTHLI	11	84.3	26s proteasome non-atpase regulatory subunit 12	Q9D8W5
STVFNPSVL	9	84.4	muscleblind-like protein 2	Q8C181; Q8C181-2; Q8C181-4
GNVINPDTI	9	84.4	Isoleucine--tRNA ligase, mitochondrial	Q8BIJ6
TGYLNTVTV	9	84.6	Receptor of activated protein C kinase 1	P68040
YCLPNLTHL	9	85	Peroxisome proliferator-activated receptor gamma coactivator-related protein 1	Q6NZN1
TSFKNPFLI	9	85.1	Tripeptidyl-peptidase 1	O89023
TGPSNVDKL	9	85.2	Serine/threonine-protein kinase Chk1	O35280-2; O35280
RALENPDTASI	11	85.2	Tyrosine-protein phosphatase non-receptor type 23	Q6PB44-1; Q6PB44-2
TALEHQEYI	9	85.3	Complement component 1 Q subcomponent-binding protein, mitochondrial	O35658
WAIENPAAF	9	85.6	protein-lysine N-methyltransferase EEF2KMT	Q3UZW7-2; Q3UZW7-3; Q3UZW7
YSASANLPVM	10	86.4	Forkhead box protein O3	Q9WVH4
LSVWNQQVL	9	87.4	Isoform 3 of Mediator of RNA polymerase II transcription subunit 14	A2ABV5-3; A2ABV5-2; A2ABV5-4; A2ABV5
GMKLNLDYL	9	87.8	Developmentally-regulated GTP-binding protein 2	Q9QXB9
SAYSNYAEQT	11	87.8	RNA-binding protein 4	Q8C7Q4
RQTVNVIAM	9	88	tumor susceptibility gene 101 protein	Q61187
SSPVNPVVFF	10	88.6	Peptidyl-prolyl cis-trans isomerase H	Q9D868; Q9D868-2
TAAENAEKI	9	88.7	Isoform 2 of Leucine-rich repeat flightless-interacting protein 2	Q91WK0-2; Q91WK0-1
VTVLNITHL	9	89.2	Activating signal cointegrator 1 complex subunit 3	E9PZJ8-2; E9PZJ8
HAISNYYDNHL	11	89.4	Exonuclease 3'-5' domain-containing protein 2	Q8VEG4-2; Q8VEG4

QGIKNSVTL	9		89.5	Centrosomal protein of 78 kDa	Q6IRU7
QALEDLDYL	9		89.6	Putative pre-mRNA-splicing factor ATP-dependent RNA helicase DHX32	Q8BZS9-2; Q8BZS9
AVNMNLHYV	9		89.7	DNA-directed RNA polymerase I subunit RPA2	P70700
SSIQNFQKGTI	11		90	PX domain-containing protein kinase-like protein	Q8BX57-1
MNVQNVEFI	9		90.1	serine/threonine-protein kinase ATR	Q9JKK8
TAPTNATYP	9		90.4	Ankyrin repeat domain-containing protein 17	Q99NH0
FQYLNRREL	9		90.7	F-box/WD repeat-containing protein 8	Q8BIA4-4; Q8BIA4
ATVINGQFV	9		91.3	Insulin receptor	P15208
KNLVNKEVM	9		91.4	glucose-6-phosphate isomerase	P06745
GAVYNGEPKL	10		91.7	sphingosine-1-phosphate lyase 1	Q8R0X7
SGNFNVDNI	9		91.9	T-complex protein 1 subunit theta	P42932
SCIPNREYV	9		92.1	NAD-dependent protein deacetylase sirtuin-7	Q8BKJ9
SQITSLVTL	9		92.1	Origin recognition complex subunit 5	Q9WUV0
FAPVNVTTE	9		92.2	Elongation factor 1-alpha 1	P10126
LSPRNLVLV	9		92.3	Protein RRNAD1	Q8BZG5
VSPSDRDFM	9		92.6	Kinesin-like protein KIF21B	Q9QXL2-3; Q9QXL2-2; Q9QXL1; Q9QXL2; Q9QXL2-4
KALKLLDYV	9		92.7	Trafficking protein particle complex subunit 11	B2RXC1
KQPLNTTRI	9		93.1	tyrosine-protein phosphatase non-receptor type 11	P35235; P35235-2
KTHINIVVI	9		93.2	Elongation factor 1-alpha 1	P10126; P62631
SAPRNFVENF	14		93.3	Elongator complex protein 2	Q91WG4-2; Q91WG4
TAVVNRVFDKI	11		93.5	Proteasome subunit beta type-9	O35522; P28076
AAHQNPEICL	10		93.5	Twinkle protein, mitochondrial	Q8CIW5; Q8CIW5-2
FAPVNVTTEVI	12		94	Elongation factor 1-alpha 1	P10126
VACVNQFII	9		94.4	transportin-1	Q8BFY9
SALNNKDVNTI	11		94.4	GTPase Era, mitochondrial	Q9CZU4
RALELGTVM	9		94.9	1-phosphatidylinositol 4,5-bisphosphate phosphodiesterase gamma-2	Q8CIH5
ISPFNKSAL	9		95.1	CLIP-associating protein 2	Q8BRT1
YAVRDTIAL	9		95.4	Phospholipid-transporting ATPase 11C	Q9QZW0-1; Q9QZW0-2
YGYSNRVVDL	11		95.4	glyceraldehyde-3-phosphate dehydrogenase	P16858
VVVENITLM	9		95.4	E3 ubiquitin-protein ligase UBR4	A2AN08-2; A2AN08-3; A2AN08-5; A2AN08
FAPYNKPSL	9		95.5	Probable E3 ubiquitin-protein ligase DTX2	Q8R3P2-2; Q8R3P2; Q8R3P2-3
YALIHPATTL	10		96.1	protein Smaug homolog 2	Q80XS6
LGVTNFVHM	9		96.3	FAD-dependent oxidoreductase domain-containing protein 2	Q3USW5
SSSTNVDLL	9		96.4	Protein SAAL1	Q9D2C2-2; Q9D2C2
GTLSNPCTL	9		96.5	F-box only protein 11	Q7TPD1; Q7TPD1-2; Q7TPD1-3
VGPENMQIM	9		96.7	Protein MON2 homolog	Q80TL7-1; Q80TL7-2
YQSMNSQYL	9	1xOxidation [M4]	96.9	Lysosomal protective protein	P16675
SALQNVKRL	9		97.1	Vacuolar protein sorting-associated protein 45	P97390
VGTNNQSYI	9		97.2	WW domain binding protein 1-like	Q8BGW2-4; Q8BGW2-3; Q8BGW2; Q8BGW2-2
SGIIDSDFL	9		97.3	General transcription factor 3C polypeptide 6	Q9D8P7
LSHINRDKL	9		97.3	Histone-lysine N-methyltransferase ASH1L	Q99MY8
SAPVNVGNTA	10		97.3	MICAL-like protein 2	Q3TN34
SSPVNVKKL	9		97.7	Heterogeneous nuclear ribonucleoprotein U	Q8VEK3-2; Q8VEK3
SALDNGPQI	9		97.7	E3 ubiquitin-protein ligase Itchy	Q8C863; Q8C863-2
LSPANGEQI	9		97.7	Inhibitor of growth protein 1	Q9QXV3
NQVKNIAEL	9		98	DNA repair endonuclease XPF	Q9QZD4
SAISNPSPCA	10		98.3	Nucleoporin NDC1	Q8VCB1
DQIQNAQYL	9		98.4	Heterogeneous nuclear ribonucleoprotein K	P61979-3; P61979-2; P61979
SQVVNVLTSVI	11		98.4	Uncharacterized protein C1orf112 homolog	Q3TQQ9-1; Q3TQQ9-2; Q3TQQ9-3
GGVRNGVAL	9		98.6	Nucleolar protein 11	Q8BJW5-2; Q8BJW5
RQFLNRDDI	9		98.7	V-type proton ATPase subunit F	Q9D1K2
ISHINKELL	9		99	maleylacetoacetate isomerase	Q9WVL0

LAFRNKDQL	9		99.6	Mast cell-expressed membrane protein 1	Q9D8U6
RQVQNTAITL	10		99.8	MORC family CW-type zinc finger protein 2B	Q69ZX6; Q8C5W4-2; Q8C5W4-1
VSPRNSLEVL	10		99.8	DDB1- and CUL4-associated factor 10	A2AKB9-3; A2AKB9-2; A2AKB9-1
KGITNHLVAI	10		99.9	Macrophage-expressed gene 1 protein	A1L314
SSIQNGKYTLN	12		100.2	Lysine-specific demethylase 7A	Q3UWM4
SAVVNKVPLS	10		100.2	Transmembrane protein 263	Q9DAM7
SSITNHINKL	10		100.3	Semaphorin-5A	Q62217
RSVGNTIVL	9		100.5	Tetratricopeptide repeat protein 30B	Q9CY00
KAVENYINCL	10		100.8	Serine-protein kinase ATM	Q62388
SSVASTDVL	9		101.2	AP-2 complex subunit alpha-1	P17426-2; P17426
FQGKNLETV	9		101.4	Plexin-B2	B2RXS4
YNVINREKL	9		101.5	protein farnesyltransferase subunit beta	Q8K2I1
SGIRNQGGTC	12		102	Ubiquitin carboxyl-terminal hydrolase 40	Q8BWR4-1
KMFVNSNHL	9		102.1	Nucleolar pre-ribosomal-associated protein 1	Q571H0
IQLVNAKHI	9		102.4	3-hydroxy-3-methylglutaryl-coenzyme A reductase	Q01237
GTFVNGEFV	9		102.5	Interferon-activable protein 203	O35368-3; O35368
AAIQGAVAM	9	1xOxidation [M9]	102.7	(E3-independent) E2 ubiquitin-conjugating enzyme UBE2O	Q6ZPJ3
KSTVNKWTL	9		103.3	WD repeat-containing protein 48	Q8BH57; Q8BH57-2; Q8BH57-3
SASSNPFAL	9		103.4	GATOR complex protein WDR59	Q8C0M0-1; Q8C0M0-2; Q8C0M0-3
GALGNYTWA	9		103.5	Macrosialin	P31996-2; P31996
AQMQNHSLEM	10		104.3	Transforming acidic coiled-coil-containing protein 3	Q9JJ11; Q9JJ11-2
YQQMNPEAL	9		104.4	Transcription factor MafB	P54841
AAELNSETL	9		104.4	Probable tRNA (uracil-O(2)-)-methyltransferase	Q9D2Q2
RMIQNQNAA	9		104.5	5'-3' exoribonuclease 2	Q9DBR1; Q9DBR1-2
FSHPNVLPVL	10		104.5	Integrin-linked protein kinase	O55222
SLIRNLEQL	9		105	Syntaxin-binding protein 2	Q64324
AGPSNFGTEL	10		105	Roquin-2	P0C090
TAVTNSARL	9		105.2	Vacuolar protein sorting-associated protein 33B	P59016
YTLLNKAPEYL	11		105.2	Ras GTPase-activating protein-binding protein 2	P97379-1; P97379-2
YTVENAKDII	10		105.5	Tryptophan--tRNA ligase, cytoplasmic	P32921-2; P32921
AIMENANVL	9		106.3	Fructose-bisphosphate aldolase A	P05064
KGPSNVFKI	9		106.4	Superkiller viralicidic activity 2-like 2	Q9CZU3
HSLINPEVF	9		106.6	CCR4-NOT transcription complex subunit 1	Q6ZQ08-2; Q6ZQ08-4; Q6ZQ08
SIRNLDTI	8		106.8	Ataxin-10	P28658
ASPLNPDMKM	10		106.9	Putative Polycomb group protein ASXL2	Q8BZ32
IQVPNCDEI	9		106.9	Coatomer subunit alpha	Q8CIE6
SALVKEEYL	9		107.2	dystonin	Q91ZU6-3; Q91ZU6-4; Q91ZU6-2; Q91ZU6
NGPQNIYNL	9		107.7	Ribonuclease kappa	Q8K3C0
MALTNVDTP	9		107.7	Cell division cycle 5-like protein	Q6A068
LMGVNLHEL	9	1xOxidation [M2]	107.9	Transcription initiation factor TFIID subunit 3	Q5HZG4
LAPQNKPEL	9		108.1	Protein FAM107B	Q3TGF2
SMLQNPLGNV	11		108.3	Cytoplasmic polyadenylation element-binding protein 1	P70166
RSPGNSPTPM	10		108.4	BAG family molecular chaperone regulator 4	Q8CI61
SSEINSAYL	9		109.2	Zinc finger CCCH-type antiviral protein 1	Q3UPF5; Q3UPF5-2
FAVTHQESI	9		109.7	Interactor protein for cytohesin exchange factors 1	Q5DU31-2; Q5DU31; Q5DU31-3
GALKNGQNL	9		109.8	Protein-glucosylgalactosylhydroxylysine glucosidase	Q8BP56-3; Q8BP56
GGIQNVGHI	9		110.3	inosine-5'-monophosphate dehydrogenase 2	P24547
NALHNIKTL	9		110.4	CUGBP Elav-like family member 2	Q9Z0H4-1; Q9Z0H4-7; Q9Z0H4-11; Q9Z0H4-9; Q9Z0H4-4; Q9Z0H4-8; Q9Z0H4-6; Q9Z0H4-2; Q9Z0H4-5
WGFVNKDQI	9		110.8	CD81 antigen	P35762
SSLQNHNHQL	10		111.5	E3 ubiquitin-protein ligase BRE1A	Q5DTM8; Q5DTM8-2; Q3U319
LMKVNLEYL	9		111.8	Armadillo-like helical domain-containing protein 3	Q6PD19-3; Q6PD19

FQVKNPPAAY	11		111.9	Isoform 2 of Formin-like protein 1	Q9JL26-2; Q9JL26
GALKNLSVIYI	11		112.1	E3 ISG15--protein ligase Herc6	F2Z461
SVIQNLRTV	9		113	26S proteasome non-ATPase regulatory subunit 1	Q3TXS7
ESPINVTTV	9		113.2	Wings apart-like protein homolog	Q65Z40
GNVTNITTV	9		113.4	Transforming growth factor beta activator LRRC33	Q8BMT4; Q8BMT4-2; Q8BMT4-3
RALQLLDEV	9		113.7	Mothers against decapentaplegic homolog 4	P97471
SSFVNEATF	9		114.5	WD40 repeat-containing protein SMU1	Q3UKJ7
SSIGNGDGI	9		114.6	Mitogen-activated protein kinase kinase kinase kinase 5	Q8BPM2-2; Q8BPM2
SHLLNTSTL	9		114.7	Zinc finger protein 106	O88466-3; O88466-1; O88466-2
SAIINPPQACIL	12		114.7	›oyllysine-residue acetyltransferase component of pyruvate dehydrogenase complex, mito	Q8BMF4
AACLNFNCM	9		114.7	Cytochrome b-245 heavy chain	Q61093
RQVENVLSL	9		115	Protein FAM173A	Q501J2
KTVVNISSL	9		115.1	Sepiapterin reductase	Q64105
RGPMNQCLV	9		115.1	Urokinase plasminogen activator surface receptor OS=Mus musculus OX=10090	P35456
RALELLNYL	9		115.8	Pre-mRNA-splicing factor ATP-dependent RNA helicase DHX15	O35286
TGISSIDFI	9		115.9	CD180 antigen	Q62192
SQVTLREPL	9		116.1	cytoplasmic FMR1-interacting protein 1	Q7TMB8-2; Q7TMB8-1
AILENANVL	9		116.3	Fructose-bisphosphate aldolase C	P05063
AMYVHAYTL	9		116.6	Proteasome subunit alpha type-3	O70435
FCLQNAQHDP	11		117	ıine nucleotide-binding protein G(I)/G(S)/G(O) subunit gamma-5 OS=Mus musculus OX=1	Q80SZ7
TSLKNAGTCL	10		117.7	D-3-phosphoglycerate dehydrogenase	Q61753
FATNNSEHITY	12		118.1	Cell division cycle 5-like protein	Q6A068
AAGVNSEMV	9		118.2	WD repeat-containing protein 82	Q8BFQ4
IINENYDYL	9		118.4	nuclear pore complex protein Nup98-Nup96	Q6PFD9
SVLRNPFETI	10		118.6	Claspin	Q80YR7
RQAENGMYI	9	1xOxidation [M7]	119	CAD protein	B2RQC6-2; B2RQC6
FSILNTPKKL	10		119.5	nuclear mitotic apparatus protein 1	E9Q7G0
SAPDNVLEV	9		119.9	E3 ubiquitin-protein ligase HECTD1	Q69ZR2
TGVVNNNEM	9		120.1	Helicase-like transcription factor	Q6PCN7
RSVDNIQFL	9		120.5	Sugar transporter SWEET1	Q9CXK4
SGPTNASAF	9		120.6	Microtubule-associated protein 4	P27546; P27546-2; P27546-3
QQVINIDQL	9		120.7	Glutamyl-tRNA(Gln) amidotransferase subunit B, mitochondrial	Q99JT1
SSLLNIQHF	9		121.1	UHRF1-binding protein 1-like	A2RSJ4
TSPTNSQQL	9		121.1	AT-rich interactive domain-containing protein 5B	Q8BM75; Q8BM75-2
LVIFNQEVI	9		121.4	Paired amphipathic helix protein Sin3a	Q60520-2; Q60520-1
SAVTNSGVHLI	11		121.4	GTP-binding protein 8	Q9CY28-2; Q9CY28-1
WKVVNPYYL	9		121.8	5'-AMP-activated protein kinase catalytic subunit alpha-1	Q5EG47
KQIYNLIHL	9		121.9	Protein fem-1 homolog B	Q9Z2G0
VAPYNTTQF	9		121.9	Protein HEXIM1	Q8R409
FQALNAEKL	9		122.1	serine/threonine-protein kinase ATR	Q9JKK8
NGILNVSAV	9		122.3	Heat shock cognate 71 kDa protein	P63017
SAPVNVGNTA	12		122.4	MICAL-like protein 2	Q3TN34
KGVYNKSTI	9		123.2	structural maintenance of chromosomes flexible hinge domain-containing protein 1	Q6P5D8
FSPLNPVRV	9		124.7	Heterogeneous nuclear ribonucleoprotein H	O35737; Q9Z2X1-2; Q9Z2X1-1
SGGVNLQSM	9		124.7	proliferating cell nuclear antigen	P17918
SIMYNVTEL	9		124.8	Trafficking protein particle complex subunit 13	Q3TIR1-2; Q3TIR1; Q3TIR1-3
AKLENAEVL	9		124.9	Transcription cofactor HES-6	Q9JHE6
TAAFNKDAL	9		125.1	Phosphatidylinositol 4,5-bisphosphate 3-kinase catalytic subunit beta isoform	Q8BTI9; O35904
RSVLNNQLL	9		125.4	CAP-Gly domain-containing linker protein 1	Q922J3-2; Q922J3-1
SSCINTEHI	9		125.7	zinc finger protein 27	P10077
NSLLNNMEL	9		125.7	Calcium-binding mitochondrial carrier protein Aralar1	Q8BH59; Q9QXX4
SQIDNKAVL	9		125.8	F-box/LRR-repeat protein 4	Q8BH70

FTLANVKEL	9		126.4	SHC SH2 domain-binding protein 1	Q9Z179
RTIRNAMKI	9		126.6	Eukaryotic translation initiation factor 3 subunit C	Q8R1B4
SSLINHQRV	9		126.7	Zinc finger protein 287	Q9EQB9
DAVANLTQL	9		127	AP-1 complex subunit gamma-like 2	O88512
VSLLNYQSAL	10		127.3	Homeodomain-interacting protein kinase 1	O88904-1; Q9QZR5; O88904-3; O88904-2
AAVSNKFRDLI	11		127.3	Conserved oligomeric Golgi complex subunit 4	Q8R1U1
KALINSVIKI	10		127.5	Lysosomal-trafficking regulator	P97412-2; P97412
HTGSNHTYL	9		127.6	Inner nuclear membrane protein Man1	Q9WU40-2; Q9WU40
SAIRSGLEL	9		128.7	Sister chromatid cohesion protein PDS5 homolog A	Q6A026
AAIVDGDRI	9		129.5	Serine/threonine-protein kinase MRCK beta	Q7TT50
TVHENLNFM	9		129.6	Vacuolar protein sorting-associated protein 11 homolog	Q91W86
AGLCNRAVF	9		130.6	Sodium/potassium-transporting ATPase subunit alpha-1	Q8VDN2; Q6PIC6; Q6PIE5
GQAQNKVYL	9		130.8	Rho guanine nucleotide exchange factor 10-like protein	A2AWP8; A2AWP8-5; A2AWP8-4; A2AWP8-2; A2AWP8-3
AAPISTTEL	9		131	Tyrosine-protein kinase BTK	P35991
RSHKNVADDY	11		131.6	sorting nexin-5	Q9D8U8
RSLDNGGYYI	10		131.8	Tyrosine-protein kinase Blk	P25911-1; P16277; P25911-2
NSTENSFTL	9		131.8	Ribonucleoside-diphosphate reductase subunit M2	P11157
SAPSSPDAL	9		131.9	Aprataxin and PNK-like factor	Q9D842-2; Q9D842; Q9D842-3
INITNGEEV	9		132	Casein kinase I isoform alpha	Q8BK63-2; Q8BK63
TAVRNGFHSL	10		132.6	e3 ubiquitin-protein ligase UBR4	A2AN08-3; A2AN08-4; A2AN08-5; A2AN08
SSPINISGQVI	11		132.6	Kinesin-like protein KIF20B	Q80WE4; Q80WE4-4; Q80WE4-2; Q80WE4-3
FQVDNFEAL	9		133.1	Integrin alpha-M	P05555; P05555-2
CQHENLVEL	9		133.2	Interleukin-1 receptor-associated kinase 4	Q8R4K2
AGVINRERI	9		133.9	Isoform A1-I of V-type proton ATPase 116 kDa subunit a isoform 1	Q9Z1G4-2; Q9Z1G4-3; Q9Z1G4
VQVPNLESL	9		134	Chromodomain-helicase-dna-binding protein 8	Q09XV5
AAATGATPI	9		134.3	40S ribosomal protein SA	P14206
SAIKNEVDSTV	11		135.4	Interferon regulatory factor 2	P23906
KAVENYLIQM	10		136	Programmed cell death protein 5	P56812
SQLQGYTVL	9		136.5	Selenocysteine lyase	Q9JLI6
FVYENPVSL	9		136.9	Rhomboid domain-containing protein 2	Q8VEK2
SSPSSRETL	9		138.7	Carnosine synthase 1	Q6ZPS2-2; Q6ZPS2-3; Q6ZPS2
FSIKNKKGSEL	11		138.8	Moesin	P26041
KALGNEIVV	9		139.4	SLAM family member 5	Q18PI6; Q18PI6-3
NAPPSNPVAM	10		139.7	Transcription initiation factor TFIID subunit 1	Q80UV9-3; Q80UV9; Q80UV9-2
RACANLQVL	9		139.8	Toll-like receptor 2	Q9QUN7
SAVVHEYAL	9		141.4	Sterol O-acyltransferase 1	Q61263
AAPANPEMDN	11		141.7	bcl-2 homologous antagonist/killer	O08734
GAISNYVSRTN	14		141.8	Calcium-responsive transactivator	Q8BW22
QMGVNPTSI	9		142.1	Isoform 3 of Aprataxin	Q7TQC5-3; Q7TQC5-1
TCLENGSFL	9		142.2	Protein Churchill	Q6DG52-2; Q6DG52
TGIKNGVHFL	10		143.3	Prolactin regulatory element-binding protein	Q9WUQ2
CSISNDKFEYL	11		144.2	Transcription factor Dp-1	Q08639
VTLGNPDVL	9		144.2	Mitochondrial import receptor subunit TOM40 homolog	Q9QYA2
SSPLNDISTSV	12		144.6	Serine/threonine-protein kinase WNK1	P83741-2; P83741; P83741-3; P83741-5; P83741-4
VAVKNSGGFL	10		145	Extended synaptotagmin-2	Q3TZZ7-2; Q3TZZ7
SAPLNNRCV	9		145.4	Collagen type IV alpha-3-binding protein	Q9EQG9-1
VTLANKELL	9		145.7	squalene monooxygenase	P52019
KAITDIIEM	9		145.8	Rab5 GDP/GTP exchange factor	Q9JM13
STLRNIRTV	9		146	Inhibitor of nuclear factor kappa-B kinase-interacting protein	Q9DBZ1
ACPFNGWYM	9	1xOxidation [M9]	146	Nitric oxide synthase, inducible	P29477
SSVENIQRV	9		146.9	Junction plakoglobin	Q02257
VSPTHMYFI	9		147	Protein phosphatase 1B	P36993; P36993-4; P36993-5; P36993-3

FHVQNIDLL	9	147.2	ATPase family AAA domain-containing protein 5	Q4QY64
RTVENIKDPL	10	147.5	Cytoplasmic dynein 1 heavy chain 1	Q9JHU4
FHLQNGDVV	9	147.6	Kinesin-associated protein 3	P70188-1; P70188-2
FAVISRHSL	9	147.8	Multidrug resistance-associated protein 1	O35379
VSPQNVHHSY	11	147.9	Isoform 3 of HBS1-like protein	Q69ZS7-3
EAIKNHEEL	9	148	45 kDa calcium-binding protein	Q61112-1; Q61112-2
RVVANSEEI	9	148.1	ATP-binding cassette sub-family D member 1	P48410
EAVVNTQEL	9	148.6	Pre-mRNA-processing-splicing factor 8	Q99PV0
AALNNHREV	9	148.9	E3 ubiquitin-protein ligase MIB2	Q8R516; Q8R516-2
SMKTNREEL	9	149	Pericentrin	P48725-1; P48725-3
AQLRNLDNA	9	149.3	ATP-dependent RNA helicase DHX33	Q80VY9
LANVNIGSL	9	149.8	60S acidic ribosomal protein P1	P47955
AMHRNLVDNI	10	150.1	plectin	Q9QXS1-6; Q9QXS1-13; Q9QXS1-7; Q9QXS1-9; Q9QXS1-5; Q9QXS1-14; Q9QXS1-12; Q9QXS1-3; Q9QXS1-2; Q9QXS1-10; Q9QXS1-15; Q9QXS1-11; Q9QXS1-16; Q9QXS1-4; Q9QXS1-1; Q9QXS1-8
NSVINVSNL	9	150.2	S-phase kinase-associated protein 2	Q9Z0Z3-1; Q9Z0Z3-2
ILHTNLVYL	9	151	Protein SSXT	Q62280
SVIQNNEHV	9	151.5	FERM domain-containing protein 6	Q8C0V9-2; Q8C0V9-3; Q8C0V9
CTIRNTEAV	9	152	Probable phospholipid-transporting ATPase VA	O54827; Q8K2X1-1
SGLINHQRI	9	152.4	zinc finger protein 24	Q91VN1
YMMVMDHHL	9	152.4	copper-transporting ATPase 1	Q64430
NGVINGAEL	9	152.8	Probable global transcription activator SNF2L2	Q6DIC0
SQKENLNFL	9	153.5	ATPase family AAA domain-containing protein 2	Q8CDM1-2; Q8CDM1
AIENIDTL	8	153.5	Protein phosphatase 1 regulatory subunit 7	Q3UM45
KAVVSQDAL	9	154.3	GDNF-inducible zinc finger protein 1	Q4VBD9
DAPRNLILI	9	154.4	Oxidoreductase NAD-binding domain-containing protein 1	Q8VE38; Q8VE38-2
IGIANFAEQI	10	154.5	Kelch-like ECH-associated protein 1	Q9Z2X8
RVLYNLEVL	9	154.8	Ubiquitin carboxyl-terminal hydrolase 24	B1AY13
SSPENKNWLL	10	155.3	Denticleless protein homolog	Q3TLR7-2; Q3TLR7
AMWANEQAL	9	155.4	Cytochrome b-245 light chain	Q61462; Q61462-2
FSEKNTDHI	9	155.5	Ras-related protein Rab-43	Q8CG50
YAITTLHNL	9	155.5	Junction plakoglobin	Q02257; Q02248
VSTSNVSSL	9	156	Fatty acid synthase	P19096
TGPFNLQLI	9	156.5	CAD protein	B2RQC6-2; B2RQC6
FQAINAGHI	9	157.4	Protein TANC1	Q0VGY8-2; Q0VGY8
AAVWNAQEA	9	157.7	AH receptor-interacting protein	O08915
STLRLLTTI	9	158.1	Sister chromatid cohesion protein PDS5 homolog B	Q4VA53-3; Q4VA53-2; Q4VA53
GQLSNGDHHF	11	158.5	YTH domain-containing family protein 1	P59326
SQFPNAEKM	9	158.7	Dedicator of cytokinesis protein 2	Q8C3J5
STVENFSQL	9	158.8	nucleolar pre-ribosomal-associated protein 1	Q571H0
LGIVNVDEA	9	159.1	Phosphoinositide 3-kinase adapter protein 1	Q9EQ32-2; Q9EQ32
QTVLNPVTM	9	159.2	Max-binding protein MNT	O08789
VMPQNQKAI	9	159.6	ATP synthase subunit d, mitochondrial	Q9DCX2
YGLKNHKLL	9	159.9	Protein mono-ADP-ribosyltransferase PARP8	Q3UD82
AGPENSSKI	9	160	autophagy-related protein 2 homolog B	Q80XK6-5; Q80XK6
GTGTNACYM	9	160	Hexokinase-3	Q3TRM8; P17710; P17710-2; P17710-4; P17710-3; Q91W97
YTIELLDTV	9	160	Small G protein signaling modulator 2	Q80U12; Q80U12-2
VSHENGEQTL	10	160.3	von Willebrand factor A domain-containing protein 8	Q8CC88
KGIGNKTEI	9	161.1	Histone-lysine N-methyltransferase NSD3	Q6P2L6-2; Q6P2L6-4; Q6P2L6
KNLLNVDKI	9	161.5	Dedicator of cytokinesis protein 10	Q8BZN6-3; Q8BZN6-4; Q8BZN6-1; Q8BZN6-2

VALLNETESVL	11		161.7	Metastasis-associated protein MTA3	Q924K8; Q924K8-2
FSQENTEKI	9		162	DEP domain-containing protein 1A	Q8CIG0-2; Q8CIG0
MAPQNLSTFC	11		162.1	DnaJ homolog subfamily B member 11	Q99KV1
KQGQNLWFL	9		162.2	Tyrosine-protein kinase BAZ1B	Q9Z277; Q9Z277-2
FALELQHAL	9		162.5	Patatin-like phospholipase domain-containing protein 7	A2AJ88-3; A2AJ88; A2AJ88-2
IAPKNPTISL	10		162.6	TOM1-like protein 1	Q923U0
TAIENSWIHL	10		162.9	Methyltransferase-like protein 16	Q9CQG2; Q9CQG2-2
FSLAHITQL	9		163.8	Ras suppressor protein 1	Q01730
NAVRNLQELL	10		163.8	Centrosomal protein of 70 kDa	Q6IQY5-2; Q6IQY5
AAPRSFIFL	9		164.7	N6-adenosine-methyltransferase subunit METTL14	Q3UIK4-1; Q3UIK4-2
GASVNNTTL	9		164.9	Protein fem-1 homolog C	Q8CEF1
YQYQNIFGPL\	11		165.1	Regulator of nonsense transcripts 1	Q9EPU0; Q9EPU0-2
TSPKNAAVF	9		165.5	PAN2-PAN3 deadenylation complex catalytic subunit Pan2	Q8BGF7-2; Q8BGF7; Q8BGF7-3; Q8BGF7-4
YAVGDTITM	9		165.8	RING finger protein 10	Q3UIW5; Q3UIW5-2
AAPAGTTFV	9		166	RNA-binding protein 38	Q62176
TSAYNTEVL	9		166.2	Friend leukemia integration 1 transcription factor	P26323
KCIENLEEL	9		166.8	Protein phosphatase 1 regulatory subunit 7	Q3UM45
AGLVNIVSF	9		167	SNF-related matrix-associated actin-dependent regulator of chromatin subfamily A-like pro	Q8BJL0-3; Q8BJL0; Q8BJL0-2
YCYDNIHFM	9		167.6	Basic leucine zipper and W2 domain-containing protein 1	Q91VK1; Q9CQC6
GQMSNGEHH\	11		167.6	YTH domain-containing family protein 3	Q8BYK6-2; Q8BYK6-3; Q8BYK6
SGPLDSTFI	9		167.7	Suppressor APC domain-containing protein 2	Q9D818-2; Q9D818; Q9D818-3
SAGNNGPCL	9		169.1	Tripeptidyl-peptidase 2	Q64514; Q64514-2
GTFVNTELI	9		169.3	Serine/threonine-protein kinase Chk2	Q9Z265
RTPQNTISI	9		169.3	G-protein coupled receptor 176	Q80WT4
AAVVMLDRI	9		169.4	E3 ubiquitin-protein ligase RNF213	E9Q555
SAPSSSTPF	9		169.9	Chromodomain-helicase-DNA-binding protein 1	P40201
IGVRNENTFL	10		170	UTP--glucose-1-phosphate uridylyltransferase	Q91ZJ5-2; Q91ZJ5-1
SSCENGTFF	9		170	B-cell lymphoma 6 protein homolog	P41183
FFHNNMEYM	9		170.2	NADH-ubiquinone oxidoreductase chain 5	P03921
FSVENKWRL	9		170.4	Cytochrome c oxidase subunit 7C, mitochondrial	P17665
VAYKNALANPI	11		170.6	Cytochrome b-c1 complex subunit 2, mitochondrial	Q9DB77
SAPSSPAEYI	10		171.3	Probable ATP-dependent RNA helicase DDX31	Q6NZQ2
YGISDGLTL	9		172.7	F-box/LRR-repeat protein 6	Q9QXW0-2; Q9QXW0; Q9QXW0-3
ICIGNTAYI	9		172.9	AP-5 complex subunit mu-1	Q8BJ63
FNVRNGYGFI	10		174.7	Y-box-binding protein 3	Q9JKB3-1; Q9Z2C8-2; Q9JKB3-2; P62960; Q9Z2C8-1
KAPHNVKQDF	11		175.1	MAX gene-associated protein	A2AWL7-2; A2AWL7-3; A2AWL7-4; A2AWL7
YSYQSRFHL	9		175.4	Isoform 2 of Splicing factor 3B subunit 3	Q921M3-2; Q921M3-1
SQLIHLIYI	9		175.4	FAST kinase domain-containing protein 5, mitochondrial	Q7TMV3
AVLRNLCLL	9	xCarbamidomethyl [C7	175.5	Peroxisomal acyl-coenzyme A oxidase 1	Q9R0H0-1; Q9R0H0-2
SIVNEDFL	8		175.5	Alkyldihydroxyacetonephosphate synthase, peroxisomal	Q8C0I1
MVLVNNVEM	9		175.5	DNA polymerase delta catalytic subunit	P52431
SGHTNAVATV	10		176	Pleiotropic regulator 1	Q922V4
ISPQNGRYQI	10		176.3	Mitogen-activated protein kinase kinase kinase 8	Q07174
SSVRNSKSL	9		177.4	muskelin	O89050
SSPLNSRAV	9		178	Baculoviral IAP repeat-containing protein 2	Q62210
NQITDLEYL	9		179.8	Leucine-rich melanocyte differentiation-associated protein	Q9D9B4
SAPQDIYAV	9		179.9	mRNA export factor	Q8C570
KTIKNTVCV	9		180.1	Kinesin-1 heavy chain	Q61768
YAVNNQFTMG	13		181.2	Histone-arginine methyltransferase CARM1	Q9WVG6; Q9WVG6-2
AAVINPPQACI	11		181.4	Pyruvate dehydrogenase protein X component, mitochondrial	Q8BKZ9
SSLMKATVL	9		181.9	7-methylguanosine phosphate-specific 5'-nucleotidase	Q3UFY7-3; Q3UFY7; Q3UFY7-2
RSFSLRDYL	9		181.9	Zinc finger and BTB domain-containing protein 45	Q52KG4

TNPLNGQYI	9	182	Neurogenic locus notch homolog protein 2	O35516; O35516-2
SALVGASEL	9	182.7	Protein KIAA0100	Q5SYL3
SSPSSTMTM	9	182.8	Host cell factor 1	Q61191
VAPASVITI	9	183.2	Transcription factor MafF	O54791
AAPTNRQIEILI	13	183.5	Nuclear pore complex protein Nup160	Q9Z0W3
SAPTARVFM	9	183.6	Pre-mRNA-processing factor 6	Q91YR7
RSLISMEEI	9	183.9	Translin-associated protein X	Q9QZE7
FTIRNKKGTEL	11	184.3	merlin	P46662-1; P46662-2
HAMNNLNGTE	11	184.5	RNA-binding protein 47	Q91WT8-1; Q91WT8-2
SQLVKADEM	9	184.6	DnaJ homolog subfamily C member 3	Q91YW3
SSFENLHFENI	11	185.7	DNA helicase B	Q6NVF4
SGCLNGSFL	9	185.9	Probable E3 ubiquitin-protein ligase HERC4	Q6PAV2-2; Q6PAV2
FGITNLVEHPA	11	186	U4/U6 small nuclear ribonucleoprotein Prp3	Q922U1
GTGSNACYM	9	186.4	Hexokinase-1	O08528; P17710; P17710-2; P17710-4; P17710-3
LGPENCATL	9	186.9	DENN domain-containing protein 4C	A6H8H2; A6H8H2-2
SQYFNRAPVL	10	188	ATP-dependent RNA helicase DHX33	Q80VY9
TTHSNHEVL	9	188.3	Pyrroline-5-carboxylate reductase 3	Q9DCC4
SAAFNQLPHL	10	188.6	Cytoplasmic polyadenylation element-binding protein 4	Q7TN98-5; Q7TN98; Q7TN98-2; Q7TN98-3; Q7TN98-4
RQFPNRDAL	9	190.1	RNA-binding protein 5	Q91YE7-2; Q91YE7
MAPENKVADW	11	190.2	Centrosomal protein of 170 kDa	Q6A065
GSPVNQQPKK	14	190.2	Protein FAM219A	Q9D772
STCVNSYAI	9	191	ATP-binding cassette sub-family G member 2	Q7TMS5
ITGTNAEVM	9	191	Glutamine synthetase	P15105
RQGANINEI	9	191.2	Poly(RC)-binding protein 1	P60335
ASPEGQDYL	9	192.1	tRNA-splicing ligase RtcB homolog	Q99LF4
NAGENSTSL	9	192.1	Oxidative stress-responsive serine-rich protein 1	Q9D722
FVISNYREQL	10	192.7	uncharacterized protein C5orf34 homolog	Q3UJC8-1; Q3UJC8-2
SMGVNDIDI	9	193	Zinc finger CCCH domain-containing protein 15	Q3TIV5-1; Q3TIV5-2
ACPQCNAEYL	10	193.1	E3 ubiquitin-protein ligase MARCH5	Q3KNM2
RQPQNPPATI	10	193.8	Telomeric repeat-binding factor 2	O35144; O35144-2
GSLTNVKAL	9	193.9	Serine/threonine-protein kinase B-raf	P28028
TCVSNGPFI	9	194.3	Polypyrimidine tract-binding protein 1	P17225
TQFPNLEVL	9	194.8	Toll-like receptor 13	Q6R5N8
VIAINQDPL	9	195	Alpha-galactosidase A	P51569
ASLTSRTSM	9	195.7	Mitofusin-2	Q80U63; Q811U4
SAILGQMTL	9	195.9	Multidrug resistance-associated protein 5	Q9R1X5
GAIRNACQML	10	196	Cullin-3	Q9JLV5
KTLGNVIFM	9	196.3	Rho guanine nucleotide exchange factor 6	Q8K4I3
CSGENFVEI	9	196.4	Cell division cycle-associated protein 2	Q14B71
WSPHNETIL	9	196.5	Histone-binding protein RBBP7	Q60973; Q60972
QGVKNNDVI	9	196.9	Focal adhesion kinase 1	P34152-5; P34152-2; P34152-6; P34152-4; P34152
RSLRHVNIDHL	11	197.7	Eukaryotic translation initiation factor 3 subunit H	Q91WK2
AGLRNAVSQM	10	198.4	Coiled-coil domain-containing protein 57	Q6PHN1
FGLQNDHCVF	11	198.9	Transforming acidic coiled-coil-containing protein 3	Q9JJ11
VSGDNAEPL	9	199.1	Kelch-like protein 21	Q3U410
FVIRNIVEA	9	199.6	40S ribosomal protein S26	P62855
VTVENVQEL	9	200	Kelch-like protein 12	Q8BZM0; Q8BZM0-2
AQTSNSQEI	9	200.1	Sp110 nuclear body protein	Q8BVK9
TQNVNQAKM	9	200.7	Melanoma-associated antigen D1	Q9QYH6
TGVQNFSRI	9	201.8	Scavenger receptor class B member 1	Q61009; Q61009-2
SSINNLQTNTV	11	202	Nuclear factor of activated T-cells 5	Q9WV30-4; Q9WV30; Q9WV30-2
YKNVNQEVV	9	202.3	Oxidoreductase htatip2	Q9Z2G9-2; Q9Z2G9-1

TAHYNRERI	9		203.2	Pre-mRNA-processing-splicing factor 8	Q99PV0
FQLINVEDF	9		203.3	RNA helicase aquarius	Q8CFQ3
YASENVNKL	9		203.4	Ras-related protein Rab-1A	P62821; Q9D1G1
QTILNSTMM	9		203.5	Tetratricopeptide repeat protein 27	Q8CD92-2; Q8CD92
SAVSNNYIQTL	12		203.8	Catenin delta-1	P30999-2; P30999-1; P30999-3
RNLGNTCYM	9	xCarbamidomethyl [C7	203.9	Ubiquitin carboxyl-terminal hydrolase 44	Q8C2S0; Q6P8X6-1; Q6P9L4; Q80U87
TQPLNGRTI	9		204.2	Carbonic anhydrase 9	Q8VHB5
IQVKNEVSL	9		204.3	Neutral amino acid transporter B(0)	P51912
VSLRNSISNFL	11		205.3	Acetyl-CoA carboxylase 1	Q5SWU9-2; Q5SWU9-1
KTNQNFTHL	9		206	NACHT, LRR and PYD domains-containing protein 3	Q8R4B8-3; Q8R4B8-1
FAHIDGDHL	9		206.4	Pre-mRNA-splicing factor ATP-dependent RNA helicase DHX15	O35286
VGPLNEDVF	9		207.1	Period circadian protein homolog 2	O54943
SAPRNFVENF	10		207.3	elongator complex protein 2	Q91WG4-2; Q91WG4
RAPTSTDTPI	10		207.3	Tyrosine-protein phosphatase non-receptor type 18	Q61152
AAPISPWTM	9		209.3	zinc finger protein 746	Q3U133
AIHNFDFL	8		209.4	Probable cation-transporting ATPase 13A3	Q5XF89-2; Q5XF89
HGIENFINEAS	13		210.1	Glutamate dehydrogenase 1, mitochondrial	P26443
RQIQNQLEETL	11		210.7	Brain-specific angiogenesis inhibitor 1-associated protein 2	Q8BKX1-3; Q8BKX1; Q8BKX1-2; Q8BKX1-4
DSPANIEVL	9		210.7	Protein Spindly	Q923A2
CAPSSRYTL	9		210.9	Alpha/beta hydrolase domain-containing protein 17C	Q8VCV1
SSPTNSAITQL	11		211.2	Zinc finger E-box-binding homeobox 2	Q9R0G7
ASLRDKDTL	9		211.6	isoleucine--tRNA ligase, cytoplasmic	Q8BU30
VSVQHVCTI	9		212.1	Hexokinase-1	P17710-1
NQYENAEKL	9		214.4	Amyloid protein-binding protein 2	Q9DAX9
SAPVSVSAV	9		214.5	Rho GTPase-activating protein 31	A6X8Z5
RSLLNWRTKL	10		215.2	pre-rRNA processing protein FTSJ3	Q9DBE9
VVVENGELI	9		215.2	DNA-directed RNA polymerase II subunit RPB1	P08775
FCIENLQKL	9		215.3	Acetyl-CoA carboxylase 1	Q5SWU9-1
FGPINSVAF	9		215.5	Eukaryotic translation initiation factor 3 subunit I	Q9QZD9
GQGRNPFFL	9		215.8	Integrator complex subunit 6	Q6PCM2-1; Q8BND4; Q6PCM2-3; Q6PCM2-2
QAPINFTSRL	10		216.3	DDB1- and CUL4-associated factor 1	Q80TR8-1; Q80TR8-4; Q80TR8-2
SLLQNGVNL	9		216.6	Erbin	Q80TH2-2; Q80TH2; Q80TH2-1
AQVENVQRI	9		216.9	Centrosomal protein of 83 kDa	Q9D5R3
HMGMNHMEM	9		216.9	High affinity copper uptake protein 1	Q8K211
SLTMDGGLRN	14		217.5	Serine/threonine-protein kinase TBK1	Q9WUN2
GAIVDQMTL	9	1xOxidation [M7]	217.7	Lysosomal alpha-mannosidase	O09159
KQSLNGSYM	9		217.9	Histone acetyltransferase KAT6B	Q8BRB7-2; Q8BRB7
AAPTNRQIEIL	11		218.7	Nuclear pore complex protein Nup160	Q9Z0W3
SMIRLSESM	9		220.5	DNA replication licensing factor MCM6	P97311
LCPANHAPL	9		221.5	Junction plakoglobin	Q02257; Q02248
YALKSQEML	9		221.6	Fanconi anemia group B protein homolog	Q5XJY6
SSLINHKSV	9		223.2	Zinc finger protein 62	Q8C827-3; Q8C827-2; Q8C827
KGQLNADEI	9		223.7	Eukaryotic translation initiation factor 4 gamma 2	Q62448
YGISNEKPEV	10		225	RNA polymerase II-associated factor 1 homolog	Q8K2T8
RALKNCRLEL	10		225.7	Carbonyl reductase [NADPH] 1	P48758
RCPENAFFL	9		226.6	Unconventional myosin-Ic	Q9WTI7; Q9WTI7-3; Q9WTI7-2; Q9WTI7-4
SQVSDYDYF	9		227.5	Protein MTSS 1	Q8R1S4; Q8R1S4-2
GAVTLQEYL	9		228.8	ARF GTPase-activating protein GIT1	Q68FF6
SSLLNNKHFLI	11		228.8	Plexin-D1	Q3UH93
RQVTNPESVL	10		229	Tyrosine--tRNA ligase, mitochondrial	Q8BYL4
AAGTNKENL	9		229.5	BCL-6 corepressor	Q8CGN4-1; Q8CGN4-2; Q8CGN4-4; Q8CGN4-3
FSKKNFESL	9		230	Dolichyl-diphosphooligosaccharide--protein glycosyltransferase subunit 2	Q9DBG6

KSCMNQVTV	9		230	Serine/threonine-protein kinase Chk1	O35280-2; O35280
SAISNASGERL	11		231.5	E3 ubiquitin-protein ligase mib1	Q80SY4
LSGEFQIVNPH	13		235.2	Ribonucleoside-diphosphate reductase large subunit	P07742
KTIYNVEHL	9		235.7	E3 ubiquitin-protein ligase RNF103	Q9R1W3
KQVENVVRM	9		236	Protein FAM173B	Q9D1Z3
NAPVNPTRAE	11		236.8	phorbol-12-myristate-13-acetate-induced protein 1	Q9JM54
RSGGNLEVM	9		236.8	COP9 signalosome complex subunit 5	O35864
CAVIDVDVI	9		237	dephospho-CoA kinase domain-containing protein	Q8BHC4
AMMAKAEYL	9		237.1	26S proteasome non-ATPase regulatory subunit 6	Q99JI4
SLPTNLIHL	9		238.7	E3 ubiquitin-protein ligase UBR2	Q6WKZ8-1; Q6WKZ8-3; Q6WKZ8-2
AAPINTQGL	9		239.4	Histone deacetylase complex subunit SAP130	Q8BIH0-1; Q8BIH0-2
ICIANTHLL	9		239.6	protein angel homolog 2	Q8K1C0-1
NSISNPVTKEM	11		240.3	mRNA-capping enzyme	O55236
SAPSADAPM	9		240.7	glyceraldehyde-3-phosphate dehydrogenase	P16858
SSLPNLAISTI	11		240.9	E3 ubiquitin-protein ligase RNF213	E9Q555
VNVENQTKI	9		244.4	Armadillo repeat-containing protein 10	Q9D0L7; Q9D0L7-2
VAVYNHYKRI	10		245.7	ATP-dependent Clp protease ATP-binding subunit clpX-like, mitochondrial	Q9JHS4
RTISNPEVVM	10		246	Afadin	Q9QZQ1; Q9QZQ1-2
SSISNSLENAL	11		247.7	Zinc finger CCHC domain-containing protein 14	Q8VIG0; Q8VIG0-3; Q8VIG0-2
HTVQNADQV	9		248	Antigen peptide transporter 2	P36371
VSPRNPAHF	9		248.1	Endoplasmic reticulum metallopeptidase 1	Q3UVK0
NCILNAEAL	9		248.7	Prolyl 3-hydroxylase 1 OS=Mus musculus OX=10090	Q3V1T4
SGIINFIHF	9		249.4	Kinetochore protein Nuf2	Q99P69-2; Q99P69
RAIDNAADL	9		249.7	Sorting nexin-8	Q8CFD4
FQKQNVTIM	9	1xOxidation [M9]	250.7	Nitric oxide synthase, inducible	P29477
ATILNLERV	9		250.7	Conserved oligomeric Golgi complex subunit 4	Q8R1U1
SQAVNKQQI	9		251	Cyclin-F	P51944-2; P51944; P51944-3
FVRINQDYI	9		251.6	S1 RNA-binding domain-containing protein 1 OS=Mus musculus OX=10090	Q497V5-2; Q497V5; Q497V5
AALTLLEKM	9		251.7	Exocyst complex component 6	Q8R313
ILHENFTTV	9		253.2	Cytoplasmic dynein 1 light intermediate chain 2	Q6PDL0
RAIYNNMKQFI	11		253.4	Sarcoplasmic/endoplasmic reticulum calcium ATPase 2	Q64518-3; Q64518-2; O55143-2; Q64518; O55143-1; Q8R429
AAIDNGLAFPL	11		253.4	Phosphatidylinositol 4-kinase type 2-alpha	Q2TBE6
YAVGNHDFIEA	11		254.4	PCI domain-containing protein 2	Q8BFV2
AAMLLQQVM	9		254.5	E3 UFM1-protein ligase 1	Q8CCJ3-3; Q8CCJ3-1
WAPHMNSVHI	10		255.1	Ankyrin repeat domain-containing protein 17	Q99NH0-1
LSGLNQEEL	9		256.6	Zinc finger CCCH-type antiviral protein 1	Q3UPF5; Q3UPF5-2
RAPENLTLSNL	11		256.9	Cytokine receptor common subunit gamma	P34902
FSIFNRSIDAF	11		257.7	Kit ligand	P20826; P20826-2
KSARNFNLPM	10		257.8	Succinyl-CoA:3-ketoacid coenzyme A transferase 1, mitochondrial	Q9D0K2
NALLNSLEF	9		258.5	Importin subunit beta-1	P70168
FHIGINRYEL	10		258.8	Extracellular serine/threonine protein kinase FAM20C	Q5MJS3
AQLQNQQSF	9		259.4	Coiled-coil-helix-coiled-coil-helix domain-containing protein 2	Q9D1L0
FTISNGGVF	9		259.4	lysine-residue succinyltransferase component of 2-oxoglutarate dehydrogenase complex,	Q9D2G2-2; Q9D2G2
SSVVGVWYL	9		259.7	Minor histocompatibility antigen H13	Q9D8V0; Q9D8V0-3; Q9D8V0-4
RIVENVNVI	9		260.1	Elongation factor 2	P58252
HSLVLVDEL	9		260.9	DNA mismatch repair protein Msh6	P54276
VGLENVSNV	9		261.6	Transformation/transcription domain-associated protein	Q80YV3
SALVHSQEL	9		262.8	Baculoviral IAP repeat-containing protein 3	O08863
AIVRNLESL	9		263	28S ribosomal protein S6, mitochondrial	P58064
SQVINPTAITV	11		263.2	Host cell factor 1	Q61191
SALEFLTHL	9		263.5	BRCA1-associated ATM activator 1	Q8C3R1-2; Q8C3R1

IGINLTDPM	9		264.6	Putative deoxyribonuclease TATDN1	Q6P8M1-2; Q6P8M1
IQWPNGITL	9		264.8	Very low-density lipoprotein receptor	P98156; P35951
TALRLITAL	9		265.3	Mediator of RNA polymerase II transcription subunit 23	Q80YQ2-2; Q80YQ2
VSARNIMLL	9		266.1	cold shock domain-containing protein E1	Q91W50
YAFKNPIYS	9		269.3	terminal uridylyltransferase 7	Q5BLK4
SSPEGVETM	9		269.5	Telomere-associated protein RIF1	Q6PR54-2; Q6PR54-3; Q6PR54
RAPTGYDVV	9		270.4	Multivesicular body subunit 12B	Q6KAU4
FMYTTPFTL	9		270.6	Dedicator of cytokinesis protein 8	Q8C147
SSMKNFKAFF	10		270.8	Anaphase-promoting complex subunit 4	Q91W96
AIQNSTSI	8		272.9	Cullin-4B	A2A432-2; A2A432
SALVSSLHM	9		273.2	Coatomer subunit gamma-2	Q9QXK3-3; Q9QXK3-2; Q9QXK3; Q9QXK3-4
SLISNGPSL	9		274.2	AT-rich interactive domain-containing protein 2	E9Q7E2
RNLGNTCFM	9	xCarbamidomethyl [C7	274.4	Ubiquitin carboxyl-terminal hydrolase 2	O88623-2; O88623-1; O88623-4; O88623-3; Q91W36
GGVVNPNEV	9		274.5	Transcription factor AP-2-alpha	P34056-3; P34056-4; P34056; P34056-2
SGPRDHVFI	9		275	Legumain	O89017
SSPSSPTAL	9		275.5	Isoform 5 of Calmodulin-binding transcription activator 2	Q80Y50-5; Q80Y50-2; Q6NVG1; Q80Y50-4; Q80Y50-3; Q80Y50-1
SSPQSTEHPM	10		275.8	Rho GTPase-activating protein 44	Q5SSM3; Q5SSM3-2; Q5SSM3-3
FSGTNDPCAL	10		276.2	Macrophage migration inhibitory factor	P34884
GSGTNVCEL	9		276.2	Protein C11orf74 homolog	Q9CQI4
RAPVKYDHI	9		276.8	Protein salvador homolog 1	Q8VEB2
TSVVLLTEM	9		277.5	AP-1 complex subunit gamma-1	P22892
FSPENYKDHS	11		277.9	Little elongation complex subunit 2	Q3UZ18-1; Q3UZ18-2
SNVVNQANL	9		278.2	Protein NDRG4	Q8BTG7-3; Q8BTG7; Q8BTG7-2
SSPRSDVPV	9		279.1	U3 small nucleolar RNA-associated protein 14 homolog A	Q640M1
YEGINVDVI	9		279.7	Probable ATP-dependent RNA helicase DDX52	Q8K301
RAYTKFYTL	9		281.2	DENN domain-containing protein 4C	A6H8H2; A6H8H2-2
GMHQNTLRI	9		282.2	60S ribosomal export protein NMD3	Q99L48-1; Q99L48-2
AAPESFEEL	9		283.5	Nucleolar protein 14	Q8R3N1
NQVVLLDTL	9		283.6	Biogenesis of lysosome-related organelles complex 1 subunit 6	Q9R0C0-1
RSLVSLTHL	9		283.8	Toll-like receptor 13	Q6R5N8
RALINSRTF	9		284	Putative Polycomb group protein ASXL1	P59598
NSTFNQVVL	9		284.1	60S ribosomal protein L18	P35980
YCIQNACKDAI	11		284.6	ne nucleotide-binding protein G(I)/G(S)/G(O) subunit gamma-10 OS=Mus musculus OX=1	Q9CXP8
VDVTNTTFL	9		284.9	Coatomer subunit epsilon	O89079
AAVGNCAEHM	10		286.2	Coiled-coil-helix-coiled-coil-helix domain-containing protein 5	Q9CQP3
QQYYNSDHM	9		286.3	H/ACA ribonucleoprotein complex non-core subunit NAF1	Q3UMQ8
SSVRDRDLL	9		288	Nesprin-3	Q4FZC9-1; Q4FZC9-2
SAVYNSFRAVI	11		288.9	Peroxisomal membrane protein pex13	Q9D0K1
RSLLLLAPL	9		289.2	Procollagen-lysine,2-oxoglutarate 5-dioxygenase 1	Q9R0E2
YQNINSTGL	9		290.2	Transmembrane protein 87B	Q8BKU8-2; Q8BKU8-3; Q8BKU8
SSPYSPAYL	9		291.4	CREB-regulated transcription coactivator 2	Q3U182
HSCWNLSTI	9		291.5	NACHT, LRR and PYD domains-containing protein 3	Q8R4B8-2; Q8R4B8-3; Q8R4B8
FSPLNPVRVHI	11		292.3	Heterogeneous nuclear ribonucleoprotein H	O35737; Q9Z2X1-2; Q9Z2X1-1
FSPLNPMRVH	11		292.3	heterogeneous nuclear ribonucleoprotein H2	P70333
FGLQNDHCVF	10		295.3	Transforming acidic coiled-coil-containing protein 3	Q9JJ11; Q9JJ11-2
FVHENIETF	9		296.5	Transmembrane protein 245	B1AZA5
SSVRDTDSL	9		296.7	LisH domain-containing protein ARMC9	Q9D2I5-9; Q9D2I5-4; Q9D2I5-3; Q9D2I5-2; Q9D2I5-1; Q9D2I5-5; Q9D2I5-8
SSLLNSEFC	9		297	Protein flightless-1 homolog	Q9JJ28
YAFWNKEDF	9		297.3	Transmembrane protein 180	Q6PDE8
FSSTNPTQV	9		298.7	Proliferation marker protein Ki-67	E9PVX6-2; E9PVX6

LINQNSDYL	9		299.8	TELO2-interacting protein 1 homolog	Q91V83
NQMVGIEYI	9		300.5	Mediator of RNA polymerase II transcription subunit 6	Q921D4
ALINKDFL	8		302.2	Transmembrane protein 82	Q8R115
FMSRTNRPPL	10		302.5	60S ribosomal protein L18	P35980
SSAKNFITKM	10		302.6	UDP-glucose:glycoprotein glucosyltransferase 1	Q6P5E4
SSPTDKHTL	9		303.5	Plasma membrane calcium-transporting ATPase 1	Q6Q477-2; Q6Q477; G5E829; Q9R0K7
RQGKNLMFL	9		303.8	Asparagine--tRNA ligase, cytoplasmic	Q8BP47
SGITNSASSTL	11		304.4	Cell division cycle 5-like protein	Q6A068
SSVSKQETI	9		304.5	Transcriptional repressor p66-beta	Q8VHR5
SCILNGTVML	10		305.7	Sodium bicarbonate cotransporter 3	Q8BTY2; Q8BTY2-2
SCLRNVTQTL	10		306.1	Rab3 GTPase-activating protein non-catalytic subunit	Q8BMG7
FSLTNGMYP	9		306.4	60S ribosomal protein L6	P47911
DQVENHEFL	9		307.2	DNA mismatch repair protein MSH2	P43247
SSLLGGDVV	9		307.2	TSC22 domain family protein 3	Q9Z2S7
AQVQNSEQLL	10		309	rab GTPase-binding effector protein 2	Q91WG2-2; Q91WG2-3; Q91WG2-1
FSSENQQFV	9		309.4	Protein strawberry notch homolog 2	Q7TNB8; Q7TNB8-2
SQSMNFSLM	9		311.1	Clathrin interactor 1	Q99KN9; Q99KN9-2
SMPISEETL	9	1xOxidation [M2]	311.5	Next to BRCA1 gene 1 protein	P97432
ISGWNGDNM	9		312.3	Elongation factor 1-alpha 1	P10126
AAPCAVSYL	9		312.9	Cartilage-associated protein	Q9CYD3
RSGVNSELV	9		313.2	Protein DEK	Q7TNV0
TGILNPEGYTL	11		313.4	Poly [ADP-ribose] polymerase 2	O88554
AAVIAHDFL	9		313.6	G1/S-specific cyclin-D3	P30282
NAPQNPESKL	10		313.6	GON-4-like protein	Q9DB00
GSLVHISYL	9		313.8	DNA-directed RNA polymerase II subunit RPB1	P08775
NGVSNGLHL	9		316.3	WD and tetratricopeptide repeats protein 1	Q80ZK9
VTVENTKTI	9		316.5	Zinc finger CCCH domain-containing protein 13	E9Q784
GQLSNGEPHF	11		316.7	YTH domain-containing family protein 2	Q91YT7
NAIANGNYP	9		317	Catalase	P24270
FQLRSFDQL	9		319.4	Hypoxia-inducible factor 1-alpha	Q61221-2; Q61221
SQLANEKLTQI	11		319.4	Isoform 2 of Rho-associated protein kinase 1	P70335-2; P70335-1
ASLVNKIGL	9		321.7	Uroporphyrinogen-III synthase	P51163
FSVENKWRLL	10		322	Cytochrome c oxidase subunit 7C, mitochondrial	P17665
STVQNAEAYV	11		322.9	Ubiquitin carboxyl-terminal hydrolase 33	Q8R5K2-2; Q8R5K2
FQYELNNPM	9		323.8	Neutral alpha-glucosidase AB	Q8BHN3-3; Q8BHN3-2; Q8BHN3
SAPKYIDYL	9		324.3	mob kinase activator 1b	Q8BPB0; Q921Y0-1
SGLRNLREPIG	12		324.8	B-cell CLL/lymphoma 9 protein	Q9D219
GCLVNRDLV	9		325	Protein KIAA0556	Q8C753-3; Q8C753-4; Q8C753; Q8C753-2
NCIVNVFAV	9		325.5	Dynein light chain Tctex-type 3 OS=Mus musculus OX=10090	P56387
FQNFGNVVEL	10		325.8	Ras GTPase-activating protein-binding protein 1	P97855
VQNINIENL	9		326.6	Protein S100-A8	P27005
TMDGGLRNVD	12		326.8	Serine/threonine-protein kinase TBK1	Q9WUN2
AGIENDEAF	9		330.5	Clathrin light chain A	O08585
AGIENYFIGL	10		330.6	C-type lectin domain family 5 member A	Q9R007; Q9R007-2; Q9R007-1
ALHVNGESL	9		331.8	Histone-lysine N-methyltransferase EHMT1	Q5DW34-2; Q5DW34; Q5DW34-3
AIMPNGDHI	9		332.1	Platelet-activating factor acetylhydrolase IB subunit alpha	P63005-1; P63005-2
SHVLNDTHM	9		332.6	Plexin-C1	Q9QZC2
STVRNADVIA	10		334.9	Multidrug resistance protein 1B	P06795; P21447
IMTQNVERI	9		335.4	vesicle-associated membrane protein 8	O70404
YMHRNREIT	9		335.4	Ubiquitin carboxyl-terminal hydrolase 25	P57080
HCLANVSAM	9		335.7	toll-like receptor 4	Q9QUK6
YVVDNIDHL	9		336.9	E3 ubiquitin-protein ligase RFWD2	Q9R1A8

ACLFNQEAL	9	338.9	Nesprin-1	Q6ZWR6-4; Q6ZWR6
AAIKDNVVI	9	339.8	AP-3 complex subunit mu-1	Q9JKC8
NSMVLFDHM	9	340.5	DNA topoisomerase 2-beta	Q64511
GQHPNIITL	9	341.1	Ribosomal protein S6 kinase alpha-2	Q9WUT3; P18653; P18654
SVLSNSANL	9	342.9	Proliferation marker protein Ki-67	E9PVX6-2; E9PVX6
FCLQNAQHDP	12	343.9	Guanine nucleotide-binding protein G(I)/G(S)/G(O) subunit gamma-5	Q80SZ7
VSPVSKTAM	9	344.2	nuclear factor related to kappa-B-binding protein	Q6PIJ4
YQVGNLAGHT	11	345.3	ABI gene family member 3	Q8BYZ1-2; Q8BYZ1
VGVINLDGL	9	346.1	NADH-ubiquinone oxidoreductase chain 6	P03925
AAEINQAAL	9	346.4	Short transient receptor potential channel 4-associated protein	Q9JLV2; Q9JLV2-2
YKAENVEVI	9	347.5	Cytochrome P450 4V2	Q9DBW0
RALFNGAQKL	10	347.7	Protein transport protein Sec61 subunit alpha isoform 1	P61620; Q9JLR1
NSVVNPNKAT	11	348	OTU domain-containing protein 5	Q3U2S4-2; Q3U2S4-1
SALILRTKL	9	352.6	Tetratricopeptide repeat protein 27	Q8CD92-2; Q8CD92
VGVVSVEFI	9	352.8	Next to BRCA1 gene 1 protein	P97432
HALDNGLFTL	10	353.3	Protein FRG1	P97376
NGVINHSETI	10	356.1	FH1/FH2 domain-containing protein 3	Q76LL6-2; Q76LL6-4; Q76LL6; Q76LL6-3
QMHQNFEEQI	10	357.9	TBC1 domain family member 15	Q9CXF4
FAPNSVHYL	9	359.6	Ran-binding protein 17	Q9EPK7-2; Q99NF8; Q9EPK7
YVITDQIPV	9	359.8	Transmembrane glycoprotein NMB	Q99P91
KNMQNVEHV	9	360.8	Proteasome subunit beta type-1	O09061
SSIQLKTNFL	10	362	HEAT repeat-containing protein 5A	Q5PRF0-1
SSVVGVTSL	9	362.8	NEDD4-like E3 ubiquitin-protein ligase WWP2	Q9DBH0
VAISNLPAATF	11	363.3	UDP-galactose translocator	Q9R0M8
SLVKNVAHM	9	364.6	Zinc finger protein 622	Q91VY9
NNIRNQAHM	9	365.9	Mitochondrial inner membrane protease ATP23 homolog	Q9CWQ3
ASMSNDFKSV	11	367.8	Syntaxin-5	Q8K1E0-2; Q8K1E0-1
SSVKNVPENL	10	367.9	aminoacyl tRNA synthase complex-interacting multifunctional protein 2	Q8R010
YSCKNLIDL	9	368	Histone H4 transcription factor	Q8K1K9
FTDVNKTEM	9	368.3	Centrosome-associated protein 350	E9Q309
AGIENKFGLYL	11	368.5	Calcium-binding mitochondrial carrier protein Aralar1	Q8BH59; Q9QXX4
FGVSNPASA	9	369.8	Nucleoporin p58/p45	Q8R332
SSMENDSSNL	10	370.1	Ubiquitin-associated protein 2-like	Q80X50-4; Q80X50-3; Q80X50-5; Q80X50-2; Q80X50
YSLPKLTCM	9	370.3	BTB/POZ domain-containing protein 9	Q8C726
STTVNGDVF	9	371.1	Clathrin light chain B	Q6IRU5-3; Q6IRU5-2; Q6IRU5
HTVQNADQVL	10	371.3	antigen peptide transporter 2	P36371
YQRRNKDVL	9	371.5	UPF0472 protein C16orf72 homolog	Q14AM7
SGPVSLQTL	9	372	WD repeat-containing protein WRAP73	Q9JM98
SAPRDLFDM	9	373.1	SHC-transforming protein 1	P98083-3; P98083-1; P98083-2
SQMKNIKKM	9	373.5	Asparagine--tRNA ligase, cytoplasmic	Q8BP47
AAITNKYQLVF	11	373.9	RNA polymerase I-specific transcription initiation factor RRN3	B2RS91
RTVENVTVF	9	374.4	Synaptic vesicle membrane protein VAT-1 homolog	Q62465
LCPANPTCI	9	379.1	Nuclear cap-binding protein subunit 1	Q3UYV9
AAPCNDLHA	9	379.8	Serine/threonine-protein kinase pim-1 OS=Mus musculus OX=10090	P06803
SQMTNLQELH	11	380.5	volume-regulated anion channel subunit LRRC8D	Q8BGR2
KIHVNGVDYL	10	380.6	28S ribosomal protein S9, mitochondrial	Q9D7N3
YAPSPNSDDF	10	381.1	Transcription factor 12	Q61286-2; Q61286
KLHLNLTEL	9	381.4	Nck-associated protein 1-like	Q8K1X4
SAIKGLSAL	9	381.9	SRA stem-loop-interacting RNA-binding protein, mitochondrial	Q9D8T7-2; Q9D8T7
YIHINIGAL	9	382.8	probable ATP-dependent RNA helicase DDX5	Q61656
AVLRNQIHV	9	384.9	COP9 signalosome complex subunit 1	Q99LD4; Q99LD4-2
RASTNAMLI	9	385.4	Pre-mRNA-splicing factor ATP-dependent RNA helicase DHX15	O35286

ALRNQATM	8		386.4	Diacylglycerol kinase zeta	Q80UP3
YAPDSIYYL	9		386.6	Zinc finger MYM-type protein 2	Q9CU65
FVARNSSEL	9		387.3	Epidermal growth factor receptor kinase substrate 8	Q08509
QLLNTDYL	8		387.8	Pericentriolar material 1 protein	Q9R0L6; Q9R0L6-2
ASMLNDELSH	11		391	E3 ubiquitin-protein ligase TRAF7	Q922B6
GGLTNGSGRY	11		391.3	Pumilio homolog 1	Q80U58-3; Q80U58-2; Q80U58; Q80U78-2; Q80U78; Q80U78-3
AAPTNANSLNS	13		391.7	CCR4-NOT transcription complex subunit 4	Q8BT14; Q8BT14-2; Q8BT14-3
DALENLRVYL	10		392.2	B-cell lymphoma/leukemia 10	Q9Z0H7
YDVENGEFL	9		394.3	E3 ubiquitin-protein ligase UBR2	Q6WKZ8; Q6WKZ8-3; Q6WKZ8-2
HTIRNYPATV	10		395.2	Lysine-specific histone demethylase 1A	Q6ZQ88
YHPGVNGPPL	10		396.4	Serine/threonine-protein phosphatase 1 regulatory subunit 10	Q811B5; Q80W00; Q80W00-2
FSLAPYRDYL	10		397.2	Uncharacterized protein C15orf39 homolog	Q3TEI4
KQVKNGPSL	9		405.9	Regulation of nuclear pre-mRNA domain-containing protein 2	Q6NXI6; Q6NXI6-2
YAFNMKATV	9		406.3	Heat shock cognate 71 kDa protein	P63017
YGYSNRVVDL	12		406.6	glyceraldehyde-3-phosphate dehydrogenase	P16858
KTVVNKDVF	9		409.2	60S ribosomal protein L27	P61358
SSHENRPLDLI	11		410	E3 ubiquitin-protein ligase HUWE1	Q7TMY8-4; Q7TMY8-3; Q7TMY8; Q7TMY8-2
SACTFGDPI	9		410.6	Cathepsin Z	Q9WUU7
YCIGNMQML	9	1xOxidation [M6]	412.6	NFX1-type zinc finger-containing protein 1	Q8R151
VAPENEGRLV	12		412.7	Transmembrane protein 43	Q9DBS1
SSSLNQEKI	9		413.8	Protein TASOR 2	Q5DTT3
TALKNPNAML	10	1xOxidation [M9]	414.1	Ras GTPase-activating-like protein IQGAP1	Q9JKF1
SAIRGQAEV	9		415	Putative Polycomb group protein ASXL1	P59598
SALKLYENL	9		415.9	N-alpha-acetyltransferase 30	Q8CES0-2; Q8CES0
SSPVSNVAI	9		416.1	Histone-lysine N-methyltransferase SETD5	Q5XJV7; Q5XJV7-2
VAPSSVVEL	9		417.8	Exportin-5	Q924C1; Q924C1-2
ASVLLQDHI	9		418.9	Atlastin-3	Q91YH5-1
TAPLNTQIPTL	11		419	B-cell CLL/lymphoma 9 protein	Q9D219
SAPQDKGFL	9		420.3	Receptor-interacting serine/threonine-protein kinase 2	P58801
AQYRLVTEI	9		421.3	probable ATP-dependent RNA helicase DDX10	Q80Y44
LSILNSNEHL	10		421.4	Centrosomal protein of 85 kDa	Q8BMK0
SSVTGLTNI	9		422.6	Ras GTPase-activating-like protein IQGAP1	Q9JKF1
YINFNVDKL	9		425.5	Deoxynucleotidyltransferase terminal-interacting protein 2	Q8R2M2
SVFPNQDEM	9		430.4	Tubulin gamma-1 chain	P83887; Q8VCK3
YTNGNASLI	9		430.6	Protein mono-ADP-ribosyltransferase PARP14	Q2EMV9; Q2EMV9-2
AAYRNLGQNL	10		432.5	Protein RCC2	Q8BK67
AAPINPSDINM	12		434.7	Enoyl-[acyl-carrier-protein] reductase, mitochondrial	Q9DCS3
RSPINCLEHV	10	xCarbamidomethyl [C6	435	Membralin	Q8CIV2-1; Q8CIV2-2
YMLALKQEL	9		436.9	DNA-directed RNA polymerase III subunit RPC7	Q6NXY9-2; Q6NXY9-1
GSPVNGSSQL	10		438.1	Nucleoporin SEH1	Q8R2U0; Q8R2U0-2
KTVVDIDTI	9		438.6	Peroxisomal 2,4-dienoyl-CoA reductase	Q9WV68
GQVKNYCLTL	10		439.5	Neutrophil cytosol factor 2	O70145
AGLTHLITM	9	1xOxidation [M9]	443	Phosphoribosyl pyrophosphate synthase-associated protein 1	Q9D0M1; Q8R574
TSSINGDLM	9		443.8	Serine/threonine-protein phosphatase 6 regulatory subunit 3	Q922D4-3; Q922D4-2; Q922D4; Q922D4-4
FRMPVLREYL	10		447.5	Diacylglycerol O-acyltransferase 2	Q9DCV3
QSLVNLRELYL	11		449.4	Protein phosphatase 1 regulatory subunit 7	Q3UM45
SYGQLSNGEP	13		456.1	YTH domain-containing family protein 2	Q91YT7
IVGINLTEM	9		456.2	ELMO domain-containing protein 2	Q8BGF6
AAIKLQELL	9		456.7	Ribosome-binding protein 1	Q99PL5-1
RHMQNSEII	9		457.5	Cohesin subunit SA-2	O35638
STGQNISQI	9		458.8	Aryl hydrocarbon receptor nuclear translocator 2	Q61324; Q61324-2

VVVTDMDTI	9	459	Ubiquitin-like modifier-activating enzyme 1	Q02053
YAYETKDAL	9	460	G protein-coupled receptor kinase 6	O70293; Q8VEB1; O70293-2
VSSINQEAL	9	462.6	Chromatin accessibility complex protein 1	Q9JKP8
TSATNGPSL	9	463.6	Helicase with zinc finger domain 2	E9QAM5
YAVEGRDLTL	10	464.5	Translocon-associated protein subunit beta	Q9CPW5
AAVINPPQACI	12	468.2	yruvate dehydrogenase protein X component, mitochondrial OS=Mus musculus OX=1009	Q8BKZ9
IGIQNFPEGL	10	471	Zinc transporter ZIP11	Q8BWY7-2; Q8BWY7; Q8BWY7-3
AALENTHLLEV	13	472.9	Cytosolic iron-sulfur assembly component 2B	Q9D187
YAYDGCDYI	9	473.9	H-2 class I histocompatibility antigen, K-B alpha chain	P01901; P04223; P04223-2
SSVKNEEQF	9	474.1	Maternal embryonic leucine zipper kinase	Q61846
SAIHILDVL	9	475.1	Cap-specific mRNA (nucleoside-2'-O-)-methyltransferase 1	Q9DBC3
FSGTDRETL	9	476	Pecanex-like protein 3	Q8VI59-1
YAVNNQFTMG	12	476.2	Histone-arginine methyltransferase CARM1	Q9WVG6; Q9WVG6-2
SAPTNTGSTG	12	477.4	Nuclear pore complex protein Nup54	Q8BTS4
EGINNLTHM	9	479.2	Liprin-beta-1	Q8C8U0-2; Q8C8U0-1; Q8C8U0-3
SPQNSDYL	8	479.5	Sortilin	Q6PHU5; Q6PHU5-2
TTPQNQVDM	9	482.2	Charged multivesicular body protein 1b-1	Q99LU0; Q9CQD4
YVTTNPQEL	9	482.2	Protein DGCR6	O35347-2; O35347
RSITNTTVC	9	485.6	Splicing factor 1	Q64213; Q64213-2; Q64213-3
SALVSSLHL	9	485.8	Coatomer subunit gamma-1	Q9QZE5
KQIRLLEAM	9	486.2	Tumor necrosis factor alpha-induced protein 2	Q61333
SMAGNIIPAI	10	486.5	SUMO-activating enzyme subunit 2	Q9Z1F9
IQYSNHREL	9	487	polypyrimidine tract-binding protein 3	Q8BHD7-2; Q8BHD7
ASLQNFNISNL	11	488.1	Serine/threonine-protein kinase WNK1	P83741-2; P83741; P83741-3; P83741-5; P83741-4
SAAQNAFKGN	12	488.5	Secretory carrier-associated membrane protein 1	Q8K021
TVISNAVDHI	10	489	N-alpha-acetyltransferase 25, NatB auxiliary subunit	Q8BWZ3-2; Q8BWZ3
QNPTNAEVL	9	489.9	Isoform Smooth muscle of Myosin light polypeptide 6	Q8CI43; Q60605; Q60605-2; P09541
RQVVHTETL	9	490.7	Ankyrin repeat domain-containing protein 13D	Q6PD24
SAKNKDYM	8	494.1	bifunctional UDP-N-acetylglucosamine 2-epimerase/N-acetylmannosamine kinase	Q91WG8
ASYVNLPTIAL	11	500	40S ribosomal protein SA	P14206
SGSLNAQVI	9	500.2	Mitochondrial import receptor subunit TOM40 homolog	Q9QYA2; Q9CZR3
VTIRLLETI	9	500.9	Methylsterol monooxygenase 1	Q9CRA4
FVTSNTQEL	9	501.3	Serrate RNA effector molecule homolog	Q99MR6-3; Q99MR6; Q99MR6-4; Q99MR6-2
ASVDNLLHL	9	502.1	Beta-1,4-glucuronyltransferase 1	Q8BWP8-2; Q8BWP8
SAPKSISAL	9	502.3	Roquin-1	Q4VGL6
RAVQDVESL	9	503.5	e3 ubiquitin-protein ligase rnf14	Q9JI90-1
SAPVAAEPF	9	505.5	2-oxoglutarate dehydrogenase, mitochondrial	Q60597-4; Q60597-3; Q60597-2; Q60597
VSPTGTHTL	9	507.9	Granulins	P28798
SGLTMITHL	9	508.1	G-protein coupled receptor-associated sorting protein 1	Q5U4C1; Q5U4C1-2
SAIVNLPGCSA	11	508.4	Coatomer subunit gamma-1	Q9QZE5
SVVQNQQQV	9	508.7	Nipped-B-like protein	Q6KCD5-1; Q6KCD5-4; Q6KCD5-2
SQMIQTEYL	9	510.3	Adhesion G protein-coupled receptor E1	Q61549
NQITNNQRI	9	512.3	phosphoglycerate kinase 1	P09041; P09411
SKCVNPVPI	9	512.7	Antileukoproteinase OS=Mus musculus OX=10090	P97430
RACYNLAAL	9	517.3	Integral membrane protein GPR137	Q80ZU9
VSLLDIDHL	9	521.7	HEAT repeat-containing protein 3	Q8BQM4
VSPTNPTKLQI	11	522.3	Disabled homolog 2-interacting protein	Q3UHC7-3; Q3UHC7-2; Q3UHC7
SSAVGLVTI	9	523.6	Monocarboxylate transporter 1	P53986
SSLWSLTHL	9	524.6	CCR4-NOT transcription complex subunit 6	Q8K3P5-2; Q8K3P5
FCLQNAQHDP	13	526.2	Guanine nucleotide-binding protein G(I)/G(S)/G(O) subunit gamma-5	Q80SZ7
KTISLTDFL	9	529.4	Eukaryotic translation initiation factor 4B	Q8BGD9
SSLRSLNNFI	10	529.7	Mitotic spindle assembly checkpoint protein MAD1	Q9WTX8; Q9WTX8-2

YVSQNKEFL	9		531.3	Angiomotin-like protein 1	Q9D4H4-2; Q9D4H4
SQQRNFQLL	9		531.6	DNA repair protein Rad50	P70388; P70388-2; P70388-3
VSPANNICF	9		534.7	Extracellular serine/threonine protein kinase FAM20C	Q5MJS3
NQFVNKFNVL	10		534.9	COP9 signalosome complex subunit 6	O88545
ASLKSTSTL	9		535.3	ATP-binding cassette sub-family B member 7, mitochondrial	Q61102
SALLNDCIRHL	11		536.1	Protein Niban	Q3UW53
VQNKNSSYF	9		536.8	Tubulin beta-2A chain	Q7TMM9; P99024; P68372; Q9CWF2
FLNVNCEHI	9		538.5	DNA replication licensing factor MCM4	P49717
SQLMMRTRI	9	1xOxidation [M4]	538.9	HIG1 domain family member 2A	Q9CQJ1
VCIINFQHL	9		539	Activating signal cointegrator 1 complex subunit 3	E9PZJ8-1
SGISLATHL	9		539	eIF-2-alpha kinase GCN2	Q9QZ05; Q9QZ05-3; Q9QZ05-4; Q9QZ05-2; Q9QZ05-6
VAVTNGGVEH	12		542.4	Leucine-rich repeat and calponin homology domain-containing protein 3	Q8BVU0
ACLQNVHKV	9		543.7	huntingtin	P42859
SSLNLRETNLE	14		544.8	Vimentin	P20152
NAVKNHWNST	11		545.3	Myb-related protein B	P48972
HLVTNQEAL	9		548.6	Isoform 2 of Guanylate-binding protein 5	Q8CFB4; Q8CFB4-2
VSLKNPVRIFV	11		550.9	Probable ATP-dependent RNA helicase DDX27	Q921N6-1
STHYNLHNL	9		551.9	Lysosomal alpha-glucosidase	P70699
HSLVHKVFL	9		557.1	Pumilio homolog 3	Q8BKS9
RGIANSLEKL	10		559.6	Nesprin-1	Q6ZWR6-4; Q6ZWR6-1
CSNKNLEKL	9		561.3	Microtubule-actin cross-linking factor 1	Q9QXZ0-3; Q9QXZ0-2; Q9QXZ0-4; Q9QXZ0
SGPRGVHFI	9		565	Cytochrome b-245 heavy chain	Q61093
SAVKNDYEMT	11		567.8	Replication protein A 70 kDa DNA-binding subunit	Q8VEE4
LAPVNIFKA	9		573.2	T-complex protein 1 subunit beta	P80314
AAVRLTELL	9		573.4	Baculoviral IAP repeat-containing protein 1e	Q9QUK4; Q9R016; Q9JIB6; Q9JIB3
AGLTHIITM	9		575	Phosphoribosyl pyrophosphate synthase-associated protein 1	Q9D0M1
FAPRDPIPYL	10		575.8	U1 small nuclear ribonucleoprotein 70 kDa	Q62376-1; Q62376-2
SHLINHVNI	9		575.9	Protein SMG5	Q6ZPY2-2; Q6ZPY2-1
LAPTNLSSSRM	11		576.6	serine/arginine repetitive matrix protein 2	Q8BTI8; Q8BTI8-2; Q8BTI8-3
VTPFNIDRL	9		578.1	DNA-directed RNA polymerase II subunit RPB1	P08775
LAYENVKEV	9		580.6	Cytoplasmic dynein 1 heavy chain 1	Q9JHU4
SSPKSTLTL	9		581.2	Tensin-3	Q5SSZ5; Q5SSZ5-2
VSLEKCDYI	9		582.2	Thioredoxin reductase 3	Q99MD6
NAVLNQRYLE	12		582.6	Coiled-coil domain-containing protein 125	Q5U465-2; Q5U465
FEHENKFYI	9		584.2	Vacuolar protein sorting-associated protein 29	Q9QZ88; Q9QZ88-2
WVLVNEKPL	9		585.5	Gamma-interferon-inducible lysosomal thiol reductase	Q9ESY9
SALGNVTTC	9		587	Eukaryotic translation initiation factor 6	O55135
SAPYGRITL	9		587.4	cytoplasmic FMR1-interacting protein 1	Q7TMB8-2; Q7TMB8-1; Q5SQX6
RSVTHFDSL	9		587.7	Long-chain-fatty-acid--CoA ligase 4	Q9QUJ7-1; Q9QUJ7-2
LSPNNHEVHI	10		587.8	Actin-related protein 2/3 complex subunit 1A	Q9R0Q6
SCGINLVTL	9		588.5	Lysosome-associated membrane glycoprotein 1	P11438
VAPENEGRL	9		590.9	Transmembrane protein 43	Q9DBS1
ACFFNQEVL	9		592.9	Xaa-Pro dipeptidase	Q11136
VIVNNTESI	9		592.9	WW domain-binding protein 2	P97765-2; P97765
VQPVGHDYV	9		595	Zinc finger RNA-binding protein	O88532
TCLENSSCI	9		595.3	Short transient receptor potential channel 4-associated protein	Q9JLV2; Q9JLV2-2
SGVDNAISL	9		595.7	U3 small nucleolar RNA-associated protein 18 homolog	Q5SSI6
RSFANDDRHV	11		597.8	spermatid perinuclear RNA-binding protein	Q91WM1; Q91WM1-2
MLSNTTAI	8		598.4	Tubulin alpha-3 chain	P05214; Q9JJZ2; Q3UX10; P68369; P05213; P68373; P68368
FSHHSMVSM	9		598.6	Ubiquitin-associated protein 2-like	Q80X50-4; Q80X50-3; Q80X50-5; Q80X50-2; Q80X50
ISAASITPL	9		600.6	Cell division cycle and apoptosis regulator protein 1	Q8CH18-2; Q8CH18; Q8CH18-3

GALGNTPPFL	10	601.9	YTH domain-containing family protein 3	Q8BYK6-2; Q8BYK6-3; Q8BYK6
SACWNSSHL	9	602.4	probable cation-transporting ATPase 13A3	Q5XF89-1
QAVINGPNV	9	602.6	DNA-directed RNA polymerase I subunit RPA1	O35134
SSGINVNAAPI	11	602.9	Caprin-1	Q60865
SAPTNTGSTG	11	604.1	Nuclear pore complex protein Nup54	Q8BTS4
AGGINVWAL	9	604.2	F-box DNA helicase 1	Q8K2I9; Q8K2I9-2
SQLPLLSTI	9	607	WD repeat-containing protein 76	A6PWY4; A6PWY4-2
SAISLKANI	9	608.2	Short transient receptor potential channel 4-associated protein	Q9JLV2-1; Q9JLV2-2
HSLENSLIDIM	11	609.7	Cytoplasmic polyadenylation element-binding protein 2	Q812E0
ALENTTSI	8	611.9	YrdC domain-containing protein, mitochondrial	Q3U5F4
GVHVNQELL	9	612.1	Vacuolar protein sorting-associated protein 8 homolog	Q0P5W1-2; Q0P5W1; Q0P5W1-3
SCPANLPNI	9	613.6	Microphthalmia-associated transcription factor	Q08874-7; Q08874; Q08874-9; Q08874-6; Q08874-2; Q08874-3; Q08874-4; Q08874-5; Q08874-8
CSQSNYQHI	9	614.4	AP-3 complex subunit delta-1	O54774
SAVVNKVPLS(	11	615.3	Transmembrane protein 263	Q9DAM7
SSSTNPSLL	9	615.8	CDKN2A-interacting protein	Q8BI72
IGITNHDEYSL	11	616.5	Talin-1	P26039
NSIIMLEAL	9	618.6	Small nuclear ribonucleoprotein G	P62309
FCAVNPKFM	9	618.7	Coronin-1A	O89053
RALDNDQHL	9	620.8	E3 ubiquitin-protein ligase RNF213	E9Q555
TGPENSNTDT	11	621.1	MAX gene-associated protein	A2AWL7-3; A2AWL7
HAITNSGQF	9	621.7	Nck-associated protein 1-like	Q8K1X4
SQGPGNRPPL	10	622.4	Lymphocyte cytosolic protein 2	Q60787
SQHQSSYGPI	10	622.5	Heterogeneous nuclear ribonucleoprotein A0	Q9CX86
SQTRNHIQTL	10	622.7	Chromosome transmission fidelity protein 18 homolog	Q8BIW9
SGLSGHTTL	9	623	Microtubule-associated protein 4	P27546; P27546-2; P27546-3; P27546-4
KQMLLFTHI	9	623.8	E3 ubiquitin-protein ligase HUWE1	Q7TMY8-4; Q7TMY8-3; Q7TMY8; Q7TMY8-2
NAIENSSSKV	10	626	Centrosomal protein of 162 kDa	Q6ZQ06
SAPQSAVELI	10	627.6	Clathrin interactor 1	Q99KN9; Q99KN9-2
KGITNHLVA	9	627.6	Macrophage-expressed gene 1 protein	A1L314
SVGLNSTAL	9	628.8	Serine beta-lactamase-like protein LACTB, mitochondrial	Q9EP89
FCLQNAQHDP	14	633	Guanine nucleotide-binding protein G(I)/G(S)/G(O) subunit gamma-5	Q80SZ7
NQHYNTSLL	9	634.7	E3 SUMO-protein ligase PIAS1	O88907
NCFKNRYTL	9	635.4	Cullin-1 OS=Mus musculus OX=10090	Q9WTX6
NSIKNHWNST	11	647.5	Myb-related protein A	P51960-2; P51960
CQVENGRVI	9	648.2	muscleblind-like protein 2	Q8C181; Q9JKP5
YAVTGDVKM	9	649.8	Nuclear factor NF-kappa-B p105 subunit	P25799-7; P25799-5; P25799-3; P25799-2; P25799-6; P25799
AQLAKKDYL	9	650.9	Neutrophil cytosol factor 2	O70145
FSCANGRCI	9	651.1	prolow-density lipoprotein receptor-related protein 1	Q91ZX7; A2ARV4
RSVIGSEFI	9	651.1	Dipeptidase 2	Q8C255; Q8C255-3
QGLTNNSLL	9	651.3	Ataxin-7	Q8R4I1
VGITNTVGSSI	11	652.5	Glutamine--fructose-6-phosphate aminotransferase [isomerizing] 1	P47856; P47856-2; Q9Z2Z9
ALENLSTL	8	653.5	Fatty acid synthase	P19096
SSAVCTEVI	9	654.3	Synergin gamma	Q5SV85; Q5SV85-2
FGLCHHGNYL	10	654.5	Prolow-density lipoprotein receptor-related protein 1	Q91ZX7
SSILNKYDDN\	11	654.7	AH receptor-interacting protein	O08915
VMAQNPYNA\	11	656.1	WD repeat-containing protein 46	Q9Z0H1
NAICCSDHL	9	657.2	Progranulin	P28798
INIMNHEKV	9	658.1	Ribosomal biogenesis factor	Q0VG62; Q0VG62-1
SGPAGAPPM	9	659.6	MAPK-interacting and spindle-stabilizing protein-like	Q8BH93
TVFKNVDLL	9	659.7	Nucleosome assembly protein 1-like 1	P28656

TSLVDADTF	9	661.2	Emerin	O08579
RAPEGDDFL	9	661.4	Splicing factor, arginine/serine-rich 19	Q5U4C3
NAPTCTVEI	9	661.6	Protein NLRC3	Q5DU56; Q5DU56-3
TGLLNDSTF	9	661.7	D-3-phosphoglycerate dehydrogenase	Q61753
VTIRLLDVL	9	662.1	Ataxin-10	P28658
RTVGNIEEL	9	662.2	Dehydrogenase/reductase SDR family member 11	Q3U0B3
SSPINLFNT	9	662.2	Major vault protein	Q9EQK5
YAGSNFPEHIF	14	662.3	Actin-related protein 2	P61161
FHHSNIEML	9	663.8	Disintegrin and metalloproteinase domain-containing protein 17	Q9Z0F8
TELQNMNEYL	10	663.8	Chromodomain-helicase-DNA-binding protein 5	A2A8L1
FTLVNKKNALI	11	667.3	EH domain-binding protein 1-like protein 1	Q99MS7-3; Q99MS7; Q99MS7-4; Q99MS7-5; Q99MS7-2
TCVSNITDI	9	667.9	Protein aurora borealis	Q8BS90-1
ISSVNVDQV	9	670.4	Centrosome and spindle pole associated protein 1	B2RX88-2; B2RX88; B2RX88-4
FQWPNSQDI	9	670.6	Putative Dol-P-Glc:Glc(2)Man(9)GlcNAc(2)-PP-Dol alpha-1,2-glucosyltransferase	Q3UGP8
RAPTGSTEL	9	672.9	Fanconi anemia core complex-associated protein 100	A2ACJ2
SAVSGPAEYL	10	674.1	Carnitine O-palmitoyltransferase 2, mitochondrial	P52825
YSPTGKEFV	9	675.2	DDB1- and CUL4-associated factor 13	Q6PAC3
KTVTNAVVTV	10	678.2	Heat shock cognate 71 kDa protein	P63017
SAPLGREHL	9	679.8	Lymphocyte cytosolic protein 2	Q60787
GQLSNGEPHF	10	680	YTH domain-containing family protein 2	Q91YT7
AQLSNSLQEG	12	682.4	Histone-lysine N-methyltransferase NSD2	Q8BVE8-2; Q8BVE8
SQPRLVDII	9	682.8	Elongator complex protein 3	Q9CZX0-2; Q9CZX0
SQILNLQTSLQ	11	683.4	multiple PDZ domain protein	Q8VBX6-4; Q8VBX6; Q8VBX6-2
HGITNLCVIG	10	684.6	ATP-dependent 6-phosphofructokinase, liver type	P12382
GMLRSLSTL	9	685.6	Ribonuclease inhibitor	Q91VI7
TATNNTSAIQD	13	687.6	Glutaredoxin-1	Q9QUH0
RQYPNPFQL	9	689.7	zinc finger protein 280c	Q6P3Y5-1; Q6P3Y5-2; Q6P3Y5-3
FMLALKENI	9	691.8	Protein diaphanous homolog 3	Q9Z207
YGYSNRVVDL	13	692.9	Glyceraldehyde-3-phosphate dehydrogenase	P16858
GACLNPVCL	9	693.2	Mitogen-activated protein kinase kinase kinase 7	Q62073
ICPVNRDTIDY	12	695.4	Diacylglycerol O-acyltransferase 2	Q9DCV3
SSPVCQEQL	9	695.9	Talin-2	Q71LX4; P26039
SAPAASTETL	10	696.1	Proline-serine-threonine phosphatase-interacting protein 1	P97814
AAPFDTVHI	9	700.1	tRNA (uracil-5-)-methyltransferase homolog A	Q8BNV1
TLIENGEKI	9	700.1	nuclear pore glycoprotein p62	Q63850
VLGQNAQTM	9	700.3	cold shock domain-containing protein E1	Q91W50
RAIELLEKL	9	702.6	Protein lin-7 homolog A	O88952; Q8JZS0
LSKINKDWL	9	710.4	Neutrophil cytosol factor 4	P97369
VSPENIIFT	9	711.3	Antizyme inhibitor 1	O35484
VAARNLMTSE	11	712.6	Centromere/kinetochore protein zw10 homolog	O54692
SQLIILDTL	9	714.2	Neurofibromin	Q04690-3; Q04690; Q04690-4; Q04690-2
TAIKNTSFC	9	717	Protein FAM135A	Q6NS59
STTKNRFVV	9	717.7	Eukaryotic translation initiation factor 4E	P63073
QMARNIWYF	9	717.9	Fatty acyl-CoA reductase 1	Q922J9-3; Q922J9-4; Q922J9
FSQGPGNRPF	11	718.9	lymphocyte cytosolic protein 2	Q60787
SAMEDLQDM	9	722.5	Alpha-actinin-4	P57780
FAIQNKHLC	9	722.6	Cyclic AMP-dependent transcription factor ATF-3	Q60765
KQLANEVLSVI	11	722.8	Nitric oxide synthase, inducible	P29477
SGVNRYYV	8	723.1	NADH dehydrogenase [ubiquinone] 1 alpha subcomplex subunit 1	O35683
TAGLNVGSL	9	723.8	Isoform 4 of CUGBP Elav-like family member 1	P28659-2; P28659; P28659-3; P28659-4
KGTSNITKL	9	724.8	Nuclear receptor subfamily 1 group D member 1	Q3UV55
SSPKNVQGL	9	725.3	sialoadhesin	Q62230-3; Q62230-2; Q62230

VMQENITKV	9		728.6	Vesicle-associated membrane protein 4	O70480
IQPDNIVYV	9		733.4	Signal recognition particle 54 kDa protein	P14576
GQLKNKYGDA	12		733.8	U6 snRNA-associated Sm-like protein LSm6	P62313
SRILNSTNI	9		734.9	Protein regulator of cytokinesis 1	Q99K43
SAVVNKVPLS(	12		739	Transmembrane protein 263	Q9DAM7
STIRLLTSL	9		743.3	T-complex protein 1 subunit gamma	P80318
VAPKNYSETIE	13		743.3	Double-strand break repair protein MRE11	Q61216; Q61216-2
RGVPNHIHM	9		745	Protein CASC3	Q8K3W3
SAPVAAEPFL	10		747.8	2-oxoglutarate dehydrogenase, mitochondrial	Q60597-4; Q60597-3; Q60597-2; Q60597
KAVANQTSAT	11		747.9	26S proteasome regulatory subunit 4	P62192
QIQNAQYL	8		748	Heterogeneous nuclear ribonucleoprotein K	P61979-3; P61979-2; P61979
TGVLDQTTI	9		753	Matrix metalloproteinase-24	Q9R0S2
HCPLNEEVI	9		753.1	E3 ubiquitin-protein ligase UBR1	O70481
HVLSNCELL	9		755.1	PX domain-containing protein kinase-like protein	Q8BX57; Q8BX57-2; Q8BX57-3
INVPNAEVL	9		755.3	Probable ubiquitin carboxyl-terminal hydrolase FAF-X	P70398
TSPLNPEAAVL	11		756	AKT-interacting protein	Q64362
VSVCNQIASHL	11		756.2	Choline/ethanolaminephosphotransferase 1	Q8BGS7; Q8BGS7-2
RNLVNAQQV	9		758.5	Terminal uridylyltransferase 4	B2RX14
HQMQLVDSI	9		762.1	Nucleoprotein TPR	F6ZDS4
HGAMNQQHM	9		763.4	Forkhead box protein J3	Q8BUR3-2; Q8BUR3
SSPTGGPTL	9		763.8	Collagen alpha-1(XV) chain	O35206
SAPTSPTRL	9		764.2	Mitochondrial dynamics protein MID51	Q8BGV8
VSPANLPAVLL	11		764.9	Host cell factor 1	Q61191
VSPKNPEQKI	10		767.4	Mitochondrial inner membrane protease subunit 2	Q8BPT6
KSLTNDWEDH	11		770.2	Heat shock protein HSP 90-beta	P11499
GAVTDDEVI	9		770.2	THO complex subunit 7 homolog	Q7TMY4; Q7TMY4-2
SAISSSDLF	9		771.2	ADP-ribosylation factor GTPase-activating protein 2	Q99K28-2; Q99K28
VCVDNSEYM	9		772.2	26S proteasome non-ATPase regulatory subunit 4	O35226-3; O35226; O35226-4; O35226-2; O35226-5
FQVTHTVAL	9		773	Mitochondrial import receptor subunit TOM40 homolog	Q9QYA2
FSARSSLYI	9		775.6	Zinc finger protein ZXDC	Q8C8V1-3; Q8C8V1; Q8C8V1-2; A2CE44
SLVNQEVL	8		777.1	DNA-directed RNA polymerase I subunit RPA2	P70700
QIIINTTHL	9		777.3	Exocyst complex component 6	Q8R313; A6H5Z3; A6H5Z3-2
LCVSNRIAI	9		778.8	Inositol 1,4,5-trisphosphate receptor type 3	P70227
RCIQNMPKTL	10		782.1	Tumor necrosis factor ligand superfamily member 13B	Q9WU72-2; Q9WU72
KAPFSVEFV	9		783.8	Mannosyl-oligosaccharide glucosidase	Q80UM7
VCLWNKDQI	9		784.4	Shugoshin 1 OS=Mus musculus OX=10090	Q9CXH7
SSPRHKEWL	9		786.9	Vacuolar protein sorting-associated protein 18 homolog	Q8R307
YRSVTHFDSL	10		788.8	Long-chain-fatty-acid--CoA ligase 4	Q9QUJ7; Q9QUJ7-2
TMETNREEL	9		789.6	Isoform 2 of Patched domain-containing protein 1	Q14B62-2; Q14B62
AQGINVNLV	9		790.2	Ankyrin repeat and SOCS box protein 11	Q9CQ31; Q9CQ31-2
SSLKDNTAL	9		791.4	Dynactin subunit 2	Q99KJ8
RGVKNRPEEF	11		792.7	sorting nexin-7	Q9CY18
AALKLGGQEL	9		794.5	protein flightless-1 homolog	Q9JJ28
SSLTDSSSL	9		795.1	SLIT-ROBO Rho GTPase-activating protein 2	Q91Z67
TSPINPYKDHN	11		798.1	zinc finger E-box-binding homeobox 2	Q9R0G7
QKVKNTEYL	9		798.1	Sorting nexin-14	Q8BHY8
TMMELRCVI	9	xCarbamidomethyl [C7	798.5	Inositol hexakisphosphate and diphosphoinositol-pentakisphosphate kinase 1	A2ARP1-2; A2ARP1-3; A2ARP1-1; Q6ZQB6; A2ARP1-7; Q6ZQB6-2; Q6ZQB6-3; A2ARP1-6
FSYKIDFAEM	10		800.4	Poly [ADP-ribose] polymerase 11	Q8CFF0-1; Q8CFF0-3
HALENLSSRHI	11		801.2	Protein SLX4IP	Q9D7Y9-2; Q9D7Y9
AAVGNHVAKL	10		803.4	NEDD8-activating enzyme E1 regulatory subunit	Q8VBW6
SGIQNVSTC	9		803.4	Scavenger receptor class B member 1	Q61009; Q61009-2

YCIENAHGQL	10	805.9	Protein TSSC1	Q8K0G5
SQGENLEGKY	11	806.6	Pogo transposable element with ZNF domain	Q8BZH4
SAPAPSSAPL	10	807.7	RNA polymerase II subunit A C-terminal domain phosphatase	Q7TSG2
NSMVLFDHV	9	808.3	DNA topoisomerase 2-alpha	Q01320
KTVSNDSFF	9	810.3	Nucleosome assembly protein 1-like 1	P28656
FAHTNIESLVK	11	815.2	Protein disulfide-isomerase A3	P27773
SSVSNPRNWL	11	818.7	Tumor necrosis factor receptor superfamily member 23	Q9ER63
RAVANETGAF	11	824.2	Transitional endoplasmic reticulum ATPase	Q01853
NTIANKLHL	9	828.1	Eukaryotic translation initiation factor 2A	Q8BJW6-2; Q8BJW6-1
FVQTNISHL	9	831.9	Fms-related tyrosine kinase 3 ligand	P49772; P49772-4; P49772-2; P49772-3
HQQQNLDTF	9	833.6	E3 ubiquitin-protein ligase RNF213	E9Q555
FTIENKTPQ	9	833.6	Staphylococcal nuclease domain-containing protein 1	Q78PY7
LSALNPELV	9	837.2	Hepatocyte growth factor receptor	P16056
TQGQNIQHL	9	839.4	Myomegalin	Q80YT7; Q80YT7-2
ASYCGHTEI	9	843.1	Ankyrin repeat domain-containing protein 39	Q9D2X0
AALENTHLLEV	14	844.7	Cytosolic iron-sulfur assembly component 2B	Q9D187
KQARNSQFDF	11	845.8	Splicing factor, suppressor of white-apricot homolog	Q3USH5
SSVPGVTVI	9	849.1	tRNA wybutosine-synthesizing protein 4	Q8BYR1-2; Q8BYR1
SCLVMTTEI	9	854.1	Exosome RNA helicase MTR4	Q9CZU3
NQFVNKFNV	9	859.8	COP9 signalosome complex subunit 6	O88545
SSVRNVSTC	9	862	Homeodomain-interacting protein kinase 2	Q9QZR5
AAIIDHERI	9	864.4	Serine/threonine-protein kinase MRCK alpha	Q3UU96-2; Q3UU96-1
GAIRNISGTLP	11	868.6	CSC1-like protein 1	Q91YT8
VAPENLPALLL	11	869.7	ATP-binding cassette sub-family A member 7	Q91V24
SNVTNATNF	9	873	Vacuolar protein sorting-associated protein 13B	Q80TY5
FCAVNPKFL	9	876.4	Coronin-1B	Q920M5-1; Q9WUM3
NQLSNHISSL	10	876.6	Glucose-6-phosphate 1-dehydrogenase 2	P97324; Q00612
FSPRAVDYV	9	876.9	Isoform 2 of Methyltransferase-like protein 6	Q8BVH9-2; Q8BVH9-1
SQLIDTHLL	9	878.8	Protein PRRC2C	Q3TLH4; Q3TLH4-5
AQLSHIEHM	9	882.7	Guanine nucleotide exchange protein SMCR8	Q3UMB5-2; Q3UMB5
SLENIATI	8	887	Protein regulator of cytokinesis 1	Q9D9H8; Q99K43
RAPAGIDTPL	10	889.7	39S ribosomal protein L41, mitochondrial	Q9CQN7
DGGLRNVDCL	10	891	Serine/threonine-protein kinase TBK1	Q9WUN2
STLVHKFFL	9	892.7	Poly(A) polymerase gamma	Q61183-2; Q61183-3; Q6PCL9; Q61183-1; Q61183-4
QGPGNRPPL	9	892.7	Lymphocyte cytosolic protein 2	Q60787
AIINPVEI	8	893.4	Protein aurora borealis	Q8BS90; Q8BS90-2
ASVYNTRHV	9	896	Probable G-protein coupled receptor 146	Q99LE2
QSDINFDVL	9	897	ATP-binding cassette sub-family A member 5	Q8K448
GCISNTASYL	10	902.4	V-type proton ATPase 116 kDa subunit a isoform 1	Q9Z1G4-2; Q9Z1G4-3; Q9Z1G4; P15920; P15920-2; Q920R6
KCLENLVQL	9	906.2	Rotatin	Q8R4Y8
FQYESKVFYL	10	908.3	14-3-3 protein eta	P68510
IAPEDQVVL	9	910.2	Ubiquitin-like protein FUBI	P35545
QTVENVEHL	9	911.3	TELO2-interacting protein 1 homolog	Q91V83
FAGHSGDVM	9	913.5	Guanine nucleotide-binding protein G(I)/G(S)/G(T) subunit beta-2	P62880
HGVSNLQEF	9	916.9	Adiponectin receptor protein 2	Q8BQS5
RAINNEMAHQ	11	917.7	Engulfment and cell motility protein 1	Q8BPU7-3; Q8BPU7-1
TGPENPWLV	9	920.8	Endoplasmic reticulum metallopeptidase 1	Q3UVK0
SRFKNRETI	9	929.2	DNA-directed RNA polymerase II subunit RPB4	Q9D7M8
RSLESRDHL	9	929.7	Coiled-coil domain-containing protein 88B	Q4QRL3; Q4QRL3-2
STLKPNVHHL	10	938.1	Methylcytosine dioxygenase TET2	Q4JK59; Q4JK59-3; Q4JK59-2
RAEDNADTL	9	939.8	proliferating cell nuclear antigen	P17918

RCQSNLDTL	9	941.1	ral transcription and DNA repair factor IIH helicase subunit XPD OS=Mus musculus OX=1	O08811
WQNENVILL	9	941.3	Deoxyribonuclease-1-like 1	Q9D7J6
SVVANGVCHL	10	942.2	Monocarboxylate transporter 1	P53986
HGPQNHEAHL	10	951.3	Cytosolic 10-formyltetrahydrofolate dehydrogenase	Q8R0Y6
HQVRSMDEL	9	953.2	Pumilio homolog 1	Q80U78-4; Q80U78-2; Q80U78; Q80U78-3
SGPSNLGKFT	14	953.7	Isoform 2 of Serine-rich coiled-coil domain-containing protein 2	Q3UHI0-2; Q3UHI0
VAPTSTVAV	9	954.7	Myc-associated zinc finger protein	P56671
RTPENHENL	9	955.4	Lysine-specific demethylase 3B	Q6ZPY7; Q6ZPY7-2
VAPTNPRFGG	13	957.1	DNA replication licensing factor MCM6	P97311
LCALNVVYM	9	959.7	Tetraspanin-31	Q9CQ88
SALGSQTHL	9	962.3	TLE family member 5	P63002-2; P63002
IAIVNHDKC	9	967.1	ATP-binding cassette sub-family E member 1	P61222
GSPENLGEST	11	971	Nuclear receptor coactivator 7	Q6DFV7-1
SCFQNQRAI	9	974.5	lated matrix-associated actin-dependent regulator of chromatin subfamily A containing DE	Q04692
SGPKDVTVL	9	978.8	Zinc finger protein 638	Q61464-4; Q61464
AAPINPSDINM	11	980.4	Enoyl-[acyl-carrier-protein] reductase, mitochondrial	Q9DCS3
SALGSTGPL	9	980.6	Ubiquitin-associated protein 2	Q91VX2
SQMEHAMETM	10	983.2	Protein S100-A10	P08207
FAGRSFRNPL	10	992.7	Alpha-enolase	P17182
HSHLLKTPL	9	993.9	Ribonucleoprotein PTB-binding 1	Q9CW46
RGLDNREEV	9	996.2	Pseudopodium-enriched atypical kinase 1	Q69Z38
SCITNGSGI	9	997.8	Echinoderm microtubule-associated protein-like 4	Q3UMY5-2; Q3UMY5-4; Q3UMY5; Q3UMY5-3
FVGINASDI	9	998.3	Prostaglandin reductase-3	Q8BGC4
WCPRNPAVL	9	999	Protein transport protein Sec31A	Q3UPL0
SACHNDSVFL	10	1001	Protein CIP2A	Q8BWY9
RCPDNSDPL	9	1001.1	Unconventional myosin-IXb	Q9QY06-3; Q9QY06-2; Q9QY06
SAMRNLNGRE	11	1001.6	cleavage stimulation factor subunit 2	Q8BIQ5-2; Q8BIQ5-1; Q8C7E9
AAISLATRV	9	1003.6	ATP-dependent RNA helicase DHX33	Q80VY9
VSVGNVGQL	9	1003.7	Proteasome assembly chaperone 2	Q9EST4-2; Q9EST4
TIYQNAGPTM	10	1003.9	Signal transducing adapter molecule 1	P70297
SSPYHVDLL	9	1007.3	Mitotic checkpoint serine/threonine-protein kinase BUB1 beta	Q9Z1S0
SQVPNYTLT	9	1008.7	Signal transducing adapter molecule 1	P70297
SSTTNPKLSTL	11	1009.6	Eukaryotic translation initiation factor 4E transporter	Q9EST3; Q9EST3-2
STLVLHDLL	9	1014.1	Breakpoint cluster region protein	Q6PAJ1; Q5SSL4; Q5SSL4-2; Q5SSL4-4; Q5SSL4-3
TTFQNDFESL	10	1019.3	Choline kinase alpha	O54804-2; O54804
RTLTGQDTM	9	1027.4	BTB/POZ domain-containing adapter for CUL3-mediated RhoA degradation protein 1	Q8BGV7
VQHASVQYI	9	1037.9	Lysosomal alpha-mannosidase	O09159
VRVTNRDII	9	1039.3	60S ribosomal protein L5	P47962
RTLSNSEENPI	11	1045.8	Rotatin	Q8R4Y8
RSITNTTVCT	10	1053.6	Splicing factor 1	Q64213
LQVQNPQAQT	11	1055.2	Transcription factor Sp1	O89090-2; O89090
SSYQHTSVTL	10	1059.6	Exportin-T	Q9CRT8
SAVKNMNLP	9	1062.4	AP-3 complex subunit sigma-1	Q9DCR2
WIVNREYL	8	1062.7	Ribosomal protein S6 kinase alpha-2	Q9WUT3
SACQNSVHPA	10	1087.5	Ankyrin repeat domain-containing protein 17	Q99NH0
KAPANRELDC	11	1089.7	BCLAF1 and THRAP3 family member 3 OS=Mus musculus OX=10090	A2AG58
FNKLNLEHI	9	1092.2	Vacuolar protein sorting-associated protein 35	Q9EQH3
IQGQNKEML	9	1092.5	Flotillin-1	O08917
CGGYNGEVI	9	1095.1	Kelch domain-containing protein 10	Q6PAR0-3; Q6PAR0-2; Q6PAR0
AALRNGLEG	9	1097.7	Nuclear pore complex protein Nup98-Nup96	Q6PFD9
TQCLNESTYI	10	1103.3	Helicase with zinc finger domain 2	E9QAM5
QGITNLKLVTL	11	1110.8	Kinetochore-associated protein 1	Q8C3Y4

SAVKLRWLL	9	1111.5	Isoform 2 of Glycerol kinase	Q64516-2; Q64516-1; Q64516-3
YQYALEHMI	9	1111.5	Kinetochore-associated protein 1	Q8C3Y4
RTVYNREKL	9	1111.7	Metal transporter CNNM4	Q69ZF7
KQTINPILL	9	1114.3	long-chain-fatty-acid--CoA ligase 3	Q9CZW4
AAVRNCDGF	9	1118.5	Iron-responsive element-binding protein 2	Q811J3
SAPSNFEHRV	11	1128.8	Serine/threonine-protein kinase PAK 4	Q8BTW9
AALENDKTIKL	11	1129.6	WD repeat-containing protein 5	P61965
SCLYNPDDL	9	1129.7	Proto-oncogene c-Rel	P15307
AAITNKYQLVF	12	1130.6	RNA polymerase I-specific transcription initiation factor RRN3	B2RS91
KVVINGQEV	9	1131.6	Leukotriene A-4 hydrolase	P24527
SSMSGYDQVL	10	1132.3	Heterogeneous nuclear ribonucleoprotein H2	P70333; O35737
SQLSDWQHL	9	1132.6	SUN domain-containing protein 1	Q9D666; Q9D666-3; Q9D666-4; Q9D666-2; Q9D666-5
LQYPNPLPTL	10	1136.3	Vam6/Vps39-like protein	Q8R5L3-2; Q8R5L3
QAVWNVDYC	9	1137.1	Cyclin-Y	Q8BGU5; Q8BGU5-2
DGVANVEHI	9	1138.8	Cytosolic 5'-nucleotidase 3A	Q9D020; Q9D020-1
VPPVNGVEI	9	1144.1	Protein PRRC2B	Q7TPM1-2; Q7TPM1-1
VAPGMMKEFM	10	1144.2	Brefeldin A-inhibited guanine nucleotide-exchange protein 3	Q3UGY8
FMKNFLEM	8	1145.2	Phosphoserine aminotransferase	Q99K85
AAPAPSLWTL	10	1149.9	Protein shisa-5	Q9D7I0; Q9D7I0-2
SIHGTNPQYL	10	1153.1	Pre-mRNA-splicing factor 38A	Q4FK66-1
SVAVNISNL	9	1158.4	Deubiquitinase OTUD6B	Q8K2H2
NSGLNFKDVL	10	1160.6	AT-rich interactive domain-containing protein 5B	Q8BM75; Q8BM75-3
SSLSNHKYVP	10	1161.2	Citramalyl-CoA lyase, mitochondrial	Q8R4N0
RAIKNDSVVA	10	1162.7	T-complex protein 1 subunit eta	P80313
CMWRNYEYC	9	1168.1	/SNF-related matrix-associated actin-dependent regulator of chromatin subfamily A memb	Q91ZW3
ACGANREEI	9	1173.1	TBC1 domain family member 8B	A3KGB4
LQVSNVLSQPI	11	1175.9	Dolichyl-diphosphooligosaccharide--protein glycosyltransferase subunit 2	Q9DBG6
FQGTSLTHL	9	1177.6	Negative regulator of reactive oxygen species	Q8BMT4; Q8BMT4-2; Q8BMT4-3
RSLQGKTEL	9	1177.7	Dihydroorotate dehydrogenase (quinone), mitochondrial	O35435
ACIVNTENKI	10	1183.9	Deoxycytidylate deaminase	Q8K2D6
RCIANPVKL	9	1191.3	Isoaspartyl peptidase/L-asparaginase	Q8C0M9
YQQTNEEAF	9	1192.7	Natural resistance-associated macrophage protein 1	P41251
SVCVNSSCI	9	1197.7	ERI1 exoribonuclease 2	Q5BKS4
VAPPSFVAI	9	1201.3	Transmembrane protein 41B	Q8K1A5; Q8K1A5-2; Q8K1A5-3
GSLLNQPKAVI	11	1203.9	Claspin	Q80YR7
GMHVNGAPP	9	1204.6	Cleavage stimulation factor subunit 2	Q8BIQ5-2; Q8BIQ5
SVLSNVEVT	9	1206.4	Cytoplasmic dynein 1 heavy chain 1	Q9JHU4
YAAAALPTL	9	1216.9	CUGBP Elav-like family member 2	Q9Z0H4-1; P28659-2; Q9Z0H4-7; Q9Z0H4-11; Q9Z0H4-9; P28659-1; Q9Z0H4-4; Q9Z0H4-8; P28659-3; P28659-4; Q9Z0H4-6; Q9Z0H4-2; Q9Z0H4-3; Q9Z0H4-5
THLQNQEKL	9	1218.3	Pericentrin	P48725; P48725-3; P48725-2
GVVRNNEAL	9	1222.4	Integrin alpha-V	P43406
RVYENVGLM	9	1223.1	tyrosine-protein phosphatase non-receptor type 11	P35235; P35235-2
SSLLDSAHL	9	1223.8	AP-3 complex subunit delta-1	O54774
RGLRNLINMEM	11	1223.8	schlafen family member 5	Q8CBA2
SAPNSPMAM	9	1227.3	transcription factor EB	Q9R210; Q08874; Q08874-9; Q08874-4; Q08874-1; Q08874-4; Q08874-8
SAPSAGSLYM	10	1229.3	BAG family molecular chaperone regulator 4	Q8CI61
RQLLLCETL	9	1236	Rho guanine nucleotide exchange factor 10-like protein	A2AWP8-1
AGLRLLTNM	9	1243.3	Armadillo repeat-containing X-linked protein 3	Q8BHS6; Q9CX83; Q9D0L7; Q9D0L7-2
YALDLIDKL	9	1244.3	Cyclin-dependent kinase 9	Q99J95-3; Q99J95; Q99J95-2
FCISNPVLA	9	1246	Cerebral dopamine neurotrophic factor	Q8CC36

VQVTGGQYL	9		1246.9	ATP-dependent DNA helicase PIF1	Q80SX8; Q80SX8-3
SIVFNGPHL	9		1253.8	Histone-lysine N-methyltransferase SMYD3	Q9CWR2
ISLMLTTKL	9		1255.6	Cytochrome c oxidase subunit 2	P00405
FIHNNLKDVYI	11		1256.9	SH3 and PX domain-containing protein 2A	O89032-1; O89032-3; O89032-2
SAPLSPLKFM	10		1263.5	Gamma-adducin	Q9QYB5-2; Q9QYB5-1
SAIQDYLQQL	10		1265.1	Glutaredoxin-1	Q9QUH0
FGLVNQERT	9		1266.3	NACHT, LRR and PYD domains-containing protein 3	Q8R4B8-4; Q8R4B8-2; Q8R4B8-3; Q8R4B8-1
FAHTNIESLVK	12		1267.5	Protein disulfide-isomerase A3	P27773
CSPQNKISF	9		1271.1	Signal peptide peptidase-like 3 OS=Mus musculus OX=10090	Q9CUS9
RAGPRLIVYI	10		1273.4	Syntaxin-binding protein 2	Q64324
AGIENKFGL	9		1279.2	Calcium-binding mitochondrial carrier protein Aralar1	Q8BH59; Q9QXX4
SAILDHPPSPM	11		1280.6	MKL/myocardin-like protein 1	Q8K4J6-2; Q8K4J6-1
SAPTDNNLCH	11		1284.1	Pyrroline-5-carboxylate reductase 3	Q9DCC4
FCERNRDVL	9		1290	Unconventional myosin-Ie	E9Q634; P70248
NGCINYEEL	9		1294.2	Isoform Smooth muscle of Myosin light polypeptide 6	Q60605-2
MMGYNTDRL	9		1295	anaphase-promoting complex subunit 1	P53995
FSKESDDPM	9		1296.9	Endoplasmin	P08113
QGPFNNQEM	9		1298.1	GRB10-interacting GYF protein 2	Q6Y7W8
SAQRNHAIRI	10		1303	cold shock domain-containing protein E1	Q91W50
SLGLNPYPGI	10		1303.5	Macrophage colony-stimulating factor 1 receptor	P09581
GTIVNGKQI	9		1305	Angiogenic factor with G patch and FHA domains 1	Q7TN31
QALRDNSTM	9		1314.4	Heat shock protein HSP 90-beta	P07901; P11499
AAAKNLSDMT	11	1xOxidation [M9]	1317.5	T-complex protein 11-like protein 2	Q8K1H7
VSPRGAGYL	9		1322.2	Serine/threonine-protein phosphatase 4 catalytic subunit	P97470
VTCSNVEVL	9		1323	Leucine-rich repeat serine/threonine-protein kinase 2	Q5S006
RQPQNQQKV	9		1325.2	cationic amino acid transporter 2	P18581-2; P18581-1
SSPRSDVPVM	10		1326.5	U3 small nucleolar RNA-associated protein 14 homolog A	Q640M1
RTVKNIADL	9		1328.3	Neuron navigator 3	Q80TN7
SMATNTGGLS	12		1333	Nuclear receptor coactivator 2	Q61026
VCIENTETLV	10		1339.2	Kinesin-like protein KIF20A	P97329
SALTGGTAHL	10		1344.3	neuroguidin	Q9DB96
ICLGNTYHL	9		1347.7	Queuine tRNA-ribosyltransferase catalytic subunit 1	Q9JMA2
RSLLNSHILHL	11		1350.3	WD repeat and FYVE domain-containing protein 3	Q6VNB8
IGGENGPVL	9		1352.3	U5 small nuclear ribonucleoprotein 200 kDa helicase	Q6P4T2
ACLVNLRSL	9		1353.1	DNA polymerase delta subunit 2 OS=Mus musculus OX=10090	O35654
SQLISLQHL	9		1355	Probable ATP-dependent RNA helicase DDX28	Q9CWT6
TILTNHQHL	9		1361.6	Protection of telomeres protein 1	Q91WC1-2; Q91WC1
MPWNVDTL	8		1362.5	Hsp90 co-chaperone Cdc37	Q61081
SQLEKLDLL	9		1363.8	Centrosomal protein of 57 kDa	Q8CEE0-1; Q8CEE0-3; Q8CEE0-2
KCIQNEAGDW	11		1379.9	SP110 nuclear body protein	Q8BVK9
GCLTNGHIYV	10		1380.3	N-acylneuraminate cytidylyltransferase	Q99KK2-2; Q99KK2
SRVTNGELL	9		1385	Isoform 3 of Endophilin-B2	Q8R3V5-3; Q8R3V5-1; Q8R3V5-4
VAISPGKHYL	10		1386.8	poly [ADP-ribose] polymerase 14	Q2EMV9
SQPIKADVL	9		1389.2	Protein SERAC1	Q3U213-2; Q3U213
YGGRHTVTM	9		1389.4	Isocitrate dehydrogenase [NAD] subunit gamma 1, mitochondrial	P70404
FTHQNGERV	9		1390.5	Cytoplasmic polyadenylation element-binding protein 4	Q7TN98-5; Q7TN98-1; Q7TN98-2; Q7TN98-3; Q7TN98-4
SSGINVNAA	9		1409.9	Caprin-1	Q60865
SVLRLVDAL	9		1412.7	Histone deacetylase 6	Q9Z2V5
YQLKLAEKL	9		1416.7	Nck-associated protein 1-like	Q8K1X4
STLRNIQGL	9		1429.5	Proteasome maturation protein	Q9CQT5
GIENIHYL	8		1431.2	malignant T-cell-amplified sequence 2	Q9DB27-2; Q9CQ21; Q9DB27
SLGINPHVL	9		1433.6	Nuclear pore complex protein Nup98-Nup96	Q6PFD9

FCPTNCHVNL	10	1439.4	Phosphatidylinositol 3,4,5-trisphosphate-dependent Rac exchanger 1 protein	Q69ZK0
FMVEKGPTL	9	1447	Ubiquitin-conjugating enzyme E2 J2	Q6P073
SSVKNEEQFV	11	1448	Maternal embryonic leucine zipper kinase	Q61846
ASHLNLAMCH	11	1451.8	Peptidyl-prolyl cis-trans isomerase FKBP4 OS=Mus musculus OX=10090	P30416
SSHYSVNTL	9	1455.3	Proto-oncogene c-Rel	P15307
VAPAAHTHI	9	1459.3	Arginine-glutamic acid dipeptide repeats protein	Q80TZ9
THICLLDTI	9	1459.9	2'-5'-oligoadenylate synthase-like protein 1	Q8VI94
SIKNVTEL	8	1461.3	Replication termination factor 2	Q99K95
CVGENFAYV	9	1464.6	Lanosterol 14-alpha demethylase	Q8K0C4
SGCKNGQVL	9	1467.9	Notchless protein homolog 1 OS=Mus musculus OX=10090	Q8VEJ4
YPTNQAVI	8	1468.6	Large neutral amino acids transporter small subunit 2	Q9QXW9
RHLENVMHI	9	1470.7	Protocadherin Fat 3	Q8BNA6
YAPKGSSVV	9	1472.2	Sphingosine-1-phosphate lyase 1	Q8R0X7
SNVKNEEKL	9	1474.3	SLAIN motif-containing protein 2	Q8CI08-2; Q8CI08
AANTLREHL	9	1474.8	Origin recognition complex subunit 3	Q9JK30-2; Q9JK30
VQVPNLESLTI	11	1478.2	Chromodomain-helicase-dna-binding protein 8	Q09XV5
AGIENKFGLYL	12	1480.3	Calcium-binding mitochondrial carrier protein Aralar1	Q8BH59; Q9QXX4
FQVQDKEVL	9	1487.6	Rab3 GTPase-activating protein non-catalytic subunit	Q8BMG7-2; Q8BMG7
TIRNADVI	8	1489.8	Phosphatidylcholine translocator ABCB4	P21440
AGVRNPQQHL	10	1499.5	Polyadenylate-binding protein 1	P29341
AAIISTSTA	9	1499.8	Biorientation of chromosomes in cell division protein 1-like 1	E9Q6J5; E9Q6J5-3; E9Q6J5-2
ACVANDDVF	9	1503.2	Isoform B of Calcipressin-1	Q9JHG6-2
AMVQHPQPTF	11	1505.1	Nuclear receptor coactivator 3	O09000
VGVVNPILA	9	1511.9	Poly(U)-binding-splicing factor PUF60	Q3UEB3-2; Q3UEB3; Q3UEB3-3
SGIDNPGSL	9	1512.1	Sodium- and chloride-dependent taurine transporter	O35316
YAPAPFLHI	9	1513.7	Ubiquitin-associated protein 2	Q91VX2
SVVINAELA	9	1521.6	Baculoviral IAP repeat-containing protein 6	O88738-2; O88738; O88738-3
FQWSEQRDYI	10	1523	surfeit locus protein 4	Q64310
SCLKMLDEM	9	1533	COP9 signalosome complex subunit 1	Q99LD4
DALSLRDTL	9	1536.2	Cytoplasmic dynein 1 light intermediate chain 1	Q8R1Q8
YTGPSHHSYM	10	1539.8	Histone acetyltransferase KAT6A	Q8BZ21
VCPSNKPAI	9	1551.4	Junction plakoglobin	Q02257
SAPSNPWGGI	10	1554.7	Cytoplasmic polyadenylation element-binding protein 3	Q7TN99-5; Q7TN99-2; Q7TN99-4; Q7TN99-6; Q7TN99-3; Q7TN99
SCQLNGATL	9	1557.7	Intersectin-2	Q9Z0R6
NSLNNNFLQTI	11	1561.5	Cullin-3	Q9JLV5
SPENEEYI	8	1562.8	Regulatory-associated protein of mTOR	Q8K4Q0-2; Q8K4Q0
SQPNNLADVG	13	1568.7	Arf-GAP domain and FG repeat-containing protein 2	Q80WC7
SSPSNKFFFH	10	1572	Histone-lysine N-methyltransferase SETD2	E9Q5F9-2; E9Q5F9
FCFSNEFSTF	10	1584.2	Transmembrane emp24 domain-containing protein 3	Q78IS1
ASVTGHTNI	9	1585.2	Protein fem-1 homolog A-A	Q9Z2G1; Q8CEF1
FAPSKGSVTL	10	1590.6	Anillin	Q8K298
GCLENLENL	9	1597.6	CD180 antigen	Q62192
KVAVNGQHM	9	1601	Galectin-9	O08573-2; O08573; O08573-3
KSLLGKDVL	9	1608	phosphoglycerate kinase 1	P09411
RLNLNNTVL	9	1613.3	Bifunctional glutamate/proline--tRNA ligase	Q8CGC7
SSGFNPEGIY\	11	1614.5	Ras association domain-containing protein 8	Q8CJ96
SALQHPQLCTI	11	1615.8	ADP-ribosylation factor-like protein 15	Q8BGR6
TSPVHTELL	9	1617.6	Mediator of DNA damage checkpoint protein 1	Q5PSV9
SSLMKVENM	9	1618.5	Coiled-coil domain-containing protein 25	Q78PG9
QCLLNVEHL	9	1619.3	staphylococcal nuclease domain-containing protein 1	Q78PY7
SSPRATETV	9	1626.9	Carcinoembryonic antigen-related cell adhesion molecule 1	P31809; Q925P2-2; Q925P2; P31809-3

AIENIEHL	8	1628.7	Leucine-rich PPR motif-containing protein, mitochondrial	Q6PB66
GGPINPATA	9	1634.7	Ubiquitin-associated protein 2-like	Q80X50-4; Q80X50-3; Q80X50-5; Q80X50
SQVELREQI	9	1638.6	SNARE-associated protein Snapin	Q9Z266
KALINPANVTF	11	1645	Acyl-protein thioesterase 1	P97823; P97823-2
SQVIQGEFL	9	1656.9	NADH dehydrogenase [ubiquinone] 1 beta subcomplex subunit 2, mitochondrial	Q9CPU2
THLLNSEHL	9	1657.5	PHD finger protein 12	Q5SPL2-2; Q5SPL2
SAPANPSSNIN	13	1659.1	SLAIN motif-containing protein 2	Q8CI08-2; Q8CI08
NAPRLIRPYM	10	1663.8	Serine/threonine-protein kinase mTOR	Q9JLN9
RGPENPKLEM	10	1671	probable ATP-dependent RNA helicase DHX58	Q99J87
FGGANHGGY	11	1675.8	Interleukin enhancer-binding factor 3	Q9Z1X4-2; Q9Z1X4; Q9Z1X4-3
RMPEKVTWM	9	1676.7	60S ribosomal protein L19	P84099
TSVRNKPDST	11	1678.3	protein TALPID3	E9PV87
FHFTNRDCDS	11	1683.1	Prostate tumor-overexpressed gene 1 protein homolog	Q91VU8
IKNENQEVI	9	1684.2	cGMP-dependent 3',5'-cyclic phosphodiesterase	Q922S4-3; Q922S4; Q922S4-2
YAPKGSSVVM	10	1686.8	Sphingosine-1-phosphate lyase 1	Q8R0X7
VSGISRDVI	9	1694	poly [ADP-ribose] polymerase 14	Q2EMV9; Q2EMV9-2
SAVHNDQFGL	13	1696.6	Uncharacterized protein C15orf61 homolog	Q0VG49-2; Q0VG49
GNYNNQSSNF	13	1698.7	Heterogeneous nuclear ribonucleoprotein A1	P49312
SAVISLEGKPL	11	1699.1	Cofilin-1	P18760
HLHENTELM	9	1708	AP-4 complex subunit epsilon-1	Q80V94
TCLENGSFLL	10	1709.5	Protein Churchill	Q6DG52-2; Q6DG52
AIRNDEEL	8	1710.1	Histone H2A type 2-C	Q64523; Q8BFU2; C0HKE4; Q8CGP5; Q8R1M2; P27661; Q8CGP7; Q8CGP6; Q6GSS7
SAYNNLKGNL	13	1718.5	V-type proton ATPase subunit C 1	Q9Z1G3
FSPQMCEHL	9	1720.9	V-type proton ATPase subunit H	Q8BVE3
SGPTSLFAV	9	1721.3	Heterogeneous nuclear ribonucleoprotein U	Q8VEK3-2; Q8VEK3
FCYDNPAAL	9	1722	Major facilitator superfamily domain-containing protein 1	Q9DC37
NQAINKFYF	9	1728.2	Centromere protein U	Q8C4M7-2; Q8C4M7
AIKNHEEL	8	1738.2	45 kDa calcium-binding protein	Q61112; Q61112-2
SFLKLYTTM	9	1741.2	eukaryotic translation initiation factor 3 subunit L	Q8QZY1
SAPFPAPDTI	10	1745.6	Band 4.1-like protein 5	Q8BGS1; Q8BGS1-3
RSLGNDVKSY	11	1746.6	Protein Njmu-R1	Q9CYI0
SALRSLSNATI	11	1752	signal recognition particle 54 kDa protein	P14576-2; P14576-1
FAPPHFPVCM	10	1764.8	Solute carrier family 12 member 6	Q924N4
RALDNRRIGQI	12	1766.5	Phosphatidylinositol 4,5-bisphosphate 3-kinase catalytic subunit beta isoform	Q8BTI9
SALILHQRI	9	1766.7	Zinc finger protein 24	Q91VN1; Q8BLB0
KSISNPPGSNL	11	1771.4	Serine/threonine-protein kinase WNK1	P83741-2; P83741; P83741-3; P83741-5; P83741-4
YAGSNFPEHIF	12	1772.5	Actin-related protein 2	P61161
HCLSLRTYL	9	1775.6	Bifunctional glutamate/proline--tRNA ligase	Q8CGC7
SPINVTTV	8	1776.7	Wings apart-like protein homolog	Q65Z40
KRVVNQEEL	9	1780.5	zinc finger protein 830	Q8R1N0
SQLEKTFAM	9	1781.6	Hermansky-Pudlak syndrome 5 protein homolog	P59438; P59438-2
KAVENGEEHT	11	1792.2	ankyrin repeat and LEM domain-containing protein 2	Q6P1H6-1; Q6P1H6-4; Q6P1H6-3; Q6P1H6-2
RAVANRTDAC	11	1795.7	26S proteasome regulatory subunit 7	P46471
CNVDNKDFM	9	1796.9	Inner centromere protein	Q9WU62-1
TQLSKTEFL	9	1800.6	protein S100-A11	P50543
TGPVNVLTT	9	1820.1	Msx2-interacting protein	Q62504-3; Q62504; Q62504-2
FIHEVNPSSL	10	1820.8	Prosaposin	Q61207
SVMENSKVLG	13	1830.4	Talin-1	P26039
SQPIAQQPL	9	1833.9	Heterogeneous nuclear ribonucleoprotein Q	Q7TMK9
VHVINVDKV	9	1844.5	Geranylgeranyl transferase type-2 subunit beta	P53612
AALVSKAIDVL	11	1844.7	Protein SOGA1	E1U8D0

LSILNSNEHLL	11	1855.6	Centrosomal protein of 85 kDa	Q8BMK0
GPLHGLANQE	12	1855.7	Citrate synthase, mitochondrial	Q9CZU6
RTIVLQESI	9	1855.8	TGF-beta receptor type-1	Q64729; Q64729-2
FSGTNDPCA	9	1880.5	Macrophage migration inhibitory factor	P34884
FCAVNPRFV	9	1904.8	Coronin-1C	Q9WUM4
FAGSNVDLP	9	1914.6	Guanine deaminase	Q9R111
TPVINYDLL	9	1931.2	Isoform B of Calcipressin-1	Q9JHG6-3; Q9JHG2; Q9JHG6-2; Q9JHG6-4; Q9JHG6
NQYTLKDEI	9	1931.4	Calcium/calmodulin-dependent protein kinase kinase 2	Q8C078-2; Q8C078-1; Q8C078-5; Q8C078-4; Q8C078-3
SSPAKQELI	9	1932.8	Sodium- and chloride-dependent betaine transporter	P31651
SQFQQEFPSL	10	1934	Protein PRRC2C	Q3TLH4; Q3TLH4-5
KGGFNRPLDF	11	1938.7	U4/U6.U5 small nuclear ribonucleoprotein 27 kDa protein	Q8K194-1
SARNLEHL	8	1940.8	F-box only protein 38	Q8BMI0
TQSVNIEQL	9	1941.7	NF-kappa-B inhibitor zeta	Q9EST8-3; Q9EST8-2; Q9EST8
QTHENREHL	9	1944.4	Dual specificity protein kinase CLK3	O35492
AACMNQKHL	9	1956	AMP deaminase 3	O08739; Q3V1D3
IMGIGVDTI	9	1959.1	Nucleoporin NUP188 homolog	Q6ZQH8
AAPPSFGTL	9	1960.7	Apoptosis-stimulating of p53 protein 2	Q8CG79
SMASSLNLPVI	11	1964.8	B-cell lymphoma/leukemia 10	Q9Z0H7
FSFGSKNTL	9	1981.9	E3 SUMO-protein ligase RanBP2	Q9ERU9
KTAGNSEFL	9	1997.4	Eukaryotic translation initiation factor 4 gamma 2	Q62448; Q62448-2
VATVNKAGSE	11	2003.1	Microtubule-actin cross-linking factor 1	Q9QXZ0-3; Q9QXZ0-2; Q9QXZ0-4; Q9QXZ0
YQLIHQGTKM	10	2009.6	Glucose-6-phosphate isomerase	P06745
QSPENVPTDH	11	2018.3	Golgin subfamily A member 2	Q921M4-1
STVRNADVIAC	12	2021.5	Multidrug resistance protein 1B	P06795; P21447
SSLKDTERI	9	2036	MAX gene-associated protein	A2AWL7-3; A2AWL7
FVLVHPTTNL	10	2037.7	POU domain, class 2, transcription factor 1	P25425-7; P25425-9; P25425-8; P25425-3; P25425-6; P25425-12; P25425-2; P25425-11; P25425-5; P25425; P25425-4; P25425-10
AVGRNTLLI	9	2042.1	Dolichyl-diphosphooligosaccharide--protein glycosyltransferase 48 kDa subunit	O54734
SGVNGTHI	8	2055.7	SRSF protein kinase 1	O70551
SQPESKVFYL	10	2056.7	14-3-3 protein zeta/delta	P63101
SGIHLTIEM	9	2060.8	Plasma membrane calcium-transporting ATPase 1	G5E829
SAVKNINLP	9	2083.9	AP-3 complex subunit sigma-2	Q8BSZ2
ALHNSQFL	8	2084.8	E3 ubiquitin-protein ligase KCMF1	Q80UY2-2; Q80UY2
FMAEHLEETL	10	2089	Eukaryotic translation initiation factor 4E transporter	Q9EST3; Q9EST3-2
STIINEDASFF	11	2089.4	Cytoplasmic polyadenylation element-binding protein 4	Q7TN98-5; Q7TN98; Q7TN98-2; Q7TN98-3; Q7TN98-4
FVVPNQKEL	9	2090.3	Long-chain-fatty-acid--CoA ligase 3	Q9CZW4
NALLNAGESRI	11	2104.9	Serine/threonine-protein kinase ATR	Q9JKK8
GGIRNGSYDIC	14	2121.6	3-ketoacyl-CoA thiolase B, peroxisomal	Q8VCH0; Q921H8
KCLKNIHRI	9	2128.3	CASP8 and FADD-like apoptosis regulator OS=Mus musculus OX=10090	O35732
KTGYNVDLL	9	2136.3	Ras-related protein Rab-20	P35295
RTLSNSLQNVI	11	2141.5	Exocyst complex component 6B	A6H5Z3; A6H5Z3-2
MLDNYDTM	8	2141.7	Ribosomal biogenesis protein LAS1L	A2BE28-2; A2BE28
GSAVISLEGKF	12	2147.2	Cofilin-1	P18760
ACLINKPEL	9	2153.8	tRNA-dihydrouridine(20a/20b) synthase [NAD(P)+]-like	Q32M08
AMSENIWSTL	10	2161.5	Peroxisomal targeting signal 1 receptor	O09012-2; O09012
AQYGNILKHVM	11	2164	Nucleoporin NUP53	Q8R4R6
AQLDNEKTNFI	11	2165.5	Leucine-rich repeat flightless-interacting protein 1	Q3UZ39-2; Q3UZ39
TCVSNGPFIM	10	2185.9	Polypyrimidine tract-binding protein 1	P17225
CQLGNFSIHM	10	2188.2	Very-long-chain enoyl-CoA reductase	Q9CY27
VQAQLGLPPL	10	2192	Transmembrane protein 59	Q9QY73
FQEENTENL	9	2192.9	26S proteasome non-ATPase regulatory subunit 12	Q9D8W5

SVLRLIQVI	9		2193.4	Conserved oligomeric Golgi complex subunit 2	Q921L5
SAPTLEDHF	9		2196.1	microfibrillar-associated protein 1A	C0HKD8
SQLELKSLI	9		2201.3	Mitogen-activated protein kinase 6	Q61532
RSLRLVTTA	9		2208.5	SUN domain-containing protein 1	Q9D666; Q9D666-3; Q9D666-4; Q9D666-2; Q9D666-5
SSPASTPLSPI	11		2212.3	Ribosomal RNA processing protein 1 homolog B	Q91YK2
IGPSSIDLI	9		2213.9	Inactive rhomboid protein 2	Q80WQ6
CADGLIMDNG	13		2216	Otogelin-like protein	F7A4A7-2; F7A4A7
VGPEHGTEI	9		2221.2	Interferon-induced very large GTPase 1	Q80SU7
FTSSMRGMDT	11		2233.5	Arginine/serine-rich coiled-coil protein 2	A2RTL5
YRISMADYV	9		2233.9	ATP-dependent 6-phosphofructokinase, liver type	P12382
GACRNLWRSI	11		2238.8	Helicase with zinc finger domain 2	E9QAM5
LGAQNLSESPI	11		2259.1	Protein/nucleic acid deglycase DJ-1	Q99LX0
FSVEGQENL	9		2270.7	Cap-specific mRNA (nucleoside-2'-O-)-methyltransferase 1	Q9DBC3
SQPIGVTKI	9		2275.1	Cytosolic arginine sensor for mTORC1 subunit 2	Q8CAB8
IPPTNTVDFI	10		2280.5	Protein FAM72A	Q8BFZ8
YLMELMTPA	9	1xOxidation [M6]	2284.1	Glucosidase 2 subunit beta	O08795; O08795-2
RGFENVELGV	11		2284.6	Protein FAM177A1	Q8BR63
RALELEQEL	9		2288.1	Alanine aminotransferase 1	Q8QZR5
AAPETREHL	9		2296.9	Ras-related protein Rab-32	Q9CZE3
ISPKHFVHL	9		2297.2	Glucocorticoid modulatory element-binding protein 1	Q9JL60
AQPSNSLLGEI	11		2300.1	Ribonucleoprotein PTB-binding 1	Q9CW46
SLVTNMDKLHI	11		2300.4	Nck-associated protein 1-like	Q8K1X4
SCPLFKDYL	9		2314.4	Thiosulfate sulfurtransferase/rhodanese-like domain-containing protein 2	Q3U269
SCPAGYIPL	9		2314.6	Macrophage-expressed gene 1 protein	A1L314
AIFESPYPM	9		2315.8	Sarcoplasmic/endoplasmic reticulum calcium ATPase 2	O55143-2; O55143
VQPEHIQYL	9		2321.1	structural maintenance of chromosomes flexible hinge domain-containing protein 1	Q6P5D8
SSCMNQKHL	9		2329.8	AMP deaminase 2 OS=Mus musculus OX=10090	Q9DBT5
FGPVDSEQL	9		2333.8	Cyclin-dependent kinase inhibitor 1	P39689
NCILNAEALM	10		2343.8	Prolyl 3-hydroxylase 1 OS=Mus musculus OX=10090	Q3V1T4
KGPLNGDTDY	11		2358.4	Disabled homolog 2	P98078; P98078-2
SLGTNRDDL	9		2368.9	Kinesin-like protein KIF16B	B1AVY7
QCIANQVQL	9		2382.8	ATP synthase mitochondrial F1 complex assembly factor 1	Q811I0
SPVNPVAI	8		2383.3	RNA-binding protein 47	Q91WT8
STYSNRDIDGI	11		2394.4	Helicase with zinc finger domain 2	E9QAM5
KQVTSAEHL	9		2396.8	Origin recognition complex subunit 5	Q9WUV0
SSVKNVPENL	11		2398.3	Aminoacyl tRNA synthase complex-interacting multifunctional protein 2	Q8R010
QAFENGRALV	12		2417.6	GEM-interacting protein	Q6PGG2; Q6PGG2-2
TKCINGEYM	9		2430	Double-stranded RNA-specific editase 1	Q91ZS8-2; Q91ZS8; Q91ZS8-3; Q91ZS8-4; Q91ZS8-5; Q91ZS8-6
SSPTGSDWF	9		2431.8	Autophagy-related protein 13	Q91YI1-2; Q91YI1
YTIENPRHF	9		2444.1	Staphylococcal nuclease domain-containing protein 1	Q78PY7
VHPTNSALNYI	11		2457	Dual specificity protein phosphatase 1	P28563
VQPSHHSEI	9		2463.2	Protein ABHD11	Q8K4F5
RAIWNYIHC	9		2464	Sestrin-2	P58043; P58006
GLLENSAHL	9		2465.8	DNA repair and recombination protein RAD54B	Q6PFE3
YGNILKHVM	9		2468.2	Nucleoporin NUP53	Q8R4R6
SVMENSKVLG	13		2472.7	Talin-2	Q71LX4
SGVEGADVV	9		2475.5	Hexokinase-1	P17710; P17710-2; P17710-4; P17710-3
TIGSNVEEI	9		2477.6	ADP-ribosylation factor-like protein 5C	Q6P068; Q9D4P0; Q80ZU0
SAPANGILVPI	13		2483	Interferon regulatory factor 2-binding protein 2	E9Q1P8
YHIGTLQEYL	10		2493.9	fucose-1-phosphate guanylyltransferase	G5E8F4
THLINFSETL	10		2503.2	Cytoplasmic FMR1-interacting protein 1	Q7TMB8

AVIRNINDQVL	11	2517.3	Interleukin-18	P70380
TQYESKVFYL	10	2526.7	14-3-3 protein gamma	P61982
QCLENQQLI	9	2542.3	POC1 centriolar protein homolog A	Q8JZX3
SSNKNSLKDSI	11	2561.5	Sentrin-specific protease 1	P59110
RGPVNLQHLIL	11	2568	Leucine-rich repeat and fibronectin type-III domain-containing protein 4	Q80XU8
SALKSVTSA	9	2568.4	Nuclear fragile X mental retardation-interacting protein 2	Q5F2E7-2; Q5F2E7-1
GGPENTLVF	9	2573.6	KN motif and ankyrin repeat domain-containing protein 2	Q8BX02; Q8BX02-2
FSLKKATFA	9	2575.6	Protein FAM193A	Q8CGI1
FIRNQEQM	8	2576	26S proteasome regulatory subunit 4	P62192
ISNRNLTL	8	2581.6	MORC family CW-type zinc finger protein 1	Q9WVL5
AALQNANTKC	10	2583.8	e3 ubiquitin-protein ligase UBR4	A2AN08
YVNPNHQATL	10	2588.1	Brefeldin A-inhibited guanine nucleotide-exchange protein 2	A2A5R2
KLWENPNVL	9	2589.3	Intraflagellar transport protein 27 homolog	Q9D0P8
RQVKNPFGLE	11	2589.8	cell growth regulator with RING finger domain protein 1	Q8BMJ7
SALTHAGAHL	10	2597.9	Splicing factor 3A subunit 3	Q9D554
QPPYNPTYM	9	2601.9	Protein shisa-5	Q9D7I0; Q9D7I0-5; Q9D7I0-3; Q9D7I0-2
CAVENGGCSH	11	2603.2	Low-density lipoprotein receptor-related protein 4	Q8VI56
SCVSNIESA	9	2617.9	Copper-transporting ATPase 1	Q64430
AMGTSTVEI	9	2625	ESF1 homolog	Q3V1V3
KLLALKDFM	9	2628.5	Phosphoinositide 3-kinase regulatory subunit 4	Q8VD65
FTWTGAEHI	9	2633.7	E3 ubiquitin-protein ligase UBR5	Q80TP3
SMPRGVVVTL	10	2636.4	E3 ubiquitin-protein ligase HECTD1	Q69ZR2
KQCPNAVVL	9	2638	Caseinolytic peptidase B protein homolog	Q60649
TSSVNKEQL	9	2660.3	E3 ubiquitin-protein ligase XIAP	Q60989
HCHLNKTSL	9	2662.5	Mono [ADP-ribose] polymerase PARP16	Q7TMM8
AALEKPLSHPN	11	2670.8	Zinc finger MIZ domain-containing protein 1	Q6P1E1-2; Q6P1E1
SMLVSVVGM	9	2692	Serine palmitoyltransferase small subunit A	Q8R207
FCVDNGAMI	9	2699.1	Probable tRNA N6-adenosine threonylcarbamoyltransferase	Q8BWU5
GALRNLSAC	9	2706.3	HEAT repeat-containing protein 3	Q8BQM4
TGPNNTTNF	9	2716.1	Trinucleotide repeat-containing gene 6A protein	Q3UHK8
VAPANSLVHAI	11	2716.4	DmX-like protein 1	Q6PNC0
GSCINVAPHL	10	2735.7	Lysine-specific demethylase 3B	Q6ZPY7; Q6ZPY7-2
SSCVNDIQHL	10	2737.2	F-box DNA helicase 1	Q8K2I9-1
TVGFNVETV	9	2742	ADP-ribosylation factor 6	P62331
FAPPQGSPVM	10	2745.3	SCY1-like protein 2	Q8CFE4; Q8CFE4-2
TCPSNLNDL	9	2746.2	Endophilin-B1	Q9JK48-3; Q9JK48; Q9JK48-2
SCLQLAEQI	9	2747	Uncharacterized protein C1orf112 homolog	Q3TQQ9; Q3TQQ9-2; Q3TQQ9-3
RAIKNGKGL	9	2748.5	staphylococcal nuclease domain-containing protein 1	Q78PY7
IIGINGDYF	9	2763.3	T-complex protein 1 subunit alpha	P11983-2; P11983
ICPLNPFLV	9	2765.4	snRNA-activating protein complex subunit 2	Q91XA5
KGPHNGHIQI	10	2765.8	F-box only protein 31	Q3TQF0
ISHVIVDEI	9	2771.7	Atp-dependent rna helicase a	O70133
LCVQNILKL	9	2773.4	Mucosa-associated lymphoid tissue lymphoma translocation protein 1 homolog	Q2TBA3-1
RVFQNEVLGT	11	2782.2	Translation initiation factor eIF-2B subunit epsilon	Q8CHW4
SQLKGFSLL	9	2794.4	Poly [ADP-ribose] polymerase 1	P11103
SGPTNEDL	8	2803.9	3-hydroxy-3-methylglutaryl-coenzyme A reductase	Q01237
VHVNRDTL	8	2805.9	Bifunctional polynucleotide phosphatase/kinase	Q9JLV6-1; Q9JLV6-2
RAYAALGLPYI	11	2811.4	CREB-binding protein	P45481
VSPTNMQGF	9	2817	Histone deacetylase 3	O88895
YTVENAKDIIA	11	2819.3	Tryptophan--tRNA ligase, cytoplasmic	P32921-2; P32921
RALENTAIKA	10	2825.9	Tripeptidyl-peptidase 2	Q64514; Q64514-2
FAHPGMMQEL	10	2827.1	Apolipoprotein B receptor	Q8VBT6

SAIQNLHSFDF	11		2833.4	Eukaryotic translation initiation factor 1	P48024
TGVGNDENM	9		2834	A-kinase anchor protein 13	E9Q394; E9Q394-2
YSGYSKETL	9		2839.3	phosphatidylinositol 4-phosphate 3-kinase C2 domain-containing subunit alpha	Q61194-1; Q61194-2
RAGPSLKTTL	10		2863	Protein FAM60A	Q8C8M1
VLTNPETL	8		2873.9	Multifunctional procollagen lysine hydroxylase and glycosyltransferase LH3	Q9R0E1
RSLLDQENL	9		2882.1	Protein phosphatase Slingshot homolog 1	Q76I79; Q76I79-2
AAPLSGQTF	9		2889.4	Transcription factor Sp1	O89090-2; O89090
SSITNHINKLDI	12		2898	Semaphorin-5A	Q62217
AAVIDQERL	9		2909.5	Serine/threonine-protein kinase MRCK gamma	Q80UW5
SGVEGQDVV	9		2925.8	Hexokinase-3	Q3TRM8
VNVTDHDVI	9		2925.9	Origin recognition complex subunit 3	Q9JK30-2; Q9JK30
SVVRNVFDF	9		2941.9	von Willebrand factor A domain-containing protein 8	Q8CC88-2; Q8CC88
FCIKNCHRV	9		2945.8	NACHT, LRR and PYD domains-containing protein 3	Q8R4B8-1
ETIMNQEKL	9		2946.4	Transcription factor BTF3	Q64152-2; Q64152
GQLSNGDHHF	10		2954.8	YTH domain-containing family protein 1	P59326
AALTHHPAAM	10		2962.3	Cyclic AMP-responsive element-binding protein 5	Q8K1L0
ACHPNLDKL	9		2965.2	NADH-cytochrome b5 reductase 1	Q9DB73
SAPLGDFRHTI	11		2969.2	Cdc42 effector protein 4	Q9JM96
SIHENFSQAM	10	1xOxidation [M10]	2985.9	Coiled-coil domain-containing protein 93	Q7TQK5
AVANAQEL	8		2986	Mitochondrial import inner membrane translocase subunit Tim13	P62075
TSLKNLRTLHL	11		2986.1	Leucine-rich repeat-containing protein 40	Q9CRC8
VHVQGQEPL	9		2993.9	Polyadenylate-binding protein 1	P29341
YSGSYNDYL	9		3006.6	RNA-binding protein 14	Q8C2Q3
LALLLGDRL	9		3014.2	Monocyte differentiation antigen CD14	P10810
FQYWKRFDL	9		3016.5	Isoform 3 of Homeobox protein cut-like 1	P53564-3
NCGVNAVEL	9		3018.6	Protein kinase C eta type	P23298
RQPKHLLAFL	10		3020.5	Eukaryotic translation initiation factor 2 subunit 2	Q99L45
NCFINRQLI	9		3041.7	T-complex protein 1 subunit beta	P80314
AVCKNTITL	9		3065.1	Isoform 3 of E3 ubiquitin-protein ligase RBBP6	P97868-3
CQCYNGVVL	9		3067.3	Asparagine--tRNA ligase, cytoplasmic	Q8BP47
FIHTNWTGHG	13		3097.1	6-phosphogluconate dehydrogenase, decarboxylating	Q9DCD0
FQNTNVKGVY	11		3097.4	Glutathione reductase, mitochondrial	P47791-2; P47791
YAVRNLTEDN	11		3109.5	Ataxin-10	P28658
SQNVPSTNYL	10		3128.2	Lysine-specific demethylase 3B	Q6ZPY7; Q6ZPY7-2
NAVSGQESL	9		3129.7	NTF2-related export protein 1	Q9QZV9
FCPHNEQIL	9		3174.2	Breast cancer type 2 susceptibility protein homolog	P97929
FKAKNLIEVM	10		3174.4	Sorting and assembly machinery component 50 homolog	Q8BGH2
GALRLTTEF	9		3176.7	Mannosyl-oligosaccharide glucosidase	Q80UM7
KLIRLMEEI	9		3181.4	probable ATP-dependent RNA helicase DDX5	Q61656
AALQSSWGMI	10	1xOxidation [M10]	3192.2	TAR DNA-binding protein 43	Q921F2
GIENIHVM	8		3194.5	Myotubularin-related protein 6	Q9Z2C9; Q8VE11
TPVKNIDTV	9		3206.5	Sister chromatid cohesion protein PDS5 homolog A	Q6A026
VTIILKEPV	9		3210.8	Ribosome-binding protein 1	Q99PL5-10; Q99PL5-11; Q99PL5-6; Q99PL5-12; Q99PL5-4; Q99PL5-9; Q99PL5-7; Q99PL5-1; Q99PL5-8; Q99PL5-5; Q99PL5-3; Q99PL5-2
VGLYLREHI	9		3211.8	Kinesin-like protein KIF15	Q6P9L6
SAPAFGPEAL	10		3226.5	Proteasome subunit beta type-6	Q60692
SALALRWEA	9		3231.1	A-kinase anchor protein 2	O54931-4; O54931-2; O54931-5; O54931
AVCENFQEFL	10		3233.9	Apoptotic protease-activating factor 1	O88879-2; O88879
SALVLVKNSEI	11		3239	Mast cell-expressed membrane protein 1	Q9D8U6
VTPKHQEYL	9		3242.6	Inhibitor of nuclear factor kappa-B kinase subunit epsilon	Q9R0T8
GQWTNKMEF'	11		3243.4	Sodium- and chloride-dependent betaine transporter	P31651

VQVENPERF	9	3247.9	Tripartite motif-containing protein 35	Q8C006
SQPVAVSHI	9	3257.5	Max-binding protein MNT	O08789
KSLQNAAAEVI	14	3263.8	Serine/threonine-protein kinase RIO1	Q922Q2
YQYKGMGLSN	10	3268.5	Proteasome subunit beta type-5	O55234
CSPEHSTEL	9	3283	Denticleless protein homolog	Q3TLR7
TSGVNKESF	9	3294.1	Round spermatid basic protein 1	Q80T69
AAEVNQEYG	9	3299.6	Fumarate hydratase, mitochondrial	P97807; P97807-2
FAIEDAENA	9	3304.4	Transcription factor E2F7	Q6S7F2-2; Q6S7F2
WCPHNDEVI	9	3308.6	Coronin-1B	Q9WUM3
SLMANGTLT	9	3329.5	Catenin delta-1	P30999-2; P30999; P30999-3
LAPTNLSSSRN	13	3335.9	Serine/arginine repetitive matrix protein 2	Q8BTI8; Q8BTI8-2; Q8BTI8-3
GICTNPAFI	9	3354.5	Prolow-density lipoprotein receptor-related protein 1	Q91ZX7
GPKNYEFL	8	3368.5	Sorting nexin-14	Q8BHY8
SCGLNTSSL	9	3381.8	Exonuclease 1	Q9QZ11
SAARGLQDVL	10	3386.7	T-complex protein 1 subunit zeta	P80317
SSPRNSQELS	12	3390.2	caspase recruitment domain-containing protein 9	A2AIV8
QYLCNVEHL	9	3390.6	Dimethyladenosine transferase 1, mitochondrial	Q8JZM0
SAPSSSSLSTL	11	3392.4	Protein phosphatase 1 regulatory subunit 12A	Q9DBR7; Q9DBR7-2
ISGWNGDNML	10	3408.8	Elongation factor 1-alpha 1	P10126
SAPRPSSQFV	10	3442.7	Golgin subfamily A member 5	Q9QYE6
KSIVNTSPPC	10	3478.5	RNA-binding protein 33	Q9CXK9-1
KGFSDKLDFL	10	3505.3	Probable rRNA-processing protein EBP2	Q9D903
AAPRGPGLFL	10	3525.4	N-acetylglucosamine-1-phosphodiester alpha-N-acetylglucosaminidase	Q8BJ48
SAPLPPSIFM	10	3527.4	Farnesyl pyrophosphate synthase	Q920E5
IMGVGQCVI	9	3534.6	long-chain fatty acid transport protein 1	Q60714
WQYPNGTHA	9	3539.9	Probable JmjC domain-containing histone demethylation protein 2C	Q69ZK6
ICPNNHEVHI	10	3542.4	Actin-related protein 2/3 complex subunit 1B	Q9WV32
SCVKSLDVL	9	3542.9	ATP-dependent RNA helicase DDX55	Q6ZPL9
GKLSNQERI	9	3561.1	Ataxin-10	P28658
KVAVNDAHL	9	3579.3	Galectin-3	P16110
SGPWNRGFG	12	3580.2	Protein SCAF8	Q6DID3
KGPENTLRV	9	3583.1	E3 ubiquitin-protein ligase MYCBP2 OS=Mus musculus OX=10090	Q7TPH6; Q7TPH6-2; Q7TPH6
TAPTHVPLQYI	11	3607.1	Serine--tRNA ligase, mitochondrial	Q9JJL8
SCPQGLVHI	9	3607.8	StAR-related lipid transfer protein 9	Q80TF6
SAPMSAAHQL	13	3616	Atrophin-1	O35126
FCGKNGLSL	9	3624	Ubiquitin carboxyl-terminal hydrolase 34	Q6ZQ93; Q6ZQ93-3; Q6ZQ93-4; Q6ZQ93-2
AQGVSIEAM	9	3666.9	INO80 complex subunit C	Q8BHA0
QIVNPHLL	8	3670.5	Ribonucleoside-diphosphate reductase large subunit	P07742
RCLHNFTSSL	10	3689.6	Origin recognition complex subunit 3	Q9JK30-1
SPSTVYLDYM	10	3693.3	Macrosialin	P31996-2; P31996
FHMAMGNPSE	11	3693.3	Segment polarity protein dishevelled homolog DVL-2	Q60838
NKLAMQEFM	9	3722.1	alpha-enolase	P17182; P21550; P17183
TGPSNQWASE	11	3734.5	GRB10-interacting GYF protein 2	Q6Y7W8-2; Q6Y7W8
GMVVDVENL	9	3772.9	Histone acetyltransferase KAT2A	Q9JHD2
YQLAHQISVTL	11	3780.2	Threonine--tRNA ligase, mitochondrial	Q3UQ84
KTVTNAVVT	9	3790.6	Heat shock cognate 71 kDa protein	P63017
SSGRDAALM	9	3819.4	T-complex protein 1 subunit beta	P80314
CSVRDQELL	9	3823.8	breast cancer type 1 susceptibility protein homolog	P48754
SQLTGLGTAV	10	3828.5	Protocadherin Fat 4	Q2PZL6
GCFKNSQVL	9	3830.8	C-type lectin domain family 4 member E	Q9R0Q8
FAWEPNGSKF	13	3835.4	Eukaryotic translation initiation factor 3 subunit B	Q8JZQ9
FAPKSWMEDS	11	3869	Cytoplasmic polyadenylation element-binding protein 4	Q7TN98-5; Q7TN98-1; Q7TN98-2; Q7TN98-3; Q7TN98-4

TAPGNSPERL	10		3869.1	Microtubule-associated protein 1S	Q8C052
YALSNAIGP	9		3871.6	Endophilin-B1	Q9JK48-3; Q9JK48; Q9JK48-2
FGVKNAKLSVI	11		3876.7	Isoleucine--tRNA ligase, cytoplasmic	Q8BU30
RTIELKTEI	9		3911.6	DNA repair protein RAD50	P70388; P70388-2; P70388-4
RTLQGHSVI	9		3933.7	Transcriptional repressor p66-beta	Q8VHR5-2; Q8VHR5
SSLKNFQSC	9		3957.3	E3 SUMO-protein ligase NSE2	Q91VT1; Q91VT1-2
SLYTNAKGGT	11		3970.5	Protein max	P28574; P28574
TISSNLTEM	9	1xOxidation [M9]	3991	Talin-2	Q71LX4; P26039
TGIVNHTHS	9		3997	Histone-arginine methyltransferase CARM1	Q9WVG6-1
RCFENTFQEL	10		4004.3	PAB-dependent poly(A)-specific ribonuclease subunit PAN3	Q640Q5-1
AQCDKTNKEF	11		4013.3	NADH dehydrogenase [ubiquinone] 1 alpha subcomplex subunit 8	Q9DCJ5
ISPHNNQHF	9		4015.4	Neurofibromin	Q04690-3; Q04690; Q04690-4; Q04690-2
TIVNIHEL	8		4020.5	Constitutive coactivator of peroxisome proliferator-activated receptor gamma	Q6RI63; Q6RI63-2
AQYENPPHIY/	11		4028.7	Unconventional myosin-Ie	E9Q634
FVHSYGEYM	9		4048	Cyclin-T1	Q9QWV9
FSSLNLRETNL	11		4061.7	Vimentin	P20152
SSPQHQELF	9		4083.9	Very long-chain specific acyl-CoA dehydrogenase, mitochondrial	P50544
VSPKKSTVL	9		4085.3	Talin-1	P26039
RGIVNGAAP	9		4112.1	V-type proton ATPase subunit B, brain isoform	P62814
SCPYGTVYL	9		4116.9	CREB-regulated transcription coactivator 1	Q68ED7
SCIKNPKILL	10		4124.3	1-phosphatidylinositol 3-phosphate 5-kinase	Q9Z1T6
RSLENNKEAKI	11		4130.1	Condensin-2 complex subunit G2	Q6DFV1
SAQILASAL	9		4141.7	Matrix metalloproteinase-15	O54732
SGPAGGAAL	9		4153.9	Protein phosphatase 1 regulatory subunit 14B	Q62084
AAPKNRRTIEV	12		4159.8	39S ribosomal protein L32, mitochondrial	Q9DCI9
KAISSSVHSL	10		4182.8	Protein Tob1	Q61471
SSLGKHSTM	9		4188.7	protein THEMIS2	Q91YX0
TCAINNTLI	9		4196.7	Phosphatidylinositol glycan anchor biosynthesis class U protein	Q8K358
TQVEGGATL	9		4200.4	Asparagine--tRNA ligase, cytoplasmic	Q8BP47
AAIRNSITSC	10		4205.3	Forkhead box protein N3	Q499D0
ACAINKVLM	9	1xOxidation [M9]	4211.6	protein FAM98A	Q80VD1; Q3TJZ6
LRHVNIDHL	9		4216.3	Eukaryotic translation initiation factor 3 subunit H	Q91WK2
WCPHNDNVI	9		4248	Coronin-1A	Q920M5-4; Q920M5-2; Q920M5; O89053; Q920M5-3
SGPCGGPEL	9		4251.2	Bromo adjacent homology domain-containing 1 protein	Q497V6-2; Q497V6
NCGANVDLL	9		4276.6	Cell division control protein 45 homolog	Q9Z1X9
SVLNVNHI	8		4288.9	Ankyrin repeat domain-containing protein 17	Q99NH0-1
YMTEGHLAM	9	1xOxidation [M9]	4318.3	dual specificity tyrosine-phosphorylation-regulated kinase 1A	Q61214
ATPGRLIDFL	10		4326.3	Probable ATP-dependent RNA helicase DDX17	Q501J6; Q501J6-2; Q61656
FGLSARDLDEI	11		4328.4	Matrin-3	Q8K310
SPQNQEQI	8		4333.5	Metal regulatory transcription factor 1	Q07243
WAAGKGNDD'	11		4361.9	Inversin	O89019-7; O89019-3; O89019-6; O89019; O89019-4; O89019-2; O89019-5
RLITLADHI	9		4372.7	Nuclear receptor corepressor 1	Q60974-1; Q60974-2
RAWVCLAGA/	13		4378.6	NHL repeat-containing protein 3	Q8CCH2
SCPSSIITL	9		4388.6	NFU1 iron-sulfur cluster scaffold homolog, mitochondrial	Q9QZ23
RSPENPPSKE	11		4404.4	Centromere protein M	Q9CQA0-1
FAIQNKHLCH	10		4415.1	Cyclic AMP-dependent transcription factor ATF-3	Q60765
GCQVNQDYF	9		4416.5	General vesicular transport factor p115	Q9Z1Z0-2; Q9Z1Z0-3; Q9Z1Z0
FSQAWLEEL	9		4431.2	Brefeldin A-inhibited guanine nucleotide-exchange protein 3	Q3UGY8
SNYEKYYLI	9		4440	phosphatidylinositol 4-phosphate 3-kinase C2 domain-containing subunit alpha	Q61194-1; Q61194-2
KMLDKYSHYL	10		4444.8	dedicator of cytokinesis protein 11	A2AF47
AMGTNDTVIG(	14		4499.2	Fatty acid synthase	P19096

RGPENLLDHQ	11		4508.5	Melanoma inhibitory activity protein 2	Q91ZV0; Q8R311
WHNLNYWQL	9		4528.9	Peroxisomal carnitine O-octanoyltransferase	Q9DC50
GMNRNQAFG	9		4539.5	CCR4-NOT transcription complex subunit 2	Q8C5L3-2; Q8C5L3; Q8C5L3-3
KSVISVIHL	9		4543	CDP-diacylglycerol--inositol 3-phosphatidyltransferase	Q8VDP6
AAVTGHIRI	9		4568.2	Coatomer subunit beta	Q9JIF7
LMGSSQATM	9		4577	Clathrin interactor 1	Q99KN9; Q99KN9-2
NMPGVMTGTI	10		4594.2	Clathrin interactor 1	Q99KN9-1
RHAAVLVETI	10		4595.2	CLIP-associating protein 2	Q8BRT1
SSVKNEEQFV	14		4602.6	Maternal embryonic leucine zipper kinase	Q61846
RSPISRQQDPI	11		4604.4	E3 ubiquitin-protein ligase RBBP6	P97868; P97868-2
YAPSSLGGAM	11		4606.4	YTH domain-containing family protein 2	Q91YT7
SMMVKQEGM	9		4608.5	Hyaluronan mediated motility receptor	Q00547-2; Q00547-1
RAPRNWNKP	9		4613	Arginine vasopressin-induced protein 1	Q9D7H4
GLKNLTAL	8		4619	Phosphoinositide 3-kinase adapter protein 1	Q9EQ32-3; Q9EQ32-2; Q9EQ32-1
VGPGNCVLV	9		4620.3	Scm-like with four MBT domains protein 1	Q9JMD1
AAPIDRVGQTI	11		4622.3	Heterogeneous nuclear ribonucleoprotein M	Q9D0E1-2; Q9D0E1
QLVRNIVPA	9		4633.5	ATP-dependent (S)-NAD(P)H-hydrate dehydratase	Q9CZ42; Q9CZ42-2; Q9CZ42-3
TSAVTDEFM	9	1xOxidation [M9]	4651	Isoform 6 of RNA binding protein fox-1 homolog 1	Q9JJ43-7; Q9JJ43-6; Q9JJ43-5
NCILDKDYL	9		4659.7	DNA repair protein RAD52 homolog OS=Mus musculus OX=10090	P43352
NGVRNGLAA	9		4663.5	Sphingosine-1-phosphate phosphatase 1	Q9JI99
SSCVNDIQHLL	11		4692.9	F-box DNA helicase 1	Q8K2I9-1
FMESGGTVL	9	1xOxidation [M2]	4709.5	Protein SGT1 homolog	Q9CX34
RNKENQETL	9		4722.1	STE20-like serine/threonine-protein kinase	O54988-2; O54988-1
SAIQNLKTTDK	14		4731.7	TRAF family member-associated NF-kappa-B activator	P70347-2; P70347-4; P70347
TAVEDSDKAYI	11		4741.9	Proline-serine-threonine phosphatase-interacting protein 2	Q99M15
ECHLNADTV	9		4742.6	AP-2 complex subunit beta	Q9DBG3-2; Q9DBG3
TIGFNVETV	9		4769	ADP-ribosylation factor 4	P61211; P61750; P61205; P84078; Q8BGX0-2; Q8BSL7; Q8BGX0-3; Q8BGX0; P84084
SAPVSGPGI	9		4773.5	Transcription intermediary factor 1-beta	Q62318
AAPGSKDHL	9		4782.6	Transmembrane protein 41B	Q8K1A5
YLGSNHISSI	10		4821.8	CD180 antigen	Q62192
VQPTHVIHL	9		4828.6	GDP-L-fucose synthase	P23591
SSFSMEEGDV	11		4830.2	transcription factor EB	Q9R210
KCLELFTEL	9		4839.1	Heat shock protein HSP 90-alpha	P07901
VGPDNVHVAL	10		4840.7	Structural maintenance of chromosomes protein 2	Q8CG48
GMLEKIDMI	9		4841.9	RNA-binding protein 12	Q8R4X3
VQYTLPDGST	11		4846.5	Beta-centractin	Q8R5C5
STPQSPAKAFI	13		4850.2	CCR4-NOT transcription complex subunit 1	Q6ZQ08-2; Q6ZQ08-4; Q6ZQ08
FQHPSHELL	9		4870.7	La-related protein 1	Q6ZQ58
ATVSNREHED	11		4891.4	NFX1-type zinc finger-containing protein 1	Q8R151
FGLQHLSSGH	11		4916.6	Adenylate kinase 4, mitochondrial	Q9WUR9
SCLRNVTQT	9		4925.2	Rab3 GTPase-activating protein non-catalytic subunit	Q8BMG7
SGPENFQVG	9		4938.8	Talin-1	P26039
RAHENRVKDM	13		4943.6	p21-activated protein kinase-interacting protein 1	Q9DCE5
TCPSNLGTGL	10		4946	Creatine kinase U-type, mitochondrial	P07310; P30275; Q6P8J7; Q04447
SVCTNVPGSY	11		4948.8	Adhesion G protein-coupled receptor E1	Q61549
SQYRFTVSDL	10		4952.9	Cyclin-G1	P51945
ACYNNQQVF	9		4965.9	Squalene synthase	P53798
YSNRVVDLM	9		4985.4	Glyceraldehyde-3-phosphate dehydrogenase	P16858
