# Supplementary material for: Palladium-Induced Temporal Internalization of MHC Class I Contributes to T Cell-Mediated Antigenicity
Source: Front Immunol. 2021 Dec 23;12:736936. doi: 10.3389/fimmu.2021.736936 (PMC8732370; doi:10.3389/fimmu.2021.736936)
Supplement: Supplementary file 5 [file Table_4.pdf]

Supplementary Table 4 Peptide list on H-2D^b in the presence of $PdCl_2$

Sequence	Sequence Length	Modification	Affinity (nM)	Protein	Protein Accession
SAISNLDYI	9		1.9	Guanine nucleotide exchange factor VAV3	Q9R0C8-4; Q9R0C8-1
YAISNIEVI	9		2.4	Netrin-G2	Q8R4F1; Q8R4F1-3; Q8R4F1-2
TAVVNVTYM	9		2.8	Insulin-like growth factor 2 mRNA-binding protein 2	Q5SF07-2; Q5SF07
YSITNTEEL	9		3.2	Leucine-rich repeats and immunoglobulin-like domains protein 2	Q52KR2
VALSNMNVI	9		3.2	Puromycin-sensitive aminopeptidase	Q11011
YAVGNHDFI	9		3.2	PCI domain-containing protein 2	Q8BFV2
FAIQNPTLI	9		3.2	Transcription factor Sp2	Q9D2H6; Q9D2H6-2
RAIENIDTL	9		3.4	Protein phosphatase 1 regulatory subunit 7	Q3UM45
YAVNNQFTM	9		3.4	Histone-arginine methyltransferase CARM1	Q9WVG6; Q9WVG6-2
NSIRNLDTI	9		3.6	Ataxin-10	P28658
RAIRNMNTL	9		3.6	E3 ubiquitin-protein ligase synoviolin	Q9DBY1-2; Q9DBY1
ESAISNLDYI	10		3.9	Guanine nucleotide exchange factor VAV3	Q9R0C8-4; Q9R0C8
FALANEHYL	9		4	N-acyl-phosphatidylethanolamine-hydrolyzing phospholipase D	Q8BH82
FSVTNPHTM	9		4	Ligand-dependent nuclear receptor-interacting factor 1	Q8CDD9-2; Q8CDD9
YQLSNLYAI	9		4	Multivesicular body subunit 12B	Q6KAU4
VALINKDFL	9		4	Transmembrane protein 82	Q8R115
SQISNTEFL	9		4.1	BRISC complex subunit Abraxas 2	Q3TCJ1
AALANLDNI	9		4.1	Arf-GAP domain and FG repeat-containing protein 1	Q8K2K6; Q8K2K6-3; Q8K2K6-1; Q8K2K6-2
YMLANLTHL	9		4.2	kelch-like protein 28	Q9CR40-1; Q9CR40-2
ASLSNRLYI	9		4.2	phosphatidylinositol 4-kinase alpha	E9Q3L2
SGIRNISFM	9		4.4	Disintegrin and metalloproteinase domain-containing protein 10	O35598
KSIVNQVFL	9		4.4	RING finger and transmembrane domain-containing protein 1	Q9DCN7
SAMENLNEM	9		4.4	N-alpha-acetyltransferase 16, NatA auxiliary subunit	Q9DBB4
YSLGNTYTL	9		4.4	G-protein-signaling modulator 1	Q6IR34-3; Q6IR34-2; Q6IR34; Q8VDU0; Q6IR34-5
STIRNADVI	9		4.6	Phosphatidylcholine translocator ABCB4	P21440
FAIRNILDYV	10		4.6	Isoform 2 of Poly [ADP-ribose] polymerase 9	Q8CAS9-3; Q8CAS9-2; Q8CAS9
YAPINANAI	9		4.6	Metastasis-associated protein MTA2	Q9R190
TAPVNIAVI	9		4.6	Diphosphomevalonate decarboxylase	Q99JF5
KAPTNVTCI	9		4.6	von Willebrand factor A domain-containing protein 8	Q8CC88-2; Q8CC88
RALSNLESI	9		4.7	Isoform 2 of Ubiquilin-1	Q8R317-2; Q8R317; Q8C5U9
SSLINGSFL	9		4.8	Tyrosine-protein kinase ABL2	Q4JIM5
FSVENFDAL	9		4.9	Integrin alpha-X	Q9QXH4
AAMKNVTEL	9		5	14-3-3 protein gamma	P61982
YGLKNLTAL	9		5	Phosphoinositide 3-kinase adapter protein 1	Q9EQ32-3; Q9EQ32-2; Q9EQ32-1
SAIRNGTDM	9		5.1	Coiled-coil domain-containing protein 189	Q6NZQ0
SMLSNPDLM	9		5.1	Isoform 2 of Ubiquilin-1	Q8R317-2; Q8R317; Q9QZM0
SAVENLNEM	9	1xOxidation [M9]	5.1	N-alpha-acetyltransferase 15, NatA auxiliary subunit	Q80UM3
YSVNNVSVI	9		5.1	Ankyrin repeat domain-containing protein 13A	Q80UP5
SQIVNPESI	9		5.3	Pentatricopeptide repeat-containing protein 2, mitochondrial	Q8R3K3
AALLNQQFL	9		5.4	Serine/threonine-protein phosphatase 2B catalytic subunit gamma isoform	P48455; P48453-2; P48453-1
SSLENATSL	9		5.4	Signal peptide peptidase-like 2A	Q9JJF9
KALINADEL	9		5.5	Spectrin alpha chain, non-erythrocytic 1	P16546; P16546-2
YAIENVETNL	10		5.5	transportin-3	Q6P2B1-1; Q6P2B1-2
SAILNEWTL	9		5.5	Pleckstrin homology domain-containing family M member 1	Q7TSI1

SMLINGYAL	9	5.5	T-complex protein 1 subunit alpha	P11983-2; P11983
RQILNADAM	9	5.6	protein virilizer homolog	A2AIV2-1
VQIANPAFI	9	5.6	Protein mono-ADP-ribosyltransferase PARP4 OS=Mus musculus OX=10090	E9PYK3
SSLQNLTSL	9	5.6	Toll-like receptor 13	Q6R5N8
YGLLNVTKI	9	5.8	Rho GTPase-activating protein 12	Q8C0D4
RSLENYHFV	9	5.8	Clathrin interactor 1	Q99KN9-1; Q99KN9-2
FAHTNIESL	9	5.9	Protein disulfide-isomerase A3	P27773
ASIENTSTL	9	5.9	PHD finger protein 12	Q5SPL2-2; Q5SPL2
AALLNTDLV	9	5.9	U3 small nucleolar RNA-interacting protein 2	Q91WM3
YQYSNTVYL	9	6	Aminopeptidase N	P97449
KSLTNLSFL	9	6	Neuron navigator 1	Q8CH77-2; Q8CH77; Q8CH77-3
VALRNLEQI	9	6.1	SH3 domain-binding protein 5-like	Q99LH9
KSLKNYITI	9	6.1	Probable cysteine--tRNA ligase, mitochondrial	Q8BYM8
AAPSNLPYL	9	6.1	Dual specificity protein phosphatase 4	Q8BFV3
FSVLNWDQV	9	6.2	Protein FAM46C	Q5SSF7
ASMTNRELM	9	6.2	ADP-dependent glucokinase	Q8VDL4; Q8VDL4-3
VALRNINLI	9	6.2	AP-1 complex subunit beta-1	Q9DBG3-2; O35643; Q9DBG3
KSVANLEYL	9	6.2	GRIP and coiled-coil domain-containing protein 2	Q8CHG3
RSLSNATII	9	6.3	signal recognition particle 54 kDa protein	P14576-2; P14576-1
SALANYIHL	9	6.3	Integral membrane protein GPR180	Q8BPS4
SSLQNYAKI	9	6.3	natural resistance-associated macrophage protein 1	P41251
AALQNLVKI	9	6.4	Importin subunit beta-1	P70168
RMLENYEEI	9	6.4	Cullin-7	Q8VE73; Q8VE73-3
FAPVNVTTEV	10	6.4	Elongation factor 1-alpha 1	P10126
SSLENAFQI	9	6.5	DDB1- and CUL4-associated factor 17	Q3TUL7-2; Q3TUL7
SSVLNLTEL	9	6.6	glycosyltransferase-like domain-containing protein 1	Q8BW56-1; Q8BW56-2
KALANVATV	9	6.6	Nicastrin	P57716
FGIKNFSAI	9	6.6	oyllysine-residue acetyltransferase component of pyruvate dehydrogenase complex, mit	Q8BMF4
SGLANGIFM	9	6.6	Mucosa-associated lymphoid tissue lymphoma translocation protein 1 homolog	Q2TBA3-2; Q2TBA3
YAIVGLETI	9	6.6	Xaa-Pro aminopeptidase 1	Q6P1B1
KANVNLAYL	9	6.7	RNA-binding protein 24	D3Z4I3; Q62176
SAYLNGHWL	9	6.7	Ras GTPase-activating protein 3	Q60790
YAMENTRQTI	10	6.7	Nuclear receptor corepressor 1	Q60974-1; Q60974-2
SSPCNIEVV	9	6.7	DNA replication ATP-dependent helicase/nuclease DNA2	Q6ZQJ5; Q6ZQJ5-2
YMNVNNYWI	9	6.7	GPI inositol-deacylase	Q3UUQ7
RSIQNAQFL	9	6.8	Lysosomal acid phosphatase	P24638
AQMQNPDTL	9	6.8	Isoform 2 of Ubiquilin-1	Q8R317-2
SSLHNVYLI	9	6.8	Origin recognition complex subunit 2	Q60862
ASLVNADKL	9	6.9	Vacuolar protein sorting-associated protein 33B	P59016
AAIENIEHL	9	6.9	Leucine-rich PPR motif-containing protein, mitochondrial	Q6PB66
FQFINPTTI	9	6.9	GATOR complex protein WDR59	Q8C0M0-1; Q8C0M0-2; Q8C0M0-3
ASVINGHTL	9	6.9	Serine/threonine-protein phosphatase 6 regulatory ankyrin repeat subunit B	B2RXR6
SSLKNGVVL	9	6.9	Rho guanine nucleotide exchange factor 6	Q8K4I3
SAIINLFHL	9	6.9	Transformation/transcription domain-associated protein	Q80YV3
FSPTNYHFL	9	7	Beta-mannosidase	Q8K2I4
SSPLNHIYL	9	7	Methionine aminopeptidase 1D, mitochondrial	Q9CPW9
YQVLNEVVI	9	7.1	NAD kinase	P58058
AALENTHLL	9	7.2	Mitotic spindle-associated MMXD complex subunit MIP18	Q9D187

VAVRNLQEI	9	7.2	alpha-galactosidase A	P51569
SSLSNEHVL	9	7.2	Serine/threonine-protein phosphatase 6 regulatory ankyrin repeat subunit C	Q8BTI7
AAITNKYQL	9	7.2	RNA polymerase I-specific transcription initiation factor RRN3	B2RS91
RALLNAESL	9	7.2	Exonuclease 3'-5' domain-containing protein 2	Q8VEG4-2; Q8VEG4
SSHCNAEAM	9	7.2	Protein JBTS17	Q8CE72
SSVSNKTTL	9	7.2	LisH domain and HEAT repeat-containing protein KIAA1468	Q148V7-3; Q148V7-1; Q148V7-2
SAVRNHTYQM	10	7.2	FTS and Hook-interacting protein	Q3U2I3-3; Q3U2I3-2; Q3U2I3
YALSDLDTL	9	7.2	THAP domain-containing protein 3	Q8BJ25
ASFSNSTYL	9	7.3	Myotubularin-related protein 13	E9PXF8-2; E9PXF8
SMVQNRVFL	9	7.4	Dedicator of cytokinesis protein 2	Q8C3J5
KAPVNTAEL	9	7.4	BRCA2 and CDKN1A-interacting protein	Q9CWI3
MGILNTDTL	9	7.4	Rho GTPase-activating protein 24	Q8C4V1-3; Q8C4V1; Q8C4V1-2
SSIENKQDWI	10	7.5	Triple functional domain protein	Q0KL02-4; Q0KL02; Q0KL02-3; Q0KL02-2
AAPTNANSL	9	7.5	CCR4-NOT transcription complex subunit 4	Q8BT14; Q8BT14-2; Q8BT14-3
FQIVNPHLL	9	7.6	Ribonucleoside-diphosphate reductase large subunit	P07742
SAAFNIVYI	9	7.6	Solute carrier family 22 member 15	Q504N2; Q504N2-3
YSLPNAPTL	9	7.6	Tubulin polyglutamylase complex subunit 2	Q66JT5
AALKNAFSL	9	7.7	DmX-like protein 2	Q8BPN8; Q8BPN8-2; Q6PNC0
GSLTNLHTL	9	7.7	Zinc finger protein 40	Q03172
STISNDVFI	9	7.7	Small G protein signaling modulator 2	Q80U12; Q80U12-2
RSIKNVTEL	9	7.8	protein RTF2 homolog	Q99K95
RALSNLESV	9	7.8	Ubiquilin-4	Q99NB8
SAALNKDFL	9	7.8	Centrosome-associated protein 350	E9Q309
AALQNLEQL	9	7.8	Nuclear factor NF-kappa-B p100 subunit	Q9WTK5
LSLPNTDYI	9	7.8	Interferon-inducible double-stranded RNA-dependent protein kinase activator A	Q9WTX2
YSVRNIFHL	9	7.9	Centromere/kinetochore protein zw10 homolog	O54692
YSLPNYLSM	9	7.9	Myoferlin	Q69ZN7-4; Q69ZN7
TAILNDYEL	9	7.9	Isoform 1 of Ninein-like protein	Q6ZQ12-1; Q6ZQ12-2; Q6ZQ12
YQYTNSEVL	9	7.9	Exportin-T	Q9CRT8
RQLENGTTL	9	8	Extended synaptotagmin-2	Q3TZZ7-2; Q3TZZ7-1
SGLVNHVPL	9	8	Aminoacyl tRNA synthase complex-interacting multifunctional protein 1	P31230
FAHHNRYVL	9	8.2	Tyrosine-protein kinase Fes/Fps	P16879
YVHVNRDTL	9	8.3	Bifunctional polynucleotide phosphatase/kinase	Q9JLV6-1; Q9JLV6-2
FGLSNYHQL	9	8.5	Regulator of chromosome condensation	Q8VE37
SMLGNYDEM	9	8.5	AF4/FMR2 family member 4	Q9ESC8
YSNRNLTHV	9	8.6	toll-like receptor 6	Q9EPW9
VMIPNVETI	9	8.6	Putative sodium-coupled neutral amino acid transporter 10	Q5I012-5; Q5I012-3; Q5I012-1; Q5I012-6; Q5I012-2; Q5I012-4
VSVANVDLL	9	8.6	Lethal(2) giant larvae protein homolog 1	Q80Y17
LAIINPVEI	9	8.6	Protein aurora borealis	Q8BS90; Q8BS90-2
ASVSNPLFL	9	8.6	Exostosin-2	P70428
VALGNMYEL	9	8.6	Poly [ADP-ribose] polymerase 1	P11103-2; P11103
SALNNFQVV	9	8.7	Syntaxin-12	Q9ER00
GSLKNVTTL	9	8.7	Erbin	Q80TH2-2; Q80TH2; Q80TH2-1
VSVTNLEEL	9	8.7	Nesprin-1	Q6ZWR6-4; Q6ZWR6
KSLKNFITI	9	8.8	Cysteine--tRNA ligase, cytoplasmic	Q9ER72; Q9ER72-2
LSIENAEEI	9	8.8	Activating signal cointegrator 1	Q9QXN3; Q9QXN3-2
ASLSNLHSL	9	8.9	Protein chibby homolog 1	Q9D1C2

SQISNGSHM	9		8.9	Serine/threonine-protein kinase TAO1	Q5F2E8
AMPINKATI	9		8.9	ATP-dependent zinc metalloprotease YME1L1	O88967
SSLANILEL	9		8.9	Translational activator of cytochrome c oxidase 1	Q8K0Z7
SSVVNFQII	9		8.9	Zinc finger CCCH-type antiviral protein 1-like	Q8BFR1; Q8BFR1-2
RQIFNGTFV	9		9	60S ribosomal protein L7	P14148
KAVLNSEVL	9		9	Shootin-1	Q8K2Q9-1; Q8K2Q9-2
FSMTNNVLL	9		9	Mitogen-activated protein kinase kinase kinase 6	Q9WTR2
FGPVNHEEL	9		9.1	Cyclin-dependent kinase inhibitor 1B	P46414
HAHLNLHYL	9		9.1	Frataxin, mitochondrial	O35943
YTATNQDFI	9		9.1	Nischarin	Q80TM9-3; Q80TM9-2; Q80TM9-1
MMHSNMETL	9	1xOxidation [M6]	9.1	Protein diaphanous homolog 1	O08808
TALENLIVL	9		9.2	E3 ubiquitin-protein ligase hectd1	Q69ZR2
HSLENCTTM	9	Carbamidomethyl [C	9.3	Leucyl-cystinyl aminopeptidase	Q8C129
YSHFNETLL	9		9.3	Multidrug resistance-associated protein 1	O35379
QQIVNIDLM	9		9.4	Ufm1-specific protease 2	Q99K23
KSLENWDYF	9		9.4	NLR family CARD domain-containing protein 4	Q3UP24
FAVVNHQGTL	10		9.5	ubiquitin carboxyl-terminal hydrolase 22	Q8CEG8; Q5DU02
AALPNIYEL	9		9.5	DNA replication licensing factor MCM5	P49718
AAPRNKHWL	9		9.6	G patch domain and KOW motifs-containing protein	Q56A08
AMLTNLESL	9		9.6	anaphase-promoting complex subunit 2	Q8BZQ7
VAIRNFLAL	9		9.6	Transmembrane protein 237	Q3V0J1; Q3V0J1-2; Q3V0J1-3
WAFKNPDTI	9		9.7	Endoplasmic reticulum-Golgi intermediate compartment protein 3	Q9CQE7-2; Q9CQE7
RSLINLHLM	9		9.7	Transmembrane protein 248	Q3TBN1
VMVTNVTSL	9	1xOxidation [M2]	9.7	Talin-2	Q71LX4; P26039
RAGTNLTTL	9		9.8	BAH and coiled-coil domain-containing protein 1	Q3UHR0
SSIQNGKYTL	10		9.8	Lysine-specific demethylase 7A	Q3UWM4
SQLRNEVAI	9		9.8	Serine/threonine-protein kinase D3	Q8K1Y2; Q62101; Q8BZ03
SAVTDFDFI	9		9.8	52 kDa repressor of the inhibitor of the protein kinase	Q9CUX1
FAPENTCHL	9	Carbamidomethyl [C	9.9	Inositol 1,4,5-trisphosphate receptor-interacting protein-like 1	A2ASA8
ISPVNPVAI	9		9.9	RNA-binding protein 47	Q91WT8-1
FALSNEHYSL	10		9.9	Leucine zipper protein 1	Q8R4U7
YMPQNPCII	9	Carbamidomethyl [C	10	Histone-binding protein RBBP4	Q60972
ASHLNLDAL	9		10.1	E3 ubiquitin-protein ligase TRIM32	Q8CH72
RSGLNATFM	9		10.1	Ribosomal protein S6 kinase alpha-4	Q9Z2B9
VALENANAV	9		10.1	Nucleolar protein 56	Q9D6Z1
SGLQNVPPL	9		10.1	Oxidative stress-responsive serine-rich protein 1	Q9D722
WSMENLEEI	9		10.1	DNA-directed RNA polymerase II subunit RPB2	Q8CFI7
KQIENGVYL	9		10.2	Protein odr-4 homolog	Q4PJX1-2; Q4PJX1-1
SAVWNSPPL	9		10.2	Inner centromere protein	Q9WU62-2; Q9WU62-1
SSYENPWTI	9		10.2	Cardiolipin synthase (CMP-forming)	Q80ZM8
LQLLNTDYL	9		10.2	Pericentriolar material 1 protein	Q9R0L6; Q9R0L6-2
MSMTNTHEL	9		10.3	Protein FAM26F	Q8C9E8
SALRMADVI	9		10.3	Putative pre-mRNA-splicing factor ATP-dependent RNA helicase DHX32	Q8BZS9-2; Q8BZS9-1
FSLENLRTM	9		10.3	KAT8 regulatory NSL complex subunit 1	Q80TG1-2; Q80TG1-3; Q80TG1; Q80TG1-4
VALLNLENM	9		10.3	Protein RRP5 homolog	Q6NS46
YSHWNLILI	9		10.4	Probable palmitoyltransferase ZDHHC16	Q9ESG8
VSILNRQVL	9		10.4	nuclear mitotic apparatus protein 1	E9Q7G0
HAIENIDTF	9		10.4	Breast cancer type 2 susceptibility protein homolog	P97929

SSVLNVVSM	9		10.4	Histone-lysine N-methyltransferase 2A	P55200-2; P55200
VGLSNLQFI	9		10.4	Solute carrier family 23 member 2	Q9EPR4
LSMRNTSVM	9	1xOxidation [M9]	10.5	BCL2/adenovirus E1B 19 kDa protein-interacting protein 3	O55003
VGIENIHVM	9		10.6	Myotubularin-related protein 6	Q8VE11
GQITNFEYL	9		10.6	Lysosomal-trafficking regulator	P97412
TAMKNVCEI	9		10.7	Ropporin-1-like protein OS=Mus musculus OX=10090	Q9EQ00
IAVANAQEL	9		10.7	mitochondrial import inner membrane translocase subunit TIM13	P62075
MSIENQEEL	9		10.7	Centromere-associated protein E	Q6RT24
SSVQNYFHL	9		10.8	Trafficking kinesin-binding protein 1	Q6PD31-1; Q6PD31-2
KANENASFL	9		10.8	CAP-Gly domain-containing linker protein 1	Q922J3-2; Q922J3-1
TSVENHEFL	9		10.8	protein elys	Q8CJF7
AAINNRLEL	9		10.8	Palmitoyltransferase ZDHHC13	Q9CWU2
SAIQNLHSF	9		10.9	eukaryotic translation initiation factor 1	P48024
ASYVNLPTI	9		10.9	40S ribosomal protein SA	P14206
FAIINSNPIEL	11		11	Transmembrane protein 131	O70472
SQMTNLQEL	9		11	Volume-regulated anion channel subunit LRRC8D	Q8BGR2
FTVTNTDCL	9		11	DNA polymerase epsilon subunit 2	O54956
KSLLNRYAV	9		11.1	Translocon-associated protein subunit beta	Q9CPW5
FGIKNMDQV	9		11.1	Protein C-ets-2	P15037
KALVNAVKL	9		11.2	vacuolar protein sorting-associated protein 13b	Q80TY5; Q80TY5
SQLLNLTQL	9		11.2	Erbin	Q80TH2-2; Q80TH2; Q80TH2-1
VSLINAHSL	9		11.2	E3 ubiquitin-protein ligase RNF31	Q924T7; Q924T7-2
ALIQNADTL	9		11.2	SH3 domain-binding protein 1	P55194
SMNVNEIFM	9		11.3	Ras-related protein Rab-5A	Q9CQD1
KAYANPTVV	9		11.3	F-box only protein 11	Q7TPD1-1; Q7TPD1-2; Q7TPD1-3
SALVNHMIV	9		11.4	small G protein signaling modulator 1	Q8BPQ7; Q8BPQ7-2
KSLENILTL	9		11.4	F-box DNA helicase 1	Q8K2I9-1
IQVRNMATL	9		11.4	ATP synthase subunit gamma, mitochondrial	Q91VR2
HGITNLCVI	9	Carbamidomethyl [(	11.5	ATP-dependent 6-phosphofructokinase, liver type	P12382
QGLVNRAYI	9		11.5	Exocyst complex component 6B	A6H5Z3; A6H5Z3-2
STYINASYI	9		11.5	Receptor-type tyrosine-protein phosphatase C	P06800-5; P06800-6; P06800
SSPSNLQII	9		11.5	Ubiquitin carboxyl-terminal hydrolase 24	B1AY13
YSIINGIVF	9		11.6	Palmitoyltransferase ZDHHC3	Q8R173
FGATNWDLI	9		11.7	Protein RCC2	Q8BK67
GALKNTDYF	9		11.7	Cytosolic 5'-nucleotidase 3A	Q9D020; Q9D020-1
YLVVNPNYL	9		11.8	DNA replication licensing factor MCM6	P97311
KAVKNWQFV	9		11.9	Ribonuclease H2 subunit A	Q9CWY8
SQHVNLDQL	9		11.9	Beclin 1-associated autophagy-related key regulator	Q8CDJ3
SGPSNLLEI	9		11.9	T-cell-interacting, activating receptor on myeloid cells protein 1	B6A8R8
RALSNTLLL	9		11.9	Exportin-6	Q924Z6-2; Q924Z6
YGIRNSLLI	9		12	Ribonucleoside-diphosphate reductase large subunit	P07742
SSPANISSL	9		12	Calpastatin	P51125-4; P51125-7; P51125-6; P51125-5; P51125-1; P51125-3; P51125-2
AALVNVQIPL	10		12	ATP-binding cassette sub-family B member 8, mitochondrial	Q9CXJ4
SAVENVVKL	9		12.1	Phospholipase A1 member A	Q8VI78
SGVSNPHVI	9		12.1	YEATS domain-containing protein 2	Q3TUF7-2; Q3TUF7; Q3TUF7-3
SSPRNHLAM	9		12.1	Regulator of G-protein signaling 12	Q8CGE9
RAVQNHNTYM	10		12.1	Adhesion G protein-coupled receptor E1	Q61549

FAPKNIYSI	9	12.1	Macrophage colony-stimulating factor 1 receptor	P09581
LAIRNDEEL	9	12.2	Histone H2AX	Q64523; Q8BFU2; P22752; Q8CGP5; Q8R1M2; P27661; Q8CGP7; Q8CGP6; Q6GSS7
TGIRNLEWL	9	12.2	Transmembrane protein 260	Q8BMD6-1; Q8BMD6-2
FALTNPEKSI	10	12.2	nucleotide exchange factor SIL1	Q9EPK6
FSPFNPTSL	9	12.3	ETS-related transcription factor Elf-4	Q9Z2U4
AAVENLPTFL	10	12.3	Importin subunit beta-1	P70168
SAYQNHLVL	9	12.3	Anoctamin-10	Q8BH79; Q8BH79-3; Q8BH79-2; Q8BH79-4
SAPENAVRM	9	12.4	Protein C10	O35127
GAIRNACQM	9	12.4	Cullin-3	Q9JLV5
KAISNSHYVL	10	12.4	autophagy-related protein 2 homolog B	Q80XK6
SGLQNFEAL	9	12.4	Protein unc-45 homolog A	Q99KD5
YQLENIQVL	9	12.5	Citron Rho-interacting kinase	P49025-4; P49025; P49025-3; P49025-5
STVVNADQI	9	12.5	ATP-binding cassette sub-family B member 6, mitochondrial	Q9DC29
ASLVNHVLL	9	12.5	DNA excision repair protein ERCC-6-like	Q8BHK9
AAIGNQLYV	9	12.5	Rab9 effector protein with kelch motifs	Q8VCH5
VSPLNVTAV	9	12.5	WASH complex subunit 3	Q9CR27
YSMLNAYSNL	10	12.5	Probable ribonuclease ZC3H12C	Q5DTV4
ASLKNLCEM	9	12.5	Zinc finger SWIM domain-containing protein 3	Q8CFL8
FSPTNPAHL	9	12.6	WD repeat-containing protein 76	A6PWY4-1; A6PWY4-3; A6PWY4-2
SQLQNEFYL	9	12.6	Collagen alpha-2(VI) chain	Q02788
LSVLNHHFI	9	12.7	GPI inositol-deacylase	Q3UUQ7-1
SAIHNSTKV	9	12.8	Interferon-induced very large GTPase 1	Q80SU7
YALENFVENL	10	12.8	Importin-4	Q8VI75
VAPVNLQHDFI	11	13	Fanconi anemia group D2 protein homolog	Q80V62
YASTNLEWL	9	13	Cytochrome c oxidase subunit 1	P00397
GSLANHTSI	9	13.1	Vacuolar protein sorting-associated protein 33A	Q9D2N9
SALENGRYEL	10	13.1	Bromodomain adjacent to zinc finger domain protein 1A	O88379
IGIENIHYL	9	13.2	malignant T-cell-amplified sequence 2	Q9DB27-2; Q9CQ21; Q9DB27
YSLVNQQSF	9	13.2	ras-related protein Rap-2b	Q8BU31; P61226; Q80ZJ1-2; Q80ZJ1
AQHVNLAVL	9	13.2	GON-4-like protein	Q9DB00
STPVNVTSL	9	13.2	Kinesin-like protein KIF23	E9Q5G3
VAYMNPIAM	9	13.3	Protein FAM133B	Q9CVI2; Q9CVI2-2
QSLRNIHTL	9	13.3	E3 ubiquitin-protein ligase synoviolin	Q9DBY1-2; Q9DBY1
YTIQNRVDI	9	13.5	Cohesin subunit SA-1	Q9D3E6
YSGANSIFL	9	13.5	Bystin	O54825
GALSNLEQL	9	13.5	Volume-regulated anion channel subunit LRRC8B	Q5DU41; Q5DU41-2
SSLVNGSTF	9	13.5	Magnesium transporter NIPA1	Q8BHK1
TTLTNSTPL	9	13.6	Protein fem-1 homolog C	Q8CEF1
LSVRNGATL	9	13.6	Peroxisome proliferator-activated receptor gamma coactivator 1-beta	Q8VHJ7-2; Q8VHJ7
FQWRNLNTI	9	13.7	Protein asteroid homolog 1	Q8BIR2; Q8BIR2-2
SSVENYYVFL	10	13.7	Isoform 2 of Tetratricopeptide repeat protein 39B	Q8BYY4-2
NMPWNVDTL	9	13.7	Hsp90 co-chaperone Cdc37	Q61081
AALQNAVAF	9	13.7	E3 ubiquitin-protein ligase TRIM56	Q80VI1
ISGVNRYYV	9	13.8	NADH dehydrogenase [ubiquinone] 1 alpha subcomplex subunit 1	O35683
RSPFNACVL	9	13.9	glycerophosphodiester phosphodiesterase 1	Q9JL56
SAIFNFQSL	9	13.9	Protein kish-A	Q9CR64-1; Q9CR64-2
VSVLNVAVL	9	13.9	Histone chaperone ASF1B	Q9DAP7

LSLENIATL	9		14	Protein regulator of cytokinesis 1	Q99K43
ASLQNAEKTM	10		14.1	Ubiquitin carboxyl-terminal hydrolase 38	Q8BW70
AVLTNQETI	9		14.1	Brefeldin A-inhibited guanine nucleotide-exchange protein 3	Q3UGY8
YTLRNQDTF	9		14.2	period circadian protein homolog 1	O35973
VAVSNLEKV	9		14.2	Isoform 3 of Ewing's tumor-associated antigen 1 homolog	Q5SVT3-2; Q5SVT3-3; Q5SVT3
FALINKLDI	9		14.2	Arginyl-tRNA--protein transferase 1	Q9Z2A5-2; Q9Z2A5
STIKNANFV	9		14.2	ATP-binding cassette sub-family B member 10, mitochondrial	Q9JI39
HSGMNATTI	9		14.3	N-acetylglucosamine-1-phosphotransferase subunits alpha/beta	Q69ZN6; Q69ZN6-2
YSIGNLQKI	9		14.4	Origin recognition complex subunit 5	Q9WUV0
ASVLNVNHI	9		14.4	Ankyrin repeat domain-containing protein 17	Q99NH0-1
VQIKNDVFI	9		14.5	Kelch domain-containing protein 10	Q6PAR0-3; Q6PAR0-2; Q6PAR0
SLLSNLDEI	9		14.5	Programmed cell death 6-interacting protein	Q9WU78-3; Q9WU78
KAILNGIDSI	10		14.6	Structural maintenance of chromosomes protein 3	Q9CW03
AAPQNFTPSM	11		14.6	AP-3 complex subunit beta-1	Q9Z1T1
YAIKNIHGI	9		14.6	Dynamin-1	P39053; P39053-3; P39053-4; Q8BZ98; Q8BZ98-2; P39053-5; P39053-6
NALRNFACL	9		14.7	Ubiquitin recognition factor in ER-associated degradation protein 1	P70362
TSIPNFSYM	9	1xOxidation [M9]	14.7	Polyadenylate-binding protein-interacting protein 1	Q8VE62
AALLNTQPAI	10		14.8	Zinc finger protein 462 OS=Mus musculus OX=10090	B1AWL2
GAHVNAQTV	9		14.9	Ankyrin repeat and SOCS box protein 11	Q9CQ31-1; Q9CQ31-2
RAILNDTKL	9		14.9	Rab GTPase-binding effector protein 1	O35551-6; O35551-1; O35551-3; O35551-2
GSLRNLEEL	9		14.9	Baculoviral IAP repeat-containing protein 1f	Q9R016; Q9R016; Q9QWK5; Q9JIB6; Q9JIB3
FQHRNHTCL	9		14.9	Homeodomain-interacting protein kinase 3	Q9ERH7
YTVANKEYV	9		15	Endoplasmic reticulum-Golgi intermediate compartment protein 1	Q9DC16
KGIVNEQFL	9		15	Very long-chain specific acyl-CoA dehydrogenase, mitochondrial	P50544
YQYINCSTL	9		15	Mitotic checkpoint serine/threonine-protein kinase BUB1 beta	Q9Z1S0
SSPLNLAHV	9		15.1	ocysteine-responsive endoplasmic reticulum-resident ubiquitin-like domain member 2 pro	Q9JJC9
KAHMNSVLM	9		15.1	Charged multivesicular body protein 3	Q9CQ10
AALLNFDEF	9		15.1	FH1/FH2 domain-containing protein 1	Q6P9Q4
NSQVNFDFI	9		15.1	Ral guanine nucleotide dissociation stimulator-like 1	Q60695
SGLLNPYSL	9		15.2	DNA helicase ino80	Q6ZPV2-2; Q6ZPV2-1
TSVSNFLVM	9		15.2	Alsin	Q920R0-2; Q920R0
NSLTNIQWL	9		15.3	Forkhead box protein M1	O08696
RQFRNPDTI	9		15.3	IQ motif and SEC7 domain-containing protein 2	Q8R0S2-2; Q5DU25; Q8R0S2
KSMENLIEL	9		15.4	Tubby-related protein 3	O88413
NSLVNICLI	9		15.5	E3 ubiquitin-protein ligase RNF26	Q8BUH7
TQIRNRALI	9		15.5	COP9 signalosome complex subunit 1	Q99LD4; Q99LD4-2
GAIENAFHL	9		15.6	Protein phosphatase 1J	Q149T7
RAAANSETI	9		15.6	GON-4-like protein	Q9DB00
SSPRNSDWF	9		15.6	Serine/threonine-protein kinase ULK2	Q9QY01
AALENPMLL	9		15.8	Ubiquitin-like modifier-activating enzyme ATG7	Q9D906
IGVSNFNPL	9		15.8	Aldo-keto reductase family 1 member B1	P45376
VSLNNLIYV	9		15.8	Kelch-like protein 24	Q8BRG6
SAVENKDKL	9		15.9	SprT-like domain-containing protein Spartan	G3X912
CQIANIYEL	9		15.9	intraflagellar transport protein 88 homolog	Q61371
YTFVNPSVL	9		15.9	Plexin-A2	P70207
QAHLNLETL	9		16	N-acylethanolamine-hydrolyzing acid amidase	Q9D7V9
ISWHNIDYI	9		16	unconventional myosin-IXa	Q8C170-2; Q8C170-1

AMNVNEIFM	9		16.1	Ras-related protein Rab-5C	P35278
GSIVNEGYL	9		16.2	Synaptogyrin-1	O55100; O55100-2
ISIMNGSSV	9		16.3	Polyadenylate-binding protein-interacting protein 2B	Q91W45
YAFYNTEQL	9		16.5	F5-like RNA polymerase II p300/CBP-associated factor-associated factor 65 kDa subunit	Q91WQ5
TALLNAPHI	9		16.6	MAGUK p55 subfamily member 5	Q9JLB2
SAMSNPRAM	9		16.7	Isoform 2 of Ubiquilin-1	Q8R317-2; Q8R317
RSFENNTYL	9		16.7	RecQ-mediated genome instability protein 1	Q9D4G9
YSGVNVTDL	9		16.7	[F-actin]-monooxygenase MICAL3	Q8CJ19-3; Q8CJ19; Q8CJ19-2
FSYLNPQEL	9		16.8	F-box/LRR-repeat protein 5	Q8C2S5-3; Q8C2S5-4; Q8C2S5; Q8C2S5-2; Q8C2S5-5
FIIENTDTV	9		16.8	Interferon alpha/beta receptor 1	P33896
SGPSNKDCI	9		16.9	Cysteine-rich with EGF-like domain protein 2	Q9CYA0
YGIQNSYVKL	10		16.9	Vacuolar protein sorting-associated protein 33A	Q9D2N9
SSPENKNWL	9		16.9	Denticleless protein homolog	Q3TLR7-2; Q3TLR7
GSLSNHTEL	9		17	Tonsoku-like protein	Q6NZL6
KMIINEELM	9	1xOxidation [M9]	17.1	Eukaryotic translation initiation factor 3 subunit C	Q8R1B4
SALANGRAL	9		17.1	Homeobox protein DLX-1	Q64317
YQHINSYQL	9		17.1	ated matrix-associated actin-dependent regulator of chromatin subfamily A containing DE	Q04692-2; Q04692
QAVLNLHSI	9		17.1	DIS3-like exonuclease 2	Q8CI75-2; Q8CI75
AALNNHVEV	9		17.1	E3 ubiquitin-protein ligase MIB1	Q80SY4
YSPQNSDYLL	10		17.2	Sortilin	Q6PHU5; Q6PHU5-2
RMLTNAMEV	9		17.4	Serine/threonine-protein kinase mTOR	Q9JLN9
IALFNQELV	9		17.4	Isoform 1 of Paired amphipathic helix protein Sin3b	Q62141-1; Q62141
GGVVNMYHM	9		17.5	Proteasome subunit beta type-8	P28063
SSIVNSIQV	9		17.5	Ribosome-binding protein 1	Q99PL5-10; Q99PL5-11; Q99PL5-6; Q99PL5-12; Q99PL5-4; Q99PL5-9; Q99PL5-7; Q99PL5-1; Q99PL5-8; Q99PL5-5; Q99PL5-3; Q99PL5-2
SSIRNFLIYV	10		17.5	mitochondrial import receptor subunit TOM5 homolog	B1AXP6-2; B1AXP6-1; B1AXP6-4; B1AXP6-3
KATINIDAI	9		17.6	Fas apoptotic inhibitory molecule 1	Q9WUD8
RGIVNLSEL	9		17.6	Decaprenyl-diphosphate synthase subunit 2	Q33DR3; Q33DR3-2; Q33DR3-3
DAVKNGDYI	9		17.6	M-phase phosphoprotein 8	Q3TYA6
STIRNAQSI	9		17.7	Neutrophil cytosol factor 1	Q09014
SQPVNPHSL	9		17.7	Zinc finger SWIM domain-containing protein 8	Q3UHH1-2; Q3UHH1; Q3UHH1-3; Q3UHH1-4
GSLINPNVI	9		17.8	EKC/KEOPS complex subunit Tprkb	Q8QZZ7
GANVNHTTV	9		17.9	Protein fem-1 homolog B	Q9Z2G0
FQVRNLPTL	9		18	Uncharacterized protein C12orf29 homolog	Q8BHN7; Q8BHN7-2
SAVKNLQQL	9		18	GATOR complex protein NPRL3	Q8VIJ8
TQMVNVYHV	9		18.1	transportin-3	Q6P2B1-1; Q6P2B1-2
TQVLNTHYV	9		18.1	Transcription activator BRG1	Q3TKT4-1; Q6DIC0; Q3TKT4-2
SAHQNYAEWL	10		18.1	Ribosome-binding protein 1	Q99PL5-1
GAFSNPETL	9		18.1	Acetoacetyl-CoA synthetase	Q9D2R0
VSIQNLVKV	9		18.1	ATP-binding cassette sub-family A member 1	P41233
TTVTNSTPL	9		18.1	Protein fem-1 homolog B	Q9Z2G0
FALKNPFYSL	10		18.2	Trafficking protein particle complex subunit 4	Q9ES56
YSFPNPEWL	9		18.2	MAP kinase-activated protein kinase 3	Q3UMW7
RGLENLTLL	9		18.3	Toll-like receptor 13	Q6R5N8
FSFRNTQEV	9		18.3	Interferon-induced very large GTPase 1	Q80SU7

NGVINAAFM	9		18.4	Clathrin coat assembly protein AP180	Q61548; Q7M6Y3-1; Q7M6Y3-5; Q61548-2; Q7M6Y3-6; Q7M6Y3-4; Q7M6Y3-2; Q7M6Y3-3; Q61548-3
AALVNPDSF	9		18.4	Zinc fingers and homeoboxes protein 1	P70121
GSIINISSI	9		18.4	Estradiol 17-beta-dehydrogenase 8	P50171; P50171-2
SAMVSIEYV	9		18.4	E3 ubiquitin-protein ligase RNF149	Q3U2C5
TQPLNHYFI	9		18.5	1-phosphatidylinositol 4,5-bisphosphate phosphodiesterase beta-2	A3KGF7; A3KGF7-3; A3KGF7-4; A3KGF7-2
SGPTNEDLYI	10		18.6	3-hydroxy-3-methylglutaryl-coenzyme a reductase	Q01237
HALVNIRAI	9		18.6	Armadillo-like helical domain-containing protein 3	Q6PD19; Q6PD19-2
GAVKNLTYF	9		18.7	CMP-N-acetylneuraminate-beta-galactosamide-alpha-2,3-sialyltransferase 4	Q91Y74
ASAINWTLI	9		18.7	Cytosolic 10-formyltetrahydrofolate dehydrogenase	Q8R0Y6; Q8K009
DALTNLTVL	9		18.7	Protein phosphatase 1 regulatory subunit 7	Q3UM45
IMVRNIDLV	9		18.8	vesicle-associated membrane protein 7	P70280
FATSNQISL	9		18.8	COMM domain-containing protein 7	Q8BG94
FGLSNQLVV	9		18.8	Mucolipin-1	Q99J21-2; Q8K595-2; Q99J21; Q8K595
MALENNYEVI	10		18.8	NEDD4-binding protein 2-like 1	Q3V2Q8; Q3V2Q8-2
TTVTNPEPM	9		18.9	DNA (cytosine-5)-methyltransferase 1	P13864-2; P13864
AGPTNVHLI	9		19.1	homeobox protein SIX5	P70178
AQVRNQGYL	9		19.1	Centrosomal protein kizuna	Q3UXL4
SSPRNSQEL	9		19.2	caspase recruitment domain-containing protein 9	A2AIV8
FSLHNPYNL	9		19.2	T-box transcription factor TBX15	O70306
VSPTNPTKL	9		19.2	Disabled homolog 2-interacting protein	Q3UHC7-3; Q3UHC7-2; Q3UHC7
ASVLNTDPAL	10		19.2	dipeptidyl peptidase 3	Q99KK7
SAIHNFYDNI	10		19.2	TGF-beta-activated kinase 1 and MAP3K7-binding protein 2	Q99K90
RQIGNRDTL	9		19.3	Mitochondrial antiviral-signaling protein	Q8VCF0
NAVRNLQEL	9		19.3	Centrosomal protein of 70 kDa	Q6IQY5-2; Q6IQY5-1
AAVLNPRFL	9		19.3	C2 domain-containing protein 5	Q7TPS5-3; Q7TPS5-2; Q7TPS5
AQILNQVMM	9		19.4	Mitotic checkpoint serine/threonine-protein kinase BUB1	O08901
FSNKNLEEL	9		19.4	DIS3-like exonuclease 1	Q8C0S1; Q8C0S1-2
SSVQNGIML	9	1xOxidation [M8]	19.6	E3 ubiquitin-protein ligase Praja-2	Q80U04; Q80U04-2
FSVVNPASF	9		19.6	Rho-related GTP-binding protein RhoQ	Q8R527
FSTWNLDHI	9		19.7	DNA fragmentation factor subunit beta	O54788
AALVSRTFL	9		19.7	ATP-binding cassette sub-family D member 1	P48410
SSLVNKEDVL	10		19.7	Nodal modulator 1	Q6GQT9
SALHNDLQI	9		19.7	Ubiquitin-conjugating enzyme E2 Q1	Q7TSS2-2; Q7TSS2
RALKNFSSL	9		19.8	Ral guanine nucleotide dissociation stimulator	Q03385
SAVRNHTYQM	11		19.8	FTS and Hook-interacting protein	Q3U2I3-3; Q3U2I3-2; Q3U2I3
AALENDKTI	9		19.9	WD repeat-containing protein 5	P61965; Q9D7H2
AMGVNLTSM	9	1xOxidation [M]	19.9	proliferating cell nuclear antigen	P17918
RAVANRTDAC	12		20	26S proteasome regulatory subunit 7 OS=Mus musculus OX=10090	P46471
AGIYNTTEL	9		20	Ubiquinone biosynthesis protein COQ9, mitochondrial	Q8K1Z0
KSYVNPTEL	9		20.1	Ral GTPase-activating protein subunit beta	Q8BQZ4-1
NQLKNTSTI	9		20.1	Probable ATP-dependent RNA helicase DDX6	P54823
SVLFNLTTM	9		20.1	Trafficking protein particle complex subunit 12	Q8K2L8
FGLQNFPEL	9		20.2	Uroporphyrinogen decarboxylase	P70697
FALNNPEMV	9		20.3	Protein NDRG1	Q62433
SMAENSIPL	9		20.3	Forkhead box protein P1	P58462-3; P58462-4; P58462; P58462-2; P58462-5
KGLLNAIVI	9		20.3	Ornithine aminotransferase, mitochondrial	P29758

SGVWNVTEL	9	20.4	Nuclear factor 1 X-type	P70257; P70257-2; P70257-1
TALKNPNAM	9	20.5	Ras GTPase-activating-like protein IQGAP1	Q9JKF1
SQALNTTVL	9	20.5	Triggering receptor expressed on myeloid cells 2	Q99NH8-1; Q99NH8-2
SAHQNGERI	9	20.5	Cytoplasmic polyadenylation element-binding protein 2	Q812E0
RAHRNDMETI	10	20.5	Prostaglandin E synthase	Q9JM51
YAADNQIYI	9	20.5	Heterogeneous nuclear ribonucleoprotein L	Q8R081
YMGTNIHSL	9	20.7	Inositol polyphosphate 1-phosphatase	P49442
SGLFNCVTI	9	20.7	Calcium channel flower homolog	Q8BG21-3; Q8BG21-2; Q8BG21
SALINLVEF	9	20.7	DNA-dependent protein kinase catalytic subunit	P97313; P97313-2
FVLENFSTL	9	20.9	E3 ubiquitin-protein ligase TRIM37	Q6PCX9
AAATNPSWL	9	21	Integrin alpha-X	Q9QXH4
SGLENTAAL	9	21	Melanoma inhibitory activity protein 2	Q91ZV0; Q91ZV0-3
MGLSNVTVV	9	21.2	Up-regulator of cell proliferation	Q5NCI0-2; Q5NCI0
WAILNEIHI	9	21.4	alpha-1,3-mannosyl-glycoprotein 4-beta-N-acetylglucosaminyltransferase A	Q812G0
VSYRNIEEL	9	21.5	Nucleoprotein TPR	F6ZDS4
TALNNPYYYL	10	21.5	Origin recognition complex subunit 3	Q9JK30-2; Q9JK30-1
AGTRNIYYL	9	21.6	heat shock protein 75 kDa, mitochondrial	Q9CQN1
SSTANIIVM	9	21.6	Solute carrier family 12 member 2	P55012
ASLVNSPSYL	10	21.7	Transmembrane protein 131-like	Q3U3D7-2; Q3U3D7-1; Q3U3D7-3
YMPQNPHII	9	21.8	Histone-binding protein RBBP7	Q60973
SSIVNSIQVL	10	21.8	Ribosome-binding protein 1	Q99PL5-10; Q99PL5-11; Q99PL5-6; Q99PL5-12; Q99PL5-4; Q99PL5-9; Q99PL5-7; Q99PL5-1; Q99PL5-8; Q99PL5-5; Q99PL5-3; Q99PL5-2
KQIANIDRI	9	21.9	Zinc finger MYND domain-containing protein 11	Q8R5C8
AAMSNPRAM	9	21.9	Ubiquilin-2	Q9QZM0
AMLENQTPEL	10	22	Fatty acid synthase	P19096
TSIKNQTQL	9	22.1	Pseudouridylate synthase 7 homolog OS=Mus musculus OX=10090	Q91VU7
VQPFNYVTL	9	22.1	Protein fem-1 homolog A-B	Q8C0T1; Q9Z2G1
IANINQELI	9	22.4	F-box only protein 38	Q8BMI0
YMLKNVRVL	9	22.5	Neuropathy target esterase	Q3TRM4; Q3TRM4-2; Q3TRM4-4; A2AJ88-3; A2AJ88; A2AJ88-2; Q3TRM4-3
VMMENFHHI	9	22.5	exocyst complex component 1	Q8R3S6
NANTNTEVL	9	22.5	Charged multivesicular body protein 4b	Q9D8B3
SALVNNTIF	9	22.5	Endoribonuclease Dicer	Q8R418; Q8R418-2
SAVENKQQI	9	22.6	Serine/threonine-protein kinase PLK2	P53351
FALKSLTYL	9	22.6	Protein-tyrosine sulfotransferase 1	O70281
TALQNNTVYL	10	22.6	ICOS ligand	Q9JHJ8-2; Q9JHJ8
RSIRNILDI	9	22.6	Tumor necrosis factor alpha-induced protein 2	Q61333
LAVRNDEEL	9	22.7	Histone H2A type 2-B	Q64522
AQLRNTQWV	9	22.8	Phospholipid-transporting ATPase IA	P70704-3; P70704-2; P70704-1
GMIENGPFL	9	22.8	Interferon-induced very large GTPase 1	Q80SU7
VAVVNKVDI	9	22.8	NPC intracellular cholesterol transporter 1	O35604
RAIKNDSVV	9	22.9	T-complex protein 1 subunit eta	P80313
SALTNCITQL	10	23	Melanoma inhibitory activity protein 3	Q8BI84-1
FSLSKYTPL	9	23	Sodium- and chloride-dependent betaine transporter	P31651
SSGKNLFYL	9	23.1	Peptidyl-glycine alpha-amidating monooxygenase	P97467
KSITNPRYV	9	23.2	DNA-directed RNA polymerase I subunit RPA1	O35134
GAVQNIAHL	9	23.2	Armadillo repeat-containing protein 8	Q9DBR3; Q9DBR3-3; Q9DBR3-2

YQPYNKDWI	9		23.3	Enhancer of rudimentary homolog	P84089
SQIGNLEAL	9		23.3	Leucine-rich repeat and calponin homology domain-containing protein 3	Q8BVU0
FKIINHSFI	9		23.4	cytochrome b	P00158
TGVTNRDLI	9		23.4	rho GTPase-activating protein 21	Q6DFV3
SMEVNVDTL	9		23.4	Dysbindin domain-containing protein 2	Q9CRD4
WAVSNREML	9		23.4	Protein RMD5 homolog A	Q80YQ8
SAVVDKDFL	9		23.6	Adenosine kinase	P55264; P55264-2
GAPINVMFL	9		23.6	Acid sphingomyelinase-like phosphodiesterase 3b	P58242
YALEHMITL	9		23.7	Kinetochore-associated protein 1	Q8C3Y4
FSIENSHMCM	10		23.7	Protein aurora borealis	Q8BS90-1
SSGINGSFL	9		23.7	Tyrosine-protein kinase ABL1	P00520-4; P00520-3; P00520-2; P00520
SAIQNLHSFDP	12		23.7	Eukaryotic translation initiation factor 1	P48024
TSVQNVSQI	9		23.8	C-type lectin domain family 5 member A	Q9R007-3; Q9R007-2
DAIHNFDFL	9		23.9	probable cation-transporting ATPase 13A3	Q5XF89-2; Q5XF89-1
HQLQNFNTL	9		23.9	RAS guanyl-releasing protein 1	Q8BTM9-4; Q8BTM9-2; Q8BTM9-3; Q8BTM9-1; Q9Z1S3
GSIANFTNV	9		23.9	ATP-citrate synthase	Q91V92
IQHSNIVTL	9		24	PAN2-PAN3 deadenylation complex subunit Pan3	Q640Q5
VMLENYNHL	9		24.2	Zinc finger protein 90	Q61967
SGLRNWTTA	9		24.2	Selenoprotein N	D3Z2R5
VAIINMPLTL	10		24.2	adenylate cyclase type 7	P51829
AAIRNYGIEL	10		24.3	Methylenetetrahydrofolate reductase	Q9WU20
GGLRNVDCL	9		24.4	Serine/threonine-protein kinase TBK1	Q9WUN2
YSYQNRYHF	9		24.5	CREB-binding protein	P45481; B2RWS6
SAIMNPASKVI	11		24.6	Clathrin heavy chain 1	Q68FD5
LQLQNLATL	9		24.6	CUGBP Elav-like family member 2	Q9Z0H4; Q9Z0H4-7; Q9Z0H4-11; Q9Z0H4-9; Q9Z0H4-4; Q9Z0H4-8; Q9Z0H4-6; Q9Z0H4-2; Q9Z0H4-3; Q9Z0H4-5
YVLTNVAYF	9		24.7	Cystine/glutamate transporter	Q9WTR6
NAIINTLAV	9		24.7	Pleiotropic regulator 1	Q922V4
KSVINTTLV	9		24.7	Lysosome membrane protein 2	O35114
KAIHNICSV	9	Carbamidomethyl [C	24.9	transportin-3	Q6P2B1-1; Q6P2B1-2
LALVNDQLL	9		24.9	Sister chromatid cohesion protein PDS5 homolog A	Q6A026
HSPTNTVHM	9		24.9	ubiquitin carboxyl-terminal hydrolase 8	Q80U87
LSPINHNTL	9		24.9	Rapamycin-insensitive companion of mTOR	Q6QI06; Q6QI06-2
RMMHSNMETL	10		24.9	Protein diaphanous homolog 1	O08808
RSAENFTVL	9		24.9	P2X purinoceptor 7	Q9Z1M0
ASHLNNVFV	9		25	exocyst complex component 1	Q8R3S6
SAIFNNVMTL	10	1xOxidation [M8]	25	Equilibrative nucleoside transporter 1	Q9JIM1-1; Q9JIM1-2
VMLENYSHL	9		25.1	Zinc finger protein 30	P16372; Q60585; Q62396
AANRNNDAL	9		25.2	Vimentin	P20152
KQLYNLDII	9		25.4	WD repeat-containing protein 75	Q3U821
ISLKNSQEI	9		25.4	Interferon-induced very large GTPase 1	Q80SU7
QSVVNLQEM	9		25.4	Zinc finger homeobox protein 3	Q61329
AMNVNDLFL	9		25.4	Ras-related protein Rab-5B	P61021
VALDNSVYL	9		25.5	Cell division cycle protein 20 homolog	Q9JJ66
SAPVNFISA	9		25.5	Tuftelin-interacting protein 11	Q9ERA6
FQHKNHTCL	9		25.6	Homeodomain-interacting protein kinase 1	O88904-1; Q9QZR5

HAIRNSFQYL	10		25.6	Myotubularin-related protein 4	Q91XS1; Q91XS1-2
SSLHNEQVL	9		25.6	Membrane-bound transcription factor site-2 protease	Q8CHX6
YVLHNSNTM	9		26.1	26S proteasome non-ATPase regulatory subunit 2	Q8VDM4
NSLLNQGFL	9		26.3	Myb-related protein B	P48972
KATTNIVEM	9		26.4	T-complex protein 11-like protein 2	Q8K1H7
AAQTNLTLL	9		26.5	Homeobox protein PKNOX1	O70477; Q8BG99
QQVVNIECI	9		26.7	AP-2 complex subunit alpha-2	P17427
SSATNRITV	9		26.9	E3 SUMO-protein ligase PIAS4	Q9JM05
FQYRNRIAM	9		27.1	Prostaglandin G/H synthase 1	P22437
RGILNWCVV	9	Carbamidomethyl [(	27.2	Diacylglycerol O-acyltransferase 1	Q9Z2A7
RSMQNYVQFL	10		27.2	Outer dense fiber protein 2	A3KGV1; A3KGV1-4; A3KGV1-3; A3KGV1-2
SAASNPFYF	9		27.2	Lysosomal-trafficking regulator	P97412-2; P97412
SAPRNFVENFS	13		27.5	Elongator complex protein 2	Q91WG4-2; Q91WG4
YTLHNIDQL	9		27.6	Zinc finger FYVE domain-containing protein 16	Q80U44
FMISHLDYL	9		27.7	Serine-protein kinase ATM	Q62388
VGIRNTFLL	9		27.8	Poly(A) RNA polymerase GLD2	Q91YI6-1; Q91YI6-2
RSLLNSHIL	9		27.8	WD repeat and FYVE domain-containing protein 3	Q6VNB8
YAGSNREDVL	10		27.8	26S proteasome non-ATPase regulatory subunit 2	Q8VDM4
SSIHNPTGRSY	13		27.8	Protein arginine N-methyltransferase 5	Q8CIG8
WSLANHEYF	9		28	Engulfment and cell motility protein 1	Q8BPU7-3; Q8BPU7-1
YQILNNEVF	9		28.1	Cytoplasmic FMR1-interacting protein 2	Q5SQX6
VGIENFELL	9		28.2	Ribosomal protein S6 kinase alpha-5	Q8C050-1; Q8C050-2
SQISNTEFLQV	12		28.2	BRISC complex subunit Abraxas 2	Q3TCJ1
KAVTNEQEL	9		28.4	Transcription termination factor 1b, mitochondrial	Q8CHZ9; B9EJ57
SAIVNFTLQHL	11		28.4	Transcription factor E2F8	Q58FA4
AAPINPSDI	9		28.5	Enoyl-[acyl-carrier-protein] reductase, mitochondrial	Q9DCS3
SSVFNVEMV	9		28.5	ATP synthase mitochondrial F1 complex assembly factor 1	Q811I0
RQLENTEHM	9		28.7	Structure-specific endonuclease subunit SLX4	Q6P1D7
GALKNIVAV	9		28.7	Glycerol-3-phosphate dehydrogenase 1-like protein	Q3ULJ0-2; P13707; Q3ULJ0
SALCNSCRL	9		28.9	Talin-1	P26039
VTNLNPDFI	9		29	polypyrimidine tract-binding protein 3	Q8BHD7-2; Q8BHD7
EAIVNQEII	9		29	Xylosyltransferase 1	Q811B1
ISGVNGTHI	9		29.1	SRSF protein kinase 1	O70551
ISLHNPVSI	9		29.1	Probable ATP-dependent RNA helicase DDX31	Q6NZQ2
YGGSNYVVV	9		29.1	Nucleoporin Nup37	Q9CWU9
KAPTNVTCIL	10		29.2	von Willebrand factor A domain-containing protein 8	Q8CC88-2; Q8CC88
SAPSCWTTL	9		29.3	Vacuolar protein sorting-associated protein 16 homolog	Q920Q4
CAYLNRHWV	9		29.4	Cullin-1	Q9WTX6
YVHVNQYDI	9		29.4	Coatomer subunit beta	Q9JIF7
RIIENVDVI	9		29.4	Actin-related protein 8	Q8R2S9
MQLQNLAAL	9		29.4	CUGBP Elav-like family member 1	P28659-2; P28659; P28659-3; P28659-4
ASIQNGKDSL	10		29.7	Serine/threonine-protein kinase MARK2	Q05512-2; Q05512
KAKVNQETI	9		29.8	SS18-like protein 2	Q9D174
LSHVNEEWL	9		30	Neutrophil cytosol factor 2	O70145
VSLVNQRDEL	10		30	EH domain-binding protein 1-like protein 1	Q99MS7-3; Q99MS7; Q99MS7-4; Q99MS7-5; Q99MS7-2
YSIKCFDTV	9		30.1	TBC1 domain family member 31	Q6NXY1
YEIENQNPL	9		30.2	Nuclear fragile X mental retardation-interacting protein 2	Q5F2E7-2; Q5F2E7-1

IQIGNHNYM	9		30.2	Receptor-interacting serine/threonine-protein kinase 1	Q60855
GAACNVWYL	9		30.2	Tensin-3	Q5SSZ5; Q5SSZ5-2
YTVKNGDSL	9		30.5	Trafficking protein particle complex subunit 10	Q3TLI0
FGLQNDHCV	9		30.6	Transforming acidic coiled-coil-containing protein 3	Q9JJ11; Q9JJ11-2
AQQVNRTTL	9		30.7	Transcription initiation factor TFIID subunit 6	Q62311
AALQNYNNEL	10		31	Sjoegren syndrome nuclear autoantigen 1 homolog	Q9JJ94
SMEVNVDVL	9		31.1	Isoform 2 of Dysbindin	Q91WZ8-2; Q91WZ8-3; Q91WZ8-1
FSFRNTQEVI	10		31.2	Interferon-induced very large GTPase 1	Q80SU7
SQNRNGEFV	9		31.3	Pre-mRNA-splicing factor 38A	Q4FK66-1
VSVENFALL	9		31.3	Ribosomal protein S6 kinase alpha-4	Q9Z2B9
ASLTNPFGKG/	13		31.4	Nucleolar GTP-binding protein 2	Q99LH1
YSHVDLVVM	9		31.5	Serine--tRNA ligase, cytoplasmic	P26638
GSVVNSVAL	9		31.5	YTH domain-containing family protein 1	P59326
QTLKNITFI	9		31.7	Macrophage scavenger receptor types I and II	P30204-2; P30204
CMLTNPELL	9		31.8	Gamma-tubulin complex component 2	Q921G8
AAVVNFEEAL	10		31.8	Exportin-2	Q9ERK4
TSAQNVEFL	9		31.8	Outer dense fiber protein 2	A3KGV1; A3KGV1-4; A3KGV1-3; A3KGV1-2
FGGSNVHVI	9		32	Fatty acid synthase	P19096
YSIQGGQHTI	9		32.1	Poly(RC)-binding protein 1	P60335
AVLTNPETL	9		32.1	Multifunctional procollagen lysine hydroxylase and glycosyltransferase LH3	Q9R0E1
GAIGNPEVL	9		32.2	Deoxyhypusine hydroxylase	Q99LN9-2; Q99LN9
TALKNSTMV	9	1xOxidation [M8]	32.5	Synembryn-B	Q80XE1; Q80XE1-1
KSVENFVSL	9		32.5	Dedicator of cytokinesis protein 2	Q8C3J5
GSVSNIYKL	9		32.9	cytoplasmic FMR1-interacting protein 1	Q7TMB8-2; Q7TMB8-1
HSVQNTEYF	9		33.1	Bloom syndrome protein homolog	O88700
SGPMNEILI	9		33.1	PHD finger protein 19	Q9CXG9
TALWNPVSL	9		33.2	Genetic suppressor element 1	Q3U3C9-4; Q3U3C9-3; Q3U3C9-2; Q3U3C9
AGHRNREVL	9		33.2	Tudor domain-containing protein 3	Q91W18-1; Q91W18-3; Q91W18-2
VSNLNRQFL	9		33.4	SUMO-activating enzyme subunit 2	Q8C878; Q8C878-2; Q9Z1F9
NSPVNQPAM	9		33.4	Histone acetyltransferase p300	B2RWS6
KQILNIYDL	9		33.5	Vacuolar protein sorting-associated protein 11 homolog	Q91W86
SSPSNKFFF	9		33.5	Histone-lysine N-methyltransferase SETD2	E9Q5F9-2; E9Q5F9
SSIINFGALI	10		33.5	Hydrocephalus-inducing protein	Q80W93
AAVRNCDEF	9		33.7	Cytoplasmic aconitate hydratase	P28271
RAQTNYTCV	9		33.8	Receptor-type tyrosine-protein phosphatase C	P06800
HMLNNPDIM	9		33.8	Isoform 2 of Ubiquilin-1	Q8R317-2; Q8R317
AAITNKYQLV	10		33.9	RNA polymerase I-specific transcription initiation factor RRN3	B2RS91
AVVLNATWL	9		33.9	Acyl-CoA desaturase 3	Q99PL7; P13011; Q99PL7-2
FSGHNLEYL	9		34	Glutathione-specific gamma-glutamylcyclotransferase 1	Q8R3J5
IGPKNYEFL	9		34	Sorting nexin-14	Q8BHY8
KSISNTSKL	9		34	protein elys	Q8CJF7
VAPLNLGMI	9	1xOxidation [M8]	34.3	U5 small nuclear ribonucleoprotein 200 kDa helicase	Q6P4T2
KAIENCEYA	9		34.3	Mothers against decapentaplegic homolog 2	Q62432
YQYQNIFGPL	10		34.3	Regulator of nonsense transcripts 1	Q9EPU0-1; Q9EPU0-2
SSLHNSLLL	9		34.5	Kinesin-like protein KIF1B	Q60575-1; Q60575-2
AAIEAMNGQYL	11		34.5	Splicing factor 3b subunit 4	Q8QZY9

NTHENLTYL	9		34.7	epiplakin	Q9QXS1-6; Q9QXS1-13; Q9QXS1-7; Q9QXS1-9; Q9QXS1-5; Q9QXS1-14; Q9QXS1-12; Q9QXS1-3; Q9QXS1-2; Q9QXS1-10; Q8R0W0; Q9QXS1-15; Q9QXS1-11; Q9QXS1-16; Q9QXS1-4; Q9QXS1-1; Q9QXS1-8
LMVHNWEYL	9		34.7	V-type proton ATPase subunit H	Q8BVE3
AAVSNLVRV	9		34.7	Vinculin	Q64727
YCITNRSHL	9		34.8	Lysine-specific demethylase 2A	P59997-1
SALVHLEVL	9		34.8	SUMO-specific isopeptidase USPL1	Q3ULM6-4; Q3ULM6-1; Q3ULM6-2; Q3ULM6-3
WSPTNLAYF	9		35.4	Leucine-rich repeat and WD repeat-containing protein 1	Q8BUI3
FAHTNIESLV	10		35.5	Protein disulfide-isomerase A3	P27773
SMNSNIWQI	9		35.7	TELO2-interacting protein 1 homolog	Q91V83
SAINSIVYI	9		35.7	Major facilitator superfamily domain-containing protein 1	Q9DC37
FMMTNKLDTAI	11		35.8	Transmembrane protein 33	Q9CR67
SSKLNYDTV	9		36.1	E3 ubiquitin-protein ligase RBBP6	P97868-3; P97868-1; P97868-2
AANINKESI	9		36.3	Aspartate--tRNA ligase, cytoplasmic	Q922B2
YGLVNPWVHV	10		36.3	Hermansky-Pudlak syndrome 3 protein homolog	Q91VB4
FGIRNGSRL	9		36.3	SUMO-activating enzyme subunit 2	Q9Z1F9
FSVGNHGCYI	10		36.4	Fas apoptotic inhibitory molecule 1	Q9WUD8
SSVSNVNLTL	10		36.5	Nuclear receptor coactivator 2	Q61026
ASFSNGPVL	9		36.6	Nuclear fragile X mental retardation-interacting protein 2	Q5F2E7-2; Q5F2E7-1
KGIHNFESI	9		36.7	Erythroid differentiation-related factor 1	Q6GQV7
SSPTNGMKTM	10		36.7	Beta-mannosidase	Q8K2I4
AAIENIEHLL	10		36.7	Leucine-rich PPR motif-containing protein, mitochondrial	Q6PB66
SGLLNSQAL	9		36.7	Fatty acid synthase	P19096
ATIINEEVL	9		36.7	Signal recognition particle 54 kDa protein	P14576-2; P14576
FSHKNWVVF	9		36.8	von Willebrand factor A domain-containing protein 8	Q8CC88
AAPGNKTSYI	10		36.8	Probable 28S rRNA (cytosine-C(5))-methyltransferase	Q8K4F6
KSIKNIQKI	9		37	ATP synthase subunit gamma, mitochondrial	Q91VR2
QSPLNPCVI	9		37	Phosphatidylinositol 4,5-bisphosphate 3-kinase catalytic subunit beta isoform	Q8BTI9
VAASNIVQM	9		37.1	Proteasome subunit beta type-2	Q9R1P3
FMATNPEHL	9		37.2	ER degradation-enhancing alpha-mannosidase-like protein 3	Q2HXL6
SGPINFTVF	9		37.2	Myosin regulatory light chain 2, skeletal muscle isoform	P97457
KAIVNVIGM	9	1xOxidation [M9]	37.3	splicing factor 3B subunit 1	Q99NB9
AAGINVEII	9		37.3	Inverted formin-2	Q0GNC1; Q0GNC1-3
FAPKNIYSIM	10		37.3	Macrophage colony-stimulating factor 1 receptor	P09581
SSIVNKEGLNS	13		37.4	Tropomodulin-1	P49813
SSLVNKEDV	9		37.5	Nodal modulator 1	Q6GQT9
CSLQNKLVI	9		37.7	ZW10 interactor	Q9CQU5
YGLTNNSKL	9		37.8	cell division cycle protein 16 homolog	Q8R349
TQVTNQHLL	9		37.8	Neuronal PAS domain-containing protein 2	P97460
RTNVNFQYI	9		37.9	Glycerol-3-phosphate acyltransferase 3	Q8C0N2
FAYEGRDYI	9		38	H-2 class I histocompatibility antigen, D-B alpha chain	P14431; P14429; P14430; P01899
TSLINSADVL	10		38.2	DNA-directed RNA polymerase III subunit RPC7	Q6NXY9
AAGINRDSL	9		38.3	Carbonyl reductase family member 4	Q91VT4
RAIHNIFRM	9		38.5	DCC-interacting protein 13-alpha	Q8K3H0; Q8K3G9
NTLNNIDVI	9		38.5	Cytosolic phospholipase A2	P47713
FAVSNGYLVSL	11		38.5	Equilibrative nucleoside transporter 2	Q61672

NALLNSENL	9	38.6	ATPase family AAA domain-containing protein 5	Q4QY64; Q4QY64-2
SSPTSTEYI	9	38.7	Glucocorticoid modulatory element-binding protein 2	P58929
YAIKNIHGV	9	38.9	Dynamin-2	P39054-1; P39054-2
SAPRDAYWI	9	38.9	Cell division cycle protein 123 homolog	Q8CII2; Q8CII2-2
YLMKNADYF	9	39.1	OTU domain-containing protein 5	Q3U2S4-2; Q3U2S4
SSLLNNKHFL	10	39.1	Plexin-D1	Q3UH93
RALINKHTF	9	39.3	Putative Polycomb group protein ASXL3	Q8C4A5-2; Q8C4A5; Q8BZ32
KMLSNKESL	9	39.4	ubiquitin-like protein 7	Q91W67
FMYKNLQCL	9	39.4	ATP-dependent RNA helicase DDX18	Q8K363
RAVDNQVYV	9	39.5	Omega-amidase NIT2	Q9JHW2
SQIKNEINI	9	39.5	ER membrane protein complex subunit 1	Q8C7X2-2; Q8C7X2
SALENAENHV	10	39.8	uncharacterized protein C17orf53 homolog	Q32P12; Q32P12-2
SSVHNRNSL	9	39.9	Helicase with zinc finger domain 2	E9QAM5
IQLMNTAHL	9	40	peroxisomal carnitine O-octanoyltransferase	Q9DC50
QQPSNYGPM	9	40.1	heterogeneous nuclear ribonucleoproteins A2/B1	O88569-3; O88569; O88569-2
FTIFNRVDI	9	40.1	Cohesin subunit SA-2	O35638
AAPSSPTTM	9	40.1	zinc finger BED domain-containing protein 3	Q9D0L1
FGIHNGVETL	10	40.3	Glycerophosphocholine phosphodiesterase GPCPD1	Q8C0L9-1
SSCMNMHSI	9	40.4	anaphase-promoting complex subunit 1	P53995
VGVWNVPYI	9	40.5	Procollagen-lysine,2-oxoglutarate 5-dioxygenase 3	Q9R0E1; Q9R0E2
GMIINVLEL	9	40.5	Proteasome inhibitor PI31 subunit	Q8BHL8
AALRLLTYL	9	40.8	Treslin	Q8BQ33
ATVSNGPFL	9	40.9	E3 ubiquitin-protein ligase MSL2	Q69ZF8
RSLENRVTF	9	41	von Willebrand factor A domain-containing protein 5A	Q99KC8
TMAYNITPL	9	41	cold shock domain-containing protein E1	Q91W50
RTIRNQNTV	9	41.1	Protein MCM10 homolog	Q0VBD2
SSIEHLTTL	9	41.2	E3 ubiquitin-protein ligase HUWE1	Q7TMY8-4; Q7TMY8-3; Q7TMY8; Q7TMY8-2
ALLQNKDVI	9	41.2	Alpha-galactosidase A	P51569
YSPQNSDYLL/	12	41.2	Sortilin	Q6PHU5; Q6PHU5-2
RSIGNKNTI	9	41.3	hippocampus abundant transcript 1 protein	P70187
FAPVNVTTEVK	14	41.3	Elongation factor 1-alpha 1	P10126
KQLVNKEHL	9	41.6	Telomere-associated protein RIF1	Q6PR54-2; Q6PR54-3; Q6PR54-1
GQLANFQEM	9	41.6	AMP deaminase 2	Q9DBT5
VQLENQMVL	9	41.7	copper chaperone for superoxide dismutase	Q9WU84
FQLENVNKL	9	41.7	Golgi membrane protein 1	Q91XA2
KQVVNIPSFI	10	41.7	40S ribosomal protein S9	Q6ZWN5
FSELNSTEM	9	41.7	Coiled-coil and C2 domain-containing protein 1B	Q8BRN9
SSLVNGSTFVL	11	41.9	Magnesium transporter NIPA1	Q8BHK1
SASVNRYIL	9	42.1	e3 ubiquitin-protein ligase UBR4	A2AN08-2; A2AN08-3; A2AN08-5; A2AN08
YSIGNGSVF	9	42.1	Malate dehydrogenase, cytoplasmic	P14152
RAIEDADWI	9	42.2	LYR motif-containing protein 9	Q3UN90
AATFNPELI	9	42.3	Peroxisomal acyl-coenzyme A oxidase 1	Q9R0H0; Q9R0H0-2
AGPENIPEPM	10	42.4	Trafficking protein particle complex subunit 12	Q8K2L8
SAGRNALHL	9	42.4	Uveal autoantigen with coiled-coil domains and ankyrin repeats	Q8CGB3-3; Q8CGB3
GAICNSWKL	9	42.5	Mediator of RNA polymerase II transcription subunit 23	Q80YQ2-2; Q80YQ2
SQNFNEEFL	9	42.6	Dedicator of cytokinesis protein 7	Q8R1A4-2; Q8R1A4
QAIKNGQAL	9	42.8	Microtubule-actin cross-linking factor 1	Q9QXZ0-3; Q9QXZ0-2; Q9QXZ0-4; Q9QXZ0
RMPMNKNEL	9	42.9	beta-1,4-glucuronyltransferase 1	Q8BWP8-2; Q8BWP8

NSPRNLAMEA	13	43.3	Eukaryotic translation initiation factor 3 subunit D	O70194
TGVVNGESL	9	43.3	Protein HIRA	Q61666; Q61666-3; Q61666-2
FSEENHEPL	9	43.5	Vacuolar protein sorting-associated protein 35	Q9EQH3
YGPENTLPTL	10	43.5	G2/M phase-specific E3 ubiquitin-protein ligase	Q5RJY2
YGVSNLQEF	9	43.6	Adiponectin receptor protein 1	Q91VH1
VAPYNTTQFL	10	43.6	Protein HEXIM1	Q8R409
FALANHLIKV	10	43.8	EH domain-containing protein 1	Q9QXY6; Q9WVK4
AQVQNSEQL	9	43.8	Rab GTPase-binding effector protein 2	Q91WG2-2; Q91WG2; Q91WG2-1
SAVTNSGVHL	10	44.1	GTP-binding protein 8	Q9CY28-2; Q9CY28-1
AAISNKITSCI	11	44.5	Multifunctional protein ADE2 OS=Mus musculus OX=10090	Q9DCL9
TAVANNQAL	9	44.8	Calcitonin gene-related peptide type 1 receptor	Q9R1W5
RVPINETFI	9	44.8	WASH complex subunit 5	Q8C2E7
SAIMNPASKV	10	44.8	Clathrin heavy chain 1	Q68FD5
AAFRNFSDEI	10	44.8	Nuclear pore complex protein Nup93	Q8BJ71-1; Q8BJ71-2
VAIGNPVHL	9	45.3	Zinc finger homeobox protein 3	Q61329
TAVISYDYL	9	45.3	Uncharacterized aarF domain-containing protein kinase 1	Q9D0L4-1
FGGHNEDFI	9	45.9	WD repeat-containing protein 26	Q8C6G8
YSHQNARKI	9	46	Isoform 2 of PHD finger protein 20	Q8BLG0-2; Q8BLG0
DALSNVDLL	9	46	cytoplasmic FMR1-interacting protein 1	Q7TMB8-2; Q7TMB8-1; Q5SQX6
STMVNADHF	9	46.1	Phospholipid scramblase 4	P58196
GAVVNVADL	9	46.2	Tankyrase-2	Q3UES3
SAIINPPQACI	11	46.2	oyllysine-residue acetyltransferase component of pyruvate dehydrogenase complex, mitc	Q8BMF4
CTIVNIHEL	9	46.2	Constitutive coactivator of peroxisome proliferator-activated receptor gamma	Q6RI63; Q6RI63-2
RAIELATTL	9	46.3	N-alpha-acetyltransferase 15, NatA auxiliary subunit	Q80UM3
SSLSNPIANTM	11	46.4	F-box only protein 33	Q8VE08
NSLNNLIEL	9	46.4	E3 ubiquitin-protein ligase HERC2	Q4U2R1-2; Q4U2R1
SIVKNLNFI	9	46.5	vacuolar protein sorting-associated protein 13b	Q80TY5; Q80TY5
SAVENILEHL	10	46.7	28S ribosomal protein S6, mitochondrial	P58064
QSFTNPTLI	9	46.8	Peroxisomal membrane protein PEX13	Q9D0K1
SSFKNLVSL	9	46.8	Toll-like receptor 9	Q9EQU3
IMNLNGRDYI	10	47.2	Transcription initiation factor IIA subunit 1	Q99PM3
GTHVNVTCI	9	47.3	Tumor necrosis factor receptor superfamily member 1B	P25119
KAVENSSTAI	10	47.5	Proteasome subunit alpha type-3	O70435
SSVSNPRNWL	10	47.5	Tumor necrosis factor receptor superfamily member 23	Q9ER63
FSPTNPAHLL	10	47.7	WD repeat-containing protein 76	A6PWY4-1; A6PWY4-3; A6PWY4-2
TSFVNFTDI	9	47.8	Eukaryotic translation initiation factor 2 subunit 2	Q99L45
VALYNQGHI	9	47.9	Synaptic vesicle membrane protein VAT-1 homolog	Q62465
SLLTNHVTL	9	47.9	Homeodomain-interacting protein kinase 1	O88904-1; O88904-2
LSAKNKDYM	9	48.1	bifunctional UDP-N-acetylglucosamine 2-epimerase/N-acetylmannosamine kinase	Q91WG8
LALENYITAL	10	48.2	Amyloid beta A4 protein	P12023-2; P12023; P12023-3
AALVDLDSL	9	48.2	Epsin-1	Q80VP1-2; Q80VP1
SSPSNGAFGE	14	48.2	T-box transcription factor TBX15	O70306
HAPCNVPPYL	10	48.3	Poly(A) RNA polymerase GLD2	Q91YI6-1
SAVSNNYIQTL	11	48.3	Catenin delta-1	P30999-2; P30999-1; P30999-3
YGINNIREL	9	48.5	Phenylalanine--tRNA ligase alpha subunit	Q8C0C7
TTLRNGEVL	9	48.6	transmembrane protein 135	Q9CYV5
GAVTNVKVI	9	48.6	ELAV-like protein 3	Q60900; P70372; Q60900-2; Q60899
FMKQNLDEL	9	48.7	5'-AMP-activated protein kinase subunit gamma-2	Q91WG5-2; Q91WG5

YSGRNAREFL	10	48.8	Carnitine O-palmitoyltransferase 2, mitochondrial	P52825
YIHSNQNVI	9	48.8	DNA excision repair protein ERCC-6-like 2	Q9JIM3
ISWENRDAV	9	48.9	Ribosomal biogenesis protein LAS1L	A2BE28-2; A2BE28
IAMSNSMINTI	11	49.1	Prokineticin receptor 1	Q9JKL1
GSHTNIHLL	9	49.2	Activity-dependent neuroprotector homeobox protein	Q9Z103
RSPENVIETI	10	49.2	eIF-2-alpha kinase activator GCN1	E9PVA8
FMIGNGENL	9	49.6	Sterol-4-alpha-carboxylate 3-dehydrogenase, decarboxylating	Q9R1J0
YQIAMVHYI	9	49.8	Inosine-5'-monophosphate dehydrogenase 1	P50096
GQVINLDQL	9	49.8	Protein FAM91A1	Q3UVG3
HMLNNPELM	9	50	Ubiquilin-4	Q99NB8
SQPPYNPTYM	10	50.1	Protein shisa-5	Q9D7I0-1; Q9D7I0-5; Q9D7I0-3; Q9D7I0-2
GAPSNPDVF	9	50.1	Ribosyldihydronicotinamide dehydrogenase [quinone]	Q9JI75
KAPTNEFYA	9	50.2	syndecan-4	O35988
FSLQNQLRL	9	50.3	protein flightless-1 homolog	Q9JJ28
RAIPNNQVL	9	50.4	Endothelial differentiation-related factor 1	Q9JMG1
YGGYNIEEI	9	50.5	NAD(P) transhydrogenase, mitochondrial	Q61941
RGPSNSTSL	9	50.7	Chloride channel protein 2	Q9R0A1
TGVVNAQAL	9	50.8	D-3-phosphoglycerate dehydrogenase	Q61753
SALQNFASTF	10	51.1	Brefeldin A-inhibited guanine nucleotide-exchange protein 3	Q3UGY8
ASFRNTGEI	9	51.2	Transaldolase	Q93092
TALRNLPEYTL	11	51.2	integrator complex subunit 1	Q6P4S8
SPLTNQYYL	9	51.2	All-trans-retinol 13,14-reductase	Q64FW2
VGVENVAEL	9	51.7	Glycogen phosphorylase, liver form	Q9ET01
SCPVNIASI	9	51.9	Thioredoxin domain-containing protein 16 OS=Mus musculus OX=10090	Q7TN22
SSIANLKVSL	10	51.9	Cyclin-dependent kinase 2-interacting protein	Q9D0V8; Q9D0V8-3; Q9D0V8-2
FNLVNNALL	9	52.2	Apoptosis inhibitor 5	O35841
AQPVNVQQL	9	52.2	E1A-binding protein p400	Q8CHI8-4; Q8CHI8-3; Q8CHI8-2; Q8CHI8-5; Q8CHI8
KAPDNRETL	9	52.7	Dolichyl-diphosphooligosaccharide--protein glycosyltransferase subunit STT3B	Q3TDQ1
GAIRNGGLYV	10	52.7	Uncharacterized aarF domain-containing protein kinase 5	Q80V03-2; Q80V03
FAYTSRHEI	9	52.8	Dehydrodolichyl diphosphate synthase complex subunit DHDDS	Q99KU1; Q99KU1-2
SAIMNPASKVI/	13	52.8	Clathrin heavy chain 1	Q68FD5
FSNVNLKEV	9	52.9	DNA damage-binding protein 1	Q3U1J4
VQIHNTENI	9	52.9	Ankyrin repeat and SOCS box protein 6	Q91ZU1
VAAINPELL	9	52.9	Kinesin-like protein KIF2C	Q922S8
ASVQNEAKL	9	53.1	coronin-1C	Q9WUM4
MAGINTDHL	9	53.1	Piezo-type mechanosensitive ion channel component 1	E2JF22
NMHNNLDEL	9	53.2	Sodium bicarbonate cotransporter 3	Q8BTY2; Q8BTY2-2
SNLSNYHQYI	10	53.3	Phosphatidylinositol 5-phosphate 4-kinase type-2 gamma	Q91XU3
YLHENASYV	9	53.5	Protein phosphatase 1 regulatory subunit 21	Q3TDD9-1
SGGQNITIM	9	53.7	Plexin-C1	Q9QZC2
YAGSNFPEHI	10	53.8	Actin-related protein 2	P61161
STIQNADLIVVI	12	53.8	Phosphatidylcholine translocator ABCB4	P21440; P06795; P21447
YSIQGQSVM	9	53.8	Protein MON2 homolog	Q80TL7; Q80TL7-2
LSIENKSEL	9	54	Nostrin	Q6WKZ7
FSITDQQTL	9	54.3	Protein NLRC5	C3VPR6
RGPVNLQHL	9	54.5	Leucine-rich repeat and fibronectin type-III domain-containing protein 4	Q80XU8
SALEDLEVL	9	54.6	Mediator of RNA polymerase II transcription subunit 4	Q9CQA5; Q9CQA5-2
HAPRNLRLTYL	11	54.7	sialoadhesin	Q62230-3; Q62230

SLITNKVVM	9	54.8	Purine nucleoside phosphorylase	P23492
HSVQNGTVRM	10	55	Isoform 2 of Rho GTPase-activating protein 24	Q8C4V1-3; Q8C4V1-1; Q8C4V1-2
ASPMNHSVQM	10	55	CREB-binding protein	P45481
VAIENPADVSV	12	55	40S ribosomal protein SA	P14206
SQGMNVTNM	9	55.4	Histone acetyltransferase p300	B2RWS6
VQVNNVVVL	9	55.5	Histone chaperone ASF1A	Q9CQE6
FAFVNEEKF	9	55.6	Myotubularin	Q9Z2C5
AGLLNNPHFI	10	55.9	Small glutamine-rich tetratricopeptide repeat-containing protein alpha	Q8BJU0-1; Q8BJU0-2
RSVPNVEEL	9	56.3	Beta/gamma crystallin domain-containing protein 3	Q80W49; Q80W49-2
VTIHNQDLL	9	56.4	Zinc finger protein ZXDC	Q8C8V1-3; Q8C8V1-4; Q8C8V1-1; Q8C8V1-2
SAPSNFEHRV	10	56.5	serine/threonine-protein kinase PAK 4	Q8BTW9
GANVNAKDTL	10	56.5	Serine/threonine-protein phosphatase 6 regulatory ankyrin repeat subunit C	Q8BTI7
RAAYNVTLL	9	56.6	alpha-ketoglutarate-dependent dioxygenase FTO	Q8BGW1; Q8BGW1-4; Q8BGW1-3; Q8BGW1-2
SALQNAESDRI	11	57.1	Centromere-associated protein E	Q6RT24
ASIVNKDGL	9	57.2	Peroxisome proliferator-activated receptor delta	P35396
SGIKNGNFAL	10	57.2	Protocadherin Fat 3	Q8BNA6; Q8BNA6-2
FAPVNVTTEVK	11	57.3	Elongation factor 1-alpha 1	P10126
YVFFNGDHM	9	57.4	E3 ubiquitin-protein ligase RNF213	E9Q555
RALLLSTYI	9	57.5	AP-2 complex subunit alpha-2	P17426-2; P17427; P17426
AKLVNQEVL	9	57.6	NADH-ubiquinone oxidoreductase 75 kDa subunit, mitochondrial	Q91VD9
YQLVNSIFQHL	11	57.7	Interferon-induced very large GTPase 1	Q80SU7
RIIANVEEI	9	57.8	ATP-binding cassette sub-family D member 2	Q61285
KQAQNIVTL	9	57.8	Citramalyl-CoA lyase, mitochondrial	Q8R4N0
VSYKNPSLM	9	58	FACT complex subunit SPT16	Q920B9
LSHINVALI	9	58	RNA-binding protein 33	Q9CXK9-1
AVVNVTYM	8	58	Insulin-like growth factor 2 mRNA-binding protein 2	Q5SF07-2; Q5SF07
FSYSGWDTL	9	58.2	Y+L amino acid transporter 1	Q9Z1K8; Q8BGK6; Q8BGK6-2
QSITNLSNL	9	58.4	Protein strawberry notch homolog 1	Q689Z5; Q689Z5-2
HSLQDWDTI	9	58.5	camp-dependent protein kinase catalytic subunit prkx	Q922R0
AGIYNLDDL	9	58.7	General transcription and DNA repair factor IIH helicase subunit XPD	O08811
TATMNLDRI	9	58.7	Inositol 1,4,5-trisphosphate receptor type 3	P70227
QAPQNKITV	9	59.1	L-lactate dehydrogenase A chain	P06151
SNLVNENTL	9	59.3	ATP-dependent RNA helicase DDX18	Q8K363
AAHTNRKEYTL	11	59.3	Claudin domain-containing protein 1	Q9CQX5
QAINNAINYL	10	59.4	DNA-binding protein Ikaros	Q03267-2; Q03267-4; Q03267-3; Q03267-6; Q03267-5; Q03267
SQVYNDAHI	9	59.5	Protein polybromo-1	Q8BSQ9; Q8BSQ9-2
MAIKNPKATL	10	59.8	Hypoxia up-regulated protein 1	Q9JKR6
LSHTNILVL	9	60.1	Toll-like receptor 9	Q9EQU3
YTIENPRHFV	10	60.1	staphylococcal nuclease domain-containing protein 1	Q78PY7
LSLENGTHTL	10	60.4	DNA (cytosine-5)-methyltransferase 1	P13864
YSIRNPPQPLI	11	60.4	focadhesin	A2AKG8-2; A2AKG8
SAVQNVADVA'	12	60.4	Cytoplasmic dynein 1 heavy chain 1	Q9JHU4
FSKVNIQVL	9	61.1	2'-5'-oligoadenylate synthase-like protein 2	Q9Z2F2
AALKNAFSLL	10	61.2	DmX-like protein 2	Q8BPN8; Q8BPN8-2; Q6PNC0
ASARNLEHL	9	61.3	F-box only protein 38	Q8BMI0
SAVQNLVVEGI	11	61.4	AMSH-like protease	Q76N33-1; Q76N33-2
SSPENLKGFI	10	61.7	Testis-expressed protein 10	Q3URQ0

KAIENLGVSYV	11		62	DNA primase large subunit	P33610
RSLLNKQETL	10		62.1	Isoform 2 of IQ calmodulin-binding motif-containing protein 1	Q8BP00-2; Q8BP00-1
KALEDVDYV	9		62.2	Serine/threonine-protein phosphatase 4 regulatory subunit 3A	Q6P2K6-2; Q6P2K6-1
DSLVNQEVL	9		62.3	DNA-directed RNA polymerase I subunit RPA2	P70700
WSPLNHSLL	9		62.5	Methylosome protein 50	Q99J09
IQAENAEFM	9		62.6	heme oxygenase 1	P14901
AAVVSVAYL	9		62.7	Natural resistance-associated macrophage protein 2	P49282-4; P49282-3; P49282-2; P49282
AAVQNPALTAL	11		63.1	Sterol regulatory element-binding protein 2	Q3U1N2-1; Q3U1N2-2
YLMGNLEEI	9		63.8	Rho guanine nucleotide exchange factor 7	Q9ES28; Q9ES28-7; Q9ES28-5; Q9ES28-3; Q9ES28-4; Q9ES28-6; Q9ES28-8; Q9ES28-2
TQPVNLFNL	9		63.9	Pericentriolar material 1 protein	Q9R0L6; Q9R0L6-2
KSLINKNEL	9		64.1	Inositol polyphosphate 5-phosphatase OCRL-1	Q6NVF0-2; Q6NVF0
FAVKNEKRFYL	11		64.1	Lysosome-associated membrane glycoprotein 2	P17047-2; P17047; P17047-3
FLVQNIHTL	9		64.2	Ribosome biogenesis protein BRX1 homolog	Q9DCA5
SQLENFCRYL	10		64.7	PAX3- and PAX7-binding protein 1	P58501
SGIKNPVSV	9		64.8	Threonine aspartase 1	Q8R1G1; Q8R1G1-2
SSLKNFQSCI	10		65	E3 SUMO-protein ligase NSE2	Q91VT1-1
TTVANVETV	9		65.3	NADH dehydrogenase [ubiquinone] flavoprotein 1, mitochondrial	Q91YT0
SNPRNWLFL	9		65.6	Tumor necrosis factor receptor superfamily member 23	Q9ER63
YQYTSPDFL	9		65.8	Insulin-induced gene 1 protein	Q8BGI3; Q91WG1
SVVLNHYEL	9		65.9	Protocadherin Fat 3	Q8BNA6; Q8BNA6-3
VSLLNPPETL	10		66	Cyclin-A2	P51943
YQHKNLLIL	9		66	Transportin-1	Q8BFY9; Q8BFY9-2; Q99LG2
ANIRNICVL	9	Carbamidomethyl [C	66.4	Elongation factor-like GTPase 1	Q8C0D5
YQFRNLAECL	10		66.8	Peptidyl-tRNA hydrolase ICT1, mitochondrial OS=Mus musculus OX=10090	Q8R035
PSHLNVDYI	9		67.3	zinc transporter 4	O35149
SSIVNKEGL	9		67.4	Tropomodulin-1	P49813
GGPENVAEM	9		67.4	Protein strawberry notch homolog 1	Q689Z5; Q689Z5-2
SRILNQNYI	9		68.1	Galactocerebrosidase	P54818
SSMRGMDTV	9		68.1	Arginine/serine-rich coiled-coil protein 2	A2RTL5
SLVTNMDKL	9		68.3	Nck-associated protein 1-like	Q8K1X4
ATLENVTNL	9		68.5	UPF0587 protein C1orf123 homolog	Q8BHG2-2; Q8BHG2; Q8BHG2-3
KAVVMLEYV	9		68.6	Cullin-2	Q9D4H8; Q9D4H8-2
SAVRNGLLLL	10		69	Cullin-9	Q80TT8-1; Q80TT8-4
FQVKNPPHTYI	11		69	Formin-like protein 3	A2APV2-3; Q6ZPF4-2; Q6ZPF4-1; A2APV2-1; A2APV2-2
KQIKNSSLL	9		69.1	uncharacterized protein KIAA1143 homolog	Q8K039
SALRNCFSHL	10	Carbamidomethyl [C	69.3	mannose-6-phosphate isomerase	Q924M7
ISPENHISL	9		69.4	Interferon-inducible double-stranded RNA-dependent protein kinase activator A	Q9WTX2
ATISNDGATI	10		69.4	T-complex protein 1 subunit eta	P80313
GSPANTRYL	9		69.5	Isoform 2 of Serine/threonine-protein phosphatase 2B catalytic subunit alpha isoform	P48453-2; P63328; P63328-2; P48453-1
SAVVDFEAL	9		69.5	Telomere-associated protein RIF1	Q6PR54-2; Q6PR54-3; Q6PR54
FMALNKEHL	9		69.7	caspase recruitment domain-containing protein 9	A2AIV8
FCYVNDIVI	9		69.7	Histone deacetylase 3	O88895-1; O09106; P70288
QSMENGLNYM	10		69.7	CCR4-NOT transcription complex subunit 1	Q6ZQ08-2; Q6ZQ08-4; Q6ZQ08-1
HALLNDAWVL	10		69.9	Lethal(2) giant larvae protein homolog 2	Q3TJ91
SIISNTEAV	9		70	Heat shock protein 75 kDa, mitochondrial	Q9CQN1
TAPPNFNHM	9		70.1	Serine/threonine-protein kinase WNK1	P83741-2; P83741; P83741-3; P83741-5; P83741-4

SGIVNMDTP	9		70.1	Ankyrin repeat domain-containing protein 17	Q99NH0
NQLQNYRNYL	10		70.2	tumor suppressor p53-binding protein 1	P70399-2; P70399-5; P70399-3; P70399; P70399
RNISNQLVI	9		70.2	Checkpoint protein HUS1	Q8BQY8-3; Q8BQY8; Q8BQY8-2
AMGVNMETA	9		70.9	V-type proton ATPase subunit B, brain isoform	P62814
SVIRNPEIL	9		70.9	eIF-2-alpha kinase activator GCN1	E9PVA8
YQGQNAWFL	9		70.9	4F2 cell-surface antigen heavy chain	P10852; P10852-2
SVATNIDEI	9		71.2	Structural maintenance of chromosomes protein 1A	Q9CU62
FSLVGFTQM	9		71.2	Very-long-chain enoyl-CoA reductase	Q9CY27
VGLNNINAL	9		71.3	CUGBP Elav-like family member 2	Q9Z0H4; Q9Z0H4-7; Q9Z0H4-11; Q9Z0H4-9; Q9Z0H4-4; Q9Z0H4-8; Q9Z0H4-6; Q9Z0H4-2; Q9Z0H4-3; Q9Z0H4-5
STLLNVHIV	9		71.5	cysteine and histidine-rich domain-containing protein 1	Q9D1P4
ISLANLDTNKL	11		71.9	Putative ataxin-7-like protein 3B	Q3UD01
SAYRNKFGEI	10		72.2	Carbohydrate sulfotransferase 14	Q80V53
HSIQNSQDM	9		72.3	BAG family molecular chaperone regulator 2	Q91YN9
AGQFNQDYL	9		72.4	annexin A11	P97384
WAQLNRDLI	9		73	Derlin-1	Q99J56
ASVLNKWQM	9		73.1	ATP-dependent RNA helicase DDX1	Q91VR5
NMHQNIKYI	9		73.2	Glycoprotein endo-alpha-1,2-mannosidase	Q6NXH2
RTVENIFYV	9		73.3	EP300-interacting inhibitor of differentiation 3	Q3V124
HGVLNTDNM	9		73.5	Selenoprotein O	Q9DBC0
GKLLNAEYI	9		74.1	Actin-binding LIM protein 2	Q8BL65-4; Q8BL65-5; Q8BL65-3; Q8BL65-2; Q8BL65
SQWNNDNPL	9		74.2	Integrin beta-2	P11835
SQIGNAMGYI	10		74.2	WASH complex subunit 4	Q3UMB9
RSPENKFPVI	10		74.3	Nuclear factor interleukin-3-regulated protein	O08750
FAFSNGYLASL	11		74.5	Equilibrative nucleoside transporter 1	Q9JIM1; Q9JIM1-2
QSLSNEALM	9		74.8	Diablo homolog, mitochondrial	Q9JIQ3
NGIRNIDLHYI	11		74.9	E3 ubiquitin-protein ligase MARCH6	Q6ZQ89; Q6ZQ89-3
SSFVDHDFL	9		75.4	Histone-lysine N-methyltransferase ASH1L	Q99MY8
RALSNSVSNM	10		75.5	Forkhead box protein O3	Q9WVH4
GQLENAEQL	9		75.8	Tetratricopeptide repeat protein 19, mitochondrial	Q8CC21
KAVVNEKVL	9		76.4	Protein MON2 homolog	Q80TL7-1; Q80TL7-2
GAPLNIHKV	9		76.5	TBC1 domain family member 5	Q80XQ2
SSTTNIDDL	9		76.5	Cytoskeleton-associated protein 5	A2AGT5; A2AGT5-3; A2AGT5-2
HILANADAI	9		76.6	Tumor necrosis factor alpha-induced protein 2	Q61333
IMGQNVADYM	10	1xOxidation [M10]	76.9	60S ribosomal protein L5	P47962
NTPENREYL	9		77	Actin-related protein 3B	Q641P0; Q641P0-2
STLMNHERI	9		77.1	Zinc finger protein 250	Q7TNU6
TSPINPQHM	9		77.5	Trinucleotide repeat-containing gene 6C protein	Q3UHC0
RSITNDSAVL	10		77.7	Hepatocyte growth factor-regulated tyrosine kinase substrate	Q99LI8
GALENAKAEI	10		78.4	Centrosomal protein POC5	Q9DBS8
TMKTNLEYL	9		78.5	Structural maintenance of chromosomes protein 6	Q924W5; Q924W5-2
RQATNQIVM	9	1xOxidation [M9]	78.9	puromycin-sensitive aminopeptidase	Q11011
VGPKNKTSI	9		79.2	Ubiquitin carboxyl-terminal hydrolase 10	P52479; P52479-2
VSILNYQSAL	10		79.4	Homeodomain-interacting protein kinase 2	Q9QZR5
TAISNYMNQL	10		79.5	DCC-interacting protein 13-alpha	Q8K3H0
VGLINKDSV	9		79.7	Histone-lysine N-methyltransferase ASH1L	Q99MY8

FAHFNSHAA	9		80.3	Arf-GAP domain and FG repeat-containing protein 1	Q8K2K6-4; Q8K2K6-3; Q8K2K6-1; Q8K2K6-2
SSLQNQALQTI	11		80.5	Golgin subfamily A member 5	Q9QYE6
GGNINVETI	9		80.5	Dolichyl-diphosphooligosaccharide--protein glycosyltransferase 48 kDa subunit	O54734
IAVANAQELL	10		80.6	mitochondrial import inner membrane translocase subunit TIM13	P62075
SSVLNQEEAY\	11		80.7	Interleukin-7 receptor subunit alpha	P16872
TSTTNFTKI	9		80.9	Transmembrane protein 131-like	Q3U3D7-2; Q3U3D7-1; Q3U3D7-3
SHLTNKYVL	9		80.9	Isoform 4 of Transmembrane protein 241	Q3UME2-3; Q3UME2-2; Q3UME2-4; Q3UME2
TSPVNPAVF	9		81	transmembrane protein 258	P61166
SSLLDLETL	9		81.1	Proline and serine-rich protein 3	Q7TSA6-2; Q7TSA6
KGVFNVEVV	9		81.2	Dedicator of cytokinesis protein 7	Q8R1A4-2; Q8R1A4
YSPESLHYM	9		82.1	Thyroid adenoma-associated protein homolog	A8C756-1
LAVSNHVFHL	10		82.3	Zinc finger MIZ domain-containing protein 1	Q6P1E1-2; Q6P1E1
SAIANIPAAAV	11		82.8	Eyes absent homolog 3	P97480; P97480-2
RLITNSEEI	9		83	ATP-binding cassette sub-family D member 3	P55096
FALSNEHYS	9		83	Leucine zipper protein 1	Q8R4U7
TGLENSTAV	9		83.2	Secretion-regulating guanine nucleotide exchange factor	Q80YD6
FMATNSLHL	9		83.3	Cyclin-T2	Q7TQK0; Q7TQK0-2; Q9QWV9
HTHMNVHYV	9		83.4	male-specific lethal 3 homolog	Q9WVG9-2; Q9WVG9-1
SGLSNYRLIFM	11		83.7	Solute carrier organic anion transporter family member 4A1	Q8K078-2; Q8K078
NSIKNGILYL	10		83.9	1-phosphatidylinositol 4,5-bisphosphate phosphodiesterase gamma-1	Q62077
FAIDDHDYL	9		84.2	Phosphatidylinositol 5-phosphate 4-kinase type-2 gamma	Q91XU3
LSNNNHTEI	9		84.2	Serine/threonine-protein kinase SMG1	Q8BKX6
MSLVNKTTHLI	11		84.3	26s proteasome non-atpase regulatory subunit 12	Q9D8W5
STVFNPSVL	9		84.4	muscleblind-like protein 2	Q8C181; Q8C181-2; Q8C181-4
TGYLNTVTV	9		84.6	Receptor of activated protein C kinase 1	P68040
YCLPNLTHL	9		85	Peroxisome proliferator-activated receptor gamma coactivator-related protein 1	Q6NZN1
TSFKNPFLI	9		85.1	Tripeptidyl-peptidase 1	O89023
TGPSNVDKL	9		85.2	Serine/threonine-protein kinase Chk1	O35280-2; O35280
RALENPDTASL	11		85.2	Tyrosine-protein phosphatase non-receptor type 23	Q6PB44; Q6PB44-2
TALEHQEYI	9		85.3	Complement component 1 Q subcomponent-binding protein, mitochondrial	O35658
FQLENFTLKV	10		85.4	Insulin-like growth factor 2 mRNA-binding protein 3	Q9CPN8
WAIENPAAF	9		85.6	protein-lysine N-methyltransferase EEF2KMT	Q3UZW7-2; Q3UZW7-3; Q3UZW7
QSLTNILHL	9		87.2	lysosomal Pro-X carboxypeptidase	Q7TMR0-1
FGNINSCHL	9	Carbamidomethyl [C	87.4	Solute carrier family 15 member 4	Q91W98
LSVWNQQVL	9		87.4	Mediator of RNA polymerase II transcription subunit 14	A2ABV5-3; A2ABV5-2; A2ABV5-4; A2ABV5
GMKLNLDYL	9		87.8	Developmentally-regulated GTP-binding protein 2	Q9QXB9
SAYSNYAEQTL	11		87.8	RNA-binding protein 4	Q8C7Q4-1
RQTVNVIAM	9		88	Tumor susceptibility gene 101 protein	Q61187
MSLENGTRV	9		88.1	peptidyl-prolyl cis-trans isomerase FKBP8	O35465-1; O35465-2
SSPVNPVVFF	10		88.6	Peptidyl-prolyl cis-trans isomerase H	Q9D868; Q9D868-2
YQIVNCQHF	9		88.7	DNA repair protein RAD51 homolog 2 OS=Mus musculus OX=10090	O35719
TAAENAEKI	9		88.7	Leucine-rich repeat flightless-interacting protein 2	Q91WK0-2; Q91WK0-1
VTVLNITHL	9		89.2	Activating signal cointegrator 1 complex subunit 3	E9PZJ8-2; E9PZJ8
HAISNYYDNHL	11		89.4	Exonuclease 3'-5' domain-containing protein 2	Q8VEG4-2; Q8VEG4
SQIKNVHSF	9		89.5	Integral membrane protein GPR137B	Q8BNQ3
QGIKNSVTL	9		89.5	Centrosomal protein of 78 kDa	Q6IRU7
QALEDLDYL	9		89.6	Putative pre-mRNA-splicing factor ATP-dependent RNA helicase DHX32	Q8BZS9-2; Q8BZS9-1
AVNMNLHYV	9		89.7	DNA-directed RNA polymerase I subunit RPA2	P70700

SSIQNFQKGTL	11		90	PX domain-containing protein kinase-like protein	Q8BX57-1
MNVQNVEFI	9		90.1	serine/threonine-protein kinase ATR	Q9JKK8
YSLANNPQYKI	11		90.2	Calpain-7	Q9R1S8
FQYLNRREL	9		90.7	F-box/WD repeat-containing protein 8	Q8BIA4-4; Q8BIA4
KNLVNKEVM	9		91.4	glucose-6-phosphate isomerase	P06745
TGMRDADYI	9		91.5	histone deacetylase 6	Q9Z2V5
GAVYNGEPKL	10		91.7	sphingosine-1-phosphate lyase 1	Q8R0X7
SGNFNVDNI	9		91.9	T-complex protein 1 subunit theta	P42932
SIGQNMVTI	9		91.9	Transferrin receptor protein 1	Q62351
SCIPNREYV	9		92.1	NAD-dependent protein deacetylase sirtuin-7	Q8BKJ9
SQITSLVTL	9		92.1	Origin recognition complex subunit 5	Q9WUV0
FAPVNVTTE	9		92.2	Elongation factor 1-alpha 1	P10126
LSPRNLVLV	9		92.3	Protein RRNAD1	Q8BZG5
KALKLLDYV	9		92.7	trafficking protein particle complex subunit 11	B2RXC1
KQPLNTTRI	9		93.1	tyrosine-protein phosphatase non-receptor type 11	P35235; P35235-2
KTHINIVVI	9		93.2	Elongation factor 1-alpha 1	P10126; P62631
AAHQNPEICL	10		93.5	Twinkle protein, mitochondrial	Q8CIW5
TAVVNRVFDKI	11		93.5	Proteasome subunit beta type-9	O35522; P28076
FAPVNVTTEVK	12		94	Elongation factor 1-alpha 1	P10126
SSTSNRESV	9		94.2	SAM and SH3 domain-containing protein 1	P59808
VACVNQFII	9		94.4	transportin-1	Q8BFY9
LAIVNTPEKL	10		94.6	C-type lectin domain family 5 member A	Q9R007-3; Q9R007-2; Q9R007-1
RALELGTVM	9		94.9	1-phosphatidylinositol 4,5-bisphosphate phosphodiesterase gamma-2	Q8CIH5
ISPFNKSAL	9		95.1	CLIP-associating protein 2	Q8BRT1
YAVRDTIAL	9		95.4	Phospholipid-transporting ATPase 11C	Q9QZW0-1; Q9QZW0-2
YGYSNRVVDLI	11		95.4	glyceraldehyde-3-phosphate dehydrogenase	P16858
VVVENITLM	9		95.4	E3 ubiquitin-protein ligase UBR4	A2AN08-2; A2AN08-3; A2AN08-5; A2AN08
FAPYNKPSL	9		95.5	Probable E3 ubiquitin-protein ligase DTX2	Q8R3P2-2; Q8R3P2; Q8R3P2-3
YALIHPATTL	10		96.1	Protein Smaug homolog 2	Q80XS6
LALVLHDTL	9		96.2	AP-5 complex subunit beta-1	Q3TAP4
LGVTNFVHM	9		96.3	FAD-dependent oxidoreductase domain-containing protein 2	Q3USW5
SSSTNVDLL	9		96.4	Protein SAAL1	Q9D2C2-2; Q9D2C2
GTLSNPCTL	9		96.5	F-box only protein 11	Q7TPD1; Q7TPD1-2; Q7TPD1-3
VGPENMQIM	9		96.7	Protein MON2 homolog	Q80TL7; Q80TL7-2
YQSMNSQYL	9	1xOxidation [M4]	96.9	lysosomal protective protein	P16675
SALQNVKRL	9		97.1	Vacuolar protein sorting-associated protein 45	P97390
LSHINRDKL	9		97.3	Histone-lysine N-methyltransferase ASH1L	Q99MY8
SGIIDSDFL	9		97.3	General transcription factor 3C polypeptide 6	Q9D8P7
SSPVNVKKL	9		97.7	Heterogeneous nuclear ribonucleoprotein U	Q8VEK3-2; Q8VEK3
SALDNGPQI	9		97.7	E3 ubiquitin-protein ligase Itchy	Q8C863; Q8C863-2
NQVKNIAEL	9		98	DNA repair endonuclease XPF	Q9QZD4
STHVNHNIYI	10		98.2	Activating molecule in BECN1-regulated autophagy protein 1	A2AH22-2; A2AH22; A2AH22-6; A2AH22-5; A2AH22-3; A2AH22-7; A2AH22-4
DQIQNAQYL	9		98.4	Heterogeneous nuclear ribonucleoprotein K	P61979-3; P61979-2; P61979
SQVVNVLTSVI	11		98.4	Uncharacterized protein C1orf112 homolog	Q3TQQ9-1; Q3TQQ9-2; Q3TQQ9-3
GGVRNGVAL	9		98.6	nucleolar protein 11	Q8BJW5-2; Q8BJW5-1
RQFLNRDDI	9		98.7	V-type proton ATPase subunit F	Q9D1K2
ISHINKELL	9		99	Maleylacetoacetate isomerase	Q9WVL0

ATLKNTEKI	9		99.5	Probable phospholipid-transporting ATPase IH	P98197
IAMSNSMINTL	11		99.5	Prokineticin receptor 1	Q9JKL1; Q8K458
KAVENPTATEI	11		99.6	Signal recognition particle 19 kDa protein	Q9D7A6
RQVQNTAITL	10		99.8	MORC family CW-type zinc finger protein 2B	Q69ZX6; Q8C5W4-2; Q8C5W4-1
VSPRNSLEVL	10		99.8	DDB1- and CUL4-associated factor 10	A2AKB9-3; A2AKB9-2; A2AKB9-1
KGITNHLVAI	10		99.9	Macrophage-expressed gene 1 protein	A1L314
SSIQNGKYTLN	12		100.2	Lysine-specific demethylase 7A	Q3UWM4
LSVSNIIQL	9		100.4	ATP-dependent 6-phosphofructokinase, liver type	P12382
KAVENYINCL	10		100.8	Serine-protein kinase ATM	Q62388
SQARNLNEV	9		101.1	condensin-2 complex subunit D3	Q6ZQK0
YNVINREKL	9		101.5	protein farnesyltransferase subunit beta	Q8K2I1
ASLHNQITA	9		102	Vacuolar protein sorting-associated protein 52 homolog	Q8C754; Q8C754-2
SGIRNQGGTC	12		102	Ubiquitin carboxyl-terminal hydrolase 40	Q8BWR4-1
KMFVNSNHL	9		102.1	nucleolar pre-ribosomal-associated protein 1	Q571H0
FGLENALRL	9		102.4	Coiled-coil domain-containing protein 77	Q9CZH8; Q9CZH8-2
IQLVNAKHI	9		102.4	3-hydroxy-3-methylglutaryl-coenzyme A reductase	Q01237
AAIQGAVAM	9	1xOxidation [M9]	102.7	(E3-independent) E2 ubiquitin-conjugating enzyme UBE2O	Q6ZPJ3
KSTVNKWTL	9		103.3	WD repeat-containing protein 48	Q8BH57; Q8BH57-2; Q8BH57-3
SASSNPFAL	9		103.4	GATOR complex protein WDR59	Q8C0M0; Q8C0M0-2; Q8C0M0-3
SRLINLTFL	9		103.5	CD180 antigen	Q62192
YQQMNPEAL	9	1xOxidation [M4]	104.4	Transcription factor MafB	P54841
RMIQNQNAA	9		104.5	5'-3' exoribonuclease 2	Q9DBR1; Q9DBR1-2
FSHPNVLPVL	10		104.5	Integrin-linked protein kinase	O55222
SLIRNLEQL	9		105	Syntaxin-binding protein 2	Q64324
VQINNPVTF	9		105	Integrin beta-2	P11835
AGPSNFGTEL	10		105	Roquin-2	P0C090
TAVTNSARL	9		105.2	Vacuolar protein sorting-associated protein 33B	P59016
YTLLNKAPEYL	11		105.2	Ras GTPase-activating protein-binding protein 2	P97379-1; P97379-2
MSIHNMDHA	9		105.4	Phosphatidylinositol 4-phosphate 5-kinase type-1 alpha	P70182-3; P70182
YTVENAKDII	10		105.5	Tryptophan--tRNA ligase, cytoplasmic	P32921-2; P32921
VQPRNVSVTM	10		106.2	Plexin-A1	P70206
AIMENANVL	9		106.3	Fructose-bisphosphate aldolase A	P05064
KGPSNVFKI	9		106.4	Superkiller viralicidic activity 2-like 2	Q9CZU3
STPTNHNLFPI	11		106.5	Dual specificity protein phosphatase 7	Q91Z46; Q91Z46-2
SIRNLDTI	8		106.8	Ataxin-10	P28658
ASPLNPDMKM	10		106.9	Putative Polycomb group protein ASXL2	Q8BZ32
IQVPNCDEI	9		106.9	Coatomer subunit alpha	Q8CIE6
VQQFNLDVI	9		107	Exocyst complex component 6	Q8R313
AAMVSKFFL	9		107	Inactive phospholipid phosphatase 7	Q91WB2
SHAMNITPL	9		107.1	Granulocyte-macrophage colony-stimulating factor receptor subunit alpha	Q00941
SALVKEEYL	9		107.2	dystonin	Q91ZU6-3; Q91ZU6-4; Q91ZU6-2; Q91ZU6
AAMKLMTAL	9		107.4	Cohesin subunit SA-2	O35638; Q9D3E6
LMGVNLHEL	9	1xOxidation [M2]	107.9	Transcription initiation factor TFIID subunit 3	Q5HZG4
LAPQNKPEL	9		108.1	Protein FAM107B	Q3TGF2
SMLQNPLGNV	11		108.3	Cytoplasmic polyadenylation element-binding protein 1	P70166
RSPGNSPTPM	10		108.4	BAG family molecular chaperone regulator 4	Q8CI61
SSEINSAYL	9		109.2	Zinc finger CCCH-type antiviral protein 1	Q3UPF5; Q3UPF5-2
ISVENSIDI	9		109.7	Interleukin-13 receptor subunit alpha-2	O88786; O88786-2

VAVNNKYRL	9	110.2	RAB6A-GEF complex partner protein 1	Q69ZJ7; Q69ZJ7-2
GGIQNVGHI	9	110.3	inosine-5'-monophosphate dehydrogenase 2	P24547
NALHNIKTL	9	110.4	CUGBP Elav-like family member 2	Q9Z0H4-1; Q9Z0H4-7; Q9Z0H4-11; Q9Z0H4-9; Q9Z0H4-4; Q9Z0H4-8; Q9Z0H4-6; Q9Z0H4-2; Q9Z0H4-5
WGFVNKDQI	9	110.8	CD81 antigen	P35762
SCLTNAVHL	9	111.5	enhancer of polycomb homolog 2	Q8C0I4
SSLQNHNHQL	10	111.5	E3 ubiquitin-protein ligase BRE1B	Q5DTM8; Q5DTM8-2; Q3U319
LMKVNLEYL	9	111.8	UPF0668 protein C10orf76 homolog	Q6PD19-3; Q6PD19-1
FQVKNPPAAYI	11	111.9	formin-like protein 1	Q9JL26-2; Q9JL26
FGLSNEDLEEL	11	111.9	Zinc finger protein 638	Q61464-4; Q61464; Q61464-2; Q61464-3; Q61464-7; Q61464-5
GALKNLSVIYI	11	112.1	E3 ISG15--protein ligase Herc6	F2Z461
SVIQNLRTV	9	113	26S proteasome non-ATPase regulatory subunit 1	Q3TXS7
ESPINVTTV	9	113.2	Wings apart-like protein homolog	Q65Z40
GNVTNITTV	9	113.4	Transforming growth factor beta activator LRRC33	Q8BMT4; Q8BMT4-2; Q8BMT4-3
SSFVNEATF	9	114.5	WD40 repeat-containing protein SMU1	Q3UKJ7
AACLNFNCM	9	114.7	Cytochrome b-245 heavy chain	Q61093
SAIINPPQACIL	12	114.7	oyllysine-residue acetyltransferase component of pyruvate dehydrogenase complex, mitc	Q8BMF4
SHLLNTSTL	9	114.7	Zinc finger protein 106	O88466-3; O88466; O88466-2
RQVENVLSL	9	115	Protein FAM173A	Q501J2
KTVVNISSL	9	115.1	Sepiapterin reductase	Q64105
QQVLNIECL	9	115.4	AP-2 complex subunit alpha-1	P17426
RAVIGSTYM	9	115.6	major vault protein	Q9EQK5
RALELLNYL	9	115.8	Pre-mRNA-splicing factor ATP-dependent RNA helicase DHX15	O35286
TGISSIDFI	9	115.9	CD180 antigen	Q62192
SQVTLREPL	9	116.1	cytoplasmic FMR1-interacting protein 1	Q7TMB8-2; Q7TMB8-1
AMYVHAYTL	9	116.6	Proteasome subunit alpha type-3	O70435
SMGSNDIDTL	10	116.7	E3 ubiquitin-protein ligase RNF13	O54965-2; O54965-1
FCLQNAQHDP	11	117	ne nucleotide-binding protein G(I)/G(S)/G(O) subunit gamma-5 OS=Mus musculus OX=1	Q80SZ7
TSLKNAGTCL	10	117.7	D-3-phosphoglycerate dehydrogenase	Q61753
HSILMIDEI	9	117.9	Solute carrier family 12 member 2	P55012
RQITNFQLSI	10	118.1	Proto-oncogene vav	P27870
FATNNSEHITY	12	118.1	Cell division cycle 5-like protein	Q6A068
IINENYDYL	9	118.4	nuclear pore complex protein Nup98-Nup96	Q6PFD9
SVLRNPFETI	10	118.6	Claspin	Q80YR7
RQAENGMYI	9	119	CAD protein	B2RQC6-2; B2RQC6
FSILNTPKKL	10	119.5	nuclear mitotic apparatus protein 1	E9Q7G0
SAPDNVLEV	9	119.9	E3 ubiquitin-protein ligase HECTD1	Q69ZR2
HQVKNHMWI	9	120.1	DNA topoisomerase 2-alpha	Q01320
RSVDNIQFL	9	120.5	Sugar transporter SWEET1	Q9CXK4
QQVINIDQL	9	120.7	Glutamyl-tRNA(Gln) amidotransferase subunit B, mitochondrial	Q99JT1
SSLLNIQHF	9	121.1	UHRF1-binding protein 1-like	A2RSJ4
LVIFNQEVI	9	121.4	Paired amphipathic helix protein Sin3a	Q60520-2; Q60520-1
SAVTNSGVHLI	11	121.4	GTP-binding protein 8	Q9CY28-2; Q9CY28-1
WKVVNPYYL	9	121.8	5'-AMP-activated protein kinase catalytic subunit alpha-1	Q5EG47
KQIYNLIHL	9	121.9	Protein fem-1 homolog B	Q9Z2G0
FQALNAEKL	9	122.1	serine/threonine-protein kinase ATR	Q9JKK8

NGILNVSAV	9	122.3	Heat shock cognate 71 kDa protein	P63017
KGVYNKSTI	9	123.2	structural maintenance of chromosomes flexible hinge domain-containing protein 1	Q6P5D8
SGGVNLQSM	9	124.7	proliferating cell nuclear antigen	P17918
FSPLNPVRV	9	124.7	Heterogeneous nuclear ribonucleoprotein F	O35737; Q9Z2X1-2; Q9Z2X1-1
SIMYNVTEL	9	124.8	Trafficking protein particle complex subunit 13	Q3TIR1-2; Q3TIR1; Q3TIR1-3
TAAFNKDAL	9	125.1	Phosphatidylinositol 4,5-bisphosphate 3-kinase catalytic subunit beta isoform	Q8BTI9; O35904-1
RSVLNNQLL	9	125.4	CAP-Gly domain-containing linker protein 1	Q922J3-2; Q922J3-1
NSLLNNMEL	9	125.7	Calcium-binding mitochondrial carrier protein Aralar2	Q8BH59; Q9QXX4
FTLANVKEL	9	126.4	SHC SH2 domain-binding protein 1	Q9Z179
RTIRNAMKI	9	126.6	Eukaryotic translation initiation factor 3 subunit C	Q8R1B4
SSLINHQRV	9	126.7	Zinc finger protein 287	Q9EQB9
DAVANLTQL	9	127	AP-1 complex subunit gamma-like 2	O88512
VSLLNYQSAL	10	127.3	Homeodomain-interacting protein kinase 1	O88904-1; Q9QZR5; O88904-3; O88904-2
AAVSNKFRDLL	11	127.3	Conserved oligomeric Golgi complex subunit 4	Q8R1U1
KALINSVIKI	10	127.5	Lysosomal-trafficking regulator	P97412-2; P97412-1
CSSVNIHYL	9	127.6	DNA annealing helicase and endonuclease ZRANB3	Q6NZP1
SLLVNGVQL	9	128.4	Arginine--tRNA ligase, cytoplasmic	Q9D0I9
SAIRSGLEL	9	128.7	Sister chromatid cohesion protein PDS5 homolog A	Q6A026
NSLSNKENYV	10	129.4	Kinesin-like protein KIF23	E9Q5G3
AAIVDGDRI	9	129.5	Serine/threonine-protein kinase MRCK beta	Q7TT50
TVHENLNFM	9	129.6	Vacuolar protein sorting-associated protein 11 homolog	Q91W86
AGLCNRAVF	9	130.6	Sodium/potassium-transporting ATPase subunit alpha-2	Q8VDN2; Q6PIC6; Q6PIE5
GQAQNKVYL	9	130.8	Rho guanine nucleotide exchange factor 10-like protein	A2AWP8-1; A2AWP8-5; A2AWP8-4; A2AWP8-2; A2AWP8-3
RSHKNVADDY	11	131.6	Sorting nexin-5	Q9D8U8
RSLDNGGYYI	10	131.8	Tyrosine-protein kinase Lyn	P25911-1; P16277; P25911-2
NSTENSFTL	9	131.8	Ribonucleoside-diphosphate reductase subunit M2	P11157
INITNGEEV	9	132	Casein kinase I isoform alpha	Q8BK63-2; Q8BK63
AQLRNLDNAVI	11	132.3	ATP-dependent RNA helicase DHX33	Q80VY9
SQLQSLTLI	9	132.3	F-box/LRR-repeat protein 6	Q9QXW0
TAVRNGFHSL	10	132.6	e3 ubiquitin-protein ligase UBR4	A2AN08
SSPINISGQVI	11	132.6	Isoform 4 of Kinesin-like protein KIF20B	Q80WE4; Q80WE4-4; Q80WE4-2; Q80WE4-3
FQVDNFEAL	9	133.1	Integrin alpha-M	P05555; P05555-2
CQHENLVEL	9	133.2	Interleukin-1 receptor-associated kinase 4	Q8R4K2
YQKENKDVI	9	134	CDK-activating kinase assembly factor MAT1	P51949
VQVPNLESL	9	134	Chromodomain-helicase-dna-binding protein 8	Q09XV5
YSVSNSVKEIM	11	134.8	Cathepsin B	P10605
KAVENYLIQM	10	136	Programmed cell death protein 5	P56812
SQLQGYTVL	9	136.5	Selenocysteine lyase	Q9JLI6
FVYENPVSL	9	136.9	Rhomboid domain-containing protein 2	Q8VEK2
CAPNNGYFMI	10	138	Nodal modulator 1	Q6GQT9
SSPSSRETL	9	138.7	Carnosine synthase 1	Q6ZPS2-2; Q6ZPS2-3; Q6ZPS2
FSIKNKKGSEL	11	138.8	Moesin	P26041
KALGNEIVV	9	139.4	SLAM family member 5	Q18PI6; Q18PI6-3
ASMGNYITRM	10	139.8	Protein unc-93 homolog B1	Q8VCW4
RACANLQVL	9	139.8	Toll-like receptor 2	Q9QUN7
SAVVHEYAL	9	141.4	sterol O-acyltransferase 1	Q61263
AAPANPEMDN	11	141.7	bcl-2 homologous antagonist/killer	O08734

GAISNYVSRTN	14		141.8	Calcium-responsive transactivator	Q8BW22
YAHENDITHF	10		142.1	Histone-lysine N-methyltransferase NSD2	Q8BVE8-4; Q8BVE8-2; Q8BVE8-1; Q8BVE8-3
TCLENGSFL	9		142.2	Protein Churchill OS=Mus musculus OX=10090	Q6DG52
TGIKNGVHFL	10		143.3	Prolactin regulatory element-binding protein	Q9WUQ2
CSISNDKFEYL	11		144.2	Transcription factor Dp-1 OS=Mus musculus OX=10090	Q08639
SSPLNDISTSV	12		144.6	Serine/threonine-protein kinase WNK1	P83741-2; P83741; P83741-3; P83741-5; P83741-4
VAVKNSGGFL	10		145	Extended synaptotagmin-2	Q3TZZ7-2; Q3TZZ7
SAPLNNRCV	9		145.4	Collagen type IV alpha-3-binding protein	Q9EQG9-1
VTLANKELL	9		145.7	squalene monooxygenase	P52019
KAITDIIEM	9		145.8	Rab5 GDP/GTP exchange factor	Q9JM13
RSLDNAESL	9		145.9	APC membrane recruitment protein 1	Q7TS75
TVIKNQHVL	9		146	Deoxyribonuclease-2-alpha	P56542
STLRNIRTV	9		146	Inhibitor of nuclear factor kappa-B kinase-interacting protein	Q9DBZ1
ACPFNGWYM	9		146	Nitric oxide synthase, inducible	P29477
SSVENIQRV	9		146.9	Junction plakoglobin	Q02257
FHVQNIDLL	9		147.2	ATPase family AAA domain-containing protein 5	Q4QY64
RTVENIKDPL	10		147.5	Cytoplasmic dynein 1 heavy chain 1	Q9JHU4
AAVRNCDGFL	11	Carbamidomethyl [C	147.5	iron-responsive element-binding protein 2	Q811J3
FAVISRHSL	9		147.8	Multidrug resistance-associated protein 1	O35379
VSPQNVHHSY	11		147.9	Isoform 3 of HBS1-like protein	Q69ZS7-3
EAIKNHEEL	9		148	45 kDa calcium-binding protein	Q61112-1; Q61112-2
RVVANSEEI	9		148.1	ATP-binding cassette sub-family D member 1	P48410
AALNNHREV	9		148.9	E3 ubiquitin-protein ligase MIB2	Q8R516; Q8R516-2
SMKTNREEL	9		149	Pericentrin	P48725-1; P48725-3
AQLRNLDNA	9		149.3	Putative ATP-dependent RNA helicase DHX33	Q80VY9
AGLHNISPQM	10		149.4	Transducin-like enhancer protein 4	Q62441
AMHRNLVDNI	10		150.1	plectin	Q9QXS1-6; Q9QXS1-13; Q9QXS1-7; Q9QXS1-9; Q9QXS1-5; Q9QXS1-14; Q9QXS1-12; Q9QXS1-3; Q9QXS1-2; Q9QXS1-10; Q9QXS1-15; Q9QXS1-11; Q9QXS1-16; Q9QXS1-4; Q9QXS1-1; Q9QXS1-8
NSVINVSNL	9		150.2	S-phase kinase-associated protein 2	Q9Z0Z3-1; Q9Z0Z3-2
ILHTNLVYL	9		151	Protein SSXT	Q62280
SGLINHQRI	9		152.4	zinc finger protein 24	Q91VN1
YMMVMDHHL	9		152.4	copper-transporting ATPase 1	Q64430
NGVINGAEL	9		152.8	Probable global transcription activator SNF2L2	Q6DIC0
RAPRNIHRHVI	11		152.9	Transcription factor 25	Q8R3L2-1; Q8R3L2-4; Q8R3L2-2; Q8R3L2-3; Q8R3L2-5
SQKENLNFL	9		153.5	ATPase family AAA domain-containing protein 2	Q8CDM1-2; Q8CDM1
AGFGNVNLGY	12		153.6	Lymphocyte antigen 6E	Q64253
KAVVSQDAL	9		154.3	GDNF-inducible zinc finger protein 1	Q4VBD9
DAPRNLILI	9		154.4	Oxidoreductase NAD-binding domain-containing protein 1	Q8VE38; Q8VE38-2
IGIANFAEQI	10		154.5	Kelch-like ECH-associated protein 1	Q9Z2X8
RVLYNLEVL	9		154.8	Ubiquitin carboxyl-terminal hydrolase 24	B1AY13
SSPENKNWLL	10		155.3	Denticleless protein homolog	Q3TLR7-2; Q3TLR7-1
AMWANEQAL	9		155.4	Cytochrome b-245 light chain	Q61462; Q61462-2
FSEKNTDHI	9		155.5	Ras-related protein Rab-43	Q8CG50
YAITTLHNL	9		155.5	Catenin beta-1	Q02257; Q02248
GALRSREFL	9		155.6	E3 ubiquitin-protein ligase RNF26	Q8BUH7; Q8BUH7-2

TGPFNLQLI	9		156.5	CAD protein	B2RQC6-2; B2RQC6
FQAINAGHI	9		157.4	Protein TANC1	Q0VGY8-2; Q0VGY8
SQLTNVILHL	10		157.5	Mitochondrial dynamics protein MID51	Q8BGV8
STLRLLTTI	9		158.1	Sister chromatid cohesion protein PDS5 homolog B	Q4VA53-3; Q4VA53-2; Q4VA53
GQLSNGDHHF	11		158.5	YTH domain-containing family protein 1	P59326
SQFPNAEKM	9		158.7	Dedicator of cytokinesis protein 2	Q8C3J5
STVENFSQL	9		158.8	Nucleolar pre-ribosomal-associated protein 1	Q571H0
QTVLNPVTM	9		159.2	Max-binding protein MNT	O08789
YGLKNHKLL	9		159.9	Poly [ADP-ribose] polymerase 8	Q3UD82
AGPENSSKI	9		160	autophagy-related protein 2 homolog B	Q80XK6-5; Q80XK6
YTIELLDTV	9		160	Small G protein signaling modulator 2	Q80U12; Q80U12-2
KGIGNKTEI	9		161.1	Histone-lysine N-methyltransferase NSD3	Q6P2L6-2; Q6P2L6-4; Q6P2L6
KNLLNVDKI	9		161.5	Dedicator of cytokinesis protein 10	Q8BZN6-3; Q8BZN6-4; Q8BZN6-1; Q8BZN6-2
VALLNETESVL	11		161.7	Metastasis-associated protein MTA3	Q924K8; Q924K8-2
FSQENTEKI	9		162	DEP domain-containing protein 1A	Q8CIG0-2; Q8CIG0
MAPQNLSTFCI	11		162.1	DnaJ homolog subfamily B member 11	Q99KV1
KQGQNLWFL	9		162.2	Tyrosine-protein kinase BAZ1B	Q9Z277; Q9Z277-2
FALELQHAL	9		162.5	Patatin-like phospholipase domain-containing protein 7	A2AJ88-3; A2AJ88; A2AJ88-2
IAPKNPTISL	10		162.6	TOM1-like protein 1	Q923U0
TAIENSWIHL	10		162.9	Methyltransferase-like protein 16	Q9CQG2; Q9CQG2-2
FSLAHITQL	9		163.8	Ras suppressor protein 1	Q01730
AAPRSFIFL	9		164.7	N6-adenosine-methyltransferase subunit METTL14	Q3UIK4-1; Q3UIK4-2
YQYQNIFGPLV	11		165.1	Regulator of nonsense transcripts 1	Q9EPU0-1; Q9EPU0-2
KMFENEFLL	9		165.6	active breakpoint cluster region-related protein	Q5SSL4; Q5SSL4-2; Q5SSL4-4; Q5SSL4-3
TSAYNTEVL	9		166.2	Friend leukemia integration 1 transcription factor	P26323
KCIENLEEL	9		166.8	Protein phosphatase 1 regulatory subunit 7	Q3UM45
AGLVNIVSF	9		167	NF-related matrix-associated actin-dependent regulator of chromatin subfamily A-like prc	Q8BJL0-3; Q8BJL0; Q8BJL0-2
YCYDNIHFM	9		167.6	Basic leucine zipper and W2 domain-containing protein 1	Q91VK1; Q9CQC6
GQMSNGEHHY	11		167.6	YTH domain-containing family protein 3	Q8BYK6-2; Q8BYK6-3; Q8BYK6
IAIKLAEYL	9		167.6	NAD-dependent malic enzyme, mitochondrial	Q99KE1
LSPINIQLL	9		167.6	WD repeat-containing and planar cell polarity effector protein fritz homolog	Q8C456-2; Q8C456
SGPLDSTFI	9		167.7	Suppressor APC domain-containing protein 2	Q9D818-2; Q9D818; Q9D818-3
GTFVNTELI	9		169.3	Serine/threonine-protein kinase Chk2	Q9Z265
RTPQNTISI	9		169.3	G-protein coupled receptor 176	Q80WT4
AAVVMLDRI	9		169.4	E3 ubiquitin-protein ligase RNF213	E9Q555
IGVRNENTFL	10		170	UTP--glucose-1-phosphate uridylyltransferase	Q91ZJ5-2; Q91ZJ5-1
SSCENGTFF	9		170	B-cell lymphoma 6 protein homolog	P41183
FFHNNMEYM	9		170.2	NADH-ubiquinone oxidoreductase chain 5	P03921
FSVENKWRL	9		170.4	Cytochrome c oxidase subunit 7C, mitochondrial	P17665
SAPSSPAEYI	10		171.3	Probable ATP-dependent RNA helicase DDX31	Q6NZQ2
ICIGNTAYI	9		172.9	AP-5 complex subunit mu-1	Q8BJ63
RGPVNLQHLI	10		173.4	Leucine-rich repeat and fibronectin type-III domain-containing protein 4	Q80XU8
FNVRNGYGFI	10		174.7	Nuclease-sensitive element-binding protein 1	Q9JKB3; Q9Z2C8-2; Q9JKB3-2; P62960; Q9Z2C8
KAPHNVKQDF	11		175.1	MAX gene-associated protein	A2AWL7-2; A2AWL7-3; A2AWL7-4; A2AWL7
SQLIHLIYI	9		175.4	FAST kinase domain-containing protein 5, mitochondrial	Q7TMV3
YSYQSRFHL	9		175.4	Splicing factor 3B subunit 3	Q921M3-2; Q921M3
AVLRNLCLL	9	Carbamidomethyl [C	175.5	Peroxisomal acyl-coenzyme A oxidase 1	Q9R0H0-1; Q9R0H0-2
SGHTNAVATV	10		176	Pleiotropic regulator 1	Q922V4

ISPQNGRYQI	10	176.3	Mitogen-activated protein kinase kinase kinase 8	Q07174
RSVNNMSASP	11	176.8	Histone acetyltransferase p300	B2RWS6
NQITDLEYL	9	179.8	leucine-rich melanocyte differentiation-associated protein	Q9D9B4
KTIKNTVCV	9	180.1	Kinesin-1 heavy chain	Q61768
ITPLNKDHI	9	180.4	nuclear pore complex protein nup155	Q99P88
YAVNNQFTMG	13	181.2	Histone-arginine methyltransferase CARM1	Q9WVG6; Q9WVG6-2
AAVINPPQACI	11	181.4	Pyruvate dehydrogenase protein X component, mitochondrial	Q8BKZ9
SSLMKATVL	9	181.9	7-methylguanosine phosphate-specific 5'-nucleotidase	Q3UFY7-3; Q3UFY7; Q3UFY7-2
RSFSLRDYL	9	181.9	Zinc finger and BTB domain-containing protein 45	Q52KG4
TNPLNGQYI	9	182	Neurogenic locus notch homolog protein 2	O35516; O35516-2
GNMRNFSDYI	10	182	Coiled-coil domain-containing protein R3HCC1L	Q8BJM3
AAPTNRQIEILE	13	183.5	Nuclear pore complex protein Nup160	Q9Z0W3
SAPTARVFM	9	183.6	Pre-mRNA-processing factor 6	Q91YR7
RSLISMEEI	9	183.9	Translin-associated protein X	Q9QZE7
FSGANKEKL	9	184.1	thioredoxin	P10639
FTIRNKKGTEL	11	184.3	merlin	P46662-1; P46662-2
HAMNNLNGTE	11	184.5	RNA-binding protein 47	Q91WT8-1; Q91WT8-2
SQLVKADEM	9	184.6	DnaJ homolog subfamily C member 3	Q91YW3
SSFENLHFENL	11	185.7	DNA helicase B	Q6NVF4
SGCLNGSFL	9	185.9	Probable E3 ubiquitin-protein ligase HERC4	Q6PAV2-2; Q6PAV2
SGVSNFAQLI	10	186.6	Receptor-type tyrosine-protein phosphatase kappa	P35822
SSIANLKVSLL	11	186.8	cyclin-dependent kinase 2-interacting protein	Q9D0V8; Q9D0V8-3; Q9D0V8-2
TTHSNHEVL	9	188.3	Pyrroline-5-carboxylate reductase 3	Q9DCC4
SAAFNQLPHL	10	188.6	Cytoplasmic polyadenylation element-binding protein 4	Q7TN98-5; Q7TN98-1; Q7TN98-2; Q7TN98-3; Q7TN98-4
RQFPNRDAL	9	190.1	RNA-binding protein 5	Q91YE7-2; Q91YE7
MAPENKVADW	11	190.2	Centrosomal protein of 170 kDa	Q6A065
STCVNSYAI	9	191	ATP-binding cassette sub-family G member 2	Q7TMS5
RQGANINEI	9	191.2	Poly(RC)-binding protein 1	P60335
SVIQNNEHVYM	11	191.7	FERM domain-containing protein 6	Q8C0V9-2; Q8C0V9-3; Q8C0V9-1
SAVIDMENM	9	191.8	protein YIPF3	Q3UDR8
ASPEGQDYL	9	192.1	tRNA-splicing ligase RtcB homolog	Q99LF4
YMVEHQEYV	9	192.5	Ephrin-A1	P52793
FVISNYREQL	10	192.7	uncharacterized protein C5orf34 homolog	Q3UJC8-1; Q3UJC8-2
SMGVNDIDI	9	193	Zinc finger CCCH domain-containing protein 15	Q3TIV5; Q3TIV5-2
RQPQNPPATI	10	193.8	Telomeric repeat-binding factor 2	O35144-1; O35144-2
TCVSNGPFI	9	194.3	Polypyrimidine tract-binding protein 1	P17225
TQFPNLEVL	9	194.8	Toll-like receptor 13	Q6R5N8
VIAINQDPL	9	195	Alpha-galactosidase A	P51569
SAILGQMTL	9	195.9	Multidrug resistance-associated protein 5	Q9R1X5
GAIRNACQML	10	196	Cullin-3	Q9JLV5
CSGENFVEI	9	196.4	Cell division cycle-associated protein 2	Q14B71
WSPHNETIL	9	196.5	Histone-binding protein RBBP7	Q60973; Q60972
RSLRHVNIDHL	11	197.7	Eukaryotic translation initiation factor 3 subunit H	Q91WK2
AGLRNAVSQM	10	198.4	Coiled-coil domain-containing protein 57	Q6PHN1
FGLQNDHCVF	11	198.9	Transforming acidic coiled-coil-containing protein 3	Q9JJ11
YAIQLITAA	9	199.1	RuvB-like 2	Q9WTM5
FSIVGGPVL	9	199.1	TATA box-binding protein-associated factor RNA polymerase I subunit C	Q6PDZ2

GGLLNITKV	9		199.6	Isoform 2 of Microfibrillar-associated protein 3-like	Q9D3X9-2; Q9D3X9-1
FVIRNIVEA	9		199.6	40S ribosomal protein S26	P62855
VTVENVQEL	9		200	Kelch-like protein 12	Q8BZM0; Q8BZM0-2
TQNVNQAKM	9		200.7	Melanoma-associated antigen D1	Q9QYH6
AANENLKHI	9		202.2	poly [ADP-ribose] polymerase 14	Q2EMV9; Q2EMV9-2
YKNVNQEVV	9		202.3	Oxidoreductase htatip2	Q9Z2G9-2; Q9Z2G9-1
FQLINVEDF	9		203.3	Intron-binding protein aquarius	Q8CFQ3
YASENVNKL	9		203.4	Ras-related protein Rab-1A	P62821; Q9D1G1
QTILNSTMM	9		203.5	Tetratricopeptide repeat protein 27	Q8CD92-2; Q8CD92
SAVSNNYIQTL	12		203.8	Catenin delta-1	P30999-2; P30999; P30999-3
RNLGNTCYM	9	Carbamidomethyl [(	203.9	Putative ubiquitin carboxyl-terminal hydrolase 50	Q8C2S0; Q6P8X6-1; Q6P9L4; Q80U87
IQVKNEVSL	9		204.3	Neutral amino acid transporter B(0)	P51912
VSLRNSISNFL	11		205.3	Acetyl-CoA carboxylase 1	Q5SWU9-2; Q5SWU9-1
KTNQNFTHL	9		206	NACHT, LRR and PYD domains-containing protein 3	Q8R4B8-3; Q8R4B8-1
FAHIDGDHL	9		206.4	Pre-mRNA-splicing factor ATP-dependent RNA helicase DHX15	O35286
RAPTSTDTPI	10		207.3	Tyrosine-protein phosphatase non-receptor type 18	Q61152
SAPRNFVENF	10		207.3	elongator complex protein 2	Q91WG4-2; Q91WG4
AAPISPWTM	9		209.3	Zinc finger protein 746	Q3U133
FTVSNFVDNL	10		209.6	Neuroligin-2	Q69ZK9
YQYLLVTAL	9		210.3	Serine palmitoyltransferase small subunit A	Q8R207
RQIQNQLEETL	11		210.7	Brain-specific angiogenesis inhibitor 1-associated protein 2	Q8BKX1-3; Q8BKX1; Q8BKX1-2; Q8BKX1-4
CAPSSRYTL	9		210.9	Alpha/beta hydrolase domain-containing protein 17C	Q8VCV1
SSPTNSAITQL	11		211.2	Zinc finger E-box-binding homeobox 2	Q9R0G7
ASLRDKDTL	9		211.6	isoleucine--tRNA ligase, cytoplasmic	Q8BU30
VSVQHVCTI	9		212.1	Hexokinase-1	P17710; P17710-2; P17710-4; P17710-3
KACINQHLM	9		212.3	E3 ubiquitin-protein ligase RNF123	Q5XPI3
NQYENAEKL	9		214.4	Amyloid protein-binding protein 2	Q9DAX9
RSLLNWRTKL	10		215.2	pre-rRNA processing protein FTSJ3	Q9DBE9
FCIENLQKL	9		215.3	Acetyl-CoA carboxylase 1	Q5SWU9-2; Q5SWU9
FGPINSVAF	9		215.5	Eukaryotic translation initiation factor 3 subunit I	Q9QZD9
GQGRNPFFL	9		215.8	integrator complex subunit 6-like	Q6PCM2-1; Q8BND4; Q6PCM2-3; Q6PCM2-2
QAPINFTSRL	10		216.3	DDB1- and CUL4-associated factor 1	Q80TR8-1; Q80TR8-4; Q80TR8-2
SLLQNGVNL	9		216.6	Erbin	Q80TH2-2; Q80TH2; Q80TH2-1
AQVENVQRI	9		216.9	Centrosomal protein of 83 kDa	Q9D5R3-1
SLTMDGGLRN	14		217.5	Serine/threonine-protein kinase TBK1	Q9WUN2
GAIVDQMTL	9		217.7	Lysosomal alpha-mannosidase	O09159
SMAYPNLVAM	10		218.3	Immunoglobulin-binding protein 1	Q61249
SMIRLSESM	9		220.5	DNA replication licensing factor MCM6	P97311
YALKSQEML	9		221.6	Fanconi anemia group B protein homolog	Q5XJY6
KGQLNADEI	9		223.7	Eukaryotic translation initiation factor 4 gamma 2	Q62448
YGISNEKPEV	10		225	RNA polymerase II-associated factor 1 homolog	Q8K2T8
RALKNCRLEL	10		225.7	Carbonyl reductase [NADPH] 1	P48758
RCPENAFFL	9		226.6	Unconventional myosin-Ic	Q9WTI7-1
SQVSDYDYF	9		227.5	Protein MTSS 1	Q8R1S4; Q8R1S4-2
VAGQNVNITL	10		227.9	E1A-binding protein p400	Q8CHI8-4; Q8CHI8-3; Q8CHI8-2; Q8CHI8-5; Q8CHI8
GAVTLQEYL	9		228.8	ARF GTPase-activating protein GIT1	Q68FF6
SSLLNNKHFLI	11		228.8	Plexin-D1	Q3UH93
QVLENLTEL	9		229.7	MAGUK p55 subfamily member 6	Q9JLB0; Q9JLB0-2

FSKKNFESL	9	230	Dolichyl-diphosphooligosaccharide--protein glycosyltransferase subunit 2	Q9DBG6
SAISNASGERL	11	231.5	E3 ubiquitin-protein ligase mib1	Q80SY4
KSIVNTSPP	9	234.9	RNA-binding protein 33	Q9CXK9-1
LSGEFQIVNPH	13	235.2	Ribonucleoside-diphosphate reductase large subunit	P07742
KTIYNVEHL	9	235.7	E3 ubiquitin-protein ligase RNF103	Q9R1W3
KQVENVVRM	9	236	Protein FAM173B	Q9D1Z3
YMADNANYFI	10	236.2	WD repeat and FYVE domain-containing protein 3	Q6VNB8; Q6VNB8-2
RSGGNLEVM	9	236.8	COP9 signalosome complex subunit 5	O35864
NAPVNPTRAEI	11	236.8	phorbol-12-myristate-13-acetate-induced protein 1	Q9JM54
AMMAKAEYL	9	237.1	26S proteasome non-ATPase regulatory subunit 6	Q99JI4
TQFTNKCHI	9	237.6	glycerol kinase	Q64516-3; Q9WU65
SLPTNLIHL	9	238.7	E3 ubiquitin-protein ligase UBR2	Q6WKZ8-1; Q6WKZ8-3; Q6WKZ8-2
ICIANTHLL	9	239.6	protein angel homolog 2	Q8K1C0-1
NSISNPVTKEM	11	240.3	mRNA-capping enzyme	O55236
SSLPNLAISTI	11	240.9	E3 ubiquitin-protein ligase RNF213	E9Q555
FGVKSLVYM	9	242.6	Sphingolipid delta(4)-desaturase DES1	O09005
VNVENQTKI	9	244.4	Armadillo repeat-containing protein 10	Q9D0L7; Q9D0L7-2
VAVYNHYKRI	10	245.7	ATP-dependent Clp protease ATP-binding subunit clpX-like, mitochondrial	Q9JHS4
RTISNPEVVM	10	246	Afadin	Q9QZQ1; Q9QZQ1-2
SSISNSLENAL	11	247.7	Zinc finger CCHC domain-containing protein 14	Q8VIG0; Q8VIG0-3; Q8VIG0-2
HTVQNADQV	9	248	antigen peptide transporter 2	P36371
NCILNAEAL	9	248.7	Prolyl 3-hydroxylase 1	Q3V1T4
EQHINIDTL	9	248.8	Phospholipid-transporting ATPase 11C	Q9QZW0; Q9QZW0-2
ISVSNMPTRTI	11	249	uncharacterized protein KIAA1551	Q5DTW7-3; Q5DTW7
SGIINFIHF	9	249.4	Kinetochore protein Nuf2	Q99P69-2; Q99P69
RAIDNAADL	9	249.7	Sorting nexin-8	Q8CFD4
ATILNLERV	9	250.7	Conserved oligomeric Golgi complex subunit 4	Q8R1U1
FQKQNVTIM	9	250.7	Nitric oxide synthase, inducible	P29477
SQAVNKQQI	9	251	Isoform 2 of Cyclin-F	P51944-2; P51944; P51944-3
GGPANFGYPD	14	251	E3 ubiquitin-protein ligase DTX3L	Q3UIR3
FVRINQDYI	9	251.6	S1 RNA-binding domain-containing protein 1 OS=Mus musculus OX=10090	Q497V5-2; Q497V5; Q497V5
AALTLLEKM	9	251.7	Exocyst complex component 6	Q8R313
GGHMNQHHL	9	252	transcription termination factor 2	Q5NC05
DGSAIRNGTDM	11	252.7	Coiled-coil domain-containing protein 189	Q6NZQ0
SALQSLLHL	9	252.9	Importin subunit alpha-5	Q60960
ILHENFTTV	9	253.2	Cytoplasmic dynein 1 light intermediate chain 2	Q6PDL0
RAIYNNMKQFI	11	253.4	Sarcoplasmic/endoplasmic reticulum calcium atpase 3	Q64518-3; Q64518-2; O55143-2; Q64518; O55143-1; Q8R429
YALALISRI	9	253.5	Probable G-protein coupled receptor 146	Q99LE2
AAMLLQQVM	9	254.5	E3 UFM1-protein ligase 1	Q8CCJ3-3; Q8CCJ3-1
FGIIDVTEV	9	255.2	Choline/ethanolaminephosphotransferase 1	Q8BGS7
SSPENKNWLLA	12	256.1	Denticleless protein homolog	Q3TLR7-2; Q3TLR7
RAPENLTLSNL	11	256.9	Cytokine receptor common subunit gamma	P34902
FSIFNRSIDAF	11	257.7	Kit ligand	P20826; P20826-2
FHIGINRYEL	10	258.8	Extracellular serine/threonine protein kinase FAM20C	Q5MJS3
AQLQNQQSF	9	259.4	Coiled-coil-helix-coiled-coil-helix domain-containing protein 2	Q9D1L0
SSVVGVWYL	9	259.7	Minor histocompatibility antigen H13	Q9D8V0; Q9D8V0-3; Q9D8V0-4
RIVENVNVI	9	260.1	Elongation factor 2	P58252

HSLVLVDEL	9		260.9	DNA mismatch repair protein MSH6	P54276
VGLENVSNV	9		261.6	Transformation/transcription domain-associated protein	Q80YV3
RSPVNAPGKW	12		261.9	Molybdenum cofactor sulfurase	Q14CH1
SALVHSQEL	9		262.8	baculoviral IAP repeat-containing protein 3	O08863
AIVRNLESL	9		263	28S ribosomal protein S6, mitochondrial	P58064
SQVINPTAITV	11		263.2	Host cell factor 1	Q61191
SALEFLTHL	9		263.5	BRCA1-associated ATM activator 1	Q8C3R1-2; Q8C3R1
HALNDMTSI	9		264.3	Tropomyosin alpha-1 chain	P21107; P58771-1
IGINLTDPM	9		264.6	Putative deoxyribonuclease TATDN1	Q6P8M1-2; Q6P8M1
IQWPNGITL	9		264.8	Very low-density lipoprotein receptor	P98156; P35951
TALRLITAL	9		265.3	Mediator of RNA polymerase II transcription subunit 23	Q80YQ2-2; Q80YQ2
VSARNIMLL	9		266.1	cold shock domain-containing protein E1	Q91W50
YAFKNPIYS	9		269.3	terminal uridylyltransferase 7	Q5BLK4
RAPTGYDVV	9		270.4	Multivesicular body subunit 12B	Q6KAU4
FMYTTPFTL	9		270.6	Dedicator of cytokinesis protein 8	Q8C147
SSMKNFKAFF	10		270.8	Anaphase-promoting complex subunit 4	Q91W96
FGLHLIDFM	9		271.6	Condensin complex subunit 2	Q8C156
VQLQLLTAI	9		273.1	AP-1 complex subunit beta-1	O35643
RNLGNTCFM	9	Carbamidomethyl [C	274.4	Ubiquitin carboxyl-terminal hydrolase 2	O88623-2; O88623-1; O88623-4; O88623-3; Q91W36
FSLENVPEGEL	11		274.5	NEDD4-binding protein 1	Q6A037
SGPRDHVFI	9		275	Legumain	O89017
SSPSSPTAL	9		275.5	lysophospholipid acyltransferase LPCAT4	Q80Y50-5; Q80Y50-2; Q6NVG1; Q80Y50-4; Q80Y50-3; Q80Y50-1
FSGTNDPCAL	10		276.2	Macrophage migration inhibitory factor	P34884
RAPVKYDHI	9		276.8	Protein salvador homolog 1	Q8VEB2
TSVVLLTEM	9		277.5	AP-1 complex subunit gamma-1	P22892
SQHPNVIHL	9		277.5	Serine/threonine-protein kinase PDIK1L	Q8QZR7
FSPENYKDHSI	11		277.9	Little elongation complex subunit 2	Q3UZ18-1; Q3UZ18-2
YEGINVDVI	9		279.7	Probable ATP-dependent RNA helicase DDX52	Q8K301
AANVNAKDM	9		281.1	Tankyrase-1	Q6PFX9-1
MCLRNRDHL	9		281.1	Mediator of RNA polymerase II transcription subunit 23	Q80YQ2
RAYTKFYTL	9		281.2	DENN domain-containing protein 4C	A6H8H2-1; A6H8H2-2
GMHQNTLRI	9		282.2	60S ribosomal export protein NMD3	Q99L48-1; Q99L48-2
VAQTNQNQL	9		283.3	Mediator of RNA polymerase II transcription subunit 23	Q80YQ2-2; Q80YQ2
NQVVLLDTL	9		283.6	Biogenesis of lysosome-related organelles complex 1 subunit 6	Q9R0C0-1
RSLVSLTHL	9		283.8	Toll-like receptor 13	Q6R5N8
RALINSRTF	9		284	Putative Polycomb group protein ASXL1	P59598
VDVTNTTFL	9		284.9	Coatomer subunit epsilon	O89079
AAVGNCAEHM	10		286.2	Coiled-coil-helix-coiled-coil-helix domain-containing protein 5	Q9CQP3
SSVRDRDLL	9		288	Nesprin-3	Q4FZC9-1; Q4FZC9-2
AIIQNHREVM	10		288.7	Ral GTPase-activating protein subunit alpha-2	A3KGS3-1
SAVYNSFRAVL	11		288.9	Peroxisomal membrane protein pex13	Q9D0K1
RSLLLLAPL	9		289.2	Procollagen-lysine,2-oxoglutarate 5-dioxygenase 1	Q9R0E2
WAVQNIRSV	9		289.3	E3 ubiquitin-protein ligase HERC2	Q4U2R1-2; Q4U2R1
SSPYSPAYL	9		291.4	CREB-regulated transcription coactivator 2	Q3U182
HSCWNLSTI	9		291.5	NACHT, LRR and PYD domains-containing protein 3	Q8R4B8-2; Q8R4B8-3; Q8R4B8
FSPLNPVRVHI	11		292.3	Heterogeneous nuclear ribonucleoprotein F	O35737; Q9Z2X1-2; Q9Z2X1-1

FSPLNPMRVHI	11	292.3	heterogeneous nuclear ribonucleoprotein H2	P70333
FGLQNDHCVF	10	295.3	Transforming acidic coiled-coil-containing protein 3	Q9JJ11
FVHENIETF	9	296.5	Transmembrane protein 245	B1AZA5
SSVRDTDSL	9	296.7	LisH domain-containing protein ARMC9	Q9D2I5-9; Q9D2I5-4; Q9D2I5-3; Q9D2I5-2; Q9D2I5-1; Q9D2I5-5; Q9D2I5-8
SSLLNSEFC	9	297	Protein flightless-1 homolog	Q9JJ28
YAFWNKEDF	9	297.3	transmembrane protein 180	Q6PDE8
FCFENVKYM	9	299.8	Type II inositol 1,4,5-trisphosphate 5-phosphatase	Q8K337-1
LINQNSDYL	9	299.8	TELO2-interacting protein 1 homolog	Q91V83
NQMVGIEYI	9	300.5	Mediator of RNA polymerase II transcription subunit 6	Q921D4
ALINKDFL	8	302.2	Transmembrane protein 82	Q8R115
FMSRTNRPPL	10	302.5	60S ribosomal protein L18	P35980
SSAKNFITKM	10	302.6	UDP-glucose:glycoprotein glucosyltransferase 1	Q6P5E4
SSPTDKHTL	9	303.5	Plasma membrane calcium-transporting ATPase 4	Q6Q477-2; Q6Q477; G5E829; Q9R0K7
RQGKNLMFL	9	303.8	Asparagine--tRNA ligase, cytoplasmic	Q8BP47
SGITNSASSTL	11	304.4	Cell division cycle 5-like protein	Q6A068
SSVSKQETI	9	304.5	Transcriptional repressor p66-beta	Q8VHR5
SCILNGTVML	10	305.7	Sodium bicarbonate cotransporter 3	Q8BTY2
SCLRNVTQTL	10	306.1	Rab3 GTPase-activating protein non-catalytic subunit	Q8BMG7
GAVFNQVAF	9	306.3	Splicing factor 3B subunit 3	Q921M3-2; Q921M3
DQVENHEFL	9	307.2	DNA mismatch repair protein MSH2	P43247
QQVLNVISL	9	308.8	Testis-expressed protein 10	Q3URQ0
AQVQNSEQLL	10	309	rab GTPase-binding effector protein 2	Q91WG2-2; Q91WG2-3; Q91WG2-1
SQSMNFSLM	9	311.1	Clathrin interactor 1	Q99KN9; Q99KN9-2
RSGVNSELV	9	313.2	Protein DEK	Q7TNV0
TGILNPEGYTL	11	313.4	Poly [ADP-ribose] polymerase 2	O88554
AAVIAHDFL	9	313.6	G1/S-specific cyclin-D3	P30282
NAPQNPESKL	10	313.6	GON-4-like protein	Q9DB00
GSLVHISYL	9	313.8	DNA-directed RNA polymerase II subunit RPB1	P08775
NGVSNGLHL	9	316.3	WD and tetratricopeptide repeats protein 1	Q80ZK9
VTVENTKTI	9	316.5	Zinc finger CCCH domain-containing protein 13	E9Q784
YMFEAREFL	9	317.6	staphylococcal nuclease domain-containing protein 1	Q78PY7
WSLTHLTAL	9	318.7	CCR4-NOT transcription complex subunit 6	Q8K3P5-2; Q8K3P5; Q8VEG6-2; Q8VEG6-3; Q8VEG6
AAISNKITSCIF	13	319	Multifunctional protein ADE2	Q9DCL9
FQLRSFDQL	9	319.4	Hypoxia-inducible factor 1-alpha	Q61221-2; Q61221
SQLANEKLTQL	11	319.4	Rho-associated protein kinase 1	P70335-2; P70335-1
SCFQNNVEI	9	319.8	Protein lin-9 homolog	Q8C735
ASLVNKIGL	9	321.7	Uroporphyrinogen-III synthase	P51163
FSVENKWRLL	10	322	Cytochrome c oxidase subunit 7C, mitochondrial	P17665
STVQNAEAYVI	11	322.9	Ubiquitin carboxyl-terminal hydrolase 33	Q8R5K2-2; Q8R5K2
SSVTNLFSNPF	11	323.3	85/88 kDa calcium-independent phospholipase A2	P97819-2; P97819
SAPKYIDYL	9	324.3	mob kinase activator 1b	Q8BPB0; Q921Y0-1
SGLRNLREPIG	12	324.8	B-cell CLL/lymphoma 9 protein	Q9D219
GCLVNRDLV	9	325	Protein KIAA0556	Q8C753-3; Q8C753-4; Q8C753; Q8C753-2
NCIVNVFAV	9	325.5	Dynein light chain Tctex-type 3 OS=Mus musculus OX=10090	P56387
VQNINIENL	9	326.6	Protein S100-A8	P27005
TMDGGLRNVD	12	326.8	Serine/threonine-protein kinase TBK1	Q9WUN2

FGLRSIYSL	9	328.4	ER membrane protein complex subunit 3	Q99KI3
AGIENDEAF	9	330.5	Clathrin light chain A	O08585
AGIENYFIGL	10	330.6	C-type lectin domain family 5 member A	Q9R007; Q9R007-2; Q9R007-1
ALHVNGESL	9	331.8	histone-lysine N-methyltransferase EHMT1	Q5DW34-2; Q5DW34; Q5DW34-3
AIMPNGDHI	9	332.1	Platelet-activating factor acetylhydrolase IB subunit alpha	P63005-1; P63005-2
CQYANIVNL	9	332.2	T-complex protein 11-like protein 2	Q8K1H7
TLAMNATTL	9	332.5	Glutamate receptor ionotropic, kainate 5	Q61626
SHVLNDTHM	9	332.6	Plexin-C1	Q9QZC2
IMTQNVERI	9	335.4	vesicle-associated membrane protein 8	O70404
YMHRNREIT	9	335.4	ubiquitin carboxyl-terminal hydrolase 25	P57080-1
HCLANVSAM	9	335.7	toll-like receptor 4	Q9QUK6
YVVDNIDHL	9	336.9	E3 ubiquitin-protein ligase RFWD2	Q9R1A8
ACLFNQEAL	9	338.9	Nesprin-1	Q6ZWR6-4; Q6ZWR6
NSMVLFDHM	9	340.5	DNA topoisomerase 2-beta	Q64511
GQHPNIITL	9	341.1	Ribosomal protein S6 kinase alpha-2	Q9WUT3; P18653; P18654
VSPVSKTAM	9	344.2	nuclear factor related to kappa-B-binding protein	Q6PIJ4
NSPFLRDFM	9	344.3	Isoform 4 of GRAM domain-containing protein 1B	Q80TI0-4; Q80TI0-1; Q80TI0-3; Q80TI0-2
PTLLNVDTI	9	345.2	Vacuolar protein sorting-associated protein 8 homolog	Q0P5W1-2; Q0P5W1; Q0P5W1-3
YQVGNLAGHT	11	345.3	ABI gene family member 3	Q8BYZ1-2; Q8BYZ1
VGVINLDGL	9	346.1	NADH-ubiquinone oxidoreductase chain 6	P03925
RALFNGAQKL	10	347.7	Protein transport protein Sec61 subunit alpha isoform 1	P61620; Q9JLR1
NSVVNPNKATI	11	348	OTU domain-containing protein 5	Q3U2S4-2; Q3U2S4-1
ISQVCNFSHL	10	351.7	GRB2-associated-binding protein 3 OS=Mus musculus OX=10090	Q8BSM5
SALILRTKL	9	352.6	Tetratricopeptide repeat protein 27	Q8CD92-2; Q8CD92
VGVVSVEFI	9	352.8	Next to BRCA1 gene 1 protein	P97432
HALDNGLFTL	10	353.3	Protein FRG1	P97376
NGVINHSETI	10	356.1	FH1/FH2 domain-containing protein 3	Q76LL6-2; Q76LL6-4; Q76LL6; Q76LL6-3
QMHQNFEEQM	10	357.9	TBC1 domain family member 15	Q9CXF4
FAPNSVHYL	9	359.6	Ran-binding protein 17	Q9EPK7-2; Q99NF8; Q9EPK7
KNMQNVEHV	9	360.8	proteasome subunit beta type-1	O09061
FAAASREVL	9	361.4	Uncharacterized protein C19orf60 homolog	Q9CYZ6
SSIQLKTNFL	10	362	HEAT repeat-containing protein 5A	Q5PRF0-1
VAISNLPAATF	11	363.3	UDP-galactose translocator	Q9R0M8
SLVKNVAHM	9	364.6	Zinc finger protein 622	Q91VY9
ASMSNDFKSVI	11	367.8	Syntaxin-5	Q8K1E0-2; Q8K1E0-1
SSVKNVPENL	10	367.9	aminoacyl tRNA synthase complex-interacting multifunctional protein 2	Q8R010
FTDVNKTEM	9	368.3	Centrosome-associated protein 350	E9Q309
AGIENKFGLYL	11	368.5	Calcium-binding mitochondrial carrier protein Aralar2	Q8BH59; Q9QXX4
YSLPKLTCM	9	370.3	BTB/POZ domain-containing protein 9	Q8C726
FCHVNPEQVI	10	371.7	CTP synthase 2	P70303; P70303-3; P70303-2
SGPVSLQTL	9	372	WD repeat-containing protein WRAP73	Q9JM98
SAPRDLFDM	9	373.1	SHC-transforming protein 1	P98083-3; P98083-1; P98083-2
AAITNKYQLVF	11	373.9	RNA polymerase I-specific transcription initiation factor RRN3	B2RS91
VQVANITQA	9	374	Phosphatidylinositide phosphatase SAC2	Q8CDA1-3; Q8CDA1-2; Q8CDA1
RTVENVTVF	9	374.4	Synaptic vesicle membrane protein VAT-1 homolog	Q62465
FRLQNSTLM	9	375.2	GRIP1-associated protein 1	Q8VD04
SQLKNKEIAL	10	375.6	Peptide chain release factor 1, mitochondrial	Q8K126
AGVSNIIEF	9	376	Protein FAM111A	Q9D2L9

LCPANPTCI	9	379.1	Nuclear cap-binding protein subunit 1	Q3UYV9
AAPCNDLHA	9	379.8	Serine/threonine-protein kinase pim-1 OS=Mus musculus OX=10090	P06803
SQMTNLQELH	11	380.5	volume-regulated anion channel subunit LRRC8D	Q8BGR2
KIHVNGVDYL	10	380.6	28S ribosomal protein S9, mitochondrial	Q9D7N3
YAPSPNSDDF	10	381.1	Transcription factor 12	Q61286-2; Q61286-1
KLHLNLTEL	9	381.4	Nck-associated protein 1-like	Q8K1X4
VVISNAGHL	9	381.6	Zinc finger ZZ-type and EF-hand domain-containing protein 1	Q5SSH7-1; Q5SSH7-2
SAIKGLSAL	9	381.9	SRA stem-loop-interacting RNA-binding protein, mitochondrial	Q9D8T7-2; Q9D8T7
YIHINIGAL	9	382.8	probable ATP-dependent RNA helicase DDX5	Q61656
YMSQNAIEKI	10	384.7	Leucine-rich repeats and immunoglobulin-like domains protein 2	Q52KR2-1
AVLRNQIHV	9	384.9	COP9 signalosome complex subunit 1	Q99LD4; Q99LD4-2
ASLQNAEKT	9	385.4	Ubiquitin carboxyl-terminal hydrolase 38	Q8BW70
RASTNAMLI	9	385.4	Pre-mRNA-splicing factor ATP-dependent RNA helicase DHX15	O35286
YAPDSIYYL	9	386.6	Zinc finger MYM-type protein 2	Q9CU65
QLLNTDYL	8	387.8	Pericentriolar material 1 protein	Q9R0L6; Q9R0L6-2
FQEMNIELI	9	389	Actin-like protein 6A	Q9Z2N8
SSLLNKITNSL	11	390.7	O-phosphoseryl-tRNA(Sec) selenium transferase	Q6P6M7
YAMRAFADAL	10	390.8	T-complex protein 1 subunit epsilon	P80316
ASMLNDELSHI	11	391	E3 ubiquitin-protein ligase TRAF7	Q922B6
GGLTNGSGRY	11	391.3	pumilio homolog 2	Q80U58-3; Q80U58-2; Q80U58-1; Q80U78-2; Q80U78-1; Q80U78-3
AAPTNANSLNS	13	391.7	CCR4-NOT transcription complex subunit 4	Q8BT14; Q8BT14-2; Q8BT14-3
DALENLRVYL	10	392.2	B-cell lymphoma/leukemia 10	Q9Z0H7
YDVENGEFL	9	394.3	E3 ubiquitin-protein ligase UBR2	Q6WKZ8; Q6WKZ8-3; Q6WKZ8-2
HTIRNYPATV	10	395.2	Lysine-specific histone demethylase 1A	Q6ZQ88
YHPGVNGPPL	10	396.4	Proline-rich protein 3	Q811B5; Q80W00; Q80W00-2
FSLAPYRDYL	10	397.2	Uncharacterized protein C15orf39 homolog	Q3TEI4
KQVKNGPSL	9	405.9	Regulation of nuclear pre-mRNA domain-containing protein 2	Q6NXI6; Q6NXI6-2
YAFNMKATV	9	406.3	Heat shock cognate 71 kDa protein	P63017
YGYSNRVVDLI	12	406.6	glyceraldehyde-3-phosphate dehydrogenase	P16858
KTVVNKDVF	9	409.2	60S ribosomal protein L27	P61358
KTIQNGREL	9	409.3	Histone-lysine N-methyltransferase NSD3	Q6P2L6-2; Q6P2L6-4; Q6P2L6
SSHENRPLDLL	11	410	E3 ubiquitin-protein ligase HUWE1	Q7TMY8-4; Q7TMY8-3; Q7TMY8; Q7TMY8-2
SACTFGDPI	9	410.6	Cathepsin Z	Q9WUU7
YCIGNMQML	9	412.6	NFX1-type zinc finger-containing protein 1	Q8R151
VAPENEGRLVI	12	412.7	Transmembrane protein 43	Q9DBS1
SSSLNQEKI	9	413.8	Protein FAM208B	Q5DTT3
SAIRGQAEV	9	415	Putative Polycomb group protein ASXL1	P59598
SALKLYENL	9	415.9	N-alpha-acetyltransferase 30	Q8CES0-2; Q8CES0
YAVGNHDFIEA	12	416.3	PCI domain-containing protein 2	Q8BFV2
ASVLLQDHI	9	418.9	Atlastin-3	Q91YH5-1
TAPLNTQIPTL	11	419	B-cell CLL/lymphoma 9 protein	Q9D219
WSEVNQAVL	9	419.9	Glucose-induced degradation protein 8 homolog	Q9D7M1
GCLENLTSL	9	421.2	Leucine-rich repeat serine/threonine-protein kinase 2	Q5S006
AQYRLVTEI	9	421.3	probable ATP-dependent RNA helicase DDX10	Q80Y44
LSILNSNEHL	10	421.4	Centrosomal protein of 85 kDa	Q8BMK0
SCVLNGELPL	10	421.4	Probable ATP-dependent RNA helicase DDX56	Q9D0R4
SSVTGLTNI	9	422.6	Ras GTPase-activating-like protein IQGAP1	Q9JKF1

SALTQENLSLL	12		424	Roquin-1	Q4VGL6
YINFNVDKL	9		425.5	Deoxynucleotidyltransferase terminal-interacting protein 2	Q8R2M2
RALNNYRQML	10		427.9	Breast cancer type 2 susceptibility protein homolog	P97929
RSHENEAEAL	10		428.8	Unconventional myosin-Va	Q99104
AAYRNLGQNL	10		432.5	Protein RCC2	Q8BK67
AAPINPSDINM	12		434.7	Enoyl-[acyl-carrier-protein] reductase, mitochondrial	Q9DCS3
RSPINCLEHV	10		435	Membralin	Q8CIV2-1
VVMALGDYM	9		437.2	Eukaryotic initiation factor 4A-I	P60843
KTVVDIDTI	9		438.6	Peroxisomal 2,4-dienoyl-CoA reductase	Q9WV68
GQVKNYCLTL	10		439.5	Neutrophil cytosol factor 2	O70145
AGLTHLITM	9		443	Phosphoribosyl pyrophosphate synthase-associated protein 1	Q9D0M1; Q8R574
FQLRLAEVL	9		444	Nucleolar protein 6	Q8R5K4-2; Q8R5K4
KHTFRNTWYL	10		447.4	Collagen alpha-1(XVI) chain	Q8BLX7
FRMPVLREYL	10		447.5	Diacylglycerol O-acyltransferase 2	Q9DCV3
QSLVNLRELYL	11		449.4	Protein phosphatase 1 regulatory subunit 7	Q3UM45
KVVALYDYM	9		454.7	Tyrosine-protein kinase BTK	P35991
MADGVANVEH	11	1xOxidation [M1]	456.1	Cytosolic 5'-nucleotidase 3A	Q9D020; Q9D020-1
IVGINLTEM	9	1xOxidation [M9]	456.2	ELMO domain-containing protein 2	Q8BGF6
AAIKLQELL	9		456.7	Ribosome-binding protein 1	Q99PL5-1
YMWPNARISVI	11		457.3	Methylcrotonoyl-CoA carboxylase beta chain, mitochondrial	Q3ULD5
RHMQNSEII	9		457.5	Cohesin subunit SA-2	O35638
VSSINQEAL	9		462.6	Chromatin accessibility complex protein 1	Q9JKP8
TSATNGPSL	9		463.6	Helicase with zinc finger domain 2	E9QAM5
RVVRNQQSM	9		465	Tyrosine-protein phosphatase non-receptor type 9	O35239
AAVINPPQACII	12		468.2	Pyruvate dehydrogenase protein X component, mitochondrial	Q8BKZ9
IGIQNFPEGL	10		471	zinc transporter zip11	Q8BWY7-2; Q8BWY7-1; Q8BWY7-3
NAQFNREEL	9		473.7	protein LSM14 homolog B	Q8CGC4
YAYDGCDYI	9		473.9	H-2 class I histocompatibility antigen, K-B alpha chain	P01901; P04223; P04223-2
HSLTLMTNM	9		474.8	Baculoviral IAP repeat-containing protein 6	O88738-2; O88738; O88738-3
SAIHILDVL	9		475.1	Cap-specific mRNA (nucleoside-2'-O-)-methyltransferase 1	Q9DBC3
FSGTDRETL	9		476	Pecanex-like protein 3	Q8VI59-1
YAVNNQFTMG	12		476.2	Histone-arginine methyltransferase CARM1	Q9WVG6; Q9WVG6-2
SAPTNTGSTGI	12		477.4	Nuclear pore complex protein Nup54	Q8BTS4
EGINNLTHM	9		479.2	Liprin-beta-1	Q8C8U0-2; Q8C8U0-1; Q8C8U0-3
FVHQNVGEV	9		480.4	Interferon-induced very large GTPase 1	Q80SU7
TALINQRDL	9		484.5	Autophagy-related protein 2 homolog A	Q6P4T0
RSITNTTVC	9		485.6	Splicing factor 1	Q64213
SALVSSLHL	9		485.8	Coatomer subunit gamma-1	Q9QZE5
KQIRLLEAM	9		486.2	Tumor necrosis factor alpha-induced protein 2	Q61333
SMAGNIIPAI	10		486.5	SUMO-activating enzyme subunit 2	Q9Z1F9
IQYSNHREL	9		487	polypyrimidine tract-binding protein 3	Q8BHD7-2; Q8BHD7
ASLQNFNISNL	11		488.1	Serine/threonine-protein kinase WNK1	P83741-2; P83741; P83741-3; P83741-5; P83741-4
YQHIGLVVM	9		488.9	Integrin alpha-M	P05555-1; P05555-2
TVISNAVDHI	10		489	N-alpha-acetyltransferase 25, NatB auxiliary subunit	Q8BWZ3-2; Q8BWZ3
RQVVHTETL	9		490.7	Ankyrin repeat domain-containing protein 13D	Q6PD24
SAKNKDYM	8		494.1	bifunctional UDP-N-acetylglucosamine 2-epimerase/N-acetylmannosamine kinase	Q91WG8
YQPILRDHI	9		495	DNA (cytosine-5)-methyltransferase 1	P13864-2; P13864
KSPQKLTTL	9		496.5	ATP-dependent RNA helicase DDX18	Q8K363

SGSLNAQVI	9		500.2	Mitochondrial import receptor subunit TOM40 homolog	Q9QYA2
VTIRLLETI	9		500.9	Methylsterol monooxygenase 1	Q9CRA4
ASVDNLLHL	9		502.1	Beta-1,4-glucuronyltransferase 1	Q8BWP8-2; Q8BWP8
SAPKSISAL	9		502.3	Roquin-1	Q4VGL6
AAVVGLVCL	9		502.9	Ubiquitin-like modifier-activating enzyme 1	Q02053
RAVQDVESL	9		503.5	e3 ubiquitin-protein ligase rnf14	Q9JI90-1
KSNVNIGNL	9		506.8	Phosphatidylinositide phosphatase SAC2	Q8CDA1-3; Q8CDA1-2; Q8CDA1
VSPTGTHTL	9		507.9	Granulins	P28798
SGLTMITHL	9		508.1	G-protein coupled receptor-associated sorting protein 1	Q5U4C1; Q5U4C1-2
SAIVNLPGCSA	11		508.4	Coatomer subunit gamma-1	Q9QZE5
SVVQNQQQV	9		508.7	Nipped-B-like protein	Q6KCD5-1; Q6KCD5-4; Q6KCD5-2
SQMIQTEYL	9		510.3	Adhesion G protein-coupled receptor E1	Q61549
NQITNNQRI	9		512.3	Phosphoglycerate kinase 2	P09041; P09411
RACYNLAAL	9		517.3	Integral membrane protein GPR137	Q80ZU9
AHQVNLEYL	9		518.6	iron-responsive element-binding protein 2	Q811J3
VSLLDIDHL	9		521.7	HEAT repeat-containing protein 3	Q8BQM4
VSPTNPTKLQI	11		522.3	Disabled homolog 2-interacting protein	Q3UHC7-3; Q3UHC7-2; Q3UHC7
SSLWSLTHL	9		524.6	CCR4-NOT transcription complex subunit 6	Q8K3P5-2; Q8K3P5-1
RMNKNFERL	9		525.4	IQ motif and SEC7 domain-containing protein 2	Q5DU25
FQLEKIDYL	9		526.4	Kinesin-like protein KIF17	Q99PW8
DSHANGDSL	9		529.5	CMRF35-like molecule 8	Q6SJQ0-2; Q6SJQ0-1
SSLRSLNNFI	10		529.7	mitotic spindle assembly checkpoint protein MAD1	Q9WTX8-1; Q9WTX8-2
FQFASFPHM	9		530.6	Mediator of RNA polymerase II transcription subunit 23	Q80YQ2-2; Q80YQ2
YVSQNKEFL	9		531.3	Angiomotin-like protein 1	Q9D4H4-2; Q9D4H4
SQQRNFQLL	9		531.6	DNA repair protein Rad50	P70388; P70388-2; P70388-3
SSGLPRNTVIL	11		533.3	Stomatin-like protein 2, mitochondrial	Q99JB2
NQFVNKFNVL	10		534.9	COP9 signalosome complex subunit 6	O88545
SALLNDCIRHL	11		536.1	Protein Niban	Q3UW53
ATLVNHRLSI	10		537.6	poly [ADP-ribose] polymerase 14	Q2EMV9
FLNVNCEHI	9		538.5	DNA replication licensing factor MCM4	P49717
VCIINFQHL	9		539	Activating signal cointegrator 1 complex subunit 3	E9PZJ8-1
SGISLATHL	9		539	eIF-2-alpha kinase GCN2	Q9QZ05; Q9QZ05-3; Q9QZ05-4; Q9QZ05-2; Q9QZ05-6
VAVTNGGVEH	12		542.4	Leucine-rich repeat and calponin homology domain-containing protein 3	Q8BVU0
NAVKNHWNST	11		545.3	Myb-related protein B	P48972
VSLKNPVRIFV	11		550.9	Probable ATP-dependent RNA helicase DDX27	Q921N6-1
STHYNLHNL	9		551.9	lysosomal alpha-glucosidase	P70699
HSLVHKVFL	9		557.1	Pumilio homolog 3	Q8BKS9
RGIANSLEKL	10		559.6	Nesprin-1	Q6ZWR6-4; Q6ZWR6-1
CSNKNLEKL	9		561.3	Microtubule-actin cross-linking factor 1	Q9QXZ0
SSPLHNHTL	9		561.7	Zinc finger protein 281	Q99LI5
SGPRGVHFI	9		565	Cytochrome b-245 heavy chain	Q61093
SAVKNDYEMTI	11		567.8	Replication protein A 70 kDa DNA-binding subunit	Q8VEE4
AAPRHQSVYI	10		568.9	hemicentin-2	A2AJ76-1; A2AJ76-2
KEVVNKDVL	9		572.3	DNA replication licensing factor MCM7	Q61881
LAPVNIFKA	9		573.2	T-complex protein 1 subunit beta	P80314
AAVRLTELL	9		573.4	Baculoviral IAP repeat-containing protein 1e	Q9QUK4; Q9R016; Q9JIB6; Q9JIB3
AGLTHIITM	9	1xOxidation [M9]	575	Phosphoribosyl pyrophosphate synthase-associated protein 1	Q9D0M1

FAPRDPIPYL	10	575.8	U1 small nuclear ribonucleoprotein 70 kDa	Q62376-1; Q62376-2
SHLINHVNI	9	575.9	Protein SMG5	Q6ZPY2-2; Q6ZPY2-1
LAPTNLSSSRM	11	576.6	serine/arginine repetitive matrix protein 2	Q8BTI8; Q8BTI8-2; Q8BTI8-3
RQWANLMEKI	10	576.7	Apolipoprotein E	P08226
VTPFNIDRL	9	578.1	DNA-directed RNA polymerase II subunit RPB1	P08775
SQVLSGCEI	9	578.2	Dolichyl-diphosphooligosaccharide--protein glycosyltransferase subunit 2	Q9DBG6
LAYENVKEV	9	580.6	Cytoplasmic dynein 1 heavy chain 1	Q9JHU4
SSPKSTLTL	9	581.2	Tensin-3	Q5SSZ5; Q5SSZ5-2
VSLEKCDYI	9	582.2	Thioredoxin reductase 3	Q99MD6
NAVLNQRYLEA	12	582.6	Coiled-coil domain-containing protein 125	Q5U465-2; Q5U465
SSLKNHKGIHM	11	583	zinc finger protein 62	Q8C827-3; Q8C827-2; Q8C827
FEHENKFYI	9	584.2	Vacuolar protein sorting-associated protein 29	Q9QZ88; Q9QZ88-2
SAPRNFVEN	9	584.7	elongator complex protein 2	Q91WG4-2; Q91WG4
WVLVNEKPL	9	585.5	Gamma-interferon-inducible lysosomal thiol reductase	Q9ESY9
SALGNVTTC	9	587	Eukaryotic translation initiation factor 6	O55135
SAPYGRITL	9	587.4	Cytoplasmic FMR1-interacting protein 1	Q7TMB8-2; Q7TMB8; Q5SQX6
RSVTHFDSL	9	587.7	Long-chain-fatty-acid--CoA ligase 4	Q9QUJ7-1; Q9QUJ7-2
LSPNNHEVHI	10	587.8	Actin-related protein 2/3 complex subunit 1A	Q9R0Q6
SCGINLVTL	9	588.5	Lysosome-associated membrane glycoprotein 1	P11438
RQWLNSGHI	9	590	Protein phosphatase 1 regulatory subunit 12A	Q9DBR7-1; Q9DBR7-2
TSPINPQHMTM	11	590.9	Trinucleotide repeat-containing gene 6C protein	Q3UHC0
ACFFNQEVL	9	592.9	Xaa-Pro dipeptidase	Q11136
FQLVSIEKL	9	595.3	endoplasmic reticulum aminopeptidase 1	Q9EQH2
TCLENSSCI	9	595.3	Short transient receptor potential channel 4-associated protein	Q9JLV2-1
RSFANDDRHV	11	597.8	spermatid perinuclear RNA-binding protein	Q91WM1; Q91WM1-2
ISAASITPL	9	600.6	Cell division cycle and apoptosis regulator protein 1	Q8CH18-2; Q8CH18; Q8CH18-3
KMWENRQNL	9	602.1	Spectrin beta chain, non-erythrocytic 1	Q62261-1; Q62261-2
SACWNSSHL	9	602.4	probable cation-transporting ATPase 13A3	Q5XF89-1
WMRENAEYL	9	603	Transcription factor IIIB 90 kDa subunit	Q8CFK2
SAPTNTGSTGI	11	604.1	Nuclear pore complex protein Nup54	Q8BTS4
SQLPLLSTI	9	607	WD repeat-containing protein 76	A6PWY4-1; A6PWY4-2
MAPQNVDRA	9	607.7	Cell division cycle-associated protein 4	Q9CWM2
HSLENSLIDIM	11	609.7	Cytoplasmic polyadenylation element-binding protein 2	Q812E0
GVHVNQELL	9	612.1	Vacuolar protein sorting-associated protein 8 homolog	Q0P5W1-2; Q0P5W1; Q0P5W1-3
CQYLNKNALTT	12	613.5	Negative elongation factor A	Q8BG30
SCPANLPNI	9	613.6	Microphthalmia-associated transcription factor	Q08874-7; Q08874; Q08874-9; Q08874-6; Q08874-2; Q08874-3; Q08874-4; Q08874-5; Q08874-8
ALLNQQFL	8	614.3	Serine/threonine-protein phosphatase 2B catalytic subunit gamma isoform	P48455; P48453-2; P48453
CSQSNYQHI	9	614.4	AP-3 complex subunit delta-1	O54774
SAVVNKVPLSC	11	615.3	Transmembrane protein 263	Q9DAM7
GLLQNLQHL	9	615.6	volume-regulated anion channel subunit LRRC8D	Q8BGR2
IGITNHDEYSL	11	616.5	Talin-1	P26039
YMYKSKELI	9	616.6	Ubiquitin carboxyl-terminal hydrolase 28	Q5I043; Q5I043-2
NSNLNRERM	9	618.2	PHD finger protein 10	Q9D8M7-2; Q9D8M7-1
NSIIMLEAL	9	618.6	Small nuclear ribonucleoprotein G	P62309
FCAVNPKFM	9	618.7	Coronin-1A	O89053
RALDNDQHL	9	620.8	E3 ubiquitin-protein ligase RNF213	E9Q555
TGPENSNTDTI	11	621.1	MAX gene-associated protein	A2AWL7-3; A2AWL7

HAITNSGQF	9	621.7	Nck-associated protein 1-like	Q8K1X4
SQHQSSYGPM	10	622.5	Heterogeneous nuclear ribonucleoprotein A0	Q9CX86
SQTRNHIQTL	10	622.7	Chromosome transmission fidelity protein 18 homolog	Q8BIW9
SGLSGHTTL	9	623	Microtubule-associated protein 4	P27546-1; P27546-2; P27546-3; P27546-4
KQMLLFTHI	9	623.8	E3 ubiquitin-protein ligase HUWE1	Q7TMY8-4; Q7TMY8-3; Q7TMY8; Q7TMY8-2
NAIENSSSKV	10	626	Centrosomal protein of 162 kDa	Q6ZQ06
SVGLNSTAL	9	628.8	Serine beta-lactamase-like protein LACTB, mitochondrial	Q9EP89
ASLTNKHSQEI	11	631.2	Spindle assembly abnormal protein 6 homolog	Q80UK7; Q80UK7-2
NQHYNTSLL	9	634.7	E3 SUMO-protein ligase PIAS1	O88907
NCFKNRYTL	9	635.4	Cullin-1	Q9WTX6
NSIKNHWNSTI	11	647.5	Myb-related protein A	P51960-2; P51960
CQVENGRVI	9	648.2	Muscleblind-like protein 2	Q8C181; Q8C181-3; Q9JKP5; Q8C181-2; Q8C181-4
YAVTGDVKM	9	649.8	Nuclear factor NF-kappa-B p105 subunit	P25799-7; P25799-5; P25799-3; P25799-2; P25799-6; P25799
AQLAKKDYL	9	650.9	Neutrophil cytosol factor 2	O70145
RSVIGSEFI	9	651.1	Dipeptidase 2	Q8C255; Q8C255-3
FGLCHHGNYL	10	654.5	Prolow-density lipoprotein receptor-related protein 1	Q91ZX7
SSILNKYDDNV	11	654.7	AH receptor-interacting protein	O08915
TQLLMTEVM	9	656.1	caspase recruitment domain-containing protein 9	A2AIV8
VMAQNPYNAV	11	656.1	WD repeat-containing protein 46	Q9Z0H1
INIMNHEKV	9	658.1	Uncharacterized protein C8orf59 homolog	Q0VG62
TVFKNVDLL	9	659.7	Nucleosome assembly protein 1-like 1	P28656
YSLGSLEHL	9	660.9	Toll-like receptor 2	Q9QUN7
TSLVDADTF	9	661.2	Emerin	O08579
RAPEGDDFL	9	661.4	Splicing factor, arginine/serine-rich 19	Q5U4C3
TGLLNDSTF	9	661.7	D-3-phosphoglycerate dehydrogenase	Q61753
VTIRLLDVL	9	662.1	Ataxin-10	P28658
RTVGNIEEL	9	662.2	Dehydrogenase/reductase SDR family member 11	Q3U0B3
FHHSNIEML	9	663.8	Disintegrin and metalloproteinase domain-containing protein 17	Q9Z0F8
FTLVNKKNALI	11	667.3	EH domain-binding protein 1-like protein 1	Q99MS7-3; Q99MS7; Q99MS7-4; Q99MS7-5; Q99MS7-2
TCVSNITDI	9	667.9	Protein aurora borealis	Q8BS90-1
FQWPNSQDI	9	670.6	Putative Dol-P-Glc:Glc(2)Man(9)GlcNAc(2)-PP-Dol alpha-1,2-glucosyltransferase	Q3UGP8
RAPTGSTEL	9	672.9	Fanconi anemia core complex-associated protein 100	A2ACJ2
YSPTGKEFV	9	675.2	DDB1- and CUL4-associated factor 13	Q6PAC3
KTVTNAVVTV	10	678.2	Heat shock cognate 71 kDa protein	P63017
SAPLGREHL	9	679.8	lymphocyte cytosolic protein 2	Q60787
SQPRLVDII	9	682.8	elongator complex protein 3	Q9CZX0-2; Q9CZX0
HGITNLCVIG	10	684.6	ATP-dependent 6-phosphofructokinase, liver type	P12382
TATNNTSAIQD	13	687.6	Glutaredoxin-1	Q9QUH0
RQYPNPFQL	9	689.7	zinc finger protein 280c	Q6P3Y5-1; Q6P3Y5-2; Q6P3Y5-3
FMLALKENI	9	691.8	Protein diaphanous homolog 3	Q9Z207
YGYSNRVVDLI	13	692.9	Glyceraldehyde-3-phosphate dehydrogenase	P16858
GACLNPVCL	9	693.2	Mitogen-activated protein kinase kinase kinase 7	Q62073
ICPVNRDTIDYI	12	695.4	Diacylglycerol O-acyltransferase 2	Q9DCV3
SSPVCQEQL	9	695.9	Talin-2	Q71LX4; P26039
AAPFDTVHI	9	700.1	tRNA (uracil-5-)-methyltransferase homolog A	Q8BNV1
TLIENGEKI	9	700.1	nuclear pore glycoprotein p62	Q63850

VLGQNAQTM	9		700.3	cold shock domain-containing protein E1	Q91W50
RAIELLEKL	9		702.6	Protein lin-7 homolog A	O88952; Q8JZS0
RVVANGDTA	9		708.5	methylthioribose-1-phosphate isomerase	Q9CQT1
LSKINKDWL	9		710.4	Neutrophil cytosol factor 4	P97369
VAARNLMTSEI	11		712.6	Centromere/kinetochore protein zw10 homolog	O54692
SQLIILDTL	9		714.2	Neurofibromin	Q04690-3; Q04690; Q04690-4; Q04690-2
TAIKNTSFC	9		717	Protein FAM135A	Q6NS59
RGIVLLEEL	9		717.3	mitochondrial fission 1 protein	Q9CQ92
STTKNRFVV	9		717.7	Eukaryotic translation initiation factor 4E	P63073
QMARNIWYF	9		717.9	Isoform 3 of Fatty acyl-CoA reductase 1	Q922J9-3; Q922J9-4; Q922J9-1
FSQGPGNRPP	11		718.9	lymphocyte cytosolic protein 2	Q60787
FAIQNKHLC	9		722.6	Cyclic AMP-dependent transcription factor ATF-3	Q60765
KQLANEVLSVL	11		722.8	Nitric oxide synthase, inducible	P29477
SGVNRYYV	8		723.1	NADH dehydrogenase [ubiquinone] 1 alpha subcomplex subunit 1	O35683
YALDLKAFL	9		723.5	Radiation-inducible immediate-early gene IEX-1	P46694
TAGLNVGSL	9		723.8	CUGBP Elav-like family member 1	P28659-2; P28659-1; P28659-3; P28659-4
SSPKNVQGL	9		725.3	sialoadhesin	Q62230-3; Q62230-2; Q62230
KQLTNLVNVCL	11		726.2	Syndetin	Q8CI71
HQLQNEKCHL	10		728.4	STE20-like serine/threonine-protein kinase	O54988-1
VMQENITKV	9		728.6	Vesicle-associated membrane protein 4	O70480
YMWDKSCDYL	10	1xOxidation [M2]	730.5	Protein C8orf37 homolog	Q3UJP5
IQPDNIVYV	9		733.4	Signal recognition particle 54 kDa protein	P14576
GQLKNKYGDA	12		733.8	U6 snRNA-associated Sm-like protein LSm6	P62313
SMPRALVSPL	10		736.6	G2 and S phase-expressed protein 1	Q8R080
NAPVNPTRA	9		738.6	phorbol-12-myristate-13-acetate-induced protein 1	Q9JM54
SAVVNKVPLSC	12		739	Transmembrane protein 263	Q9DAM7
SAPLPYEAL	9		741.6	Phosphorylase b kinase regulatory subunit alpha, skeletal muscle isoform	P18826-2; P18826
STIRLLTSL	9		743.3	T-complex protein 1 subunit gamma	P80318
VAPKNYSETIE	13		743.3	Double-strand break repair protein MRE11	Q61216; Q61216-2
RGVPNHIHM	9		745	Protein CASC3	Q8K3W3
AQFYGLTPL	9		745.1	SH3KBP1-binding protein 1	Q6P7W2
KAVANQTSATI	11		747.9	26S proteasome regulatory subunit 4	P62192
QIQNAQYL	8		748	Heterogeneous nuclear ribonucleoprotein K	P61979-3; P61979-2; P61979
HCPLNEEVI	9		753.1	E3 ubiquitin-protein ligase UBR1	O70481
LSHKNPDQVYI	11		753.5	Serine/threonine-protein kinase VRK1	Q80X41-2; Q80X41-5; Q80X41-3; Q80X41
HVLSNCELL	9		755.1	PX domain-containing protein kinase-like protein	Q8BX57; Q8BX57-2; Q8BX57-3
ASCSNITRL	9		755.2	Toll-like receptor 9	Q9EQU3
INVPNAEVL	9		755.3	Probable ubiquitin carboxyl-terminal hydrolase FAF-X	P70398
VSVCNQIASHL	11		756.2	Choline/ethanolaminephosphotransferase 1	Q8BGS7
IHQVNLEYL	9		760.9	Cytoplasmic aconitate hydratase	P28271
HQMQLVDSI	9		762.1	Nucleoprotein TPR	F6ZDS4
HGAMNQQHM	9		763.4	Forkhead box protein J3	Q8BUR3-2; Q8BUR3
SSPTGGPTL	9		763.8	Collagen alpha-1(XV) chain	O35206
SAPTSPTRL	9		764.2	Mitochondrial dynamics protein MID51	Q8BGV8
KSLTNDWEDH	11		770.2	Heat shock protein HSP 90-beta	P11499
SAISSSDLF	9		771.2	ADP-ribosylation factor GTPase-activating protein 2	Q99K28-2; Q99K28
VCVDNSEYM	9		772.2	26S proteasome non-ATPase regulatory subunit 4	O35226-3; O35226; O35226-4; O35226-2; O35226-5
FQVTHTVAL	9		773	Mitochondrial import receptor subunit TOM40 homolog	Q9QYA2

GAPVNVSSSDI	11		774.2	Casein kinase I isoform delta	Q9DC28-2; Q9DC28-1
SLVNQEVL	8		777.1	DNA-directed RNA polymerase I subunit RPA2	P70700
QIIINTTHL	9		777.3	Exocyst complex component 6	Q8R313; A6H5Z3; A6H5Z3-2
LCVSNRIAI	9		778.8	Inositol 1,4,5-trisphosphate receptor type 3	P70227
FCPRLLDYL	9		779	MAP kinase-activating death domain protein	Q80U28-3; Q80U28-2; Q80U28-5; Q80U28-13; Q80U28-7; Q80U28-9; Q80U28-11; Q80U28; Q80U28-14; Q80U28-6; Q80U28-15; Q80U28-10; Q80U28-12; Q80U28-4; Q80U28-8
RCIQNMPKTL	10		782.1	Tumor necrosis factor ligand superfamily member 13B OS=Mus musculus OX=10090	Q9WU72
KAPFSVEFV	9		783.8	Mannosyl-oligosaccharide glucosidase	Q80UM7
VCLWNKDQI	9		784.4	Shugoshin 1	Q9CXH7
FVGENDRTTL	10		785.1	striated muscle-specific serine/threonine-protein kinase	Q62407-4; Q62407-3; Q62407-1
SSPRHKEWL	9		786.9	Vacuolar protein sorting-associated protein 18 homolog	Q8R307
TMETNREEL	9		789.6	patched domain-containing protein 1	Q14B62-2; Q14B62
AQGINVNLV	9		790.2	Ankyrin repeat and SOCS box protein 11	Q9CQ31; Q9CQ31-2
ISYVLPEHM	9		790.5	ATP-citrate synthase	Q91V92
CMYFKRFYL	9		790.6	Cyclin-H	Q61458
RGVKNRPEEF	11		792.7	sorting nexin-7	Q9CY18
AALKLGQEL	9		794.5	protein flightless-1 homolog	Q9JJ28
QKVKNTEYL	9		798.1	Sorting nexin-14	Q8BHY8
TSPINPYKDHM	11		798.1	zinc finger E-box-binding homeobox 2	Q9R0G7
TMMELRCVI	9	Carbamidomethyl [C	798.5	Inositol hexakisphosphate and diphosphoinositol-pentakisphosphate kinase 1	A2ARP1-2; A2ARP1-3; A2ARP1-1; Q6ZQB6; A2ARP1-7; Q6ZQB6-2; Q6ZQB6-3; A2ARP1-6
FSYKIDFAEM	10		800.4	Poly [ADP-ribose] polymerase 11	Q8CFF0-1; Q8CFF0-3
CQTHNLDHL	9		801.2	Protein NLRC5	C3VPR6
HALENLSSRHL	11		801.2	protein SLX4IP	Q9D7Y9-2; Q9D7Y9
AAVGNHVAKL	10		803.4	NEDD8-activating enzyme E1 regulatory subunit	Q8VBW6
QGLVNSTGM	9		805.6	Chromodomain-helicase-DNA-binding protein 7	A2AJK6-2; A2AJK6-3; A2AJK6-1
YCIENAHGQL	10		805.9	Protein TSSC1	Q8K0G5
ATCQNLQWL	9		806.5	Myomegalin	Q80YT7; Q80YT7-2
SQGENLEGKY	11		806.6	Pogo transposable element with ZNF domain	Q8BZH4
NSMVLFDHV	9		808.3	DNA topoisomerase 2-alpha	Q01320
SSVSNPRNWL	11		818.7	Tumor necrosis factor receptor superfamily member 23	Q9ER63
FSPSHETHL	9		821.5	Leucine-rich repeat and WD repeat-containing protein 1	Q8BUI3
RAVANETGAFI	11		824.2	Transitional endoplasmic reticulum ATPase	Q01853
TSFKGRTFM	9		827	Cleavage and polyadenylation specificity factor subunit 3	Q9QXK7
FVQTNISHL	9		831.9	Fms-related tyrosine kinase 3 ligand	P49772; P49772-4; P49772-2; P49772-3
KGSANQETL	9		832	Fanconi anemia group I protein homolog	Q8K368-1
HQQQNLDTF	9		833.6	E3 ubiquitin-protein ligase RNF213	E9Q555
LSALNPELV	9		837.2	Hepatocyte growth factor receptor	P16056
TQGQNIQHL	9		839.4	Myomegalin	Q80YT7; Q80YT7-2
KQARNSQFDF	11		845.8	Splicing factor, suppressor of white-apricot homolog	Q3USH5
VAPKNYSETIE	14		849.5	Double-strand break repair protein MRE11	Q61216; Q61216-2
RCIFNRSLL	9		851.5	Protein O-linked-mannose beta-1,4-N-acetylglucosaminyltransferase 2	Q8BW41; Q8BW41-2
SCLVMTTEI	9		854.1	Exosome RNA helicase MTR4	Q9CZU3
NQFVNKFNV	9		859.8	COP9 signalosome complex subunit 6	O88545
SSVRNVSTC	9		862	Homeodomain-interacting protein kinase 2	Q9QZR5
AAIIDHERI	9		864.4	Serine/threonine-protein kinase MRCK alpha	Q3UU96-2; Q3UU96-1

HSVSNDKAL	9		866	Cyclic AMP-dependent transcription factor ATF-6 alpha	F6VAN0
GAIRNISGTLP	11		868.6	CSC1-like protein 1	Q91YT8
VAPENLPALLL	11		869.7	ATP-binding cassette sub-family A member 7	Q91V24
FCAVNPKFL	9		876.4	Coronin-6	Q920M5-1; Q9WUM3
NQLSNHISSL	10		876.6	Glucose-6-phosphate 1-dehydrogenase X	P97324; Q00612
FSPRAVDYV	9		876.9	Isoform 2 of Methyltransferase-like protein 6	Q8BVH9-2; Q8BVH9-1
SQLIDTHLL	9		878.8	Protein Prrc2c	Q3TLH4; Q3TLH4-5
QGPGNRPPL	9		892.7	lymphocyte cytosolic protein 2	Q60787
STLVHKFFL	9		892.7	Isoform 2 of Poly(A) polymerase alpha	Q61183-2; Q61183-3; Q6PCL9; Q61183-1; Q61183-4
QSDINFDVL	9		897	ATP-binding cassette sub-family A member 5	Q8K448
GCISNTASYL	10		902.4	V-type proton ATPase 116 kDa subunit a isoform 1	Q9Z1G4-2; Q9Z1G4-3; Q9Z1G4; P15920; P15920-2; Q920R6
KCLENLVQL	9		906.2	Rotatin	Q8R4Y8
FQYESKVFYL	10		908.3	14-3-3 protein eta	P68510
IAPEDQVVL	9		910.2	Ubiquitin-like protein FUBI	P35545
QTVENVEHL	9		911.3	TELO2-interacting protein 1 homolog	Q91V83
KIHRNYSFLDY	12		911.7	Copine-2	P59108
SAMELIWNL	9	1xOxidation [M3]	912.1	Nuclear pore complex protein Nup85	Q8R480
FAGHSGDVM	9		913.5	Guanine nucleotide-binding protein G(I)/G(S)/G(T) subunit beta-2	P62880
GGVSNVTEA	9		915.3	DNA replication ATP-dependent helicase/nuclease DNA2	Q6ZQJ5; Q6ZQJ5-2
RAINNEMAHQI	11		917.7	Engulfment and cell motility protein 1	Q8BPU7-3; Q8BPU7-1
TGPENPWLV	9		920.8	Endoplasmic reticulum metallopeptidase 1	Q3UVK0
SRFKNRETI	9		929.2	DNA-directed RNA polymerase II subunit RPB4	Q9D7M8
RSLESRDHL	9		929.7	Coiled-coil domain-containing protein 88B	Q4QRL3; Q4QRL3-2
STLKPNVHHL	10		938.1	Methylcytosine dioxygenase TET2	Q4JK59; Q4JK59-3; Q4JK59-2
RAEDNADTL	9		939.8	proliferating cell nuclear antigen	P17918
RCQSNLDTL	9		941.1	Tfiih basal transcription factor complex helicase xpd subunit	O08811
SVVANGVCHL	10	Carbamidomethyl [C	942.2	Monocarboxylate transporter 1	P53986
SGPSNLGKFTI	14		953.7	Serine-rich coiled-coil domain-containing protein 2	Q3UHI0-2; Q3UHI0
RTPENHENL	9		955.4	Lysine-specific demethylase 3B	Q6ZPY7; Q6ZPY7-2
VAPTNPRFGG	13		957.1	DNA replication licensing factor MCM6	P97311
LCALNVVYM	9		959.7	Tetraspanin-31 OS=Mus musculus OX=10090	Q9CQ88
SMVKKWQTM	9		965.1	40S ribosomal protein S3a	P97351
IAIVNHDKC	9		967.1	ATP-binding cassette sub-family E member 1	P61222
GSPENLGESTI	11		971	Nuclear receptor coactivator 7	Q6DFV7-1
SCFQNQRAI	9		974.5	ated matrix-associated actin-dependent regulator of chromatin subfamily A containing DE	Q04692
SGPKDVTVL	9		978.8	Zinc finger protein 638	Q61464-4; Q61464
SQMEHAMETM	10		983.2	Protein S100-A10	P08207
FAGRSFRNPL	10		992.7	Alpha-enolase	P17182
HSHLLKTPL	9		993.9	Ribonucleoprotein PTB-binding 1	Q9CW46
RGLDNREEV	9		996.2	Pseudopodium-enriched atypical kinase 1	Q69Z38
EAIHNFVEVL	10		997.5	AP-2 complex subunit sigma	P62743
WCPRNPAVL	9		999	Protein transport protein Sec31A	Q3UPL0
SACHNDSVFL	10		1001	Protein CIP2A	Q8BWY9
SAMRNLNGRE	11		1001.6	cleavage stimulation factor subunit 2	Q8BIQ5-2; Q8BIQ5-1; Q8C7E9

YQLLLKELL	9	1002.2	Triple functional domain protein	A2CG49-9; A2CG49-2; Q0KL02-4; A2CG49; A2CG49-7; A2CG49-5; A2CG49-10; A2CG49-8; Q0KL02; Q0KL02-3; Q0KL02-2
AAISLATRV	9	1003.6	Putative ATP-dependent RNA helicase DHX33	Q80VY9
VSVGNVGQL	9	1003.7	Proteasome assembly chaperone 2	Q9EST4-2; Q9EST4
TIYQNAGPTM	10	1003.9	signal transducing adapter molecule 1	P70297
RQYRLTEHM	9	1008	hypermethylated in cancer 2 protein	Q9JLZ6; Q9R1Y5-1
KALALKEAL	9	1008.1	Chromosome-associated kinesin KIF4	P33174
SSTTNPKLSTL	11	1009.6	Eukaryotic translation initiation factor 4E transporter	Q9EST3; Q9EST3-2
STLVLHDLL	9	1014.1	Breakpoint cluster region protein	Q6PAJ1; Q5SSL4; Q5SSL4-2; Q5SSL4-4; Q5SSL4-3
RGVEGSDYI	9	1017.5	Receptor-type tyrosine-protein phosphatase F	Q64487-5; Q64487-9; B0V2N1-2; Q64487-3; Q64487-11; Q64487-6; Q64487-2; Q64487-7; Q64487-8; A2A8L5; B0V2N1-5; B0V2N1; Q64487-12; Q64487-10; B0V2N1-3; B0V2N1-6; B0V2N1-4; Q64487-1; Q64487
TTFQNDFESL	10	1019.3	Choline kinase alpha	O54804-2; O54804
VQHASVQYI	9	1037.9	Lysosomal alpha-mannosidase	O09159
VRVTNRDII	9	1039.3	60S ribosomal protein L5	P47962
RTLSNSEENPL	11	1045.8	Rotatin	Q8R4Y8
CSLENREECLM	11	1051.8	Ribonucleoside-diphosphate reductase large subunit	P07742
RSITNTTVCT	10	1053.6	Splicing factor 1	Q64213
LQVQNPQAQT	11	1055.2	Transcription factor Sp1	O89090-2; O89090
RSLRLREMM	9	1061.8	E3 ubiquitin-protein ligase UBR5	Q80TP3
SAVKNMNLP	9	1062.4	AP-3 complex subunit sigma-1	Q9DCR2
NALSNPKLLQM	11	1084.1	Dedicator of cytokinesis protein 10	Q8BZN6-3; Q8BZN6-4; Q8BZN6-1; Q8BZN6-2
QGLRNSVRM	9	1086.3	Sperm-specific antigen 2 homolog	Q922B9-1; Q922B9-3
KAPANRELDCI	11	1089.7	BCLAF1 and THRAP3 family member 3 OS=Mus musculus OX=10090	A2AG58
FNKLNLEHI	9	1092.2	Vacuolar protein sorting-associated protein 35	Q9EQH3
TQCLNESTYI	10	1103.3	Helicase with zinc finger domain 2	E9QAM5
ASPESLHYHL	10	1103.5	Carbohydrate sulfotransferase 14	Q80V53
CTVMNIAEHYM	11	1108.6	Eukaryotic translation initiation factor 3 subunit B	Q8JZQ9
QGITNLKLVTL	11	1110.8	Kinetochore-associated protein 1	Q8C3Y4
SAVKLRWLL	9	1111.5	Isoform 1 of Glycerol kinase	Q64516-2; Q64516-1; Q64516
YQYALEHMI	9	1111.5	Kinetochore-associated protein 1	Q8C3Y4
RTVYNREKL	9	1111.7	metal transporter CNNM4	Q69ZF7
KQTINPILL	9	1114.3	long-chain-fatty-acid--CoA ligase 3	Q9CZW4
SCLLNTKVL	9	1121.7	BAH and coiled-coil domain-containing protein 1	Q3UHR0
AALENDKTIKL	11	1129.6	WD repeat-containing protein 5	P61965
SCLYNPDDL	9	1129.7	Proto-oncogene c-Rel	P15307
AAITNKYQLVF	12	1130.6	RNA polymerase I-specific transcription initiation factor RRN3	B2RS91
SQLSDWQHL	9	1132.6	SUN domain-containing protein 1	Q9D666; Q9D666-3; Q9D666-4; Q9D666-2; Q9D666-5
LQYPNPLPTL	10	1136.3	Vam6/Vps39-like protein	Q8R5L3-2; Q8R5L3-1
QAVWNVDYC	9	1137.1	Cyclin-Y	Q8BGU5; Q8BGU5-2
DGVANVEHI	9	1138.8	Cytosolic 5'-nucleotidase 3A	Q9D020; Q9D020-1
VPPVNGVEI	9	1144.1	Protein PRRC2B	Q7TPM1-2; Q7TPM1-1
VAPGMMKEFM	10	1144.2	Brefeldin A-inhibited guanine nucleotide-exchange protein 3	Q3UGY8

AAPAPSLWTL	10	1149.9	Protein shisa-5	Q9D7I0-1; Q9D7I0-2
SIHGTNPQYL	10	1153.1	Pre-mRNA-splicing factor 38A	Q4FK66-1
SVAVNISNL	9	1158.4	Deubiquitinase OTUD6B	Q8K2H2
NSGLNFKDVL	10	1160.6	AT-rich interactive domain-containing protein 5B	Q8BM75; Q8BM75-3
RAIKNDSVVA	10	1162.7	T-complex protein 1 subunit eta	P80313
FQVAHTVHM	9	1163.3	Mitochondrial import receptor subunit TOM40B	Q9CZR3
CMWRNYEYC	9	1168.1	SNF-related matrix-associated actin-dependent regulator of chromatin subfamily A meml	Q91ZW3
ACGANREEI	9	1173.1	TBC1 domain family member 8B	A3KGB4
LQVSNVLSQPI	11	1175.9	Dolichyl-diphosphooligosaccharide--protein glycosyltransferase subunit 2	Q9DBG6
FQGTSLTHL	9	1177.6	Transforming growth factor beta activator LRRC33	Q8BMT4; Q8BMT4-2; Q8BMT4-3
RSLQGKTEL	9	1177.7	Dihydroorotate dehydrogenase (Quinone), mitochondrial	O35435
ACIVNTENKI	10	1183.9	Deoxycytidylate deaminase OS=Mus musculus OX=10090	Q8K2D6
IAPRSFWMTL	10	1186.1	nuclear pore complex protein Nup85	Q8R480
RCIANPVKL	9	1191.3	Isoaspartyl peptidase/L-asparaginase	Q8C0M9
GSLLNQPKAVI	11	1203.9	Claspin	Q80YR7
GMHVNGAPP	9	1204.6	cleavage stimulation factor subunit 2	Q8BIQ5-2; Q8BIQ5-1
SQGSNFLSAL	10	1212.7	Palmitoyltransferase ZDHHC18	Q5Y5T2
LATVNHRSII	10	1213.1	Testis-specific serine/threonine-protein kinase 2 OS=Mus musculus OX=10090	O54863
SGLLCVDKI	9	1214.1	thyroid hormone receptor alpha	P63058-1
YAAAALPTL	9	1216.9	CUGBP Elav-like family member 2	Q9Z0H4-1; P28659-2; Q9Z0H4-7; Q9Z0H4-11; Q9Z0H4-9; P28659-1; Q9Z0H4-4; Q9Z0H4-8; P28659-3; P28659-4; Q9Z0H4-6; Q9Z0H4-2; Q9Z0H4-3; Q9Z0H4-5
THLQNQEKL	9	1218.3	Pericentrin	P48725-1; P48725-3; P48725-2
RVYENVGLM	9	1223.1	tyrosine-protein phosphatase non-receptor type 11	P35235; P35235-2
SSLLDSAHL	9	1223.8	AP-3 complex subunit delta-1	O54774
RGLRNLINMEN	11	1223.8	schlafen family member 5	Q8CBA2
RQLLLCETL	9	1236	Rho guanine nucleotide exchange factor 10-like protein	A2AWP8-1
YALDLIDKL	9	1244.3	Cyclin-dependent kinase 9	Q99J95-3; Q99J95; Q99J95-2
FCISNPVLA	9	1246	Cerebral dopamine neurotrophic factor OS=Mus musculus OX=10090	Q8CC36
VQVTGGQYL	9	1246.9	ATP-dependent DNA helicase PIF1	Q80SX8; Q80SX8-3
WMNINLREV	9	1248.4	U6 snRNA-associated Sm-like protein LSm4	Q9QXA5
SIVFNGPHL	9	1253.8	Histone-lysine N-methyltransferase SMYD3	Q9CWR2
FIHNNLKDVYI	11	1256.9	SH3 and PX domain-containing protein 2A	O89032-1; O89032-3; O89032-2
FQVENSNRLLL	11	1259.8	Murinoglobulin-1	P28665
SAPLSPLKFM	10	1263.5	Gamma-adducin	Q9QYB5-2; Q9QYB5-1
SAIQDYLQQL	10	1265.1	Glutaredoxin-1	Q9QUH0
FGLVNQERT	9	1266.3	NACHT, LRR and PYD domains-containing protein 3	Q8R4B8-4; Q8R4B8-2; Q8R4B8-3; Q8R4B8-1
FAHTNIESLVKI	12	1267.5	Protein disulfide-isomerase A3	P27773
KALQFLEQV	9	1269.3	T-complex protein 1 subunit zeta	P80317
CSPQNKISF	9	1271.1	Signal peptide peptidase-like 3	Q9CUS9
RAGPRLIVYI	10	1273.4	Syntaxin-binding protein 2	Q64324
AGIENKFGL	9	1279.2	Calcium-binding mitochondrial carrier protein Aralar2	Q8BH59; Q9QXX4
SAILDHPPSPM	11	1280.6	MKL/myocardin-like protein 1	Q8K4J6-2; Q8K4J6-1
SAPTDNNLCHI	11	1284.1	Pyrroline-5-carboxylate reductase 3	Q9DCC4
FCERNRDVL	9	1290	Unconventional myosin-Ie	E9Q634; P70248
MMGYNTDRL	9	1295	anaphase-promoting complex subunit 1	P53995
SAQRNHAIRI	10	1303	cold shock domain-containing protein E1	Q91W50

SLGLNPYPGI	10		1303.5	Macrophage colony-stimulating factor 1 receptor	P09581
GTIVNGKQI	9		1305	angiogenic factor with G patch and FHA domains 1	Q7TN31
QALRDNSTM	9		1314.4	Heat shock protein HSP 90-alpha	P07901; P11499
RALTNHTVYC	10		1315.8	L-xylulose reductase	Q91X52
AAAKNLSDMTI	11	1xOxidation [M9]	1317.5	T-complex protein 11-like protein 2	Q8K1H7
VTCSNVEVL	9		1323	Leucine-rich repeat serine/threonine-protein kinase 2	Q5S006
RQPQNQQKV	9		1325.2	cationic amino acid transporter 2	P18581-2; P18581-1
SSPRSDVPVM	10		1326.5	U3 small nucleolar RNA-associated protein 14 homolog A	Q640M1
RTVKNIADL	9		1328.3	Neuron navigator 3	Q80TN7
SMATNTGGLS	12		1333	Nuclear receptor coactivator 2	Q61026
FCFDNTFSTI	10		1340.8	Transmembrane emp24 domain-containing protein 5	Q9CXE7
SALTGGTAHL	10		1344.3	neuroguidin	Q9DB96
ICLGNTYHL	9		1347.7	Queuine tRNA-ribosyltransferase catalytic subunit 1 OS=Mus musculus OX=10090	Q9JMA2
RSLLNSHILHL	11		1350.3	WD repeat and FYVE domain-containing protein 3	Q6VNB8
IGGENGPVL	9		1352.3	U5 small nuclear ribonucleoprotein 200 kDa helicase	Q6P4T2
FQCLNHRCI	9		1352.7	Low-density lipoprotein receptor-related protein 10	Q7TQH7
ACLVNLRSL	9		1353.1	DNA polymerase delta subunit 2	O35654
SQLISLQHL	9		1355	Probable ATP-dependent RNA helicase DDX28	Q9CWT6
VQVEDIVFL	9		1358.4	Transcription initiation factor TFIID subunit 13	P61216
TILTNHQHL	9		1361.6	Protection of telomeres protein 1	Q91WC1-2; Q91WC1
MPWNVDTL	8		1362.5	Hsp90 co-chaperone Cdc37	Q61081
SQLEKLDLL	9		1363.8	Centrosomal protein of 57 kDa	Q8CEE0; Q8CEE0-3; Q8CEE0-2
VQEFNVTHL	9		1365.3	tRNA pseudouridine(38/39) synthase	Q9JI38
TCIVNESML	9		1378.2	probable cation-transporting ATPase 13A3	Q5XF89-1
KCIQNEAGDW	11		1379.9	SP110 nuclear body protein	Q8BVK9
GCLTNGHIYV	10		1380.3	N-acylneuraminate cytidylyltransferase	Q99KK2-2; Q99KK2
SRVTNGELL	9		1385	Endophilin-B2	Q8R3V5-3; Q8R3V5-1; Q8R3V5-4
VAISPGKHYL	10		1386.8	poly [ADP-ribose] polymerase 14	Q2EMV9
SFINNSIVYL	10		1390.3	Uromodulin-like 1	Q5DID3-2; Q5DID3-3; Q5DID3
FTHQNGERV	9		1390.5	Cytoplasmic polyadenylation element-binding protein 4	Q7TN98-5; Q7TN98-1; Q7TN98-2; Q7TN98-3; Q7TN98-4
IGPFNNIDI	9		1393.8	Structural maintenance of chromosomes flexible hinge domain-containing protein 1	Q6P5D8
FGIRDDMVL	9		1394.4	Leucine--tRNA ligase, cytoplasmic	Q8BMJ2
SGLRLTDTF	9		1396.9	Trafficking protein particle complex subunit 13	Q3TIR1-2; Q3TIR1; Q3TIR1-3
YNISLKEVM	9		1403.7	Translation initiation factor eIF-2B subunit epsilon	Q8CHW4
KVAVNGVHL	9		1409.1	Valacyclovir hydrolase	Q8R164
SVLRLVDAL	9		1412.7	histone deacetylase 6	Q9Z2V5
YQLKLAEKL	9		1416.7	Nck-associated protein 1-like	Q8K1X4
STLRNIQGL	9		1429.5	Proteasome maturation protein	Q9CQT5
GIENIHYL	8		1431.2	malignant T-cell-amplified sequence 2	Q9DB27-2; Q9CQ21; Q9DB27
SLGINPHVL	9		1433.6	nuclear pore complex protein Nup98-Nup96	Q6PFD9
FCPTNCHVNL	10		1439.4	Phosphatidylinositol 3,4,5-trisphosphate-dependent Rac exchanger 1 protein	Q69ZK0
FMVEKGPTL	9		1447	Ubiquitin-conjugating enzyme E2 J2	Q6P073
SSVKNEEQFVI	11		1448	Maternal embryonic leucine zipper kinase	Q61846
ASHLNLAMCHI	11		1451.8	Peptidyl-prolyl cis-trans isomerase FKBP4	P30416
VAPAAHTHI	9		1459.3	Arginine-glutamic acid dipeptide repeats protein	Q80TZ9
CVGENFAYV	9		1464.6	Lanosterol 14-alpha demethylase	Q8K0C4
NALLLNSVM	9		1467.3	VPS35 endosomal protein sorting factor-like	Q8BWQ6; Q8BWQ6-3

SGCKNGQVL	9	1467.9	Notchless protein homolog 1	Q8VEJ4
RHLENVMHI	9	1470.7	Protocadherin Fat 3	Q8BNA6
SNVKNEEKL	9	1474.3	SLAIN motif-containing protein 2	Q8CI08-2; Q8CI08-1
VQVPNLESLTL	11	1478.2	Chromodomain-helicase-DNA-binding protein 8	Q09XV5
AGIENKFGLYL	12	1480.3	Calcium-binding mitochondrial carrier protein Aralar2	Q8BH59; Q9QXX4
FIVTFTTFL	9	1481.5	autophagy-related protein 9B	Q6EBV9
FQVQDKEVL	9	1487.6	Rab3 GTPase-activating protein non-catalytic subunit	Q8BMG7-2; Q8BMG7
AGVRNPQQHL	10	1499.5	Polyadenylate-binding protein 1	P29341
AMVQHPQPTP	11	1505.1	Nuclear receptor coactivator 3	O09000
NCLENVVIL	9	1508.7	Brefeldin A-inhibited guanine nucleotide-exchange protein 1	G3X9K3
VGVVNPILA	9	1511.9	Poly(U)-binding-splicing factor PUF60	Q3UEB3-2; Q3UEB3; Q3UEB3-3
SVLVNFGQHV	10	1512.3	Vacuolar protein sorting-associated protein 13B	Q80TY5
YQLLLKEML	9	1527.5	Guanine nucleotide exchange factor DBS	Q64096
SCLKMLDEM	9	1533	COP9 signalosome complex subunit 1	Q99LD4
DALSLRDTL	9	1536.2	Cytoplasmic dynein 1 light intermediate chain 1	Q8R1Q8
SVIRLIFEI	9	1548.8	Putative pre-mRNA-splicing factor ATP-dependent RNA helicase DHX32	Q8BZS9-2; Q8BZS9
NSLNNNFLQTL	11	1561.5	Cullin-3	Q9JLV5
FSCPPHFTEM	10	1563.5	DNA-directed RNA polymerase I subunit RPA34	Q76KJ5
SQPNNLADVG	13	1568.7	Arf-GAP domain and FG repeat-containing protein 2	Q80WC7
SSPSNKFFFH	10	1572	Histone-lysine N-methyltransferase SETD2	E9Q5F9-2; E9Q5F9
KGPLSQAFL	9	1573.3	poly [ADP-ribose] polymerase 14	Q2EMV9; Q2EMV9-2
FCFSNEFSTF	10	1584.2	Transmembrane emp24 domain-containing protein 3	Q78IS1
FMMEVKDPNM	10	1588.7	E3 ubiquitin-protein ligase RBBP6	P97868-1; P97868-2
GCLENLENL	9	1597.6	CD180 antigen	Q62192
KVAVNGQHM	9	1601	Galectin-9	O08573-2; O08573; O08573-3
TIVGANLTVL	10	1601.4	Trophoblast glycoprotein-like	Q8C013
RSYDKSADPM	10	1607	Tyrosine-protein phosphatase non-receptor type 12	P35831
KSLLGKDVL	9	1608	phosphoglycerate kinase 1	P09411
RLNLNNTVL	9	1613.3	Bifunctional glutamate/proline--tRNA ligase	Q8CGC7
SSGFNPEGIYV	11	1614.5	Ras association domain-containing protein 8	Q8CJ96
YAPPSQSGHF	11	1616.6	Serine incorporator 3	Q9QZI9
SSLMKVENM	9	1618.5	Coiled-coil domain-containing protein 25	Q78PG9
QCLLNVEHL	9	1619.3	staphylococcal nuclease domain-containing protein 1	Q78PY7
SVLSLQEEI	9	1625	DNA mismatch repair protein Mlh1	Q9JK91
SCVSNIESAL	10	1631	copper-transporting ATPase 1	Q64430
SQVELREQI	9	1638.6	SNARE-associated protein Snapin	Q9Z266
TVFAYGTYADY	12	1640.4	COP9 signalosome complex subunit 7a	Q9CZ04-2; Q9CZ04
KALINPANVTF	11	1645	Acyl-protein thioesterase 1	P97823; P97823-2
SCVVQLDYL	9	1646.1	Hermansky-Pudlak syndrome 5 protein homolog	P59438; P59438-2; P59438-3
RALLLLERL	9	1648.1	telomere length regulation protein TEL2 homolog	Q9DC40-1
THLLNSEHL	9	1657.5	PHD finger protein 12	Q5SPL2-2; Q5SPL2-1
NAPRLIRPYM	10	1663.8	Serine/threonine-protein kinase mTOR	Q9JLN9
RGPENPKLEM	10	1671	probable ATP-dependent RNA helicase DHX58	Q99J87
FGGANHGGGY	11	1675.8	Interleukin enhancer-binding factor 3	Q9Z1X4-2; Q9Z1X4; Q9Z1X4-3
RMPEKVTWM	9	1676.7	60S ribosomal protein L19	P84099
TSVRNKPDSTL	11	1678.3	protein TALPID3	E9PV87
FHFTNRDCDSI	11	1683.1	Prostate tumor-overexpressed gene 1 protein homolog	Q91VU8
IKNENQEVI	9	1684.2	cGMP-dependent 3',5'-cyclic phosphodiesterase	Q922S4; Q922S4

VSGISRDVI	9	1694	poly [ADP-ribose] polymerase 14	Q2EMV9; Q2EMV9-2
SAVHNDQFGL	13	1696.6	Uncharacterized protein C15orf61 homolog	Q0VG49-2; Q0VG49
SAVISLEGKPL	11	1699.1	Cofilin-1	P18760
TCLENGSFLL	10	1709.5	Protein Churchill	Q6DG52-2; Q6DG52
AIRNDEEL	8	1710.1	Histone H2A type 3	Q64523; Q8BFU2; C0HKE4; Q8CGP5; Q8R1M2; P27661; Q8CGP7; Q8CGP6; Q6GSS7
SAYNNLKGNL(	13	1718.5	V-type proton ATPase subunit C 1	Q9Z1G3
FSPQMCEHL	9	1720.9	V-type proton ATPase subunit H	Q8BVE3
SGPTSLFAV	9	1721.3	Heterogeneous nuclear ribonucleoprotein U	Q8VEK3-2; Q8VEK3
LKVTNQELL	9	1723.5	Transmembrane protein 214	Q8BM55; Q8BM55-2; Q8BM55-3
GCINNPVAL	9	1727.7	Lysine-specific histone demethylase 1B	Q8CIG3-2; Q8CIG3
SAAADLAPF	9	1732.8	Homeobox protein Hox-A10	P31310
KLIQLMEEI	9	1733.4	Probable ATP-dependent RNA helicase DDX17	Q501J6; Q501J6-2
SFLKLYTTM	9	1741.2	eukaryotic translation initiation factor 3 subunit L	Q8QZY1
SAPFPAPDTI	10	1745.6	Band 4.1-like protein 5	Q8BGS1; Q8BGS1-3
RSLGNDVKSYI	11	1746.6	Protein Njmu-R1	Q9CYI0
SALRSLSNATI	11	1752	signal recognition particle 54 kDa protein	P14576-2; P14576-1
FAPPHFPVCM	10	1764.8	Solute carrier family 12 member 6	Q924N4
RALDNRRIGQI	12	1766.5	Phosphatidylinositol 4,5-bisphosphate 3-kinase catalytic subunit beta isoform	Q8BTI9
SALILHQRI	9	1766.7	Zinc finger protein 24	Q91VN1; Q8BLB0
KSISNPPGSNL	11	1771.4	Serine/threonine-protein kinase WNK1	P83741-2; P83741; P83741-3; P83741-5; P83741-4
HCLSLRTYL	9	1775.6	Bifunctional glutamate/proline--tRNA ligase	Q8CGC7
KRVVNQEEL	9	1780.5	zinc finger protein 830	Q8R1N0
ASPSSEMVTL	10	1780.6	Protein Daple	Q6VGS5; Q6VGS5-2
SQLEKTFAM	9	1781.6	Hermansky-Pudlak syndrome 5 protein homolog	P59438; P59438-2
SQGPTLQFTL	10	1782.2	Polycomb protein suz12	Q80U70
KAVENGEEHTI	11	1792.2	ankyrin repeat and LEM domain-containing protein 2	Q6P1H6-1; Q6P1H6-4; Q6P1H6-3; Q6P1H6-2
GVMLNTERL	9	1794.2	116 kDa U5 small nuclear ribonucleoprotein component	O08810
RAVANRTDAC	11	1795.7	26S proteasome regulatory subunit 7 OS=Mus musculus OX=10090	P46471
CNVDNKDFM	9	1796.9	Inner centromere protein	Q9WU62-2; Q9WU62
RLQRNQETI	9	1801	ATP-dependent RNA helicase DDX50	Q99MJ9
FIHEVNPSSL	10	1820.8	Prosaposin	Q61207
SVMENSKVLGI	13	1830.4	Talin-1	P26039
SQPIAQQPL	9	1833.9	Heterogeneous nuclear ribonucleoprotein Q	Q7TMK9
VHVINVDKV	9	1844.5	Geranylgeranyl transferase type-2 subunit beta	P53612
AALVSKAIDVL	11	1844.7	Protein SOGA1	E1U8D0
LSILNSNEHLL	11	1855.6	Centrosomal protein of 85 kDa	Q8BMK0
RTIVLQESI	9	1855.8	TGF-beta receptor type-1	Q64729; Q64729-2
LCPTNYKYV	9	1876.5	histone-lysine N-methyltransferase EHMT1	Q5DW34
HVYGNQTDPL	10	1876.9	protein GAPT	Q8CB93
FCAVNPRFV	9	1904.8	Coronin-1C	Q9WUM4
YMVKHKSHL	9	1914.5	Parafibromin	Q8JZM7
SCLLHIAYL	9	1918.8	WD repeat-containing protein 81	Q5ND34
TPVLNYDLL	9	1930.7	Calcipressin-2	Q9JHG6-3; Q9JHG2; Q9JHG6-2; Q9JHG6; Q9JHG6
TPVINYDLL	9	1931.2	Calcipressin-2	Q9JHG6-3; Q9JHG2; Q9JHG6-2; Q9JHG6-4; Q9JHG6
NQYTLKDEI	9	1931.4	Calcium/calmodulin-dependent protein kinase kinase 2	Q8C078-2; Q8C078-1; Q8C078-5; Q8C078-4; Q8C078-3

SSPAKQELI	9		1932.8	Sodium- and chloride-dependent betaine transporter	P31651
SMGLSLVEM	9	1xOxidation [M9]	1934.8	Dual specificity mitogen-activated protein kinase kinase 1	P31938
KGGFNRPLDF	11		1938.7	U4/U6.U5 small nuclear ribonucleoprotein 27 kDa protein	Q8K194-1
SARNLEHL	8		1940.8	F-box only protein 38	Q8BMI0
TQSVNIEQL	9		1941.7	NF-kappa-B inhibitor zeta	Q9EST8-3; Q9EST8-2; Q9EST8
QTHENREHL	9		1944.4	Dual specificity protein kinase CLK3	O35492
AACMNQKHL	9		1956	AMP deaminase 3	O08739; Q3V1D3
IMGIGVDTI	9		1959.1	Nucleoporin NUP188 homolog	Q6ZQH8
SMASSLNLPVL	11		1964.8	B-cell lymphoma/leukemia 10	Q9Z0H7
TCISNLNVHL	10		1969.6	Zinc finger protein 672	Q99LH4
SSLTKKEHM	9		1981.2	Transcription initiation factor TFIID subunit 5	Q8C092
FSFGSKNTL	9		1981.9	E3 SUMO-protein ligase RanBP2	Q9ERU9
RQISNIKKEKL	11		1987.5	zinc finger E-box-binding homeobox 2	Q9R0G7
QLVINQEAL	9		1990.2	Guanylate-binding protein 1	Q01514; Q9Z0E6
KTAGNSEFL	9		1997.4	Eukaryotic translation initiation factor 4 gamma 2	Q62448; Q62448-2
NILVNQERL	9		2000.5	Endonuclease/exonuclease/phosphatase family domain-containing protein 1	Q3TGW2
SVGVNLEAF	9		2000.6	Mediator of RNA polymerase II transcription subunit 27	Q9DB40-2; Q9DB40
VATVNKAGSEI	11		2003.1	Microtubule-actin cross-linking factor 1	Q9QXZ0-3; Q9QXZ0-2; Q9QXZ0-4; Q9QXZ0
QSPENVPTDH	11		2018.3	Golgin subfamily A member 2	Q921M4-1
STVRNADVIAG	12		2021.5	multidrug resistance protein 1B	P06795; P21447
VHPQNIPDSPN	11		2026.6	interferon regulatory factor 2-binding protein-like	Q8K3X4
SAVISSIAHYL	11		2032.8	E3 ubiquitin-protein ligase RNF139	Q7TMV1
SSLKDTERI	9		2036	MAX gene-associated protein	A2AWL7-3; A2AWL7
AAPCFLTLRVA	13		2053.4	GPI-anchor transamidase	Q9CXY9
SGVNGTHI	8		2055.7	SRSF protein kinase 1	O70551
SQPESKVFYL	10		2056.7	14-3-3 protein zeta/delta	P63101
SGIHLTIEM	9		2060.8	Plasma membrane calcium-transporting ATPase 1	G5E829
SAVKNINLP	9		2083.9	AP-3 complex subunit sigma-2	Q8BSZ2
FMAEHLEETL	10		2089	Eukaryotic translation initiation factor 4E transporter	Q9EST3; Q9EST3-2
STIINEDASFF	11		2089.4	Cytoplasmic polyadenylation element-binding protein 4	Q7TN98-5; Q7TN98; Q7TN98-2; Q7TN98-3; Q7TN98-4
FVVPNQKEL	9		2090.3	Long-chain-fatty-acid--CoA ligase 3	Q9CZW4
NALLNAGESRI	11		2104.9	serine/threonine-protein kinase ATR	Q9JKK8
KCLKNIHRI	9		2128.3	CASP8 and FADD-like apoptosis regulator OS=Mus musculus OX=10090	O35732
KTGYNVDLL	9		2136.3	Ras-related protein Rab-20	P35295
RTLSNSLQNVI	11		2141.5	Exocyst complex component 6B	A6H5Z3; A6H5Z3-2
GSAVISLEGKP	12		2147.2	Cofilin-1	P18760
ACLINKPEL	9		2153.8	tRNA-dihydrouridine(20a/20b) synthase [NAD(P)+]-like	Q32M08
AMSENIWSTL	10		2161.5	Peroxisomal targeting signal 1 receptor	O09012-2; O09012-1
AQYGNILKHVM	11		2164	Nucleoporin NUP53	Q8R4R6
AQLDNEKTNFI	11		2165.5	Leucine-rich repeat flightless-interacting protein 1	Q3UZ39-2; Q3UZ39-1
AVGVNCEHL	9		2171.9	Transcription elongation factor A protein 2	Q9QVN7
TCVSNGPFIM	10		2185.9	Polypyrimidine tract-binding protein 1	P17225
CQLGNFSIHM	10		2188.2	Very-long-chain enoyl-CoA reductase	Q9CY27
VQAQLGLPPL	10		2192	Transmembrane protein 59	Q9QY73
SVLRLIQVI	9		2193.4	Conserved oligomeric Golgi complex subunit 2	Q921L5
KSPDNHSQTL	10		2195.7	Tyrosine-protein phosphatase non-receptor type 12	P35831
SAPTLEDHF	9		2196.1	microfibrillar-associated protein 1A	C0HKD8

SQLELKSLI	9	2201.3	Mitogen-activated protein kinase 6	Q61532
RMLKLKTQM	9	2206.5	E3 ubiquitin-protein ligase RNF8	Q8VC56
QSNWNPQHL	9	2210.9	Teashirt homolog 1	Q5DTH5; Q8CGV9; Q68FE9
SSPASTPLSPM	11	2212.3	Ribosomal RNA processing protein 1 homolog B	Q91YK2
IGPSSIDLI	9	2213.9	Inactive rhomboid protein 2	Q80WQ6
CADGLIMDNG	13	2216	Otogelin-like protein	F7A4A7-2; F7A4A7
FTSSMRGMDT	11	2233.5	Arginine/serine-rich coiled-coil protein 2	A2RTL5
YRISMADYV	9	2233.9	ATP-dependent 6-phosphofructokinase, liver type	P12382
GACRNLWRSF	11	2238.8	Helicase with zinc finger domain 2	E9QAM5
AQVNNFDLNF	10	2241.9	Excitatory amino acid transporter 1	P56564
FSVEGQENL	9	2270.7	Cap-specific mRNA (nucleoside-2'-O-)-methyltransferase 1	Q9DBC3
SQPIGVTKI	9	2275.1	Cytosolic arginine sensor for mTORC1 subunit 2	Q8CAB8
IPPTNTVDFI	10	2280.5	Protein FAM72A	Q8BFZ8
RGFENVELGVI	11	2284.6	Protein FAM177A1	Q8BR63
RALELEQEL	9	2288.1	Alanine aminotransferase 1	Q8QZR5
AAPETREHL	9	2296.9	Ras-related protein Rab-32	Q9CZE3
ISPKHFVHL	9	2297.2	Glucocorticoid modulatory element-binding protein 1	Q9JL60
AQPSNSLLGEI	11	2300.1	Ribonucleoprotein PTB-binding 1	Q9CW46
SLVTNMDKLHI	11	2300.4	Nck-associated protein 1-like	Q8K1X4
FSHYMEENL	9	2308.4	Serine/threonine-protein kinase PLK2	P53351
FVYVFHTL	8	2310.8	Transmembrane glycoprotein NMB	Q99P91
SCPLFKDYL	9	2314.4	Thiosulfate sulfurtransferase/rhodanese-like domain-containing protein 2	Q3U269
SCPAGYIPL	9	2314.6	Macrophage-expressed gene 1 protein	A1L314
VQPEHIQYL	9	2321.1	structural maintenance of chromosomes flexible hinge domain-containing protein 1	Q6P5D8
SSCMNQKHL	9	2329.8	AMP deaminase 2	Q9DBT5
RTLSNHKSWT	11	2333.6	nucleolar pre-ribosomal-associated protein 1	Q571H0
FGPVDSEQL	9	2333.8	Cyclin-dependent kinase inhibitor 1	P39689
NCILNAEALM	10	2343.8	Prolyl 3-hydroxylase 1	Q3V1T4
KGPLNGDTDY	11	2358.4	Disabled homolog 2	P98078; P98078-2
SLGTNRDDL	9	2368.9	Kinesin-like protein KIF16B	B1AVY7
QCIANQVQL	9	2382.8	ATP synthase mitochondrial F1 complex assembly factor 1	Q811I0
STYSNRDIDGL	11	2394.4	Helicase with zinc finger domain 2	E9QAM5
KQVTSAEHL	9	2396.8	Origin recognition complex subunit 5	Q9WUV0
NAYMLIYRL	9	2397.2	ubiquitin carboxyl-terminal hydrolase 47	Q8BY87-2; Q8BY87-1
SSVKNVPENL\	11	2398.3	aminoacyl tRNA synthase complex-interacting multifunctional protein 2	Q8R010
YLMELGEVI	9	2409.6	HEAT repeat-containing protein 6	Q6P1G0
QAFENGRALVI	12	2417.6	GEM-interacting protein	Q6PGG2; Q6PGG2-2
VSPINFPERF	10	2417.8	Solute carrier family 52, riboflavin transporter, member 2	Q9D8F3; Q9D8F3-2
RMGANSLERM	10	2420.4	Heterogeneous nuclear ribonucleoprotein M	Q9D0E1-2; Q9D0E1
YTIENPRHF	9	2444.1	staphylococcal nuclease domain-containing protein 1	Q78PY7
SILNRQVL	8	2444.3	Nuclear mitotic apparatus protein 1	E9Q7G0
SGIAGFSHL	9	2454.8	U3 small nucleolar RNA-associated protein 18 homolog	Q5SSI6
VHPTNSALNYL	11	2457	Dual specificity protein phosphatase 1	P28563
RAIWNYIHC	9	2464	Sestrin-2	P58043; P58006
GLLENSAHL	9	2465.8	DNA repair and recombination protein RAD54B	Q6PFE3
YGNILKHVM	9	2468.2	Nucleoporin NUP53	Q8R4R6
SVMENSKVLGI	13	2472.7	Talin-2	Q71LX4
YCLKNNKVSVL	11	2476	FYVE, RhoGEF and PH domain-containing protein 6	Q69ZL1

TIGSNVEEI	9	2477.6	ADP-ribosylation factor-like protein 5C	Q6P068; Q9D4P0; Q80ZU0
LGGSAVISLEG	14	2482.2	Cofilin-1	P18760
SAPANGILVPN	13	2483	Interferon regulatory factor 2-binding protein 2	E9Q1P8
YHIGTLQEYL	10	2493.9	fucose-1-phosphate guanylyltransferase	G5E8F4
THLINFSETL	10	2503.2	Cytoplasmic FMR1-interacting protein 1	Q7TMB8
AAPAPAHHDFI	11	2503.9	CCAAT/enhancer-binding protein beta	P28033-3; P28033
AVIRNINDQVL	11	2517.3	Interleukin-18	P70380
QCLENQQLI	9	2542.3	POC1 centriolar protein homolog A OS=Mus musculus OX=10090	Q8JZX3
MGVRNSPQAL	10	2542.5	Serine/threonine-protein kinase TAO1	Q5F2E8
SSNKNSLKDSI	11	2561.5	Sentrin-specific protease 1	P59110
RGPVNLQHLIL	11	2568	Leucine-rich repeat and fibronectin type-III domain-containing protein 4	Q80XU8
SALKSVTSA	9	2568.4	Nuclear fragile X mental retardation-interacting protein 2	Q5F2E7-2; Q5F2E7-1
GGPENTLVF	9	2573.6	KN motif and ankyrin repeat domain-containing protein 2	Q8BX02; Q8BX02-2
FSLKKATFA	9	2575.6	Protein FAM193A	Q8CGI1
FIRNQEQM	8	2576	26S proteasome regulatory subunit 4	P62192
SMLCIPLWI	9	2580.5	Sodium- and chloride-dependent GABA transporter 3	P31650
YVNPNHQATL	10	2588.1	Brefeldin A-inhibited guanine nucleotide-exchange protein 2	A2A5R2
RQVKNPFGLEI	11	2589.8	cell growth regulator with RING finger domain protein 1	Q8BMJ7
SSLELVGHL	9	2591.5	Putative Polycomb group protein ASXL1	P59598
SMLEKTALL	9	2594.2	Sideroflexin-5	Q925N0
SALTHAGAHL	10	2597.9	Splicing factor 3A subunit 3	Q9D554
QPPYNPTYM	9	2601.9	Protein shisa-5	Q9D7I0-1; Q9D7I0-5; Q9D7I0-3; Q9D7I0-2
CAVENGGCSH	11	2603.2	Low-density lipoprotein receptor-related protein 4	Q8VI56
TLLSNFKTHL	10	2624.6	Brefeldin A-inhibited guanine nucleotide-exchange protein 1	G3X9K3
AMGTSTVEI	9	2625	ESF1 homolog	Q3V1V3
KLLALKDFM	9	2628.5	Phosphoinositide 3-kinase regulatory subunit 4	Q8VD65
FTWTGAEHI	9	2633.7	E3 ubiquitin-protein ligase UBR5	Q80TP3
KQCPNAVVL	9	2638	Caseinolytic peptidase B protein homolog	Q60649
RFLENHEKM	9	2643.1	pre-mRNA-processing factor 40 homolog A	Q9R1C7-2; Q9R1C7-1
TSSVNKEQL	9	2660.3	E3 ubiquitin-protein ligase XIAP	Q60989
HCHLNKTSL	9	2662.5	Mono [ADP-ribose] polymerase PARP16	Q7TMM8
AALEKPLSHPM	11	2670.8	Zinc finger MIZ domain-containing protein 1	Q6P1E1-2; Q6P1E1
SMLVSVVGM	9	2692	Serine palmitoyltransferase small subunit A	Q8R207
GALRNLSAC	9	2706.3	HEAT repeat-containing protein 3	Q8BQM4
TGPNNTTNF	9	2716.1	Trinucleotide repeat-containing gene 6A protein	Q3UHK8
VAPANSLVHAI	11	2716.4	DmX-like protein 1	Q6PNC0
GSCINVAPHL	10	2735.7	Lysine-specific demethylase 3B	Q6ZPY7; Q6ZPY7-2
SSCVNDIQHL	10	2737.2	F-box DNA helicase 1	Q8K2I9-1
TVGFNVETV	9	2742	ADP-ribosylation factor 6	P62331
TCPSNLNDL	9	2746.2	Endophilin-B1	Q9JK48-3; Q9JK48; Q9JK48-2
SCLQLAEQI	9	2747	Uncharacterized protein C1orf112 homolog	Q3TQQ9-1
RAIKNGKGL	9	2748.5	staphylococcal nuclease domain-containing protein 1	Q78PY7
GCPQNSTFTEI	12	2749.2	Mini-chromosome maintenance complex-binding protein	Q8R3C0
AMPSLKITNDY	12	2750	Keratinocyte-associated transmembrane protein 2	Q8K201
IIGINGDYF	9	2763.3	T-complex protein 1 subunit alpha	P11983-2; P11983
ICPLNPFLV	9	2765.4	snRNA-activating protein complex subunit 2	Q91XA5
LCVQNILKL	9	2773.4	Mucosa-associated lymphoid tissue lymphoma translocation protein 1 homolog	Q2TBA3-1
RVFQNEVLGTI	11	2782.2	Translation initiation factor eIF-2B subunit epsilon	Q8CHW4

SQLKGFSLL	9		2794.4	Poly [ADP-ribose] polymerase 1	P11103
TGVQNFSRIHL	11		2799.9	Scavenger receptor class B member 1	Q61009; Q61009-2
VHVNRDTL	8		2805.9	Bifunctional polynucleotide phosphatase/kinase	Q9JLV6-1; Q9JLV6-2
RAYAALGLPYM	11		2811.4	CREB-binding protein	P45481
FAHPGMMQEL	10		2827.1	Apolipoprotein B receptor	Q8VBT6
SAIQNLHSFDP	11		2833.4	Eukaryotic translation initiation factor 1	P48024
YSGYSKETL	9		2839.3	Phosphatidylinositol 4-phosphate 3-kinase C2 domain-containing subunit alpha	Q61194; Q61194-2
FQQTATTTM	9		2845.6	Glutamine-rich protein 1	Q3UA37
FTIENVTRT	9		2848.5	RNA-binding protein PNO1	Q9CPS7
CALLAGSEYL	10		2852.7	Eukaryotic translation initiation factor 3 subunit D	O70194
AVHLNRNFITV	12		2854.5	UPF0668 protein C10orf76 homolog	Q6PD19-3; Q6PD19-1
RAGPSLKTTL	10		2863	SIN3-HDAC complex-associated factor	Q8C8M1
RSLSSPTVTL	10		2864.3	E3 ubiquitin-protein ligase NEDD4-like	Q8CFI0-2; Q8CFI0; Q8CFI0-3
RSLLDQENL	9		2882.1	Protein phosphatase Slingshot homolog 1	Q76I79; Q76I79-2
SSITNHINKLDK	12		2898	Semaphorin-5A	Q62217
AAVIDQERL	9		2909.5	Serine/threonine-protein kinase MRCK gamma	Q80UW5
SQKENLNFLHL	11		2918.2	ATPase family AAA domain-containing protein 2	Q8CDM1-2; Q8CDM1
VNVTDHDVI	9		2925.9	Origin recognition complex subunit 3	Q9JK30-2; Q9JK30-1
HAPYDAMVM	9		2940.7	peroxisomal carnitine O-octanoyltransferase	Q9DC50
SVVRNVFDF	9		2941.9	von Willebrand factor A domain-containing protein 8	Q8CC88-2; Q8CC88
FCIKNCHRV	9		2945.8	NACHT, LRR and PYD domains-containing protein 3	Q8R4B8-1
ETIMNQEKL	9	1xOxidation [M4]	2946.4	Transcription factor BTF3	Q64152-2; Q64152
GQLSNGDHHF	10		2954.8	YTH domain-containing family protein 1	P59326
ACHPNLDKL	9		2965.2	NADH-cytochrome b5 reductase 1	Q9DB73
SAPLGDFRHTI	11		2969.2	Cdc42 effector protein 4	Q9JM96
VCHQDMETL	9		2982.3	Thyroid adenoma-associated protein homolog	A8C756-1
SIHENFSQAM	10		2985.9	Coiled-coil domain-containing protein 93	Q7TQK5
TSLKNLRTLHL	11		2986.1	Leucine-rich repeat-containing protein 40	Q9CRC8
SLVSNLLHSTL	11		3000.8	Folliculin-interacting protein 1	Q68FD7
LALLLGDRL	9		3014.2	Monocyte differentiation antigen CD14	P10810
FQYWKRFDL	9		3016.5	Isoform 3 of Homeobox protein cut-like 1	P53564-3
NCGVNAVEL	9		3018.6	Protein kinase C eta type	P23298
RQPKHLLAFL	10		3020.5	Eukaryotic translation initiation factor 2 subunit 2	Q99L45
YQFASGAFLHI	11		3039.7	Programmed cell death 6-interacting protein	Q9WU78-3; Q9WU78
NCFINRQLI	9		3041.7	T-complex protein 1 subunit beta	P80314
AVCKNTITL	9		3065.1	Isoform 3 of E3 ubiquitin-protein ligase RBBP6	P97868-3
YAVRNLTEDNS	11		3109.5	Ataxin-10	P28658
SQNVPSTNYL	10		3128.2	Lysine-specific demethylase 3B	Q6ZPY7; Q6ZPY7-2
FCPHNEQIL	9		3174.2	Breast cancer type 2 susceptibility protein homolog	P97929
FKAKNLIEVM	10		3174.4	Sorting and assembly machinery component 50 homolog	Q8BGH2
KLIRLMEEI	9		3181.4	probable ATP-dependent RNA helicase DDX5	Q61656
GIENIHVM	8		3194.5	Myotubularin-related protein 6	Q9Z2C9; Q8VE11
TPVKNIDTV	9		3206.5	Sister chromatid cohesion protein PDS5 homolog A	Q6A026
VTIILKEPV	9		3210.8	Ribosome-binding protein 1	Q99PL5-10; Q99PL5-11; Q99PL5-6; Q99PL5-12; Q99PL5-4; Q99PL5-9; Q99PL5-7; Q99PL5-1; Q99PL5-8; Q99PL5-5; Q99PL5-3; Q99PL5-2
VGLYLREHI	9		3211.8	Kinesin-like protein KIF15	Q6P9L6
SAPAFGPEAL	10		3226.5	Proteasome subunit beta type-6	Q60692

SALALRWEA	9	3231.1	A-kinase anchor protein 2	O54931-4; O54931-2; O54931-5; O54931
AVCENFQEFL	10	3233.9	apoptotic protease-activating factor 1	O88879-1
SHFLNDTCLE\	12	3236	F-box/LRR-repeat protein 4	Q8BH70
SALVLVKNSEN	11	3239	Mast cell-expressed membrane protein 1	Q9D8U6
VTPKHQEYL	9	3242.6	Inhibitor of nuclear factor kappa-B kinase subunit epsilon	Q9R0T8
GQWTNKMEF\	11	3243.4	Sodium- and chloride-dependent betaine transporter	P31651
VQVENPERF	9	3247.9	Tripartite motif-containing protein 35	Q8C006
SQPVAVSHI	9	3257.5	Max-binding protein MNT	O08789
HSLQGSHIL	9	3267.9	Protein Daple	Q6VGS5; Q6VGS5-2
YQYKGMGLSM	10	3268.5	proteasome subunit beta type-5	O55234
TSGVNKESF	9	3294.1	Round spermatid basic protein 1	Q80T69
WCPHNDEVI	9	3308.6	Coronin-1B	Q9WUM3
AMFSGRMEVL	10	3318.4	BTB/POZ domain-containing adapter for CUL3-mediated RhoA degradation protein 3	O70479; Q922M3
GPKNYEFL	8	3368.5	Sorting nexin-14	Q8BHY8
SCGLNTSSL	9	3381.8	Exonuclease 1	Q9QZ11
SSPRNSQELSI	12	3390.2	caspase recruitment domain-containing protein 9	A2AIV8
YQALPCLPSM	10	3403	Coatomer subunit alpha	Q8CIE6
LSTKGLTYL	9	3434.6	Vacuolar protein sorting-associated protein 13B	Q80TY5
SAPRPSSQFV	10	3442.7	Golgin subfamily A member 5	Q9QYE6
VQYPNSINLTL	11	3455.7	Laminin subunit gamma-1	P02468
KSIVNTSPPC	10	3478.5	RNA-binding protein 33	Q9CXK9-1
KGFSDKLDFL	10	3505.3	Probable rRNA-processing protein EBP2	Q9D903
AAPRGPGLFL	10	3525.4	N-acetylglucosamine-1-phosphodiester alpha-N-acetylglucosaminidase	Q8BJ48
SAPLPPSIFM	10	3527.4	Farnesyl pyrophosphate synthase	Q920E5
IMGVGQCVI	9	3534.6	Long-chain fatty acid transport protein 1	Q60714
WQYPNGTHA	9	3539.9	probable JmjC domain-containing histone demethylation protein 2C	Q69ZK6
ICPNNHEVHI	10	3542.4	Actin-related protein 2/3 complex subunit 1B	Q9WV32
SCVKSLDVL	9	3542.9	ATP-dependent RNA helicase DDX55	Q6ZPL9
GKLSNQERI	9	3561.1	Ataxin-10	P28658
KVAVNDAHL	9	3579.3	Galectin-3	P16110
SGPWNRGFG(	12	3580.2	Protein SCAF8	Q6DID3
KGPENTLRV	9	3583.1	E3 ubiquitin-protein ligase MYCBP2 OS=Mus musculus OX=10090	Q7TPH6; Q7TPH6-2; Q7TPH6
TAPTHVPLQYI	11	3607.1	Serine--tRNA ligase, mitochondrial	Q9JJL8
SCPQGLVHI	9	3607.8	StAR-related lipid transfer protein 9	Q80TF6
SALRLKQRL	9	3614.9	E3 ubiquitin-protein ligase HERC2	Q4U2R1-2; Q4U2R1
FCGKNGLSL	9	3624	Ubiquitin carboxyl-terminal hydrolase 34	Q6ZQ93; Q6ZQ93-3; Q6ZQ93-4; Q6ZQ93-2
AQGVSIEAM	9	3666.9	INO80 complex subunit C	Q8BHA0
QIVNPHLL	8	3670.5	Ribonucleoside-diphosphate reductase large subunit	P07742
LCLINYNRTV	10	3683.4	Prolow-density lipoprotein receptor-related protein 1	Q91ZX7
RCLHNFTSSL	10	3689.6	Origin recognition complex subunit 3	Q9JK30-1
FHMAMGNPSE	11	3693.3	Segment polarity protein dishevelled homolog DVL-2	Q60838
HQHEPFLDFL	10	3717.1	NF-kappa-B inhibitor beta	Q60778
AAPVSGPRL	9	3734.5	protein LTV1 homolog	Q6NSQ7
TGPSNQWASE	11	3734.5	GRB10-interacting GYF protein 2	Q6Y7W8-2; Q6Y7W8-1
ASCMMLDHL	9	3752.8	Isocitrate dehydrogenase [NAD] subunit gamma 1, mitochondrial	P70404
KCIVLVTEL	9	3752.8	Serine/threonine-protein kinase WNK1	P83741; Q80XP9-1
GMVVDVENL	9	3772.9	Histone acetyltransferase KAT2A	Q9JHD2
YQLAHQISVTL	11	3780.2	Threonine--tRNA ligase, mitochondrial	Q3UQ84

FMHGKIPTL	9		3791.5	Pre-mRNA-processing-splicing factor 8	Q99PV0
AMGTAWRDYI	10		3799.5	Phospholipase D2	P97813
CSVRDQELL	9		3823.8	breast cancer type 1 susceptibility protein homolog	P48754
KMKVRFSFFL	10	1xOxidation [M2]	3825.7	Very-long-chain (3R)-3-hydroxyacyl-CoA dehydratase 3	Q8K2C9
SQLTGLGTAV	10		3828.5	Protocadherin Fat 4	Q2PZL6
GCFKNSQVL	9		3830.8	C-type lectin domain family 4 member E	Q9R0Q8
GLRNVDCL	8		3847.7	Serine/threonine-protein kinase TBK1	Q9WUN2
IQYSKHPPPL	10		3866.9	sequestosome-1	Q64337; Q64337-2
FAPKSWMEDS	11		3869	Cytoplasmic polyadenylation element-binding protein 4	Q7TN98-5; Q7TN98-1; Q7TN98-2; Q7TN98-3; Q7TN98-4
YTQINVGNL	9		3958.6	Probable ATP-dependent RNA helicase DDX17	Q501J6; Q501J6-2
SLYTNAKGGTI	11		3970.5	Protein max	P28574; P28574-2
SRLELVDYL	9		3982.7	FAST kinase domain-containing protein 3, mitochondrial	Q8BSN9
TGIVNHTHS	9		3997	Histone-arginine methyltransferase CARM1	Q9WVG6-1
RCFENTFQEL	10		4004.3	PAB-dependent poly(A)-specific ribonuclease subunit PAN3	Q640Q5-1
ISPHNNQHF	9		4015.4	Neurofibromin	Q04690-3; Q04690; Q04690-4; Q04690-2
NQMPNAVQKL	10		4030.6	HMG box-containing protein 1	Q8R316-2; Q8R316-1
QGIENSPYVHL	11		4053.5	Protein RMD5 homolog A	Q80YQ8
FSSLNLRETNL	11		4061.7	Vimentin	P20152
CQYQAPDMDT	11		4072	NF-kappa-B essential modulator	O88522
VIGRNGEMI	9		4083.5	Far upstream element-binding protein 1	Q91WJ8-2; Q91WJ8
RCIHNSKIVM	10		4113	F-box only protein 10	Q7TQF2
SCPYGTVYL	9		4116.9	CREB-regulated transcription coactivator 1	Q68ED7
SCIKNPKILL	10		4124.3	1-phosphatidylinositol 3-phosphate 5-kinase	Q9Z1T6
GMYSGHGKLE	12		4128.8	BMP and activin membrane-bound inhibitor homolog	Q9D0L6
SAQILASAL	9		4141.7	Matrix metalloproteinase-15	O54732
FQSISTEFL	9		4167.9	Cytoplasmic dynein 1 heavy chain 1	Q9JHU4
SVLSHVESL	9		4170.2	ADP-ribose glycohydrolase ARH3	Q8CG72
SSLGKHSTM	9		4188.7	protein THEMIS2	Q91YX0
TCAINNTLI	9		4196.7	Phosphatidylinositol glycan anchor biosynthesis class U protein	Q8K358
AAIRNSITSC	10		4205.3	Forkhead box protein N3 OS=Mus musculus OX=10090	Q499D0
ACAINKVLM	9		4211.6	Protein FAM98B	Q80VD1; Q3TJZ6
LRHVNIDHL	9		4216.3	Eukaryotic translation initiation factor 3 subunit H	Q91WK2
SGLLLKQHI	9		4217.2	Paraplegin	Q3ULF4
WCPHNDNVI	9		4248	Coronin-1A	Q920M5-1; O89053
VQCINCEKL	9		4269.8	NG finger and CHY zinc finger domain-containing protein 1 OS=Mus musculus OX=1009	Q9CR50
NCGANVDLL	9		4276.6	Cell division control protein 45 homolog	Q9Z1X9
SVLNVNHI	8		4288.9	Ankyrin repeat domain-containing protein 17	Q99NH0-1
YMTEGHLAM	9	1xOxidation [M9]	4318.3	dual specificity tyrosine-phosphorylation-regulated kinase 1A	Q61214
ATPGRLIDFL	10		4326.3	Probable ATP-dependent RNA helicase DDX17	Q501J6; Q501J6-2; Q61656
FGLSARDLDEL	11		4328.4	Matrin-3	Q8K310
RSPENPPSKEL	11		4404.4	Centromere protein M	Q9CQA0-1
KMRLLTFM	8	2xOxidation [M2; M8	4412.6	Eukaryotic translation initiation factor 3 subunit M	Q99JX4
FAIQNKHLCH	10		4415.1	Cyclic AMP-dependent transcription factor ATF-3	Q60765
FSFQPPATVHI	13		4425.8	Far upstream element-binding protein 2	Q3U0V1
SNYEKYYLI	9		4440	phosphatidylinositol 4-phosphate 3-kinase C2 domain-containing subunit alpha	Q61194-1; Q61194-2
KMLDKYSHYL	10		4444.8	dedicator of cytokinesis protein 11	A2AF47
SAMSNPRAMC	11		4471.2	Isoform 2 of Ubiquilin-1	Q8R317-2; Q8R317

YCCKNHTRAY	11		4477.4	Queuine tRNA-ribosyltransferase accessory subunit 2 OS=Mus musculus OX=10090	B8ZXI1
RGPENLLDHQ	11		4508.5	Melanoma inhibitory activity protein 2	Q91ZV0; Q8R311
WHNLNYWQL	9		4528.9	peroxisomal carnitine O-octanoyltransferase	Q9DC50
RNLTNQLGLL	10		4540.2	Myeloperoxidase	P11247
KSVISVIHL	9		4543	CDP-diacylglycerol--inositol 3-phosphatidyltransferase	Q8VDP6
AAVTGHIRI	9		4568.2	Coatomer subunit beta	Q9JIF7
YGWTANMERI	11		4571.8	Heat shock protein HSP 90-alpha	P07901; P11499
NMPGVMTGTM	10		4594.2	Clathrin interactor 1	Q99KN9-1
RHAAVLVETI	10		4595.2	CLIP-associating protein 2	Q8BRT1
SSVKNEEQFVI	14		4602.6	Maternal embryonic leucine zipper kinase	Q61846
SMMVKQEGM	9	1xOxidation [M3]	4608.5	Hyaluronan mediated motility receptor	Q00547-2; Q00547-1
RAPRNWNKP	9		4613	Arginine vasopressin-induced protein 1	Q9D7H4
GLKNLTAL	8		4619	Phosphoinositide 3-kinase adapter protein 1	Q9EQ32-3; Q9EQ32-2; Q9EQ32-1
MAPENGYLME	11		4640.9	U1 small nuclear ribonucleoprotein 70 kDa	Q62376-1
KLMELLEEI	9		4655.1	N-alpha-acetyltransferase 20	P61600
NCILDKDYL	9		4659.7	DNA repair protein RAD52 homolog	P43352
GNLSNFLIHL	10		4674.4	Protein YIPF1	Q91VU1
SSCVNDIQHLL	11		4692.9	F-box DNA helicase 1	Q8K2I9-1
CSVKNEAHA	9		4719.9	E3 SUMO-protein ligase RanBP2	Q9ERU9
RNKENQETL	9		4722.1	STE20-like serine/threonine-protein kinase	O54988-2; O54988-1
TAVEDSDKAYI	11		4741.9	Proline-serine-threonine phosphatase-interacting protein 2	Q99M15
ECHLNADTV	9		4742.6	AP-2 complex subunit beta	Q9DBG3
TIGFNVETV	9		4769	ADP-ribosylation factor 2	P61211; P61750; P61205; P84078; Q8BGX0-2; Q8BSL7; Q8BGX0-3; Q8BGX0; P84084
AAPGSKDHL	9		4782.6	Transmembrane protein 41B	Q8K1A5
SCLKLQRIYM	10		4787.5	F-box/LRR-repeat protein 17	Q9QZN1-1
YLGSNHISSI	10		4821.8	CD180 antigen	Q62192
VQPTHVIHL	9		4828.6	GDP-L-fucose synthase	P23591
SSFSMEEGDV	11		4830.2	Transcription factor EB	Q9R210
KCLELFTEL	9		4839.1	Heat shock protein HSP 90-alpha	P07901
GMLEKIDMI	9		4841.9	RNA-binding protein 12	Q8R4X3
VQYTLPDGSTI	11		4846.5	Beta-centractin	Q8R5C5
LSPEQLQYL	9		4878.8	Xaa-Pro aminopeptidase 2	B1AVD1
KLKINIDVL	9		4890.4	Endoplasmic reticulum-Golgi intermediate compartment protein 3	Q9CQE7-2; Q9CQE7
FGLQHLSSGHI	11		4916.6	Adenylate kinase 4, mitochondrial	Q9WUR9
HCYIGLPFL	9		4924.9	Transmembrane protein 131	O70472
SRVPMFPVPM	10		4937.2	Protein transport protein Sec16A OS=Mus musculus OX=10090	E9QAT4
RAHENRVKDM	13		4943.6	p21-activated protein kinase-interacting protein 1	Q9DCE5
SVCTNVPGSYI	11		4948.8	Adhesion G protein-coupled receptor E1	Q61549
SQYRFTVSDL	10		4952.9	Cyclin-G1	P51945
ACYNNQQVF	9		4965.9	Squalene synthase	P53798
